# Development of a single-tube one-step RT-LAMP assay to detect the Chikungunya virus genome

**DOI:** 10.1371/journal.pntd.0006448

**Published:** 2018-05-29

**Authors:** Benjamin Lopez-Jimena, Stefanie Wehner, Graham Harold, Mohammed Bakheit, Sieghard Frischmann, Michaël Bekaert, Oumar Faye, Amadou Alpha Sall, Manfred Weidmann

**Affiliations:** 1 Institute of Aquaculture, School of Natural Sciences, University of Stirling, Stirling, Scotland, United Kingdom; 2 MAST Diagnostica GmbH, Reinfeld, Germany; 3 Arbovirus and viral haemorrhagic fever unit, Institut Pasteur de Dakar, Dakar, Senegal; Baylor College of Medicine, UNITED STATES

## Abstract

**Background:**

A single-tube one-step real-time reverse transcription loop-mediated isothermal amplification (RT-LAMP) assay for rapid detection of chikungunya virus (CHIKV) targeting the conserved 6K-E1 target region was developed. The assay was validated with sera collected from a CHIKV outbreak in Senegal in 2015.

**Methodology/Principal findings:**

A novel design approach by combining Principal Component Analysis and phylogenetic analysis of 110 available CHIKV sequences and the LAMP oligonucleotide design software LAVA was used. The assay was evaluated with an External Quality Assessment panel from the European Network for Diagnostics of "Imported" Viral Diseases and was shown to be sensitive and specific and did not cross-detect other arboviruses. The limit of detection as determined by probit analysis, was 163 molecules, and 100% reproducibility in the assays was obtained for 10^3^ molecules (7/8 repetitions were positive for 10^2^ molecules). The assay was validated using 35 RNA samples extracted from sera, and results were compared with those obtained by quantitative RT-PCR carried out at the Institut Pasteur Dakar, demonstrating that the RT-LAMP is 100% sensitive and 80% specific, with a positive predictive value of 97% and negative predictive value of 100%.

**Conclusions/Significance:**

The RT-LAMP appeared to show superior performance with material stored for months compared to qRT-PCR and can be therefore recommended for use in infrastructures with poor settings.

## Author summary

Current chikungunya virus (CHIKV) outbreaks highlight the necessity of sensitive techniques to allow the virus detection even at an early stage (before the onset of clinical symptoms). In addition, CHIKV sometimes is misdiagnosed with other pathogens (i.e., dengue virus or malaria), which implies that specific methods have to be developed. Apart from specificity and sensitivity, these techniques have to be affordable for laboratories with very limited resources, and reactions should be easily performed without the need of experienced researchers and expensive equipment. Finally, because of the increase in number of publicly available sequences, the assay should cover all the possible variations observed in those sequences. We have considered all these premises, and we were able to develop a reverse transcription loop-mediated isothermal amplification (RT-LAMP) by designing primers using a combination of Principal Component Analysis, phylogenetic analysis and LAVA algorithm. Our assay is specific and does not cross-react with other arboviruses tested, sensitive and has been validated at the Institut Pasteur Dakar, showing very good performance parameters.

## Introduction

Chikungunya virus (CHIKV), a single-stranded positive-sense enveloped RNA virus belonging to the genus *Alphavirus*, family *Togaviridae*, was originally isolated in Tanzania in 1953 [1]. The RNA genome (approximately 12 kb), capped at the 5’ and with a poly(A) tail at 3’ end, comprises two open reading frames (ORFs) interrupted by an untranslated region, the junction region (J). The ORF at the 5’ end encodes four non-structural proteins (nsP1, nsP2, nsP3 and nsP4) and the other ORF encodes five structural proteins, including the capsid (C), envelope 3 (E3), E2, 6K and E1 [2]. This virus is clustered into three major distinct genotypes based on phylogenetic analysis of the E1 gene sequence: Asian, East/Central/South African and West African [3–5], and these clusters were also obtained when using full-genome sequences [6], dividing the East/Central/South African clade into three subgroups (I-II-III). CHIKV is the causative agent of Chikungunya fever, an arthropod-borne viral disease transmitted by the mosquitoes *Aedes aegypti* and *A*. *albopictus*, characterised by a sudden onset of fever, headache, fatigue, nausea, vomiting, rash, myalgia and severe arthralgia, with polyarthralgia as the typical clinical sign of the disease which can persist for several months [7]. Recently, another mosquito belonging to the genus *Aedes*, *A*. *hensilli* Farner, was described as the most important vector of CHIKV during the outbreak on Yap Island, Federal States of Micronesia in 2013 [8].

Clinical symptoms are similar to those observed in other diseases, such as malaria and dengue fever [9, 10], but the prognoses of these infections are greatly different and a number of Chikungunya fever cases are commonly misdiagnosed as dengue virus (DENV) infections. In addition, dual infections with CHIKV and DENV have been reported [11].

Because of the lack of antiviral treatments for Chikungunya fever and the malaria- and dengue-like symptoms, accurate, specific and sensitive methodologies are needed in order to provide a definite diagnosis. Although virus isolation from blood of viraemic patients, infected tissues or blood-feeding arthropods is considered the gold standard for CHIKV detection, it is time-consuming, needing at least 7 days. Immunofluorescence assays for CHIKV detection require materials that may not be easily available in diagnostic laboratories worldwide and the performance of laboratories shows great variability [12, 13]. Other serological techniques are based on enzyme-linked immunosorbent assay (ELISA) and immunochromatography for rapid detection, with better sensitivity and specificity achieved by the ELISA, although there are some issues with false-negative and false-positive results [14].

Detection of viral RNA is a sensitive method for diagnosis in acute stages when antibody levels are not high [15]. Several reverse transcription (RT) PCR-based methods have been developed [15–23]. However, these methodologies need sophisticated and expensive equipment that may not be present in laboratories with limited resources. Currently, cost-effective techniques based on the RT loop-mediated isothermal amplification (LAMP) have emerged to substitute PCR because of its simplicity, rapidity, specificity and sensitivity, showing that only a heating block or water bath capable to maintain a constant temperature (60°C to 65°C) is needed [24–26]. Furthermore, reactions could be visualized by monitoring either the turbidity in a photometer or the fluorescence in a fluorimeter, by naked eye under a UV lamp when using an intercalating dye and by colour change [25–29]. As a matter of fact, RT-LAMP assays amplifying the CHIKV structural E1 gene have been previously developed [27, 29]. However, these previous RT-LAMP assays date from 2007 and 2012, and the information about the primer design is limited. In this study, we downloaded 110 CHIKV sequences from the NCBI database, and used LAMP Assay Versatile Analysis (LAVA) algorithm [30] to derive LAMP primers for an already known conserved CHIKV genome region (6K-E1) to cover all possible circulating CHIKV strains. This methodology allowed designing a highly sensitive and specific single-tube one-step real-time RT-LAMP for the detection of CHIKV RNA.

## Materials and methods

### Development of quantitative RNA standard

An RNA standard was transcribed from the CHIKV 6K-E1 region, ligated into pCRII and evaluated as previously published [31].

These serially 10-fold dilutions were used as templates for absolute one-step quantitative RT-PCR (qRT-PCR) using the Light Cycler 480 Master Hydrolisis Probes (Roche, Mannheim, Germany) in 20 μL reaction volume containing 1x LightCycler 480 RNA Master Hydrolysis Probes, 3.25 mM activator Mn(OAc)_2_, 500 nM primers CHIKMW FP1 (5’-YGAYCAYGCMGWCACAG-3’) and CHIKMW RP1 (5’-AARGGYGGGTAGTCCATGTT-3’), 200 nM probe TaqMan probe CHIK P2 (5’-6FAM-CCAATGTCYTCMGCCTGGACRCCKTT—TMR-3’) [32], and 1 μL RNA as template. This assay was repeated 8 times. The qRT-PCR reactions were run in the LightCycler 2.0 (Roche), as follows: reverse transcription for 3 min at 63°C, activation for 30 s at 95°C, followed by 45 cycles consisting of amplification for 5 s at 95°C and 15 s at 60°C. Finally, a cooling step was added of 40 s at 40°C. Analysis of the reactions was conducted using LightCycler software version 4.1.1.21 (Roche).

The specificity was evaluated using the ENIVD External Quality Assessment (EQA) panel provided by the Robert Koch Institute (Berlin, Germany) (Table 1), that includes 12 serum samples (volume per sample, 100 μL) consisting of 3 CHIKV strains (1 of them with different concentrations), 1 DENV2 strain, 2 related alphaviruses (O’nyong-nyong virus—ONNV—and Sindbis virus—SINDV -) and 2 samples of human plasma as negative controls [33]. Viral RNA was extracted from these serum samples using the QIAamp Viral RNA mini kit (QIAGEN, Courtaboeuf, France) and the analysis of these RNA samples was repeated twice.

**Table 1:** Specificity of the RT-LAMP assay.

		qRT-PCR		RT-LAMP		
Virus	Sample identification (copies/mL)	C_P_ values	C_P_ values	Genie II		ESE-Quant Tube Scanner
				min:s	Annealing (^o^C)	min
CHIKV-PCR-EQA 07–2014 ENIVD	#10 CHIK Caribbean (2.2x10^4^)	nd^a^	nd	58:00	83.26	59.7
	#12 CHIK Caribbean (1.3x10^5^)	34.7	32.59	nd	nd	26.7
	#4 CHIK Caribbean (1.8x10^6^)	32.66	35.9	41:30	84.66	26.3
	#9 CHIK Caribbean (2.4x10^7^)	28.78	nd	32:30	84.76	23.3
	#2 CHIK Caribbean (1.8x10^8^)	25.94	26.6	28:45	84.74	20.7
	#6 CHIK India (1.1x10^8^)	25.51	26.85	30:45	86.1	22.7
	#7 CHIK Seychelles (2x10^8^)	25.37	26.88	31:15	85.95	23.3
	#11 ONNV (1.8x10^4^)	nd	nd	nd	nd	nd
	#8 DENV2 (8.3x10^4^)	nd	nd	nd	nd	nd
	#1 SINDV (7.5x10^6^)	nd	nd	nd	nd	nd
	#3 negative (plasma)	nd	nd	nd	nd	nd
	#5 negative (plasma)	nd	nd	nd	nd	nd
DENV	ATCC VR-344, DENV1	nd	nd	nd	nd	nd
	ATCC VR-345, DENV2	nd	nd	nd	nd	nd
	ATCC VR-1256, DENV3	nd	nd	nd	nd	nd
	ATCC VR-1257, DENV4	nd	nd	nd	nd	nd
YFV	YFV	nd	nd	nd	nd	nd
WNV	WNV	nd	nd	nd	nd	nd
NTAV	NTAV	nd	nd	nd	nd	nd
SAV	Salmonid alphavirus, F02-143	nd	nd	nd	nd	nd

^a^ non-detected

### LAMP primer design

A total number of 110 sequences available of the Chikungunya virus (S1 File) were downloaded from the NCBI database. BLAST (v.2.2.28+) [34] was used to identify the 6K-E1 target region within these sequences. For the following analysis steps an alignment of these target regions was calculated by GramAlign v3.0 [35]. The sequences were split into different subgroups with the help of Principal Component Analysis (PCA) of R/adegenet v2.0.0 [36] and phylogenetic tree (Neighbor-Joining tree using the R/ape 3.2 package) as the generation of LAMP primers for all sequences at once was not possible. LAMP DNA signatures for each subset were designed by a modified version [https://github.com/pseudogene/lava-dna] of LAVA version 0.1 [30] applying *loose* parameters. It was checked for all combinations of the subgroups, if a combined primer generation was possible.

All the designed sets of primers were additionally checked for primer dimerisation with the VisualOMP version 7.8.42.0 (DNA Software, Ann Arbor, MI). In addition, primer combinations were tested for primer dimerisation by comparing amplification signals in positive and negative controls. Table 2 includes the final list of primers for the RT-LAMP CHIKV assay, consisting of 17 unique primers (3 amplicons).

**Table 2:** Primers designed for the RT-LAMP.

Clusters	Location 6K-E1 (nt)	Oligo name	Oligo sequence (5’-3’)
1 & 3	18–384	0F3*	TCCCGAACACGGTGGGAGTACC
		0B3	GCGGATGCGGTATGAGCCCT
		0FIP	ACCGCAGCATTTCACGTACGGAAGTCAACAGACCGGGCTACAGC
		0BIP	AGGTCTTCACCGGCGTCTACCCCGGACTTCTCCACATGTGCTTCG
		0FLOOP	TCGCACGTGATGTAATCAAGCGA
		0BLOOP	GGCGGCGCCTACTGCTTCTG
3 & 4	18–492	1F3*	TCCCGAACACGGTGGGAGTACC
		1B3	GGCCCCACAATGAATTTGGCGT
		1FIP	GGGTAGACGCCGGTGAAGACCTCGCTTGATTACATCACGTGCGA
		1BIP	ACTGCTTCTGCGACGCTGAAAACTGTGACGGCATGGTCGCC
		1FLOOP	CCTTGCACTCTGCTGTACCGCA
		1BLOOP	GCATCAGCTAAGCTCCGCGTCC
2	1084–1305	2F3	CGCCGAGTTTCGCGTGCAAG
		2B3	AACGACACGCATAGCACCACA
		2FIP	GGGTGGTGTGTGATGCTGGGTGCTCCACACAAGTACACTGCGCA
		2BIP	GGGTCCAGGATATATCCACAACGGCAAGGCAGCAACAGCAACAATTAATCCT
		2FLOOP	ATGTGGTCCTTTGGAGGGTGGCA
		2BLOOP	TGGGTGCAGAAGATTACGGGAGGA

* 0F3 (clusters 1 & 3) and 1F3 (clusters 3 & 4) primers are identical.

### One-step real-time RT-LAMP

RT-LAMP reactions were run at 64°C for 60 min using either an ESE-Quant TubeScanner (QIAGEN Lake Constance GmbH, Stockach, Germany) or Genie II (Optigene, Horsham, UK), in a final reaction volume of 25 μL. The Genie II device allows to create an annealing curve for confirmation of amplification specificity by an additional heating and cooling step from 98°C to 80°C (0.05°C/s) for 6 min to allow the re-annealing of the amplified product.

Each reaction consisted of 1x RM Trehalose, 6 mM MgSO_4_, 5% polyethylene glycol (PEG), 1 μL fluorochrome dye (FD), 0.1 μM F3, 0.1 μM B3, 0.8 μM FIP, 0.8 μM BIP, 0.4 μM FLOOP, 0.4 μM BLOOP (final concentration for each set of primers), 8 U *Bst* 2.0 DNA Polymerase, 10 U Transcriptor Reverse Transcriptase and 1 μL template (RNA or H_2_O as negative control). Before adding *Bst* 2.0 DNA Polymerase, Transcriptor Reverse Transcriptase and template, mixes were incubated at 95°C for 5 min to melt any primer multimers and cooled immediately on ice for 5 min.

RM Trehalose, MgSO_4_, PEG and FD were supplied by MAST Diagnostica GmbH (Reinfeld, Germany). *Bst* 2.0 DNA Polymerase and Transcriptor Reverse Transcriptase were obtained from New England BioLabs (Hitchin, Herts, UK) and Roche, respectively.

### Determination of the analytical specificity and sensitivity

The analytical specificity of the RT-LAMP assay was evaluated using the EQA panel detailed above [33], repeating the assay in both the Genie II and ESE-Quant TubeScanner. In order to evaluate the specificity of the RT-LAMP protocol developed to detect CHIKV, other RNA viruses were tested (Table 1) including flaviviruses such as, dengue virus (DENV, serotypes 1 to 4), yellow fever virus (YFV), West Nile virus (WNV) and Ntaya virus (NTAV), and other alphaviruses such as salmonid alphavirus (SAV). DENV strains were provided by ENIVD / Robert Koch Institute. Flavivirus (YFV, WNV and NTAV) were provided by M. Weidmann, whilst SAV was provided by B. Lopez-Jimena.

The analytical sensitivity of the CHIKV RT-LAMP assay was tested using the ESE-Quant TubeScanner and the CHIKV RNA standard ranging from10^5^ to 10 molecules/μL, in 8 independent runs. The values obtained were subjected to probit analysis (Statgraphics plus v5.1, Statistical Graphics Corp., Princeton, NJ) and the limit of detection at 95% probability was obtained.

### Validation with clinical samples

Thirty-three qRT-PCR positive and 2 qRT-PCR negative serum samples from a recent CHIKV outbreak in Senegal (2015) were collected and analysed in triplicates at the Institute Pasteur Dakar (IPD) (S1 Table). The IPD has ethical approval for use of these anonymized samples for retrospective studies by the Ministry of Health of Senegal. RNA extractions were performed with the QIAamp Viral RNA mini kit and RNA samples were stored at -80°C.

The RNA samples were analysed by qRT-PCR, as previously described [17]. In addition, CHIKV RT-LAMP reactions were run at 64°C for 60 min in an ABI7500 Fast Real-time PCR system (Applied Biosystems, Foster City, CA).

Sensitivity, specificity, positive predictive value (PPV) and negative predictive value (NPV) were obtained for the developed RT-LAMP when compared against the results obtained by qRT-PCR.

## Results

### Quantitative RNA standard

**Fig 1:**
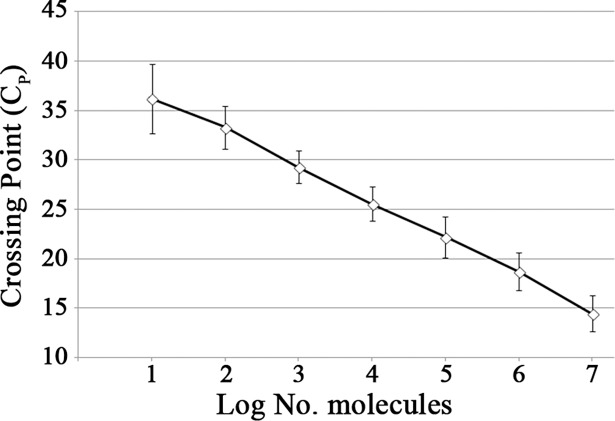
CHIKV RNA standard curve quantified by absolute one-step qRT-PCR.

The molecular RNA standard (10^7^−10 molecules/μL) was evaluated using a one-step qRT-PCR, and this assay was repeated 8 times. Fig 1 shows the mean C_P_ value ± standard deviation (SD). This qRT-PCR showed 100% reproducibility, as positive results were obtained for all the dilutions tested and for all the 8 independent runs. It detected 6 samples (numbers 2, 4, 6, 7, 9 and 12) (Table 1) of the ENIVD EQA panel. Sample number 9, was detected in only 1 of 2 cases.

### LAMP amplicon design

**Fig 2:**
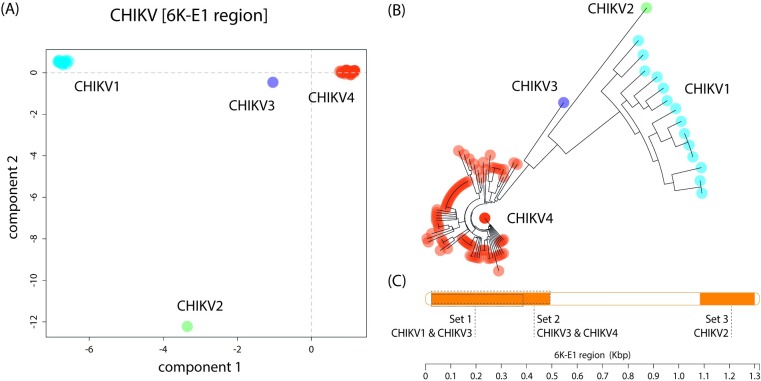
LAMP primer design. (A) PCA of the CHIKV data. (B) Neighbour joining tree of CHIKV data. (C) Primer sets location of the different groups within the CHIKV genome, designed with LAVA software. Note that groups 1–3 and 3–4 have been combined to reduce the number of primers in the LAMP reaction.

The sequences retrieved from GenBank were split into 4 groups, 2 of which consisted of only 1 viral strain, namely CHIKV strain HD 180760 (HM045817|2005|Senegal, group 2) and CHIKV isolate CHIKV STMWG01 (KJ679577|2011|India, group 3) (Fig 2A and 2B). LAVA, the program for LAMP signature design, was executed for each group separately as well as for all possible combinations of the groups. A combined design of LAMP primers was possible for the groups 1 and 3 and groups 3 and 4. The final selection of primer sets is included in Fig 2C and Table 2.

### Analytical specificity of the RT-LAMP

**Fig 3:**
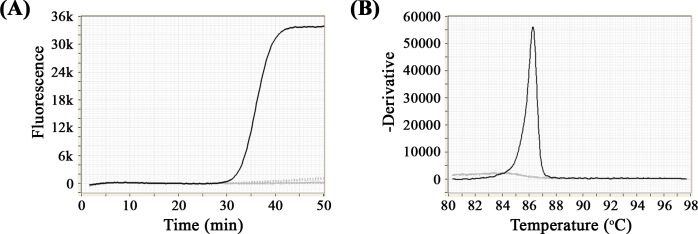
Specificity of the RT-LAMP assay to detect CHIKV RNA (black line). There was no amplification of the negative control (continuous grey line) and RNA of other viruses assayed (discontinuous grey lines). (A) shows the amplification profile for the RT-LAMP, and (B) represents the annealing curve for specificity.

The RT-LAMP protocol developed was specific to detect CHIKV RNA, and no signal was detected when using RNA of other viruses (Fig 3A). In addition, the annealing curve showed a single temperature peak at 86.3°C (Fig 3B).

All serum samples of the ENIVD EQA panel detected by qRT-PCR were also detected by RT-LAMP on the 2 LAMP devices, except for RNA sample number 12 which was only detected with the ESE-Quant TubeScanner (Table 1). In addition, RNA sample number 10, negative by qRT-PCR, was also detected by RT-LAMP.

### Analytical sensitivity of the RT-LAMP

**Fig 4:**
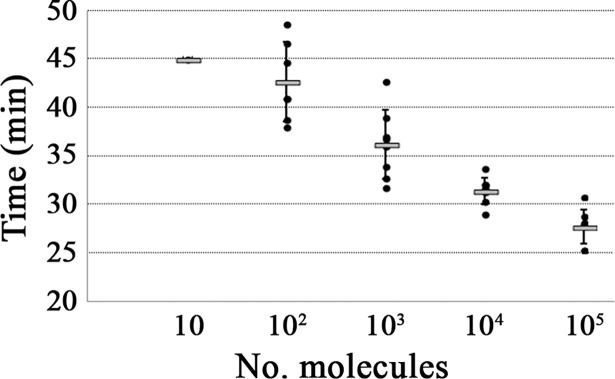
Times (min) of positive detection using serial 10-fold dilutions of CHIKV RNA standard. The mean values are represented with a grey bar and error bars indicate the SD. Black dots refer to positive signals of 8 independent runs.

The developed RT-LAMP detected up to 10 molecules per reaction, although this was only achieved in 1 of 8 repetitions at 45 min. The lowest number of RNA molecules detected in the 8 reactions, showing 100% reproducibility, was 10^3^ (36.2 ± 3.5 min), whilst 10^2^ molecules were detected in 7 of 8 repetitions (mean time, 42.7 ± 4.1 min) (Fig 4). Probit analysis of the results of 8 runs revealed that the limit of detection at 95% probability was 163 molecules.

### Validation of the RT-LAMP

S1 Table summarises the results obtained after the analyses of the RNA samples by qRT-PCR (C_T_ values) and RT-LAMP (T_T_ values, min). RNA samples used in this study were initially analysed by qRT-PCR in 2015 resulting in 33/35 positives (“initial C_T_ values”). On repetition of the qRT-PCR after storage at -80°C for 7 months only 19/35 samples remained positive (“current C_T_ values”).

When RT-LAMP was used 24/35 samples showed amplification in the 3 replicates tested, with T_T_ values below 30 min in 22 out of 35 samples. Four samples with negative current C_T_ values (IPD 277599, 277530, 274843 and 264781) were negative in RT-LAMP, while qRT-PCR negative sample IPD 264842 was positive by RT-LAMP (T_T_ = 29–42 min). Samples IPD 274461, 274688, 274464, 277604, positive in initial qRT-PCR but with no current C_T_ values, showed positive results by RT-LAMP in the 3 replicates analysed. In addition, 7 RNA samples negative in the 2016 by qRT-PCR amplified by RT-LAMP but not in all 3 replicates (IPD numbers 277586, 264998, 274443, 277593, 277551, 277545 and 264779).

The evaluation of the RT-LAMP assay demonstrated a 100% sensitivity (95% confidence interval -CI-, 88–100%) as all the RNA samples were positive by qRT-PCR and RT-LAMP and 80% specificity (95% CI, 28–99%), due to 1 sample negative by qRT-PCR but positive by RT-LAMP. The calculated predictive values were 97% PPV (95% CI, 83–99%) and 100% NPV (95% CI, 39–100%).

## Discussion

Rapid diagnostic methods are emerging as cost-effective, specific and sensitive techniques for laboratories with limited resources. The use of isothermal amplification methods avoids expensive equipment as results of the reactions can be visualised by different ways, ranging from naked eye (colorimetric detection) to quantitative results (spectrophotometer and fluorimeter) or dedicated devices, such as ESE-Quant TubeScanner, T8 (Axxin, Fairfield, Australia) and Genie II, within a short period of time. Current CHIKV outbreaks in different parts of the world [8, 37–39] highlight early detection (before the onset of clinical symptoms) is crucial to prevent virus spread, to control outbreaks and to initiate appropriate symptomatic therapy as specific treatment or vaccines are currently not available.

In addition, affordable and economic techniques are required for remote rural locations in which CHIKV is currently mainly diagnosed based on clinical signs and symptoms [40], which is not very reliable.

EQA panels have been developed in order to evaluate and verify the performance and reliability of current diagnostic assays in laboratories worldwide, by using different samples (both negative and positive samples in different concentrations) which provide information about the specificity and sensitivity of the assays [33, 41]. The EQA panel used in this study [33] comprises 2 of the 3 CHIKV genotypes, as well as different concentrations of 1 of the CHIKV strains, and the specificity is analysed against other arboviruses and negative samples used as negative controls. The analysis showed that the RT-LAMP developed is specific and sensitive, allowed the detection of all the CHIKV samples included in the EQA panel and no false positives were detected. In contrast, the qRT-PCR used to develop the molecular RNA standard did not detect one of the EQA panel samples (Table 1).

Use of RT-LAMP is spreading and similar specificity and sensitivity levels compared to real-time RT-PCR methods are being reported [22, 42, 43]. Related to CHIKV detection, 2 RT-LAMP methods for detection of CHIKV have been described in 2007 and 2012 [27, 29], although information about the number of sequences considered for the primer design is limited.

The accelerating determination of RNA virus genome sequences limits the traditional design approach in which conserved regions are identified as target regions for molecular assays in alignments of available sequences. Whereas in a recent design of a DENV RT-LAMP assay more than 2,000 whole genome sequences were used to design LAMP amplicons distributed across the DENV genome sequence (DENV LAMP paper accepted to be published at PLoS Neglected Tropical Diseases), we here chose an already known conserved target region (6K-E1) of the CHIKV genome, which was extracted from an initial alignment of all the 110 sequences of the target region deposited in GenBank. A PCA on Single Nucleotide Polymorphism and sequence variation of the 6K-E1 region was carried out using R/adegenet. This assessment allows to discriminate the sequences according to sequence diversity and similarity. Four distinct groups (Fig 2A) were identified and the LAMP primer-design was carried on each individual group and all potential combinations in order to minimise the number of primer sets designed.

Previously published primers [29] covered all the sequences used in this study and no mismatches were observed in the primers when the sequences were located in the viral genome. However, the other existing assay [27] covered all the sequences but only groups 1 and 3 without any mismatches. Two mismatches located in the F3 and F1c regions were observed when the primers were aligned with sequences belonging to groups 2 and 4 (data non-shown). In addition, these RT-LAMP assays were validated using serum samples, including healthy samples as negative controls. All the healthy samples showed no amplification. Thirty-eight out of 69 positive samples (acute-phase serum samples) and 3 out of 42 positives in unknown samples were detected [27, 29].

CHIKV RT-LAMP detection limits observed in previous studies were 20 and 27 RNA molecules detected per reaction in 30 and 77 min, respectively [27, 29], but these authors did not mention if the detection limits were calculated based on a probit analysis or a determined number of repetitions. Therefore, it is hard to compare the analytical sensitivity of our assay with those previously published. As a matter of fact, our RT-LAMP was capable to detect 10 RNA molecules if the run time was extended to 45 min. However, the probit analysis (calculated after 8 independent data sets) determined that the limit of detection was 163 molecules detected at 40.2 min. The specificity as shown by testing the EQA samples showed concordant results to qRT-PCR results, including sample 10 which was qRT-PCR negative, but RT-LAMP positive.

The methodology was validated using RNA samples collected during a CHIKV outbreak in Senegal (2015). The results showed a 100% concordance between the positive results obtained with the qRT-PCR used by IPD and the RT-LAMP protocol developed. In addition, the RT-LAMP seemed to be more sensitive as 11 samples out of 35 samples that initially were positive in 2015 (“initial C_T_ values”) but negative in 2016 (“current C_T_ values) by qRT-PCR were all detected with the RT-LAMP developed. This appears to indicate that LAMP is less affected by the storage conditions of RNA extracts than qRT-PCR and could explain why sample IPD 264842 negative by qRT-PCR was detected in 3 out of 3 LAMP replicates (T_T_ values ranging from 29 to 42 min).

Indeed LAMP shows a higher robustness in terms of pH change, temperature stability and the use of untreated fluids, such as stool, blood cultures, and plant extracts that commonly inhibit PCR reactions [44, 45]. A LAMP assay for *Salmonella enterica* serovar Typhi was developed and compared with an in-house qPCR, and demonstrated that LAMP reactions were specific and sensitive at pH 7.3–9.3, temperatures between 57–67°C, and even when using samples without an extensive DNA purification, that did not yield to amplification by qPCR [44]. The optimization of another LAMP assay allowed direct testing of crude homogenates in grapevine samples without the need for DNA extraction [45]. Our observation that stored RNA extracts can be more reliably detected by RT-LAMP than by qRT-PCR after months of storage could be good news for laboratories with unstable electricity supplies causing temperature fluctuation in their freezers which affects RNA stability.

The determination of clinical sensitivity, specificity, PPV and NPV allows interpretation of diagnostic results for clinical decisions [46, 47]. The RT-LAMP developed scored a sensitivity of 100% and specificity of 80% in reference to the qRT-PCR used by IPD, which means that all samples detected as positive by the LAMP assay are truly positive but the sample IPD 264842, negative by qRT-PCR and 3 out of 3 positives by RT-LAMP, may actually be positive, as this sample comes from a CHIKV outbreak and, as discussed before, our results demonstrated the higher sensitivity of our RT-LAMP assay, with 11 out of 35 samples initially positive in 2015 (“initial C_T_ values”), negative in 2016 (“current C_T_ values) by qRT-PCR, but positive by RT-LAMP. We tested for qPCR inhibitors in sample IPD 264842 by spiking CHIKV into this sample and confirmed that no inhibition was observed by qRT-PCR (data non-shown).

The scores obtained for PPV and NPV estimate the probability that the disease is present or absent depending if the result is positive or negative. Since the samples were collected in an outbreak, the results obtained with the RT-LAMP (PPV = 97% and NPV = 100%) highlight a good performance of the method in determining true positive cases while excluding negative cases. It has to be cautioned that only 35 samples were analysed and a greater number of positive and negative samples would allow to obtain more accurate results.

To conclude, a single-tube one-step real-time RT-LAMP assay was successfully designed using combined PCA and the LAVA software from 110 GenBank sequences for the conserved 6K-E1 target region. The assay was evaluated with an EQA panel from ENIVD and validated using viral RNA extracted from 35 serum samples collected during a recent CHIKV outbreak in Senegal. In comparison to qRT-PCR, the RT-LAMP appeared to show superior performance with material stored for months and can be therefore recommended for use in infrastructure poor settings.

## Supporting information

S1 File GenBank sequences used to generate LAMP primers. Each sequence is provided as a FASTA formatted sequences, the sequence header provide the NCBI GenBank accession number, Collection year, Collection country and the group number used for the primer design.
(DOCX)

S2 File STARD 2015 checklist. (DOCX)

S1 Table RNA samples collected from the CHIKV outbreak and analysed in this study. (DOCX)
