# Supplementary material for: Development of a single-tube one-step RT-LAMP assay to detect the Chikungunya virus genome
Source: PLoS Negl Trop Dis. 2018 May 29;12(5):e0006448. doi: 10.1371/journal.pntd.0006448 (PMC5973553; doi:10.1371/journal.pntd.0006448)
Supplement: S1 File — Each sequence is provided as a FASTA formatted sequences, the sequence header provide the NCBI GenBank accession number, Collection year, Collection country and the group number used for the primer design. (DOCX) [file pntd.0006448.s001.docx]

>KJ451624|2014|British Virgin Islands, Group 1

ATGGCTGCGTGAGACACACGTAGCCTACCAGTTTCTTACTGCTCTACTCTGCAAAGCAAGAGATTAATAACCCATCATGGATTCTGTGTACGTGGATATAGACGCTGACAGCGCCTTTTTGAAGGCCCTGCAACGTGCGTACCCCATGTTTGAGGTGGAACCTAGGCAGGTCACATCAAATGACCATGCTAATGCTAGAGCGTTCTCGCATCTAGCCATAAAACTAATAGAGCAGGAAATTGATCCCGACTCAACCATCCTGGATATAGGTAGTGCGCCAGCAAGGAGGATGATGTCGGACAGGAAGTACCACTGCGTTTGCCCGATGCGCAGCGCAGAAGATCCCGAGAGACTCGCTAATTATGCGAGAAAGCTCGCATCTGCCGCAGGAAAAGTCCTGGACAGAAACATTTCTGGAAAGATCGGGGACTTACAAGAGGTGATGGCCGTGCCAGACACGGAGACGCCAACATTTTGCTTACACACAGATGTCTCATGTAGACAGAGAGCAGACGTCGCGATATACCAAGACGTCTATGCTGTACATGCACCCACGTCGCTATATCACCAGGCGATTAAAGGAGTCCGAGTGGCGTACTGGGTAGGGTTCGACACAACCCCGTTCATGTACAACGCTATGGCGGGTGCCTACCCCTCATACTCGACAAATTGGGCGGATGAGCAGGTACTGAAGGCTAAGAACATAGGATTATGTTCAACAGACCTGACAGAAGGTAGACGAGGCAAATTGTCTATCATGAGAGGGAAAAAGCTAAAACCGTGCGACCGTGTGCTGTTCTCAGTAGGGTCAACGCTTTACCCGGAAAGCCGCATGCTACTTAAGAGCTGGCACCTACCATCGGTGTTCCATCTAAAGGGCAAGCTTAGCTTCACATGCCGCTGTGACACAGTGGTTTCGTGTGAGGGCTACGTCGTTAAGAGAATAACGATGAGCCCAGGCCTTTATGGAAAAACCACGGGGTATGCGGTAACCCACCACGCAGACGGATTCTTGATGTGCAAGACTACCGACACGGTAGACGGTGAAAGAGTGTCATTCTCGGTGTGCACGTACGTGCCGGCGACCATTTGTGATCAAATGACCGGCATCCTTGCTACAGAAGTCACGCCGGAGGATGCACAGAAGCTGTTGGTGGGGCTGAACCAGAGAATAGTGGTTAACGGCAGAACGCAACGGAACACGAACACCATGAAGAACTACCTGCTTCCCGTGGTCGCCCAGGCCTTCAGTAAGTGGGCGAAGGAGTGCCGGAAGGACATGGAAGATGAGAAGCTTTTGGGGGTCAGAGAAAGAACACTGACCTGCTGCTGTCTGTGGGCATTTAAGAAGCAGAAAACACACACGGTCTACAAGAGGCCTGATACCCAGTCAATCCAGAAGGTTCAGGCCGAATTCGACAGCTTTGTAGTACCAGGCCTGTGGTCGTCCGGGTTGTCAATCCCGTTGAGGACTAGAATCAAGTGGCTGTTACGCAAGGTGCCGAAAGCAGACCTGATCCCATACAGCGGAAATGCCCAAGAAGCCCAGGATGCTGAAAAAGAAGCAGAGGAAGAACGAGAAGCAGAACTGACTCATGAGGCTCTACCACCCCTACAGGCAGCACAGGAAGATGTCCAGGTCGAAATCGACGTGGAACAACTTGAGGATAGAGCTGGTGCTGGAATAATAGAGACTCCGAGAGGCGCTATTAAAGTTACTGCCCAACTAACGGACCACGTCGTGGGGGAGTACCTGGTACTTTCCCCGCAGACCGTATTACGCAGCCAGAAGCTCAGCCTGATCCACGCTTTAGCGGAGCAAGTGAAGACGTGTACGCATAGCGGACGAGCAGGGAGGTATGCGGTCGAAGCGTACGATGGCCGAGTCCTAGTGCCCTCAGGCTATGCAATTTCGCCCGAAGACTTCCAGAGTCTAAGCGAAAGCGCAACGATGGTGTACAACGAAAGAGAGTTCGTAAACAGAAAGTTACACCACATTGCGATGCACGGACCAGCTCTGAACACTGACGAAGAGTCGTATGAGCTTGTGAGGGCAGAGAGGACAGAACACGAGTACGTCTACGACGTGGACCAGAGAAGATGCTGCAAGAAGGAAGAAGCTGCAGGATTGGTACTGGTGGGCGACTTGACTAATCCGCCCTACCACGAATTCGCATACGAAGGGCTAAAAATTCGCCCCGCTTGTCCATATAAAATTGCAGTCATAGGAGTCTTCGGGGTACCAGGATCTGGTAAGTCAGCCATTATCAAGAACCTAGTTACCAGGCAAGACCTGGTGACTAGCGGAAAGAAAGAAAACTGCCAAGAAATCAGCACCGACGTGATGAGACAGAGAGGTCTAGAAATATCTGCACGTACGGTTGATTCGCTGCTCTTGAATGGATGCAATAGACCAGTCGACGTGTTGTACGTAGACGAGGCGTTTGCGTGCCACTCTGGAACGTTACTTGCCTTGATCGCCTTGGTGAGACCAAGACTGAAAGTTGTACTTTGTGGTGACCCGAAGCAGTGCGGCTTCTTCAATATGATGCAGATGAAAGTCAACTACAATCATAACATCTGCACTCAAGTGTACCACAAAAGTATTTCCAGGCGGTGTACACTGCCTGTGACTGCCATTGTGTCGTCGTTGCATTACGAAGGCAAAATGCGCACTACGAATGAGTACAACATGCCGATTGTAGTGGACACTACGGGCTCAACAAAACCTGACCCTGGAGACCTCGTGTTAACGTGCTTCAGAGGGTGGGTTAAACAACTGCAAATTGACTATCGTGGACACGAGGTCATGACAGCAGCCGCATCCCAAGGGTTAACCAGAAAAGGAGTTTACGCAGTTAGGCAAAAAGTTAACGAAAACCCACTCTATGCATCAACATCAGAGCACGTCAACGTACTCCTAACGCGTACGGAAGGTAAACTGGTATGGAAGACACTCTCTGGTGACCCGTGGATAAAGACGCTGCAGAACCCACCGAAAGGAAACTTCAAGGCAACTATTAAGGAGTGGGAGGTGGAGCACGCATCGATAATGGCGGGCATCTGCAGTCACCAAGTGACCTTTGACACGTTCCAAAACAAAGCCAACGTTTGCTGGGCTAAGAGCTTGGTCCCTATCCTCGAAACAGCGGGGATAAAACTAAATGATAGGCAGTGGTCCCAGATAATTCAAGCTTTCAAAGAAGACAAAGCATACTCACCCGAAGTAGCCCTGAATGAAATATGCACGCGCATGTATGGGGTGGATCTAGACAGCGGGCTATTCTCTAAACCGTTGGTATCTGTGTATTACGCGGATAACCACTGGGATAATAGGCCGGGAGGAAAGATGTTCGGATTCAACCCTGAGGCAGCGTCCATTCTAGAAAGAAAGTACCCGTTTACAAAAGGAAAGTGGAACATCAACAAGCAGATCTGCGTGACTACCAGGAGGATAGAAGACTTCAACCCTACCACCAACATTATACCGGCCAACAGGAGACTACCACACTCATTAGTGGCCGAACACCGCCCAGTAAAAGGGGAAAGAATGGAATGGCTGGTTAACAAGATAAACGGACATCATGTGCTCCTGGTTAGCGGCTATAACCTTGCACTGCCTACTAAGAGAGTCACCTGGGTAGCGCCACTAGGTGTCCGCGGAGCGGACTATACATACAACCTAGAGCTGGGTCTACCGGCAACACTTGGTAGGTATGACCTAGTGGTCATAAACATCCACACACCTTTTCGCATACACCATTACCAACAGTGCGTAGATCACGCAATGAAACTGCAAATGCTAGGAGGTGACTCACTGAGACTGCTCAAACCGGGTGGCTCTCTATTGATCAGAGCATACGGTTACGCAGATAGAACCAGTGAACGAGTAATCTGCGTACTGGGACGTAAGTTTAGATCGTCCAGAGCATTGAAACCACCATGTATCACCAGTAATACTGAGATGTTCTTCCTATTTAGCAGTTTTGACAATGGCAGAAGGAATTTTACAACGCATGTTATGAACAATCAACTGAACGCAGCCTTTGTAGGACAGGCCACCCGAGCAGGATGTGCACCATCGTACCGGGTAAAGCGCATGGACATCGCGAAGAACGATGAAGAGTGCGTGGTCAACGCCGCCAACCCTCGCGGGTTACCGGGTGACGGTGTTTGCAAGGCAGTATATAAAAAATGGCCGGAGTCCTTTAAAAATAGTGCAACACCAGTAGGAACCGCAAAAACAGTTATGTGCGGTACATATCCAGTAATCCATGCCGTAGGACCAAACTTCTCAAATTACACGGAGTCCGAAGGGGACCGGGAATTGGCGGCTGCCTATCGAGAAGTCGCAAAGGAAGTAACTAGACTGGGAGTAAATAGCGTAGCTATACCTCTCCTCTCCACAGGTGTATACTCAGGAGGGAAAGACAGGCTAACCCAGTCACTGAACCACCTCTTTACAGCCATGGACTCGACGGATGCAGACGTGGTCATCTACTGCCGAGACAAGGAATGGGAGAAGAAAATATCTGAGGCCATACAGATGCGGACCCAAGTGGAGCTGCTGGATGAGCACATCTCCATAGACTGCGATGTCATTCGCGTGCACCCTGACAGTAGTTTGGCAGGTAGAAAAGGATACAGCACCACGGAAGGCGCACTGTATTCATATCTAGAAGGGACACGTTTTCACCAGACGGCAGTGGATGTGGCAGAGATACATACTATGTGGCCAAAGCAAATAGAGGCCAATGAGCAAGTCTGCCTATATGCCCTGGGGGAAAGTATTGAGTCAATCAGGCAGAAATGCCCGGTGGATGATGCAGATGCATCATCTCCCCCGAAAACCGTCCCGTGCCTTTGCCGTTATGCCATGACTCCTGAACGCGTCACCCGACTTCGCATGAACCATGTCACAAATATAATTGTGTGTTCTTCATTTCCCCTTCCAAAGTACAAGATAGAAGGAGTGCAAAAAGTCAAATGCTCCAAGGTAATGTTATTTGATCACAATGTGCCATCGCGCGTAAGTCCAAGGGAATACAGATCTTCCCAGGAGTCTGTACGGGAAGTGAGTATGACAACGTCATTGACGCATAGTCAGTTTGATCTAAGCGCCGATGGCGAGACACTGCCCGTCCCGTCAGACCTGGATGCTGACGCCCCAGCCCTAGAACCGGCCCTAGACGACGGGGCGATACATACGACCGGAAACCTTGCGGCCGTGTCTGACTGGGTAATGAGCACCGTACCCGTCGCGCCGCCTAGAAGAAGGAGAGGGAGAAACCTGACCGTGATATGTGACGAGAGAGAAGGGAATATAACACCCATGGCTAGCGTCCGATTCTTTAGAGCAGAGCAGTGTCCGACCGTACAAGAAACAGCGGAGACGCGTGACACAGCTATTTCCTTTCGGGCACCGCCAAGTATCACCGTGGAACTGAGCCATCCACCGATCTCCTTCGGAGCACCAAGCGAGACGTTCCCCATCACATTTGGGGACTTCAACGATGGAGAAATCGAAAGCTTGTCTTCTGAGCTACTAACTTTCGGAGACTTCCTACCCGGTGAAGTGGATGATTTGACAGATAGCGACTGGTCCACGTGCTCAGACACGGACGACGAGTTATGACTAGACAGGGCAGGTGGGTATATATTCTCGTCGGACACTGGTCCAGGCCATTTACAACAGAAGTCGGTACGCCAGTCAGTGCTGCCGGTAAACACCCTGGAGGAAGTTCACGAGGAGAAGTGTTACCCACCTAAGCTGGATGAATTAAAGGAGCAACTACTACTTAAGAAACTCCAGGAGAGTGCGTCCACGGCCAATAGAAGCAGGTATCAATCACGCAAAGTGGAAAATATGAAAGCAACAATCATCCAGAGACTAAAGAGAGGCTGTAAACTGTATTTAATGGCAGAGACCCCGAAAGTCCCGACTTATCGGACCGTATACCCGGCGCCTGTGTACTCGCCTCCGATCAACGTCCGATTGTCCAATCCCGAGTCCGCAGTGGCAGCATGTAATGAGTTCTTAGCTAGAAACTACCCAACTGTTTCATCATACCAAATCACCGATGAGTATGATGCATATCTAGACATGGTGGACGGGTCGGAGAGTTGCTTGGACCGAGCGACATTCAATCCGTCAAAACTTAGGAGCTACCCGAAACAACATGCTTATCACGCGCCCTCTATCAGAAGCGCTGTACCTTCCCCATTCCAGAACACACTACAGAATGTACTGGCAGCAGCCACGAAAAGGAACTGCAACGTCACACAGATGAGGGAATTACCCACTTTGGACTCAGCAGTATTCAACGTGGAGTGTTTTAAAAAATTCGCATGCAACCGAGAATACTGGGAAGAATTTGCTGCCAGCCCTATCAGGATAACGACTGAGAATCTAACAACCTATGTCACTAAATTAAAGGGGCCAAAAGCAGCAGCGTTGCTTGCAAGAACCCATAATCTGCTGCCGCTGCAGGATGTACCAATGGATAGGTTCACAGTAGATATGAAAAGGGACGTGAAGGTAACTCCTGGCACAAAGCATACAGAGGAAAGGCCTAAGGTGCAGGTTATACAGGCGGCTGAACCCTTGGCAACAGCGTACCTATGTGGAATTCACAGAGAATTGGTTAGGAGATTGAACGCCGTCCTCCTACCCAACGTGCATACACTATTTGACATGTCTGCCGAGGACTTCGATGCCATTATAGCCGCACACTTTAAGCCAGGAGACGCCGTTTTAGAAACGGACATAGCCTCCTTTGATAAGAGCCAGGATGATTCACTTGCGCTTACCGCCTTAATGCTGTTAGAAGATTTGGGAGTGGATCACTCCTTGTTGGACCTGATAGAGGCTGCTTTTGGAGAGATTTCCAGCTGTCACCTGCCGACAGGTACGCGCTTCAAGTTCGGCGCTATGATGAAATCCGGTATGTTCCTAACTCTGTTCGTCAACACATTGTTAAATATCACCATCGCTAGCCGGGTGTTGGAAGATCGTCTGACAAAATCTGCATGCGCGGCCTTCATCGGCGACGACAACATAATACATGGTGTCGTCTCCGATGAATTGATGGCAGCCAGATGCGCTACTTGGATGAACATGGAAGTGAAGATCATAGATGCAGTTGTATCCCAGAAAGCTCCCTACTTTTGTGGAGGGTTTATACTGCATGATACTGTGACAGGAACAGCTTGCAGGGTGGCGGACCCGCTAAAAAGGTTATTTAAACTGGGCAAACCGTTAGCGGCAGGTGACGAACAAGACGAAGACAGAAGGCGGGCGCTGGCTGATGAAGTAATCAGATGGCAACGAACAGGGCTAATAGATGAGCTGGAGAAAGCGGTGTACTCTAGGTACGAAGTGCAGGGTATATCAGTTGCTGTAATGTCCATGGCCACCTTTGCAAGCTCCAGATCCAACTTCGAGAAGCTCAGAGGACCCGTCATAACCTTGTACGGCGGTCCTAAATAGGTACGCACTACAGCTACCTATTTTGCAAAAGCCGACAGCAGGTACCTAAATACCAATCAGCCATAATGGAGTTTATCCCAACCCAAACTTTCTACAATAGGAGGTACCAGCCTCGACCTTGGACTCCGCGCCCTACTATCCAAGTCATCAGACCCAGACCGCGTCCGCAAAGGAAGGCCGGGCAACTTGCCCAGCTGATCTCAGCAGTTAATAAACTGACAATGCGCGTGGTACCTCAACAGAAGCCGCGCAAGAATCGGAAGAATAAGAAGCAAAAGCAAAAGCAGCAGGCGCCACGAAACAATACGAATCAAAAGAAGCAGCCCCCCAAAAAGAAACCGGTTCAAAAGAAAAAGAAGCCGGGCCGCAGAGAGAGAATGTGCATGAAAATCGAAAATGATTGCATCTTCGAAGTCAAGCATGAAGGTAAGGTAACAGGTTACGCGTGCTTGGTAGGGGACAAAGTAATGAAGCCAGCACACGTAAAGGGGACCATCGATAACGCGGACCTGGCCAAATTGGCCTTCAAGCGGTCATCTAAGTACGACCTTGAATGCGCGCAGATACCCGTGCACATGAAGTCCGACGCTTCGAAGTTCACCCATGAGAAACCGGAGGGGTACTACAACTGGCACCACGGAGCAGTACAGTACTCAGGAGGCCGATTCACCATCCCTACAGGTGCGGGCAAACCAGGGGATAGTGGTAGACCGATCTTCGACAACAAGGGGCGCGTGGTGGCCATAGTTTTAGGAGGAGCTAATGAAGGAGCCCGTACAGCCCTCTCGGTGGTGACCTGGAACAAAGACATCGTCACGAAAATCACCCCTGAGGGGGCCGAAGAGTGGAGTCTGGCCATTCCAGTTATGTGCCTGCTGGCAAATACCACGTTCCCCTGCTCCCGGCCCCCTTGCACACCCTGCTGCTACGAAAAAGAGCCGGAGAAAACCTTGCGCATGCTTGAAGACAATGTCATGAGCCCCGGGTACTATCAGCTGCTACAAGCATCCTTAACATGTTCTCCCCGACGCCAGCGGCGCAGTATTAAGGACCACTTCAATGTCTATAAAGCCACAAGACCGTACCTAGCTCACTGTCCCGACTGTGGAGAAGGGCACTCGTGCCATAGTCCCGTAGCGCTAGAACGCATCAGAAACGAAGCGACAGACGGGACGTTGAAAATCCAGGTTTCCTTGCAAATCGGAATAAAGACGGATGATAGCCATGATTGGACCAAGCTGCGTTATATGGACAATCACATGCCAGCAGACGCAGAGCGGGCCGGGCTATTTGTAAGAACGTCAGCACCGTGCACGATTACTGGAACAATGGGACACTTCATTCTGGCCCGATGTCCGAAAGGAGAAACTCTGACGGCGGGGTTCACTGACGGTAGGAAGATCAGTCACTCATGTACGCACCCATTTCACCATGACCCTCCTGTGATAGGCCGGGAAAAATTCCATTCCCGACCGCAGCACGGTAGGGAACTACCTTGCAGCACGTACGCGCAGAGCACCGCTGCAACTGCCGAGGAGATAGAGGTACACATGCCCCCAGACACCCCAGATCGCACATTAATGTCACAACAGTCCGGCAATGTAAAGATCACAGTCAATAGTCAGACGGTGCGGTACAAGTGCAATTGTGGTGACTCAAGTGAAGGATTAACCACTACAGATAAAGTGATTAATAACTGCAAGGTCGATCAATGCCATGCCGCGGTCACCAATCACAAAAAATGGCAGTATAATTCCCCTCTGGTCCCGCGTAATGCTGAATTCGGGGACCGGAAAGGAAAAGTTCACATTCCATTTCCTCTGGCAAATGTGACATGCAGGGTGCCTAAAGCAAGAAACCCCACCGTGACGTACGGAAAAAACCAAGTCATCATGTTGCTGTATCCTGACCACCCAACGCTCCTGTCCTACAGGAATATGGGAGAAGAACCAAACTATCAAGAAGAGTGGGTGACGCATAAGAAGGAGATCAGGTTAACCGTGCCGACTGAGGGGCTCGAGGTCACGTGGGGTAACAATGAGCCGTACAAGTATTGGCCGCAGTTATCCACAAACGGTACAGCCCACGGCCACCCGCATGAGATAATTCTGTATTATTATGAGCTGTACCCAACTATGACTGCGGTAGTTTTGTCAGTGGCCTCGTTCATACTCCTGTCGATGGTGGGTGTGGCAGTGGGGATGTGCATGTGTGCACGACGCAGATGCATTACACCGTACGAACTGACACCAGGAGCTACCGTCCCTTTCCTGCTTAGCCTAATATGCTGCATTAGAACAGCTAAAGCGGCCACATACCAGGAGGCCGCGGTATACCTGTGGAACGAGCAGCAGCCTTTATTTTGGATGCAAGCCCTTATTCCGCTGGCAGCCCTGATTGTCCTATGTAACTGTCTGAGACTCTTACCATGCTGTTGTAAAATGTTGACTTTTTTAGCCGTACTGAGCGTCGGTGCCCACACTGTGAGCGCGTACGAACACGTAACAGTGATCCCGAACACGGTGGGAGTACCGTATAAGACTCTAGTCAACAGACCGGGCTACAGCCCCATGGTATTGGAGATGGAGCTTCTGTCTGTCACCTTGGAACCAACGCTATCGCTTGATTACATCACGTGCGAGTATAAAACCGTTATCCCGTCTCCGTACGTGAAATGCTGCGGTACAGCAGAGTGTAAGGACAAGAGCCTACCTGATTACAGCTGTAAGGTCTTCACCGGCGTCTACCCATTCATGTGGGGCGGCGCCTACTGCTTCTGCGACACCGAAAATACGCAATTGAGCGAAGCACATGTGGAGAAGTCCGAATCATGCAAAACAGAATTTGCATCAGCATACAGGGCTCATACCGCATCCGCATCAGCTAAGCTCCGCGTCCTTTACCAAGGAAATAATATCACTGTGGCTGCTTATGCAAACGGCGACCATGCCGTCACAGTTAAGGACGCTAAATTCATAGTGGGGCCAATGTCTTCAGCCTGGACACCTTTCGACAATAAAATCGTGGTGTACAAAGGCGACGTCTACAACATGGACTACCCGCCCTTCGGCGCAGGAAGACCAGGACAATTTGGCGACATCCAAAGTCGCACGCCTGAGAGCGAAGACGTCTATGCTAATACACAACTGGTACTGCAGAGACCGTCCGCGGGTACGGTGCACGTGCCGTACTCTCAGGCACCATCTGGCTTCAAGTATTGGCTAAAAGAACGAGGGGCGTCGCTGCAGCACACAGCACCATTTGGCTGTCAAATAGCAACAAACCCGGTAAGAGCGATGAACTGCGCCGTAGGGAACATGCCTATCTCCATCGACATACCGGACGCGGCCTTTACCAGGGTCGTCGACGCGCCATCTTTAACGGACATGTCGTGTGAGGTATCAGCCTGCACCCATTCCTCAGACTTTGGGGGCGTAGCCATCATTAAATATGCAGCCAGTAAGAAAGGCAAGTGTGCAGTGCACTCGATGACTAACGCCGTCACTATTCGGGAAGCTGAAATAGAAGTAGAAGGGAACTCTCAGTTGCAAATCTCTTTTTCGACGGCCCTAGCCAGCGCCGAATTTCGCGTACAAGTCTGTTCTACACAAGTACACTGTGCAGCCGAGTGCCATCCACCGAAAGACCATATAGTCAATTACCCGGCGTCACACACCACCCTCGGGGTCCAAGACATTTCCGCTACGGCGATGTCATGGGTGCAGAAGATCACGGGAGGTGTGGGACTGGTTGTCGCTGTTGCAGCACTGATCCTAATCGTGGTGCTATGCGTGTCGTTTAGCAGGCACTAACTTGACAACTAGGTATGAAGGCATACGCGTCCCTAAAGAGACACACCGCATATAGCTAGGAATCAACAGATAAGTATAGATCTAAGGGCTGAACAACCCCTGAATAACAAAATATAAAAATCAACAAAAATCATAAAATAGAAAACTAGAAATAGAAGTAGGTAAGAAGGTATATGTGTCCCCTAAGAGACACACCATATATAGCTAAGAATCAATAGATAAGCATAGATCAAAGGGCTGAACAACCCCTGAATAATAACAAAATATAAAAACCAATAAAAATCATAAAATAGAAAACCACAAATAGAAGTAGTTCAAAGGGCTATAAAACCCCTGAATAGTAACAAAATATAAAACTAATAAAAATCAAACGAATACCATAATTGGCAATCGGAAGAGATGTAGGTACTTAAGCTTCTTAAAAGCAGCCGAACTCGCTTTGAGATGTAGGCGTAGCACACCGAACTCTTCCATAATTCTCCGAACCCACAGGGACGTAGGAGATGTTCAAAGTGACTATAAAACCCTGAACAGTAATAAAATATAAAATTAATAATGAGTACCATAATTGGCAAATGGAAGAGACGTAGGTACTAAGCTTCTTAAAAGCAGCCGAACTCACTTTGAGATGTAGGCATAGCATACCGAACTCTTCCACAATTCTCCGTACCCATAGGGACGTAGGAGATGTTATTTTGTTTTTAATATTT

>KF318729|2012|China: Zhejiang Province, Group 1

ATGGCTGCGTGAGACACACGTAGCCTATCAGTTTCTTACTGCTCTACTCTGCAAAGCAAGAGATTAATAACCCATCATGGATTCTGTGTACGTGGATATAGACGCTGACAGCGCCTTTTTGAAGGCCCTGCAACGTGCGTACCCCATGTTTGAGGTGGAACCTAGGCAGGTCACATCAAATGACCATGCTAATGCTAGAGCGTTCTCGCATCTAGCCATAAAACTAATAGAGCAGGAAATTGATCCCGACTCAACCATCCTGGATATAGGTAGTGCGCCAGCAAGGAGGATGATGTCGGACAGGAAGTACCACTGCGTTTGCCCGGTGCGCAGCGCAGAAGATCCCGAGAGACTCGCTAATTATGCGAGAAAGCTCGCATCTGCCGCAGGAAAAGTCCTGGACAGAAACATTTCTGGAAAGATCGGGGACTTACAAGAGGTGATGGCCGTGCCAGACACGGAGACGCCAACATTTTGCTTACACACAGATGTCTCATGTAGACAGAGAGCAGACGTCGCGATATACCAAGACGTCTATGCTGTACATGCACCCACGTCGCTATATCACCAGGCGATTAAAGGAGTCCGAGTGGCGTACTGGGTAGGGTTCGACACAACCCCGTTCATGTACAACGCTATGGCGGGTGCCTACCCCTCATACTCGACAAATTGGGCGGATGAGCAGGTACTGAAGGCTAAGAACATAGGATTATGTTCAACAGACCTGACAGAAGGTAGACGAGGCAAATTGTCTATCATGAGAGGGAAAAAGCTAAAACCGTGCGACCGTGTGCTGTTCTCAGTAGGGTCAACGCTTTACCCGGAAAGCCGCATGCTACTTAAGAGCTGGCACCTACCATCGGTGTTCCATCTAAAGGGCAAGCTTAGCTTCACATGCCGCTGTGACACAGTGGTTTCGTGTGAGGGCTACGTCGTTAAGAGAATAACGATGAGCCCAGGCCTTTATGGAAAAACCACGGGGTATGCGGTAACCCACCACGCAGACGGATTCTTGATGTGCAAGACTACCGACACGGTAGACGGTGAAAGAGTGTCATTCTCGGTGTGCACGTACGTGCCGGCGACCATTTGTGATCAAATGACCGGCATCCTTGCTACAGAAGTCACGCCGGAGGATGCACAGAAGCTGTTGGTGGGGCTGAACCAGAGAATAGTGGTTAACGGCAGAACGCAACGGAACACGAACACCATGAAGAACTACCTGCTTCCCGTGGTCGCCCAGGCCTTCAGTAAGTGGGCGAAGGAGTGCCGGAAGGACATGGAAGATGAGAAGCTTCTGGGGGTCAGAGAAAGAACACTGACCTGCTGCTGTCTGTGGGCATTTAAGAAGCAGAAAACACACACGGTCTACAAGAGGCCTGATACCCAGTCAATCCAGAAGGTTCAGGCCGAATTCGACAGCTTTGTAGTACCAGGCCTGTGGTCGTCCGGGTTGTCAATCCCGTTGAGGACTAGAATCAAGTGGCTGTTACGCAAGGTGCCGAAAGCAGACCTGATCCCATACAGCGGAAATGCCCAAGAAGCCCAGGATGCTGAAAAAGAAGCAGAGGAAGAACGAGAAGCAGAACTGACTCATGAGGCTCTACCACCCCTACAGGCAGCACAGGAAGATGTCCAGGTCGAAATCGACGTGGAACAACTTGAGGATAGAGCTGGTGCTGGAATAATAGAGACTCCGAGAGGCGCTATTAAAGTTACTGCCCAACTAACGGACCACGTCGTGGGGGAGTACCTGGTACTTTCCCCGCAGACCGTATTACGCAGCCAGAAGCTCAGCCTGATCCACGCTTTAGCGGAGCAAGTGAAGACGTGTACGCATAGCGGACGAGCAGGGAGGTATGCGGTCGAAGCGTACGATGGCCGAGTCCTAGTGCCCTCAGGCTATGCAATTTCGCCCGAAGACTTCCAGAGTCTAAGCGAAAGCGCAACGATGGTGTACAACGAAAGAGAGTTCGTAAACAGAAAGTTACACCACATTGCGATGCACGGACCAGCTCTGAACACTGACGAAGAGTCGTATGAGCTTGTGAGGGCAGAGAGGACAGAACACGAGTACGTCTACGACGTGGACCAGAGAAGATGCTGCAAGAAGGAAGAAGCTGCAGGATTGGTACTGGTGGGCGACTTGACTAATCCGCCCTACCACGAATTCGCATACGAAGGGCTAAAAATTCGCCCCGCTTGTCCATATAAAATTGCAGTCATAGGAGTCTTCGGGGTACCAGGATCTGGTAAGTCAGCCATTATCAAGAACCTAGTTACCAGGCAAGACCTGGTGACTAGCGGAAAGAAAGAAAACTGCCAAGAAATCAGCACCGACGTGATGAGACAGAGAGGTCTAGAAATATCTGCACGTACGGTTGATTCGCTGCTCTTGAATGGATGCAATAGACCAGTCGACGTGTTGTACGTAGACGAGGCGTTTGCGTGCCACTCTGGAACGTTACTTGCCTTGATCGCCTTGGTGAGACCAAGACTGAAAGTTGTACTTTGTGGTGACCCGAAGCAGTGCGGCTTCTTCAATATGATGCAGATGAAAGTCAACTACAATCATAACATCTGCACTCAAGTGTACCACAAAAGTATCTCCAGGCGGTGCACACTGCCTGTGACTGCCATTGTGTCGTCGTTGCATTACGAAGGCAAAATGCGCACTACGAATGAGTACAACATGCCGATTGTAGTGGACACTACGGGCTCAACAAAACCTGACCCTGGAGACCTCGTGTTAACGTGCTTCAGAGGGTGGGTTAAACAACTGCAAATTGACTATCGTGGACACGAGGTCATGACAGCAGCCGCATCCCAAGGGTTAACCAGAAAAGGAGTTTACGCAGTTAGGCAAAAAGTTAACGAAAACCCACTCTATGCATCAACATCAGAGCACGTCAACGTACTCCTAACGCGTACGGAAGGTAAACTGGTATGGAAGACACTCTCTGGTGACCCGTGGATAAAGACGCTGCAGAACCCACCGAAAGGAAACTTCAAGGCAACTATTAAGGAGTGGGAGGTGGAGCACGCATCGATAATGGCGGGCATCTGCAGTCACCAAGTGACCTTTGACACGTTCCAAAACAAAGCCAACGTTTGCTGGGCTAAGAGCTTGGTCCCTATCCTCGAAACAGCGGGGATAAAACTAAATGATAGGCAGTGGTCCCAGATAATTCAAGCTTTCAAAGAAGACAAAGCATACTCACCCGAAGTAGCCCTGAATGAAATATGCACGCGCATGTATGGGGTGGATCTAGACAGCGGGCTATTCTCTAAACCGTTGGTATCTGTGTATTACGCGGATAACCACTGGGATAATAGGCCGGGAGGAAAGATGTTCGGATTCAACCCTGAGGCAGCGTCCATTCTAGAAAGAAAGTACCCGTTTACAAAAGGAAAGTGGAACATCAACAAGCAGATCTGCGTGACTACCAGGAGGATAGAAGACTTCAACCCTACCACCAACATTATACCGGCCAACAGGAGACTACCACACTCATTAGTGGCCGAACACCGCCCAGTAAAAGGGGAAAGAATGGAATGGCTGGTTAACAAGATAAACGGACATCATGTGCTCCTGGTTAGCGGCTATAACCTTGCACTGCCTACTAAGAGAGTCACCTGGGTAGCGCCACTAGGTGTCCGCGGAGCGGACTATACATACAACCTAGAGATGGGTCTACCGGCAACACTTGGTAGGTATGACCTAGTGGTCATAAACATCCACACACCTTTTCGCATACACCATTACCAACAGTGCGTAGATCACGCAATGAAACTGCAAATGCTAGGAGGTGACTCACTGAGACTGCTCAAACCGGGTGGCTCTCTATTGATCAGAGCATACGGTTACGCAGATAGAACCAGTGAACGAGTAATCTGCGTACTGGGACGTAAGTTTAGATCGTCCAGAGCATTGAAACCACCATGTATCACCAGTAATACTGAGATGTTCTTCCTATTTAGCAGTTTTGACAATGGCAGAAGGAATTTTACAACGCATGTTATGAACAATCAACTGAACGCAGCCTTTGTAGGACAGGCCACCCGAGCAGGATGTGCACCATCGTACCGGGTAAAACGCATGGACATCGCGAAGAACGATGAAGAGTGCGTGGTCAACGCCGCCAACCCTCGCGGGTTACCGGGTGACGGTGTTTGCAAGGCAGTATATAAAAAATGGCCGGAGTCCTTTAAAAATAGTGCAACACCAGTAGGAACCGCAAAAACAGTTATGTGCGGTACATATCCAGTAATCCATGCCGTAGGACCAAACTTCTCAAATTACACGGAGTCCGAAGGGGACCGGGAATTGGCGGCTGCCTATCGAGAAGTCGCAAAGGAAGTAACTAGACTGGGAGTAAATAGCGTAGCTATACCTCTCCTCTCCACAGGTGTATACTCAGGAGGGAAAGACAGGCTAACCCAGTCACTGAACCACCTCTTTACAGCCATGGACTCGACGGATGCAGACGTGGTCATCTACTGCCGAGACAAGGAATGGGAGAAGAAAATATCTGAGGCCATACAGATGCGGACCCAAGTGGAGCTGCTGGATGAGCACATCTCCATAGACTGCGATGTCATTCGCGTGCACCCTGACAGTAGTTTGGCAGGTAGAAAAGGATACAGCACCACGGAAGGCGCACTGTACTCATATCTAGAAGGGACACGTTTTCACCAGACGGCAGTGGATGTGGCAGAGATACATACTATGTGGCCAAAGCAAATAGAGGCCAATGAGCAAGTCTGCCTATATGCCCTGGGGGAAAGTATTGAGTCAATCAGGCAGAAATGCCCGGTGGATGATGCAGATGCATCATCTCCCCCGAAAACCGTCCCGTGCCTTTGCCGTTATGCCATGACTCCTGAACGCGTCACCCGACTTCGCATGAACCATGTCACAAATATAATTGTGTGTTCTTCATTTCCCCTTCCAAAGTACAAGATAGAAGGAGTGCAAAAAGTCAAATGCTCCAAGGTAATGTTATTTGATCACAATGTGCCATCGCGCGTAAGTCCAAGGGAATACAGATCTTCCCAGGAGTCTGTACGGGAAGTGAGTATGACAACGTCATTGACGCATAGTCAGTTTGATCTAAGCGCCGATGGCGAGACACTGCCCGTCCCGTCAGACCTGGATGCTGACGCCCCAGCCCTAGAACCGGCCCTAGACGACGGGGCGATACATACGACCGGAAACCTTGCGGCCGTGTCTGACTGGGTAATGAGCACCGTACCCGTCGCGCCGCCTAGAAGAAGGAGAGGGAGAAACCTGACCGTGATATGTGACGAGAGAGAAGGGAATATAACACCCATGGCTAGCGTCCGATTCTTTAGAGCAGAGCAGTGTCCGACCGTACAAGAAACAGCGGAGACGCGTGACACAGCTATTTCCTTTCGGGCACCGCCAAGTATCACCGTGGAACTGAGCCATCCACCGATCTCCTTCGGAGCACCAAGCGAGACGTTCCCCATCACATTTGGGGACTTCAACGATGGAGAAATCGAAAGCTTGTCTTCTGAGCTACTAACTTTCGGAGACTTCCTACCCGGTGAAGTGGATGATTTGACAGATAGCGACTGGTCCACGTGCTCAGACACGGACGACGAGTTATGACTAGACAGGGCAGGTGGGTATATATTCTCGTCGGACACTGGTCCAGGCCATTTACAACAGAAGTCGGTACGCCAGTCAGTGCTGCCGGTAAACACCCTGGAGGAAGTTCACGAGGAGAAGTGTTACCCACCTAAGCTGGATGAATTAAAGGAGCAACTACTACTTAAGAAACTCCAGGAGAGTGCGTCCACGGCCAATAGAAGCAGGTATCAATCACGCAAAGTGGAAAATATGAAAGCAACAATCATCCAGAGACTAAAGAGAGGCTGTAAACTGTATTTAATGGCAGAGACCCCGAAAGTCCCGACTTATCGGACCGTATACCCGGCGCCTGTGTACTCGCCTCCGATCAACGTCCGATTGTCCAATCCCGAGTCCGCAGTGGCAGCATGTAACGAGTTCTTAGCTAGAAACTACCCAACTGTTTCATCATACCAAATCACCGATGAGTATGATGCATATCTAGACATGGTGGACGGGTCGGAGAGTTGCTTGGACCGAGCGACATTCAATCCGTCAAAACTTAGGAGCTACCCGAAACAACATGCTTATCACGCGCCCTCTATCAGAAGCGCTGTACCTTCCCCATTCCAGAACACACTACAGAATGTACTGGCAGCAGCCACGAAAAGGAACTGCAACGTCACACAGATGAGGGAATTACCCACTTTGGACTCAGCAGTATTCAACGTGGAGTGTTTTAAAAAATTCGCATGCAACCGAGAATACTGGGAAGAATTTGCTGCCAGCCCTATCAGGATAACGACTGAGAATCTAACAACCTATGTCACTAAATTAAAGGGGCCAAAAGCAGCAGCGTTGCTTGCAAGAACCCATAATCTGCTGCCGCTGCAGGATGTACCAATGGATAGGTTCACAGTAGATATGAAAAGGGACGTGAAGGTAACTCCTGGCACAAAGCATACAGAGGAAAGGCCTAAGGTGCAGGTTATACAGGCGGCTGAACCCTTGGCAACAGCGTACCTATGTGGAATTCACAGAGAATTGGTTAGGAGATTGAACGCCGTCCTCCTACCCAACGTGCATACACTATTTGACATGTCTGCCGAGGACTTCGATGCCATTATAGCCGCACACTTTAAGCCAGGAGACGCCGTTTTAGAAACGGACATAGCCTCCTTTGATAAGAGCCAGGATGATTCACTTGCGCTTACCGCCTTAATGCTGTTAGAAGATTTGGGAGTGGATCACTCCTTGTTGGACCTGATAGAGGCTGCTTTTGGAGAGATTTCCAGCTGTCACCTGCCGACAGGTACGCGCTTCAAGTTCGGCGCTATGATGAAATCCGGTATGTTCCTAACTCTGTTCGTCAACACATTGTTAAATATCACCATCGCTAGCCGGGTGTTGGAAGATCGTCTGACAAAATCTGCATGCGCGGCCTTCATCGGCGACGACAACATAATACATGGTGTCGTCTCCGATGAATTGATGGCAGCCAGATGCGCTACTTGGATGAACATGGAAGTGAAGATCATAGATGCAGTTGTATCCCAGAAAGCTCCCTACTTTTGTGGAGGGTTTATACTGCATGATACTGTGACAGGAACAGCTTGCAGGGTGGCGGACCCGCTAAAAAGGTTATTTAAACTGGGCAAACCGTTAGCGGCAGGTGACGAACAAGACGAAGACAGAAGGCGGGCGCTGGCTGATGAAGTAATCAGATGGCAACGAACAGGGCTAATAGATGAGCTGGAGAAAGCGGTGTACTCTAGGTACGAAGTGCAGGGTATATCAGTTGCTGTAATGTCCATGGCCACCTTTGCAAGCTCCAGATCCAACTTCGAGAAGCTCAGAGGACCCGTCATAACCTTGTACGGCGGTCCTAAATAGGTACGCACTACAGCTACCTATTTTGCAAAAGCCGACAGCAGGTACCTAAATACCAATCAGCCATAATGGAGTTTATCCCAACCCAAACTTTCTACAATAGGAGGTACCAGCCTCGACCTTGGACTCCGCGCCCTACTATCCAAGTCATCAGACCCAGACCGCGTCCGCAAAGGAAGGCCGGGCAACTTGCCCAGCTGATCTCAGCAGTTAATAAACTGACAATGCGCGTGGTACCTCAACAGAAGCCGCGCAAGAATCGGAAGAATAAGAAGCAAAAGCAAAAGCAGCAGGCGCCACGAAACAATACGAATCAAAAGAAGCAGCCCCCTAAAAAGAAACCGGTTCAAAAGAAAAAGAAGCCGGGCCGCAGAGAGAGAATGTGCATGAAAATCGAAAATGATTGTATCTTCGAAGTCAAGCATGAAGGTAAGGTAACAGGTTACGCGTGCTTGGTAGGGGACAAAGTAATGAAGCCAGCACACGTAAAGGGGACCATCGATAACGCGGACCTGGCCAAATTGGCCTTCAAGCGGTCATCTAAGTACGACCTTGAATGCGCGCAGATACCCGTGCACATGAAGTCCGACGCTTCGAAGTTCACCCATGAGAAACCGGAGGGGTACTACAACTGGCACCACGGAGCAGTACAGTACTCAGGAGGCCGATTTACCATCCCTACAGGTGCGGGCAAACCAGGGGATAGTGGTAGACCGATCTTCGACAACAAGGGGCGCGTGGTGGCCATAGTTTTAGGAGGAGCTAATGAAGGAGCCCGTACAGCCCTCTCGGTGGTGACCTGGAACAAAGACATCGTCACGAAAATCACCCCTGAGGGGGCCGAAGAGTGGAGTCTGGCCATTCCAGTTATGTGCCTGCTGGCAAATACCACGTTCCCCTGCTCCCAGCCCCCTTGCACACCCTGCTGCTACGAAAAAGAGCCGGAGAAAACCTTGCGCATGCTTGAAGACAATGTCATGAGCCCCGGGTACTATCAGCTGCTACAAGCATCCTTAACATGTTCTCCCCGACGCCAGCGGCGCAGTATTAAGGACCACTTCAATGTCTATAAAGCCACAAGACCGTACCTAGCTCACTGTCCCGACTGTGGAGAAGGGCACTCGTGCCATAGTCCCGTAGCGCTAGAACGCATCAGAAACGAAGCGACAGACGGGACGTTGAAAATCCAGGTTTCCTTGCAAATCGGAATAAAGACGGATGATAGCCATGATTGGACCAAGCTGCGTTATATGGACAATCACATGCCAGCAGACGCAGAGCGGGCCGGGCTATTTGTAAGAACGTCAGCACCGTGCACGATTACTGGAACAATGGGACACTTCATTCTGGCCCGATGTCCGAAAGGAGAAACTCTGACGGTAGGGTTCACTGACGGTAGGAAGATCAGTCACTCATGTACGCACCCATTTCACCATGACCCTCCTGTGATAGGCCGGGAAAAATTCCATTCCCGACCGCAGCACGGTAGGGAACTACCTTGCAGCACGTACGCGCAGAGCACCGCTGCAACTGCCGAGGAGATAGAGGTACATATGCCCCCAGACACCCCAGATCGCACATTAATGTCACAACAGTCCGGCAATGTAAAGATCACAGTCAATAGTCAGACGGTGCGGTACAAGTGCAATTGTGGTGACTCAAGTGAAGGATTAACCACTACAGATAAAGTGATTAATAACTGCAAGGTTGATCAATGCCATGCCGCGGTCACCAATCACAAAAAATGGCAGTATAATTCCCCTCTGGTCCCGCGTAATGCTGAATTCGGGGACCGGAAAGGAAAAGTTCACATTCCATTTCCTCTGGCAAATGTGACATGCAGGGTGCCTAAAGCAAGAAACCCCACCGTGACGTACGGAAAAAACCAAGTCATCATGTTGCTGTATCCTGACCACCCAACGCTCCTGTCCTACAGGAATATGGGAGAAGAACCAAACTATCAAGAAGAGTGGGTGACGCATAAGAAGGAGATCAGGTTAACCGTGCCGACTGAAGGGCTCGAGGTCACGTGGGGTAACAATGAGCCGTACAAGTATTGGCCGCAGTTATCCACAAACGGTACAGCCCACGGCCACCCGCATGAGATAATTCTGTATTATTATGAGCTGTACCCAACTATGACTGTGGTAGTTTTGTCAGTGGCCTCGTTCATACTCCTGTCGATGGTGGGTGTGGCAGTGGGGATGTGCATGTGTGCACGACGCAGATGCATTACACCGTACGAACTGACACCAGGAGCTACCGTCCCTTTCCTGCTTAGCCTAATATGCTGCATTAGAACAGCTAAAGCGGCCACATACCAGGAGGCCGCGGTATACCTGTGGAACGAGCAGCAGCCTTTATTTTGGCTGCAAGCCCTTATTCCGCTGGCAGCCCTGATTGTCCTATGTAACTGTCTGAGACTCTTACCATGCTGTTGTAAAATGTTGACTTTTTTAGCCGTACTGAGCGTCGGTGCCCACACTGTGAGTGCGTACGAACACGTAACAGTGATCCCGAACACGGTGGGAGTACCGTATAAGACTCTAGTCAACAGACCGGGCTACAGCCCCATGGTATTGGAGATGGAGCTTCTGTCTGTCACCTTGGAACCAACGCTATCGCTTGATTACATCACGTGCGAGTATAAAACCGTTATCCCGTCTCCGTACGTGAAATGCTGCGGTACAGCAGAGTGTAAGGACAAGAGCCTACCTGATTACAGCTGTAAGGTCTTCACCGGCGTCTACCCATTCATGTGGGGCGGCGCCTACTGCTTCTGCGACACCGAAAATACGCAATTGAGCGAAGCACATGTGGAGAAGTCCGAATCATGCAAAACAGAATTTGCATCAGCATACAGGGCTCATACCGCATCCGCGTCAGCTAAGCTCCGCGTCCTTTACCAAGGAAATAATATCACTGTAGCTGCTTATGCAAACGGCGACCATGCCGTCACAGTTAAGGACGCTAAATTCATAGTGGGGCCAATGTCTTCAGCCTGGACACCTTTCGACAATAAAATCGTGGTGTACAAAGGCGACGTCTACAACATGGACTACCCGCCCTTCGGCGCAGGAAGACCAGGACAATTTGGCGACATCCAAAGTCGCACGCCTGAGAGCGAAGACGTCTATGCTAATACACAACTGGTACTGCAGAGACCGTCCGCGGGTACGGTGCACGTGCCGTACTCTCAGGCACCATCTGGCTTCAAGTATTGGCTAAAAGAACGAGGGGCGTCGCTGCAGCACACAGCACCATTTGGCTGTCAAATAGCAACAAACCCGGTAAGAGCGATGAACTGCGCCGTAGGGAACATGCCTATCTCCATCGACATACCGGACGCGGCCTTTACCAGGGTCGTCGACGCGCCATCTTTAACGGACATGTCGTGTGAGGTATCAGCCTGCACCCATTCCTCAGACTTTGGGGGCGTAGCCATCATTAAATATGCAGCCAGTAAGAAAGGCAAGTGTGCAGTGCACTCGATGACTAACGCCGTCACTATTCGGGAAGCTGAAATAGAAGTAGAAGGGAACTCTCAGTTGCAAATCTCTTTTTCGACGGCCCTAGCCAGCGCCGAATTTCGCGTACAAGTCTGTTCTACACAAGTACACTGTGCAGCCGAGTGCCATCCACCGAAAGACCATATAGTCAATTACCCGGCGTCACACACCACCCTCGGGGTCCAAGACATTTCCGCTACGGCGATGTCATGGGTGCAGAAGATCACGGGAGGTGTGGGACTGGTTGTCGCTGTTGCAGCACTGATCCTAATCGTGGTGCTATGCGTGTCGTTTAGCAGGCACTAACTTGACAACTAGGTATGAAGGCATACGCGTCCCTAAAGAGACACACCGCATATAGCTAGGAATCAATAGATAAGTATAGATCTAAGGGCTGAACAACCCCTGAATAGTAACAAAATATAAAAATCAACAAAAATCATAAAATAGAAAACTAGAAATAGAAGTAGGTAAGAAGGTATATGTGTCCCCTAAGAGACACACCATATATAGCTAAGAATCAATAGATAAGCATAGATCAAAGGGCTGAACAACCCCTGAATAATAACAAAATATAAAAACCAATAAAAATCATAAAATAGAAAACCACAAATAGAAGTAGTTCAAAGGGCTATAAAACCCCTGAATAGTAACAAAATATAAAACTAATAAAAATCAAACGAATACCATAATTGGCAATCGGAAGAGATGTAGGTACTTAAGCTTCTTAAAAGCAGCCGAACTCGCTTTGAGATGTAGGCGTAGCACACCGAACTCTTCCATAATTCTCCGAACCCACAGGGACGTAGGAGATGTTCAAAGTGACTATAAAACCCTGAACAGTAATAAAATATAAAATTAATAATGAGTACCATAATTGGCAAATGGAAGAGACGTAGGTACTAAGCTTCTTAAAAGCAGCCGAACTCACTTTGAGATGTAGGCATAGCATACCGAACTCTTCCACAATTCTCCGTACCCATAGGGACGTAGGAGATGTTATTTTGTTTTTAATATTTCTT

>FJ807897|2007|Indonesia, Group 1

ATGGCTGCGTGAGACACACGTAGCCTACCAGTTTCTTACTGCTCTACTCTGCAAAGCAAGAGATTAATAACCCATCATGGATTCTGTGTACGTGGATATAGACGCTGACAGCGCCTTTTTGAAGGCCCTGCAACGTGCGTACCCCATGTTTGAGGTGGAACCTAGGCAGGTTACATCAAATGACCATGCTAATGCTAGAGCGTTCTCGCATCTAGCCATAAAACTAATAGAGCAGGAAATTGATCCCGACTCAACCATCCTGGATATAGGTAGTGCGCCAGCAAGGAGGATGATGTCGGACAGGAAGTACCACTGCGTTTGCCCGATGCGCAGCGCAGAAGATCCCGAGAGACTCGCTAATTATGCGAGAAAGCTCGCATCTGCCGCAGGAAAAGTCCTGGACAGAAACATTTCTGGAAAGATCGGGGACTTACAAGCGGTGATGGCCGTGCCAGACACGGAGACGCCAACATTTTGCTTACACACAGATGTCTCATGTAGACAGAGAGCAGACGTCGCGATATACCAAGACGTCTATGCTGTACATGCACCCACGTCGCTATATCACCAGGCGATTAAAGGAGTCCGAGTGGCGTACTGGGTAGGGTTCGACACAACCCCGTTCATGTACAACGCTATGGCGGGTGCCTACCCCTCATACTCGACAAATTGGGCGGATGAGCAGGTACTGAAGGCTAAGAACATAGGATTATGTTCAACAGACCTGACAGAAGGTAGACGAGGCAAATTGTCTATCATGAGAGGGAAAAAGCTAAAACCGTGCGACCGTGTGCTGTTCTCAGTAGGGTCAACGCTTTACCCGGAAAGCCGCATGCTACTTAAGAGCTGGCACCTACCATCGGTGTTCCATCTAAAGGGCAAGCTTAGCTTCACATGCCGCTGTGACACAGTGGTTTCGTGTGAGGGCTACGTCGTTAAGAGAATAACGATGAGCCCAGGCCTTTATGGAAAAACCACGGGGTATGCGGTAACCCACCACGCAGACGGATTCTTGATGTGCAAGACTACCGACACGGTTGACGGCGAAAGAGTGTCATTCTCGGTGTGCACGTACGTGCCGGCGACCATTTGTGATCAAATGACCGGCATCCTTGCTACAGAAGTCACGCCGGAGGATGCACAGAAGCTGTTGGTGGGGCTGAACCAGAGAATAGTGGTTAACGGCAGAACGCAACGGAACACGAACACCATGAAGAACTATCTACTTCCCGTGGTCGCCCAGGCCTTCAGTAAGTGGGCGAAGGAGTGCCGGAAGGACATGGAAGATGAGAAGCTTCTGGGGGTCAGAGAAAGAACACTAACCTGCTGCTGTCTGTGGGCATTTAAGAAGCAGAAAACACACACGGTCTACAAGAGGCCTGATACCCAGTCAATCCAGAAGGTTCAGGCCGAATTTGACAGCTTTGTAGTACCAGGCCTGTGGTCGTCCGGGTTGTCAATCCCGTTGAGGACTAGAATCAAGTGGCTGTTACGCAAGGTGCCGAAAGCAGACCTGATCCCATACAGCGGAAATGCCCAAGAAGCCCAGGATGCTGAAAAAGAAGCAGAGGAAGAACGAGAAGCAGAACTGACTCATGAGGCTCTACCACCCCTACAGGCAGCACAGGAAGATGTCCAGGTCGAAATCGACGTGGAACAACTTGAGGATAGAGCTGGTGCTGGAATAATAGAGACTCCGAGAGGCGCTATTAAAGTTACTGCCCAACTAACAGACCACGTCGTGGGGGAGTACCTGGTACTTTCCCCGCAGACCGTATTACGCAGCCAGAAGCTCAGCCTGATCCACGCTTTAGCGGAGCAAGTGAAGACGTGCACGCATAGCGGACGAGCAGGGAGGTATGCGGTCGAAGCGTACGATGGCCGAGTCCTAGTGCCCTCAGGCTATGCAATTTCGCCTGAAGACTTCCAGAGTCTAAGCGAAAGCGCAACGATGGTGTACAACGAAAGAGAGTTCGTAAACAGGAAGTTACACCACATTGCGATGCACGGACCAGCCCTGAACACTGACGAAGAGTCGTATGAGCTTGTGAGGGCAGAGAGGACAGAACACGAGTACGTCTACGACGTGGACCAGAGAAGATGCTGTAAGAAGGAAGAAGCTGCAGGATTGGTACTGGTGGGCGACTTGACTAATCCGCCCTACCACGAATTCGCATACGAAGGGCTAAAAATTCGCCCCGCTTGTCCATACAAAATTGCAGTCATAGGAGTCTTCGGGGTACCAGGATCTGGTAAGTCAGCCATTATCAAGAACCTAGTTACCAGGCAAGACCTGGTGACTAGCGGAAAGAAAGAAAACTGCCAAGAAATCAGCACCGACGTGATGAGACAGAGAGGTCTAGAAATATCTGCACGTACGGTTGATTCGCTGCTCTTGAATGGATGCAATAGACCAGTCGACGTGTTGTACGTAGACGAGGCGTTTGCGTGCCACTCTGGAACGTTACTTGCCTTGATCGCCTTGGTGAGACCAAGACTGAAAGTTGTACTTTGTGGTGACCCGAAGCAGTGCGGCTTCTTCAATATGATGCAGATGAAAGTCAACTACAATCATAACATCTGCACTCAAGTGTACCACAAAAGTATCTCCAGGCGGTGTACACTGCCTGTGACTGCCATTGTGTCGTCGTTGCATTACGAAGGCAAAATGCGCACTACGAATGAGTACAACATGCCGATTGTAGTGGACACTACGGGCTCAACGAAACCTGACCCTGGAGACCTCGTGTTAACGTGCTTCAGAGGGTGGGTTAAACAACTGCAAATTGACTATCGTGGACACGAGGTCATGACAGCAGCCGCATCCCAAGGGTTAACCAGAAAAGGAGTTTACGCAGTTAGGCAAAAAGTTAACGAAAACCCACTCTATGCATCAACATCAGAGCACGTCAACGTACTCCTAACGCGTACGGAAGGTAAACTGGTATGGAAGACACTCTCTGGTGACCCGTGGATAAAGACGCTGCAGAACCCACCGAAAGGAAACTTCAAGGCAACTATTAAGGAGTGGGAGGTGGAGCACGCATCGATAATGGCGGGCATCTGCAGTCACCAAGTGACCTTTGACACGTTCCAAAACAAAGCCAACGTTTGCTGGGCTAAGAGCTTGGTCCCTATCCTCGAAACAGCAGGGATAAAACTAAATGATAGGCAGTGGTCCCAGATAATTCAAGCCTTCAAAGAAGACAAAGCATACTCACCCGAAGTAGCCCTGAATGAAATATGCACGCGCATGTATGGGGTGGATCTAGACAGCGGGCTATTCTCTAAACCGTTGGTATCTGTGTATTACGCGGATAACCACTGGGATAATAGGCCGGGAGGAAAGATGTTCGGATTCAACCCTGAGGCAGCGTCCATTCTAGAAAGAAAGTACCCGTTTACAAAAGGAAAGTGGAACATCAACAAGCAGATCTGCGTGACTACCAGGAGGATAGAAGACTTCAACCCTACCACCAACATTATACCGGCCAACAGGAGACTACCACACTCATTAGTGGCCGAACACCGCCCAGTAAAAGGGGAAAGAATGGAATGGCTGGTTAACAAGATAAACGGACATCATGTGCTCCTGGTTAGCGGCTATAACCTTGCACTGCCTACTAAGAGAGTCACCTGGGTAGCGCCACTAGGTGTCCGCGGAGCGGACTATACATACAACCTAGAGCTGGGTCTACCGGCAACACTTGGTAGGTATGACCTAGTGGTCATAAACATCCACACACCTTTTCGCATACACCATTACCAACAGTGCGTAGATCACGCAATGAAACTGCAAATGCTAGGGGGTGACTCACTGAGACTGCTCAAACCGGGTGGCTCTCTATTGATCAGAGCATACGGTTACGCAGATAGAACCAGTGAACGAGTAATCTGCGTACTGGGACGTAAGTTTAGATCGTCCAGAGCATTGAAACCACCATGTATCACCAGTAATACTGAGATGTTCTTCCTATTTAGCAGTTTTGACAATGGCAGAAGGAATTTTACAACGCATGTTATGAACAATCAACTGAACGCAGCCTTTGTAGGACAGGCCACCCGAGCAGGATGTGCACCATCGTACCGGGTAAAACGCATGGACATCGCGAATAACGATGAAGAGTGCGTGGTCAACGCCGCCAACCCTCGCGGGTTACCGGGTGACGGTGTTTGCAAGGCAGTATATAAAAAATGGCCGGAGTCCTTTAAAAATAGTGCAACACCAGTAGGAACCGCAAAAACAGTTATGTGCGGTACATATCCAGTAATCCACGCCGTAGGACCAAGCTTCTCAAATTACTCGGAGTCCGAAGGGGACCGGGAATTGGCGGCTGCCTATCGAGAAGTCGCAAAGGAAGTAACTAGACTGGGAGTAAATAGCGTAGCTATACCTCTCCTCTCCACAGGTGTATACTCAGGAGGGAAAGACAGGCTAACCCAGTCACTGAACCACCTCTTTACAGCCATGGACTCGACGGATGCAGACGTGGTCATCTACTGCCGAGACAAGGAATGGGAGAAGAAAATATCTGAGGCCATACAGATGCGGACCCAAGTGGAGCTGCTGGATGAGCACATCTCCATAGACTGCGATGTCATTCGCGTGCACCCTGACAGTAGTTTGGCAGGTAGAAAAGGATACAGCACCACGGAAGGCGCACTGTATTCATATCTAGAAGGGACACGTTTTCACCAGACGGCAGTGGATGTGGCAGAGATACATACTATGTGGCCAAAGCAAATAGAGGCCAATGAGCAAGTCTGCCTATATGCCCTGGGGGAAAGTATTGAATCAATCAGGCAGAAATGCCCGGTGGATGATGCAGATGCATCATCTCCCCCGAAAACCGTCCCGTGCCTTTGCCGTTATGCCATGACTCCTGAACGCGTCACCCGACTTCGCATGAACCATGTCACAAATATAATTGTGTGTTCTTCATTTCCCCTTCCAAAGTACAAGATAGAAGGAGTGCAAAAAGTCAAATGCTCCAAGGTAATGTTATTTGATCACAATGTGCCATCGCGCGTAAGTCCAAGGGAATACAGATCTTCCCAGGAGTCTGTACGGGAAGTGAGTATGACAACGTCATTGACGCATAGCCAGTTTGATCTAAGCGCCGATGGCGAGACACTGCCCGTCCCGTCAGACCTGGATGCTGACGCCCCAGCCCTAGAACCGGCCCTAGACGACGGGGCGATACATACGACCGGAAACCTTGCGGCCGTGTCTGACTGGGTAATGAGCACCGTACCTGTCGCGCCGCCTAGAAGAAGGAGAGGGAGAAACCTGACCGTGATATGTGACGAGAGAGAAGGGAATATAACACCCATGGCTAGCGTCCGATTCTTTAGAGCAGAGCAGTGTCCGGCCGTACAAGAAACAGCGGAGACGCGTGACACAGCTATTTCCTTTCGGGCACCGCCAAGTATCACCGTGGAACTGAGCCATCCACCGATCTCCTTCGGAGCACCAAGCGAGACGTTCCCCATCACATTTGGGGACTTCGACGATGGAGAAATCGAAAGCTTGTCTTCTGAGCTACTAACTTTCGGAGACTTCCTACCCGGTGAAGTGGATGATTTGACAGATAGCGACTGGTCCACGTGCTCAGACACGGACGACGAGTTATGACTAGACAGGGCAGGTGGGTATATATTCTCGTCGGACACTGGTCCAGGCCATTTACAACAGAAGTCGGTACGCCAGTCAGTGCTGCCGGTAAACACCCTGGAGGAAGTTCACGAGGAGAAGTGTTACCCACCTAAGCTGGATGAATTAAAGGAGCAACTACTACTTAAGAAACTCCAGGAGAGTGCGTCCACGGCCAATAGAAGCAGGTATCAATCACGCAAAGTGGAAAATATGAAAGCAACAATCATCCAGAGACTAAAGAGAGGCTGTAAACTGTATTTAATGGCAGAGACCCCGAAAGTCCCGACTTATCGGACCGTATACCCGGCGCCTGTGTACTCGCCTCCGATCAACATCCGATTGTCCAATCCCGAGTCCGCAGTGGCAGCATGTAATGAGTTCTTAGCTAGAAACTACCCAACTGTTTCATCATACCAAATCACCGATGAGTATGATGCATATCTAGACATGGTGGACGGGTCGGAGAGTTGCTTGGACCGAGCGACATTCAATCCGTCAAAACTTAGGAGCTACCCGAAACAACATGCTTATCACGCGCCTTCTATCAGAAGCGCTGTACCTTCCCCATTCCAGAACACACTACAGAATGTACTGGCAGCAGCCACGAAAAGGAACTGCAACGTCACACAGATGAGGGAATTACCCACTTTGGACTCAGCAGTATTCAACGTGGAGTGTTTTAAAAAATTCGCATGCAACCGAGAATACTGGGAAGAATTTGCTGCCAGCCCTATCAGGATAACGACTGAGAATCTAACAACCTATGTCACTAAACTAAAGGGGCCAAAAGCAGCAGCGTTGTTTGCAAGAACCCATAATCTGCTGCCGCTGCAGGATGTACCAATGGATAGGTTCACAGTAGATATGAAAAGGGACGTGAAGGTAACTCCTGGTACAAAGCATACAGAGGAAAGGCCTAAGGTGCAGGTTATACAGGCGGCTGAACCCTTGGCAACAGCGTACCTATGTGGAATTCACAGAGAATTGGTTAGGAGATTGAACGCCGTCCTCCTACCCAATGTGCATACACTATTTGACATGTCTGCCGAGGACTTCGATGCCATTATAGCCGCACACTTTAAGCCAGGAGACGCCGTTTTAGAAACGGACATAGCCTCCTTTGATAAGAGCCAGGATGATTCACTTGCGCTTACCGCCTTAATGCTGTTAGAAGATTTGGGAGTGGATCACTCCTTGTTGGACCTGATAGAGGCTGCTTTTGGAGAGATTTCCAGCTGTCACCTGCCGACAGGTACGCGCTTCAAGTTCGGCGCTATGATGAAATCCGGTATGTTCCTAACTCTGTTCGTCAACACATTGTTAAATATCACCATCGCTAGCCGGGTGTTGGAAGATCGTCTGACAAAATCTGCATGCGCGGCCTTCATCGGCGACGACAACATAATACATGGTGTCGTCTCCGATGAATTGATGGCAGCCAGATGCGCTACTTGGATGAACATGGAAGTGAAGATCATAGATGCAGTTGTATCCCAGAAAGCTCCTTACTTTTGTGGAGGGTTTATACTGCATGATACTGTGACAGGAACAGCTTGCAGGGTGGCGGACCCGCTAAAAAGGTTATTTAAACTGGGCAAACCGTTAGCGGCAGGTGACGAACAAGACGAAGACAGAAGACGGGCGCTGGCTGATGAAGTAATCAGATGGCAACGAACAGGGCTAATAGATGAGCTGGAGAAAGCGGTGTACTCTAGGTATGAAGTGCAGGGTATATCAGTTGCTGTAATGTCCATGGCCACCTTTGCAAGCTCCAGATCCAACTTCGAGAAGCTCAGAGGACCCGTCATAACTTTGTACGGCGGTCCTAAATAGGTACGCACTACAGCTACCTATTTTGCAAAAGCCGACGGCAGGTACCTAAATACCAATCAGCCATAATGGAGTTTATCCCAACCCAAACTTTCTACAATAGGAGGTACCAGCCTCGACCTTGGACTCCGCGCCCTACTATCCAAGTCATCAGACCCAGACCGCGTCCGCAAAGGAAGGCCGGGCAACTTGCCCAGCTGATCTCAGCAGTTAATAAACTGACAATGCGCGTGGTACCTCAACAGAAGCCGCGCAAGAATCGGAAGAATAAGAAGCAAAAGCAAAAGCAGCAGGCGCCACGAAACAATACGAATCAAAAGAAGCAGCCCCCTAAAAAGAAACCGGTTCAAAAGAAAAAGAAGCCGGGCCGCAGAGAGAGAATGTGCATGAAAATCGAAAATGATTGCATCTTCGAAGTCAAGCATGAAGGTAAGGTAACAGGTTACGCGTGCTTGGTAGGGGACAAAGTAATGAAGCCAGCACACGTAAAGGGGACCATCGATAACGCGGACCTGGCCAAATTGGCCTTCAAGCGGTCATCTAAGTACGACCTTGAATGCGCGCAGATACCCGTGCACATGAAGTCCGACGCTTCGAAGTTCACCCATGAGAAACCGGAGGGGTACTACAACTGGCACCACGGAGCAGTACAGTACTCAGGAGGCCGATTCACCATCCCTACAGGTGCGGGCAAACCAGGGGATAGTGGTAGACCGATCTTCGACAACAAGGGGCGCGTGGTGGCCATAGTTTTAGGAGGAGCTAATGAAGGAGCCCGTACAGCCCTCTCGGTGGTGACCTGGAACAAAGACATCGTCACGAAAATCACCCCTGAGGGGGCCGAAGAGTGGAGTCTGGCCATTCCAGTTATGTGCCTGCTGGCAAATACCACGTTCCCCTGCTCCCAGCCCCCTTGCACACCCTGCTGCTACGAAAAAGAGCCGGAGAAAACCTTGCGCATGCTTGAAGACAATGTCATGAGCCCCGGGTACTATCAGCTGCTACAAGCATCCTTAACATGTTCTCCCCGACGCCAGCGGCGCAGTATTAAGGACCACTTCAATGTCTATAAAGCCACAAGACCGTACCTAGCTCACTGTCCCGACTGTGGAGAAGGGCACTCGTGCCATAGTCCCGTAGCGCTAGAACGCATCAGAAACGAAGCGACAGACGGGACGTTGAAAATCCAGGTTTCCTTGCAAATCGGAATAAAGACGGATGATAGCCATGATTGGACCAAGCTGCGTTATATGGACAATCACATGCCAGCAGACGCAGAGCGGGCCGGGCTATTTGTAAGAACGTCAGCACCGTGCACGATTACTGGAACAATGGGACACTTCATTCTGGCCCGATGTCCGAAAGGAGAAACTCTGACGGTGGGGTTCACTGACGGTAGGAAGATCAGTCACTCATGTACGCACCCATTTCACCATGACCCTCCTGTGATAGGCCGGGAAAAATTCCATTCCCGACCGCAGCACGGTAGGGAACTACCTTGCAGCACGTACGCGCAGAGCACCGCTGCAACTGCCGAGGAGATAGAGGTACATATGCCCCCAGACACCCCAGATCGCACATTAATGTCACAACAGTCCGGCAATGTAAAGATCACAGTCAATAGTCAGACGGTGCGGTACAAGTGCAATTGTGGTGACTCAAGTGAAGGATTAACCACTACAGATAAAGTGATTAATAACTGCAAGGTTGATCAATGCCATGCCGCGGTCACCAATCACAAAAAATGGCAGTATAATTCCCCTCTGGTCCCGCGCAATGCTGAATTCGGGGACCGGAAAGGAAAAGTTCACATTCCATTTCCTCTGGCAAATGTGACATGCAGGGTGCCTAAAGCAAGAAACCCCACCGTGACGTACGGAAAAAACCAAGTCATCATGTTGCTGTATCCGGACCACCCAACGCTCCTGTCCTACAGGAATATGGGAGAAGAACCAAACTATCAAGAAGAGTGGGTGACGCATAAGAAGGAGATCAGGTTAACCGTGCCGACTGAAGGGCTCGAGGTCACGTGGGGTAACAATGAGCCGTACAAGTATTGGCCGCAGTTATCCACAAACGGTACAGCCCACGGCCACCCGCATGAGATAATTCTGTATTATTATGAGCTGTACCCAACTATGACTGTGGTAGTTTTGTCAGTGGCCTCGTTCATACTCCTGTCGATGGTGGGTGTAGCAGTGGGGATGTGCATGTGTGCACGACGCAGATGCATTACACCGTACGAACTGACACCAGGAGCTACCGTCCCTTTCCTGCTTAGCCTAATATGCTGCATTAGAACAGCTAAAGCGGCCACATACCAGGAGGCCGCGGTATACCTGTGGAACGAGCAGCAGCCTTTATTTTGGCTGCAAGCCCTTATTCCGCTGGCAGCCCTGATTGTCCTATGTAACTGTCTGAGACTCTTACCATGCTGTTGTAAAATGCTGACTTTTTTAGCCGTACTGAGCGTCGGTGCCCACACTGTGAGCGCGTACGAACACGTAACAGTGATCCCGAACACGGTGGGAGTACCGTATAAGACTCTAGTCAACAGACCGGGCTACAGCCCCATGGCACTGGAGATGGAGCTTCTGTCTGTCACCTTGGAACCAACGCTATCGCTTGATTACATCACGTGCGAGTATAAAACCGTTATCCCGTCTCCGTACGTGAAATGCTGCGGTACAGCAGAGTGTAAGGACAAGAGCCTACCTGATTACAGCTGTAAGGTCTTCACCGGCGTCTACCCATTCATGTGGGGCGGCGCCTACTGCTTCTGCGACACCGAAAATACGCAATTGAGCGAAGCACATGTGGAGAAGTCCGAATCATGCAAAACAGAATTTGCATCAGCATACAGGGCTCATACCGCATCCGCATCAGCTAAGCTCCGCGTCCTTTACCAAGGAAGTAATATCACTGTTGCTGCTTATGCAAACGGCGACCATGCCGTCACAGTTAAGGACGCTAAATTCATAGTGGGGCCAATGTCTTCAGCCTGGACACCTTTCGACAATAAAATCGTGGTGTACAAAGGCGACGTCTACAACATGGACTACCCGCCCTTCGGCGCAGGAAGACCAGGACAATTTGGCGACATCCAAAGTCGCACGCCTGAGAGCGAAGACGTCTATGCTAATACACAACTGGTACTGCAGAGACCGTCCGCGGGTACGGTGCACGTGCCGTACTCTCAGGCACCATCTGGCTTCAAGTATTGGCTAAAAGAACGAGGGGCGTCGCTGCAGCACACAGCACCATTTGGCTGTCAAATAGCAACAAACCCGGTAAGAGCGATGAACTGCGCCGTAGGGAACATGCCTATCTCCATCGACATACCGGACGCGGCCTTTACCAGGGTCGTCGACGCGCCATCTTTAACGGACATGTCGTGTGAGGTATCAGCCTGCACCCATTCCTCAGACTTTGGGGGCGTTGCCATCATTAAATATGCAGCCAGTAAGAAAGGCAAGTGTGCAGTGCACTCGATGACTAACGCCGTCACTATTCGGGAAGCTGAAATAGAAGTAGAAGGGAACTCTCAGTTGCAAATCTCTTTTTCGACGGCCCTAGCCAGCGCCGAATTCCGCGTACAAGTCTGTTCTACACAAGTACACTGTGCAGCCGAGTGCCATCCACCGAAAGACCATATAGTCAATTACCCGGCGTCACACACCACCCCCGGGGTCCAAGACATTTCCGCTACGGCGATGTCATGGGTGCAGAAGATCACGGGAGGTGTGGGACTGGTTGTCGCTGTTGCAGCACTGATCCTAATCGTGGTGCTATGCGTGTCGTTTAGCAGGCACTAACTTGACAACTAGGTATGAAGGCATACGCGTCCCTAAAGAGACACACCGCATATAGTTATGAATCAATAGATAAGTATAGATCAAAGGGCTGAACAACCCCTGAATAGTAACAAAATATAAAAATCAACAAAAATCATAAAATAGAAAACTAGAAATAGAAGTAGGTAAGAAGGTATATGTGTCCCCTAAGAGACACACCATATATAGCTAAGAATCAATAGATAAGCATAGATCAAAGGGCTGAATAACCCCTGAATAATAACAAAATATAAAAACCAATAAAAATCATAAAATAGAAAACCATAAATAGAAGTAGTTCAAAGGGCTATAAAACCCCTGAATAGTAACAAAATATAAAACTAATAAAAATCAAACGAATACCATAATTGGCAATCGGAAGAGATGTAGGTACTTAAGCTTCTTAAAAGCAGCCGAACTCGCTTTGAGATGTAGGCGTAGCACACCGAACTCTTCCATAATTCTCCGAACCCACAGGGACGTAGGAGATGTTCAAAGATTATAAAACCCTGAACAGTAATAAAATATAAAATTAATAATGAGTACCATAATTGGCAAATGGAAGAGATGTAGGTACTAAGCTTCTTAAAAGCAGCCGAACTCACTTTGAGATGTAGGCATAGCATACCGAACTCTTCCACAATTCTCCGTACCCATAGGGACGTAGGAGATGTTATTTTGTTTTTAATATTTC

>KM673291|2013|Indonesia: Bali, Group 1

ATGGCTGCGTGAGACACACGTAGCCTACCAGTTTCTTACTGCTCTACTCTGCAAAGCAAGAGATTAATAACCCATCATGGATTCTGTGTACGTGGATATAGACGCTGACAGCGCCTTTTTGAAGGCCCTGCAACGTGCGTACCCCATGTTTGAGGTGGAACCTAGGCAGGTCACATCAAATGACCATGCTAATGCTAGAGCGTTCTCGCATCTAGCCATAAAACTAATAGAGCAGGAAATTGATCCCGACTCAACCATCCTGGATATAGGTAGTGCGCCAGCAAGGAGGATGATGTCGGACAGGAAGTACCACTGCGTTTGCCCGATGCGCAGCGCAGAAGATCCCGAGAGACTCGCTAATTATGCGAGAAAGCTCGCATCTGCCGCAGGAAAAGTCCTGGACAGAAACATTTCTGGAAAGATCGGGGACTTACAAGCGGTGATGGCCGTGCCAGACACGGAGACGCCAACATTTTGCTTACACACAGATGTCTCATGTAGACAGAGAGCAGACGTCGCGATATACCAAGACGTCTATGCTGTACATGCACCCACGTCGCTATATCACCAGGCGATTAAAGGAGTCCGAGTGGCGTACTGGGTAGGGTTTGACACAACCCCGTTCATGTACAACGCTATGGCGGGTGCCTACCCCTCATACTCGACAAATTGGGCGGATGAGCAGGTACTGAAGGCTAAGAACATAGGATTATGTTCAACAGACCTGACGGAAGGTAGACGAGGTAAATTGTCTATCATGAGAGGGAAAAAGCTGAAACCGTGCGACCGTGTGCTGTTCTCAGTAGGGTCAACGCTTTACCCGGAAAGCCGCATGCTACTTAAGAGCTGGCACCTACCATCGGTGTTCCATCTAAAGGGCAAGCTTAGCTTCACATGCCGCTGTGACACAGTGGTTTCGTGTGAGGGCTACGTCGTCAAGAGAATAACGATGAGCCCAGGCCTTTATGGAAAAACCACGGGGTATGCGGTAACCCACCACGCAGACGGATTCTTGATGTGCAAGACTACCGACACGGTTGACGGCGAAAGAGTGTCATTCTCGGTGTGCACGTACGTGCCGGCGACCATTTGTGATCAAATGACCGGCATCCTTGCTACAGAAGTCACGCCGGAGGACGCACAGAAGCTGTTGGTGGGGCTGAACCAGAGAATAGTGGTTAACGGCAGAACGCAACGGAACACGAACACCATGAAGAACTATCTACTTCCCGTGGTCGCCCAGGCCTTCAGTAAGTGGGCGAAGGAGTGCCGGAAGGACATGGAAGATGAGAAGCTTCTGGGGGTCAGAGAAAGAACACTAACCTGCTGCTGTCTGTGGGCATTTAAGAAGCAGAAAACACACACGGTCTACAAGAGGCCTGATACCCAGTCAATCCAGAAGGTTCAGGCCGAATTTGACAGCTTTGTAGTACCAGGCCTGTGGTCGTCCGGGTTGTCAATCCCGTTGAGGACTAGAATCAAGTGGCTGTTACGCAAGGTGCCGAAAGCAGACCTGATCCCATACAGCGGAAATGCCCAAGAAGCCCAGGATGCAGAAAAAGAAGCAGAGGAAGAACGAGAAGCAGAACTGACTCATGAGGCTCTACCACCCCTACAGGCAGCACAGGAAGATGTCCAGGTCGAAATCGACGTGGAACAACTTGAGGATAGAGCTGGTGCTGGAATAATAGAGACTCCGAGAGGCGCTATTAAAGTTACTGCCCAACTAACCGACCACGTCGTGGGGGAGTACCTGGTACTTTCCCCGCAGACCGTATTACGCAGCCAGAAGCTCAGCCTGATCCACGCTTTAGCGGAGCAAGTGAAGACGTGTACGCATAGCGGACGAGCAGGGAGGTATGCGGTCGAAGCGTACGATGGCCGAGTCCTAGTGCCCTCAGGCTATGCAATTTCGCCTGAGGACTTCCAGAGTCTAAGCGAAAGCGCAACGATGGTGTACAACGAAAGAGAGTTCGTAAACAGAAAGTTACACCACATTGCGATGCACGGACCAGCCCTGAACACTGACGAAGAGTCGTATGAGCTTGTGAGGGCAGAGAGAACAGAACACGAGTACGTCTACGACGTGGACCAGAGAAGATGCTGTAAGAAGGAAGAAGCTGCAGGATTGGTACTGGTGGGCGACTTGACCAATCCGCCCTACCACGAATTTGCATACGAAGGGCTAAAAATTCGCCCCGCTTGTCCATACAAAATTGCAGTCATAGGAGTCTTCGGGGTACCAGGATCTGGTAAGTCAGCCATTATCAAGAACCTAGTTACCAGGCAAGACCTGGTGACTAGCGGAAAGAAAGAAAACTGCCAAGAAATCAGCACCGACGTGATGAGACAGAGAGGTCTAGAAATATCTGCACGTACGGTTGATTCACTGCTCTTGAATGGATGCAATAGACCAGTCGACGTGTTATACGTAGACGAGGCGTTTGCGTGCCACTCTGGAACGTTACTTGCCTTGATCGCCTTGGTGAGACCAAGACTGAAAGTTGTACTTTGTGGTGACCCGAAGCAGTGCGGCTTCTTCAATATGATGCAGATGAAAGTCAACTACAATCATAACATCTGCACTCAAGTGTACAACAAAAGTATCTCCAGGCGGTGTACACTGCCTGTGACTGCCATTGTGTCGTCGTTGCATTACGAAGGCAAAATGCGCACTACGAATGAGTACAACATGCCGATTGTAGTGGACACTACGGGCTCAACGAAACCTGACCCTGGAGACCTCGTGTTAACGTGCTTCAGAGGGTGGGTTAAACAGCTGCAAATTGACTATCGTGGACACGAGGTCATGACAGCAGCCGCATCCCAAGGGCTAACCAGAAAAGGAGTTTACGCAGTCAGGCAAAAAGTTAACGAAAACCCACTCTATGCATCAACATCAGAGCACGTCAACGTACTCTTAACGCGTACGGAAGGTAAACTGGTATGGAAGACACTCTCTGGTGACCCGTGGATAAAGACGCTGCAGAACCCACCGAAAGGAAACTTCAAGGCAACTATTAAGGAGTGGGAGGTGGAGCACGCATCGATAATGGCGGGCATCTGCAGTCACCAAGTGACCTTTGACACGTTCCAAAACAAAGCCAACGTTTGCTGGGCTAAGAGCTTGGTCCCTGTCCTCGAAACAGCGGGGATAAAACTAAATGATAGGCAGTGGTCCCAGATAATTCAAGCCTTCAAAGAAGACAAAGCATACTCACCCGAAGTAGCCCTGAATGAAATATGCACGCGCATGTATGGGGTGGATCTAGACAGCGGGCTATTCTCTAAACCATTGGTATCTGTGTATTACGCGGATAACCACTGGGATAATAGGCCGGGAGGAAAGATGTTCGGATTCAACCCTGAGGCAGCGTCCATTCTAGAAAGAAAGTACCCGTTTACAAAAGGAAAGTGGAACATCAACAAGCAGATCTGCGTGACTACCAGGAGGATAGAAGACTTCAACCCTACCACCAACATTATACCGGCCAACAGGAGACTACCACACTCATTAGTGGCCGAACACCGCCCGGTAAAAGGGGAAAGAATGGAATGGCTGGTTAACAAGATAAACGGACATCATGTGCTCCTGGTTAGCGGCTATAACCTTGCACTGCCTACTAAGAGAGTCACCTGGGTAGCGCCACTAGGTGTCCGCGGAGCGGACTATACATACAACCTAGAGCTGGGTCTACCGGCAACACTTGGTAGGTATGACCTAGTGGTCATAAACATCCATACACCTTTTCGCATACACCATTACCAACAGTGCGTAGATCACGCAATGAAACTGCAAATGCTAGGGGGTGACTCACTGAGACTGCTCAAACCGGGTGGCTCTCTATTGATCAGAGCATACGGTTACGCAGATAGAACCAGTGAACGAGTAATCTGCGTACTGGGACGTAAGTTTAGATCATCCAGAGCATTGAAACCACCATGTATCACCAGTAATACTGAGATGTTTTTCCTATTCAGCAGTTTTGACAATGGCAGAAGGAATTTTACAACGCATGTCATGAACAATCAACTGAACGCAGCCTTTGTAGGACAGGCCACCCGAGCAGGATGTGCACCATCGTACCGGGTAAAACGCATGGACATCGCGAAGAACGATGAAGAGTGCGTGGTCAACGCTGCCAACCCTCGCGGGTTACCGGGTGACGGTGTTTGCAAGGCAGTATATAAAAAATGGCCGGAGTCCTTTAAAAATAGTGCAACACCAGTAGGAACTGCAAAAACAGTTATGTGCGGTACATATCCAGTAATCCACGCCGTAGGACCAAACTTCTCAAATTACTCGGAGTCCGAAGGGGACCGGGAATTGGCGGCTGCCTATCGAGAAGTCGCAAAAGAAGTAACTAGACTGGGAGTAAATAGCGTAGCTATACCTCTCCTCTCCACAGGTGTATACTCAGGAGGGAAAGACAGGCTAACCCAGTCACTGAACCACCTCTTTACAGCCATGGACTCGACGGATGCAGACGTGGTCATCTACTGCCGAGACAAGGAATGGGAGAAGAAAATATCTGAGGCCATACAGATGCGGACCCAAGTGGAGCTGCTGGATGAGCACATCTCCATAGACTGCGATGTCATTCGCGTGCACCCTGACAGTAGTTTGGCAGGTAGAAAAGGATACAGCACCACGGAAGGCGCACTGTATTCATATCTAGAAGGGACACGTTTTCACCAGACGGCAGTGGATGTGGCAGAGATACATACTATGTGGCCAAAGCAAACAGAGGCCAATGAGCAAGTCTGCCTATATGCCCTGGGGGAAAGTATTGAATCGATCAGGCAGAAATGCCCGGTGGATGATGCAGATGCATCATCTCCCCCGAAAACTGTCCCGTGCCTTTGCCGTTATGCCATGACTCCTGAACGCGTCACCCGACTTCGCATGAACCATGTCACAAATATAATTGTGTGTTCTTCATTTCCCCTTCCAAAGTACAAGATAGAAGGAGTGCAAAAAGTCAAATGCTCCAAGGTAATGTTATTTGATCACAATGTGCCATCGCGCGTAAGTCCAAGGGAATACAGATCTTCCCAGGAGTCTGTACGGGAAGTGAGTATGACAACGTCATTGACGCATAGCCAGTTTGATCTAAGCGCCGATGGCGAGACACTGCCTGTCCCGTCAGACCTGGATGCTGACGCCCCAGCCCTAGAACCGGCCCTAGACGACGGGGCGATCGGAAACCTTGCGGCCGTGTCTGACTGGGTAATGAGCACCGTACCTGTCGCGCCGCCTAGAAGAAGGAGAGGGAGAAACCTGACCGTGGTATGTGACGAGAGAGAAGGGAATATAACACCCATGGCTAGCGTCCGATTCTTTAGAGCAGAGCAGTGTCCGGCCGTACAAGAAACAGCGGAGACGCGTGACACAGCTATCTCCCTTCGGGCACCGCCAAGTACCACCGTGGAACTGAGCCATCCACCGATCTCCTTCGGAGCACCAAGCGAGACGTTCCCCATCACATTTGGGGACTTCGACGATGGAGAAATCGAAAGCTTGTCTTCTGAGCTACTAACTTTCGGAGACTTCCTACCCGGTGAAGTGGATGATTTGACAGATAGCGACTGGTCCACGTGCTCAGACACGGACGACGAGTTATGACTAGACAGGGCAGGTGGGTATATATTCTCGTCGGACACTGGTCCAGGCCATTTACAACAGAAGTCGGTACGCCAGTCAGTGCTGCCGGTAAACACCCTGGAGGAAGTTCACGAGGAGAAGTGTTACCCACCTAAGCTGGATGAATTAAAGGAGCAACTACTACTTAAGAAACTCCAGGAGAGTGCGTCCACGGCCAATAGAAGCAGGTATCAATCACGCAAAGTGGAAAATATGAAAGCAACAATCATTCAGAGACTAAAGAGAGGCTGTAAACTGTATTTAATGGCAGAGACCCCGAAAGTCCCGACTTATCGGACCGTATACCCGGCACCTGTGTACTCGCCTCCGATCAATGTCCGATTGTCCAATCCCGAGTCCGCAGTGGCAGCATGTAATGAGTTCTTAGCTAGAAACTACCCAACTGTTTCATCATACCAAATCACCGATGAGTATGATGCATATCTAGACATGGTGGACGGGTCGGAGAGTTGCTTGGACCGAGCGACATTCAATCCGTCAAAACTCAGGAGCTACCCGAAACAACATGCTTATCACGCGCCTTCTATCAGAAGCGCTGTACCTTCCCCATTCCAGAACACACTACAGAATGTACTGGCAGCAGCCACGAAAAGGAACTGCAACGTCACACAGATGAGGGAATTACCCACTTTGGACTCAGCAGTATTCAACGTGGAGTGTTTTAAAAAATTTGCATGCAACCGAGAATACTGGGAAGAATTTGCTGCCAGCCCTATCAGGATAACAACTGAGAATCTAACAACCTATGTCACTAAACTAAAGGGGCCAAAAGCAGCAGCGTTGTTTGCAAGAACCCATAATCTGCTGCCGCTGCAGGATGTACCAATGGATAGGTTCACAGTAGATATGAAAAGGGACGTGAAGGTAACTCCTGGTACAAAGCATACAGAGGAAAGGCCTAAGGTGCAGGTTATACAGGCGGCTGAACCCTTGGCAACAGCGTATCTATGTGGAATTCACAGAGAATTGGTTAGGAGATTGAACGCCGTCCTCCTACCCAATGTGCATACACTATTTGACATGTCTGCCGAGGACTTCGATGCCATTATAGCCGCACACTTTAAGCCAGGAGACGCCGTTTTAGAAACGGACATAGCCTCCTTTGATAAGAGCCAGGATGATTCACTTGCGCTCACCGCCTTAATGCTGTTAGAAGATTTGGGAGTGGATCACTCCTTGTTGGACCTGATAGAGGCTGCTTTTGGAGAGATTTCCAGCTGTCACCTGCCGACAGGTACGCGCTTCAAGTTCGGCGCTATGATGAAATCCGGTATGTTCCTAACTCTGTTCGTCAACACATTGTTAAATATCACCATCGCTAGCCGGGTGTTGGAAGATCGTCTGACAAAATCTGCATGCGCGGCCTTCATCGGCGACGACAACATAATACATGGTGTCGTCTCCGATGAATTGATGGCAGCCAGATGCGCTACTTGGATGAACATGGAGGTGAAGATCATAGATGCAGTTGTATCCCAGAAAGCTCCTTACTTTTGTGGAGGGTTTATACTGCATGATACTGTGACAGGAACAGCTTGCAGGGTGGCGGACCCGCTAAAAAGGTTATTTAAACTGGGCAAACCGTTAGCGGCAGGTGACGAACAAGACGAAGACAGAAGACGGGCGCTGGCCGATGAAGTAATCAGATGGCAACGAACAGGGCTAATAGATGAGCTGGAGAAAGCGGTGTACTCTAGGTACGAAGTGCAGGGTATATCAGTTGCTGTAATGTCCATGGCCACCTTTGCAAGCTCCAGATCCAACTTCGAGAAGCTCAGAGGACCCGTCATAACTTTGTACGGCGGTCCTAAATAGGTACGCACTACAGCTACCTATTTTGCAGAAGCCGACAGCAGGTACCTAAATACCAATCAGCCATAATGGAGTTTATCCCAACCCAAACTTTCTACAATAGGAGGTACCAGCCTCGACCTTGGACTCCGCGCCCTACTATCCAAGTTATCAGACCCAGACCGCGTCCGCAAAGGAAGGCCGGGCAACTTGCCCAGCTGATCTCAGCAGTTAATAAACTGACAATGCGCGTGGTACCTCAACAGAAGCCGCGCAAGAATCGGAAGAATAAGAAGCAAAAGCAAAAGCAGCAGGCGCCACGAAACAATATGAATCAAAAGAAGCAGCCCCCTAAAAAGAAACCGGTTCAAAAGAAAAAGAAGCCGGGCCGCAGAGAGAGAATGTGCATGAAAATCGAAAATGATTGCATCTTCGAAGTCAAGCATGAAGGTAAGGTAACAGGTTACGCGTGCTTGGTAGGGGACAAAGTAATGAAGCCAGCACACGTAAAGGGGACCATCGATAACGCGGACCTGGCCAAATTGGCCTTCAAGCGATCATCTAAGTACGACCTTGAATGCGCGCAGATACCCGTGCACATGAAGTCCGACGCTTCGAAGTTCACCCATGAGAAACCGGAGGGGTACTACAACTGGCACCACGGAGCAGTACAGTACTCAGGAGGCCGATTCACCATCCCTACAGGTGCGGGCAAACCAGGGGATAGTGGTAGACCGATCTTCGACAACAAGGGGCGCGTGGTGGCCATAGTTTTAGGAGGAGCTAATGAAGGAGCCCGTACAGCCCTCTCGGTGGTGACCTGGAACAAAGACATCGTCACGAAAATCACCCCTGAGGGGGCCGAAGAGTGGAGTCTGGCCATTCCAGTTATGTGCCTGCTGGCAAATACCACGTTCCCCTGCTCCCAGCCCCCTTGCACACCCTGCTGCTACGAAAAAGAGCCGGAGAAAACCTTGCGCATGCTTGAAGACAATGTCATGAGCCCCGGGTACTATCAGCTGCTACAAGCATCCTTAACATGTTCTCCCCGACGCCAGCGGCGCAGTATTAAGGACCACTTCAATGTCTATAAAGCCACAAGACCGTACCTAGCTCACTGCCCCGACTGTGGAGAAGGGCACTCGTGCCATAGTCCCGTAGCGCTAGAACGCATCAGAAATGAAGCGACAGACGGGACGTTGAAAATCCAGGTTTCCTTGCAAATCGGAATAAAGACGGATGATAGCCATGATTGGACCAAGCTGCGTTATATGGACAATCACATGCCAGCAGACGCAGAGCGGGCCGGGCTATTTGTAAGAACGTCAGCACCGTGCACGATTACTGGAACAATGGGACACTTCATTTTGGCCCGATGTCCGAAAGGAGAAACTCTGACGGTGGGGTTCACTGACGGTAGAAAGATCAGTCACTCATGTACGCACCCATTTCACCACGACCCTCCTGTGATAGGCCGGGAAAAATTCCATTCCCGACCGCAGCACGGTAGGGAACTACCTTGCAGCACGTACGCGCAGAGCACCGCTGCAACTGCCGAGGAGATAGAGGTACATATGCCCCCAGACACCCCAGATCGCACATTAATGTCACAACAGTCCGGCAATGTAAAGATCACAGTCAATAGTCAGACGGTGCGATACAAGTGCAATTGTGGTGATTCAAGCGAAGGATTAACCACTACAGATAAAGTGATTAATAACTGCAAGGTTGATCAATGCCATGCCGCGGTCACCAATCACAAAAAATGGCAGTATAATTCCCCTCTGGTCCCGCGCAATGCTGAATCCGGGGACCGGAAAGGAAAAGTTCACATTCCATTTCCTCTGGCAAATGTGACATGCAGGGTGCCTAAAGCAAGAAACCCCACCGTGACGTACGGAAAAAACCAAGTCATCATGTTGCTGTATCCTGACCACCCAACGCTCCTGTCCTACAGGAATATGGGAGAAGAACCAAACTATCGAGAAGAGTGGGTGACGCATAAGAAGGAGATCAGGTTAACCGTGCCGACTGAAGGGCTCGAGGTCACGTGGGGTAACAATGAGCCGTACAAGTATTGGCCGCAGTTATCCACAAACGGTACAGCCCACGGCCACCCGCATGAGATAATTCTGTATTATTATGAGCTGTACCCAACTATGACTGTGGTAGTTGTGTCAGTGGCCTCGTTCATACTCCTGTCGATGGTGGGTGTGGCAGTGGGGATGTGCATGTGTGCACGACGCAGATGCATTACACCGTACGAACTGACACCAGGAGCTACCGTCCCTTTCCTGCTTAGCCTAATATGCTGCATTAGAACAGCTAAAGCGGCCACATACCAGGAGGCCGCGGTATACCTGTGGAACGAGCAGCAGCCCTTATTTTGGCTGCAAGCCCTTATTCCGCTGGCAGCCCTGATTGTCCTATGTAACTGTCTGAGACTCCTACCATGCTGTTGTAAAATGTTGACTTTTTTAGCCGTACTGAGCGTCGGTGCCCACACTGTGAGCGCGTACGAACACGTAACAGTGATCCCGAACACGGTGGGAGTACCGTATAAGACTCTAGTCAACAGACCGGGCTACAGCCCCATGGTACTGGAGATGGAGCTTCTGTCTGTCACCTTGGAACCGACGCTATCGCTTGATTACATCACGTGCGAGTATAAAACCGTTATCCCGTCTCCGTACGTGAAATGCTGCGGTACGGCAGAGTGTAAGGACAAGAGCCTACCTGATTACAGCTGTAAGGTCTTCACCGGCGTCTACCCATTCATGTGGGGCGGCGCCTACTGCTTCTGCGACACCGAGAATACGCAATTGAGCGAAGCACATGTGGAGAAGTCCGAATCATGCAAAACAGAATTTGCATCAGCATATAGGGCTCATACCGCATCCGCATCAGCTAAGCTCCGCGTTCTTTACCAAGGAAATAATATCACTGTAGCTGCTTATGCAAACGGCGACCATGCCGTCACAGTTAAGGACGCTAAATTCATAGTGGGGCCAATGTCTTCAGCCTGGACACCTTTCGACAATAAAATCGTGGTGTACAAAGGCGACGTCTACAACATGGACTACCCGCCCTTCGGCGCAGGAAGACCAGGACAATTTGGCGACATCCAAAGTCGCACGCCTGAGAGCGAAGACGTCTATGCTAATACACAACTGGTACTGCAGAGACCGTCCGCGGGTACGGTGCACGTGCCGTACTCTCAGGCACCATCTGGCTTCAAGTATTGGCTAAAAGAACGAGGGGCGTCGCTGCAGCATACAGCACCATTTGGCTGTCAAATAGCAACAAACCCGGTAAGAGCGATGAACTGCGCCGTAGGGAACATGCCTATCTCCATCGACATACCGGACGCGGCCTTTACCAGGGTCGTCGACGCGCCATCTTTAACGGATATGTCGTGTGAGGTGTCAGCCTGCACCCATTCCTCAGACTTTGGGGGCGTAGCCATCATTAAATATACAGCCAGTAAGAAAGGCAAGTGTGCAGTGCACTCGATGACTAACGCCGTCACTATTCGGGAAGCTGAAATAGAAGTAGAAGGGAACTCTCAGTTGCAAATCTCTTTTTCGACGGCCCTAGCCAGCGCCGAATTCCGCGTACAAGTCTGTTCTACACAAGTACACTGTGCAGCCGAGTGCCACCCACCGAAAGACCATATAGTCAATTACCCGGCGTCACACACCACCCCCGGGGTTCAAGACATTTCCGCTACGGCGATGTCATGGGTGCAGAAGATCACGGGAGGTGTGGGACTGGTTGTCGCTGTTGCAGCACTGATCCTAATCGTGGTGCTATGCGTGTCGTTTAGCAGGCACTAACTTGACAACTAGGTATGAAGGCATACGCGTCCCTAAAGAGACACACCACATATAGCTAAGAATCAATAGATAAGTATAGTAACAAAATATAAAAATCAACAAAAATCATAAAATAGAAAACTAGAAACAGAAGTAGGTAAGAAGGTATATGTGTCCCCTGAGAGACACACCATATATAGCTAAGAATCAATAGATAAGCATAGATCAAAGGGCTGAATAACCCCTGAATAGTAACAAAATATAAAAACCAATAAAAATCATAAAATAGAAAACCATAAACAGAAGTAGTTCAAAGGGCTATAAAACCCCTGAATAGTAACAAAATATAAAACTAACAAAAATCAAACGAATACCATAATTGGCAATCGGAAGAGATGTAGGTACTTAAGCTTCCTAAAAGCAGCCGAACTCGCTTTGAGATGTAGGCGTAGCACACCGAACTCTTCCATAATTCTCCGAACCCACAGGGACGTAGGAGATGTTCAAAGTGACTATAAAAACCCTGAACAGTAATAAAACATAAAATTAATAATGAGTACCATAATTGGCAAACGGAAGAGACGTAGGTACTAAGCTTCCTAAAAGCAGCCGAACTCACTTTGAGATGTAGGCATAGCATACCGAACTCTTCCACAATTCTCCGAACCCATAGGGACGTAGGAGATGTTATTTTGTTTTTAATATTTC

>EU703759|2006|Malaysia: Bagan Panchor, Group 1

ATGGCTGCGTGAGACACACGTAGCCTACCAGTTTCTTACTGCTCTACTCTGCAAAGCAAGAGATCAATAACCCATCATGGATTCTGTGTACGTGGATATAGACGCTGACAGCGCCTTTTTGAAGGCCCTGCAACGTGCGTACCCCATGTTTGAGGTGGAACCTAGGCAGGTCACATCAAATGACCATGCTAATGCTAGAGCGTTCTCGCATCTAGCCATAAAACTAATAGAGCAGGAAATTGATCCCGACTCAACCATCCTGGATATAGGTAGTGCGCCAGCAAGGAGGATGATGTCGGACAGGAAGTACCACTGCGTTTGCCCGATGCGCAGCGCAGAAGATCCCGAGAGACTCGCTAATTATGCGAGAAAGCTCGCATCTGCCGCAGGAAAAGTCCTGGACAGAAACATTTCTGGAAAGATCGGGGACTTACAAGCGGTGATGGCCGTGCCAGACACGGAGACGCCAACATTTTGCTTACACACAGATGTCTCATGTAGACAGAGAGCAGACGTCGCGATATACCAAGACGTCTATGCTGTACATGCACCCACGTCGCTATATCACCAGGCGATTAAAGGAGTCCGAGTGGCGTACTGGGTAGGGTTTGACACAACCCCGTTCATGTACAACGCTATGGCGGGTGCCTACCCCTCATACTCGACAAATTGGGCGGATGAGCAGGTACTGAAGGCTAAGAACATAGGATTATGTTCAACAGACCTGACGGAAGGTAGACGAGGCAAATTGTCTATCATGAGAGGGAAAAAGCTAAAACCGTGCGACCGTGTGCTGTTCTCAGTAGGGTCAACGCTTTACCCGGAAAGCCGCATGCTACTTAAGAGCTGGCACCTACCATCGGTGTTCCATCTAAAGGGCAAGCTTAGCTTCACATGCCGCTGTGACACAGTGGTTTCGTGTGAGGGCTACGTCGTCAAGAGAATAACGATGAGCCCAGGCCTTTATGGAAAAACCACGGGGTATGCGGTAACCCACCACGCAGACGGATTCTTGATGTGCAAGACTACCGATACGGTTGACGGCGAAAGAGTGTCATTCTCGGTGTGCACGTACGTGCCGGCGACCATTTGTGATCAAATGACCGGCATCCTTGCTACAGAAGTCACGCCGGAGGATGCACAGAAGCTGTTGGTGGGGCTGAACCAGAGAATAGTGGTTAACGGCAGAACGCAACGGAACACGAACACCATGAAGAACTATCTACTTCCCGTGGTCGCCCAGGCCTTCAGTAAGTGGGCGAAGGAGTGCCGGAAGGACATGGAAGATGAGAAGCTTCTGGGGGTCAGAGAAAGAACACTAACCTGCTGCTGTCTGTGGGCATTTAAGAAGCAGAAAACACACACGGTCTACAAGAGGCCTGATACCCAGTCAATCCAGAAGGTTCAGGCCGAATTTGACAGCTTTGTAGTACCAGGCCTGTGGTCGTCCGGGTTGTCAATCCCGTTGAGGACTAGAATCAAGTGGCTGTTACGCAAGGTGCCGAAAGCAGACCTGATCCCATACAGCGGAAATGCCCAAGAAGCCCAGGATGCAGAAAAAGAAGCAGAGGAAGAACGAGAAGCAGAACTGACTCATGAGGCTCTACCACCCCTACAGGCAGCACAGGAAGATGTCCAGGTCGAAATCGACGTGGAACAACTTGAGGATAGAGCTGGTGCTGGAATAATAGAGACTCCGAGAGGCGCTATTAAAGTTACTGCCCAACTAACCGACCACGTCGTGGGGGAGTACCTGGTACTTTCCCCGCAGACCGTATTACGCAGCCAGAAGCTCAGCCTGATCCACGCTTTAGCGGAGCAAGTGAAGACGTGTACGCATAGCGGACGAGCAGGGAGGTATGCGGTCGAAGCGTACGATGGCCGAGTTCTAGTGCCCTCAGGCTATGCAATTTCGCCTGAAGACTTCCAGAGTCTAAGCGAAAGCGCAACGATGGTGTACAACGAAAGAGAGTTCGTAAACAGAAAGTTACACCACATTGCGATGCACGGACCAGCCCTGAACACTGACGAAGAGTCGTATGAGCTTGTGAGGGCAGAGAGAACAGAACACGAGTACGTCTACGACGTGGACCAGAGAAGATGCTGTAAGAAGGAAGAAGCTGCAGGATTGGTACTGGTGGGCGACTTGACTAATCCGCCCTACCACGAATTTGCATACGAAGGGCTAAAAATTCGCCCCGCTTGTCCATACAAAATTGCAGTCATAGGAGTCTTCGGGGTACCAGGATCTGGTAAGTCAGCCATTATCAAGAACCTAGTTACCAGGCAAGACCTGGTGACTAGCGGAAAGAAAGAAAACTGCCAAGAAATCAGCACCGACGTGATGAGACAGAGAGGTCTAGAAATATCTGCACGTACGGTTGATTCGCTGCTCTTGAATGGATGCAATAGACCAGTCGACGTGTTGTACGTAGACGAGGCGTTTGCGTGCCACTCTGGAACGTTACTTGCCTTGATCGCCTTGGTGAGACCAAGACTGAAAGTTGTACTTTGTGGTGACCCGAAGCAGTGCGGCTTCTTCAATATGATGCAGATGAAAGTCAACTACAATCATAACATCTGCACTCAAGTGTACCACAAAAGTATCTCCAGGCGGTGTACACTGCCTGTGACTGCCATTGTGTCGTCGTTGCATTACGAAGGCAAAATGCGCACTACGAATGAGTACAACATGCCGATTGTAGTGGACACTACGGGCTCAACGAAACCTGACCCTGGAGACCTCGTGTTAACGTGCTTCAGAGGGTGGGTTAAACAACTGCAAATTGACTATCGTGGACACGAGGTCATGACAGCAGCCGCATCCCAAGGGTTAACCAGAAAAGGAGTTTACGCAGTTAGGCAAAAAGTTAACGAAAACCCACTCTATGCATCAACATCAGAGCACGTCAACGTACTCCTAACGCGTACGGAAGGTAAACTGGTATGGAAGACACTCTCTGGTGACCCGTGGATAAAGACGCTGCAGAACCCACCGAAAGGAAACTTCAAGGCAACTATTAAGGAGTGGGAGGTGGAGCACGCATCGATAATGGCGGGCATCTGCAGTCACCAAGTGACCTTTGACACGTTCCAAAACAAAGCCAACGTTTGCTGGGCTAAGAGCTTGGTCCCTGTCCTCGAAACAGCGGGGATAAAACTAAATGATAGGCAGTGGTCCCAGATAATTCAAGCCTTCAAAGAAGACAAAGCATACTCACCCGAAGTAGCCCTGAATGAAATATGCACGCGCATGTATGGGGTGGATCTAGACAGCGGGCTATTCTCTAAACCGTTGGTATCTGTGTATTACGCGGATAACCACTGGGATAATAGGCCGGGAGGAAAGATGTTCGGATTCAACCCTGAGGCAGCGTCCATTCTAGAAAGAAAGTACCCGTTTACAAAAGGAAAGTGGAACATCAACAAGCAGATCTGCGTGACTACCAGGAGGATAGAAGACTTCAACCCTACCACCAACATTATACCGGCCAACAGGAGACTACCACACTCATTAGTGGCCGAACACCGCCCAGTAAAAGGGGAAAGAATGGAATGGCTGGTTAACAAGATAAACGGACATCATGTGCTCCTGGTTAGCGGCTATAACCTTGCACTGCCTACTAAGAGAGTCACCTGGGTAGCGCCACTAGGTGTCCGCGGAGCGGACTATACATACAACCTAGAGCTGGGTCTACCGGCAACACTTGGTAGGTATGACCTAGTGGTCATAAACATCCACACACCTTTTCGCATACACCATTACCAACAGTGCGTAGATCACGCAATGAAACTGCAAATGCTAGGGGGTGACTCACTGAGACTGCTCAAACCGGGTGGCTCTCTATTGATCAGAGCATACGGTTACGCAGATAGAACCAGTGAACGAGTAATCTGCGTACTGGGACGTAAGTTTAGATCGTCCAGAGCATTGAAACCACCTTGTATCACCAGTAATACTGAGATGTTTTTCCTATTTAGCAGTTTTGACAATGGCAGAAGGAATTTTACAACGCATGTCATGAACAATCAACTGAACGCAGCCTTTGTAGGACAGGCCACCCGAGCAGGATGTGCACCATCGTACCGGGTAAAACGCATGGACATCGCGAAGAACGATGAAGAGTGCGTGGTCAACGCCGCCAACCCTCGCGGGTTACCGGGTGACGGTGTTTGCAAGGCAGTATATAAAAAATGGCCGGAGTCCTTTAAAAATAGTGCAACACCAGTAGGAACTGCAAAAACAGTTATGTGCGGTACATATCCAGTAATCCACGCCGTAGGACCAAACTTCTCAAATTACACGGAGTCCGAAGGGGACCGGGAATTGGCGGCTGCCTATCGAGAAGTCGCAAAGGAAGTAACTAGACTGGGAGTAAATAGCGTAGCTATACCTCTCCTCTCCACAGGTGTATACTCAGGAGGGAAAGACAGGCTAACCCAGTCACTGAACCACCTCTTTACAGCCATGGACTCGACGGATGCAGACGTGGTCATCTACTGCCGAGACAAGGAATGGGAGAAGAAAATATCTGAGGCCATACAGATGCGGACCCAAGTGGAGCTGCTGGATGAGCACATCTCCATAGACTGCGATGTCATTCGCGTGCACCCTGACAGTAGTTTGGCAGGTAGAAAAGGATACAGCACCACGGAAGGCGCACTGTATTCATATCTAGAAGGGACACGTTTTCACCAGACGGCAGTGGATGTGGCAGAGATACATACTATGTGGCCAAAGCAAACAGAGGCCAATGAGCAAGTCTGCCTATATGCCCTGGGGGAAAGTATTGAATCAATCAGGCAGAAATGCCCGGTGGATGATGCAGATGCATCATCTCCCCCGAAAACTGTCCCGTGCCTTTGCCGTTATGCCATGACTCCTGAACGCGTCACCCGACTTCGCATGAATCATGTCACAAATATAATTGTGTGTTCTTCATTTCCCCTTCCAAAGTACAAGATAGAAGGAGTGCAAAAAGTCAAATGCTCCAAGGTAATGTTATTTGATCACAATGTGCCATCGCGCGTAAGTCCAAGGGAATACAGATCTTCCCAGGAGTCTGTACGGGAAGTGAGTATGACAACGTCATTGACGCATAGCCAGTTTGATCTAAGCGCCGATGGCGAGACACTGCCTGTCCCGTCAGACCTGGATGCTGACGCCCCAGCCCTAGAACCGGCCCTAGACGACGGGGCGGTACATACATTACCAACCATAATCGGAAACCTTGCGGCCGTGTCTGACTGGGTAATGAGCACCGTACCTGTCGCGCCGCCTAGAAGAAGGAGAGGGAGAAACCTGACCGTGATATGTGACGAGAGAGAAGGGAATATAACACCCATGGCTAGCGTCCGATTCTTTAGAGCAGAGCAGTGTCCGGCCGTACAAGAAACAGCGGAGACGCGTGACACAGCTATTTCCCTTCGGGCACCGCCAAGTATCACCGTGGAACTGAGCCATCCACCGATCTCCTTCGGAGCACCAAGCGAGACGTTCCCCATCACATTTGGGGACTTCGACGATGGAGAAATCGAAAGCTTGTCTTCTGAGCTACTAACTTTCGGAGACTTCCTACCCGGTGAAGTGGATGATTTGACAGATAGCGACTGGTCCACGTGCTCAGACACGGACGACGAGTTATGACTAGACAGGGCAGGTGGGTATATATTCTCGTCGGACACTGGTCCAGGCCATTTACAACAGAAGTCGGTACGCCAGTCAGTGCTGCCGGTAAACACCCTGGAGGAAGTTCACGAGGAGAAGTGTTACCCACCTAAGCTGGATGAATTAAAGGAGCAACTACTACTTAAGAAACTCCAGGAGAGTGCGTCCATGGCCAATAGAAGCAGGTATCAATCACGCAAAGTGGAAAATATGAAAGCAACAATCATTCAGAGATTAAAGAGAGGCTGTAAACTGTATTTAATGGCAGAGACCCCGAAAGTCCCGACTTATCGGACCGTATACCCGGCGCCTGTGTACTCGCCTCCGATCAACGTCCGATTGTCCAATCCCGAGTCCGCAGTGGCAGCATGTAATGAGTTCTTAGCTAGAAACTACCCAACTGTTTCATCATACCAAATCACCGATGAGTATGATGCATATCTAGACATGGTGGACGGGTCGGAGAGTTGCTTGGACCGAGCGACATTCAATCCGTCAAAACTCAGGAGCTACCCGAAACAACATGCTTATCACGCGCCTTCTATCAGAAGCGCTGTACCTTCCCCATTCCAGAACACACTACAGAATGTACTGGCAGCAGCCACGAAAAGGAACTGCAACGTCACACAGATGAGGGAATTACCCACTTTGGACTCAGCAGTATTCAACGTGGAGTGTTTTAAAAAATTCGCATGCAACCGAGAATACTGGGAAGAATTTGCTGCCAGCCCTATCAGGATAACAACTGAGAATCTAACAACCTATGTCACTAAACTAAAGGGGCCAAAAGCAGCAGCGTTGTTTGCAAGAACCCATAATCTGCTGCCGCTGCAGGATGTACCAATGGATAGGTTCACAGTAGATATGAAAAGGGACGTGAAGGTAACTCCTGGTACAAAGCATACAGAGGAAAGGCCTAAGGTGCAGGTTATACAGGCGGCTGAACCCTTGGCAACAGCGTACCTATGTGGAATTCACAGAGAATTGGTTAGGAGATTGAACGCCGTCCTCCTACCCAATGTGCATACACTATTTGACATGTCTGCCGAGGACTTCGATGCCATTATAGCCGCACACTTTAAGCCAGGAGACGCCGTTTTAGAAACGGACATAGCCTCCTTTGATAAGAGCCAGGATGATTCACTTGCGCTTACCGCCTTAATGCTGTTAGAAGATTTGGGAGTGGATCACTCCCTGTTGGACCTGATAGAGGCTGCTTTTGGAGAGATTTCCAGCTGTCACCTGCCGACAGGTACGCGCTTCAAGTTCGGCGCTATGATGAAATCCGGTATGTTCCTAACTCTGTTCGTCAACACATTGTTAAATATCACCATCGCTAGCCGGGTGTTGGAAGATCGTCTGACAAAATCTGCATGCGCGGCCTTCATCGGCGACGACAACATAATACATGGTGTCGTCTCCGATGAATTGATGGCAGCCAGATGCGCTACTTGGATGAACATGGAAGTGAAGATCATAGATGCAGTTGTATCCCAGAAAGCTCCTTACTTTTGTGGAGGGTTTATACTGCATGATACTGTGACAGGAACAGCTTGCAGAGTGGCGGACCCGCTAAAAAGGTTATTTAAACTGGGCAAACCGTTAGCGGCAGGTGACGAACAAGACGAAGACAGAAGACGGGCGCTGGCCGATGAAGTAATCAGATGGCAACGAACAGGGCTAATAGATGAGCTGGAGAAAGCGGTGTACTCTAGGTACGAAGTGCAGGGTATATCAGTTGCTGTAATGTCTATGGCCACCTTTGCAAGCTCCAGATCCAACTTCGAGAAGCTCAGAGGACCCGTCATAACTTTGTACGGCGGTCCTAAATAGGTACGCACTACAGCTACCTATTTTGCAGAAGCCGACAGCAGGTACCTAAATACCAATCAGCCATAATGGAGTTTATCCCAACCCAAACTTTCTACAATAGGAGGTACCAGCCTCGACCTTGGACTCCGCGCCCTACTATCCAAGTTATCAGACCCAGACCGCGTCCGCAAAGGAAGGCCGGGCAACTTGCCCAGCTGATCTCAGCAGTTAATAAACTGACAATGCGCGCGGTACCTCAACAGAAGCCGCGCAAGAATCGGAAGAATAAGAAGCAAAAGCAAAAGCAGCAGGCGCCACGAAACAATATGAATCAAAAGAAGCAGCCCCCTAAAAAGAAACCGGTTCAAAAGAAAAAGAAGCCGGGCCGCAGAGAGAGAATGTGCATGAAAATCGAAAATGATTGCATCTTCGAAGTCAAGCATGAAGGTAAGGTAACAGGTTACGCGTGCTTGGTAGGGGACAAAGTAATGAAGCCAGCACACGTAAAGGGGACCATCGATAACGCGGACCTGGCCAAATTGGCCTTCAAGCGGTCATCTAAGTACGACCTTGAATGCGCGCAGATACCCGTGCACATGAAGTCCGACGCTTCGAAGTTCACCCATGAGAAACCGGAGGGGTACTACAACTGGCACCACGGAGCAGTACAGTACTCAGGAGGCCGATTCACCATCCCTACAGGTGCGGGCAAACCAGGGGACAGCGGTAGACCGATCTTCGACAACAAGGGGCGCGTGGTGGCCATAGTTTTAGGAGGAGCTAATGAAGGAGCCCGTACAGCCCTCTCGGTGGTGACCTGGAACAAAGACATCGTCACGAAAATCACCCCTGAGGGGGCCGAAGAGTGGAGTCTGGCCATTCCAGTTATGTGCCTGCTGGCAAATACCACGTTCCCCTGCTCCCAGCCCCCTTGCACACCCTGCTGCTACGAAAAAGAGCCGGAGAAAACCTTGCGCATGCTTGAAGACAATGTCATGAGCCCCGGGTACTATCAGCTGCTACAAGCATCCTTAACATGTTCTCCCCGACGCCGGCGGCGCAGTATTAAGGACCACTTCAATGTCTATAAAGCCACAAGACCGTACCTAGCTCACTGTCCCGACTGTGGAGAAGGGCACTCGTGCCATAGTCCCGTAGCGCTAGAACGCATCAGAAACGAAGCGACAGACGGGACGTTGAAAATCCAGGTTTCCTTGCAAATCGGAATAAAGACGGATGATAGCCATGATTGGACCAAGCTGCGTTATATGGACAATCACATGCCAGCAGACGCAGAGCGGGCCGGGCTATTTGTAAGAACGTCAGCACCGTGCACGATTACTGGAACAATGGGACACTTCATTCTGGCCCGATGTCCGAAAGGAGAAACTCTGACGGTGGGGTTCACTGATGGTAGAAAGATCAGTCACTCATGTACGCACCCATTTCACCACGACCCTCCTGTGATAGGCCGGGAAAAATTCCATTCCCGACCGCAGCACGGTAGGGAACTACCTTGCAGCACGTACGCGCAGAGCACCGCTGCAACTGCCGAGGAGATAGAGGTACATATGCCCCCAGACACCCCAGATCGCACATTAATGTCACAACAGTCCGGCAATGTAAAGATCACAGTCAATAGTCAGACGGTGCGGTACAAGTGCAATTGTGGTGACTCAAGTGAAGGATTAACCACTACAGATAAAGTGATTAATAACTGCAAGGTTGATCAATGCCATGCCGCGGTCACCAATCACAAAAAATGGCAGTATAATTCCCCTCTGGTCCCGCGCAATGCTGAATCCGGGGACCGGAAAGGAAAAGTTCACATTCCATTTCCTCTGGCAAATGTGACATGCAGGGTGCCTAAAGCAAGAAACCCCACCGTGACGTACGGAAAAAACCAAGTCATCATGTTGCTGTATCCTGACCACCCAACGCTCCTGTCCTACAGGAATATGGGAGAAGAACCAAACTATCAAGAAGAGTGGGTGACGCATAAGAAGGAGATCAGGTTAACCGTGCCGACTGAAGGGCTCGAGGTCACGTGGGGTAACAATGAGCCGTACAAGTATTGGCCGCAGTTATCCACAAACGGTACAGCCCACGGCCACCCGCATGAGATAATTCTGTATTATTATGAGCTGTACCCAACTATGACTGTGGTAGTTGTGTCAGTGGCCTCGTTCATACTCCTGTCGATGGTGGGTGTGGCAGTGGGGATGTGCATGTGTGCACGACGCAGATGCATTACACCGTACGAACTGACACCAGGAGCTACCGTCCCTTTCCTGCTTAGCCTAATATGCTGCATTAGAACAGCTAAAGCGGCCACATACCAGGAGGCCGCGGTATACCTGTGGAACGAGCAGCAGCCTTTATTTTGGCTGCAAGCCCTTATTCCGCTGGCAGCCCTGATTGTCCTATGTAACTGTCTGAGACTCTTACCATGCTGTTGTAAAATGTTGACTTTTTTAGCCGTACTGAGCGTCGGTGCCCACACTGTGAGCGCGTACGAACACGTAACAGTGATCCCGAACACGGTGGGAGTACCGTATAAGACTCTAGTCAACAGACCGGGCTACAGCCCCATGGTACTGGAGATGGAGCTTCTGTCTGTCACTTTGGAGCCAACGCTATCGCTTGATTACATCACGTGCGAGTATAAAACCGTTATCCCGTCTCCGTACGTGAAATGCTGCGGTACAGCAGAGTGTAAGGACAAGAGCCTACCTGATTACAGCTGTAAGGTCTTCACCGGCGTCTACCCATTCATGTGGGGCGGCGCCTACTGCTTCTGCGACACCGAAAATACGCAATTGAGCGAAGCACATGTGGAGAAGTCCGAATCATGCAAAACAGAATTTGCATCAGCATACAGGGCTCATACCGCATCCGCATCAGCTAAGCTCCGCGTCCTTTACCAAGGAAATAATATCACTGTAGCTGCTTATGCAAACGGCGACCATGCCGTCACAGTTAAGGACGCCAAATTCATAGTGGGGCCAATGTCTTCAGCCTGGACACCTTTCGACAATAAAATCGTGGTGTACAAAGGCGACGTCTACAACATGGACTACCCGCCCTTCGGCGCAGGAAGACCAGGACAATTTGGCGACATCCAAAGTCGCACGCCTGAGAGCGAAGACGTCTATGCTAATACACAACTGGTACTGCAGAGACCGTCCGCGGGTACGGTGCACGTGCCGTACTCTCAGGCACCATCTGGCTTCAAGTATTGGCTAAAAGAACGAGGGGCGTCGCTACAGCACACAGCACCATTTGGCTGTCAAATAGCAACAAACCCGGTAAGAGCGATGAACTGCGCCGTAGGGAACATGCCTATCTCCATCGACATACCGGACGCGGCCTTTACCAGGGTCGTCGACGCGCCATCTTTAACGGACATGTCGTGTGAGGTATCAGCCTGCACCCATTCCTCAGACTTTGGGGGCGTAGCCATCATTAAATATGCAGCCAGTAAGAAAGGCAAGTGTGCAGTGCACTCGATGACTAACGCCGTCACTATTCGGGAAGCTGAAATAGAAGTGGAAGGGAACTCTCAGTTGCAAATCTCTTTTTTGACGGCCCTAGCCAGCGCCGAATTCCGCGTACAAGTCTGTTCTACACAAGTACACTGTGCAGCCGAGTGCCATCCACCGAAAGACCATATAGTCAATTACCCGGCGTCACACACCACCCCCGGGGTCCAAGACATTTCCGCTACGGCGATGTCATGGGTGCAGAAGATCACGGGAGGTGTGGGACTGGTTGTCGCTGTTGCAGCACTGATCCTAATCGTGGTGCTATGCGTGTCGTTTAGCAGGCACTAACTTGACAACTAGGTATGAAGGCATACGCGTCCCTAAAGAGACACACCACATATAGCTAAGAATCAATAGATAAGTATAGATCAAAGGGCTGAACAATCCCTGAATAGTAACAAAATATAAAAATCAACAAAAATCATAAAATAGAAAACTAGAAATAGAAGTAGGTAAGAAGGTATATGTGTCCCCTAAGAGACACACCATATATAGCTAAGAATCAATAGATAAGCATAGATCAAAGGGCTGAATAACCCCTGAATAATAACAAAATATAAAAACCAATAAAAATCATAAAATAGAAAACCATAAACAGAAGTAGTTCAAAGGGCTATGAAACCCCTGAATAGTAACAAAATATAAAACTAATAAAAATCAAACGAATACCATAATTGGCAATCGGAAGAGATGTAGGTACTTAAGCTTCCTAAAAGCAGCCGAACTCGCTTTGAGATGTAGGCGTAGCACACCGAACTCTTCCACGATTCTCCGAACCCACAGGGACGTAGGAGAAGTTCAAAGTGACTATAAAAACCCTGAACAGTAATAAAACATAAAATTAATAATGAGTACCATAATTGGCAAACGGAAGAGACGTAGGTACTAAGCTTCCTAAAAGCAGCCGAACTCACTTTGAGATGTAGGCATAGCATACCGAACTCTTCCACAATTCTCCGTACCCATAGGGACGTAGGAGATGTTATTTTGTTTTTAATATTTC

>EU703760|2006|Malaysia: Bagan Panchor, Group 1

ATGGCTGCGTGAGATACACGTAGCCTATCAGTTTCTTACTGCTCTACTCTGCAAAGCAAGAGATCAATAACCCATCATGGATTCTGTGTACGTGGATATAGACGCTGACAGCGCCTTTTTGAAGGCCCTGCAACGTGCGTACCCCATGTTTGAGGTGGAACCTAGGCAGGTCACATCAAATGACCATGCTAATGCTAGAGCGTTCTCGCATCTAGCCATAAAACTAATAGAGCAGGAAATTGATCCCGACTCAACCATCCTGGATATAGGTAGTGCGCCAGCAAGGAGGATGATGTCGGACAGGAAGTACCACTGCGTTTGCCCGATGCGCAGCGCAGAAGATCCCGAGAGACTCGCTAATTATGCGAGAAAGCTCGCATCTGCCGCAGGAAAAGTCCTGGACAGAAACATTTCTGGAAAGATCGGGGACTTACAAGCGGTGATGGCCGTGCCAGACACGGAGACGCCAACATTTTGCTTACACACAGATGTCTCATGTAGACAGAGAGCAGACGTCGCGATATACCAAGACGTCTATGCTGTACATGCACCCACGTCGCTATATCACCAGGCGATTAAAGGAGTCCGAGTGGCGTACTGGGTAGGGTTTGACACAACCCCGTTCATGTACAACGCTATGGCGGGTGCCTACCCCTCATACTCGACAAATTGGGCGGATGAGCAGGTACTGAAGGCTAAGAACATAGGATTATGTTCAACAGACCTGACGGAAGGTAGACGAGGCAAATTGTCTATCATGAGAGGGAAAAAGCTAAAACCGTGCGACCGTGTGCTGTTCTCAGTAGGGTCAACGCTTTACTCGAAAAGCCGCATGCTACTTAAGAGCTGGCACCTACCATCGGTGTTCCATCTAAAGGGCAAGCTTAGCTTCACATGCCGCTGTGACACAGTGGTTTCGTGTGAGGGCTACGTCGTCAAGAGAATAACGATGAGCCCAGGCCTTTATGGAAAAACCACGGGGTATGCGGTAACCCACCACGCAGACGGATTCTTGATGTGCAAGACTACCGATACGGTTGACGGCGAAAGAGTGTCATTCTCGGTGTGCACGTACGTGCCGGCGACCATTTGTGATCAAATGACCGGCATCCTTGCTACAGAAGTCACGCCGGAGGATGCACAGAAGCTGTTGGTGGGGCTGAACCAGAGAATAGTGGTTAACGGCAGAACGCAACGGAACACGAACACCATGAAGAACTATCTACTTCCCGTGGTCGCCCAGGCCTTCAGTAAGTGGGCGAAGGAGTGCCGGAAGGACATGGAAGATGAGAAGCTTCTGGGGGTCAGAGAAAGAACACTAACCTGCTGCTGTCTGTGGGCATTTAAGAAGCAGAAAACACACACGGTCTACAAGAGGCCTGATACCCAGTCAATCCAGAAGGTTCAGGCCGAATTTGACAGCTTTGTAGTACCAGGCCTGTGGTCGTCCGGGTTGTCAATCCCGTTGAGGACTAGAATCAAGTGGCTGTTACGCAAGGTGCCGAAAGCAGACCTGATCCCATACAGCGGAAATGCCCAAGAAGCCCAGGATGCAGAAAAAGAAGCAGAGGAAGAACGAGAAGCAGAACTGACTCATGAGGCTCTACCACCCCTACAGGCAGCACAGGAAGATGTCCAGGTCGAAATCGACGTGGAACAACTTGAGGATAGAGCTGGTGCTGGAATAATAGAGACTCCGAGAGGCGCTATTAAAGTTACTGCCCAACTAACCGACCACGTCGTGGGGGAGTACCTGGTACTTTCCCCGCAGACCGTATTACGCAGCCAGAAGCTCAGCCTGATCCACGCTTTAGCGGAGCAAGTGAAGACGTGTACGCATAGCGGACGAGCAGGGAGGTATGCGGTCGAAGCGTACGATGCCCGAGTCCTAGTGCCCTCAGGCTATGCAATTTCGCCTGAAGACTTCCAGAGTCTAAGCGAAAGCGCAACGATGGTGTACAACGAAAGAGAGTTCGTAAACAGAAAGTTACACCACATTGCGATGCACGGACCAGCCCTGAACACTGACGAAGAGTCGTATGAGCTTGTGAGGGCAGAGAGAACAGAACACGAGTACGTCTACGACGTGGACCAGAGAAGATGCTGTAAGAAGGAAGAAGCTGCAGGATTGGTACTGGTGGGCGACTTGACTAATCCGCCCTACCACGAATTTGCATACGAAGGGCTAAAAATTCGCCCCGCTTGTCCATACAAAATTGCAGTCATAGGAGTCTTCGGGGTACCAGGATCTGGTAAGTCAGCCATTATCAAGAACCTAGTTACCAGGCAAGACCTGGTGACTAGCGGAAAGAAAGAAAACTGCCAAGAAATCAGCACCGACGTGATGAGACAGAGAGGTCTAGAAATATCTGCACGTACGGTTGATTCGCTGCTCTTGAATGGATGCAATAGACCAGTCGACGTGTTGTACGTAGACGAGGCGTTTGCGTGCCACTCTGGAACGTTACTTGCCTTGATCGCCTTGGTGAGACCAAGACTGAAAGTTGTACTTTGTGGTGACCCGAAGCAGTGCGGCTTCTTCAATATGATGCAGATGAAAGTCAACTACAATCATAACATCTGCACTCAAGTGTACCACAAAAGTATCTCCAGGCGGTGTACACTGCCTGTGACTGCCATTGTGTCGTCGTTGCATTACGAAGGCAAAATGCGCACTACGAATGAGTACAACATGCCGATTGTAGTGGACACTACGGGCTCAACGAAACCTGACCCTGGAGACCTCGTGTTAACGTGCTTCAGAGGGTGGGTTAAACAACTGCAAATTGACTATCGTGGACACGAGGTCATGACAGCAGCCGCATCCCAAGGGTTAACCAGAAAAGGAGTTTACGCAGTTAGGCAAAAAGTTAACGAAAACCCACTCTATGCATCAACATCAGAGCACGTCAACGTACTCCTAACGCGTACGGAAGGTAAACTGGTATGGAAGACACTCTCTGGTGACCCGTGGATAAAGACGCTGCAGAACCCACCGAAAGGAAACTTCAAGGCAACTATTAAGGAGTGGGAGGTGGAGCACGCATCGATAATGGCGGGCATCTGCAGTCACCAAGTGACCTTTGACACGTTCCAAAACAAAGCCAACGTTTGCTGGGCTAAGAGCTTGGTCCCTGTCCTCGAAACAGCGGGGATAAAACTAAATGATAGGCAGTGGTCCCAGATAATTCAAGCCTTCAAAGAAGACAAAGCATACTCACCCGAAGTAGCCCTGAATGAAATATGCACGCGCATGTATGGGGTGGATCTAGACAGCGGGCTATTCTCTAAACCGTTGGTATCTGTGTATTACGCGGATAACCACTGGGATAATAGGCCGGGAGGAAAGATGTTCGGATTCAACCCTGAGGCAGCGTCCATTCTAGAAAGAAAGTACCCGTTTACAAAAGGAAAGTGGAACATCAACAAGCAGATCTGCGTGACTACCAGGAGGATAGAAGACTTCAACCCTACCACCAACATTATACCGGCCAACAGGAGACTACCACACTCATTAGTGGCCGAACACCGCCCAGTAAAAGGGGAAAGAATGGAATGGCTGGTTAACAAGATAAACGGACATCATGTGCTCCTGGTTAGCGGCTATAACCTTGCACTGCCTACTAAGAGAGTCACCTGGGTAGCGCCACTAGGTGTCCGCGGAGCGGACTATACATACAACCTAGAGCTGGGTCTACCGGCAACACTTGGTAGGTATGACCTAGTGGTCATAAACATCCACACACCTTTTCGCATACACCATTACCAACAGTGCGTAGATCACGCAATGAAACTGCAAATGCTAGGGGGTGACTCACTGAGACTGCTCAAACCGGGTGGCTCTCTATTGATCAGAGCATACGGTTACGCAGATAGAACCAGTGAACGAGTAATCTGCGTACTGGGACGTAAGTTTAGATCGTCCAGAGCATTGAAACCACCTTGTATCACCAGTAATACTGAGATGTTTTTCCTATTTAGCAGTTTTGACAATGGCAGAAGGAATTTTACAACGCATGTCATGAACAATCAACTGAACGCAGCCTTTGTAGGACAGGCCACCCGAGCAGGATGTGCACCATCGTACCGGGTAAAACGCATGGACATCGCGAAGAACGATGAAGAGTGCGTGGTCAACGCCGCCAACCCTCGCGGGTTACCGGGTGACGGTGTTTGCAAGGCAGTATATAAAAAATGGCCGGAGTCCTTTAAAAATAGTGCAACACCAGTAGGAACTGCAAAAACAGTTATGTGCGGTACATATCCAGTAATCCACGCCGTAGGACCAAACTTCTCAAATTACACGGAGTCCGAAGGGGACCGGGAATTGGCGGCTGCCTATCGAGAAGTCGCAAAGGAAGTAACTAGACTGGGAGTAAATAGCGTAGCTATACCTCTCCTCTCCACAGGTGTATACTCAGGAGGGAAAGACAGGCTAACCCAGTCACTGAACCACCTCTTTACAGCCATGGACTCGACGGATGCAGACGTGGTCATCTACTGCCGAGACAAGGAATGGGAGAAGAAAATATCTGAGGCCATACAGATGCGGACCCAAGTGGAGCTGCTGGATGAGCACATCTCCATAGACTGCGATGTCATTCGCGTGCACCCTGACAGTAGTTTGGCAGGTAGAAAAGGATACAGCACCACGGAAGGCGCACTGTATTCATATCTAGAAGGGACACGTTTTCACCAGACGGCAGTGGATGTGGCAGAGATACATACTATGTGGCCAAAGCAAACAGAGGCCAATGAGCAAGTCTGCCTATATGCCCTGGGGGAAAGTATTGAATCAATCAGGCAGAAATGCCCGGTGGATGATGCAGATGCATCATCTCCCCCGAAAACTGTCCCGTGCCTTTGCCGTTATGCCATGACTCCTGAACGCGTCACCCGACTTCGCATGAACCATGTCACAAATATAATTGTGTGTTCTTCATTTCCCCTTCCAAAGTACAAGATAGAAGGAGTGCAAAAAGTCAAATGCTCCAAGGTAATGTTATTTGATCACAATGTGCCATCGCGCGTAAGTCCAAGGGAATACAGATCTTCCCAGGAGTCTGTACGGGAAGTGAGTATGACAACGTCATTGACGCATAGCCAGTTTGATCTAAGCGCCGATGGCGAGACACTGCCTGTCCCGTCAGACCTGGATGCTGACGCCCCAGCCCTAGAACCGGCCCTAGACGACGGGGCGGTACATACATTACCAACCATAATCGGAAACCTTGCGGCCGTGTCTGACTGGGTAATGAGCACCGTACCTGTCGCGCCGCCTAGAAGAAGGAGAGGGAGAAACCTGACCGTGATATGTGACGAGAGAGAAGGGAATATAACACCCATGGCTAGCGTCCGATTCTTTAGAGCAGAGCAGTGTCCGGCCGTACAAGAAACAGCGGAGACGCGTGACACAGCTATTTCCCTTCGGGCACCGCCAAGTATCACCGTGGAACTGAGCCATCCACCGATCTCCTTCGGAGCACCAAGCGAGACGTTCCCCATCACATTTGGGGACTTCGACGATGGAGAAATCGAAAGCTTGTCTTCTGAGCTACTAACTTTCGGAGACTTCCTACCCGGTGAAGTGGATGATTTGACAGATAGCGACTGGTCCACGTGCTCAGACACGGACGACGAGTTATGACTAGACAGGGCAGGTGGGTATATATTCTCGTCGGACACTGGTCCAGGCCATTTACAACAGAAGTCGGTACGCCAGTCAGTGCTGCCGGTAAACACCCTGGAGGAAGTTCACGAGGAGAAGTGTTACCCACCTAAGCTGGATGAATTAAAGGAGCAACTACTACTTAAGAAACTCCAGGAAAGTGCGTCCATGGCCAATAGAAGCAGGTATCAATCACGCAAAGTGGAAAATATGAAAGCAACAATCATTCAGAGATTAAAGAGAGGCTGTAAACTGTATTTAATGGCAGAGACCCCGAAAGTCCCGACTTATCGGACCGTATACCCGGCGCCTGTGTACTCGCCTCCGATCAACGTCCGATTGTCCAATCCCGAGTCCGCAGTGGCAGCATGTAATGAGTTCTTAGCTAGAAACTACCCAACTGTTTCATCATACCAAATCACCGATGAGTATGATGCATATCTAGACATGGTGGACGGGTCGGAGAGTTGCTTGGACCGAGCGACATTCAATCCGTCAAAACTCAGGAGCTACCCGAAACAACATGCTTATCACGCGCCTTCTATCAGAAGCGCTGTACCTTCCCCATTCCAGAACACACTACAGAATGTACTGGCAGCAGCCACGAAAAGGAACTGCAACGTCACACAGATGAGGGAATTACCCACTTTGGACTCAGCAGTATTCAACGTGGAGTGTTTTAAAAAATTCGCATGCAACCGAGAATACTGGGAAGAATTTGCTGCCAGCCCTATCAGGATAACAACTGAGAATCTAACAACCTATGTCACTAAACTAAAGGGGCCAAAAGCAGCAGCGTTGTTTGCAAGAACCCATAATCTGCTGCCGCTGCAGGATGTACCAATGGATAGGTTCACAGTAGATATGAAAAGGGACGTGAAGGTAACTCCTGGTACAAAGCATACAGAGGAAAGGCCTAAGGTGCAGGTTATACAGGCGGCTGAACCCTTGGCAACAGCGTACCTATGTGGAATTCACAGAGAATTGGTTAGGAGATTGAACGCCGTCCTCCTACCCAATGTGCATACACTATTTGACATGTCTGCCGAGGACTTCGATGCCATTATAGCCGCACACTTTAAGCCAGGAGACGCCGTTTTAGAAACGGACATAGCCTCCTTTGATAAGAGCCAGGATGATTCACTTGCGCTTACCGCCTTAATGCTGTTAGAAGATTTGGGAGTGGATCACTCCCTGTTGGACCTGATAGAGGCTGCTTTTGGAGAGATTTCCAGCTGTCACCTGCCGACAGGTACGCGCTTCAAGTTCGGCGCTATGATGAAATCCGGTATGTTCCTAACTCTGTTCGTCAACACATTGTTAAATATCACCATCGCTAGCCGGGTGTTGGAAGATCGTCTGACAAAATCTGCATGCGCGGCCTTCATCGGCGACGACAACATAATACATGGTGTCGTCTCCGATGAATTGATGGCAGCCAGATGCGCTACTTGGATGAACATGGAAGTGAAGATCATAGATGCAGTTGTATCCCAGAAAGCTCCTTACTTTTGTGGAGGGTTTATACTGCATGATACTGTGACAGGAACAGCTTGCAGGGTGGCGGACCCGCTAAAAAGGTTATTTAAACTGGGCAAACCGTTAGCGGCAGGTGACGAACAAGACGAAGACAGAAGACGGGCGCTGGCCGATGAAGTAATCAGATGGCAACGAACAGGGCTAATAGATGAGCTGGAGAAAGCGGTGTACTCTAGGTACGAAGTGCAGGGTATATCAGTTGCTGTAATGTCTATGGCCACCTTTGCAAGCTCCAGATCCAACTTCGAGAAGCTCAGAGGACCCGTCATAACTTTGTACGGCGGTCCTAAATAGGTACGCACTACAGCTACCTATTTTGCAGAAGCCGACAGCAGGTACCTAAATACCAATCAGCCATAATGGAGTTTATCCCAACCCAAACTTTCTACAATAGGAGGTACCAGCCTCGACCTTGGACTCCGCGCCCTACTATCCAAGTTATCAGACCCAGACCGCGTCCGCAAAGGAAGGCCGGGCAACTTGCCCAGCTGATCTCAGCAGTTAATAAACTGACAATGCGCGCGGTACCTCAACAGAAGCCGCGCAAGAATCGGAAGAATAAGAAGCAAAAGCAAAAGCAGCAGGCGCCACGAAACAATATGAATCAAAAGAAGCAGCCCCCTAAAAAGAAACCGGTTCAAAAGAAAAAGAAGCCGGGCCGCAGAGAGAGAATGTGCATGAAAATCGAAAATGATTGCATCTTTGAAGTCAAGCATGAAGGTAAGGTAACAGGTTACGCGTGCTTGGTAGGGGACAAAGTAATGAAGCCAGCACACGTAAAGGGGACCATCGATAACGCGGACCTGGCCAAATTGGCCTTCAAGCGGTCATCTAAGTACGACCTTGAATGCGCGCAGATACCCGTGCACATGAAGTCCGACGCTTTGAAGTTCACCCATGAGAAACCGGAGGGGTACTACAACTGGCACCACGGAGCAGTACAGTACTCAGGAGGCCGATTCACCATCCCTACAGGTGCGGGCAAACCAGGGGATAGTGGTAGACCGATCTTCGACAACAAGGGGCGCGTGGTGGCCATAGTTTTAGGAGGAGCTAATGAAGGAGCCCGTACAGCCCTCTCGGTGGTGACCTGGAACAAAGACATCGTCACGAAAATCACCCCTGAGGGGGCCGAAGAGTGGAGTCTGGCCATTCCAGTTATGTGCCTGCTGGCAAATACCACGTTCCCCTGCTCCCAGCCCCCTTGCACACCCTGCTGCTACGAAAAAGAGCCGGAGAAAACCTTGCGCATGCTTGAAGACAATGTCATGAGCCCCGGGTACTATCAGCTGCTACAAGCATCCTTAACATGTTCTCCCCGACGCCAGCGGCGCAGTATTAAGGACCACTTCAATGTCTATAAAGCCACAAGACCGTACCTAGCTCACTGTCCCGACTGTGGAGAAGGGCACTCGTGCCATAGTCCCGTAGCGCTAGAACGCATCAGAAACGAAGCGACAGACGGGACGTTGAAAATCCAGGTTTCCTTGCAAATCGGAATAAAGACGGATGATAGCCATGATTGGACCAAGCTGCGTTATATGGACAATCACATGCCAGCAGACGCAGAGCGGGCCGGGCTATTTGTAAGAACGTCAGCACCGTGCACGATTACTGGAACAATGGGACACTTCATTCTGGCCCGATGTCCGAAAGGAGAAACTCTGACGGTGGGGTTCACTGATGGTAGAAAGATCAGTCACTCATGTACGCACCCATTTCACCACGACCCTCCTGTGATAGGCCGGGAAAAATTCCATTCCCGACCGCAGCACGGTAGGGAACTACCTTGCAGCACGTACGCGCAGAGCACCGCTGCAACTGCCGAGGAGATAGAGGTACATATGCCCCCAGACACCCCAGATCGCACATTAATGTCACAACAGTCCGGCAATGTAAAGATCACAGTCAATAGTCAGACGGTGCGGTACAAGTGCAATTGTGGTGACTCAAGTGAAGGATTAACCACTACAGATAAAGTGATTAATAACTGCAAGGTTGATCAATGCCATGCCGCGGTCACCAATCACAAAAAATGGCAGTATAATTCCCCTCTGGTCCCGCGCAATGCTGAATCCGGGGACCGGAAAGGAAAAGTTCACATTCCATTTCCTCTGGCAAATGTGACATGCAGGGTGCCTAAAGCAAGAAACCCCACCGTGACGTACGGAAAAAACCAAGTCATCATGTTGCTGTATCCTGACCACCCAACGCTCCTGTCCTACAGGAATATGGGAGAAGAACCAAACTATCAAGAAGAGTGGGTGACGCATAAGAAGGAGATCAGGTTAACCGTGCCGACTGAAGGGCTCGAGGTCACGTGGGGTAACAATGAGCCGTACAAGTATTGGCCGCAGTTATCCACAAACGGTACAGCCCACGGCCACCCGCATGAGATAATTCTGTATTATTATGAGCTGTACCCAACTATGACTGTGGTAGTTGTGTCAGTGGCCTCGTTCATACTCCTGTCGATGGTGGGTGTGGCAGTGGGGATGTGCATGTGTGCACGACGCAGATGCATTACACCGTACGAACTGACACCAGGAGCTACCGTCCCTTTCCTGCTTAGCCTAATATGCTGCATTAGAACAGCTAAAGCGGCCACATACCAGGAGGCCGCGGTATACCTGTGGAACGAGCAGCAGCCTTTATTTTGGCTGCAAGCCCTTATTCCGCTGGCAGCCCTGATTGTCCTATGTAACTGTCTGAGACTCTTACCATGCTGTTGTAAAATGTTGACTTTTTTAGCCGTACTGAGCGTCGGTGCCCACACTGTGAGCGCGTACGAACACGTAACAGTGATCCCGAACACGGTGGGAGTACCGTATAAGACTCTAGTCAACAGACCGGGCTACAGCCCCATGGTACTGGAGATGGAGCTTCTGTCTGTCACTTTGGAGCCAACGCTATCGCTTGATTACATCACGTGCGAGTATAAAACCGTTATCCCGTCTCCGTACGTGAAATGCTGCGGTACAGCAGAGTGTAAGGACAAGAGCCTACCTGATTACAGCTGTAAGGTCTTCACCGGCGTCTACCCATTCATGTGGGGCGGCGCCTACTGCTTCTGCGACACCGAAAATACGCAATTGAGCGAAGCACATGTGGAGAAGTCCGAATCATGCAAAACAGAATTTGCATCAGCATACAGGGCTCATACCGCATCCGCATCAGCTAAGCTCCGCGTCCTTTACCAAGGAAATAATATCACTGTAGCTGCTTATGCAAACGGCGACCATGCCGTCACAGTTAAGGACGCCAAATTCATAGTGGGGCCAATGTCTTCAGCCTGGACACCTTTCGACAATAAAATCGTGGTGTACAAAGGCGACGTCTACAACATGGACTACCCGCCCTTCGGCGCAGGAAGACCAGGACAATTTGGCGACATCCAAAGTCGCACGCCTGAGAGCGAAGACGTCTATGCTAATACACAACTGGTACTGCATAGACCGTCCGCGGGTACGGTGCACGTGCCGTACTCTCAGGCACCATCTGGCTTCAAGTATTGGCTAAAAGAACGAGGGGCGTCGCTACAGCACACAGCACCATTTGGCTGTCAAATAGCAACAAACCCGGTAAGAGCGATAAACTGCGCCGTAGGGAACATGCCTATCTCCATCGACATACCGGACGCGGCCTTTACCAGGGTCGTCGACGCGCCATCTTTAACGGACATGTCGTGTGAGGTATCAGCCTGCACCCATTCCTCAGACTTTGGGGGCGTAGCCATCATTAAATATGCAGCCAGTAAGAAAGGCAAGTGTGCAGTGCACTCGATGACTAACGCCGTCACTATTCGGGAAGCTGAAATAGAAGTGGAAGGGAACTCTCAGTTGCAAATCTCTTTTTTGACGGCCCTAGCCAGCGCCGAATTCCGCGTACAAGTCTGTTCTACACAAGTACACTGTGCAGCCGAGTGCCATCCACCGAAAGACCATATAGTCAATTACCCGGCGTCACACACCACCCCCGGGGTCCAAGACATTTCCGCTACGGCGATGTCATGGGTGCAGAAGATCACGGGAGGTGTGGGACTGGTTGTCGCTGTTGCAGCACTGATCCTAATCGTGGTGCTATGCGTGTCGTTTAGCAGGCACTAACTTGACAACTAGGTATGAAGGCATACGCGTCCCTAAAGAGACACACCACATATAGCTAAGAATCAATAGATAAGTATAGATCAAAGGGCTGAACAATCCCTGAATAGTAACAAAATATAAAAATCAACAAAAATCATAAAATAGAAAACTAGAAATAGAAGTAGGTAAGAAGGTATATGTGTCCCCTAAGAGACACACCATATATAGCTAAGAATCAATAGATAAGCATAGATCAAAGGGCTGAATAACCCCTGAATAATAACAAAATATAAAAACCAATAAAAATCATAAAATAGAAAACCATAAACAGAAGTAGTTCAAAGGGCTATGAAACCCCTGAATAGTAACAAAATATAAAACTAATAAAAATCAAACGAATACCATAATTGGCAATCGGAAGAGATGTAGGTACTTAAGCTTCCTAAAAGCAGCCGAACTCGCTTTGAGATGTAGGCGTAGCACACCGAACTCTTCCACGATTCTCCGAACCCACAGGGACGTAGGAGAAGTTCAAAGTGACTATAAAAACCCTGAACAGTAATAAAACATAAAATTAATAATGAGTACCATAATTGGCAAACGGAAGAGACGTAGGTACTAAGCTTCCTAAAAGCAGCCGAACTCACTTTGAGATGTAGGCATAGCATACCGAACTCTTCCACAATTCTCCGTACCCATAGGGACGTAGGAGATGTTATTTTGTTTTTAATATTTC

>EU703761|2006|Malaysia: Bagan Panchor, Group 1

ATGGCTGCGTGAGACTCACGTAGCCTACCAGTTTCTTACTGCTCTACTCTGCAAAGCAAGAGATCAATAACCCATCATGGATTCTGTGTACGTGGATATAGACGCTGACAGCGCCTTTTTGAAGGCCCTGCAACGTGCGTACCCCATGTTTGAGGTGGAACCTAGGCAGGTCACATCAAATGACCATGCTAATGCTAGAGCGTTCTCGCATCTAGCCATAAAACTAATAGAGCAGGAAATTGATCCCGACTCAACCATCCTGGATATAGGTAGTGCGCCAGCAAGGAGGATGATGTCGGACAGGAAGTACCACTGCGTTTGCCCGATGCGCAGCGCAGAAGATCCCGAGAGACTCGCTAATTATGCGAGAAAGCTCGCATCTGCCGCAGGAAAAGTCCTGGACAGAAACATTTCTGGAAAGATCGGGGACTTACAAGCGGTGATGGCCGTGCCAGACACGGAGACGCCAACATTTTGCTTACACACAGATGTCTCATGTAGACAGAGAGCAGACGTCGCGATATACCAAGACGTCTATGCTGTACATGCACCCACGTCGCTATATCACCAGGCGATTAAAGGAGTCCGAGTGGCGTACTGGGTAGGGTTTGACACAACCCCGTTCATGTACAACGCTATGGCGGGTGCCTACCCCTCATACTCGACAAATTGGGCGGATGAGCAGGTACTGAAGGCTAAGAACATAGGATTATGTTCAACAGACCTGACGGAAGGTAGACGAGGCAAATTGTCTATCATGAGAGGGAAAAAGCTAAAACCGTGCGACCGTGTGCTGTTCTCAGTAGGGTCAAAGCTTTACCCGGAAAGCCGCATGCTACTTAAGAGCTGGCACCTACCATCGGTGTTCCATCTAAAGGGCAAGCTTAGCTTCACATGCCGCTGTGACACAGTGGTTTCGTGTGAGGGCTACGTCGTCAAGAGAATAACGATGAGCCCAGGCCTTTATGGAAAAACCACGGGGTATGCGGTAACCCACCACGCAGACGGATTCTTGATGTGCAAGACTACCGATACGGTTGACGGCGAAAGAGTGTCATTCTCGGTGTGCACGTACGTGCCGGCGACCATTTGTGATCAAATGACCGGCATCCTTGCTACAGAAGTCACGCCGGAGGATGCACAGAAGCTGTTGGTGGGGCTGAACCAGAGAATAGTGGTTAACGGCAGAACGCAACGGAACACGAACACCATGAAGAACTATCTACTTCCCGTGGTCGCCCAGGCCTTCAGTAAGTGGGCGAAGGAGTGCCGGAAGGACATGGAAGATGAGAAGCTTCTGGGGGTCAGAGAAAGAACACTAACCTGCTGCTGTCTGTGGGCATTTAAGAAGCAGAAAACACACACGGTCTACAAGAGGCCTGATACCCAGTCAATCCAGAAGGTTCAGGCCGAATTTGACAGCTTTGTAGTACCAGGCCTGTGGTCGTCCGGGTTGTCAATCCCGTTGAGGACTAGAATCAAGTGGCTGTTACGCAAGGTGCCGAAAGCAGACCTGATCCCATACAGCGGAAATGCCCAAGAAGCCCAGGATGCAGAAAAAGAAGCAGAGGAAGAACGAGAAGCAGAACTGACTCATGAGGCTCTACCACCCCTACAGGCAGCACAGGAAGATGTCCAGGTCGAAATCGACGTGGAACAACTTGAGGATAGAGCTGGTGCTGGAATAATAGAGACTCCGAGAGGCGCTATTAAAGTTACTGCCCAACTAACCGACCACGTCGTGGGGGAGTACCTGGTACTTTCCCCGCAGACCGTATTACGCAGCCAGAAGCTCAGCCTGATCCACGCTTTAGCGGAGCAAGTGAAGACGTGTACGCATAGCGGACGAGCAGGGAGGTATGCGGTCGAAGCGTACGATGCCCGAGTCCTAGTGCCCTCAGGCTATGCAATTTCGCCTGAAGACTTCCAGAGTCTAAGCGAAAGCGCAACGATGGTGTACAACGAAAGAGAGTTCGTAAACAGAAAGTTACACCACATTGCGATGCACGGACCAGCCCTGAACACTGACGAAGAGTCGTATGAGCTTGTGAGGGCAGAGAGAACAGAACACGAGTACGTCTACGACGTGGACCAGAGAAGATGCTGTAAGAAGGAAGAAGCTGCAGGATTGGTACTGGTGGGCGACTTGACTAATCCGCCCTACCACGAATTTGCATACGAAGGGCTAAAAATTCGCCCCGCTTGTCCATACAAAATTGCAGTCATAGGAGTCTTCGGGGTACCAGGATCTGGTAAGTCAGCCATTATCAAGAACCTAGTTACCAGGCAAGACCTGGTGACTAGCGGAAAGAAAGAAAACTGCCAAGAAATCAGCACCGACGTGATGAGACAGAGAGGTCTAGAAATATCTGCACGTACGGTAGATTCGCTGCTCTTGAATGGATGCAATAGACCAGTCGACGTGTTGTACGTAGACGAGGCGTTTGCGTGCCACTCTGGAACGTTACTTGCCTTGATCGCCTTGGTGAGACCAAGACTGAAAGTTGTACTTTGTGGTGACCCGAAGCAGTGCGGCTTCTTCAATATGATGCAGATGAAAGTCAACTACAATCATAACATCTGCACTCAAGTGTACCACAAAAGTATCTCCAGGCGGTGTACACTGCCTGTGACTGCCATTGTGTCGTCGTTGCATTACGAAGGCAAAATGCGCACTACGAATGAGTACAACATGCCGATTGTAGTGGACACTACGGGCTCAACGAAACCTGACCCTGGAGACCTCGTGTTAACGTGCTTCAGAGGGTGGGTTAAACAACTGCAAATTGACTATCGTGGACACGAGGTCATGACAGCAGCCGCATCCCAAGGGTTAACCAGAAAAGGAGTTTACGCAGTTAGGCAAAAAGTTAACGAAAACCCACTCTATGCATCAACATCAGAGCACGTCAACGTACTCCTAACGCGTACGGAAGGTAAACTGGTATGGAAGACACTCTCTGGTGACCCGTGGATAAAGACGCTGCAGAACCCACCGAAAGGAAACTTCAAGGCAACTATTAAGGAGTGGGAGGTGGAGCACGCATCGATAATGGCGGGCATCTGCAGTCACCAAGTGACCTTTGACACGTTCCAAAACAAAGCCAACGTTTGCTGGGCTAAGAGCTTGGTCCCTGTCCTCGAAACAGCGGGGATAAAACTAAATGATAGGCAGTGGTCCCAGATAATTCAAGCCTTCAAAGAAGACAAAGCATACTCACCCGAAGTAGCCCTGAATGAAATATGCACGCGCATGTATGGGGTGGATCTAGACAGCGGGCTATTCTCTAAACCGTTGGTATCTGTGTATTACGCGGATAACCACTGGGATAATAGGCCGGGAGGAAAGATGTTCGGATTCAACCCTGAGGCAGCGTCCATTCTAGAAAGAAAGTACCCGTTTACAAAAGGAAAGTGGAACATCAACAAGCAGATCTGCGTGACTACCAGGAGGATAGAAGACTTCAACCCTACCACCAACATTATACCGGCCAACAGGAGACTACCACACTCATTAGTGGCCGAACACCGCCCAGTAAAAGGGGAAAGAATGGAATGGCTGGTTAACAAGATAAACGGACATCATGTGCTCCTGGTTAGCGGCTATAACCTTGCACTGCCTACTAAGAGAGTCACCTGGGTAGCGCCACTAGGTGTCCGCGGAGCGGACTATACATACAACCTAGAGCTGGGTCTACCGGCAACACTTGGTAGGTATGACCTAGTGGTCATAAACATCCACACACCTTTTCGCATACACCATTACCAACAGTGCGTAGATCACGCAATGAAACTGCAAATGCTAGGGGGTGACTCACTGAGACTGCTCAAACCGGGTGGCTCTCTATTGATCAGAGCATACGGTTACGCAGATAGAACCAGTGAACGAGTAATCTGCGTACTGGGACGTAAGTTTAGATCGTCCAGAGCATTGAAACCACCTTGTATCACCAGTAATACTGAGATGTTTTTCCTATTTAGCAGTTTTGACAATGGCAGAAGGAATTTTACAACGCATGTCATGAACAATCAACTGAACGCAGCCTTTGTAGGACAGGCCACCCGAGCAGGATGTGCACCATCGTACCGGGTAAAACGCATGGACATCGCGAAGAACGATGAAGAGTGCGTGGTCAACGCCGCCAACCCTCGCGGGTTACCGGGTGACGGTGTTTGCAAGGCAGTATATAAAAAATGGCCGGAGTCCTTTAAAAATAGTGCAACACCAGTAGGAACTGCAAAAACAGTTATGTGCGGTACATATCCAGTAATCCACGCCGTAGGACCAAACTTCTCAAATTACACGGAGTCCGAAGGGGACCGGGAATTGGCGGCTGCCTATCGAGAAGTCGCAAAGGAAGTAACTAGACTGGGAGTAAATAGCGTAGCTATACCTCTCCTCTCCACAGGTGTATACTCAGGAGGGAAAGACAGGCTAACCCAGTCACTGAACCACCTCTTTACAGCCATGGACTCGACGGATGCAGACGTGGTCATCTACTGCCGAGACAAGGAATGGGAGAAGAAAATATCTGAGGCCATACAGATGCGGACCCAAGTGGAGCTGCTGGATGAGCACATCTCCATAGACTGCGATGTCATTCGCGTGCACCCTGACAGTAGTTTGGCAGGTAGAAAAGGATACAGCACCACGGAAGGCGCACTGTATTCATATCTAGAAGGGACACGTTTTCACCAGACGGCAGTGGATGTGGCAGAGATACATACTATGTGGCCAAAGCAAACAGAGGCCAATGAGCAAGTCTGCCTATATGCCCTGGGGGAAAGTATTGAATCAATCAGGCAGAAATGCCCGGTGGATGATGCAGATGCATCATCTCCCCCGAAAACTGTCCCGTGCCTTTGCCGTTATGCCATGACTCCTGAACGCGTCACCCGACTTCACATGAACCGTGTCACAAATATAATTGTGTGTTCTTCATTTCCCCTTCCAAAGTACAAGATAGAAGGAGTGCAAAAAGTCAAATGCTCCAAGGTAATGTTATTTGATCACAATGTGCCATCGCGCGTAAGTCCAAGGGAATACAGATCTTCCCAGGAGTCTGTACGGGAAGTGAGTATGACAACGTCATTGACGCATAGCCAGTTTGATCTAAGCGCCGATGGCGAGACACTGCCTGTCCCGTCAGACCTGGATGCTGACGCCCCAGCCCTAGAACCGGCCCTAGACGACGGGGCGGTACATACATTACCAACCATAATTGGAAACCTTGCGGCCGTGTCTGACTGGGTAATGAGCACCGTACCTGTCGCGCCGCCTAGAAGAAGGAGAGGGAGAAACCTGACCGTGATATGTGACGAGAGAGAAGGGAATATAACACCCATGGCTAGCGTCCGATTCTTTAGAGCAGAGCAGTGTCCGGCCGTACAAGAAACAGCGGAGACGCGTGACACAGCTATTTCCCTTCGGGCACCGCCAAGTATCACCGTGGAACTGAGCCATCCACCGATCTCCTTCGGAGCACCAAGCGAGACGTTCCCCATCACATTTGGGGACTTCGACGATGGAGAAATCGAAAGCTTGTCTTCTGAGCTACTAACTTTCGGAGACTTCCTACCCGGTGAAGTGGATGATTTGACAGATAGCGACTGGTCCACGTGCTCAGACACGGACGACGAGTTATGACTAGACAGGGCAGGTGGGTATATATTCTCGTCGGACACTGGTCCAGGCCATTTACAACAGAAGTCGGTACGCCAGTCAGTGCTGCCGGTAAACACCCTGGAGGAAGTTCACGAGGAGAAGTGTTACCCACCTAAGCTGGATGAATTAAAGGAGCAACTACTACTTAAGAAACTCCAGGAGAGTGCGTCCATGGCCAATAGAAGCAGGTATCAATCACGCAAAGTGGAAAATATGAAAGCAACAATCATTCAGAGATTAAAGAGAGGCTGTAAACTGTATTTAATGGCAGAGACCCCGAAAGTCCCGACTTATCGGACCGTATACCCGGCGCCTGTGTACTCGCCTCCGATCAACGTCCGATTGTCCAATCCCGAGTCCGCAGTGGCAGCATGTAATGAGTTCTTAGCTAGAAACTACCCAACTGTTTCATCATACCAAATCACCGATGAGTATGATGCATATCTAGACATGGTGGACGGGTCGGAGAGTTGCTTGGACCGAGCGACATTCAATCCGTCAAAACTCAGGAGCTACCCGAAACAACATGCTTATCACGCGCCTTCTATCAGAAGCGCTGTACCTTCCCCATTCCAGAACACACTACAGAATGTACTGGCAGCAGCCACGAAAAGGAACTGCAACGTCACACAGATGAGGGAATTACCCACTTTGGACTCAGCAGTATTCAACGTGGAGTGTTTTAAAAAATTCGCATGCAACCGAGAATACTGGGAAGAATTTGCTGCCAGCCCTATCAGGATAACAACTGAGAATCTAACAACCTATGTCACTAAACTAAAGGGGCCAAAAGCAGCAGCGTTGTTTGCAAGAACCCATAATCTGCTGCCGCTGCAGGATGTACCAATGGATAGGTTCACAGTAGATATGAAAAGGGACGTGAAGGTAACTCCTGGTACAAAGCATACAGAGGAAAGGCCTAAGGTGCAGGTTATACAGGCGGCTGAACCCTTGGCAACAGCGTACCTATGTGGAATTCACAGAGAATTGGTTAGGAGATTGAACGCCGTCCTCCTACCCAATGTGCATACACTATTTGACATGTCTGCCGAGGACTTCGATGCCATTATAGCCGCACACTTTAAGCCAGGAGACGCCGTTTTAGAAACGGACATAGCCTCCTTTGATAAGAGCCAGGATGATTCACTTGCGCTTACCGCCTTAATGCTGTTAGAAGATTTGGGAGTGGATCACTCCCTGTTGGACCTGATAGAGGCTGCTTTTGGAGAGATTTCCAGCTGTCACCTGCCGACAGGTACGCGCTTCAAGTTCGGCGCTATGATGAAATCCGGTATGTTCCTAACTCTGTTCGTCAACACATTGTTAAATATCACCATCGCTAGCCGGGTGTTGGAAGATCGTCTGACAAAATCTGCATGCGCGGCCTTCATCGGCGACGACAACATAATACATGGTGTCGTCTCCGATGAATTGATGGCAGCCAGATGCGCTACTTGGATGAACATGGAAGTGAAGATCATAGATGCAGTTGTATCCCGGAAAGCTCCTTACTTTTGTGGAGGGTTTATACTGCATGATACTGTGACAGGAACAGCTTGCAGAGTGGCGGACCCGCTAAAAAGGTTATTTAAACTGGGCAAACCGTTAGCGGCAGGTGACGAACAAGACGAAGACAGAAGACGGGCGCTGGCCGATGAAGTAATCAGATGGCAACGAACAGGGCTAATAGATGAGCTGGAGAAAGCGGTGTACTCTAGGTACGAAGTGCAGGGTATATCAGTTGCTGTAATGTCTATGGCCACCTTTGCAAGCTCCAGATCCAACTTCGAGAAGCTCAGAGGACCCGTCATAACTTTGTACGGCGGTCCTAAATAGGTACGCACTACAGCTACCTATTTTGCAGAAGCCGACAGCAGGTACCTAAATACCAATCAGCCATAATGGAGTTTATCCCAACCCAAACTTTCTACAATAGGAGGTACCAGCCTCGACCTTGGACTCCGCGCCCTACTATCCAAGTTATCAGACCCAGACCGCGTCCGCAAAGGAAGGCCGGGCAACTTGCCCAGCTGATCTCAGCAGTTAATAAACTGACAATGCGCGCGGTACCTCAACAGAAGCCGCGCAAGAATCGGAAGAATAAGAAGCAAAAGCAAAAGCAGCAGGCGCCACGAAACAATATGAATCAAAAGAAGCAGCCCCCTAAAAAGAAACCGGTTCAAAAGAAAAAGAAGCCGGGCCGCAGAGAGAGAATGTGCATGAAAATCGAAAATGATTGCATCTTCGAAGTCAAGCATGAAGGTAAGGTAACAGGTTACGCGTGCTTGGTAGGGGACAAAGTAATGAAGCCAGCACACGTAAAGGGGACCATCGATAACGCGGACCTGGCCAAATTGGCCTTCAAGCGGTCATCTAAGTACGACCTTGAATGCGCGCAGATACCCGTGCACATGAAGTCCGACGCTTCGAAGTTCACCCATGAGAAACCGGAGGGGTACTACAACTGGCACCACGGAGCAGTACAGTACTCAGGAGGCCGATTCACCATCCCTACAGGTGCGGGCAAACCAGGGGATAGTGGTAGACCGATCTTTGACAACAAGGGGCGCGTGGTGGCCATAGTTTTAGGAGGAGCTAATGAAGGAGCCCGTACAGCCCTCTCGGTGGTGACCTGGAACAAAGACATCGTCACGAAAATCACCCCTGAGGGGGCCGAAGAGTGGAGTCTGGCCATTCCAGTTATGTGCCTGCTGGCAAATACCACGTTCCCCTGCTCCCAGCCCCCTTGCACACCCTGCTGCTACGAAAAAGAGCCGGAGAAAACCTTGCGCATGCTTGAAGACAATGTCATGAGCCCCGGGTACTATCAGCTGCTACAAGCATCCTTAACATGTTCTCCCCGACGCCGGCGGCGCAGTATTAAGGACCACTTCAATGTCTATAAAGCCACAAGACCGTACCTAGCTCACTGTCCCGACTGTGGAGAAGGGCACTCGTGCCATAGTCCCGTAGCGCTAGAACGCATCAGAAACGAAGCGACAGACGGGACGTTGAAAATCCAGGTTTCCTTGCAAATCGGAATAAAGACGGATGATAGCCATGATTGGACCAAGCTGCGTTATATGGACAATCACATGCCAGCAGACGCAGAGCGGGCCGGGCTATTTGTAAGAACGTCAGCACCGTGCACGATTACTGGAACAATGGGACACTTCATTCTGGCCCGATGTCCGAAAGGAGAAACTCTGACGGTGGGGTTCACTGATGGTAGAAAGATCAGTCACTCATGTACGCACCCATTTCACCACGACCCTCCTGTGATAGGCCGGGAAAAATTCCATTCCCGACCGCAGCACGGTAGGGAACTACCTTGCAGCACGTACGCGCAGAGCACCGCTGCAACTGCCGAGGAGATAGAGGTACATATGCCCCCAGACACCCCAGATCGCACATTAATGTCACAACAGTCCGGCAATGTAAAGATCACAGTCAATAGTCAGACGGTGCGGTACAAGTGCAATTGTGGTGACTCAAGTGAAGGATTAACCACTACAGATAAAGTGATTAATAACTGCAAGGTTGATCAATGCCATGCCGCGGTCACCAATCACAAAAAATGGCAGTATAATTCCCCTCTGGTCCCGCGCAATGCTGAATCCGGGGACCGGAAAGGAAAAGTTCACATTCCATTTCCTCTGGCAAATGTGACATGCAGGGTGCCTAAAGCAAGAAACCCCACCGTGACGTACGGAAAAAACCAAGTCATCATGTTGCTGTATCCTGACCACCCAACGCTCCTGTCCTACAGGAATATGGGAGAAGAACCAAACTATCAAGAAGAGTGGGTGACGCATAAGAAGGAGATCAGGTTAACCGTGCCGACTGAAGGGCTCGAGGTCACGTGGGGTAACAATGAGCCGTACAAGTATTGGCCGCAGTTATCCACAAACGGTACAGCCCACGGCCACCCGCATGAGATAATTCTGTATTATTATGAGCTGTACCCAACTATGACTGTGGTAGTTGTGTCAGTGGCCTCGTTCATACTCCTGTCGATGGTGGGTGTGGCAGTGGGGATGTGCATGTGTGCACGACGCAGATGCATTACACCGTACGAACTGACACCAGGAGCTACCGTCCCTTTCCTGCTTAGCCTAATATGCTGCATTAGAACAGCTAAAGCGGCCACATACCAGGAGGCCGCGGTATACCTGTGGAACGAGCAGCAGCCTTTATTTTGGCTGCAAGCCCTTATTCCGCTGGCAGCCCTGATTGTCCTATGTAACTGTCTGAGACTCTTACCATGCTGTTGTAAAATGTTGACTTTTTTAGCCGTACTGAGCGTCGGTGCCCACACTGTGAGCGCGTACGAACACGTAACAGTGATCCCGAACACGGTGGGAGTACCGTATAAGACTCTAGTCAACAGACCGGGCTACAGCCCCATGGTACTGGAGATGGAGCTTCTGTCTGTCACTTTGGAGCCAACGCTATCGCTTGATTACATCACGTGCGAGTATAAAACCGTTATCCCGTCTCCGTACGTGAAATGCTGCGGTACAGCAGAGTGTAAGGACAAGAGCCTACCTGATTACAGCTGTAAGGTCTTCACCGGCGTCTACCCATTCATGTGGGGCGGCGCCTACTGCTTCTGCGACACCGAAAATACGCAATTGAGCGAAGCACATGTGGAGAAGTCCGAATCATGCAAAACAGAATTTGCATCAGCATACAGGGCTCATACCGCATCCGCATCAGCTAAGCTCCGCGTCCTTTACCAAGGAAATAATATCACTGTAGCTGCTTATGCAAACGGCGACCATGCCGTCACAGTTAAGGACGCCAAATTCATAGTGGGGCCAATGTCTTCAGCCTGGACACCTTTCGACAATAAAATCGTGGTGTACAAAGGCGACGTCTACAACATGGACTACCCGCCCTTCGGCGCAGGAAGACCAGGACAATTTGGCGACATCCAAAGTCGCACGCCTGAAAGCGAAGACTTCTATGCTAATACACAACTGGTACTGCAGAGACCGTCCGCGGGTACGGTGCACGTGCCGTACTCTCAGGCACCATCTGGCTTCAAGTATTGGCTAAAAGAACGAGGGGCGTCGCTACAGCACACAGCACCATTTGGCTGTCAAATAGCAACAAACCCGGTAAGAGCGATGAACTGCGCCGTAGGGAACATGCCTATCTCCATCGACATACCGGACGCGGCCTTTACCAGGGTCGTCGACGCGCCATCTTTAACGGACATGTCGTGTGAGGTATCAGCCTGCACCCATTCCTCAGACTTTGGGGGCGTAGCCATCATTAAATATGCAGCCAGTAAGAAAGGCAAGTGTGCAGTGCACTCGATGACTAACGCCGTCACTATTTGGGAAGCTGAAATAGAAGTGGAAGGGAACTCTCAGTTGCAAATCTCTTTTTTGACGGCCCTAGCCAGCGCCGAATTCCGCGTACAAGTCTGTTCTACACAAGTACACTGTGCAGCCGAGTGCCATCCACCGAAAGACCATATAGTCAATTACCCGGCGTCACACACCACCCCCGGGGTCCAAGACATTTCCGCTACGGCGATGTCATGGGTGCAGAAGATCACGGGAGGTGTGGGACTGGTTGTCGCTGTTGCAGCACTGATCCTAATCGTGGTGCTATGCGTGTCGTTTAGCAGGCACTAACTTGACAACTAGGTATGAAGGCATACGCGTCCCTAAAGAGACACACCACATATAGCTAAGAATCAATAGATAAGTATAGATCAAAGGGCTGAACAATCCCTGAATAGTAACAAAATATAAAAATCAACAAAAATCATAAAATAGAAAACTAGAAATAGAAGTAGGTAAGAAGGTATATGTGTCCCCTAAGAGACACACCATATATAGCTAAGAATCAATAGATAAGCATAGATCAAAGGGCTGAATAACCCCTGAATAATAACAAAATATAAAAACCAATAAAAATCATAAAATAGAAAACCATAAACAGAAGTAGTTCAAAGGGCTATGAAACCCCTGAATAGTAACAAAATATAAAACTAATAAAAATCAAACGAATACCATAATTGGCAATCGGAAGAGATGTAGGTACTTAAGCTTCCTAAAAGCAGCCGAACTCGCTTTGAGATGTAGGCGTAGCACACCGAACTCTTCCACGATTCTCCGAACCCACAGGGACGTAGGAGAAGTTCAAAGTGACTATAAAAACCCTGAACAGTAATAAAACATAAAATTAATAATGAGTACCATAATTGGCAAACGGAAGAGACGTAGGTACTAAGCTTCCTAAAAGCAGCCGAACTCACTTTGAGATGTAGGCATAGCATACCGAACTCTTCCACAATTCTCCGTACCCATAGGGACGTAGGAGATGTTATTTTGTTTTTAATATTTC

>EU703762|2006|Malaysia: Bagan Panchor, Group 1

ATGGCTGCGTGAGACACACGTAGCCTACCAGTTTCTTACTGCTCTACTCTGCAAAGCAAGAGATCAATAACCCATCATGGATTCTGTGTACGTGGATATAGACGCTGACAGCGCCTTTTTGAAGGCCCTGCAACGTGCGTACCCCATGTTTGAGGTGGAACCTAGGCAGGTCACATCAAATGACCATGCTAATGCTAGAGCGTTCTCGCATCTAGCCATAAAACTAATAGAGCAGGAAATTGATCCCGACTCAACCATCCTGGATATAGGTAGTGCGCCAGCAAGGAGGATGATGTCGGACAGGAAGTACCACTGCGTTTGCCCGATGCGCAGCGCAGAAGATCCCGAGAGACTCGCTAATTATGCGAGAAAGCTCGCATCTGCCGCAGGAAAAGTCCTGGACAGAAACATTTCTGGAAAGATCGGGGACTTACAAGCGGTGATGGCCGTGCCAGACACGGAGACGCCAACATTTTGCTTACACACAGATGTCTCATGTAGACAGAGAGCAGACGTCGCGATATACCAAGACGTCTATGCTGTACATGCACCCACGTCGCTATATCACCAGGCGATTAAAGGAGTCCGAGTGGCGTACTGGGTAGGGTTTGACACAACCCCGTTCATGTACAACGCTATGGCGGGTGCCTACCCCTCATACTCGACAAATTGGGCGGATGAGCAGGTACTGAAGGCTAAGAACATAGGATTATGTTCAACAGACCTGACGGAAGGTAGACGAGGCAAATTGTCTATCATGAGAGGGAAAAAGCTAAAACCGTGCGACCGTGTGCTGTTCTCAGTAGGGTCAACGCTTTACCCGGAAAGCCGCATGCTACTTAAGAGCTGGCACCTACCATCGGTGTTCCATCTAAAGGGCAAGCTTAGCTTCACATGCCGCTGTGACACAGTGGTTTCGTGTGAGGGCTACGTCGTCAAGAGAATAACGATGAGCCCAGGCCTTTATGGAAAAACCACGGGGTATGCGGTAACCCACCACGCAGACGGATTCTTGATGTGCAAGACTACCGATACGGTTGACGGCGAAAGAGTGTCATTCTCGGTGTGCACGTACGTGCCGGCGACCATTTGTGATCAAATGACCGGCATCCTTGCTACAGAAGTCACGCCGGAGGATGCACAGAAGCTGTTGGTGGGGCTGAACCAGAGAATAGTGGTTAACGGCAGAACGCAACGGAACACGAACACCATGAAGAACTATCTACTTCCCGTGGTCGCCCAGGCCTTCAGTAAGTGGGCGAAGGAGTGCCGGAAGGACATGGAAGATGAGAAGCTTCTGGGGGTCAGAGAAAGAACACTAACCTGCTGCTGTCTGTGGGCATTTAAGAAGCAGAAAACACACACGGTCTACAAGAGGCCTGATACCCAGTCAATCCAGAAGGTTCAGGCCGAATTTGACAGCTTTGTAGTACCAGGCCTGTGGTCGTCCGGGTTGTCAATCCCGTTGAGGACTAGAATCAAGTGGCTGTTACGCAAGGTGCCGAAAGCAGACCTGATCCCATACAGCGGAAATGCCCAAGAAGCCCAGGATGCAGAAAAAGAAGCAGAGGAAGAACGAGAAGCAGAACTGACTCATGAGGCTCTACCACCCCTACAGGCAGCACAGGAAGATGTCCAGGTCGAAATCGACGTGGAACAACTTGAGGATAGAGCTGGTGCTGGAATAATAGAGACTCCGAGAGGCGCTATTAAAGTTACTGCCCAACTAACCGACCACGTCGTGGGGGAGTACCTGGTACTTTCCCCGCAGACCGTATTACGCAGCCAGAAGCTCAGCCTGATCCACGCTTTAGCGGAGCAAGTGAAGACGTGTACGCATAGCGGACGAGCAGGGAGGTATGCGGTCGAAGCGTACGATGGCCGAGTTCTAGTGCCCTCAGGCTATGCAATTTCGCCTGAAGACTTCCAGAGTCTAAGCGAAAGCGCAACGATGGTGTACAACGAAAGAGAGTTCGTAAACAGAAAGTTACACCACATTGCGATGCACGGACCAGCCCTGAACACTGACGAAGAGTCGTATGAGCTTGTGAGGGCAGAGAGAACAGAACACGAGTACGTCTACGACGTGGACCAGAGAAGATGCTGTAAGAAGGAAGAAGCTGCAGGATTGGTACTGGTGGGCGACTTGACTAATCCGCCCTACCACGAATTTGCATACGAAGGGCTAAAAATTCGCCCCGCTTGTCCATACAAAATTGCAGTCATAGGAGTCTTCGGGGTACCAGGATCTGGTAAGTCAGCCATTATCAAGAACCTAGTTACCAGGCAAGACCTGGTGACTAGCGGAAAGAAAGAAAACTGCCAAGAAATCAGCACCGACGTGATGAGACAGAGAGGTCTAGAAATATCTGCACGTACGGTTGATTCGCTGCTCTTGAATGGATGCAATAGACCAGTCGACGTGTTGTACGTAGACGAGGCGTTTGCGTGCCACTCTGGAACGTTACTTGCCTTGATCGCCTTGGTGAGACCAAGACTGAAAGTTGTACTTTGTGGTGACCCGAAGCAGTGCGGCTTCTTCAATATGATGCAGATGAAAGTCAACTACAATCATAACATCTGCACTCAAGTGTACCACAAAAGTATCTCCAGGCGGTGTACACTGCCTGTGACTGCCATTGTGTCGTCGTTGCATTACGAAGGCAAAATGCGCACTACGAATGAGTACAACATGCCGATTGTAGTGGACACTACGGGCTCAACGAAACCTGACCCTGGAGACCTCGTGTTAACGTGCTTCAGAGGGTGGGTTAAACAACTGCAAATTGACTATCGTGGACACGAGGTCATGACAGCAGCCGCATCCCAAGGGTTAACCAGAAAAGGAGTTTACGCAGTTAGGCAAAAAGTTAACGAAAACCCACTCTATGCATCAACATCAGAGCACGTCAACGTACTCCTAACGCGTACGGAAGGTAAACTGGTATGGAAGACACTCTCTGGTGACCCGTGGATAAAGACGCTGCAGAACCCACCGAAAGGAAACTTCAAGGCAACTATTAAGGAGTGGGAGGTGGAGCACGCATCGATAATGGCGGGCATCTGCAGTCACCAAGTGACCTTTGACACGTTCCAAAACAAAGCCAACGTTTGCTGGGCTAAGAGCTTGGTCCCTGTCCTCGAAACAGCGGGGATAAAACTAAATGATAGGCAGTGGTCCCAGATAATTCAAGCCTTCAAAGAAGACAAAGCATACTCACCCGAAGTAGCCCTGAATGAAATATGCACGCGCATGTATGGGGTGGATCTAGACAGCGGGCTATTCTCTAAACCGTTGGTATCTGTGTATTACGCGGATAACCACTGGGATAATAGGCCGGGAGGAAAGATGTTCGGATTCAACCCTGAGGCAGCGTCCATTCTAGAAAGAAAGTACCCGTTTACAAAAGGAAAGTGGAACATCAACAAGCAGATCTGCGTGACTACCAGGAGGATAGAAGACTTCAACCCTACCACCAACATTATACCGGCCAACAGGAGACTACCACACTCATTAGTGGCCGAACACCGCCCAGTAAAAGGGGAAAGAATGGAATGGCTGGTTAACAAGATAAACGGACATCATGTGCTCCTGGTTAGCGGCTATAACCTTGCACTGCCTACTAAGAGAGTCACCTGGGTAGCGCCACTAGGTGTCCGCGGAGCGGACTATACATACAACCTAGAGCTGGGTCTACCGGCAACACTTGGTAGGTATGACCTAGTGGTCATAAACATCCACACACCTTTTCGCATACACCATTACCAACAGTGCGTAGATCACGCAATGAAACTGCAAATGCTAGGGGGTGACTTACTGTGACTGCTCAAACCGGGTGGCTCTCTATTGATCAGAGCATACGGTTACGCAGATAGAACCAGTGAACGAGTAATCTGCGTACTGGGACGTAAGTTTAGATCGTCCAGAGCATTGAAACCACCTTGTATCACCAGTAATACTGAGATGTTTTTCCTATTTAGCAGTTTTGACAATGGCAGAAGGAATTTTACAACGCATGTCATGAACAATCAACTGAACGCAGCCTTTGTAGGACAGGCCACCCGAGCAGGATGTGCACCATCGTACCGGGTAAAACGCATGGACATCGCGAAGAACGATGAAGAGTGCGTGGTCAACGCCGCCAACCCTCGCGGGTTACCGGGTGACGGTGTTTGCAAGGCAGTATATAAAAAATGGCCGGAGTCCTTTAAAAATAGTGCAACACCAGTAGGAACTGCAAAAACAGTTATGTGCGGTACATATCCAGTAATCCACGCCGTAGGACCAAACTTCTCAAATTACACGGAGTCCGAAGGGGACCGGGAATTGGCGGCTGCCTATCGAGAAGTCGCAAAGGAAGTAACTAGACTGGGAGTAAATAGCGTAGCTATACCTCTCCTCTCCACAGGTGTATACTCAGGAGGGAAAGACAGGCTAACCCAGTCACTGAACCACCTCTTTACAGCCATGGACTCGACGGATGCAGACGTGGTCATCTACTGCCGAGACAAGGAATGGGAGAAGAAAATATTTGAGGCCATACAGATGCGGACCCAAGTGGAGCTGCTGGATGAGCACATCTCCATAGACTGCGATGTCATTCGCGTGCACCCTGACAGTAGTTTGGCAGGTAGAAAAGGATACAGCACCACGGAAGGCGCACTGTATTCATATCTAGAAGGGACACGTTTTCACCAGACGGCAGTGGATGTGGCAGAGATACATACTATGTGGCCAAAGCAAACAGAGGCCAATGAGCAAGTCTGCCTATATGCCCTGGGGGAAAGTATTGAATCAATCAGGCAGAAATGCCCGGTGGATGATGCAGATGCATCATCTCCCCCGAAAACTGTCCCGTGCCTTTGCCGTTATGCCATGACTCCTGAACGCGTCACCCGACTTCGCATGAATCATGTCACAAATATAATTGTGTGTTCTTCATTTCCCCTTCCAAAGTACAAGATAGAAGGAGTGCAAAAAGTCAAATGCTCCAAGGTAATGTTATTTGATCACAATGTGCCATCGCGCGTAAGTCCAAGGGAATACAGATCTTCCCAGGAGTCTGTACGGGAAGTGAGTATGACAACGTCATTGACGCATAGCCAGTTTGATCTAAGCGCCGATGGCGAGACACTGCCTGTCCCGTCAGACCTGGATGCTGACGCCCCAGCCCTAGAACCGGCCCTAGACGACGGGGCGGTACATACATTACCAACCATAATCGGAAACCTTGCGGCCGTGTCTGACTGGGTAATGAGCACCGTACCTGTCGCGCCGCCTAGAAGAAGGAGAGGGAGAAACCTGACCGTGATATGTGACGAGAGAGAAGGGAATATAACACCCATGGCTAGCGTCCGATTCTTTAGAGCAGAGCAGTGTCCGGCCGTACAAGAAACAGCGGAGACGCGTGACACAGCTATTTCCCTTCGGGCACCGCCAAGTATCACCGTGGAACTGAGCCATCCACCGATCTCCTTCGGAGCACCAAGCGAGACGTTCCCCATCACATTTGGGGACTTCGACGATGGAGAAATCGAAAGCTTGTCTTTTGAGCTACTAACTTTCGGAGACTTCCTACCCGGTGAAGTGGATGATTTGACAGATAGCGACTGGTCCACGTGCTCAGACACGGACGACGAGTTATGACTAGACAGGGCAGGTGGGTATATATTCTCGTCGGACACTGGTCCAGGCCATTTACAACAGAAGTCGGTACGCCAGTCAGTGCTGCCGGTAAACACCCTGGAGGAAGTTCACGAGGAGAAGTGTTACCCACCTGAGCTGGATGAATTAAAGGAGCAACTACTACTTAAGAAACTCCAGGAGAGTGCGTCCATGGCCAATAGAAGCAGGTATCAATCACGCAAAGTGGAAAATATGAAAGCAACAATCATTCAGAGATTAAAGAGAGGCTGTAAACTGTATTTAATGGCAGAGACCCCGAAAGTCCCGACTTATCGGACCGTATACCCGGCGCCTGTGTACTCGCCTCCGATCAACGTCCGATTGTCCAATCCCGAGTCCGCAGTGGCAGCATGTAATGAGTTCTTAGCTAGAAACTACCCAACTGTTTCATCATACCAAATCACCGATGAGTATGATGCATATCTAGACATGGTGGACGGGTCGGAGAGTTGCTTGGACCGAGCGACATTCAATCCGTCAAAACTCAGGAGCTACCCGAAACAACATGCTTATCACGCGCCTTCTATCAGAAGCGCTGTACCTTCCCCATTCCAGAACACACTACAGAATGTACTGGCAGCAGCCACGAAAAGGAACTGCAACGTCACACAGATGAGGGAATTACCCACTTTGGACTCAGCAGTATTCAACGTGGAGTGTTTTAAAAAATTCGCATGCAACCGAGAATACTGGGAAGAATTTGCTGCCAGCCCTATCAGGATAACAACTGAGAATCTAACAACCTATGTCACTAAACTAAAGGGGCCAAAAGCAGCAGCGTTGTTTGCAAGAACCCATAATCTGCTGCCGCTGCAGGATGTACCAATGGATAGGTTCACAGTAGATATGAAAAGGGACGTGAAGGTAACTCCTGGTACAAAGCATACAGAGGAAAGGCCTAAGGTGCAGGTTATACAGGCGGCTGAACCCTTGGCAACAGCGTACCTATGTGGAATTCACAGAGAATTGGTTAGGAGATTGAACGCCGTCCTCCTACCCAATGTGCATACACTATTTGACATGTCTGCCGAGGACTTCGATGCCATTATAGCCGCACACTTTAAGCCAGGAGACGCCGTTTTAGAAACGGACATAGCCTCCTTTGATAAGAGCCAGGATGATTCACTTGCGCTTACCGCCTTAATGCTGTTAGAAGATTTGGGAGTGGATCACTCCCTGTTGGACCTGATAGAGGCTGCTTTTGGAGAGATTTCCAGCTGTCACCTGCCGACAGGTACGCGCTTCAAGTTCGGCGCTATGATGAAATCCGGTATGTTCCTAACTCTGTTCGTCAACACATTGTTAAATATCACCATCGCTAGCCGGGTGTTGGAAGATCGTCTGACAAAATCTGCATGCGCGGCCTTCATCGGCGACGACAACATAATACATGGTGTCGTCTCCGATGAAGTGATGGCAGCCAGATGCGCTACTTGGATGAACATGGAAGTGAAGATCATAGATGCAGTTGTATCCCAGAAAGCTCCTTACTTTTGTGGAGGGTTTATACTGCATGATACTGTGACAGGAACAGCTTGCAGAGTGGCGGACCCGCTAAAAAGGTTATTTAAACTGGGCAAACCGTTAGCGGCAGGTGACGAACAAGACGAAGACAGAAGACGGGCGCTGGCCGATGAAGTAATCAGATGGCAACGAACAGGGCTAATAGATGAGCTGGAGAAAGCGGTGTACTCTAGGTACGAAGTGCAGGGTATATCAGTTGCTGTAATGTCTATGGCCACCTTTGCAAGCTCCAGATCCAACTTCGAGAAGCTCAGAGGACCCGTCATAACTTTGTACGGCGGTCCTAAATAGGTACGCACTACAGCTACCTATTTTGCAGAAGCCGACAGCAGGTACCTAAATACCAATCAGCCATAATGGAGTTTATCCCAACCCAAACTTTCTACAATAGGAGGTACCAGCCTCGACCTTGGACTCCGCGCCCTACTATCCAAGTTATCAGACCCAGACCGCGTCCGCAAAGGAAGGCCGGGCAACTTGCCCAGCTGATCTCAGCAGTTAATAAACTGACAATGCGCGCGGTACCTCAACAGAAGCCGCGCAAGAATCGGAAGAATAAGAAGCAAAAGCAAAAGCAGCAGGCGCCACGAAACAATATGAATCAAAAGAAGCAGCCCCCTAAAAAGAAACCGGTTCAAAAGAAAAAGAAGCCGGGCCGCAGAGAGAGAATGTGCATGAAAATCGAAAATGATTGCATCTTCGAAGTCAAGCATGAAGGTAAGGTAACAGGTTACGCGTGCTTGGTAGGGGACAAAGTAATGAAGCCAGCACACGTAAAGGGGACCATCGATAACGCGGACCTGGCCAAATTGGCCTTCAAGCGGTCATCTAAGTACGACCTTGAATGCGCGCAGATACCCGTGCACATGAAGTCCGACGCTTCGAAGTTCACCCATGAGAAACCGGAGGGGTACTACAACTGGCACCACGGAGCAGTACAGTACTCAGGAGGCCGATTCACCATCCCTACAGGTGCGGGCAAACCAGGGGATAGTGGTAGACCGATCTTCGACAACAAGGGGCGCGTGGTGGCCATAGTTTTAGGAGGAGCTAATGAAGGAGCCCGTACAGCCCTCTCGGTGGTGACCTGGAACAAAGACATCGTCACGAAAATCACCCCTGAGGGGGCCGAAGAGTGGAGTCTGGCCATTCCAGTTATGTGCCTGCTGGCAAATACCACGTTCCCCTGCTCCCAGCCCCCTTGCACACCCTGCTGCTACGAAAAAGAGCCGGAGAAAACCTTGCGCATGCTTGAAGACAATGTCATGAGCCCCGGGTACTATCAGCTGCTACAAGCATCCTTAACATGTTCTCCCCGACGCCGGCGGCGCAGTATTAAGGACCACTTCAATGTCTATAAAGCCACAAGACCGTACCTAGCTCACTGTCCCGACTGTGGAGAAGGGCACTCGTGCCATAGTCCCGTAGCGCTAGAACGCATCAGAAACGAAGCGACAGACGGGACGTTGAAAATCCAGGTTTCCTTGCAAATCGGAATAAAGACGGATGATAGCCATGATTGGACCAAGCTGCGTTACATGGACAATCACATGCCAGCAGACGCAGAGAGGGCCGGGCTATTTGTAAGAACGTCAGCACCGTGCACGATTACTGGAACAATGGGACACTTCATTCTGGCCCGATGTCCGAAAGGAGAAACTCTGACGGTGGGGTTCACTGATGGTAGAAAGATCAGTCACTCATGTACGCACCCATTTCACCACGACCCTCCTGTGATAGGCCGGGAAAAATTCCATTCCCGACCGCAGCACGGTAGGGAACTACCTTGCAGCACGTACGCGCAGAGCACCGCTGCAACTGCCGAGGAGATAGAGGTACATATGCCCCCAGACACCCCAGATCGCACATTAATGTCACAACAGTCCGGCAATGTAAAGATCACAGTCAATAGTCAGACGGTGCGGTACAAGTGCAATTGTGGTGACTCAAGTGAAGGATTAACCACTACAGATAAAGTGATTAATAACTGCAAGGTTGATCAATGCCATGCCGCGGTCACCAATCACAAAAAATGGCAGTATAATTCCCCTCTGGTCCCGCGCAATGCTGAATCCGGGGACCGGAAAGGAAAAGTTCACATTCCATTTCCTCTGGCAAATGTGACATGCAGGGTGCCTAAAGCAAGAAACCCCACCGTGACGTACGGAAAAAACCAAGTCATCATGTTGCTGTATCCTGACCACCCAACGCTCCTGTCCTACAGGAATATGGGAGAAGAACCAAACTATCAAGAAGAGTGGGTGACGCATAAGAAGGAGATCAGGTTAACCGTGCCGACTGAAGGGCTCGAGGTCACGTGGGGTAACAATGAGCCGTACAAGTATTGGCCGCAGTTATCCACAAACGGTACAGCCCACGGCCACCCGCATGAGATAATTCTGTATTATTATGAGCTGTACCCAACTATGACTGTGGTAGTTGTGTCAGTGGCCTCGTTCATACTCCTGTCGATGGTGGGTGTGGCAGTGGGGATGTGCATGTGTGCACGACGCAGATGCATTACACCGTACGAACTGACACCAGGAGCTACCGTCCCTTTCCTGCTTAGCCTAATATGCTGCATTAGAACAGCTAAAGCGGCCACATACCAGGAGGCCGCGGTATACCTGTGGAACGAGCAGCAGCCTTTATTTTGGCTGCAAGCCCTTATTCCGCTGGCAGCCCTGATTGTCCTATGTAACTGTCTGAGACTCTTACCATGCTGTTGTAAAATGTTGACTTTTTTAGCCGTACTGAGCGTCGGTGCCCACACTGTGAGCGCGTACGAACACGTAACAGTGATCCCGAACACGGTGGGAGTACCGTATAAGACTCTAGTCAACAGACCGGGCTACAGCCCCATGGTACTGGAGATGGAGCTTCTGTCTGTCACTTTGGAGCCAACGCTATCGCTTGATTACATCACGTGCGAGTATAAAACCGTTATCCCGTCTCCGTACGTGAAATGCTGCGGTACAGCAGAGTGTAAGGACAAGAGCCTACCTGATTACAGCTGTAAGGTCTTCACCGGCGTCTACCCATTCATGTGGGGCGGCGCCTACTGCTTCTGCGACACCGAAAATACGCAATTGAGCGAAGCACATGTGGAGAAGTCCGAATCATGCAAAACAGAATTTGCATCAGCATACAGGGCTCATACCGCATCCGCATCAGCTAAGCTCCGCGTCCTTTACCAAGGAAATAATATCACTGTAGCTGCTTATGCAAACGGCGACCATGCCGTCACAGTTAAGGACGCCAAATTCATAGTGGGGCCAATGTCTTCAGCCTGGACACCTTTCGACAATAAAATCGTGGTGTACAAAGGCGACGTCTACAACATGGACTACCCGCCCTTCGGCGCAGGAAAACCAGGACAATTTGGCGACCTCCAAAGTCGCACGCCTGAGAGCGAAGACGTCTATGCTAATACACAACTGGTACTGCAGAGACCGTCCGCGGGTACGGTGCACGTGCCGTACTCTCAGGCACCATCTGGCTTCAAGTATTGGCTAAAAGAACGAGGGGCGTCGCTACAGCACACAGCACCATTTGGCTGTCAAATAGCAACAAACCCGGTAAGAGCGATGAACTGCGCCGTAGGGAACATGCCTATCTCCATCGACATACCGGACGCGGCCTTTACCAGGGTCGTCGACGCGCCATCTTTAACGGACATGTCGTGTGAGGTATCAGCCTGCACCCATTCCTCAGACTTTGGGGGCGTAGCCATCATTAAATATGCAGCCAGTAAGAAAGGCAAGTGTGCAGTGCACTCGATGACTAACGCCGTCACTATTCGGGAAGCTGAAATAGAAGTGGAAGGGAACTCTCAGTTGCAAATCTCTTTTTCGACGGCCCTAGCCAGCGCCGAATTCCGCGTACAAGTCTGTTCTACACAAGTACACTGTGCAGCCGAGTGCCATCCACCGAAAGACCATATAGTCAATTACCCGGCGTCACACACCACCCTCGGGGTCCAAGACATTTCCGCTACGGCGATGTCATGGGTGCAGAAGATCACGGGAGGTGTGGGACTGGTTGTCGCTGTTGCAGCACTGATCCTAATCGTGGTGCTATGCGTGTCGTTTAGCAGGCACTAACTTGACAACTAGGTATGAAGGCATACGCGTCCCTAAAGAGACACACCACATATAGCTAAGAATCAATAGATAAGTATAGATCAAAGGGCTGAACAATCCCTGAATAGTAACAAAATATAAAAATCAACAAAAATCATAAAATAGAAAACTAGAAATAGAAGTAGGTAAGAAGGTATATGTGTCCCCTAAGAGACACACCATATATAGCTAAGAATCAATAGATAAGCATAGATCAAAGGGCTGAATAACCCCTGAATAATAACAAAATATAAAAACCAATAAAAATCATAAAATAGAAAACCATAAACAGAAGTAGTTCAAAGGGCTATGAAACCCCTGAATAGTAACAAAATATAAAACTAATAAAAATCAAACGAATACCATAATTGGCAATCGGAAGAGATGTAGGTACTTAAGCTTCCTAAAAGCAGCCGAACTCGCTTTGAGATGTAGGCGTAGCACACCGAACTCTTCCACGATTCTCCGAACCCACAGGGACGTAGGAGATGTTCAAAGTGACTATAAAAACCCTGAACAGTAATAAAACATAAAATTAATAATGAGTACCATAATTGGCAAACGGAAGAGACGTAGGTACTAAGCTTCCTAAAAGCAGCCGAACTCACTTTGAGATGTAGGCATAGCATACCGAACTCTTCCACAATTCTCCGTACCCATAGGGACGTAGGAGATGTTATTTTGTTTTTAATATTTC

>FN295483|2006|Malaysia:Perak, Group 1

CAAAGCAAGAGATCAATAACCCATCATGGATTCTGTGTACGTGGATATAGACGCTGACAGCGCCTTTTTGAAGGCCCTGCAACGTGCGTACCCCATGTTTGAGGTGGAACCTAGGCAGGTCACATCAAATGACCATGCTAATGCTAGAGCGTTCTCGCATCTAGCCATAAAACTAATAGAGCAGGAAATTGATCCCGACTCAACCATCCTGGATATAGGTAGTGCGCCAGCAAGGAGGATGATGTCGGACAGGAAGTACCACTGCGTTTGCCCGATGCGCAGCGCAGAAGATCCCGAGAGACTCGCTAATTATGCGAGAAAGCTCGCATCTGCCGCAGGAAAAGTCCTGGACAGAAACATTTCTGGAAAGATCGGGGACTTACAAGCGGTGATGGCCGTGCCAGACACGGAGACGCCAACATTTTGCTTACACACAGATGTCTCATGTAGACAGAGAGCAGACGTCGCGATATACCAAGACGTCTATGCTGTACATGCACCCACGTCGCTATATCACCAGGCGATTAAAGGAGTCCGAGTGGCGTACTGGGTAGGGTTTGACACAACCCCGTTCATGTACAACGCTATGGCGGGTGCCTACCCCTCATACTCGACAAATTGGGCGGATGAGCAGGTACTGAAGGCTAAGAACATAGGATTATGTTCAACAGACCTGACGGAAGGTAGACGAGGCAAATTGTCTATCATGAGAGGGAAAAAGCTAAAACCGTGCGACCGTGTGCTGTTCTCAGTAGGGTCAACGCTTTACCCGGAAAGCCGCATGCTACTTAAGAGCTGGCACCTACCATCGGTGTTCCATCTAAAGGGCAAGCTTAGCTTCACATGCCGCTGTGACACAGTGGTTTCGTGTGAGGGCTACGTCGTCAAGAGAATAACGATGAGCCCAGGCCTTTATGGAAAAACCACGGGGTATGCGGTAACCCACCACGCAGACGGATTCTTGATGTGCAAGACTACCGACACGGTTGACGGCGAAAGAGTGTCATTCTCGGTGTGCACGTACGTGCCGGCGACCATTTGTGATCAAATGACCGGCATCCTTGCTACAGAAGTCACGCCGGAGGATGCACAGAAGCTGTTGGTGGGGCTGAACCAGAGAATAGTGGTTAACGGCAGAACGCAACGGAACACGAACACCATGAAGAACTATCTGCTTCCCGTGGTCGCCCAGGCCTTCAGTAAGTGGGCGAAGGAGTGCCGGAAGGACATGGAAGATGAGAAGCTTCTGGGGGTCAGAGAAAGAACACTAACCTGCTGCTGTCTGTGGGCATTTAAGAAGCAGAAAACACACACGGTCTACAAGAGGCCTGATACCCAGTCAATCCAGAAGGTTCAGGCCGAATTTGACAGCTTTGTAGTACCAGGCCTGTGGTCGTCCGGGTTGTCAATCCCGTTGAGGACTAGAATCAAGTGGCTGTTACGCAAGGTGCCGAAAGCAGACCTGATCCCATACAGCGGAAATGCCCAAGAAGCCCAGGATGCAGAAAAAGAAGCAGAGGAAGAACGAGAAGCAGAACTGACTCATGAGGCTCTACCACCCCTACAGGCAGCACAGGAAGATGTCCAGGTCGAAATCGACGTGGAACAACTTGAGGATAGAGCTGGTGCTGGAATAATAGAGACTCCGAGAGGCGCTATTAAAGTTACTGCCCAACTAACCGACCACGTCGTGGGGGAGTACCTGGTACTTTCCCCGCAGACCGTATTACGCAGCCAGAAGCTCAGCCTGATCCACGCTTTAGCGGAGCAAGTGAAGACGTGTACGCATAGCGGACGAGCAGGGAGGTATGCGGTCGAAGCGTACGATGGCCGAGTTCTAGTGCCCTCAGGCTATGCAATTTCGCCTGAAGACTTCCAGAGTCTAAGCGAAAGCGCAACGATGGTGTACAACGAAAGAGAGTTCGTAAACAGAAAGTTACACCACATTGCGATGCACGGACCAGCCCTGAACACTGACGAAGAGTCGTATGAGCTTGTGAGGGCAGAGAGAACAGAACACGAGTACGTCTACGACGTGGACCAGAGAAGATGCTGTAAGAAGGAAGAAGCTGCAGGATTGGTACTGGTGGGCGACTTGACTAATCCGCCCTACCACGAATTTGCATACGAAGGGCTAAAAATTCGCCCCGCTTGTCCATACAAAATTGCAGTCATAGGAGTCTTCGGGGTACCAGGATCTGGTAAGTCAGCCATTATCAAGAACCTAGTTACCAGGCAAGACCTGGTGACTAGCGGAAAGAAAGAAAACTGCCAAGAAATCAGCACCGACGTGATGAGACAGAGAGGTCTAGAAATATCTGCACGTACGGTTGATTCGCTGCTCTTGAATGGATGCAATAGACCAGTCGACGTGTTGTACGTAGACGAGGCGTTTGCGTGCCACTCTGGAACGTTACTTGCCTTGATCGCCTTGGTGAGACCAAGACTGAAAGTTGTACTTTGTGGTGACCCGAAGCAGTGCGGCTTCTTCAATATGATGCAGATGAAAGTCAACTACAATCATAACATCTGCACTCAAGTGTACCACAAAAGTATCTCCAGGCGGTGTACACTGCCTGTGACTGCCATTGTGTCGTCGTTGCATTACGAAGGCAAAATGCGCACTACGAATGAGTACAACATGCCGATTGTAGTGGACACTACGGGCTCAACGAAACCTGACCCTGGAGACCTCGTGTTAACGTGCTTCAGAGGGTGGGTTAAACAACTGCAAATTGACTATCGTGGACACGAGGTCATGACAGCAGCCGCATCCCAAGGGTTAACCAGAAAAGGAGTTTACGCAGTTAGGCAAAAAGTTAACGAAAACCCACTCTATGCATCAACATCAGAGCACGTCAACGTACTCCTAACGCGTACGGAAGGTAAACTGGTATGGAAGACACTCTCTGGTGACCCGTGGATAAAGACGCTGCAGAACCCACCGAAAGGAAACTTCAAGGCAACTATTAAGGAGTGGGAGGTGGAGCACGCATCGATAATGGCGGGCATCTGCAGTCACCAAGTGACCTTTGACACGTTCCAAAACAAAGCCAACGTTTGCTGGGCTAAGAGCTTGGTCCCTGTCCTCGAAACAGCGGGGATAAAACTAAATGATAGGCAGTGGTCCCAGATAATTCAAGCCTTCAAAGAAGACAAAGCATACTCACCCGAAGTAGCCCTGAATGAAATATGCACGCGCATGTATGGGGTGGATCTAGACAGCGGGCTATTCTCTAAACCGTTGGTATCTGTGTATTACGCGGATAACCACTGGGATAATAGGCCGGGAGGAAAGATGTTCGGATTCAACCCTGAGGCAGCGTCCATTCTAGAAAGAAAGTACCCGTTTACAAAAGGAAAGTGGAACATCAACAAGCAGATCTGCGTGACTACCAGGAGGATAGAAGACTTCAACCCTACCACCAACATTATACCGGCCAACAGGAGACTACCACACTCATTAGTGGCCGAACACCGCCCAGTAAAAGGGGAAAGAATGGAATGGCTGGTTAACAAGATAAACGGACATCATGTGCTCCTGGTTAGCGGCTATAACCTTGCACTGCCTACTAAGAGAGTCACCTGGGTAGCGCCACTAGGTGTCCGCGGAGCGGACTATACATACAACCTAGAGCTGGGTCTACCGGCAACACTTGGTAGGTATGACCTAGTGGTCATAAACATCCACACACCTTTTCGCATACACCATTACCAACAGTGCGTAGATCACGCAATGAAACTGCAAATGCTAGGGGGTGACTCACTGAGACTGCTCAAACCGGGTGGCTCTCTATTGATCAGAGCATACGGTTACGCAGATAGAACCAGTGAACGAGTAATCTGCGTACTGGGACGTAAGTTTAGATCGTCCAGAGCATTGAAACCACCTTGTATCACCAGTAATACTGAGATGTTTTTCCTATTTAGCAGTTTTGACAATGGCAGAAGGAATTTTACAACGCATGTCATGAACAATCAACTGAACGCAGCCTTTGTAGGACAGGCCACCCGAGCAGGATGTGCACCATCGTACCGGGTAAAACGCATGGACATCGCGAAGAACGATGAAGAGTGCGTGGTCAACGCCGCCAACCCTCGCGGGTTACCGGGTGACGGTGTTTGCAAGGCAGTATATAAAAAATGGCCGGAGTCCTTTAAAAATAGTGCAACACCAGTAGGAACTGCAAAAACAGTTATGTGCGGTACATATCCAGTAATCCACGCCGTAGGACCAAACTTCTCAAATTACACGGAGTCCGAAGGGGACCGGGAATTGGCGGCTGCCTATCGAGAAGTCGCAAAGGAAGTAACTAGACTGGGAGTAAATAGCGTAGCTATACCTCTCCTCTCCACAGGTGTATACTCAGGAGGGAAAGACAGGCTAACCCAGTCACTGAACCACCTCTTTACAGCCATGGACTCGACGGATGCAGACGTGGTCATCTACTGCCGAGACAAGGAATGGGAGAAGAAAATATCTGAGGCCATACAGATGCGGACCCAAGTGGAGCTGCTGGATGAGCACATCTCCATAGACTGCGATGTCATTCGCGTGCACCCTGACAGTAGTTTGGCAGGTAGAAAAGGATACAGCACCACGGAAGGCGCACTGTATTCATATCTAGAAGGGACACGTTTTCACCAGACGGCAGTGGATGTGGCAGAGATACATACTATGTGGCCAAAGCAAACAGAGGCCAATGAGCAAGTCTGCCTATATGCCCTGGGGGAAAGTATTGAATCAATCAGGCAGAAATGCCCGGTGGATGATGCAGATGCATCATCTCCCCCGAAAACTGTCCCGTGCCTTTGCCGTTATGCCATGACTCCTGAACGCGTCACCCGACTTCGCATGAACCATGTCACAAATATAATTGTGTGTTCTTCATTTCCCCTTCCAAAGTACAAGATAGAAGGAGTGCAAAAAGTCAAATGCTCCAAGGTAATGTTATTTGATCACAATGTGCCATCGCGCGTAAGTCCAAGGGAATACAGATCTTCCCAGGAGTCTGTACGGGAAGTGAGTATGACAACGTCATTGACGCATAGCCAGTTTGATCTAAGCGCCGATGGCGAGACACTGCCTGTCCCGTCAGACCTGGATGCTGACGCCCCAGCCCTAGAACCGGCCCTAGACGACGGGGCGATCGGAAACCTTGCGGCCGTGTCTGACTGGGTAATGAGCACCGTACCTGTCGCGCCGCCTAGAAGAAGGAGAGGGAGAAACCTGACCGTGATATGTGACGAGAGAGAAGGGAATATAACACCCATGGCTAGCGTCCGATTCTTTAGAGCAGAGCAGTGTCCGGCCGTACAAGAAACAGCGGAGACGCGTGACACAGCTATTTCCCTTCGGGCACCGCCAAGTATCACCGTGGAACTGAGCCATCCACCGATCTCCTTCGGAGCACCAAGCGAGACGTTCCCCATCACATTTGGGGACTTCGACGATGGAGAAATCGAAAGCTTGTCTTCTGAGCTACTAACTTTCGGAGACTTCCTACCCGGTGAAGTGGATGATTTGACAGATAGCGACTGGTCCACGTGCTCAGACACGGACGACGAGTTATGACTAGACAGGGCAGGTGGGTATATATTCTCGTCGGACACTGGTCCAGGCCATTTACAACAGAAGTCGGTACGCCAGTCAGTGCTGCCGGTAAACACCCTGGAGGAAGTTCACGAGGAGAAGTGTTACCCACCTAAGCTGGATGAATTAAAGGAGCAACTACTACTTAAGAAACTCCAGGAGAGTGCGTCCACGGCCAATAGAAGCAGGTATCAATCACGCAAAGTGGAAAATATGAAAGCAACAATCATTCAGAGATTAAAGAGAGGCTGTAAACTGTATTTAATGGCAGAGACCCCGAAAGTCCCGACTTATCGGACCGTATACCCGGCGCCTGTGTACTCGCCTCCGATCAACGTCCGATTGTCCAATCCCGAGTCCGCAGTGGCAGCATGTAATGAGTTCTTAGCTAGAAACTACCCAACTGTTTCATCATACCAAATCACCGATGAGTATGATGCATATCTAGACATGGTGGACGGGTCGGAGAGTTGCTTGGACCGAGCGACATTCAATCCGTCAAAACTCAGGAGCTACCCGAAACAACATGCTTATCACGCGCCTTCTATCAGAAGCGCTGTACCTTCCCCATTCCAGAACACACTACAGAATGTACTGGCAGCAGCCACGAAAAGGAACTGCAACGTCACACAGATGAGGGAATTACCCACTTTGGACTCAGCAGTATTCAACGTGGAGTGTTTTAAAAAATTCGCATGCAACCGAGAATACTGGGAAGAATTTGCTGCCAGCCCTATCAGGATAACAACTGAGAATCTAACAACCTATGTCACTAAACTAAAGGGGCCAAAAGCAGCAGCGTTGTTTGCAAGAACCCATAATCTGCTGCCGCTGCAGGATGTACCAATGGATAGGTTCACAGTAGATATGAAAAGGGACGTGAAGGTAACTCCTGGTACAAAGCATACAGAGGAAAGGCCTAAGGTGCAGGTTATACAGGCGGCTGAACCCTTGGCAACAGCGTACCTATGTGGAATTCACAGAGAATTGGTTAGGAGATTGAACGCCGTCCTCCTACCCAATGTGCATACACTATTTGACATGTCTGCCGAGGACTTCGATGCCATTATAGCCGCACACTTTAAGCCAGGAGACGCCGTTTTAGAAACGGACATAGCCTCCTTTGATAAGAGCCAGGATGATTCACTTGCGCTTACCGCCTTAATGCTGTTAGAAGATTTGGGAGTGGATCACTCCTTGTTGGACCTGATAGAGGCTGCTTTTGGAGAGATTTCCAGCTGTCACCTGCCGACAGGTACGCGCTTCAAGTTCGGCGCTATGATGAAATCCGGTATGTTCCTAACTCTGTTCGTCAACACATTGTTAAATATCACCATCGCTAGCCGGGTGTTGGAAGATCGTCTGACAAAATCTGCATGCGCGGCCTTCATCGGCGACGACAACATAATACATGGTGTCGTCTCCGATGAATTGATGGCAGCCAGATGCGCTACTTGGATGAACATGGAAGTGAAGATCATAGATGCAGTTGTATCCCAGAAAGCTCCTTACTTTTGTGGAGGGTTTATACTGCATGATACTGTGACAGGAACAGCTTGCAGGGTGGCGGACCCGCTAAAAAGGTTATTTAAACTGGGCAAACCGTTAGCGGCAGGTGACGAACAAGACGAAGACAGAAGACGGGCGCTGGCCGATGAAGTAATCAGATGGCAACGAACAGGGCTAATAGATGAGCTGGAGAAAGCGGTGTACTCTAGGTACGAAGTGCAGGGTATATCAGTTGCTGTAATGTCTATGGCCACCTTTGCAAGCTCCAGATCCAACTTCGAGAAGCTCAGAGGACCCGTCATAACTTTGTACGGCGGTCCTAAATAGGTACGCACTACAGCTACCTATTTTGCAGAAGCCGACAGCAGGTACCTAAATACCAATCAGCCATAATGGAGTTTATCCCAACCCAAACTTTCTACAATAGGAGGTACCAGCCTCGACCTTGGACTCCGCGCCCTACTATCCAAGTTATCAGACCCAGACCGCGTCCGCAAAGGAAGGCCGGGCAACTTGCCCAGCTGATCTCAGCAGTTAATAAACTGACAATGCGCGTGGTACCTCAACAGAAGCCGCGCAAGAATCGGAAGAATAAGAAGCAAAAGCAAAAGCAGCAGGCGCCACGAAACAATATGAATCAAAAGAAGCAGCCCCCTAAAAAGAAACCGGTTCAAAAGAAAAAGAAGCCGGGCCGCAGAGAGAGAATGTGCATGAAAATCGAAAATGATTGCATCTTCGAAGTCAAGCATGAAGGTAAGGTAACAGGTTACGCGTGCTTGGTAGGGGACAAAGTAATGAAGCCAGCACACGTAAAGGGGACCATCGATAACGCGGACCTGGCCAAATTGGCCTTCAAGCGGTCATCTAAGTACGACCTTGAATGCGCGCAGATACCCGTGCACATGAAGTCCGACGCTTCGAAGTTCACCCATGAGAAACCGGAGGGGTACTACAACTGGCACCACGGAGCAGTACAGTACTCAGGAGGCCGATTCACCATCCCTACAGGTGCGGGCAAACCAGGGGATAGTGGTAGACCGATCTTCGACAACAAGGGGCGCGTGGTGGCCATAGTTTTAGGAGGAGCTAATGAAGGAGCCCGTACAGCCCTCTCGGTGGTGACCTGGAACAAAGACATCGTCACGAAAATCACCCCTGAGGGGGCCGAAGAGTGGAGTCTGGCCATTCCAGTTATGTGCCTGCTGGCAAATACCACGTTCCCCTGCTCCCAGCCCCCTTGCACACCCTGCTGCTACGAAAAAGAGCCGGAGAAAACCTTGCGCATGCTTGAAGACAATGTCATGAGCCCCGGGTACTATCAGCTGCTACAAGCATCCTTAACATGTTCTCCCCGACGCCGGCGGCGCAGTATTAAGGACCACTTCAATGTCTATAAAGCCACAAGACCGTACCTAGCTCACTGTCCCGACTGTGGAGAAGGGCACTCGTGCCATAGTCCCGTAGCGCTAGAACGCATCAGAAACGAAGCGACAGACGGGACGTTGAAAATCCAGGTTTCCTTGCAAATCGGAATAAAGACGGATGATAGCCATGATTGGACCAAGCTGCGTTATATGGACAATCACATGCCAGCAGACGCAGAGCGGGCCGGGCTATTTGTAAGAACGTCAGCACCGTGCACGATTACTGGAACAATGGGACACTTCATTCTGGCCCGATGTCCGAAAGGAGAAACTCTGACGGTGGGGTTCACTGATGGTAGAAAGATCAGTCACTCATGTACGCACCCATTTCACCACGACCCTCCTGTGATAGGCCGGGAAAAATTCCATTCCCGACCGCAGCACGGTAGGGAACTACCTTGCAGCACGTACGCGCAGAGCACCGCTGCAACTGCCGAGGAGATAGAGGTACATATGCCCCCAGACACCCCAGATCGCACATTAATGTCACAACAGTCCGGCAATGTAAAGATCACAGTCAATAGTCAGACGGTGCGGTACAAGTGCAATTGTGGTGACTCAAGTGAAGGATTAACCACTACAGATAAAGTGATTAATAACTGCAAGGTTGATCAATGCCATGCCGCGGTCACCAATCACAAAAAATGGCAGTATAATTCCCCTCTGGTCCCGCGCAATGCTGAATCCGGGGACCGGAAAGGAAAAGTTCACATTCCATTTCCTCTGGCAAATGTGACATGCAGGGTGCCTAAAGCAAGAAACCCCACCGTGACGTACGGAAAAAACCAAGTCATCATGTTGCTGTATCCTGACCACCCAACGCTCCTGTCCTACAGGAATATGGGAGAAGAACCAAACTATCAAGAAGAGTGGGTGACGCATAAGAAGGAGATCAGGTTAACCGTGCCGACTGAAGGGCTCGAGGTCACGTGGGGTAACAATGAGCCGTACAAGTATTGGCCGCAGTTATCCACAAACGGTACAGCCCACGGCCACCCGCATGAGATAATTCTGTATTATTATGAGCTGTACCCAACTATGACTGTGGTAGTTGTGTCAGTGGCCTCGTTCATACTCCTGTCGATGGTGGGTGTGGCAGTGGGGATGTGCATGTGTGCACGACGCAGATGCATTACACCGTACGAACTGACACCAGGAGCTACCGTCCCTTTCCTGCTTAGCCTAATATGCTGCATTAGAACAGCTAAAGCGGCCACATACCAGGAGGCCGCGGTATACCTGTGGAACGAGCAGCAGCCTTTATTTTGGCTGCAAGCCCTTATTCCGCTGGCAGCCCTGATTGTCCTATGTAACTGTCTGAGACTCTTACCATGCTGTTGTAAAATGTTGACTTTTTTAGCCGTACTGAGCGTCGGTGCCCACACTGTGAGCGCGTACGAACACGTAACAGTGATCCCGAACACGGTGGGAGTACCGTATAAGACTCTAGTCAACAGACCGGGCTACAGCCCCATGGTACTGGAGATGGAGCTTCTGTCTGTCACCTTGGAACCAACGCTATCGCTTGATTACATCACGTGCGAGTATAAAACCGTTATCCCGTCTCCGTACGTGAAATGCTGCGGTACAGCAGAGTGTAAGGACAAGAGCCTACCTGATTACAGCTGTAAGGTCTTCACCGGCGTCTACCCATTCATGTGGGGCGGCGCCTACTGCTTCTGCGACACCGAAAATACGCAATTGAGCGAAGCACATGTGGAGAAGTCCGAATCATGCAAAACAGAATTTGCATCAGCATACAGGGCTCATACCGCATCCGCATCAGCTAAGCTCCGCGTCCTTTACCAAGGAAATAATATCACTGTAGCTGCTTATGCAAACGGCGACCATGCCGTCACAGTTAAGGACGCCAAATTCATAGTGGGGCCAATGTCTTCAGCCTGGACACCTTTCGACAATAAAATCGTGGTGTACAAAGGCGACGTCTACAACATGGACTACCCGCCCTTCGGCGCAGGAAGACCAGGACAATTTGGCGACATCCAAAGTCGCACGCCTGAGAGCGAAGACGTCTATGCTAATACACAACTGGTACTGCAGAGACCGTCCGCGGGTACGGTGCACGTGCCGTACTCTCAGGCACCATCTGGCTTCAAGTATTGGCTAAAAGAACGAGGGGCGTCGCTACAGCACACAGCACCATTTGGCTGTCAAATAGCAACAAACCCGGTAAGAGCGATGAACTGCGCCGTAGGGAACATGCCTATCTCCATCGACATACCGGACGCGGCCTTTACCAGGGTCGTCGACGCGCCATCTTTAACGGACATGTCGTGTGAGGTATCAGCCTGCACCCATTCCTCAGACTTTGGGGGCGTAGCCATCATTAAATATGCAGCCAGTAAGAAAGGCAAGTGTGCAGTGCACTCGATGACTAACGCCGTCACTATTCGGGAAGCTGAAATAGAAGTGGAAGGGAACTCTCAGTTGCAAATCTCTTTTTCGACGGCCCTAGCCAGCGCCGAATTCCGCGTACAAGTCTGTTCTACACAAGTACACTGTGCAGCCGAGTGCCATCCACCGAAAGACCATATAGTCAATTACCCGGCGTCACACACCACCCCCGGGGTCCAAGACATTTCCGCTACGGCGATGTCATGGGTGCAGAAGATCACGGGAGGTGTGGGACTGGTTGTCGCTGTTGCAGCACTGATCCTAATCGTGGTGCTATGCGTGTCGTTTAGCAGGCACTAACTTGACAACTAGGTATGAAGGCATACGCGTCCCTAAAGAGACACACCACATATAGCTAAGAATCAATAGATAAGTATAGATCAAAGGGCTGAACAATCCCTGAATAGTAACAAAATATAAAAATCAACAAAAATCATAAAATAGAAAACTAGAAATAGAAGTAGGTAAGAAGGTATATGTGTCCCCTAAGAGACACACCATATATAGCTAAGAATCAATAGATAAGCATAGATCAAAGGGCTGAATAACCCCTGAATAATAACAAAATATAAAAACCAATAAAAATCATAAAATAGAAAACCATAAACAGAAGTAGTTCAAAGGGCTATGAAACCCCTGAATAGTAACAAAATATAAAACTAATAAAAATC

>FN295484|2006|Malaysia:Perak, Group 1

CAAAGCAAGAGATCAATAACCCATCATGGATTCTGTGTACGTGGATATAGACGCTGACAGCGCCTTTTTGAAGGCCCTGCAACGTGCGTACCCCATGTTTGAGGTGGAACCTAGGCAGGTCACATCAAATGACCATGCTAATGCTAGAGCGTTCTCGCATCTAGCCATAAAACTAATAGAGCAGGAAATTGATCCCGACTCAACCATCCTGGATATAGGTAGTGCGCCAGCAAGGAGGATGATGTCGGACAGGAAGTACCACTGCGTTTGCCCGATGCGCAGCGCAGAAGATCCCGAGAGACTCGCTAATTATGCGAGAAAGCTCGCATCTGCCGCAGGAAAAGTCCTGGACAGAAACATTTCTGGAAAGATCGGGGACTTACAAGCGGTGATGGCCGTGCCAGACACGGAGACGCCAACATTTTGCTTACACACAGATGTCTCATGTAGACAGAGAGCAGACGTCGCGATATACCAAGACGTCTATGCTGTACATGCACCCACGTCGCTATATCACCAGGCGATTAAAGGAGTCCGAGTGGCGTACTGGGTAGGGTTTGACACAACCCCGTTCATGTACAACGCTATGGCGGGTGCCTACCCCTCATACTCGACAAATTGGGCGGATGAGCAGGTACTGAAGGCTAAGAACATAGGATTATGTTCAACAGACCTGACGGAAGGTAGACGAGGCAAATTGTCTATCATGAGAGGGAAAAAGCTAAAACCGTGCGACCGTGTGCTGTTCTCAGTAGGGTCAACGCTTTACCCGGAAAGCCGCATGCTACTTAAGAGCTGGCACCTACCATCGGTGTTCCATCTAAAGGGCAAGCTTAGCTTCACATGCCGCTGTGACACAGTGGTTTCGTGTGAGGGCTACGTCGTCAAGAGAATAACGATGAGCCCAGGCCTTTATGGAAAAACCACGGGGTATGCGGTAACCCACCACGCAGACGGATTCTTGATGTGCAAGACTACCGATACGGTTGACGGCGAAAGAGTGTCATTCTCGGTGTGCACGTACGTGCCGGCGACCATTTGTGATCAAATGACCGGCATCCTTGCTACAGAAGTCACGCCGGAGGATGCACAGAAGCTGTTGGTGGGGCTGAACCAGAGAATAGTGGTTAACGGCAGAACGCAACGGAACACGAACACCATGAAGAACTATCTACTTCCCGTGGTCGCCCAGGCCTTCAGTAAGTGGGCGAAGGAGTGCCGGAAGGACATGGAAGATGAGAAGCTTCTGGGGGTCAGAGAAAGAACACTAACCTGCTGCTGTCTGTGGGCATTTAAGAAGCAGAAAACACACACGGTCTACAAGAGGCCTGATACCCAGTCAATCCAGAAGGTTCAGGCCGAATTTGACAGCTTTGTAGTACCAGGCCTGTGGTCGTCCGGGTTGTCAATCCCGTTGAGGACTAGAATCAAGTGGCTGTTACGCAAGGTGCCGAAAGCAGACCTGATCCCATACAGCGGAAATGCCCAAGAAGCCCAGGATGCAGAAAAAGAAGCAGAGGAAGAACGAGAAGCAGAACTGACTCATGAGGCTCTACCACCCCTACAGGCAGCACAGGAAGATGTCCAGGTCGAAATCGACGTGGAACAACTTGAGGATAGAGCTGGTGCTGGAATAATAGAGACTCCGAGAGGCGCTATTAAAGTTACTGCCCAACTAACCGACCACGTCGTGGGGGAGTACCTGGTACTTTCCCCGCAGACCGTATTACGCAGCCAGAAGCTCAGCCTGATCCACGCTTTAGCGGAGCAAGTGAAGACGTGTACGCATAGCGGACGAGCAGGGAGGTATGCGGTCGAAGCGTACGATGGCCGAGTTCTAGTGCCCTCAGGCTATGCAATTTCGCCTGAAGACTTCCAGAGTCTAAGCGAAAGCGCAACGATGGTGTACAACGAAAGAGAGTTCGTAAACAGAAAGTTACACCACATTGCGATGCACGGACCAGCCCTGAACACTGACGAAGAGTCGTATGAGCTTGTGAGGGCAGAGAGAACAGAACACGAGTACGTCTACGACGTGGACCAGAGAAGATGCTGTAAGAAGGAAGAAGCTGCAGGATTGGTACTGGTGGGCGACTTGACTAATCCGCCCTACCACGAATTTGCATACGAAGGGCTAAAAATTCGCCCCGCTTGTCCATACAAAATTGCAGTCATAGGAGTCTTCGGGGTACCAGGATCTGGTAAGTCAGCCATTATCAAGAACCTAGTTACCAGGCAAGACCTGGTGACTAGCGGAAAGAAAGAAAACTGCCAAGAAATCAGCACCGACGTGATGAGACAGAGAGGTCTAGAAATATCTGCACGTACGGTTGATTCGCTGCTCTTGAATGGATGCAATAGACCAGTCGACGTGTTGTACGTAGACGAGGCGTTTGCGTGCCACTCTGGAACGTTACTTGCCTTGATCGCCTTGGTGAGACCAAGACTGAAAGTTGTACTTTGTGGTGACCCGAAGCAGTGCGGCTTCTTCAATATGATGCAGATGAAAGTCAACTACAATCATAACATCTGCACTCAAGTGTACCACAAAAGTATCTCCAGGCGGTGTACACTGCCTGTGACTGCCATTGTGTCGTCGTTGCATTACGAAGGCAAAATGCGCACTACGAATGAGTACAACATGCCGATTGTAGTGGACACTACGGGCTCAACGAAACCTGACCCTGGAGACCTCGTGTTAACGTGCTTCAGAGGGTGGGTTAAACAACTGCAAATTGACTATCGTGGACACGAGGTCATGACAGCAGCCGCATCCCAAGGGTTAACCAGAAAAGGAGTTTACGCAGTTAGGCAAAAAGTTAACGAAAACCCACTCTATGCATCAACATCAGAGCACGTCAACGTACTCCTAACGCGTACGGAAGGTAAACTGGTATGGAAGACACTCTCTGGTGACCCGTGGATAAAGACGCTGCAGAACCCACCGAAAGGAAACTTCAAGGCAACTATTAAGGAGTGGGAGGTGGAGCACGCATCGATAATGGCGGGCATCTGCAGTCACCAAGTGACCTTTGACACGTTCCAAAACAAAGCCAACGTTTGCTGGGCTAAGAGCTTGGTCCCTGTCCTCGAAACAGCGGGGATAAAACTAAATGATAGGCAGTGGTCCCAGATAATTCAAGCCTTCAAAGAAGACAAAGCATACTCACCCGAAGTAGCCCTGAATGAAATATGCACGCGCATGTATGGGGTGGATCTAGACAGCGGGCTATTCTCTAAACCGTTGGTATCTGTGTATTACGCGGATAACCACTGGGATAATAGGCCGGGAGGAAAGATGTTCGGATTCAACCCTGAGGCAGCGTCCATTCTAGAAAGAAAGTACCCGTTTACAAAAGGAAAGTGGAACATCAACAAGCAGATCTGCGTGACTACCAGGAGGATAGAAGACTTCAACCCTACCACCAACATTATACCGGCCAACAGGAGACTACCACACTCATTAGTGGCCGAACACCGCCCAGTAAAAGGGGAAAGAATGGAATGGCTGGTTAACAAGATAAACGGACATCATGTGCTCCTGGTTAGCGGCTATAACCTTGCACTGCCTACTAAGAGAGTCACCTGGGTAGCGCCACTAGGTGTCCGCGGAGCGGACTATACATACAACCTAGAGCTGGGTCTACCGGCAACACTTGGTAGGTATGACCTAGTGGTCATAAACATCCACACACCTTTTCGCATACACCATTACCAACAGTGCGTAGATCACGCAATGAAACTGCAAATGCTAGGGGGTGACTCACTGAGACTGCTCAAACCGGGTGGCTCTCTATTGATCAGAGCATACGGTTACGCAGATAGAACCAGTGAACGAGTAATCTGCGTACTGGGACGTAAGTTTAGATCGTCCAGAGCATTGAAACCACCTTGTATCACCAGTAATACTGAGATGTTTTTCCTATTTAGCAGTTTTGACAATGGCAGAAGGAATTTTACAACGCATGTCATGAACAATCAACTGAACGCAGCCTTTGTAGGACAGGCCACCCGAGCAGGATGTGCACCATCGTACCGGGTAAAACGCATGGACATCGCGAAGAACGATGAAGAGTGCGTGGTCAACGCCGCCAACCCTCGCGGGTTACCGGGTGACGGTGTTTGCAAGGCAGTATATAAAAAATGGCCGGAGTCCTTTAAAAATAGTGCAACACCAGTAGGAACTGCAAAAACAGTTATGTGCGGTACATATCCAGTAATCCACGCCGTAGGACCAAACTTCTCAAATTACACGGAGTCCGAAGGGGACCGGGAATTGGCGGCTGCCTATCGAGAAGTCGCAAAGGAAGTAACTAGACTGGGAGTAAATAGCGTAGCTATACCTCTCCTCTCCACAGGTGTATACTCAGGAGGGAAAGACAGGCTAACCCAGTCACTGAACCACCTCTTTACAGCCATGGACTCGACGGATGCAGACGTGGTCATCTACTGCCGAGACAAGGAATGGGAGAAGAAAATATCTGAGGCCATACAGATGCGGACCCAAGTGGAGCTGCTGGATGAGCACATCTCCATAGACTGCGATGTCATTCGCGTGCACCCTGACAGTAGTTTGGCAGGTAGAAAAGGATACAGCACCACGGAAGGCGCACTGTATTCATATCTAGAAGGGACACGTTTTCACCAGACGGCAGTGGATGTGGCAGAGATACATACTATGTGGCCAAAGCAAACAGAGGCCAATGAGCAAGTCTGCCTATATGCCCTGGGGGAAAGTATTGAATCAATCAGGCAGAAATGCCCGGTGGATGATGCAGATGCATCATCTCCCCCGAAAACTGTCCCGTGCCTTTGCCGTTATGCCATGACTCCTGAACGCGTCACCCGACTTCGCATGAACCATGTCACAAATATAATTGTGTGTTCTTCATTTCCCCTTCCAAAGTACAAGATAGAAGGAGTGCAAAAAGTCAAATGCTCCAAGGTAATGTTATTTGATCACAATGTGCCATCGCGCGTAAGTCCAAGGGAATACAGATCTTCCCAGGAGTCTGTACGGGAAGTGAGTATGACAACGTCATTGACGCATAGCCAGTTTGATCTAAGCGCCGATGGCGAGACACTGCCTGTCCCGTCAGACCTGGATGCTGACGCCCCAGCCCTAGAACCGGCCCTAGACGACGGGGCGATCGGAAACCTTGCGGCCGTGTCTGACTGGGTAATGAGCACCGTACCTGTCGCGCCGCCTAGAAGAAGGAGAGGGAGAAACCTGACCGTGATATGTGACGAGAGAGAAGGGAATATAACACCCATGGCTAGCGTCCGATTCTTTAGAGCAGAGCAGTGTCCGGCCGTACAAGAAACAGCGGAGACGCGTGACACAGCTATTTCCCTTCGGGCACCGCCAAGTATCACCGTGGAACTGAGCCATCCACCGATCTCCTTCGGAGCACCAAGCGAGACGTTCCCCATCACATTTGGGGACTTCGACGATGGAGAAATCGAAAGCTTGTCTTCTGAGCTACTAACTTTCGGAGACTTCCTACCCGGTGAAGTGGATGATTTGACAGATAGCGACTGGTCCACGTGCTCAGACACGGACGACGAGTTATGACTAGACAGGGCAGGTGGGTATATATTCTCGTCGGACACTGGTCCAGGCCATTTACAACAGAAGTCGGTACGCCAGTCAGTGCTGCCGGTAAACACCCTGGAGGAAGTTCACGAGGAGAAGTGTTACCCACCTAAGCTGGATGAATTAAAGGAGCAACTACTACTTAAGAAACTCCAGGAGAGTGCGTCCACGGCCAATAGAAGCAGGTATCAATCACGCAAAGTGGAAAATATGAAAGCAACAATCATTCAGAGATTAAAGAGAGGCTGTAAACTGTATTTAATGGCAGAGACCCCGAAAGTCCCGACTTATCGGACCGTATACCCGGCGCCTGTGTACTCGCCTCCGATCAACGTCCGATTGTCCAATCCCGAGTCCGCAGTGGCAGCATGTAATGAGTTCTTAGCTAGAAACTACCCAACTGTTTCATCATACCAAATCACCGATGAGTATGATGCATATCTAGACATGGTGGACGGGTCGGAGAGTTGCTTGGACCGAGCGACATTCAATCCGTCAAAACTCAGGAGCTACCCGAAACAACATGCTTATCACGCGCCTTCTATCAGAAGCGCTGTACCTTCCCCATTCCAGAACACACTACAGAATGTACTGGCAGCAGCCACGAAAAGGAACTGCAACGTCACACAGATGAGGGAATTACCCACTTTGGACTCAGCAGTATTCAACGTGGAGTGTTTTAAAAAATTCGCATGCAACCGAGAATACTGGGAAGAATTTGCTGCCAGCCCTATCAGGATAACAACTGAGAATCTAACAACCTATGTCACTAAACTAAAGGGGCCAAAAGCAGCAGCGTTGTTTGCAAGAACCCATAATCTGCTGCCGCTGCAGGATGTACCAATGGATAGGTTCACAGTAGATATGAAAAGGGACGTGAAGGTAACTCCTGGTACAAAGCATACAGAGGAAAGGCCTAAGGTGCAGGTTATACAGGCGGCTGAACCCTTGGCAACAGCGTACCTATGTGGAATTCACAGAGAATTGGTTAGGAGATTGAACGCCGTCCTCCTACCCAATGTGCATACACTATTTGACATGTCTGCCGAGGACTTCGATGCCATTATAGCCGCACACTTTAAGCCAGGAGACGCCGTTTTAGAAACGGACATAGCCTCCTTTGATAAGAGCCAGGATGATTCACTTGCGCTTACCGCCTTAATGCTGTTAGAAGATTTGGGAGTGGATCACTCCTTGTTGGACCTGATAGAGGCTGCTTTTGGAGAGATTTCCAGCTGTCACCTGCCGACAGGTACGCGCTTCAAGTTCGGCGCTATGATGAAATCCGGTATGTTCCTAACTCTGTTCGTCAACACATTGTTAAATATCACCATCGCTAGCCGGGTGTTGGAAGATCGTCTGACAAAATCTGCATGCGCGGCCTTCATCGGCGACGACAACATAATACATGGTGTCGTCTCCGATGAATTGATGGCAGCCAGATGCGCTACTTGGATGAACATGGAAGTGAAGATCATAGATGCAGTTGTATCCCAGAAAGCTCCTTACTTTTGTGGAGGGTTTATACTGCATGATACTGTGACAGGAACAGCTTGCAGGGTGGCGGACCCGCTAAAAAGGTTATTTAAACTGGGCAAACCGTTAGCGGCAGGTGACGAACAAGACGAAGACAGAAGACGGGCGCTGGCCGATGAAGTAATCAGATGGCAACGAACAGGGCTAATAGATGAGCTGGAGAAAGCGGTGTACTCTAGGTACGAAGTGCAGGGTATATCAGTTGCTGTAATGTCTATGGCCACCTTTGCAAGCTCCAGATCCAACTTCGAGAAGCTCAGAGGACCCGTCATAACTTTGTACGGCGGTCCTAAATAGGTACGCACTACAGCTACCTATTTTGCAGAAGCCGACAGCAGGTACCTAAATACCAATCAGCCATAATGGAGTTTATCCCAACCCAAACTTTCTACAATAGGAGGTACCAGCCTCGACCTTGGACTCCGCGCCCTACTATCCAAGTTATCAGACCCAGACCGCGTCCGCAAAGGAAGGCCGGGCAACTTGCCCAGCTGATCTCAGCAGTTAATAAACTGACAATGCGCGTGGTACCTCAACAGAAGCCGCGCAAGAATCGGAAGAATAAGAAGCAAAAGCAAAAGCAGCAGGCGCCACGAAACAATATGAATCAAAAGAAGCAGCCCCCTAAAAAGAAACCGGTTCAAAAGAAAAAGAAGCCGGGCCGCAGAGAGAGAATGTGCATGAAAATCGAAAATGATTGCATCTTCGAAGTCAAGCATGAAGGTAAGGTAACAGGTTACGCGTGCTTGGTAGGGGACAAAGTAATGAAGCCAGCACACGTAAAGGGGACCATCGATAACGCGGACCTGGCCAAATTGGCCTTCAAGCGGTCATCTAAGTACGACCTTGAATGCGCGCAGATACCCGTGCACATGAAGTCCGACGCTTCGAAGTTCACCCATGAGAAACCGGAGGGGTACTACAACTGGCACCACGGAGCAGTACAGTACTCAGGAGGCCGATTCACCATCCCTACAGGTGCGGGCAAACCAGGGGATAGTGGTAGACCGATCTTCGACAACAAGGGGCGCGTGGTGGCCATAGTTTTAGGAGGAGCTAATGAAGGAGCCCGTACAGCCCTCTCGGTGGTGACCTGGAACAAAGACATCGTCACGAAAATCACCCCTGAGGGGGCCGAAGAGTGGAGTCTGGCCATTCCAGTTATGTGCCTGCTGGCAAATACCACGTTCCCCTGCTCCCAGCCCCCTTGCACACCCTGCTGCTACGAAAAAGAGCCGGAGAAAACCTTGCGCATGCTTGAAGACAATGTCATGAGCCCCGGGTACTATCAGCTGCTACAAGCATCCTTAACATGTTCTCCCCGACGCCGGCGGCGCAGTATTAAGGACCACTTCAATGTCTATAAAGCCACAAGACCGTACCTAGCTCACTGTCCCGACTGTGGAGAAGGGCACTCGTGCCATAGTCCCGTAGCGCTAGAACGCATCAGAAACGAAGCGACAGACGGGACGTTGAAAATCCAGGTTTCCTTGCAAATCGGAATAAAGACGGATGATAGCCATGATTGGACCAAGCTGCGTTATATGGACAATCACATGCCAGCAGACGCAGAGCGGGCCGGGCTATTTGTAAGAACGTCAGCACCGTGCACGATTACTGGAACAATGGGACACTTCATTCTGGCCCGATGTCCGAAAGGAGAAACTCTGACGGTGGGGTTCACTGATGGTAGAAAGATCAGTCACTCATGTACGCACCCATTTCACCACGACCCTCCTGTGATAGGCCGGGAAAAATTCCATTCCCGACCGCAGCACGGTAGGGAACTACCTTGCAGCACGTACGCGCAGAGCACCGCTGCAACTGCCGAGGAGATAGAGGTACATATGCCCCCAGACACCCCAGATCGCACATTAATGTCACAACAGTCCGGCAATGTAAAGATCACAGTCAATAGTCAGACGGTGCGGTACAAGTGCAATTGTGGTGACTCAAGTGAAGGATTAACCACTACAGATAAAGTGATTAATAACTGCAAGGTTGATCAATGCCATGCCGCGGTCACCAATCACAAAAAATGGCAGTATAATTCCCCTCTGGTCCCGCGCAATGCTGAATCCGGGGACCGGAAAGGAAAACTTCACATTCCATTTCCTCTGGCAAATGTGACATGCAGGGTGCCTAAAGCAAGAAACCCCACCGTGACGTACGGAAAAAACCAAGTCATCATGTTGCTGTATCCTGACCACCCAACGCTCCTGTCCTACAGGAATATGGGAGAAGAACCAAACTATCAAGAAGAGTGGGTGACGCATAAGAAGGAGATCAGGTTAACCGTGCCGACTGAAGGGCTCGAGGTCACGTGGGGTAACAATGAGCCGTACAAGTATTGGCCGCAGTTATCCACAAACGGTACAGCCCACGGCCACCCGCATGAGATAATTCTGTATTATTATGAGCTGTACCCAACTATGACTGTGGTAGTTGTGTCAGTGGCCTCGTTCATACTCCTGTCGATGGTGGGTGTGGCAGTGGGGATGTGCATGTGTGCACGACGCAGATGCATTACACCGTACGAACTGACACCAGGAGCTACCGTCCCTTTCCTGCTTAGCCTAATATGCTGCATTAGGACAGCTAAAGCGGCCACATACCAGGAGGCCGCGGTATACCTGTGGAACGAGCAGCAGCCTTTATTTTGGCTGCAAGCCCTTATTCCGCTGGCAGCCCTGATTGTCCTATGTAACTGTCTGAGACTCTTACCATGCTGTTGTAAAATGTTGACTTTTTTAGCCGTACTGAGCGTCGGTGCCCACACTGTGAGCGCGTACGAACACGTAACAGTGATCCCGAACACGGTGGGAGTACCGTATAAGACTCTAGTCAACAGACCGGGCTACAGCCCCATGGTACTGGAGATGGAGCTTCTGTCTGTCACCTTGGAACCAACGCTATCGCTTGATTACATCACGTGCGAGTATAAAACCGTTATCCCGTCTCCGTACGTGAAATGCTGCGGTACAGCAGAGTGTAAGGACAAGAGCCTACCTGATTACAGCTGTAAGGTCTTCACCGGCGTCTACCCATTCATGTGGGGCGGCGCCTACTGCTTCTGCGACACCGAAAATACGCAATTGAGCGAAGCACATGTGGAGAAGTCCGAATCATGCAAAACAGAATTTGCATCAGCATACAGGGCTCATACCGCATCCGCATCAGCTAAGCTCCGCGTCCTTTACCAAGGAAATAATATCACTGTAGCTGCTTATGCAAACGGCGACCATGCCGTCACAGTTAAGGACGCCAAATTCATAGTGGGGCCAATGTCTTCAGCCTGGACACCTTTCGACAATAAAATCGTGGTGTACAAAGGCGACGTCTACAACATGGACTACCCGCCCTTCGGCGCAGGAAGACCAGGACAATTTGGCGACATCCAAAGTCGCACGCCTGAGAGCGAAGACGTCTATGCTAATACACAACTGGTACTGCAGAGACCGTCCGCGGGTACGGTGCACGTGCCGTACTCTCAGGCACCATCTGGCTTCAAGTATTGGCTAAAAGAACGAGGGGCGTCGCTACAGCACACAGCACCATTTGGCTGTCAAATAGCAACAAACCCGGTAAGAGCGATGAACTGCGCCGTAGGGAACATGCCTATCTCCATCGACATACCGGACGCGGCCTTTACCAGGGTCGTCGACGCGCCATCTTTAACGGACATGTCGTGTGAGGTATCAGCCTGCACCCATTCCTCAGACTTTGGGGGCGTAGCCATCATTAAATATGCAGCCAGTAAGAAAGGCAAGTGTGCAGTGCACTCGATGACTAACGCCGTCACTATTCGGGAAGCTGAAATAGAAGTGGAAGGGAACTCTCAGTTGCAAATCTCTTTTTCGACGGCCCTAGCCAGCGCCGAATTCCGCGTACAAGTCTGTTCTACACAAGTACACTGTGCAGCCGAGTGCCATCCACCGAAAGACCATATAGTCAATTACCCGGCGTCACACACCACCCCCGGGGTCCAAGACATTTCCGCTACGGCGATGTCATGGGTGCAGAAGATCACGGGAGGTGTGGGACTGGTTGTCGCTGTTGCAGCACTGATCCTAATCGTGGTGCTATGCGTGTCGTTTAGCAGGCACTAACTTGACAACTAGGTATGAAGGCATACGCGTCCCTAAAGAGACACACCACATATAGCTAAGAATCAATAGATAAGTATAGATCAAAGGGCTGAACAATCCCTGAATAGTAACAAAATATAAAAATCAACAAAAATCATAAAATAGAAAACTAGAAATAGAAGTAGGTAAGAAGGTATATGTGTCCCCTAAGAGACACACCATATATAGCTAAGAATCAATAGATAAGCATAGATCAAAGGGCTGAATAACCCCTGAATAATAACAAAATATAAAAACCAATAAAAATCATAAAATAGAAAACCATAAACAGAAGTAGTTCAAAGGGCTATGAAACCCCTGAATAGTAACAAAATATAAAACTAATAAAAATC

>KJ451622|2013|Micronesia: Yap State, Group 1

ATGGCTGCGTGAGACACACGTAGCCTACTAGTTTCTTACTGCTCTACTCTGCAAAGCAAGAGATTAATAACCCATCATGGATTCTGTGTACGTGGATATAGACGCTGACAGCGCCTTTTTGAAGGCCCTGCAACGTGCGTACCCCATGTTTGAGGTGGAACCTAGGCAGGTCACATCAAATGACCATGCTAATGCTAGAGCGTTCTCGCATCTAGCCATAAAACTAATAGAGCAGGAAATTGATCCCGACTCAACCATCCTGGATATAGGTAGTGCGCCAGCAAGGAGGATGATGTCGGACAGGAAGTACCACTGCGTTTGCCCGATGCGCAGCGCAGAAGATCCCGAGAGACTCGCTAATTATGCGAGAAAGCTCGCATCTGCCGCAGGAAAAGTCCTGGACAGAAACATTTCTGGAAAGATCGGGGACTTACAAGAGGTGATGGCCGTGCCAGACACGGAGACGCCAACATTTTGCTTACACACAGATGTCTCATGTAGACAGAGAGCAGACGTCGCGATATACCAAGACGTCTATGCTGTACATGCACCCACGTCGCTATATCACCAGGCGATTAAAGGAGTCCGAGTGGCGTACTGGGTAGGGTTCGACACAACCCCGTTCATGTACAACGCTATGGCGGGTGCCTACCCCTCATACTCGACAAATTGGGCGGATGAGCAGGTACTGAAGGCTAAGAACATAGGATTATGTTCAACAGACCTGACAGAAGGTAGACGAGGCAAATTGTCTATCATGAGAGGGAAAAAGCTAAAACCGTGCGACCGTGTGCTGTTCTCAGTAGGGTCAACGCTTTACCCGGAAAGCCGCATGCTACTTAAGAGCTGGCACCTACCATCGGTGTTCCATCTAAAGGGCAAGCTTAGCTTCACATGCCGCTGTGACACAGTGGTTTCGTGTGAGGGCTACGTCGTTAAGAGAATAACGATGAGCCCAGGCCTTTATGGAAAAACCACGGGGTATGCGGTAACCCACCACGCAGACGGATTCTTGATGTGCAAGACTACCGACACGGTAGACGGTGAAAGAGTGTCATTCTCGGTGTGCACGTACGTGCCGGCGACCATTTGTGATCAAATGACCGGCATCCTTGCTACAGAAGTCACGCCGGAGGATGCACAGAAGCTGTTGGTGGGGCTGAACCAGAGAATAGTGGTTAACGGCAGAACGCAACGGAACACGAACACCATGAAGAACTACCTGCTTCCCGTGGTCGCCCAGGCCTTCAGTAAGTGGGCGAAGGAGTGCCGGAAGGACATGGAAGATGAGAAGCTTCTGGGGGTCAGAGAAAGAACACTGACCTGCTGCTGTCTGTGGGCATTTAAGAAGCAGAAAACACACACGGTCTACAAGAGGCCTGATACCCAGTCAATCCAGAAGGTTCAGGCCGAATTCGACAGCTTTGTAGTACCAGGCCTGTGGTCGTCCGGGTTGTCAATCCCGTTGAGGACTAGAATCAAGTGGCTGTTACGCAAGGTGCCGAAAGCAGACCTGATCCCATACAGCGGAAATGCCCAAGAAGCCCAGGATGCTGAAAAAGAAGCAGAGGAAGAACGAGAAGCAGAACTGACTCATGAGGCTCTACCACCCCTACAGGCAGCACAGGAAGATGTCCAGGTCGAAATCGACGTGGAACAACTTGAGGATAGAGCTGGTGCTGGAATAATAGAGACTCCGAGAGGCGCTATTAAAGTTACTGCCCAACTAACGGACCACGTCGTGGGGGAGTACCTGGTACTTTCCCCGCAGACCGTATTACGCAGCCAGAAGCTCAGCCTGATCCACGCTTTAGCGGAGCAAGTGAAGACGTGTACGCATAGCGGACGAGCAGGGAGGTATGCGGTCGAAGCGTACGATGGCCGAGTCCTAGTGCCCTCAGGCTATGCAATTTCGCCCGAAGACTTCCAGAGTCTAAGCGAAAGCGCAACGATGGTGTACAACGAAAGAGAGTTCGTAAACAGAAAGTTACACCACATTGCGATGCACGGACCAGCTCTGAACACTGACGAAGAGTCGTATGAGCTTGTGAGGGCAGAGAGGACAGAACACGAGTACGTCTACGACGTGGACCAGAGAAGATGCTGCAAGAAGGAAGAAGCTGCAGGATTGGTACTGGTGGGCGACTTGACTAATCCGCCCTACCACGAATTTGCATACGAAGGGCTAAAAATTCGCCCCGCTTGTCCATATAAAATTGCAGTCATAGGAGTCTTCGGGGTACCAGGATCTGGTAAGTCAGCCATTATCAAGAACCTAGTTACCAGGCAAGACCTGGTGACTAGCGGAAAGAAAGAAAACTGCCAAGAAATCAGCACCGACGTGATGAGACAGAGAGGTCTAGAAATATCTGCACGTACGGTTGATTCGCTGCTCTTGAATGGATGCAATAGACCAGTCGACGTGTTGTACGTAGACGAGGCGTTTGCGTGCCACTCTGGAACGTTACTTGCCTTGATCGCCTTGGTGAGACCAAGACTGAAAGTTGTACTTTGTGGTGACCCGAAGCAGTGCGGCTTCTTCAATATGATGCAGATGAAAGTCAACTACAATCATAACATCTGCACTCAAGTGTACCACAAAAGTATTTCCAGGCGGTGTACACTGCCTGTGACTGCCATTGTGTCGTCGTTGCATTACGAAGGCAAAATGCGCACTACGAATGAGTACAACATGCCGATTGTAGTGGACACTACGGGCTCAACAAAACCTGACCCTGGAGACCTCGTGTTAACGTGCTTCAGAGGGTGGGTTAAACAACTGCAAATTGACTATCGTGGACACGAGGTCATGACAGCAGCCGCATCCCAAGGGTTAACCAGAAAAGGAGTTTACGCAGTTAGGCAAAAAGTTAACGAAAACCCACTCTATGCATCAACATCAGAGCACGTCAACGTACTCCTAACGCGTACGGAAGGTAAACTGGTATGGAAGACACTCTCTGGTGACCCGTGGATAAAGACGCTGCAGAACCCACCGAAAGGAAACTTCAAGGCAACTATTAAGGAGTGGGAGGTGGAGCACGCATCGATAATGGCGGGCATCTGCAGTCACCAAGTGACCTTTGACACGTTCCAAAACAAAGCCAACGTTTGCTGGGCTAAGAGCTTGGTCCCTATCCTCGAAACAGCGGGGATTAAACTAAATGATAGGCAGTGGTCCCAGATAATTCAAGCTTTCAAAGAAGACAAAGCATACTCACCCGAAGTAGCCCTGAATGAAATATGCACGCGCATGTATGGGGTGGATCTAGACAGCGGGCTATTCTCTAAACCGTTGGTATCTGTGTATTACGCGGATAACCACTGGGATAATAGGCCGGGAGGAAAGATGTTCGGATTCAACCCTGAGGCAGCGTCCATTCTAGAAAGAAAGTACCCGTTTACAAAAGGAAAGTGGAACATCAACAAGCAGATCTGCGTGACTACCAGGAGGATAGAAGACTTCAACCCTACCACCAACATTATACCGGCCAACAGGAGACTACCACACTCATTAGTGGCCGAACACCGCCCAGTAAAAGGGGAAAGAATGGAATGGCTGGTTAACAAGATAAACGGACATCATGTGCTCCTGGTTAGCGGCTATAACCTTGCACTGCCTACTAAGAGAGTCACCTGGGTAGCGCCACTAGGTGTCCGCGGAGCGGACTATACATACAACCTAGAGCTGGGTCTACCGGCAACACTTGGTAGGTATGACCTAGTGGTCATAAACATCCACACACCTTTTCGCATACACCATTACCAACAGTGCGTAGATCACGCAATGAAACTGCAAATGCTAGGAGGTGACTCACTGAGACTGCTCAAACCGGGTGGCTCTCTATTGATCAGAGCATACGGTTACGCAGATAGAACCAGTGAACGAGTAATCTGCGTACTGGGACGTAAGTTTAGATCGTCCAGAGCATTGAAACCACCATGTATCACCAGTAATACTGAGATGTTCTTCCTATTTAGCAGTTTTGACAATGGCAGAAGGAATTTTACAACGCATGTTATGAACAATCAACTGAACGCAGCCTTTGTAGGACAGGCCACCCGAGCAGGATGTGCACCATCGTACCGGGTAAAGCGCATGGACATCGCGAAGAACGATGAAGAGTGCGTGGTCAACGCCGCCAACCCTCGTGGGTTACCGGGTGACGGTGTTTGCAAGGCAGTATATAAAAAATGGCCGGAGTCCTTTAAAAATAGTGCAACACCAGTAGGAACCGCAAAAACAGTTATGTGCGGTACATATCCAGTAATCCATGCCGTAGGACCAAACTTCTCAAATTACACGGAGTCCGAAGGGGACCGGGAATTGGCGGCTGCCTATCGAGAAGTCGCAAAGGAAGTAACTAGACTGGGAGTAAATAGCGTAGCTATACCTCTCCTCTCCACAGGTGTATACTCAGGAGGGAAAGACAGGCTAACCCAGTCACTGAACCACCTCTTTACAGCCATGGACTCGACGGATGCAGACGTGGTCATCTACTGCCGAGACAAGGAATGGGAGAAGAAAATATCTGAGGCCATACAGATGCGGACCCAAGTGGAGCTGCTGGATGAGCACATCTCCATAGACTGCGATGTCATTCGCGTGCACCCTGACAGTAGTTTGGCAGGTAGAAAAGGATACAGCACCACGGAAGGCGCACTGTATTCATATCTAGAAGGGACACGTTTTCACCAGACGGCAGTGGATGTGGCAGAGATACATACTATGTGGCCAAAGCAAATAGAGGCCAATGAGCAAGTCTGCCTATATGCCCTGGGGGAAAGTATTGAGTCAATCAGGCAGAAATGCCCGGTGGATGATGCAGATGCATCATCTCCCCCGAAAACCGTCCCGTGCCTTTGCCGTTATGCCATGACTCCTGAACGCGTCACCCGACTTCGCATGAACCATGTCACAAACATAATTGTGTGTTCTTCATTTCCCCTTCCAAAGTACAAGATAGAAGGAGTGCAAAAAGTCAAATGCTCCAAGGTAATGTTATTTGATCACAATGTGCCATCGCGCGTAAGTCCAAGGGAATACAGATCTTCCCAGGAGTCTGTACGGGAAGTGAGTATGACAACGTCATTGACGCATAGTCAGTTTGATCTAAGCGCCGATGGCGAGACACTGCCCGTCCCGTCAGACCTGGATGCTGACGCCCCAGCCCTAGAACCGGCCCTAGACGACGGGGCGATACATACGACCGGAAACCTTGCGGCCGTGTCTGACTGGGTAATGAGCACCGTACCCGTCGCGCCGCCTAGAAGAAGGAGAGGGAGAAACCTGACCGTGATATGTGACGAGAGAGAAGGGAATATAACACCCATGGCTAGCGTCCGATTCTTTAGAGCAGAGCAGTGTCCGACCGTACAAGAAACAGCGGAGACGCGTGACACAGCTATTTCCTTTCGGGCACCGCCAAGTATCACCGTGGAACTGAGCCATCCACCGATCTCCTTCGGAGCACCAAGCGAGACGTTCCCCATCACATTTGGGGACTTCAACGATGGAGAAATCGAAAGCTTGTCTTCTGAGCTACTAACTTTCGGAGACTTCCTACCCGGTGAAGTGGATGATTTGACAGATAGCGACTGGTCCACGTGCTCAGACACGGACGACGAGTTATGACTAGATAGGGCAGGTGGGTATATATTCTCGTCGGACACTGGTCCAGGCCATTTACAACAGAAGTCGGTACGCCAGTCAGTGCTGCCGGTAAACACCCTGGAGGAGGTTCACGAGGAGAAGTGTTACCCACCTAAGCTGGATGAATTAAAGGAGCAACTACTACTTAAGAAACTCCAGGAGAGTGCGTCCACGGCCAATAGAAGCAGGTATCAATCACGCAAAGTGGAAAATATGAAAGCAACAATCATCCAGAGACTAAAGAGAGGCTGTAAACTGTATTTAATGGCAGAGACCCCGAAAGTCCCGACTTATCGGACCGTATACCCGGCGCCTGTGTACTCGCCTCCGATCAACGTCCGATTGTCCAATCCCGAGTCCGCAGTGGCAGCATGTAATGAGTTCTTAGCTAGAAACTACCCAACTGTTTCATCATACCAAATCACCGATGAGTATGATGCATATCTAGACATGGTGGACGGGTCGGAGAGTTGCTTGGACCGAGCGACATTCAATCCGTCAAAACTTAGGAGCTACCCGAAACAACATGCTTATCACGCGCCCTCTATCAGAAGCGCTGTACCTTCCCCATTCCAGAACACACTACAGAATGTACTGGCAGCAGCCACGAAAAGGAACTGCAACGTCACACAGATGAGGGAATTACCCACTTTGGACTCAGCAGTATTCAACGTGGAGTGTTTTAAAAAATTCGCATGCAACCGAGAATACTGGGAAGAATTTGCTGCCAGCCCTATCAGGATAACGACTGAGAATCTAACAACCTATGTCACTAAATTAAAGGGGCCAAAAGCAGCAGCGTTGCTTGCAAGAACCCATAATCTGCTGCCGCTGCAGGATGTACCAATGGATAGGTTCACAGTAGATATGAAAAGGGACGTGAAGGTAACTCCTGGCACAAAGCATACAGAGGAAAGGCCTAAGGTGCAGGTTATACAGGCGGCTGAACCCTTGGCAACAGCGTACCTATGTGGAATTCACAGAGAATTGGTTAGGAGATTGAACGCCGTCCTCCTACCCAACGTGCATACACTATTTGACATGTCTGCCGAGGACTTCGATGCCATTATAGCCGCACACTTTAAGCCAGGAGACGCCGTTTTAGAAACGGACATAGCCTCCTTTGATAAGAGCCAGGATGATTCACTTGCGCTTACCGCCTTAATGCTGTTAGAAGATTTGGGAGTGGATCACTCCTTGTTGGACCTGATAGAGGCTGCTTTTGGAGAGATTTCCAGCTGTCACCTGCCGACAGGTACGCGCTTCAAGTTCGGCGCTATGATGAAATCCGGTATGTTCCTAACTCTGTTCGTCAACACATTGTTAAATATCACCATCGCTAGCCGGGTGTTGGAAGATCGTCTGACAAAATCTGCATGCGCGGCCTTCATCGGCGACGACAACATAATACATGGTGTCGTCTCCGATGAATTGATGGCAGCCAGATGCGCTACTTGGATGAACATGGAAGTGAAGATCATAGATGCAGTTGTATCCCAGAAAGCTCCCTACTTTTGTGGAGGGTTTATACTGCATGATACTGTGACAGGAACAGCTTGCAGGGTGGCGGACCCGCTAAAAAGGTTATTTAAACTGGGCAAACCGTTAGCGGCAGGTGACGAACAAGACGAAGACAGAAGGCGGGCGCTGGCTGATGAAGTAATCAGATGGCAACGAACAGGGCTAATAGATGAGCTGGAGAAAGCGGTGTACTCTAGGTACGAAGTGCAGGGTATATCAGTTGCTGTAATGTCCATGGCCACCTTCGCAAGCTCCAGATCCAACTTCGAGAAGCTCAGAGGACCCGTCATAACCTTGTACGGCGGTCCTAAATAGGTACGCACTACAGCTACCTATTTTGCAAAAGCCGACAGCAGGTACCTAAATACCAATCAGCCATAATGGAGTTTATCCCAACCCAAACTTTCTACAATAGGAGGTACCAGCCTCGACCTTGGACTCCGCGCCCTACTATCCAAGTCATCAGACCCAGACCGCGTCCGCAAAGGAAGGCCGGGCAACTTGCCCAGCTGATCTCAGCAGTTAATAAACTGACAATGCGCGTGGTACCTCAACAGAAACCGCGCAAGAATCGGAAGAATAAGAAGCAAAAGCAAAAGCAGCAGGCGCCACGAAACAATACGAATCAAAAGAAGCAGCCCCCTAAAAAGAAACCGGTTCAAAAGAAAAAGAAGCCGGGCCGCAGAGAGAGAATGTGCATGAAAATCGAAAATGATTGCATCTTCGAAGTCAAGCATGAAGGTAAGGTAACAGGTTACGCGTGCTTGGTAGGGGACAAAGTAATGAAGCCAGCACACGTAAAGGGGACCATCGATAACGCGGACCTGGCCAAATTGGCCTTCAAGCGGTCATCTAAGTACGACCTTGAATGCGCGCAGATACCCGTGCACATGAAGTCCGACGCTTCGAAGTTCACCCATGAGAAACCGGAGGGGTACTACAACTGGCACCACGGAGCAGTACAGTACTCAGGAGGCCGATTCACCATCCCTACAGGTGCGGGCAAACCAGGGGATAGTGGTAGACCGATCTTCGACAACAAGGGGCGCGTGGTGGCCATAGTTTTAGGAGGAGCTAATGAAGGAGCCCGTACAGCCCTCTCGGTGGTGACCTGGAACAAAGACATCGTCACGAAAATCACCCCTGAGGGGGCCGAAGAGTGGAGTCTGGCCATTCCAGTTATGTGCCTGCTGGCAAATACCACGTTCCCCTGCTCCCGGCCCCCTTGCACACCCTGCTGCTACGAAAAAGAGCCGGAGAAAACCTTGCGCATGCTTGAAGACAATGTCATGAGCCCCGGGTACTATCAGCTGCTACAAGCATCCTTAACATGTTCTCCCCGACGCCAGCGGCGCAGTATTAAGGACCACTTCAATGTCTATAAAGCCACAAGACCGTACCTAGCTCACTGTCCCGACTGTGGAGAAGGGCACTCGTGCCATAGTCCCGTAGCGCTAGAACGCATCAGAAACGAAGCGACAGACGGGACGTTGAAAATCCAGGTTTCCTTGCAAATCGGAATAAAGACGGATGATAGCCATGATTGGACCAAGCTGCGTTATATGGACAATCACATGCCAGCAGACGCAGAGCGGGCCGGGCTATTTGTAAGAACGTCAGCACCGTGCACGATTACTGGAACAATGGGACACTTCATTCTGGCCCGATGTCCGAAAGGAGAAACTCTGACGGTGGGGTTCACTGACGGTAGGAAGATCAGTCACTCATGTACGCACCCATTTCACCATGACCCTCCTGTGATAGGCCGGGAAAAATTCCATTCCCGACCGCAGCACGGTAGGGAACTACCTTGCAGCACGTACGCGCAGAGCACCGCTGCAACTGCCGAGGAGATAGAGGTACATATGCCCCCAGACACCCCAGATCGCACATTAATGTCACAACAGTCCGGCAATGTAAAGATCACAGTCAATAGTCAGACGGTGCGGTACAAGTGCAATTGTGGTGACTCAAGTGAAGGATTAACCACTACAGATAAAGTGATTAATAACTGCAAGGTCGATCAATGCCATGCCGCGGTCACCAATCACAAAAAATGGCAGTATAATTCCCCTCTGGTCCCGCGTAATGCTGAATTCGGGGACCGGAAAGGAAAAGTTCACATTCCATTTCCTCTGGCAAATGTGACATGCAGGGTGCCTAAAGCAAGAAACCCCACCGTGACGTACGGAAAAAACCAAGTCATCATGTTGCTGTATCCTGACCACCCAACGCTCCTGTCCTACAGGAATATGGGAGAAGAACCAAACTATCAAGAAGAGTGGGTGACGCTTAAGAAGGAGATCAGGTTAACCGTGCCGACTGAAGGGCTCGAGGTCACGTGGGGTAACAATGAGCCGTACAAGTATTGGCCGCAGTTATCCACAAACGGTACAGCCCACGGCCACCCGCATGAGATAATTCTGTATTATTATGAGCTGTACCCAACTATGACTGTGGTAGTTTTGTCAGTGGCCTCGTTCATACTCCTGTCGATGGTGGGTGTGGCAGTGGGGATGTGCATGTGTGCACGACGCAGATGCATTACACCGTACGAACTGACACCAGGAGCTACCGTCCCTTTCCTGCTTAGCCTAATATGCTGCATTAGAACAGCTAAAGCGGCCACATACCAGGAGGCCGCGGTATACCTGTGGAACGAGCAGCAGCCTTTATTTTGGCTGCAAGCCCTTATTCCGCTGGCAGCCCTGATTGTCCTATGTAACTGTCTGAGACTCTTACCATGCTGTTGTAAAATGTTGACTTTTTTAGCCGTACTGAGCGTCGGTGCCCACACTGTGAGCGCGTACGAACACGTAACAGTGATCCCGAACACGGTGGGAGTACCGTATAAGACTCTAGTCAACAGACCGGGCTACAGCCCCATGGTATTGGAGATGGAGCTTCTGTCTGTCACCTTGGAACCAACGCTATCGCTTGATTACATCACGTGCGAGTATAAAACCGTTATCCCGTCTCCGTACGTGAAATGCTGCGGTACAGCAGAGTGTAAGGACAAGAGCCTACCTGATTACAGCTGTAAGGTCTTCACCGGCGTCTACCCATTCATGTGGGGCGGCGCCTACTGCTTCTGCGACACCGAAAATACGCAATTGAGCGAAGCACATGTGGAGAAGTCCGAATCATGCAAAACAGAATTTGCATCAGCATACAGGGCTCATACCGCATCCGCATCAGCTAAGCTCCGCGTCCTTTACCAAGGAAATAATATCACTGTAGCTGCTTATGCAAACGGCGACCATGCCGTCACAGTTAAGGACGCTAAATTCATAGTGGGGCCAATGTCTTCAGCCTGGACACCTTTCGACAATAAAATCGTGGTGTACAAAGGCGACGTCTACAACATGGACTACCCGCCCTTCGGCGCAGGAAGACCAGGACAATTTGGCGACATCCAAAGTCGCACGCCTGAGAGCGAAGACGTCTATGCTAATACACAACTGGTACTGCAGAGACCGTCCGCGGGTACGGTGCACGTGCCGTACTCTCAGGCACCATCTGGCTTCAAGTATTGGCTAAAAGAACGAGGGGCGTCGCTGCAGCACACAGCACCATTTGGCTGTCAAATAGCAACAAACCCGGTAAGAGCGATGAACTGCGCCGTAGGGAACATGCCTATCTCCATCGACATACCGGACGCGGCCTTTACCAGGGTCGTCGACGCGCCATCTTTAACGGACATGTCGTGTGAGGTATCAGCCTGCACCCATTCCTCAGACTTTGGGGGCGTAGCCATCATTAAATATGCAGCCAGTAAGAAAGGCAAGTGTGCAGTGCACTCGATGACTAACGCCGTCACTATTCGGGAAGCTGAAATAGAAGTAGAAGGGAACTCTCAGTTGCAAATCTCTTTTTCGACGGCCCTAGCCAGCGCCGAATTTCGCGTACAAGTCTGTTCTACACAAGTACACTGTGCAGCCGAGTGCCATCCACCGAAAGACCATATAGTCAATTACCCGGCGTCACACACCACCCTCGGGGTCCAAGACATTTCCGCTACGGCGATGTCATGGGTGCAGAAGATCACGGGAGGTGTGGGACTGGTTGTCGCTGTTGCAGCACTGATCCTAATCGTGGTGCTATGCGTGTCGTTTAGCAGGCACTAACTTGACAACTAGGTATGAAGGCATACGCGTCCCTAAAGAGACACACCGCATATAGCTAGGAATCAACAGATAAGTATAGATCTAAGGGCTGAACAACCCCTGAATAGTAACAAAATATAAAAATCAACAAAAATCATAAAATAGAAAACTAGAAATAGAAGTAGGTAAGAAGGTATATGTGTCCCCTAAGAGACACACCATATATAGCTAAGAATCAATAGATAAGCATAGATCAAAGGGCTGAACAACCCCTGAATAATAACAAAATATAAAAACCAATAAAAATCATAAAATAGAAAACCACAAATAGAAGTAGTTCAAAGGGCTATAAAACCCCTGAATAGTAACAAAATATAAAACTAATAAAAATCAAACGAATACCATAATTGGCAATCGGAAGAGATGTAGGTACTTAAGCTTCTTAAAAGCAGCCGAACTCGCTTTGAGATGTAGGCGTAGCACACCGAACTCTTCCATAATTCTCCGAACCCACAGGGACGTAGGAGATGTTCAAAGTGACTATAAAACCCTGAACAGTAATAAAATATAAAATTAATAATGAGTACCATAATTGGCAAATGGAAGAGACGTAGGTACTAAGCTTCTTAAAAGCAGCCGAACTCACTTTGAGATGTAGGCATAGCATACCGAACTCTTCCACAATTCTCCGTACCCATAGGGACGTAGGAGATGTTATTTTGTTTTTAATATTT

>KJ451623|2013|Micronesia: Yap State, Group 1

ATGGCTGCGTGAGACACACGTAGCCTACTAGTTTCTTACTGCTCTACTCTGCAAAGCAAGAGATTAATAACCCATCATGGATTCTGTGTACGTGGATATAGACGCTGACAGCGCCTTTTTGAAGGCCCTGCAACGTGCGTACCCCATGTTTGAGGTGGAACCTAGGCAGGTCACATCAAATGACCATGCTAATGCTAGAGCGTTCTCGCATCTAGCCATAAAACTAATAGAGCAGGAAATTGATCCCGACTCAACCATCCTGGATATAGGTAGTGCGCCAGCAAGGAGGATGATGTCGGACAGGAAGTACCACTGCGTTTGCCCGATGCGCAGCGCAGAAGATCCCGAGAGACTCGCTAATTATGCGAGAAAGCTCGCATCTGCCGCAGGAAAAGTCCTGGACAGAAACATTTCTGGAAAGATCGGGGACTTACAAGAGGTGATGGCCGTGCCAGACACGGAGACGCCAACATTTTGCTTACACACAGATGTCTCATGTAGACAGAGAGCAGACGTCGCGATATACCAAGACGTCTATGCTGTACATGCACCCACGTCGCTATATCACCAGGCGATTAAAGGAGTCCGAGTGGCGTACTGGGTAGGGTTCGACACAACCCCGTTCATGTACAACGCTATGGCGGGTGCCTACCCCTCATACTCGACAAATTGGGCGGATGAGCAGGTACTGAAGGCTAAGAACATAGGATTATGTTCAACAGACCTGACAGAAGGTAGACGAGGCAAATTGTCTATCATGAGAGGGAAAAAGCTAAAACCGTGCGACCGTGTGCTGTTCTCAGTAGGGTCAACGCTTTACCCGGAAAGCCGCATGCTACTTAAGAGCTGGCACCTACCATCGGTGTTCCATCTAAAGGGCAAGCTTAGCTTCACATGCCGCTGTGACACAGTGGTTTCGTGTGAGGGCTACGTCGTTAAGAGAATAACGATGAGCCCAGGCCTTTATGGAAAAACCACGGGGTATGCGGTAACCCACCACGCAGACGGATTCTTGATGTGCAAGACTACCGACACGGTAGACGGTGAAAGAGTGTCATTCTCGGTGTGCACGTACGTGCCGGCGACCATTTGTGATCAAATGACCGGCATCCTTGCTACAGAAGTCACGCCGGAGGATGCACAGAAGCTGTTGGTGGGGCTGAACCAGAGAATAGTGGTTAACGGCAGAACGCAACGGAACACGAACACCATGAAGAACTACCTGCTTCCCGTGGTCGCCCAGGCCTTCAGTAAGTGGGCGAAGGAGTGCCGGAAGGACATGGAAGATGAGAAGCTTCTGGGGGTCAGAGAAAGAACACTGACCTGCTGCTGTCTGTGGGCATTTAAGAAGCAGAAAACACACACGGTCTACAAGAGGCCTGATACCCAGTCAATCCAGAAGGTTCAGGCCGAATTCGACAGCTTTGTAGTACCAGGCCTGTGGTCGTCCGGGTTGTCAATCCCGTTGAGGACTAGAATCAAGTGGCTGTTACGCAAGGTGCCGAAAGCAGACCTGATCCCATACAGCGGAAATGCCCAAGAAGCCCAGGATGCTGAAAAAGAAGCAGAGGAAGAACGAGAAGCAGAACTGACTCATGAGGCTCTACCACCCCTACAGGCAGCACAGGAAGATGTCCAGGTCGAAATCGACGTGGAACAACTTGAGGATAGAGCTGGTGCTGGAATAATAGAGACTCCGAGAGGCGCTATTAAAGTTACTGCCCAACTAACGGACCACGTCGTGGGGGAGTACCTGGTACTTTCCCCGCAGACCGTATTACGCAGCCAGAAGCTCAGCCTGATCCACGCTTTAGCGGAGCAAGTGAAGACGTGTACGCATAGCGGACGAGCAGGGAGGTATGCGGTCGAAGCGTACGATGGCCGAGTCCTAGTGCCCTCAGGCTATGCAATTTCGCCCGAAGACTTCCAGAGTCTAAGCGAAAGCGCAACGATGGTGTACAACGAAAGAGAGTTCGTAAACAGAAAGTTACACCACATTGCGATGCACGGACCAGCTCTGAACACTGACGAAGAGTCGTATGAGCTTGTGAGGGCAGAGAGGACAGAACACGAGTACGTCTACGACGTGGACCAGAGAAGATGCTGCAAGAAGGAAGAAGCTGCAGGATTGGTACTGGTGGGCGACTTGACTAATCCGCCCTACCACGAATTTGCATACGAAGGGCTAAAAATTCGCCCCGCTTGTCCATATAAAATTGCAGTCATAGGAGTCTTCGGGGTACCAGGATCTGGTAAGTCAGCCATTATCAAGAACCTAGTTACCAGGCAAGACCTGGTGACTAGCGGAAAGAAAGAAAACTGCCAAGAAATCAGCACCGACGTGATGAGACAGAGAGGTCTAGAAATATCTGCACGTACGGTTGATTCGCTGCTCTTGAATGGATGCAATAGACCAGTCGACGTGTTGTACGTAGACGAGGCGTTTGCGTGCCACTCTGGAACGTTACTTGCCTTGATCGCCTTGGTGAGACCAAGACTGAAAGTTGTACTTTGTGGTGACCCGAAGCAGTGCGGCTTCTTCAATATGATGCAGATGAAAGTCAACTACAATCATAACATCTGCACTCAAGTGTACCACAAAAGTATTTCCAGGCGGTGTACACTGCCTGTGACTGCCATTGTGTCGTCGTTGCATTACGAAGGCAAAATGCGCACTACGAATGAGTACAACATGCCGATTGTAGTGGACACTACGGGCTCAACAAAACCTGACCCTGGAGACCTCGTGTTAACGTGCTTCAGAGGGTGGGTTAAACAACTGCAAATTGACTATCGTGGACACGAGGTCATGACAGCAGCCGCATCCCAAGGGTTAACCAGAAAAGGAGTTTACGCAGTTAGGCAAAAAGTTAACGAAAACCCACTCTATGCATCAACATCAGAGCACGTCAACGTACTCCTAACGCGTACGGAAGGTAAACTGGTATGGAAGACACTCTCTGGTGACCCGTGGATAAAGACGCTGCAGAACCCACCGAAAGGAAACTTCAAGGCAACTATTAAGGAGTGGGAGGTGGAGCACGCATCGATAATGGCGGGCATCTGCAGTCACCAAGTGACCTTTGACACGTTCCAAAACAAAGCCAACGTTTGCTGGGCTAAGAGCTTGGTCCCTATCCTCGAAACAGCGGGGATTAAACTAAATGATAGGCAGTGGTCCCAGATAATTCAAGCTTTCAAAGAAGACAAAGCATACTCACCCGAAGTAGCCCTGAATGAAATATGCACGCGCATGTATGGGGTGGATCTAGACAGCGGGCTATTCTCTAAACCGTTGGTATCTGTGTATTACGCGGATAACCACTGGGATAATAGGCCGGGAGGAAAGATGTTCGGATTCAACCCTGAGGCAGCGTCCATTCTAGAAAGAAAGTACCCGTTTACAAAAGGAAAGTGGAACATCAACAAGCAGATCTGCGTGACTACCAGGAGGATAGAAGACTTCAACCCTACCACCAACATTATACCGGCCAACAGGAGACTACCACACTCATTAGTGGCCGAACACCGCCCAGTAAAAGGGGAAAGAATGGAATGGCTGGTTAACAAGATAAACGGACATCATGTGCTCCTGGTTAGCGGCTATAACCTTGCACTGCCTACTAAGAGAGTCACCTGGGTAGCGCCACTAGGTGTCCGCGGAGCGGACTATACATACAACCTAGAGCTGGGTCTACCGGCAACACTTGGTAGGTATGACCTAGTGGTCATAAACATCCACACACCTTTTCGCATACACCATTACCAACAGTGCGTAGATCACGCAATGAAACTGCAAATGCTAGGAGGTGACTCACTGAGACTGCTCAAACCGGGTGGCTCTCTATTGATCAGAGCATACGGTTACGCAGATAGAACCAGTGAACGAGTAATCTGCGTACTGGGACGTAAGTTTAGATCGTCCAGAGCATTGAAACCACCATGTATCACCAGTAATACTGAGATGTTCTTCCTATTTAGCAGTTTTGACAATGGCAGAAGGAATTTTACAACGCATGTTATGAACAATCAACTGAACGCAGCCTTTGTAGGACAGGCCACCCGAGCAGGATGTGCACCATCGTACCGGGTAAAGCGCATGGACATCGCGAAGAACGATGAAGAGTGCGTGGTCAACGCCGCCAACCCTCGTGGGTTACCGGGTGACGGTGTTTGCAAGGCAGTATATAAAAAATGGCCGGAGTCCTTTAAAAATAGTGCAACACCAGTAGGAACCGCAAAAACAGTTATGTGCGGTACATATCCAGTAATCCATGCCGTAGGACCAAACTTCTCAAATTACACGGAGTCCGAAGGGGACCGGGAATTGGCGGCTGCCTATCGAGAAGTCGCAAAGGAAGTAATTAGACTGGGAGTAAATAGCGTAGCTATACCTCTCCTCTCCACAGGTGTATACTCAGGAGGGAAAGACAGGCTAACCCAGTCACTGAACCACCTCTTTACAGCCATGGACTCGACGGATGCAGACGTGGTCATCTACTGCCGAGACAAGGAATGGGAGAAGAAAATATCTGAGGCTATACAGATGCGGACCCAAGTGGAGCTGCTGGATGAGCACATCTCCATAGACTGCGATGTCATTCGCGTGCACCCTGACAGTAGTTTGGCAGGTAGAAAAGGATACAGCACCACGGAAGGCGCACTGTATTCATATCTAGAAGGGACACGTTTTCACCAGACGGCAGTGGATGTGGCAGAGATACATACTATGTGGCCAAAGCAAATAGAGGCCAATGAGCAAGTCTGCCTATATGCCCTGGGGGAAAGTATTGAGTCAATCAGGCAGAAATGCCCGGTGGATGATGCAGATGCATCATCTCCCCCGAAAACCGTCCCGTGCCTTTGCCGTTATGCCATGACTCCTGAACGCGTCACCCGACTTCGCATGAACCATGTCACAAACATAATTGTGTGTTCTTCATTTCCCCTTCCAAAGTACAAGATAGAAGGAGTGCAAAAAGTCAAATGCTCCAAGGTAATGTTATTTGATCACAATGTGCCATCGCGCGTAAGTCCAAGGGAATACAGATCTTCCCAGGAGTCTGTACGGGAAGTGAGTATGACAACGTCATTGACGCATAGTCAGTTTGATCTAAGCGCCGATGGCGAGACACTGCCCGTCCCGTCAGACCTGGATGCTGACGCCCCAGCCCTAGAACCGGCCCTAGACGACGGGGCGATACATACGACCGGAAACCTTGCGGCCGTGTCTGACTGGGTAATGAGCACCGTACCCGTCGCGCCGCCTAGAAGAAGGAGAGGGAGAAACCTGACCGTGATATGTGACGAGAGAGAAGGGAATATAACACCCATGGCTAGCGTCCGATTCTTTAGAGCAGAGCAGTGTCCGACCGTACAAGAAACAGCGGAGACGCGTGACACAGCTATTTCCTTTCGGGCACCGCCAAGTATCACCGTGGAACTGAGCCATCCACCGATCTCCTTCGGAGCACCAAGCGAGACGTTCCCCATCACATTTGGGGACTTCAACGATGGAGAAATCGAAAGCTTGTCTTCTGAGCTACTAACTTTCGGAGACTTCCTACCCGGTGAAGTGGATGATTTGACAGATAGCGACTGGTCCACGTGCTCAGACACGGACGACGAGTTATGACTAGATAGGGCAGGTGGGTATATATTCTCGTCGGACACTGGTCCAGGCCATTTACAACAGAAGTCGGTACGCCAGTCAGTGCTGCCGGTAAACACCCTGGAGGAGGTTCACGAGGAGAAGTGTTACCCACCTAAGCTGGATGAATTAAAGGAGCAACTACTACTTAAGAAACTCCAGGAGAGTGCGTCCACGGCCAATAGAAGCAGGTATCAATCACGCAAAGTGGAAAATATGAAAGCAACAATCATCCAGAGACTAAAGAGAGGCTGTAAACTGTATTTAATGGCAGAGACCCCGAAAGTCCCGACTTATCGGACCGTATACCCGGCGCCTGTGTACTCGCCTCCGATCAACGTCCGATTGTCCAATCCCGAGTCCGCAGTGGCAGCATGTAATGAGTTCTTAGCTAGAAACTACCCAACTGTTTCATCATACCAAATCACCGATGAGTATGATGCATATCTAGACATGGTGGACGGGTCGGAGAGTTGCTTGGACCGAGCGACATTCAATCCGTCAAAACTTAGGAGCTACCCGAAACAACATGCTTATCACGCGCCCTCTATCAGAAGCGCTGTACCTTCCCCATTCCAGAACACACTACAGAATGTACTGGCAGCAGCCACGAAAAGGAACTGCAACGTCACACAGATGAGGGAATTACCCACTTTGGACTCAGCAGTATTCAACGTGGAGTGTTTTAAAAAATTCGCATGCAACCGAGAATACTGGGAAGAATTTGCTGCCAGCCCTATCAGGATAACGACTGAGAATCTAACAACCTATGTCACTAAATTAAAGGGGCCAAAAGCAGCAGCGTTGCTTGCAAGAACCCATAATCTGCTGCCGCTGCAGGATGTACCAATGGATAGGTTCACAGTAGATATGAAAAGGGACGTGAAGGTAACTCCTGGCACAAAGCATACAGAGGAAAGGCCTAAGGTGCAGGTTATACAGGCGGCTGAACCCTTGGCAACAGCGTACCTATGTGGAATTCACAGAGAATTGGTTAGGAGATTGAACGCCGTCCTCCTACCCAACGTGCATACACTATTTGACATGTCTGCCGAGGACTTCGATGCCATTATAGCCGCACACTTTAAGCCAGGAGACGCCGTTTTAGAAACGGACATAGCCTCCTTTGATAAGAGCCAGGATGATTCACTTGCGCTTACCGCCTTAATGCTGTTAGAAGATTTGGGAGTGGATCACTCCTTGTTGGACCTGATAGAGGCTGCTTTTGGAGAGATTTCCAGCTGTCACCTGCCGACAGGTACGCGCTTCAAGTTCGGCGCTATGATGAAATCCGGTATGTTCCTAACTCTGTTCGTCAACACATTGTTAAATATCACCATCGCTAGCCGGGTGTTGGAAGATCGTCTGACAAAATCTGCATGCGCGGCCTTCATCGGCGACGACAACATAATACATGGTGTCGTCTCCGATGAATTGATGGCAGCCAGATGCGCTACTTGGATGAACATGGAAGTGAAGATCATAGATGCAGTTGTATCCCAGAAAGCTCCCTACTTTTGTGGAGGGTTTATACTGCATGATACTGTGACAGGAACAGCTTGCAGGGTGGCGGACCCGCTAAAAAGGTTATTTAAACTGGGCAAACCGTTAGCGGCAGGTGACGAACAAGACGAAGACAGAAGGCGGGCGCTGGCTGATGAAGTAATCAGATGGCAACGAACAGGGCTAATAGATGAGCTGGAGAAAGCGGTGTACTCTAGGTACGAAGTGCAGGGTATATCAGTTGCTGTAATGTCCATGGCCACCTTCGCAAGCTCCAGATCCAACTTCGAGAAGCTCAGAGGACCCGTCATAACCTTGTACGGCGGTCCTAAATAGGTACGCACTACAGCTACCTATTTTGCAAAAGCCGACAGCAGGTACCTAAATACCAATCAGCCATAATGGAGTTTATCCCAACCCAAACTTTCTACAATAGGAGGTACCAGCCTCGACCTTGGACTCCGCGCCCTACTATCCAAGTCATCAGACCCAGACCGCGTCCGCAAAGGAAGGCCGGGCAACTTGCCCAGCTGATCTCAGCAGTTAATAAACTGACAATGCGCGTGGTACCTCAACAGAAACCGCGCAAGAATCGGAAGAATAAGAAGCAAAAGCAAAAGCAGCAGGCGCCACGAAACAATACGAATCAAAAGAAGCAGCCCCCTAAAAAGAAACCGGTTCAAAAGAAAAAGAAGCCGGGCCGCAGAGAGAGAATGTGCATGAAAATCGAAAATGATTGCATCTTCGAAGTCAAGCATGAAGGTAAGGTAACAGGTTACGCGTGCTTGGTAGGGGACAAAGTAATGAAGCCAGCACACGTAAAGGGGACCATCGATAACGCGGACCTGGCCAAATTGGCCTTCAAGCGGTCATCTAAGTACGACCTTGAATGCGCGCAGATACCCGTGCACATGAAGTCCGACGCTTCGAAGTTCACCCATGAGAAACCGGAGGGGTACTACAACTGGCACCACGGAGCAGTACAGTACTCAGGAGGCCGATTCACCATCCCTACAGGTGCGGGCAAACCAGGGGATAGTGGTAGACCGATCTTCGACAACAAGGGGCGCGTGGTGGCCATAGTTTTAGGAGGAGCTAATGAAGGAGCCCGTACAGCCCTCTCGGTGGTGACCTGGAACAAAGACATCGTCACGAAAATCACCCCTGAGGGGGCCGAAGAGTGGAGTCTGGCCATTCCAGTTATGTGCCTGCTGGCAAATACCACGTTCCCCTGCTCCCGGCCCCCTTGCACACCCTGCTGCTACGAAAAAGAGCCGGAGAAAACCTTGCGCATGCTTGAAGACAATGTCATGAGCCCCGGGTACTATCAGCTGCTACAAGCATCCTTAACATGTTCTCCCCGACGCCAGCGGCGCAGTATTAAGGACCACTTCAATGTCTATAAAGCCACAAGACCGTACCTAGCTCACTGTCCCGACTGTGGAGAAGGGCACTCGTGCCATAGTCCCGTAGCGCTAGAACGCATCAGAAACGAAGCGACAGACGGGACGTTGAAAATCCAGGTTTCCTTGCAAATCGGAATAAAGACGGATGATAGCCATGATTGGACCAAGCTGCGTTATATGGACAATCACATGCCAGCAGACGCAGAGCGGGCCGGGCTATTTGTAAGAACGTCAGCACCGTGCACGATTACTGGAACAATGGGACACTTCATTCTGGCCCGATGTCCGAAAGGAGAAACTCTGACGGTGGGGTTCACTGACGGTAGGAAGATCAGTCACTCATGTACGCACCCATTTCACCATGACCCTCCTGTGATAGGCCGGGAAAAATTCCATTCCCGACCGCAGCACGGTAGGGAACTACCTTGCAGCACGTACGCGCAGAGCACCGCTGCAACTGCCGAGGAGATAGAGGTACATATGCCCCCAGACACCCCAGATCGCACATTAATGTCACAACAGTCCGGCAATGTAAAGATCACAGTCAATAGTCAGACGGTGCGGTACAAGTGCAATTGTGGTGACTCAAGTGAAGGATTAACCACTACAGATAAAGTGATTAATAACTGCAAGGTCGATCAATGCCATGCCGCGGTCACCAATCACAAAAAATGGCAGTATAATTCCCCTCTGGTCCCGCGTAATGCTGAATTCGGGGACCGGAAAGGAAAAGTTCACATTCCATTTCCTCTGGCAAATGTGACATGCAGGGTGCCTAAAGCAAGAAACCCCACCGTGACGTACGGAAAAAACCAAGTCATCATGTTGCTGTATCCTGACCACCCAACGCTCCTGTCCTACAGGAATATGGGAGAAGAACCAAACTATCAAGAAGAGTGGGTGACGCTTAAGAAGGAGATCAGGTTAACCGTGCCGACTGAAGGGCTCGAGGTCACGTGGGGTAACAATGAGCCGTACAAGTATTGGCCGCAGTTATCCACAAACGGTACAGCCCACGGCCACCCGCATGAGATAATTCTGTATTATTATGAGCTGTACCCAACTATGACTGTGGTAGTTTTGTCAGTGGCCTCGTTCATACTCCTGTCGATGGTGGGTGTGGCAGTGGGGATGTGCATGTGTGCACGACGCAGATGCATTACACCGTACGAACTGACACCAGGAGCTACCGTCCCTTTCCTGCTTAGCCTAATATGCTGCATTAGAACAGCTAAAGCGGCCACATACCAGGAGGCCGCGGTATACCTGTGGAACGAGCAGCAGCCTTTATTTTGGCTGCAAGCCCTTATTCCGCTGGCAGCCCTGATTGTCCTATGTAACTGTCTGAGACTCTTACCATGCTGTTGTAAAATGTTGACTTTTTTAGCCGTACTGAGCGTCGGTGCCCACACTGTGAGCGCGTACGAACACGTAACAGTGATCCCGAACACGGTGGGAGTACCGTATAAGACTCTAGTCAACAGACCGGGCTACAGCCCCATGGTATTGGAGATGGAGCTTCTGTCTGTCACCTTGGAACCAACGCTATCGCTTGATTACATCACGTGCGAGTATAAAACCGTTATCCCGTCTCCGTACGTGAAATGCTGCGGTACAGCAGAGTGTAAGGACAAGAGCCTACCTGATTACAGCTGTAAGGTCTTCACCGGCGTCTACCCATTCATGTGGGGCGGCGCCTACTGCTTCTGCGACACCGAAAATACGCAATTGAGCGAAGCACATGTGGAGAAGTCCGAATCATGCAAAACAGAATTTGCATCAGCATACAGGGCTCATACCGCATCCGCATCAGCTAAGCTCCGCGTCCTTTACCAAGGAAATAATATCACTGTAGCTGCTTATGCAAACGGCGACCATGCCGTCACAGTTAAGGACGCTAAATTCATAGTGGGGCCAATGTCTTCAGCCTGGACACCTTTCGACAATAAAATCGTGGTGTACAAAGGCGACGTCTACAACATGGACTACCCGCCCTTCGGCGCAGGAAGACCAGGACAATTTGGCGACATCCAAAGTCGCACGCCTGAGAGCGAAGACGTCTATGCTAATACACAACTGGTACTGCAGAGACCGTCCGCGGGTACGGTGCACGTGCCGTACTCTCAGGCACCATCTGGCTTCAAGTATTGGCTAAAAGAACGAGGGGCGTCGCTGCAGCACACAGCACCATTTGGCTGTCAAATAGCAACAAACCCGGTAAGAGCGATGAACTGCGCCGTAGGGAACATGCCTATCTCCATCGACATACCGGACGCGGCCTTTACCAGGGTCGTCGACGCGCCATCTTTAACGGACATGTCGTGTGAGGTATCAGCCTGCACCCATTCCTCAGACTTTGGGGGCGTAGCCATCATTAAATATGCAGCCAGTAAGAAAGGCAAGTGTGCAGTGCACTCGATGACTAACGCCGTCACTATTCGGGAAGCTGAAATAGAAGTAGAAGGGAACTCTCAGTTGCAAATCTCTTTTTCGACGGCCCTAGCCAGCGCCGAATTTCGCGTACAAGTCTGTTCTACACAAGTACACTGTGCAGCCGAGTGCCATCCACCGAAAGACCATATAGTCAATTACCCGGCGTCACACACCACCCTCGGGGTCCAAGACATTTCCGCTACGGCGATGTCATGGGTGCAGAAGATCACGGGAGGTGTGGGACTGGTTGTCGCTGTTGCAGCACTGATCCTAATCGTGGTGCTATGCGTGTCGTTTAGCAGGCACTAACTTGACAACTAGGTATGAAGGCATACGCGTCCCTAAAGAGACACACCGCATATAGCTAGGAATCAACAGATAAGTATAGATCTAAGGGCTGAACAACCCCTGAATAGTAACAAAATATAAAAATCAACAAAAATCATAAAATAGAAAACTAGAAATAGAAGTAGGTAAGAAGGTATATGTGTCCCCTAAGAGACACACCATATATAGCTAAGAATCAATAGATAAGCATAGATCAAAGGGCTGAACAACCCCTGAATAATAACAAAATATAAAAACCAATAAAAATCATAAAATAGAAAACCACAAATAGAAGTAGTTCAAAGGGCTATAAAACCCCTGAATAGTAACAAAATATAAAACTAATAAAAATCAAACGAATACCATAATTGGCAATCGGAAGAGATGTAGGTACTTAAGCTTCTTAAAAGCAGCCGAACTCGCTTTGAGATGTAGGCGTAGCACACCGAACTCTTCCATAATTCTCCGAACCCACAGGGACGTAGGAGATGTTCAAAGTGACTATAAAACCCTGAACAGTAATAAAATATAAAATTAATAATGAGTACCATAATTGGCAAATGGAAGAGACGTAGGTACTAAGCTTCTTAAAAGCAGCCGAACTCACTTTGAGATGTAGGCATAGCATACCGAACTCTTCCACAATTCTCCGTACCCATAGGGACGTAGGAGATGTTATTTTGTTTTTAATATTT

>HE806461|2011|New Caledonia, Group 1

ACGTAGCCTACCAGTTTCTTACTGCTCTACTCTGCAAAGCAAGAGATTAATAACCCATCATGGATTCTGTGTACGTGGATATAGACGCTGACAGCGCCTTTTTGAAGGCCCTGCAACGTGCGTACCCCATGTTTGAGGTGGAACCTAGGCAGGTCACATCAAATGACCATGCTAATGCTAGAGCGTTCTCGCATCTAGCCATAAAACTAATAGAGCAGGAAATTGATCCCGACTCAACCATCCTGGATATAGGTAGTGCGCCAGCAAGGAGGATGATGTCGGACAGGAAGTACCACTGCGTTTGCCCGATGCGCAGCGCAGAAGATCCCGAGAGACTCGCTAATTATGCGAGAAAGCTCGCATCTGCCGCAGGAAAAGTCCTGGACAGAAACATTTCTGGAAAGATCGGGGACTTACAAGCGGTGATGGCCGTGCCAGACACGGAGACGCCAACATTTTGCTTACACACAGATGTCTCATGTAGACAGAGAGCAGACGTCGCGATATACCAAGACGTCTATGCTGTACATGCACCCACGTCGCTATATCACCAGGCGATTAAAGGAGTCCGAGTGGCGTACTGGGTAGGGTTTGACACAACCCCGTTCATGTACAACGCTATGGCGGGTGCCTACCCCTCATACTCGACAAATTGGGCGGATGAGCAGGTACTGAAGGCTAAGAACATAGGATTATGTTCAACAGACCTGACGGAAGGTAGACGAGGCAAATTGTCTATCATGAGAGGGAAAAAGCTGAAACCGTGCGACCGTGTGCTGTTCTCAGTGGGGTCAACGCTTTACCCGGAAAGCCGCATGCTACTTAAGAGCTGGCACCTACCATCGGTGTTCCATCTAAAGGGCAAACTTAGCTTCACATGCCGCTGTGACACAGTGGTTTCGTGTGAGGGCTACGTCGTCAAGAGAATAACGATGAGCCCAGGCCTTTATGGAAAAACCACGGGGTATGCGGTAACCCACCACGCAGACGGATTCTTGATGTGCAAGACTACCGACACGGTTGACGGCGAAAGAGTGTCATTCTCGGTGTGCACGTACGTGCCGGCGACCATTTGTGATCAAATGACCGGCATCCTTGCTACAGAAGTCACGCCGGAGGATGCACAGAAGCTGTTGGTGGGGCTGAACCAGAGAATAGTGGTTAACGGCAGAACGCAACGGAACACGAACACCATGAAGAACTATCTACTTCCCGTGGTCGCCCAGGCCTTCAGTAAGTGGGCGAAGGAGTGCCGGAAGGACATGGAAGATGAGAAGCTTCTGGGGGTCAGAGAAAGAACACTAACCTGCTGCTGTCTGTGGGCATTTAAGAAGCAGAAAACACACACGGTCTACAAGAGGCCTGATACCCAGTCAATCCAGAAGGTTCAGGCCGAATTTGACAGCTTTGTAGTACCAGGCCTGTGGTCGTCCGGGTTGTCAATCCCGTTGAGGACTAGAATCAAGTGGCTGTTACGCAAGGTGCCGAAAGCAGACCTGATCCCATACAGCGGAAATGCCCAAGAAGCCCAGGATGCAGAAAAAGAAGCAGAGGAAGAACGAGAAGCAGAACTGACTCATGAGGCTCTACCACCCCTACAGGCAGCACAGGAAGATGTCCAGGTCGAAATCGACGTGGAACAACTTGAGGATAGAGCTGGTGCTGGAATAATAGAGACTCCGAGAGGCGCTATTAAAGTTACTGCCCAACTAACCGACCAAGTCGTGGGGGAGTACCTGGTACTTTCCCCGCAGACCGTATTACGCAGCCAGAAGCTCAGCCTGATCCACGCTTTAGCGGAGCAAGTGAAGACGTGTACGCATAGCGGACGAGCAGGGAGGTATGCGGTCGAAGCGTACGATGGCCGAGTCCTAGTGCCCTCAGGCTATGCAATTTCGCCTGAAGACTTCCAGAGTCTAAGCGAAAGCGCAACGATGGTGTACAACGAAAGAGAGTTCGTAAACAGAAAGTTACACCACATTGCGATGCACGGACCAGCCCTGAACACTGACGAAGAGTCGTATGAGCTTGTGAGGGCAGAGAGAACAGAACACGAGTACGTCTACGACGTGGACCAGAGAAGATGCTGTAAGAAGGAAGAAGCTGCAGGATTGGTACTGGTGGGCGACTTGACTAATCCGCCCTACCACGAATTTGCATACGAAGGGCTAAAAATTCGCCCCGCTTGTCCATACAAAATTGCAGTCATAGGAGTCTTCGGGGTACCAGGATCTGGTAAGTCAGCCATTATCAAGAACCTAGTTACCAGGCAAGACCTGGTGACTAGCGGAAAGAAAGAAAACTGCCAAGAAATCAGCACCGACGTGATGAGACAGAGAGGTCTAGAAATATCTGCACGTACGGTTGATTCGCTGCTCTTGAATGGATGCAATAGACCAGTCGACGTGTTGTACGTAGACGAGGCGTTTGCGTGCCACTCTGGAACGTTACTTGCCTTGATCGCCTTGGTGAGACCAAGACTGAAAGTTGTACTTTGTGGTGACCCGAAGCAGTGCGGCTTCTTCAATATGATGCAGATGAAAGTCAACTACAATCATAACATCTGCACTCAAGTGTACCACAAAAGTATCTCCAGGCGGTGTACACTGCCTGTGACTGCCATTGTGTCGTCGTTGCATTACGAAGGCAAAATGCGCACTACGAATGAGTACAACATGCCGATTGTAGTGGACACTACGGGCTCAACGAAACCTGACCCTGGAGACCTCGTGTTAACGTGCTTCAGAGGGTGGGTTAAACAACTGCAAATTGACTATCGTGGACACGAGGTCATGACAGCAGCCGCATCCCAAGGGTTAACCAGAAAAGGAGTTTACGCAGTCAGGCAAAAAGTTAACGAAAACCCACTCTATGCATCAACATCAGAGCACGTCAACGTACTCTTAACGCGTACGGAAGGTAAACTGGTATGGAAGACACTCTCTGGTGACCCGTGGATAAAGACGCTGCAGAACCCACCGAAAGGAAACTTCAAGGCAACTATTAAGGAGTGGGAGGTGGAGCACGCATCGATAATGGCGGGCATCTGCAGTCACCAAGTGACCTTTGACACGTTCCAAAACAAAGCCAACGTTTGCTGGGCTAAGAGCTTGGTCCCTGTCCTCGAAACAGCGGGGATAAAACTAAATGATAGGCAGTGGTCCCAGATAATTCAAGCCTTCAAAGAAGACAAAGCATACTCACCCGAAGTAGCCCTGAATGAAATATGCACGCGCATGTATGGGGTGGATCTAGACAGCGGGCTATTCTCTAAACCATTGGTATCTGTGTATTACGCGGATAACCACTGGGATAATAGGCCGGGAGGAAAGATGTTCGGATTCAACCCTGAGGCAGCGTCCATTCTAGAAAGAAAGTACCCGTTTACAAAAGGAAAGTGGAACATCAACAAGCAGATCTGCGTGACTACCAGGAGGATAGAAGACTTCAACCCTACCACCAACATTATACCGGCCAACAGGAGACTACCACACTCATTAGTGGCCGAACACCGCCCGGTAAAAGGGGAAAGAATGGAATGGCTGGTTAACAAGATAAACGGACATCATGTGCTCCTGGTTAGCGGCTATAACCTTGCACTGCCTACTAAGAGAGTCACCTGGGTAGCGCCACTAGGTGTCCGCGGAGCGGACTATACATACAACCTAGAGCTGGGTCTACCGGCAACACTTGGTAGGTATGACCTAGTGGTCATAAACATCCACACACCTTTTCGCATACACCATTACCAACAGTGCGTAGATCACGCAATGAAACTGCAAATGCTAGGGGGTGACTCACTGAGACTGCTCAAACCGGGTGGCTCTCTATTGATCAGAGCATACGGTTACGCAGATAGAACCAGTGAACGAGTAATCTGCGTACTGGGACGTAAGTTTAGATCATCCAGAGCATTGAAACCACCATGTATCACCAGTAATACTGAGATGTTTTTCCTATTCAGCAGTTTTGACAATGGCAGAAGGAATTTTACAACGCATGTCATGAACAATCAACTGAACGCAGCCTTTGTAGGACAGGCCACCCGAGCAGGATGTGCACCATCGTACCGGGTAAAACGCATGGACATCGCGAAGAACGATGAAGAGTGCGTGGTCAACGCCGCCAACCCTCGCGGGTTACCGGGTGACGGTGTTTGCAAGGCAGTATATAAAAAATGGCCGGAGTCCTTTAAAAATAGTGCAACACCAGTAGGAACTGCAAAAACAGTTATGTGCGGTACATATCCAGTAATCCACGCCGTAGGACCAAACTTCTCAAATTACTCGGAGTCCGAAGGGGACCGGGAATTGGCGGCTGCCTATCGAGAAGTCGCAAAAGAAGTAACTAGACTGGGAGTAAATAGCGTAGCTATACCTCTCCTCTCCACGGGTGTATACTCAGGAGGGAAAGACAGGCTAACCCAGTCACTGAACCACCTCTTTACAGCCATGGACTCGACGGATGCAGACGTGGTCATCTACTGCCGAGACAAGGAATGGGAGAAGAAAATATCTGAGGCCATACAGATGCGGACCCAAGTGGAGCTGCTGGATGAGCACATCTCCATAGACTGCGATGTCATTCGCGTGCACCCTGACAGTAGTTTGGCAGGTAGAAAAGGATACAGCACCACGGAAGGCGCACTGTATTCATATCTAGAAGGGACACGTTTTCACCAGACGGCAGTGGATGTGGCAGAGATACATACTATGTGGCCAAAGCAAACAGAGGCCAATGAGCAAGTCTGCCTATATGCCCTGGGGGAAAGTATTGAATCGATCAGGCAGAAATGCCCGGTGGATGATGCAGATGCATCATCTCCCCCGAAAACTGTCCCGTGCCTTTGCCGTTATGCCATGACTCCTGAACGCGTCACCCGACTTCGCATGAACCATGTCACAAATATAATTGTGTGTTCTTCATTTCCCCTTCCAAAGTACAAGATAGAAGGAGTGCAAAAAGTCAAATGCTCCAAGGTAATGTTATTTGATCACAATGTGCCATCGCGCGTAAGTCCAAGGGAATACAGATCTTCCCAGGAGTCTGTACGGGAAGTGAGTATGACAACGTCATTGACGCATAGCCAGTTTGATCTAAGCGCCGATGGCGAGACACTGCCTGTCCCGTCAGACCTGGATGCTGACGCCCCAGCCCTAGAACCGGCCCTAGACGACGGGGCGATCGGAAACCTTGCGGCCGTGTCTGACTGGGTAAAGAGCACCGTACCTGTCGCGCCGCCTAGAAGAAGGAGAGGGAGAAACCTGACCGTGGTATGTGACGAGAGAGAAGGGAATATAACACCCATGGCTAGCGTCCGATTCTTTAGAGCAGAGCAGTGTCCGGCCGTACAAGAAACAGCGGAGACGCGTGACACAGCTATCTCCCTTCGGGCACCGCCAAGTACCACCGTGGAACTGAGCCATCCACCGATCTCCTTCGGAGCACCAAGCGAGACGTTCCCCATCACATTTGGGGACTTCGACGATGGAGAAATCGAAAGCTTGTCTTCTGAGCTACTAACTTTCGGAGACTTCCTACCCGGTGAAGTGGATGATTTGACAGATAGCGACTGGTCCACGTGCTCAGACACGGACGACGAGTTATGACTAGACAGGGCAGGTGGGTATATATTCTCGTCGGACACTGGTCCAGGCCATTTACAACAGAAGTCGGTACGCCAGTCAGTGCTGCCGGTAAACACCCTGGAGGAAGTTCACGAGGAGAAGTGTTACCCACCTAAGCTGGATGAATTAAAGGAGCAACTACTACTTAAGAAACTCCAGGAGAGTGCGTCCACGGCCAATAGAAGCAGGTATCAATCACGCAAAGTGGAAAATATGAAAGCAACAATCATTCAGAGACTAAAGAGAGGCTGTAAACTGTATTTAATGGCAGAGACCCCGAAAGTCCCGACTTATCGGACCGTATACCCGGCGCCTGTGTACTCGCCTCCGATCAACGTCCGATTGTCCAATCCCGAGTCCGCAGTGGCAGCATGTAATGAGTTCTTAGCTAGAAACTACCCAACTGTTTCATCATACCAAATCACCGATGAGTATGATGCATATCTAGACATGGTGGACGGGTCGGAGAGTTGCTTGGACCGAGCGACATTCAATCCGTCAAAACTCAGGAGCTACCCGAAACAACATGCTTATCACGCGCCTTCTATCAGAAGCGCTGTACCTTCCCCATTCCAGAACACACTACAGAATGTACTGGCAGCAGCCACGAAAAGGAACTGCAACGTCACACAGATGAGGGAATTACCCACTTTGGACTCAGCAGTATTCAACGTGGAGTGTTTTAAAAAATTCGCATGCAACCGAGAATACTGGGAAGAATTTGCTGCCAGCCCTATCAGGATAACAACTGAGAATCTAACAACCTATGTCACTAAACTAAAGGGGCCGAAAGCAGCAGCGTTGTTTGCAAGAACCCATAATCTGCTGCCGCTGCAGGATGTACCAATGGATAGGTTCACAGTAGATATGAAAAGGGACGTGAAGGTAACTCCTGGTACAAAGCATACAGAGGAAAGGCCTAAGGTGCAGGTTATACAGGCGGCTGAACCCTTGGCAACAGCGTATCTATGTGGAATTCACAGAGAATTGGTTAGGAGATTGAACGCCGTCCTCCTACCCAATGTGCATACACTATTTGACATGTCTGCCGAGGACTTCGATGCCATTATAGCCGCACACTTTAAGCCAGGAGACGCCGTTTTAGAAACGGACATAGCCTCCTTTGATAAGAGCCAGGATGATTCACTTGCGCTCACCGCCTTAATGCTGTTAGAAGATTTGGGAGTGGATCACTCTTTGTTGGACCTGATAGAGGCTGCTTTTGGAGAGATTTCCAGCTGTCACCTGCCGACAGGTACGCGCTTCAAGTTCGGCGCTATGATGAAATCCGGTATGTTCCTAACTCTGTTCGTCAACACATTGTTAAATATCACCATCGCTAGCCGGGTGTTGGAAGATCGTCTGACAAAATCTGCGTGCGCGGCCTTCATCGGCGACGACAACATAATACATGGTGTCGTCTCCGATGAATTGATGGCAGCCAGATGCGCTACTTGGATGAACATGGAGGTGAAGATCATAGATGCAGTTGTATCCCAGAAAGCTCCTTACTTTTGTGGAGGGTTTATACTGCATGATACTGTGACAGGAACAGCTTGCAGGGTGGCGGACCCGCTAAAAAGGTTATTTAAACTGGGCAAACCGTTAGCGGCAGGTGACGAACAAGACGAAGACAGAAGACGGGCGCTGGCCGATGAAGTAATCAGATGGCAACGAACAGGGCTAATAGATGAGCTGGAGAAAGCGGTGTACTCTAGGTACGAAGTGCAGGGTATATCAGTTGCTGTAATGTCCATGGCCACCTTTGCAAGCTCCAGATCCAACTTCGAGAAGCTCAGAGGACCCGTCATAACTTTGTACGGCGGTCCTAAATAGGTACGCACTACAGCTACCTATTTTGCAGAAGCCGACAGTAGGTACCTAAATACCAATCAGCCATAATGGAGTTTATCCCAACCCAAACTTTCTACAATAGGAGGTACCAGCCTCGACCTTGGACTCCGCGCCCTACTATCCAAGTTATCAGACCCAGACCGCGTCCGCAAAGGAAGGCCGGGCAACTTGCCCAGCTGATCTCAGCAGTTAATAAACTGACAATGCGCGTGGTACCTCAACAGAAGCCGCGCAAGAATCGGAAGAATAAGAAGCAAAAGCAAAAGCAGCAGGCGCCACGAAACAATATGAATCAAAAGAAGCAGCCCCCTAAAAAGAAACCGGTTCAAAAGAAAAAGAAGCCGGGCCGCAGAGAGAGAATGTGCATGAAAATCGAAAATGATTGCATCTTCGAAGTCAAGCATGAAGGTAAGGTAACAGGTTACGCGTGCTTGGTAGGGGACAAAGTAATGAAGCCAGCACACGTAAAGGGGACCATCGATAACGCGGACCTGGCCAAATTGGCCTTCAAGCGGTCATCTAAGTACGACCTTGAATGCGCGCAGATACCCGTGCACATGAAGTCCGACGCTTCGAAGTTCACCCATGAGAAACCGGAGGGGTACTACAACTGGCACCACGGAGCAGTACAGTACTCAGGAGGCCGATTCACCATCCCTACAGGCGCGGGCAAACCAGGGGATAGTGGTAGACCGATCTTCGACAACAAGGGGCGCGTGGTGGCCATAGTTTTAGGAGGAGCTAATGAAGGAGCCCGTACAGCCCTCTCGGTGGTGACCTGGAACAAAGACATCGTCACGAAAATCACCCCTGAGGGGGCCGAAGAGTGGAGTCTGGCCATTCCAGTTATGTGCCTGCTGGCAAATACCACGTTCCCCTGCTCCCAGCCCCCTTGCACACCCTGCTGCTACGAAAAAGAGCCGGAGAAAACCTTGCGCATGCTTGAAGACAATGTCATGAGCCCCGGGTACTATCAGCTGCTACAAGCATCCTTAACATGTTCTCCCCGACGCCAGCGGCGCAGTATTAAGGACCACTTCAATGTCTATAAAGCCACAAGACCGTACCTAGCTCACTGCCCCGACTGTGGAGAAGGGCACTCGTGCCATAGTCCCGTAGCGCTAGAACGCATCAGAAATGAAGCGACAGATGGGACGTTGAAAATCCAGGTTTCCTTGCAAATCGGAATAAAGACGGATGATAGCCATGATTGGACCAAGCTGCGTTATATGGACAATCACATGCCAGCAGACGCAGAGCGGGCCGGGCTATTTGTAAGAACGTCAGCACCGTGCACGATTACTGGAACAATGGGACACTTCATTCTGGCCCGATGTCCGAAAGGAGAAACTCTGACGGTGGGGTTCACTGACGGTAGAAAGATCAGTCACTCATGTACGCACCCATTTCACCACGACCCTCCTGTGATAGGCCGGGAAAAATTTCATTCCCGACCGCAGCACGGTAGGGAACTACCTTGCAGCACGTACGCGCAGAGCACCGCTGCAACTGCCGAGGAGATAGAGGTACATATGCCCCCAGACACCCCAGATCGCACATTAATGTCACAACAGTCCGGCAATGTAAAGATCACAGTCAATAGTCAGACGGTGCGATACAAGTGCAATTGTGGTGATTCAAGCGAAGGATTAACCACTACAGATAAAGTGATTAATAACTGCAAGGTTGATCAATGCCATGCCGCGGTCACCAATCACAAAAAATGGCAGTACAATTCCCCTCTGGTCCCGCGCAATGCTGAATCCGGGGACCGGAAAGGAAAAGTTCACATTCCATTTCCTCTGGCAAATGTGACATGCAGGGTGCCTAAAGCAAGAAACCCCACCGTGACGTACGGAAAAAACCAAGTCATCATGTTGCTGTATCCTGACCACCCAACGCTCCTGTCCTACAGGAATATGGGAGAAGAACCAAACTATCAAGAAGAGTGGGTGACGCATAAGAAGGAGATCAGGTTAACCGTGCCGACTGAAGGGCTCGAGGTCACGTGGGGTAACAATGAGCCGTACAAGTATTGGCCGCAGTTATCCACAAACGGTACAGCCCACGGCCACCCGCATGAGATAATTCTGTATTATTATGAGCTGTACCCAACTATGACTGTGGTAGTTGTGTCAGTGGCCTCGTTCATACTCCTGTCGATGGTGGGTGTGGCAGTGGGGATGTGCATGTGTGCACGACGCAGATGCATTACACCATACGAACTGACACCAGGAGCTACCGTCCCTTTCCTGCTTAGCCTAATATGCTGCATTAGAACAGCTAAAGCGGCCACATACCAGGAGGCCGCGGTATACCTGTGGAACGAGCAGCAGCCTTTATTTTGGCTGCAAGCCCTTATTCCGCTGGCAGCCCTGATCGTCCTATGCAACTGTCTGAGACTCCTACCATGCTGTTGTAAAATGTTGACTTTTTTAGCCGTACTGAGCGTCGGTGCCCACACTGTGAGCGCGTACGAACACGTAACAGTGATCCCGAACACGGTGGGAGTACCGTATAAGACTCTAGTCAACAGACCGGGCTACAGCCCCATGGTACTGGAGATGGAGCTTCTGTCTGTCACCTTGGAACCGACGCTATCGCTTGATTACATCACGTGCGAGTATAAAACCGTTATCCCGTCTCCGTACGTGAAATGCTGCGGTACGGCAGAGTGTAAGGACAAGAGCCTACCTGATTACAGCTGTAAGGTCTTCACCGGCGTCTACCCATTCATGTGGGGCGGCGCCTACTGCTTCTGCGACACCGAGAATACGCAATTGAGCGAAGCACATGTGGAGAAGTCCGAATCATGCAAAACAGAATTTGCATCAGCATATAGGGCTCATACCGCATCCGCATCAGCTAAGCTCCGCGTCCTTTACCAAGGAAATAATATCACTGTAGCTGCTTATGCAAACGGCGACCATGCCGTCACAGTTAAGGACGCTAAATTCATAGTGGGGCCAATGTCTTCAGCCTGGACACCTTTCGACAATAAAATCGTGGTGTACAAAGGCGACGTCTACAACATGGACTACCCGCCCTTCGGCGCAGGAAGACCAGGACAATTTGGCGACATCCAAAGTCGCACGCCTGAGAGCGAAGACGTCTATGCTAATACACAACTGGTACTGCAGAGACCGTCCGCGGGTACGGTGCACGTGCCGTACTCTCAGGCACCATCTGGCTTCAAGTATTGGCTAAAAGAACGAGGGGCGTCGCTGCAGCATACAGCACCATTTGGCTGTCAAATAGCAACAAACCCGGTAAGAGCGATGAACTGCGCCGTAGGGAACATGCCTATCTCCATCGACATACCGGACGCGGCCTTTACCAGGGTCGTCGACGCGCCATCTTTAACGGATATGTCGTGTGAGGTATCAGCCTGCACCCATTCCTCAGACTTTGGGGGCGTAGCCATCATTAAATATGCAGCCAGTAAGAAAGGCAAGTGTGCAGTGCATTCGATGACTAACGCCGTCACTATTCGGGAAGCTGAAATAGAAGTAGAAGGGAACTCTCAGTTGCAAATCTCTTTTTCGACGGCCCTAGCCAGCGCCGAATTCCGCGTACAAGTCTGTTCTACACAAGTACACTGTGCAGCCGAGTGCCATCCACCGAAAGACCATATAGTCAATTACCCGGCGTCACACACCACCCCCGGGGTCCAAGACATTTCCGCTACGGCGATGTCATGGGTGCAGAAGATCACGGGAGGTGTGGGACTGGTTGTCGCTGTTGCAGCACTGATCCTAATCGTGGTGCTATGCGTGTCGTTTAGCAGGCACTAACTTGACAACTAGGTATGAAGGCATACGCGTCCCTAAAGAGACACACCACATATAGCTAAGAATCAATAGATAAGTATAGTAACAAAATATAAAAATCAACAAAAATCATAAAATAGAAAACTAGAAATAGAAGTAGGTAAGAAGGTATATGTGTCCCCTGAGAGACACACCATATATAGCTAAGAATCAATAGATAAGCATAGATCAAAGGGCTGAATAACCCCTGAATAGTAACAAAATATAAAAACCAATAAAAATCATAAAATAGAAAACCCTAAACAGAAGTAGTTCAAAGGGCTATAAAACCCCTGAATAGTAACAAAATATAAAACTAACAAAAATCAAA

>AB860301|2013|Philippines, Group 1

AAAGCGAGAGATTAATAACCCATCATGGATTCTGTGTACGTGGATATAGACGCTGACAGCGCCTTTTTGAAGGCCCTGCAACGTGCGTACCCCATGTTTGAGGTGGAACCTAGGCAGGTCACATCAAATGACCATGCTAATGCTAGAGCGTTCTCGCATCTAGCCATAAAACTAATAGAGCAGGAAATTGATCCCGACTCAACCATCCTGGATATAGGTAGTGCACCAGCAAGGAGGATGATGTCGGACAGGAAGTACCACTGCGTTTGCCCGATGCGCAGCGCAGAAGATCCCGAGAGACTCGCTAATTATGCGAGAAAGCTCGCATCTGCCGCAGGAAAAGTCCTGGACAGAAACATTTCTGGAAAGATCGGGGACTTACAAGAGGTGATGGCCGTGCCAGACACGGAGACGCCAACATTTTGCTTACACACAGATGTCTCATGTAGACAGAGAGCAGACGTCGCGATATACCAAGACGTCTATGCTGTACATGCACCCACGTCGCTATATCACCAGGCGATTAAAGGAGTCCGAGTGGCGTACTGGGTAGGGTTCGACACAACCCCGTTCATGTACAACGCTATGGCGGGTGCCTACCCCTCATACTCGACAAATTGGGCGGATGAGCAGGTACTGAAGGCTAAGAACATAGGATTATGTTCAACAGACCTGACAGAAGGTAGACGAGGCAAATTGTCTATCATGAGAGGGAAAAAGCTAAAACCGTGCGACCGTGTGCTGTTCTCAGTAGGGTCAACGCTTTACCCGGAAAGCCGCATGCTACTTAAGAGCTGGCACCTACCATCGGTGTTCCATCTAAAGGGCAAGCTTAGCTTCACATGCCGCTGTGACACAGTGGTTTCGTGTGAGGGCTACGTCGTTAAGAGAATAACGATGAGCCCAGGCCTTTATGGAAAAACCACGGGGTATGCGGTAACCCACCACGCAGACGGATTCTTGATGTGCAAGACTACCGACACGGTAGACGGTGAAAGAGTGTCATTCTCGGTGTGCACGTACGTGCCGGCGACCATTTGTGATCAAATGACCGGCATCCTTGCTACAGAAGTCACGCCGGAGGATGCACAGAAGCTGTTGGTGGGGCTGAACCAGAGAATAGTGGTTAACGGCAGAACGCAACGGAACACGAACACCATGAAGAACTACCTGCTTCCCGTGGTCGCCCAGGCCTTCAGTAAGTGGGCGAAGGAGTGCCGGAAGGACATGGAAGATGAGAAGCTTCTGGGGGTCAGAGAAAGAACACTGACCTGCTGCTGTCTGTGGGCATTTAAGAAGCAGAAAACACACACGGTCTACAAGAGGCCTGATACCCAGTCAATCCAGAAGGTTCAGGCCGAATTCGACAGCTTTGTAGTACCAGGCCTGTGGTCGTCCGGGTTGTCAATCCCGTTGAGGACTAGAATCAAGTGGCTGTTACGCAAGGTGCCGAAAGCAGACCTGATCCCATACAGCGGAAATGCCCAAGAAGCCCAGGATGCTGAAAAAGAAGCAGAGGAAGAACGAGAAGCAGAACTGACTCATGAGGCTCTACCACCCCTACAGGCAGCACAGGAAGATGTCCAGGTCGAAATCGACGTGGAACAACTTGAGGATAGAGCTGGTGCTGGAATAATAGAGACTCCGAGAGGCGCTATTAAAGTTACTGCCCAACTAACGGACCACGTCGTGGGGGAGTACCTGGTACTTTCCCCGCAGACCGTATTACGCAGCCAGAAGCTCAGCCTGATCCACGCTTTAGCGGAGCAAGTGAAGACGTGTACGCATAGCGGACGAGCAGGGAGGTATGCGGTCGAAGCGTACGATGGCCGAGTCCTAGTGCCCTCAGGCTATGCAATTTCGCCCGAAGACTTCCAGAGTCTAAGCGAAAGCGCAACGATGGTGTACAACGAAAGAGAGTTCGTAAACAGAAAGTTACACCACATTGCGATGCACGGACCAGCTCTGAACACTGACGAAGAGTCGTATGAGCTTGTGAGGGCAGAGAGGACAGAACACGAGTACGTCTACGACGTGGACCAGAGAAGATGCTGCAAGAAGGAAGAAGCTGCAGGATTGGTACTGGTGGGCGACTTGACTAATCCGCCCTACCACGAATTCGCATACGAAGGGCTAAAAATTCGCCCCGCTTGTCCATATAAAATTGCAGTCATAGGAGTCTTCGGGGTACCAGGATCTGGTAAGTCAGCCATTATCAAGAACCTAGTTACCAGGCAAGACCTGGTGACTAGCGGAAAGAAAGAAAACTGCCAAGAAATCAGCACCGACGTGATGAGACAGAGAGGTCTAGAAATATCTGCACGTACGGTTGATTCGCTGCTCTTGAATGGATGCAATAGACCAGTCGACGTGTTGTACGTAGACGAGGCGTTTGCGTGCCACTCTGGAACGTTACTTGCCTTGATCGCCTTGGTGAGACCAAGACTGAAAGTTGTACTTTGTGGTGACCCGAAGCAGTGCGGCTTCTTCAATATGATGCAGATGAAAGTCAACTACAATCATAACATCTGCACTCAAGTGTACCACAAAAGTATTTCCAGGCGGTGTACACTGCCTGTGACTGCCATTGTGTCGTCGTTGCATTACGAAGGCAAAATGCGCACTACGAATGAGTACAACATGCCGATTGTAGTGGACACTACGGGCTCAACAAAACCTGACCCTGGAGACCTCGTGTTAACGTGCTTCAGAGGGTGGGTTAAACAACTGCAAATTGACTATCGTGGACACGAGGTCATGACAGCAGCTGCATCCCAAGGGTTAACCAGAAAAGGAGTTTACGCAGTTAGGCAAAAAGTTAACGAAAACCCACTCTATGCATCAACATCAGAGCACGTCAACGTACTCCTAACGCGTACGGAAGGTAAACTGGTATGGAAGACACTCTCTGGTGACCCGTGGATAAAGACGCTGCAGAACCCACCGAAAGGAAACTTCAAGGCAACTATTAAGGAGTGGGAGGTGGAGCACGCATCGATAATGGCGGGCATCTGCAGTCACCAAGTGACCTTTGACACGTTCCAAAACAAAGCCAACGTTTGCTGGGCTAAGAGCTTGGTCCCTATCCTCGAAACAGCGGGGATAAAACTAAATGATAGGCAGTGGTCCCAGATAATTCAAGCTTTCAAAGAAGACAAAGCATACTCACCCGAAGTAGCCCTGAATGAAATATGCACGCGCATGTATGGGGTGGATCTAGACAGCGGGCTATTCTCTAAACCGTTGGTATCTGTGTATTACGCGGATAACCACTGGGATAATAGGCCGGGAGGAAAGATGTTCGGATTCAACCCTGAGGCAGCGTCCATTCTAGAAAGAAAGTACCCGTTTACAAAAGGAAAGTGGAACATCAACAAGCAGATCTGCGTGACTACCAGGAGGATAGAAGACTTCAACCCTACCACCAACATTATACCGGCCAACAGGAGACTACCACACTCATTAGTGGCCGAACACCGCCCAGTAAAAGGGGAAAGAATGGAATGGCTGGTTAACAAGATAAACGGACATCATGTGCTCCTGGTTAGCGGCTATAACCTTGCACTGCCTACTAAGAGAGTCACCTGGGTAGCGCCACTAGGTGTCCGCGGAGCGGACTATACATACAACCTAGAGCTGGGTCTACCGGCAACACTTGGTAGGTATGACCTAGTGGTCATAAACATCCACACACCTTTTCGCATACACCATTACCAACAGTGCGTAGATCACGCAATGAAACTGCAAATGCTAGGAGGTGACTCACTGAGACTGCTCAAACCGGGTGGCTCTCTATTGATCAGAGCATACGGTTACGCAGATAGAACCAGTGAACGAGTAATCTGCGTACTGGGACGTAAGTTTAGATCGTCCAGAGCATTGAAACCACCATGTATCACCAGTAATACTGAGATGTTCTTCCTATTTAGCAGTTTTGACAATGGCAGAAGGAATTTTACAACGCATGTTATGAACAATCAACTGAACGCAGCCTTTGTAGGACAGGCCACCCGAGCAGGATGTGCACCATCGTACCGGGTAAAGCGCATGGACATCGCGAAGAACGATGAAGAGTGCGTGGTCAACGCCGCCAACCCTCGCGGGTTACCGGGTGACGGTGTTTGCAAGGCAGTATATAAAAAATGGCCGGAGTCCTTTAAAAATAGTGCAACACCAGTAGGAACCGCAAAAACAGTTATGTGCGGTACATATCCAGTAATCCATGCCGTAGGACCAAACTTCTCAAATTACACGGAGTCCGAAGGGGACCGGGAATTGGCGGCTGCCTATCGAGAAGTCGCAAAGGAAGTAACTAGACTGGGAGTAAATAGCGTAGCTATACCTCTCCTCTCCACAGGTGTATACTCAGGAGGGAAAGACAGGCTAACCCAGTCACTGAACCACCTCTTTACAGCCATGGACTCGACGGATGCAGACGTGGTCATCTACTGCCGAGACAAGGAATGGGAGAAGAAAATATCTGAGGCCATACAGATGCGGACCCAAGTGGAGCTGCTGGATGAGCACATCTCCATAGACTGCGATGTCATTCGCGTGCACCCTGACAGTAGTTTGGCAGGTAGAAAAGGATACAGCACCACGGAAGGCGCACTGTATTCATATCTAGAAGGGACACGTTTTCACCAGACGGCAGTGGATGTGGCAGAGATACATACTATGTGGCCAAAGCAAATAGAGGCCAATGAGCAAGTCTGCCTATATGCCCTGGGGGAAAGTATTGAGTCAATCAGGCAGAAATGCCCGGTGGATGATGCAGATGCATCATCTCCCCCGAAAACCGTCCCGTGCCTTTGCCGTTATGCCATGACTCCTGAACGCGTCACCCGACTTCGCATGAACCATGTCACAAATATAATTGTGTGTTCTTCATTTCCCCTTCCAAAGTACAAGATAGAAGGAGTGCAAAAAGTCAAATGCTCCAAGGTAATGTTATTTGATCACAATGTGCCATCGCGCGTAAGTCCAAGGGAATACAGATCTTCCCAGGAGTCTGTACGGGAAGTGAGTATGACAACGTCATTGACGCATAGTCAGTTTGATCTAAGCGCCGATGGCGAGACACTGCCCGTCCCGTCAGACCTGGATGCTGACGCCCCAGCCCTAGAACCGGCCCTAGACGACGGGGCGATACATACGACCGGAAACCTTGCGGCCGTGTCTGACTGGGTAATGAGCACCGTACCCGTCGCGCCGCCTAGAAGAAGGAGAGGGAGAAACCTGACCGTGATATGTGACGAGAGAGAAGGGAATATAACACCCATGGCTAGCGTCCGATTCTTTAGAGCAGAGCAGTGTCCGACCGTACAAGAAACAGCGGAGACGCGTGACACAGCTATTTCCTTTCGGGCACCGCCAAGTATCACCGTGGAACTGAGCCATCCACCGATCTCCTTCGGAGCACCAAGCGAGACGTTCCCCATCACATTTGGGGACTTCAACGATGGAGAAATCGAAAGCTTGTCTTCTGAGCTACTAACTTTCGGAGACTTCCTACCCGGTGAAGTGGATGATTTGACAGATAGCGACTGGTCCACGTGCTCAGACACGGACGACGAGTTATGACTAGACAGGGCAGGTGGGTATATATTCTCGTCGGACACTGGTCCAGGCCATTTACAACAGAAGTCGGTACGCCAGTCAGTGCTGCCGGTAAACACCCTGGAGGAAGTTCACGAGGAGAAGTGTTACCCACCTAAGCTGGATGAATTAAAGGAGCAACTACTACTTAAGAAACTCCAGGAGAGTGCGTCCACGGCCAATAGAAGCAGGTATCAATCACGCAAAGTGGAAAATATGAAAGCAACAATCATCCAGAGACTAAAGAGAGGCTGTAAACTGTATTTAATGGCAGAGACCCCGAAAGTCCCGACTTATCGGACCGTATACCCGGCGCCTGTGTACTCGCCTCCGATCAACGTCCGATTGTCCAATCCCGAGTCCGCAGTGGCAGCATGTAATGAGTTCTTAGCTAGAAACTACCCAACTGTTTCATCATACCAAATCACCGATGAGTATGATGCATATCTAGACATGGTGGACGGGTCGGAGAGTTGCTTGGACCGAGCGACATTCAATCCGTCAAAACTTAGGAGCTACCCGAAACAACATGCTTATCACGCGCCCTCTATCAGAAGCGCTGTACCTTCCCCATTCCAGAACACACTACAGAATGTACTGGCAGCAGCCACGAAAAGGAACTGCAACGTCACACAGATGAGGGAATTACCCACTTTGGACTCAGCAGTATTCAACGTGGAGTGTTTTAAAAAATTCGCATGCAACCGAGAATACTGGGAAGAATTTGCTGCCAGCCCTATCAGGATAACGACTGAGAATCTAACAACCTATGTCACTAAATTAAAGGGGCCAAAAGCAGCAGCGTTGCTTGCAAGAACCCATAATCTGCTGCCGCTGCAGGATGTACCAATGGATAGGTTCACAGTAGATATGAAAAGGGACGTGAAGGTAACTCCTGGCACAAAGCATACAGAGGAAAGGCCTAAGGTGCAGGTTATACAGGCGGCTGAACCCTTGGCAACAGCGTACCTATGTGGAATTCACAGAGAATTGGTTAGGAGATTGAACGCCGTCCTCCTACCCAACGTGCATACACTATTTGACATGTCTGCCGAGGACTTCGATGCCATTATAGCCGCACACTTTAAGCCAGGAGACGCCGTTTTAGAAACGGACATAGCCTCCTTTGATAAGAGCCAGGATGATTCACTTGCGCTTACCGCCTTAATGCTGTTAGAAGATTTGGGAGTGGATCACTCCTTGTTGGACCTGATAGAGGCTGCTTTTGGAGAGATTTCCAGCTGTCACCTGCCGACAGGTACGCGCTTCAAGTTCGGCGCTATGATGAAATCCGGTATGTTCCTAACTCTGTTCGTCAACACATTGTTAAATATCACCATCGCTAGCCGGGTGTTGGAAGATCGTCTGACAAAATCTGCATGCGCGGCCTTCATCGGCGACGACAACATAATACATGGTGTCGTCTCCGATGAATTGATGGCAGCCAGATGCGCTACTTGGATGAACATGGAAGTGAAGATCATAGATGCAGTTGTATCCCAGAAAGCTCCCTACTTTTGTGGAGGGTTTATACTGCATGATACTGTGACAGGAACAGCTTGCAGGGTGGCGGACCCGCTAAAAAGGTTATTTAAACTGGGCAAACCGTTAGCGGCAGGTGACGAACAAGACGAAGACAGAAGGCGGGCGCTGGCTGATGAAGTAATCAGATGGCAACGAACAGGGCTAATAGATGAGCTGGAGAAAGCGGTGTACTCTAGGTACGAAGTGCAGGGTATATCAGTTGCTGTAATGTCCATGGCCACCTTTGCAAGCTCCAGATCCAACTTCGAGAAGCTCAGAGGACCCGTCATAACCTTGTACGGCGGTCCTAAATAGGTACGCACTACAGCTACCTATTTTGCAAAAGCCGACAGCAGGTACCTAAATACCAATCAGCCATAATGGAGTTTATCCCAACCCAAACTTTCTACAATAGGAGGTACCAGCCTCGACCTTGGACTCCGCGCCCTACTATCCAAGTCATCAGACCCAGACCACGTCCGCAAAGGAAGGCCGGGCAACTTGCCCAGCTGATCTCAGCAGTTAATAAACTGACAATGCGCGTGGTACCTCAACAGAAGCCGCGCAAGAATCGGAAGAATAAGAAGCAAAAGCAAAAGCAGCAGGCGCCACGAAACAATACGAATCAAAAGAAGCAGCCCCCTAAAAAGAAACCGGTTCAAAAGAAAAAGAAGCCGGGCCGCAGAGAGAGAATGTGCATGAAAATCGAAAATGATTGCATCTTCGAAGTCAAGCATGAAGGTAAGGTAACAGGTTACGCGTGCTTGGTAGGGGACAAAGTAATGAAGCCAGCACACGTAAAGGGGACCATCGATAACGCGGACCTGGCCAAATTGGCCTTCAAGCGGTCATCTAAGTACGACCTTGAATGCGCGCAGATACCCGTGCACATGAAGTCCGACGCTTCGAAGTTCACCCATGAGAAACCGGAGGGGTACTACAACTGGCACCACGGAGCAGTACAGTACTCAGGAGGCCGATTCACCATCCCTACAGGTGCGGGCAAACCAGGGGATAGTGGTAGACCGATCTTCGACAACAAGGGGCGCGTGGTGGCCATAGTTTTAGGAGGAGCTAATGAAGGAGCCCGTACAGCCCTCTCGGTGGTGACCTGGAACAAAGACATCGTCACGAAAATCACCCCTGAGGGGGCCGAAGAGTGGAGTCTGGCCATTCCAGTTATGTGCCTGCTGGCAAATACCACGTTCCCCTGCTCCCGGCCCCCTTGCACACCCTGCTGCTACGAAAAAGAGCCGGAGAAAACCTTGCGCATGCTTGAAGACAATGTCATGAGCCCCGGGTACTATCAGCTGCTACAAGCATCCTTAACATGTTCTCCCCGACGCCAGCGGCGCAGTATTAAGGACCACTTCAATGTCTATAAAGCCACAAGACCGTACCTAGCTCACTGTCCCGACTGTGGAGAAGGGCACTCGTGCCATAGTCCCGTAGCGCTAGAACGCATCAGAAACGAAGCGACAGACGGGACGTTGAAAATCCAGGTTTCCTTGCAAATCGGAATAAAGACGGATGATAGCCATGATTGGACCAAGCTGCGTTATATGGACAATCACATGCCAGCAGACGCAGAGCGGGCCGGGCTATTTGTAAGAACGTCAGCACCGTGCACGATTACTGGAACAATGGGACACTTCATTCTGGCCCGATGTCCGAAAGGAGAAACTCTGACGGTGGGGTTCACTGACGGTAGGAAGATCAGTCACTCATGTACGCACCCATTTCACCATGACCCTCCTGTGATAGGCCGGGAAAAATTCCATTCCCGACCGCAGCACGGTAGGGAACTACCTTGCAGCACGTACGCGCAGAGCACCGCTGCAACTGCCGAGGAGATAGAGGTACATATGCCCCCAGACACCCCAGATCGCACATTAATGTCACAACAGTCCGGCAATGTAAAGATCACAGTCAATAGTCAGACGGTGCGGTACAAGTGCAATTGTGGTGACTCAAGTGAAGGATTAACCACTACAGATAAAGTGATTAATAACTGCAAGGTCGATCAATGCCATGCCGCGGTCACCAATCACAAAAAATGGCAGTACAATTCCCCTCTGGTCCCGCGTAATGCTGAATTCGGGGACCGGAAAGGAAAAGTTCACATTCCATTTCCTCTGGCAAATGTGACATGCAGGGTGCCTAAAGCAAGAAACCCCACCGTGACGTACGGAAAAAACCAAGTCATCATGTTGCTGTATCCTGACCACCCAACGCTCCTGTCCTACAGGAATATGGGAGAAGAACCAAACTATCAAGAAGAGTGGGTGACGCATAAGAAGGAGATCAGGTTAACCGTGCCGACTGAAGGGCTCGAGGTCACGTGGGGTAACAATGAGCCGTACAAGTATTGGCCGCAGTTATCCACAAACGGTACAGCCCACGGCCACCCGCATGAGATAATTCTGTATTATTATGAGCTGTACCCAACTATGACTGTGGTAGTTTTGTCAGTGGCCTCGTTCATACTCCTGTCGATGGTGGGTGTGGCAGTGGGGATGTGCATGTGTGCACGACGCAGATGCATTACACCGTACGAACTGACACCAGGAGCTACCGTCCCTTTCCTGCTTAGCCTAATATGCTGCATTAGAACAGCTAAAGCGGCCACATACCAGGAGGCCGCGGTATACCTGTGGAACGAGCAGCAGCCTTTATTTTGGCTGCAAGCCCTTATTCCGCTGGCAGCCCTGATTGTCCTATGTAACTGTCTGAGACTCTTACCATGCTGTTGTAAAATGTTGACTTTTTTAGCCGTACTGAGCGTCGGTGCCCACACTGTGAGCGCGTACGAACACGTAACAGTGATCCCGAACACGGTGGGAGTACCGTATAAGACTCTAGTCAACAGACCGGGCTACAGCCCCATGGTATTGGAGATGGAGCTTCTGTCTGTCACCTTGGAACCAACGCTATCGCTTGATTACATCACGTGCGAGTATAAAACCGTTATCCCGTCTCCGTACGTGAAATGCTGCGGTACAGCAGAGTGTAAGGACAAGAGCCTACCTGATTACAGCTGTAAGGTCTTCACCGGCGTCTACCCATTCATGTGGGGCGGCGCCTACTGCTTCTGCGACACCGAAAATACGCAACTGAGCGAAGCACATGTGGAGAAGTCCGAATCATGCAAAACAGAATTTGCATCAGCATACAGGGCTCATACCGCATCCGCATCAGCTAAGCTCCGCGTCCTTTACCAAGGAAATAATATCACTGTAGCTGCTTATGCAAACGGCGACCATGCCGTCACAGTTAAGGACGCTAAATTCATAGTGGGGCCAATGTCTTCAGCCTGGACACCTTTCGACAATAAAATCGTGGTGTACAAAGGCGACGTCTACAACATGGACTACCCGCCCTTCGGCGCAGGAAGACCAGGACAATTTGGCGACATCCAAAGTCGCACGCCTGAGAGCGAAGACGTCTATGCTAATACACAACTGGTACTGCAGAGACCGTCCGCGGGTACGGTGCACGTGCCGTACTCTCAGGCACCATCTGGCTTCAAGTATTGGCTAAAAGAACGAGGGGCGTCGCTGCAGCACACAGCACCATTTGGCTGTCAAATAGCAACAAACCCGGTAAGAGCGATGAACTGCGCCGTAGGGAACATGCCTATCTCCATCGACATACCGGACGCGGCCTTTACTAGGGTCGTCGACGCGCCATCTTTAACGGACATGTCGTGTGAGGTATCAGCCTGCACCCATTCCTCAGACTTTGGGGGCGTAGCCATCATTAAATATGCAGCCAGTAAGAAAGGCAAGTGTGCAGTGCACTCGATGACTAACGCCGTCACTATTCGGGAAGCTGAAATAGAAGTAGAAGGGAACTCTCAGTTGCAAATCTCTTTTTCGACGGCCCTAGCCAGCGCCGAATTTCGCGTACAAGTCTGTTCTACACAAGTACACTGTGCAGCCGAGTGCCATCCACCGAAAGACCATATAGTCAATTACCCGGCGTCACACACCACCCTCGGGGTCCAAGACATTTCCGCTACGGCGATGTCATGGGTGCAGAAGATCACGGGAGGTATGGGACTGGTTGTCGCTGTTGCAGCACTGATCCTAATCGTGGTGCTATGCGTGTCGTTTAGCAGGCACTAACTTGACAACTAGGTATGAAGGCATACGCGTCCCTAAAGAGACACACCGCATATAGCTAGGAATCAATAGATAAGTATAGATCTAAGGGCTGAACAACCCCTGGATAGTAACAAAATATAAAAATCAACAAAAATCATAAAATAGAAAACTAGAAATAGAAGTAGGTAAGAAGGTAYATGTGTCCCCTAAGAGACACACCATATATAGCTAAGAATCAATAGATAAGCATAGATCAAAGGGCTGAACAACCCCTGAATAATAACAAAATATAAAAACCAATAAAAATCATAAAATAGAAAACCACAAATAGAAGTAGTTCAAAGGGCTATAAAACCCCTGAATAGTAACAAAATATAAAACTAATAAAAATCAAACGAATACCATAATTGGCAATCGGAAGAGATGTAGGTACTTAAGCTTCTTAAAAGCAGCCGAACTCGCTTTGAGATGTAGGCGTAGCACACCGAACTCTTCCATAATTCTCCGAACCCACAGGGACGTAGGAGATGTTCAAAGTGACTATAAAACCCTGAACAGTAATAAAATATAAAATTAATAATGAGTACCATAATTGGCAAATGGAAGAGACGTAGGTACTAAGCTTCTTAAAAGCAGCCGAACTCACTTTGAGATGTAGGCATAGCATACCGAACTCTTCCACAATTCTCCGTACCCATAGGGACG

>HM045817|2005|Senegal, Group 2

ACGTAGCCTACCAGTTTCTTACTGCTCTACTCTGCTTAGCAAGAGACTTGAGAACCCATCATGGATCCCGTGTACGTGGACATAGACGCCGACAGCGCCTTTTTGAAGGCCCTGCAGCGTGCGTACCCCATGTTTGAGGTGGAACCAAGGCAGGTCACACCGAATGACCATGCCAATGCTAGAGCATTCTCGCATCTAGCTATAAAACTAATAGAGCAGGAAATTGATCCCGACTCAACCATCCTGGACATAGGCAGCGCGCCAGCAAGGAGGATGATGTCGGATAGGAAGTACCACTGCGTTTGCCCAATGCGCAGCGCAGAAGACCCTGAGAGACTCGCCAACTACGCGAGAAAACTAGCATCTGCCGCAGGAAAAGTCTTGGACAGAAACATCTCCGGAAAAATCGGAGATCTACAAGCAGTAATGGCCGTACCAGACGCAGAAACGCCCACATTCTGCTTGCACACTGACGTCTCATGTAGACAAAGGGCGGACGTCGCTATATACCAGGACGTTTACGCCGTGCATGCACCAACATCGCTATACCACCAGGCGATTAAAGGAGTCCGTGTAGCATACTGGATAGGGTTTGATACAACCCCGTTCATGTATAATGCCATGGCAGGTGCATACCCCTCGTACTCGACAAACTGGGCAGATGAGCAGGTGCTGAAGGCAAAGAACATAGGATTATGCTCAACAGACCTGACGGAAGGTAGACGAGGTAAATTGTCTATCATGAGAGGAAAAAAGATGAAGCCATGTGACCGCGTACTGTTCTCAGTCGGGTCAACGCTTTACCCGGAGAGCCGTAAGCTTCTTAAGAGTTGGCACTTACCTTCAGTGTTCCATCTAAAAGGGAAGCTCAGCTTCACGTGCCGCTGTGATACAGTGGTTTCGTGTGAAGGCTATGTCGTTAAGAGAATAACGATTAGCCCGGGCCTCTACGGTAAAACCACAGGGTACGCAGTAACCCACCATGCAGACGGATTCATAATGTGCAAAACAACCGATACGGTAGACGGCGAGAGAGTGTCATTTTCGGTATGCACGTACGTACCCGCAACCATTTGTGATCAAATGACAGGTATTCTTGCCACGGAGGTTACACCGGAGGATGCACAGAAGCTGCTGGTGGGACTGAACCAGAGGATAGTGGTCAATGGCAGAACGCAGAGGAACACGAACACAATGAAGAATTACTTGCTTCCTGTGGTAGCCCAAGCCTTCAGTAAGTGGGCAAAGGAATGCCGGAAAGATATGGAAGATGAAAAACTTTTGGGCATCAGAGAAAGGACACTGACATGCTGCTGCCTTTGGGCGTTTAAGAAGCAGAAGACACACACGGTCTACAAGAGGCCTGACACTCAGTCAATTCAGAAAGTCCCAGCCGAATTTGACAGCTTTGTGGTACCAAGTCTGTGGTCATCTGGACTGTCGATCCCGCTACGGACCAGAATCAAGTGGCTGCTAAGCAAAGTGCCAAAGACTGATTTGATCCCTTACAGCGGTGACGCCAAAGAAGCCCGCGACGCTGAAAAAGAAGCAGAAGAAGAACGAGAAGCGGAGCTAACTCGCGAGGCACTACCACCACTACAGGCGGCACAGGATGACGTCCAGGTCGAAATTGACGTGGAACAGCTCGAAGACAGAGCTGGGGCAGGAATAATTGAAACTCCAAGAGGAGCCATCAAAGTCACTGCCCAACCAACAGACCACGTCGTGGGAGAGTACTTGGTACTTTCCCCGCAGACCGTGTTACGAAGCCAAAAGCTCAGCCTGATCCACGCATTGGCGGAACAAGTGAAGACATGCACACACAGCGGACGAGCAGGAAGGTACGCGGTCGAAGCATACGACGGCAGAATCCTTGTGCCCTCAGGCTATGCAATATCACCTGAAGACTTCCAGAGCCTGAGCGAAAGTGCGACGATGGTGTATAATGAAAGGGAGTTCGTAAATAGGAAATTACACCATATCGCGTTGCACGGACCAGCCCTGAACACTGACGAGGAGTCGTACGAGCTGGTAAGGGCAGAAAGGACAGAGCATGAGTACGTCTATGATGTGGACCAAAGAAGGTGCTGCAAGAAAGAGGAGGCAGCCGGGCTGGTACTGGTCGGTGACTTGACCAACCCGCCCTACCATGAGTTCGCATATGAAGGGCTGAGAATCCGCCCCGCCTGCCCATACAAGACCGCAGTAATAGGGGTCTTTGGAGTGCCAGGATCCGGCAAATCAGCAATCATTAAGAACCTGGTTACCAGGCAAGACCTCGTGACCAGTGGAAAGAAAGAAAATTGCCAAGAAATCTCCACTGATGTGATGCGACAGAGGAACCTGGAGATATCTGCACGCACGGTCGACTCACTGCTCTTGAACGGATGCAACAGACCAGTCGACGTGTTGTACGTCGACGAAGCGTTTGCGTGCCATTCTGGCACGCTACTTGCTCTGATAGCCTTGGTGAGACCGAGGCAGAAAGTCGTGCTATGCGGTGATCCGAAACAGTGCGGCTTCTTCAATATGATGCAGATGAAAGTTAACTACAACCATAACATCTGCACCCAGGTGTACCATAAAAGTATTTCCAGGCGGTGTACACTGCCTGTGACTGCCATTGTGTCCTCGTTGCATTACGAAGGCAAAATGCGCACAACAAATGAGTACAACAAGCCAATTGTAGTGGATACTACAGGCTCGACAAAACCCGACCCCGGAGACCTTGTGCTAACATGTTTCAGAGGGTGGGTTAAGCAACTGCAAATTGACTATCGTGGACACGAGGTCATGACAGCAGCTGCATCCCAGGGGCTAACCAGAAAAGGGGTCTATGCCGTCAGGCAAAAAGTCAATGAAAACCCCCTTTACGCATCAACATCAGAGCACGTGAACGTGCTGCTGACGCGTACGGAAGGCAAACTAGTATGGAAGACACTTTCTGGAGACCCATGGATAAAGACACTGCAGAACCCGCCGAAAGGAAATTTTAAAGCAACAATTAAGGAATGGGAAGTGGAACATGCTTCAATAATGGCGGGTATCTGTAACCACCAAGTGACCTTTGACACGTTCCAGAATAAAGCCAATGTCTGCTGGGCGAAGAGCTTAGTCCCCATCCTAGAAACAGCAGGGATAAAACTAAACGACAGGCAGTGGTCCCAGATAATCCAGGCTTTTAAAGAAGACAGAGCATACTCACCCGAGGTGGCCCTGAATGAGATATGCACGCGCATGTACGGGGTAGACCTGGACAGCGGACTGTTCTCTAAACCACTGGTGTCCGTGCATTATGCGGATAATCACTGGGACAACAGGCCGGGAGGGAAGATGTTCGGATTCAACCCCGAAGCGGTGTCCATACTGGAGAGGAAATACCCGTTTACAAAAGGGAAGTGGAATACCAACAAGCAAATCTGTGTGACTACTAGAAGGATTGAAGATTTTAACCCGAACATCAACATTATACCTGCCAACAGGAGACTACCGCATTCATTGGTGGCCGAACATCGCCCGGTAAAAGGGGAGAGGATGGAATGGTTGGTCAACAAAATAAATGGCCACCATGTGCTCCTGGTCAGCGGCTACAACCTCGTTCTGCCCACTAAGAGAGTCACCTGGGTGGCGCCGCTGGGCATTCGGGGAGCTGACTACACGTATAACCTAGAGTTAGGCCTACCAGCAACGCTCGGTAGGTATGACCTAGTGATTATAAACATCCACACACCCTTTCGCATACATCATTACCAACAGTGCGTGGATCACGCAATGAAGCTGCAGATGCTCGGAGGAGACTCCCTGAGACTGCTCAAGCCGGGTGGTTCATTACTGATCAGGGCATACGGCTACGCAGACAGAACAAGCGAACGAGTAGTCTGCGTACTGGGACGCAAGTTCCGATCATCCAGAGCGTTGAAACCGCCGTGCGTCACTAGTAACACCGAGATGTTTTTCTTGTTCAGCAACTTTGATAACGGCAGAAGGAACTTTACCACGCACGTAATGAACAACCAGCTGAATGCCGCTTTTGTTGGTCAGGCCACCCGAGCAGGGTGTGCACCGTCGTACCGGGTCAAACGCATGGACATCGCAAAGAACGATGAAGAGTGCGTAGTCAACGCCGCCAACCCTCGTGGGCTACCAGGCGATGGCGTCTGTAAAGCAGTATACAAAAAATGGCCGGAGTCCTTCAAGAACAGTGCAACACCAGTGGGAACCGCAAAGACAGTCATGTGCGGTACATACCCGGTAATCCATGCAGTAGGACCTAATTTCTCAAATTACTCTGAGTCCGAAGGAGACCGGGAGTTGGCAGCTGCTTACCGAGAAGTCGCTAAGGAGGTGACTAGACTAGGAGTAAACAGCGTAGCTATACCGCTCCTTTCCACCGGTGTGTACTCTGGAGGAAAAGACAGGCTGACTCAGTCATTAAACCACCTATTTACAGCATTAGATTCAACTGATGCTGATGTGGTTATCTACTGCCGTGACAAGGAGTGGGAGAAGAAAATAGCCGAGGCCATACAAATGAGGACCCAAGTGGAACTGCTAGACGAACACATCTCTGTAGACTGCGATATCATCCGAGTGCACCCTGACAGCAGTCTGGCAGGTAGAAAAGGGTACAGCACTACAGAAGGTTCACTGTACTCCTACTTGGAAGGGACACGGTTCCATCAGACGGCAGTGGACATGGCAGAAGTATACACCATGTGGCCAAAGCAGACGGAGGCTAATGAACAAGTTTGCTTGTACGCATTGGGGGAAAGTATAGAATCAATCAGGCAAAAGTGCCCAGTGGATGACGCAGATGCATCGTCGCCCCCAAAAACAGTCCCGTGCCTCTGCCGTTATGCCATGACACCCGAACGAGTCACCAGGCTTCGTATGAACCATGTCACAAGCATAATAGTATGCTCATCATTCCCCCTTCCAAAGTACAAAATAGAAGGAGTGCAGAAAGTCAAGTGCTCTAAAGTGATGCTGTTCGACCATAACGTGCCATCACGCGTTAGTCCAAGGGAATATAAATCACCTCAGGAGACCGCACAAGAAGTAAGTTCGACCACGTCACTGACGCACAGCCAATTCGACCTTAGCGTTGACGGTGAGGAACTGCCCGCTCCGTCTGACTTGGAAGCTGATGCTCCAATTCCGGAACCAACACCAGACGACAGAGCGGTACTTACTTTGCCTCCCACGATTGAAAATTTTTCGGCTGTGTCAGACTGGGTAATGAATACCGCGCCAGTCGCACCACCCAGAAGAAGACGTGGGAAAAACCTGAATGTCACCTGCGACGAGAGAGAAGGGAACGTACTTCCCATGGCTAGCGTTCGGTTTTTCAGAGCGGACCTGCACTCCATCGAACAGGAAACGGCAGAGATACGCGATACGGCTGCGTCCCTCCAGGCGCCCCTGAGTGTCGCTACAGAACTAAATCAACTGCCGATCTCATTCGGAGCACCAAACGAGACTTTCCCCATAACGTTTGGGGACTTTGATGAAGGGGAGATTGAAAGCTTGTCCTCTGAGTTACTGACCTTTGGGGACTTCTCGCCGGGCGAAGTGGATGATCTGACAGACAGCGACTGGTCCACGTGTTCAGACACGGACGATGAATTAKGACTAGATAGGGCAGGTGGGTACATATTCTCATCTGACACCGGCCCCGGCCACCTGCAACAGAGGTCTGTCCGTCAGACAGTACTGCCGGTAAATACCTTGGAGGAAGTTCAGGAGGAGAAATGTTACCCACCTAAGTTGGATGAAGTGAAAGAGCAGTTGTTACTTAAGAAACTCCAAGAAAGTGCGTCCATGGCCAACAGAAGCAGGTACCAATCCCGCAAAGTAGAGAACACGAAAGCAACAATAGTCCAAAGGCTGAAGGGTGGCTGCAAACTTTATTTAATGTCGGAGACCCCGAAAGTTCCTACTTACCGAACTACATATCCGGCACCAGTGTACTCACCCCCAATCAATATCCGACTGTCCAACCCTGAGTCTGCTGTGGCAGCGTGCAATGAGTTCCTAGCAAGGAACTACCCGACAGTTGCGTCGTACCAAATCACCGATGAGTACGATGCATACCTGGATATGGTGGACGGGTCGGAAAGTTGCCTTGACCGGGCGACGTTTAATCCATCAAAGCTTAGAAGTTATCCAAAACAGCACTCCTACCATGCACCTACAATCAGAAGTGCCGTACCTTCCCCGTTCCAGAACACGCTGCAGAACGTACTGGCTGCTGCCACGAAAAGAAATTGCAACGTCACACAGATGAGAGAACTGCCTACTTTGGATTCAGCGGTATTTAATGTTGAGTGCTTTAAAAAATTTGCGTGCAATCAAGAATACTGGAAGGAATTTGCCGCCAGTCCTATTAGGATAACGACTGAGAACTTGACAACTTATGTCACAAAACTAAAAGGACCAAAAGCAGCAGCTTTATTTGCCAAGACACATAACCTGCTACCACTGCAGGAGGTGCCGATGGACAGGTTTACTGTAGACATGAAAAGGGATGTGAAGGTGACTCCGGGGACGAAGCACACTGAGGAAAGACCTAAAGTGCAGGTCATACAGGCAGCCGAACCTTTGGCAACAGCATATCTGTGTGGGATCCACAGAGAGTTGGTTAGAAGACTGAATGCAGTTCTTCTACCTAATGTACACACGCTGTTTGACATGTCTGCCGAGGACTTTGACGCCATTATTGCCGCGCACTTCAAGCCGGGGGACGCCGTATTGGAAACCGATATAGCCTCCTTTGACAAGAGCCAAGACGACTCATTGGCGCTCACTGCTCTAATGTTGCTAGAGGATTTGGGGGTGGATCATCCCCTGTTGGACTTGATAGAGGCTGCCTTCGGGGAGATCTCCAGCTGCCACCTACCGACGGGCACCCGTTTTAAGTTCGGCGCCATGATGAAGTCTGGTATGTTCCTAACCCTGTTCGTCAACACACTGCTAAACATCACCATAGCCAGCCGGGTGCTGGAGGACCGCTTGACAAAGTCTGCGTGCGCGGCCTTCATCGGCGACGACAATATAATACATGGGGTTGTCTCTGACGAACTGATGGCAGCAAGATGTGCTACATGGATGAACATGGAAGTGAAGATCATAGATGCGGTCGTGTCTCAGAAAGCCCCGTACTTCTGCGGAGGGTTTATACTGTATGACACAGTAGCAGGCACGGCCTGCAGAGTGGCAGACCCGTTAAAGCGGCTGTTCAAGCTGGGCAAACCGCTGGCGGCGGGAGATGAACAAGACGACGATAGAAGACGTGCACTGGCTGACGAAGTGGTTAGATGGCAACGAACAGGGCTAACTGATGAGCTAGAAAAAGCGGTACACTCCAGGTATGAAGTGCAGGGCATATCTGTCGTGGTAATGTCTATGGCCACCTTTGCAAGCTCTAGATCTAACTTTGAAAAGCTCAGGGGACCCATCGTAACCCTGTACGGTGGTCCTAAATAGGTACGCACTACAGCTACCTATTTCGTCAGAAACCAATCGCAGCTACTCGCACACCTACCAGCTACAATGGAGTTCATCCCGACGCAAACTTTCTATAACAGAAGGTACCAACCCCGACCCTGGGCCCCACGCCCTACAATTCAAGTAATTAGACCTAGACCACGTCCACAGAGGCAGGCAGGGCAACTCGCCCAGCTGATCTCTGCAGTCAATAAATTGACCATGCGCGCGGTACCTCAACAGAAGCCTCGCAGAAATCGGAAAAACAAGAAGCAAAGGCAGAAGAAGCAGGCGTCGCAAAACGACCCAAAGCAAAAGAAGCAACCACCACAAAAGAAGCCGGCTCAAAAGAAGAAGAAACCAGGCCGTAGGGAGAGAATGTGCATGAAAATTGAAAATGATTGCATCTTCGAAGTCAAGCATGAAGGCAAAGTGATGGGCTACGCATGCCTGGTGGGGGATAAAGTAATGAAACCAGCACATGTGAAGGGAACTATCGACAATGCCGATCTGGCTAAACTGGCCTTCAAGCGGTCATCTAAATACGATCTTGAATGTGCACAGATACCAGTGCACATGAAGTCTGATGCCTCGAAGTTCACCCACGAGAAACCCGAGGGGTACTATAACTGGCATCACGGAGCAGTGCAGTATTCAGGAGGCCGGTTCACTATCCCGACGGGTGCAGGCAAGCCGGGAGACAGCGGCAGACCGATCTTCGACAACAAAGGACGGGTGGTGGCCATCGTCCTAGGAGGGGCCAACGAAGGTGCCCGCACGGCCCTCTCCGTGGTGACGTGGAACAAAGACATCGTCACAAAAATTACCCCTGAGGGAGCCGAAGAGTGGAGCCTCGCCCTCCCGGTCTTGTGCCTGTTGGCAAACACTACATTCCCCTGCTCTCAGCCACCTTGCACACCCTGCTGCTACGAAAAGGAACCGGAAAGCACCTTGCGCATGCTTGAGGACAACGTGATGAGACCCGGATACTACCAGCTGCTAAAAGCATCGCTGACTTGCTCTCCCCACCGCCAAAGACGCAGTACTAAGGACAATTTTAATGTCTATAAAGCCACAAGACCATACCTAGCTCATTGTCCTGACTGCGGAGAAGGGCATTCGTGCCATAGCCCTATCGCATTGGAGCGCATTAGAAATGAAGCAACGGACGGAACGCTGAAAATCCAGGTCTCTTTGCAGATCGGGATAAAGACAGATGACAGCCACGATTGGACTAAGCTGCGCTATATGGATAGCCACACGCCAGCGGACGCGGAGCGAGCCGGACTGCTTGTAAGGACTTCAGCACCGTGCATGATCACCGGGACCATGGGACACTTTATTCTCGCCCGATGCCCGAAAGGAGAGACGCTGACAGTGGGATTTACGGACAGCAGAAAGATCAGCCACACATGCACACACCCGTTCCATCATGAACCACCTGTGATAGGTAGGGAGAGGTTCCACTCTCGACCACAACATGGTAAAGAGTTACCTTGCAGCACGTACGTGCAGAGCACCGCTGCCACTGCCGAAGAGATAGAGGTGCATATGCCCCCAGATACTCCTGACCGCACGCTGATGACACAGCAGTCTGGCAACGTGAAGATCACAGTTAATGGGCAGACGGTGCGGTACAAGTGCAACTGCGGTGGCTCAAACGAGGGACTGACAACCACAGACAAAGTGATCAATAATTGCAAAATTGATCAGTGCCATGCTGCAGTCACTAATCACAAGAAGTGGCAATACAACTCCCCTTTGGTCCCGCGCAACGCTGAACTCGGGGACCGTAAAGGAAAGATTCACATCCCATTCCCATTGGCAAACGTGACTTGCAGAGTGCCAAAAGCAAGAAACCCCACAGTAACGTACGGAAAAAACCAAGTCACCATGCTGCTGTATCCTGACCATCCGACACTCTTGTCTTATCGTAACATGGGACAGGAACCAAATTACCACGAGGAGTGGGTGACACACAAGAAGGAGGTCACCTTGACCGTGCCTACTGAGGGTCTGGAGGTCACTTGGGGCAACAACGAACCATACAAGTACTGGCCGCAGATGTCTACGAACGGTACTGCTCATGGTCATCCACATGAGATAATCTTGTACTATTATGAGCTGTACCCCACTATGACTGTAGTCATTGTGTCGGTGGCCTCGTTCGTGCTTCTGTCGATGGTGGGCACAGCAGTGGGGATGTGTGTATGCGCACGGCGCAGATGCATTACACCATATGAATTAACACCAGGAGCCACTGTTCCCTTTCTGCTCAGCCTGCTATGCTGCGTCAGAACGACCAAGGCGGCCACATATTACGAGGCTGCGGCATACCTATGGAACGAACAGCAGCCCCTGTTCTGGTTGCAGGCTCTTATCCCGCTGGCCGCCTTGATCGTCCTGTGCAACTGTCTGAGGCTCTTGCCATGCTGCTGTAAGACCCTGGCTTTTTTAGCCGTAATGAGCATCGGTGCCCACACTGTGAGCGCGTACGAACACGTAACAGTGATCCCGAACACGGTGGGAGTACCGTATAAGACTCTTGTCAACAGACCGGGTTACAGCCCCATGGTATTGGAGATGGAGCTACAATCAGTTACCTTGGAACCAACATTGTCACTTGACTACATCACGTGCGAGTACAAAACTGTCATCCCCTCCCCGTACGTGAAGTGCTGTGGTACAGCAGAGTGCAAGGACAAGAGCCTACCAGACTACAGTTGCAAGGTCTTTACTGGAGTCTACCCATTTATGTGGGGCGGCGCCTATTGCTTTTGCGACGCCGAAAATACGCAATTGAGCGAGGCACACGTAGAGAAATCTGAATCTTGCAAAACAGAGTTTGCATCGGCCTACAGAGCCCACACCGCATCGGCGTCGGCGAAGCTCCGCGTCCTTTACCAAGGAAACAACATTACCGTAGCTGCCTACGCTAACGGCGACCATGCCGTCACAGTAAAGGACGCCAAGTTTGTCGTGGGCCCAATGTCCTCCGCCTGGACACCTTTTGACAACAAAATCGTGGTGTACAAAGGCGACGTCTACAACATGGACTACCCACCTTTTGGCGCAGGAAGACCAGGACAATTTGGTGACATTCAAAGTCGTACACCGGAAAGTAAAGACGTTTATGCCAACACTCAGTTGGTACTACAGAGGCCAGCAGCAGGCACGGTACATGTACCATACTCTCAGGCACCATCTGGCTTCAAGTATTGGCTGAAGGAAAGAGGAGCATCGCTACAGCACACGGCACCGTTCGGTTGCCAGATTGCGACAAACCCGGTAAGAGCTATAAATTGCGCTGTGGGGAACATACCAATTTCCATCGACATACCGGATGCGGCCTTTACTAGGGTTGTCGATGCACCCTCTGTAACGGACATGTCATGCGAAGTACCAGCCTGCACTCACTCCTCCGACTTTGGGGGCGTCGCCATCATCAAATACACAGCTAGCAAGAAAGGTAAATGTGCAGTACATTCGATGACCAACGCCGTTACCATTCGAGAAGTCGACGTAGAAGTAGAGGGGAACTCCCAGCTGCAAATATCCTTCTCAACAGCCCTGGCAAGCGCCGAGTTTCGCGTGCAAGTGTGCTCCACACAAGTACACTGCGCAGCCGCATGCCACCCTCCAAAGGACCACATAGTCAATTACCCAGCATCACACACCACCCTTGGGGTCCAGGATATATCCACAACGGCAATGTCTTGGGTGCAGAAGATTACGGGAGGAGTAGGATTAATTGTTGCTGTTGCTGCCTTAATCTTAATTGTGGTGCTATGCGTGTCGTTTAGCAGGCACTAAACCGATGATAAGGCACGAAGTAACTAAATAGCAAAAGTAGAAAGTACATAATCAGGTATATGTGCCCCCTAAGAGGCACAATATATATAGCTAAGCACTATTAGATCAAAGGGCTATACAACCCCTGAATAGTAACAAAACACAAAAATCAATAAAAATCATAAAAAGAAAATCTCATAAACAGGTATAAGTGTCCCCTAAGAGACACATTGTATGTAGGTAGTAAGTATAGATCAAAGGGCTATATTAACCCCTGAATAGTAACAAAACACAAAAACAATAAAAACTACAAAATAGAAAATCTATAAACAAAAGTAGTTCAAAGGGCTACAAAACCCCTGAATAGTAACAAAACATAAAATGTAATAAAAATTAAGTGTGTACCCGAAAGAGGTACAGTAAGAATCAGTGAATACCACAATTGGCGACGAGAAGAGACGTAGGTATTTAAGCTTCCTAAAAGCAGCCGAACTCACTTTGAGACGTAGGCATAGCATACCGAACTCTTCCACTATTCTCCGAACCCATAGGGAC

>KJ679577|2011|India, Group 3

GAGACACACGTAGCCTACCAGTTTCTTACTGCTCTACTCTGCAAAGCAAGAGATTAAGAACCCATCATGGATCCTGTGTACGTGGACATAGACGCTGACAGCGCCTTTTTGAAGGCCCTGCAACGTGCGTACCCCATGTTTGAGGTGGAACCTAGGCAGGTCACACCGAATGACCATGCTAATGCTAGAGCGTTCTCGCATCTAGCTATAAAACTAATAGAGCAGGAAATTGATCCCGACTCAACCATCCTGGATATTGGTAGTGCGCCAGCAAGGAGGATGATGTCGGACAGGAAGTACCACTGCGTTTGCCCGATGCGCAGTGCAGAAGATCCCGAGAGACTCGCCAATTATGCGAGAAAGCTAGCATCTGCCGCAGGAAAAGTCCTGGACAGAAACATCTCTGGAAAGATCGGGGACTTACAAGCAGTAATGGCCGTGCCAGACACGGAGACGCCAACATTCTGCTTACACACAGATGTATCATGTAGACAGAGAGCAGACGTCGCGATATACCAAGACGTCTATGCTGTACACGCACCCACGTCGCTATACCACCAGGCGATTAAAGGGGTCCGATTGGCGTACTGGGTAGGGTTTGACACAACCCCGTTCATGTACAATGCCATGGCGGGTGCCTACCCCTCATACTCGACAAATTGGGCAGATGAGCAGGTACTGAAGGCTAAGAACATAGGATTATGTTCAACAGACCTGACGGAAGGTAGACGAGGCAAATTGTCTATTATGAGAGGAAAAAAGCTAGAACCGTGCGACCGTGTGCTGTTCTCAGTAGGGTCAACGCTCTACCCGGAAAGCCGTAAGCTACTTAAGAGCTGGCACCTACCCTCGGTGTTCCATTTAAAGGGCAAGCTCAGCTTCACATGCCGCTGTGATACAGTGGTTTCGTGCGAAGGCTACGTCGTTAAGAGAATAACGATGAGCCCAGGCCTTTACGGAAAAACCACAGGGTATGCGGTAACCCACCACGCAGACGGATTCCTGATGTGCAAGACCACCGACACGGTTGACGGCGAAAGAGTGTCATTCTCGGTGTGCACGTACGTGCCGGCGACCATTTGTGATCAAATGACCGGCATCCTTGCTACAGAAGTCACGCCGGAGGATGCACAGAAGCTGTTGGTGGGGCTGAACCAGAGAATAGTGGTTAACGGCAGAACGCAACGGAATACGAACACCATGAAAAACTATATGATTCCCGTGGTCGCCCAAGCCTTCAGTAAGTGGGCAAAGGAGTGCCGGAAAGACATGGAAGATGAAAAACTCCTGGGGGTCAGAGAAAGAACACTGACCTGCTGCTGTCTATGGGCATTTAAGAAGCAGAAAACACACACGGTCTACAAGAGGCCTGATACCCAGTCAATTCAGAAGGTTCAGGCCGAGTTTGACAGCTTTGTGGTACCGAGCCTGTGGTCGTCCGGGTTGTCAATCCCGTTGAGGACTAGAATCAAATGGTTGTTAAGCAAGGTGCCAAAAACCGACCTGACCCCATACAGCGGGGACGCCCAAGAAGCCCGGGACGCAGAAAAAGAAGCAGAGGAAGAACGAGAAGCAGAACTGACTCTTGAAGCCCTACCACCCCTTCAGGCAGCACAGGAAGATGTTCAGGTCGAAATCGACGTGGAACAGCTTGAGGACAGAGCGGGTGCAGGAATAATAGAGACTCCGAGAGGAGCTATCAAAGTTACTGCCCAACCAACAGACCACGTCGTGGGAGAGTACTTGGTTCTTTCCCCGCAGACCGTACTACGTAGCCAAAAGCTTAGCCTGATTCACGCTTTGGCGGAGCAAGTGAAGACGTGCACGCACAGCGGACGAGCAGGGAGGTATGCGGTCGAAGCGTACGACGGCAGAGTCCTAGTGCCCTCAGGCTACGCAATCTCGCCTGAAGACTTCCAGAGCCTAAGCGAAAGCGCAACGATGGTGTACAACGAAAGAGAGTTCGTAAACAGAAAGCTACACCATATTGCGATGCATGGACCAGCCCTGAACACCGACGAAGAGTCGTATGAGCTGGTGAGGGCAGAGAGGACAGAACACGAGTACGTCTACGACGTGGACCAGAGAAGATGCTGTAAGAAGGAAGAAGCTGCAGGACTGGTACTGGTGGGCGACTTGACTAATCCGCCCTACCACGAATTCGCATATGAAGGGCTAAAAATCCGCCCTGCCTGCCCATACAAAATTGCAGTCATAGGAGTCTTCGGAGTACCAGGATCTGGCAAGTCAGCTATTATCAAGAACCTAGTTACCAGGCAAGACCTGGTGACTAGCGGAAAGAAAGAAAACTGCCAAGAAATCACCACCGACGTGATGAGACAGAGAGGTCTAGAGATATCTGCACGTACGGTTGACTCGCTGCTCTTGAATGGATGTAACAGACCAGTCGACGTGTTGTACGTAGACGAGGCGTTTGCGTGCCACTCTGGAACGTTACTTGCATTGATCGCCTTGGTGAGACCAAGACAGAAAGTTGTACTTTGTGGTGACCCGAAGCAGTGCGGCTTCTTCAATATGATGCAGATGAAAGTCAACTATAATCACAACATCTGCACCCAAGTGTACCACAAAAGTATCTCCAGGCGGTGTACACTGCCTGTGACTGCCATTGTGTCATCGTTGCATTACGAAGGCAAAATGCGCACTACGAATGAGTACAACAAGCCGATTGTAGTGGACACTACAGGCTCAACAAAACCTGACCCTGGAGATCTCGTGTTAACGTGCTTCAGAGGATGGGTTAAACAACTGCAAATTGACTATCGTGGACACGAGGTCATGACAGCAGCCGCATCCCAAGGGTTAACCAGAAAAGGAGTTTACGCAGTTAGGCAAAAAGTTAACGAAAACCCGCTTTATGCATCAACGTCAGAGCACGTCAACGTACTCCTAACGCGTACGGAAGGTAAACTGGTATGGAAGACACTCTCCGGTGACCCGTGGATAAAGACGCTGCAGAACCCACCGAAAGGAAACTTCAAAGCAACTATTAAGGAGTGGGAGGTGGAGCATGCATCAATAATGGCGGGCATCTGCAGTCACCAAATGACCTTTGATACATTCCAAAACAAAGCCAACGTTTGTTGGGCTAAGAGTTTGGTCCCTATCCTCGAAACAGCGGGGATAAAACTAAACGACAGGCAGTGGTCCCAGATAATTCAAGCCTTCAAAGAAGACAAAGCATATTCACCCGAAGTAGCCCTGAATGAAATATGCACGCGCATGTATGGGGTGGATCTAGACAGCGGGCTATTTTCTAAACCGTTGGTGTCTGTGTATTACGCGGATAACCACTGGGATAATAGGCCTGGAGGGAAGATGTTCGGATTCAACCCCGAGGCAGCATCCATTCTAGAAAGAAAGTATCCATTTACAAAAGGGAAGTGGAACATCAACAAGCAGATCTGCGTGACTACCAGGAGGATAGAAGACTTCAACCCTACCACCAACATTATACCGGCCAACAGGAGACTACCACACTCATTAGTGGCCGAACACCGCCCAGTAAAAGGGGAAAGAATGGAATGGCTGGTTAACAAGATAAACGGCCACCACGTGCTCCTGGTCAGTGGCTGTAGCCTTGCACTGCCTACTAAGAGAGTCACTTGGGTAGCGCCACTAGGTGTCCGCGGAGCGGACTATACATACAACCTAGAGTTGGGTCTGCCAGCAACGCTTGGTAGGTATGACCTAGTGGTCATAAACATCCACACACCTTTTCGCATACACCATTATCAACAGTGCGTAGACCACGCAATGAAACTGCAAATGCTCGGGGGTGACTCATTGAGACTGCTCAAACCGGGTGGCTCTCTATTGATCAGAGCATATGGTTACGCAGATAGAACCAGTGAACGAGTCATCTGCGTATTGGGACGCAAGTTTAGATCATCTAGAGCGTTGAAACCACCATGTGTCACCAGCAACACTGAGATGTTTTTTCTATTCAGCAACTTTGACAATGGCAGAAGGAATTTCACAACTCATGTCATGAACAATCAACTGAATGCAGCCTTTGTAGGACAGGCCACCCGAGCAGGATGTGCACCGTCGTACCGGGTAAAACGCATGGATATCGCGAAGAACGATGAAGAGTGCGTAGTCAACGCCGCCAACCCTCGCGGGTTACCAGGTGACGGTGTTTGCAAGGCAGTATACAAAAAATGGCCGGAGTCCTTTAAGAACAGTGCAACACCAGTGGGAACCGCAAAAACAGTCATGTGCGGTACGTATCCAGTAATCCACGCCGTTGGACCAAACTTCTCTAATTATTCGGAGTCTGAAGGGGACCGAGAATTGGCGGCTGCCTATCGAGAAGTCGCAAAGGAGGTAACTAGACTGGGAGTAAATAGTGTAGCTATACCTCTCCTCTCCACAGGTGTATACTCAGGAGGGAAAGACAGGCTGACCCAGTCACTGAACCACCTCTTTACAGCCATGGACTCGACGGATGCAGACGTGGTCATCTACTGCCGCGACAAAGAATGGGAGAAGAAAATATCTGAGGCCATACAGATGCGGACCCAAGTGGAGCTGCTGGATGAGCACATCTCCATAGACTGCGATGTTGTTCGCGTGCACCCTGACAGCAGCTTGGCAGGCAGAAAAGGATACAGCACCACGGAAGGCGCACTGTACTCATATCTAGAAGGGACCCGTTTTCACCAAACGGCAGTGGATATGGCAGAGATATATACTATGTGGCCAAAGCAAACAGAGGCCAACGAGCAAGTTTGCCTATATGCCCTGGGGGAAAGTATTGAATCGATCAGGCAGAAATGCCCGGTGGATGATGCAGATGCATCATCTCCCCCGAAAACTGTCCCGTGCCTCTGCCGTTACGCCATGACACCAGAACGCGTTACCCGACTTCGCATGAACCATGTCACAAGCATAATTGTGTGTTCTTCGTTTCCCCTTCCAAAGTACAAAATAGAAGGAGTGCAAAAAGTCAAATGCTCCAAGGTAATGCTATTTGACCACAACGTGCCATCGCGCGTAAGTCCAAGGGAATACAGACCTTCCCAGGAGTCTGTACAGGAAGCGAGTACGACCACGTCACTGACGCATAGCCAATTCGATCTAAGCGTTGACGGCAAGATACTGCCCGTCCCGTCAGACCTGGATGCTGACGCCCCAGCCCTAGAACCAGCCCTTGACGACGGGGCGATACACACGTTGCCATCTGCAACCGGAAACCTTGCGGCCGTGTCTGACTGGGTAATGAGCACCGTACCTGTCGCGCCGCCCAGAAGAAGGCGAGGGAGAAACCTGACTGTGACATGCGACGAGAGAGAAGGGAATATAACACCCATGGCTAGCGTCCGATTCTTTAGGGCAGAGCTGTGTCCAGTCGTACAAGAAACAGCGGAGACGCGTGACACAGCTATGTCTCTTCAGGCACCGCCGAGTACCGCCACGGAACTGAGTCACCCGCCGATCTCCTTCGGTGCACCAAGCGAGACGTTCCCCATCACATTTGGGGACTTCAACGAAGGAGAAATCGAAAGCTTGTCTTCTGAGCTACTAACTTTCGGAGACTTCCTACCCGGAGAAGTGGATGATTTGACAGATAGCGACTGGTCCACGTGCTCAGACACGGACGACGAGTTACGACTAGACAGGGCAGGTGGGTATATATTCTCGTCGGACACTGGTCCAGGTCATTTACAACAGAAGTCAGTACGCCAGTCAGTGCTGCCGGTGAACACCCTGGAGGAAGTCCACGAGGAGAAGTGTTACCCACCTAAGCTGGATGAAGCAAAGGAGCAACTACTACTTAAGAAACTCCAGGAGAGTGCATCCATGGCCAACAGAAGCAGGTATCAGTCGCGCAAAGTAGAAAACATGAAAGCAACAATCATCCAGAGACTAAAGAGAGGCTGTAGATTATACTTAATGTCAGAGACCCCAAAAGTCCCTACCTACCGGACCACATATCCGGCGCCTGTGTACTCGCCTCCGATTAACGTCCGACTGTCCAACCCCGAGTCCGCAGTGGCAGCATGCAATGAGTTCTTGGCTAGAAACTATCCAACTGTTTCATCATACCAAATCACCGACGAGTATGATGCATATCTAGACATGGTGGACGGGTCGGAGAGTTGTCTGGACCGAGCGACATTCAATCCGTCAAAACTTAGGAGCTACCCAAAACAGCACGCTTACCACGCGCCCTCCATCAGAAGCGCTGTACCGTCCCCATTCCAGAACACACTACAGAATGTACTGGCAGCAGCCACGAAAAGAAACTGCAACGTCACACAGATGAGGGAATTACCCACTTTGGACTCAGCAGTATTCAACGTGGAGTGTTTCAAAAAATTCGCATGCAACCAAGAATACTGGGAAGAATTTGCTGCCAGCCCTATCAGGATAACAACTGAGAATTTAACAACCTATGTTACTAAACTAAAGGGGCCAAAAGCAGCAGCGCTATTTGCAAAAACCCATAATCTGCTGCCACTGCAGGAAGTGCCAATGGATAGGTTCACAGTAGACATGAAAAGGGATGTGAAGGTGACTCCTGGTACAAAGCACACAGAGGAAAGACCTAAGGTACAGGTTATACAGGCGGCTGAACCCTTGGCAACAGCATACCTATGTGGGATTCACAGAGAGCTGGTTAGGAGGCTGAACGCCGTCCTCCTACCCAATGTACATACACTATTTGACATGTCTGCCGAGGATTTCGATGCCATCATAGCCGCACACTTTAAGCCAGGAGACACTGTTTTAGAAACGGACATAGCCTCCTTTGATAAGAGCCAAGATGATTCACTTGCGCTTACTGCTTTAATGCTGTTAGAGGATTTAGGGGTGGATCACTCCCTGTTGGACTTGATAGAGGCTGCTTTCGGAGAGATTTCCAGCTGTCATCTACCGACAGGTACGCGCTTCAAGTTCGGCGCCATGATGAAATCTGGTATGTTCCTAACTCTGTTCGTCAACACACTGCTAAATATCACCATCGCCAGCCGAGTGCTGGAAGATCGTCTGACAAAATCCGCGTGCGCAGCCTTCATCGGCGACGACAACATAATACATGGAGTCGTCTCCGATGAATTGATGGCAGCCAGATGCGCCACTTGGATGAACATGGAAGTGAAGATCATAGATGCAGTTGTATCCCAGAAAGCCCCTTACTTTTGTGGAGGGTTTATACTGCACGATATCGTGACAGGAACAGCTTGCAGAGTGGCAGACCCGCTAAAAAGGCTATTTAAACTGGGCAAACCGCTAGCGGCAGGTGACGAACAAGATGAGGATAGAAGACGAGCGCTGGCTGACGAAGTGGTCAGATGGCAACGAACAGGGCTAATTGATGAGTTGGAGAAAGCGGTATACTCTAGGTATGAAGTGCAGGGTATATCAGTTGTGGTAATGTCCATGGCCACCTTTGCAAGCTCCAGATCCAACTTCGAGAAGCTCAGAGGACCCGTCGTAACTTTGTACGGCGGTCCTAAATAGGTACGCACTACAGCTACCTATTTTGCAGAAGCCGACAGTAAGTACCTAAACACTAATCAGCTACAATGGAGTTCATCCCAACCCAAACTTTTTACAACAGGAGGTACCAGCCTCGACCCTGGACTCCGCGCCCTACTATCCAAGTCATCAGGCCCAGACCGCGCCCGCAGAGGCAAGCTGGGCAACTTGCCCAGCTGATCTCAGCAGTTAATAAACTGACAATGCGCGCGGTACCCCAACAGAAGCCACGCAGGAATCGGAAGAATAAGAAGCAAAAGCAAAAGCAACAGGCGCCACAAAACAACACAAACCAAAAGAAGCAGCCACCTAAAAAGAAACCAGCTCAAAAGAAAAAGAAGCCGGGCCGCAGAGAGAGGATGTGCATGAAAATCGAAAATGACTGTATTTTCGAAGTCAAGCACGAAGGTAAGGTAACAGGTTACGCGTGCTTGGTGGGGGACAAAGTAATGAAACCAGCACACGTAAAGGGGACCATCGATAACGCGGACCTGGCCAAATTGGCCTTTAAGCGGTCATCTAAGTACGACCTTGAATGCGCGCAGATACCCGTGCACATGAAGTCCGACGCTTCGAAGTTCACCCATGAGAAACCGGAGGGGTACTACAACTGGCACCACGGAGCAGTACAGTACTCAGGAGGCCGGTTCACCATCCCTACAGGTGCGGGCAAACCAGGGGACAGCGGTAGACCGATCTTCGACAACAAGGGACGCGTGGTGGCCATAGTCTTAGGAGGAGCTAATGAAGGAGCCCGTACAGCCCTCTCAGTGGTGACCTGGAATAAAGACATTGTCACTAAAATCACCCCTGAGGGAGCCGAAGAGTGGAGTCTTGCCATCCCAGTTATGTGCCTGTTGGCAAATACCACGTTCCCCTGCTCCCAGCCCCCTTGCATACCCTGCTGCTACGAAAAGGAACCGGAGGAAACCCTACGCATGCTTGAGGACAACGTCATGAGACCTGGGTACTATCAGCTGCTACAAGCATCATTAACATGTTCTCCCCACCGCCAGCGACGCAGCACCAAGGACAACTTCAATGTCTATAAAGCCACAAGACCATACTTAGCTCACTGTCCCGACTGTGGAGAAGGGCACTCGTGCCATAGTCCCGTAGCACTAGAACGCATCAGAAATGAAGCGACAGACGGGACGCTGAAAATCCAGGTCTCCTTGCAAATTGGAATAGGGACGGATGATAGCCATGATTGGACCAAGCTGCGTTACATGGACAATCACATACCAGCAGACGCAGGGAGGGCCGGGCTATTTGTAAGAACATCAGCACCATGCACGATTACTGGAACAATGGGACACTTCATCCTGGCCCGATGTCCGAAAGGAGAAACTCTGACGGTGGGATTCACTGACAGTAGGAAGATTAGTCACTCATGTACGCACCCATTTCACCACGACCCTCCTGTGATAGGCCGGGAAAAATTCCATTCCCGACCGCAGCACGGTAAAGAGCTACCTTGCAGCACGTACGTGCAGAGCAACGCCGCAACTGCCGAGGAGATAGAGGTACACATGCCCCCAGACACCCCTGATCGCACATTGCTGTCACAACAGTCCGGCAACGTAAAGATCACAGTCAACGGCCGGACGGTGCGGTATAAGTGTAATTGCGGTGGCTCAAATGAAGGACTAATAACTACAGATAAAGTGATTAATAACTGCAAGGTTGATCAATGTCATGCCGCGGTCACCAATCACAAAAAGTGGCAGTATAACTCCCCTCTGGTCCCGCGTAACGCTGAACTCGGGGACCGAAAAGGAAAAATTCACATCCCGTTTCCGCTGGCAAATGTAACATGCATGGTGCCTAAAGCAAGGAACCCCACCGTGACGTACGGGAAAAACCAAGTCATCATGCTACTGTATCCTGACCACCCAACACTCCTGTCCTACCGGAGTATGGGAGAAGAACCAAACTATCAAGAAGAGTGGGTGACGCACAAGAAGGAGGTCGTGCTAACCGTGCCGACTGAAGGGCTCGAGGTTACGTGGGGCAACAACGAGCCGTATAAGTATTGGCCGCAGTTATCTGCAAACGGTACAGCCCACGGCCACCCGCATGAGATAATCTTGTACTATTATGAGCTGTACCCTACTATGACTGTAGTAGTTGTGTCAGTGGCCTCGTTCATACTCCTGTCGATGGTGGGTATGGCAGTGGGGATGTGCATGTGTGCACGACGCAGATGCATCACACCATACGAACTGACACCAGGAGCTACCGTCCCTTTCCTGCTTAGCCTAATATGCTGCATCAGAACAGCTAAAGCGGCCACATACCAAGAGGCTGCGGTATACCTGTGGAACGAGCAGCAACCTTTGTTTTGGCTACAAGCCCTTATTCCGCTGGCAGCCCTGATTGTCCTATGCAACTGTCTGAGACTCTTACCATGCTGTTGTAAAACGTTGGCTTTTTTAGCCGTAATGAGCATCGGTGCCCACACTGTGAGCGCGTACGAACACGTAACAGTGATCCCGAACACGGTGGGAGTACCGTATAAGACTCTAGTCAACAGACCGGGCTACAGCCCCATGGTACTGGAGATGGAGCTACTGTCAGTCACTTTGGAGCCAACGCTATCGCTTGATTACATCACGTGCGAATACAAAACCGTCATCCCGTCTCCGTACGTGAAATGCTGCGGTACAGCAGAGTGCAAGGACAAAAACCTACCTGACTACAGCTGTAAGGTCTTCACCGGCGTCTACCCATTTATGTGGGGCGGCGCCTACTGCTTCTGCGACGCTGAAAACACGCAATTGAGCGAAGCACATGTGGAGAAGTCCGAATCATGCAAAACAGAATTTGCATCAGCATACAGGGCTCATACCGCATCCGCATCAGCTAAGCTCCGCGTCCTTTACCAAGGAAATAACATCACTGTAACTGCCTATGCAAACGGCGACCATGCCGTCACAGTTAAGGACGCCAAATTCATTGTGGGGCCAATGTCTTCAGCCTGGACACCTTTCGACAACAAAATCGTGGTGTACAAAGGTGACGTTTACAACATGGACTACCCGCCCTTTGGCGCAGGAAGACCAGGACAATTTGGCGATATCCAAAGTCGCACGCCTGAGAGCAAAGACGTCTATGCTAACACACAACTGGTACTGCAGAGACCGGCTGCGGGTACGGTACACGTGCCATACTCTCAGGCACCATCTGGCTTTAAGTATTGGTTAAAAGAACGAGGGGCGTCGCTACAGCACACAGCACCATTTGGCTGCCAAATAGCAACAAACCCGGTAAGAGCGATGAACTGCGCCGTAGGGAACATGCCCATCTCCATCGACATACCGGATGCGGCCTTCACTAGGGTCGTCGACGCGCCCTCTTTAACGGACATGTCATGCGAGGTACCAGCCTGCACCCATTCCTCAGACTTTGGGGGCGTCGCCATTATTAAATATGCAGTCAGCAAGAAAGGCAAGTGTGCGGTGCATTCGATGACCAACGCCGTCACTATCCGGGAAGCTGAGATAGAAGTTGAAGGGAATTCTCAGCTGCAAATCTCTTTCTCGACGGCCTTGGCCAGCGCCGAATTCCGCGTACAAGTCTGTTCTACACAAGTACACTGTGCAGCCGAGTGCCACCCTCCGAAGGACCACATAGTCAACTACCCGGCGTCACATACCACCCTCGGGGTCCAGGACATTTCCGCTACGGCGATGTCATGGGTGCAGAAGATCACGGGAGGTGTGGGACTGGTTGTCGCTGTTGCAGCACTGATTCTAATCGTGGTGCTATGCGTGTCGTTCAGCAGGCACTAACTTGACGACTAAGCATGAAGGTATATGTGTCCCCTAAGAGACACACCGTATATAGCTAATAATCTGTAGATCAAAGGGCTATAAAAACCCCTGAATAGTAACAAAACATAAAACTAATAAAAATCAAATGAATACCATAATTGGCAAACGGAAGAGATGTAGGTACTTAAGCTTCCTAAAAGCAGCCGAACTCACTTTGAGATGTAGGCATAGCATACCGAACTCTTCCACGATTCTCCGAACCCATAGGGACGTAGGAGATGT

>FJ807898|2008|Bangladesh, Group 4

ATGGCTGCGTGAGACACACGTAGCCTACCAGTTTCTTACTGCTCTACTCTGCAAAGCAAGAGATTAATAACCCATCATGGATCCTGTGTACGTGGACATAGACGCTGACAGCGCCTTTTTGAAGGCCCTGCAACGTGCGTACCCCATGTTTGAGGTGGAACCAAGGCAGGTCACACCGAATGACCATGCTAATGCTAGAGCGTTCTCGCATCTAGCTATAAAACTAATAGAGCAGGAAATTGACCCCGACTCAACCATCCTGGATATCGGCAGTGCGCCAGCAAGGAGGATGATGTCGGACAGGAAGTACCACTGCGTCTGCCCGATGCGCAGTGCGGAAGATCCCGAGAGACTCGCTAATTATGCGAGAAAGCTAGCATCTGCCGCAGGAAAAGTCCTGGACAGAAACATCTCTGGAAAGATCGGGGACTTACAAGCAGTAATGGCCGTGCCAGACAAGGAGACGCCGACATTCTGCTTACACACAGACGTCTCATGTAGACAGAGAGCAGACGTCGCTATATACCAAGACGTCTATGCTGTACACGCACCCACGTCGCTATACCACCAGGCGATTAAAGGGGTCCGAGTGGCGTACTGGGTTGGGTTCGACACAACCCCGTTCACGTACAATGCCATGGCGGGTGCCTACCCCTCATACTCGACAAACTGGGCAGATGAGCAGGTACTGAAGGCTAAGAACATAGGATTATGTTCAACAGACCTGACGGAAGGTAGACGAGGCAAGTTGTCTATTATGAGAAGGAAAAAGCTAAAACCGTGCGACCGTGTGCTGTTCTCAGTAGGGTCAACGCTCTACCCGGAAAGCCGCAAGCTACTTAAGAGCTGGCACCTGCCATCGGTGTTCCATTTAAAGGGCAAACTCAGTTTCACATGCCGCTGTGATACAGTGGTTTCGTGTGAGGGCTACGTCGTTAAGAGAATAACGATGAGCCCAGGCCTTTATGGAAAAACCACAGGGTATGCGGTAACCCACCACGCAGACGGATTCCTGATGTGCAAGACTACCGACACGGTTGACGGCGAAAGAGTGTCATTCTCGGTGTGCACATACGTGCCGGCGACCATTTGTGATCAAATGACCGGCATCCTTGCTACAGAAGTCACGCCGGAGGATGCACAGAAGCTGTTGGTGGGGCTGAACCAGAGAATAGTGGTTAACGGCAGAACGCAACGGAATATGAACACCATGAAAAATTATCTGCTTCCCGTGGTCGCCCAAGCCTTCAGTAAGTGGGCAAAGGAGTGCCGGAAAGACATGGAAGATGAAAAACTCCTGGGGGTCAGAGAAAGAACACTGACCTGCTGCTGTCTATGGGCATTCAAGAAGCAGAAAACACACACGGTCTACAAGAGGCCTGATACCCAGTCAATTCAGAAGGTTCAGGCCGAGTTTGACAGCTTTGTGGTACCGAGTCTGTGGTCGTCCGGGTTGTCAATCCCTTTGAGGACTAGAATCAAATGGTTGTTAAGCAAGGTGCCAAAAACCGACCTGATCCCATACAGCGGAGACGCCCGAGAAGCCCGGGACGCAGAAAAAGAAGCAGAGGAAGAACGAGAAGCAGAACTGACTCGCGAAGCTCTACCACCTCTACAGGCAGCACAGGAAGATGTTCAGGTCGAAATCGACGTGGAACAGCTTGAGGACAGAGCGGGCGCAGGAATAATAGAGACTCCGAGAGGAGCTATCAAAGTTACTGCCCAACCAACAGACCACGTCGTGGGAGAGTACCTGGTACTCTCCCCGCAGACCGTACTACGTAGCCAGAAGCTCAGTCTGATTCACGCTTTGGCGGAGCAAGTGAAGACGTGCACGCACAACGGACGAGCAGGGAGGTATGCGGTCGAAGCGTACGACGGCCGAGTCCTAGTGCCCTCAGGCTATGCAATCTCGCCTGAAGACTTCCAGAGTCTAAGCGAAAGCGCAACGATGGTGTATAACGAAAGAGAGTTCGTAAACAGAAAGCTACACCATATTGCGATGCACGGACCAGCCCTGAACACCGACGAAGAGTCGTATGAGCTGGTGAGGGCAGAGAGGACAGAACACGAGTACGTCTACGACGTGGATCAGAGAAGATGCTGTAAGAAGGAAGAAGCCGCAGGACTGGTACTGGTGGGCGACTTGACTAATCCGCCCTACCACGAATTCGCATATGAAGGGCTAAAAATCCGCCCTGCCTGCCCATACAAAATTGCAGTCATAGGAGTCTTCGGAGTACCGGGATCTGGCAAGTCAGCTATTATCAAGAACCTAGTTACCAGGCAGGACCTGGTGACTAGCGGAAAGAAAGAAAACTGCCAAGAAATCACCACCGACGTGATGAGACAGAGAGGTCTAGAGATATCTGCACGTACGGTTGACTCGCTGCTCTTGAATGGATGCAACAGACCAGTCGACGTGTTGTACGTAGACGAGGCGTTTGCGTGCCACTCTGGAACGCTACTTGCTTTGATCGCCTTGGTGAGACCAAGGCAGAAAGTTGTACTTTGTGGTGACCCGAAGCAGTGCGGCTTCTTCAATATGATGCAGATGAAAGTCAACTATAATCACAACATCTGCACCCAAGTGTACCACAAAAGTATCTCCAGGCGGTGTACACTGCCTGTGACCGCCATTGTGTCATCGTTGCATTACGAAGGCAAAATGCGCACTACGAATGAGTACAACAAGCCGATTGTAGTGGACACTACAGGCTCAACAAAACCTGACCCTGGAGACCTCGTGTTAACGTGCTTCAGAGGGTGGGTTAAACAACTGCAAATTGACTATCGTGGATACGAGGTCATGACAGCAGCCGCATCCCAAGGGTTAACCAGAAAAGGAGTTTACGCAGTTAGACAAAAAGTTAATGAAAACCCGCTCTATGCATCAACGTCAGAGCACGTCAACGTACTCCTAACGCGTACGGAAGGTAAACTAGTATGGAAGACACTTTCCGGCGACCCGTGGATAAAGACGCTGCAGAACCCACCGAAAGGAAACTTCAAAGCAACTATTAAGGAGTGGGAGGTGGAGCATGCATCAATAATGGCGGGCATCTGCAGTCACCAAATGACCTTCGATACATTCCAAAATAAAGCCAACGTTTGTTGGGCTAAGAGCTTGGTCCCTATCCTCGAAACAGCGGGGATAAAACTAAATGATAGGCAGTGGTCTCAGATAATTCAAGCCTTCAAAGAAGACAAAGCATACTCACCTGAAGTAGCCCTGAATGAAATATGTACGCGCATGTATGGGGTGGATCTAGACAGCGGGCTATTTTCTAAACCGTTGGTGTCTGTGTATTACGCGGATAACCACTGGGATAATAGGCCTGGAGGGAAAATGTTCGGATTTAACCCCGAGGCAGCATCCATTCTAGAAAGAAAGTATCCATTCACAAAAGGGAAGTGGAACATCAACAAGCAGATCTGCGTGACTACCAGGAGGATAGAAGACTTTAACCCTACCACCAACATCATACCGGCCAACAGGAGACTACCACACTCATTAGTGGCCGAACACCGCCCAGTAAAAGGGGAAAGAATGGAATGGCTGGTTAACAAGATAAACGGCCACCACGTGCTCCTGGTCAGTGGCTATAACCTTGCACTGCCTACTAAGAGAGTCACTTGGGTAGCGCCGTTAGGTGTCCGCGGAGCGGACTACACATACAACCTAGAGTTGGGTCTGCCAGCAACGCTTGGTAGGTATGACCTTGTAGTCATAAACATCCACACACCTTTTCGCATACACCATTACCAACAGTGCGTCGACCACGCAATGAAACTGCAAATGCTCGGGGGTGACTCATTGAGACTGCTCAAACCGGGCGGCTCTCTATTGATCAGAGCATATGGTTACGCAGATAGAACCAGTGAACGAGTCATCTGCGTATTGGGACGCAAGTTTAGATCGTCTAGAGCGTTGAAACCACCATGTGTCACCAGCAACACTGAGATGTTTTTCCTATTCAGCAACTTTGACAATGGCAGAAGGAATTTCACAACTCATGTCATGAACAATCAACTGAATGCAGCCTTCGTAGGACAGGTCACCCGAGCAGGATGTGCACCGTCGTACCGGGTAAAACGCATGGACATCGCGAAGAACGATGAAGAGTGCGTAGTCAACGCCGCTAACCCTCGCGGGTTACCGGGTGACGGTGTTTGCAAGGCAGTATACAAAAAATGGCCGGAGTCCTTTAAGAACAGTGCAACACCAGTGGGAACCGCAAAAACAGTTATGTGCGGTACGTATCCAGTAATCCACGCTGTTGGACCAAACTTCTCTAATTATTCGGAGTCTGAAGGGGACCGGGAATTGGCAGCTGCCTATCGAGAAGTCGCAAAGGAAGTAACTAGGCTGGGAGTAAATAGTGTAGCTATACCTCTCCTCTCCACAGGTGTATACTCAGGAGGGAAAGACAGGCTGACCCAGTCACTGAACCACCTCTTTACAGCCATGGACTCGACGGATGCAGACGTGGTCATCTACTGCCGCGACAAAGAATGGGAGAAGAAAATATCTGAGGCCATACAGATGCGGACCCAAGTAGAGCTGCTGGATGAGCACATCTCCATAGACTGCGATATTGTTCGCGTGCACCCTGACAGCAGCTTGGCAGGCAGAAAAGGATACAGCACCACGGAAGGCGCACTGTACTCATATCTAGAAGGGACCCGTTTTCATCAGACGGCTGTGGATATGGCGGAGATACATACTATGTGGCCAAAGCAAACAGAGGCCAATGAGCAAGTCTGCCTATATGCCCTGGGGGAAAGTATTGAATCGATCAGGCAGAAATGCCCGGTGGATGATGCAGACGCATCATCTCCCCCCAAAACTGTCCCGTGCCTTTGCCGTTACGCTATGACTCCAGAACGCGTCACCCGGCTTCGCATGAACCACGTCACAAGCATAATTGTGTGTTCTTCGTTTCCCCTCCCAAAGTACAAAATAGAAGGAGTGCAAAAAGTCAAATGCTCTAAGGTAATGCTATTTGACCACAACGTGCCATCGCGCGTAAGTCCAAGGGAATATAGATCTTCCCAGGAGTCTGCACAGGAGGCGAGTACAATCACGTCACTGACGCATAGTCAATTCGACCTAAGCGTTGATGGCGAGATACTGCCCGTCCCGTCAGACCTGGATGCTGACGCCCCAGCCCTAGAACCAGCACTAGACGACGGGGCGACACACACGCTGCCATCCACAACCGGAAACCTTGCGGCCGTGTCTGACTGGGTAATGAGCACCGTACCTGTCGCGCCGCCCAGAAGAAGGCGAGGGAGAAACCTGACTGTGACATGTGACGAGAGAGAAGGGAATATAACACCCATGGCTAGCGTCCGATTCTTTAGGGCAGAGCTGTGTCCGGTCGTACAAGAAACAGCGGAGACGCGTGACACAGCAATGTCTCTTCAGGCACCACCGAGTACCGCCACGGAACCGAATCATCCGCCGATCTCCTTCGGAGCATCAAGCGAGACGTTCCCCATTACATTTGGGGACTTCAACGAAGGAGAAATCGAAAGCTTGTCTTCTGAGCTACTAACTTTCGGAGACTTCTTACCAGGAGAAGTGGATGACTTGACAGACAGCGACTGGTCCACGTGCTCAGACACGGACGACGAGTTATGACTAGACAGGGCAGGTGGGTATATATTCTCGTCGGACACCGGTCCAGGTCATTTACAACAGAAGTCAGTACGCCAGTCAGTGCTGCCGGTGAACACCCTGGAGGAAGTCCACGAGGAGAAGTGTTACCCACCTAAGCTGGATGAAGCAAAGGAGCAACTATTACTTAAGAAACTCCAGGAGAGTGCATCCATGGCCAACAGAAGCAGGTATCAGTCGCGCAAAGTAGAAAACATGAAAGCAGCAATCATCCAGAGACTAAAGAGAGGCTGTAGACTATACTTAATGTCAGAGACCCCAAAAGTCCCTACTTACCGGACTACATATCCGGCGCCTGTGTACTCGCCTCCGATCAACGTCCGATTGTCCAATCCCGAGTCCGCAGTGGCAGCATGCAATGAGTTCTTAGCTAGAAACTATCCAACTGTCTCATCATACCAAATTACCGACGAGTATGATGCATATCTAGACATGGTGGACGGGTCGGAGAGTTGCTTGGACCGAGCGACATTCAATCCGTCAAAACTCAGGAGCTACCCGAAACAGCACGCTTACCACGCGCCCTCCATCAGAAGCGCTGTACCGTCCCCATTCCAGAACACACTACAGAATGTACTGGCAGCAGCCACGAAAAGAAACTGCAACGTCACACAGATGAGGGAATTACCCACTTTGGACTCAGCAGTATTCAACGTGGAGTGTTTCAAAAAATTCGCATGCAACCAAGAATACTGGGAAGAATTTGCTGCCAGTCCTATTAGGATAACAACTGAGAATTTAGCAACCTATGTTACTAAACTAAAAGGGCCAAAAGCAGCAGCGCTATTCGCAAAAACCCATAATCTACTGCCACTACAGGAAGTACCAATGGATAGGTTCACAGTAGATATGAAAAGGGACGTGAAGGTGACTCCTGGTACAAAGCATACAGAGGAAAGACCTAAGGTGCAGGTTATACAGGCGGCTGAACCCTTGGCGACAGCATACCTATGTGGGATTCACAGAGAGCTGGTTAGGAGGCTGAACGCCGTCCTCCTACCCAATGTACATACACTATTTGACATGTCTGCCGAGGATTTCGATGCCATCATAGCCGCACACTTTAAGCCAGGAGACACTGTTTTGGAAACGGACATAGCCTCCTTTGATAAGAGCCAAGATGATTCACTTGCGCTTACTGCTTTGATGCTGTTAGAGGATTTAGGGGTGGATCACTCCCTGCTGGACTTGATAGAGGCTGCTTTCGGAGAGATTTCCAGCTGTCACCTACCGACAGGTACGCGCTTCAAGTTCGGCGCCATGATGAAATCAGGTATGTTCCTAACTCTGTTCGTCAACACATTGTTAAACATCACCATCGCCAGCCGAGTGCTGGAAGATCGTCTGACAAAATCCGCGTGCGCGGCCTTCATCGGCGACGACAACATAATACATGGAGTCGTCTCCGATGAATTGATGGCAGCCAGATGTGCCACTTGGATGAACATGGAAGTGAAGATCATAGATGCAGTTGTATCCTTGAAAGCCCCTTACTTTTGTGGAGGGTTTATACTGCACGATACTGTGACAGGAACAGCTTGCAGAGTGGCAGACCCGCTAAAAAGGCTTTTTAAACTGGGCAAACCGCTAGCGGCAGGTGACGAACAAGATGAAGATAGAAGACGAGCGCTGGCTGACGAAGTGATCAGATGGCAACGAACAGGGCTAATTGATGAGCTGGAGAAAGCGGTATACTCTAGGTACGAAGTGCAGGGTATATCAGTTGTGGTAATGTCCATGGCCACCTTTGCAAGCTCCAGATCCAACTTCGAGAAGCTCAGAGGACCCGTCATAACTTTGTACGGCGGTCCTAAATAGGTACGCACTACAGCTACCTATTTTGCAGAAGCCGACAGCAAGTATCTAAACACTAATCAGCTACAATGGAGTTCATCCCAACCCAAACTTTTTACAATAGGAGGTACCAGCCTCGACCCTGGACTCCGCGCTCTACTATCCAAGTCATCAGGCCCAGACCGCGCCCTCAGAGGCAAGCTGGGCAACTTGCCCAGCTGATCTCAGCAGTTAATAAACTGACAATGCGCGCGGTACCCCAACAGAAGCCACGCAGGAATCGGAAGAATAAGAAGCAAAAGCAAAAACAACAGGCGCCACAAAACAACACAAATCAAAAGAAGCAGCCACCTAAAAAGAAACCGGCTCAAAAGAAAAAGAAGCCGGGCCGCAGAGAGAGGATGTGCATGAAAATCGAAAATGATTGTATTTTCGAAGTCAAGCACGAAGGTAAGGTAACAGGTTACGCGTGCCTGGTGGGGGACAAAGTAATGAAACCAGCACACGTAAAGGGGACCATCGATAACGCGGACCTGGCCAAACTGGCCTTTAAGCGGTCATCTAAGTATGACCTTGAATGCGCGCAGATACCCGTGCACATGAAGTCCGACGCTTCGAAGTTCACCCATGAGAAACCGGAGGGGTACTACAACTGGCACCACGGAGCAGTACAGTACTCAGGAGGCCGGTTCACCATCCCTACAGGTGCTGGCAAACCAGGGGACAGCGGCAGACCGATCTTCGACAACAAGGGACGCGTGGTGGCCATAGTCTTAGGAGGAGCTAATGAAGGAGCCCGTACAGCCCTCTCGGTGGTGACCTGGAATAAAGACATTGTCACTAAAATCACCCCCGAGGGGGCCGAAGAGTGGAGTCTTGCCATCCCAGTTATGTGCCTGTTGGCAAACACCACGTTCCCCTGCTCCCAGCCCCCTTGCACGCCCTGCTGCTACGAAAAGGAACCGGAGGAAACCCTACGCATGCTTGAGGACAACGTCATGAGACCTGGGTACTATCAGCTGCTACAAGCATCCTTAACATGTTCTCCCCACCGCCAGCGACGCAGCACCAAGGACAACTTCAATGTCTATAAAGCCACAAGACCATACTTAGCTCACTGTCCCGACTGTGGAGAAGGGCACTCGTGCCATAGTCCCGTAGCACTAGAACGCATCAGAAATGAAGCGACAGACGGGACGCTGAAAATCCAGGTCTCCTTGCAAATCGGAATAAAGACGGATGACAGCCACGATTGGACCAAGCTGCGTTATATGGACAACCACATGCCAGCAGACGCAGAGAGGGCGGGGCTATTTGTAAGAACATCAGCACCGTGTACGATTACTGGAACAATGGGACACTTCATCCTGGCCCGATGTCCAAAAGGGGAAACTCTGACGGTGGGATTCACTGACAGTAGGAAGATTAGTCATTCATGTACGCACCCATTTCACCACGACCCTCCTGTGATAGGTCGGGAAAAATTCCATTCCCGACCGCAGCACGGTAAAGAGCTACCTTGCAGCACGTACGTGCAGAGCACCGCCGCAACTACCGAGGAGATAGAGGTACACATGCCCCCAGACACCCCTGATCGCACATTAATGTCACAACAGTCCGGCAACGTAAAGATCACGGTCAATGGCCAGACGGTGCGGTACAAGTGTAATTGCGGTGGCTCTAATGAAGGACTAACAACTACAGACAAAGTGATTAATAACTGCAAGGTTGATCAATGTCATGCCGCGGTCACCAATCACAAAAAGTGGCAGTATAACTCCCCTCTGGTCCCGCGTAATGCTGAACTTGGGGACCGAAAAGGAAAAATTCACATCCCGTTTCCGCTGGCAAATGTAACATGCAGGGTGCCTAAAGCAAGGAACCCCACCGTGACGTACGGGAAAAACCAAGTCATCATGCTACTGTATCCTGACCACCCAACACTCCTGTCCTACCGGAATATGGGAGAAGAACCAAACTATCAAGAAGAGTGGGTGATGCATAAGAAGGAAGTCGTGCTAGCCGTGCCGACTGAAGGGCTCGAGGTCACGTGGGGCAACAACGAGCCGTATAAGTATTGGCCGCAGTTATCTACAAACGGTACAGCCCATGGCCACCCGCATGAGATAATTCTGTATTATTATGAGCTGTACCCTACTATGACTGTAGTAGTTGTGTCAGTGGCCACGTTCATACTCCTGTCGATGGTGGGTATGGCAGCGGGGATGTGCATGTGTGCACGACGCAGATGCATCACACCGTATGAACTGACACCAGGAGCTACCGTCCCTTTCCTGCTTAGCCTAATATGCTGCATCAGAACAGCTAAAGCGGCCACATACCAAGAGGCTGCGATATACCTGTGGAACGAGCAGCAACCTTTGTTTTGGCTACAAGCCCTTATTCCGCTGGCAGCCCTGATTGTTCTATGCAACTGTCTGAGACTCTTACCATGCTGCTGTAAAACGTTGGCTTTTTTAGCCGTAATGAGCGTCGGTGCCCACACTGTGAGCGCGTACGAACACGCAACAGTGATCCCGAACACGGTGGGAGTACCGTATAAGACTCTAGTCAATAGACCTGGCTACAGCCCCATGGTATTGGAGATGGAACTACTGTCAGTCACTTTGGAGCCAACACTATCGCTTGATTACATCACGTGCGAGTACAAAACCGTCATCCCGTCTCCGTACGTGAAGTGCTGCGGTACAGCAGAGTGCAAGGACAAAAACCTACCTGACTACAGCTGTAAGGTCTTCACCGGCGTCTACCCATTTATGTGGGGCGGCGCCTACTGCTTCTGCGACGCTGAAAACACGCAGTTGAGCGAAGCACATGTGGAGAAGTCCGAATCATGCAAAACAGAATTTGCATCAGCATACAGGGCTCATACCGCATCTGCATCAGCTAAGCTCCGCGTCCTTTACCAAGGAAATAACATCACTGTAACTGCCTATGCAAACGGCGACCATGCCGTCACAGTTAAGGACGCCAAATTCATTGTGGGGCCAATGTCTTCAGCCTGGACACCTTTCGACAACAAAATTGTGGTGTACAAAGGTGACGTCTATAACATGGACTACCCGCCCTTTGGCGCAGGAAGACCAGGACAATTTGGCGATATCCAAAGTCGCACACCTGAGAGTAAAGACGTCTATGCTAATACACAACTGGTACTGCAGAGACCGGCTGTGGGTACGGTACACGTGCCATACTCTCAGGCACCATCTGGCTTTAAGTATTGGCTAAAAGAACGCGGGGCGTCACTGCAGCACACAGCACCATTTGGCTGCCAAATAGCAACAAACCCGGTAAGAGCGGTGAACTGCGCCGTAGGGAACATGCCCATCTCCATCGACATACCGGAAGCGGCCTTCACTAGGGTCGTCGACGCGCCCTCTTTAACGGACATGTCGTGCGAGGTACCAGCCTGCACCCATTCCTCAGACTTTGGGGGCGTCGCCATTATTAAATATGCAGCCAGCAAGAAAGGCAAGTGTGCGGTGCATTCGATGACTAACGCCGTCACTATTCGGGAAGCTGAGATAGAAGTTGAAGGGGATTCTCAGCTGCAAATCTCTTTCTCGACGGCCTTAGCCAGCGCCGAATTCCGCGTACAAGTCTGCTCAACACAAGTACACTGTGCAGCTGAGTGCCACCCCCCGAAGGACCACATAGTCAACTACCCGGCGTCACATACCACCCTCGGGGTCCAGGACATCTCCGCTACGGCGATGTCATGGGTGCAGAAGATCACGGGAGGTGTGGGACTGGTTGTTGCTGTTGCCGCACTGATTCTAATCGTGGTGCTATGCGTGTCGTTCAGCAGGCACTAACTTGACAATTAAGTATGAAGGTATATGTGTCCCCTAAGAGACACACCGTACATAGCAAATAATCTATAGATCAAAGGGCTACGCAACCCCTGAATAGTAACAAAATACAAAATCACTAAAAATTATAAAAACAGAAAAATACATAAATAGGTATACGTGTCCCCTAAGAGACACATTGTATGTAGGTGATAAGTATAGATCAAAGGGCCGAATAACCCCTGAATAGTAACAAAATATGAAAATCAATAAAAATCATAAAATAGAAAAACCATAAACAGAAGTAGTTCAAAGGGCTATAAAACCCCTGAATAGTAACAAAACATAAAATTAATAAAAATCAAATGAATACCATAATTGGCAAACGGAAGAGATGTAGGTACTTAAGCTTCCTAAAAGCAGCCGAACTCACTTTGAGAAGTAGGCATAGCATACCGAACTCTTCCACGATTCTCCGAACCCACAGGGACGTAGGAGATGTTATTTTGTTTTTAATATTTC

>GU199350|2008|China, Group 4

TCTACTCTGCAAAGCAAGAGATTAATAACCCATCATGGATCCTGTGTACGTGGACATAGACGCTGACAGCGCCTTTTTGAAGGCCCTGCAACGTGCGTACCCCATGTTTGAGGTGGAACCAAGGCAGGTCACACCGAATGACCATGCTAATGCTAGAGCGTTCTCGCATCTAGCTATAAAACTAATAGAGCAGGAAATTGACCCCGACTCAACCATCCTGGATATCGGCAGTGCGCCAGCAAGGAGGATGATGTCGGACAGGAAGTACCACTGCGTCTGCCCGATGCGCAGTGCGGAAGATCCCGAGAGACTCGCTAATTATGCGAGAAAGCTAGCATCTGCCGCAGGAAAAGTCCTGGACAGAAACATCTCTGGAAAGATCGGGGACTTACAAGCAGTAATGGCCGTGCCAGACAAGGAGACGCCAACATTCTGCTTACACACAGACGTCTCATGTAGACAGAGAGCAGACGTCGCTATATACCAAGACGTCTATGCTGTACACGCACCCACGTCGCTATACCACCAGGCGATTAAAGGGGTCCGAGTGGCGTACTGGGTTGGGTTCGACACAACCCCGTTCATGTACAATGCCATGGCGGGTGCCTACCCCTCATACTCGACAAACTGGGCAGATGAGCAGGTACTGAAGGCTAAGAACATAGGATTATGTTCAACAGACCTGACGGAAGGTAGACGAGGCAAGTTGTCTATTATGAGAGGGAAAAAGCTAAAACCGTGCGACCGTGTGCTGTTCTCAGTAGGGTCAACGCTCTACCCGGAAAGCCGCAAGCTACTTAAGAGCTGGCACCTGCCATCGGTGTTCCATTTAAAGGGCAAACTCAGCTTCACATGCCGCTGTGATACAGTGGTTTCGTGTGAGGGCTACGTCGTTAAGAGAATAACGATGAGTCCAGGCCTTTATGGAAAAACCACAGGGTATGCGGTAACCCACCACGCAGACGGATTCCTGATGTGCAAGACTACCGACACGGTTGACGGCGAAAGAGTGTCATTCTCGGTGTGCACATACGTGCCGGCGACCATTTGTGATCAAATGACCGGCATCCTTGCTACAGAAGTCACGCCGGAGGATGCACAGAAGCTGTTGGTGGGGCTGAACCAGAGAATAGTGGTTAACGGCAGAACGCAACGGAATATGAACACCATGAAAAATTATCTGCTTCCCGTGGTCGCCCAAGCCTTCAGTAAGTGGGCAAAGGAGTGCCGGAAAGACATGGAAGATGAAAAACTCCTGGGGGTCAGAGAAAGAACACTGACCTGCTGCTGTCTATGGGCATTCAAGAAGCAGAAAACACACACGGTCTACAAGAGGCCGGATACCCAGTCAATTCAGAAGGTTCAGGCCGAGTTTGACAGCTTTGTGGTACCGAGTCTGTGGTCGTCCGGGTTGTCAATCCCTTTGAGGACTAGAATCAAATGGTTGTTAAGCAAGGTGCCAAAAACCGACCTGATCCCATACAGCGGAGACGCCCGAGAAGCCCGGGACGCAGAAAAAGAAGCAGAGGAAGAACGAGAAGCAGAACTGACTCGCGAAGCCCTACCACCTCTACAGGCAGCACAGGAAGATGTTCAGGTCGAAATCGACGTGGAACAGCTTGAGGACAGAGCGGGCGCAGGAATAATAGAGACTCCGAGAGGAGCTATCAAAGTTACTGCCCAACCAACAGACCACGTCGTGGGAGAGTACCTGGTACTCTCCCCGCAGACCGTACTACGTAGCCAGAAGCTCAGTCTGATTCACGCTTTGGCGGAGCAAGTGAAGACGTGCACGCACAACGGACGAGCAGGGAGGTATGCGGTCGAAGCGTACGACGGCCGAGTCCTAGTGCCCTCAGGCTATGCAATCTCGCCTGAAGACTTCCAGAGTCTAAGCGAAAGCGCAACGATGGTGTATAACGAAAGAGAGTTCGTAAACAGAAAGCTACACCATATTGCGATGCACGGACCAGCCCTGAACACCGACGAAGAGTCGTATGAGCTGGTGAGGGCAGAGAGGACAGAACACGAGTACGTCTACGACGTGGATCAGAGAAGATGCTGTAAGAAGGAAGAAGCCGCAGGACTGGTACTGGTGGGCGACTTGACTAATCCGCCTTACCACGAATTCGCATATGAAGGGCTAAAAATCCGCCCTGCCTGCCCATACAAAATTGCAGTCATAGGAGTCTTCGGAGTACCGGGATCTGGCAAGTCAGCTATTATCAAGAACCTAGTTACCAGGCAGGACCTGGTGACTAGTGGAAAGAAAGAAAACTGCCAAGAAATCACCACCGACGTGATGAGACAGAGAGGTCTAGAGATATCTGCACGTACGGTTGACTCGCTGCTCTTGAATGGATGCAACAGACCAGTCGACGTGTTGTACGTAGACGAGGCGTTTGCGTGCCACTCTGGAACGCTACTTGCTTTGATCGCCTTGGTGAGACCAAGGCAGAAAGTTGTACTTTGTGGTGACCCGAAGCAGTGCGGCTTCTTCAATATGATGCAGATGAAAGTCAACTATAATCACAACATCTGCACCCAAGTGTACCACAAAAGTATCTCCAGGCGGTGCACACTGCCTGTGACCGCCATTGTGTCATCGTTGCATTACGAAGGCAAAATGCGCACTACGAATGAGTACAACAAGCCGATTGTAGTGGACACTACAGGCTCAACAAAACCTGACCCTGGAGACCTCGTGTTAACGTGCTTCAGAGGGTGGGTTAAACAACTGCAAATTGACTATCGTGGATACGAGGTCATGACAGCAGCCGCATCCCAAGGGTTAACCAGAAAAGGAGTTTACGCAGTTAGACAAAAAGTTAATGAAAACCCGCTCTATGCATCAACGTCAGAGCACGTCAACGTACTCCTAACGCGTACGGAAGGTAAACTGGTATGGAAGACACTTTCCGGCGACCCGTGGATAAAGACGCTGCAGAACCCACCGAAAGGAAACTTCAAAGCAACTATTAAGGAGTGGGAGGTGGAGCATGCATCAATAATGGCGGGCATCTGCAGTCACCAAATGACCTTCGATACATTCCAAAATAAAGCCAACGTTTGTTGGGCTAAGAGCTTGGTCCCTATCCTCGAAACAGCGGGGATAAAACTAAATGATAGGCAGTGGTCTCAGATAATTCAAGCCTTCAAAGAAGACAAAGCATACTCACCTGAAGTAGCCCTGAATGAAATATGTACGCGCATGTATGGGGTGGATCTAGACAGCGGGCTATTTTCTAAACCGTTGGTGTCTGTGTATTACGCGGATAACCACTGGGATAATAGGCCTGGAGGGAAAATGTTCGGATTTAACCCCGAGGCAGCATCCATTCTAGAAAGAAAGTACCCATTCACAAAAGGGAAGTGGAACATCAACAAGCAGATCTGCGTGACTACCAGGAGGATAGAAGACTTTAACCCTACCACCAACATCATACCGGCCAACAGGAGACTACCACACTCATTAGTGGCCGAACACCGCCCAGTAAAAGGGGAAAGAATGGAATGGCTGGTTAACAAGATAAACGGCCACCACGTGCTCCTGGTCAGTGGCTATAACCTTGCACTGCCTACTAAGAGAGTCACTTGGGTAGCGCCGTTAGGTGTCCGCGGAGCGGACTACACATACAACCTAGAGTTGGGTCTGCCAGCAACGCTTGGTAGGTATGACCTTGTGGTCATAAACATCCACACACCTTTTCGCATACACCATTACCAACAGTGCGTCGACCACGCAATGAAACTGCAAATGCTCGGGGGTGACTCATTGAGACTGCTCAAACCGGGCGGCTCTCTATTGATCAGAGCATATGGTTACGCAGATAGAACCAGTGAACGAGTCATCTGCGTATTGGGACGCAAGTTTAGATCGTCTAGAGCGTTGAAACCACCATGTGTCACCAGCAACACTGAGATGTTTTTCCTATTCAGTAACTTTGACAATGGCAGAAGGAATTTCACAACTCATGTCATGAACAATCAACTGAATGCAGCCTTCGTAGGACAGGTCACCCGAGCAGGATGTGCACCGTCGTACCGGGTAAAACGCATGGACATCGCGAAGAACGATGAAGAGTGCGTAGTCAACGCCGCTAACCCTCGCGGGTTACCGGGTGACGGTGTTTGCAAGGCAGTATACAAAAAATGGCCGGAGTCCTTTAAGAACAGTGCAACACCAGTGGGAACCGCAAAAACAGTTATGTGCGGTACGTATCCAGTAATCCACGCTGTTGGACCAAACTTCTCTAATTATTCGGAGTCTGAAGGGGACCGGGAATTGGCAGCTGCCTATCGAGAAGTCGCAAAGGAAGTAACTAGGCTGGGAGTAAATAGTGTAGCTATACCTCTCCTCTCCACAGGTGTATACTCAGGAGGGAAAGACAGGCTGACCCAGTCACTGAACCACCTCTTTACAGCCATGGACTCGACGGATGCAGACGTGGTCATCTACTGCCGCGACAAAGAATGGGAGAAGAAAATATCTGAGGCCATACAGATGCGGACCCAAGTAGAGCTGCTGGATGAGCACATCTCCATAGACTGCGATATTGTTCGCGTGCACCCTGACAGCAGCTTGGCAGGCAGAAAAGGATACAGCACCACGGAAGGCGCACTGTACTCATATCTAGAAGGGACCCGTTTTCATCAGACGGCTGTGGATATGGCGGAGATACATACTATGTGGCCAAAGCAAACAGAGGCCAATGAGCAAGTCTGCCTATATGCCCTGGGGGAAAGTATTGAATCGATCAGGCAGAAATGCCCGGTGGATGATGCAGACGCATCATCTCCCCCCAAAACTGTCCCGTGCCTTTGCCGTTACGCTATGACTCCAGAACGCGTCACCCGGCTTCGCATGAACCACGTCACAAGCATAATTGTGTGTTCTTCGTTTCCCCTCCCAAAGTACAAAATAGAAGGAGTGCAAAAAGTCAAATGCTCTAAGGTAATGCTATTTGACCACAACGTGCCATCGCGCGTAAGTCCAAGGGAATATAGATCTTCCCAGGAGTCTGCACAGGAGGCGAGTACAATCACGTCACTGACGCATAGTCAATTCGACCTAAGCGTTGATGGCGAGATACTGCCCGTCCCGTCAGACCTGGATGCTGACGCCCCAGCCCTAGAACCAGCACTAGACGACGGGGCGACACACACGCTGCCATCCACAACCGGAAACCTTGCGGCCGTGTCTGACTGGGTAATAAGCACCGTACCTGTCGCGCCGCCCAGAAGAAGGCGAGGGAGAAACCTGACTGTGACATGTGACGAGAGAGAAGGGAATATAACACCCATGGCTAGCGTCCGATTCTTTAGGGCAGAGCTGTGTCCGGTCGTACAAGAAACAGCGGAGACGCGTGACACAGCAATGTCTCTTCAGGCACCACCGAGTACCGCCACGGAACCGAATCATCCGCCGATCTCCTTCGGAGCATCAAGCGAGACGTTCCCCATTACATTTGGGGACTTCAACGAAGGAGAAATCGAAAGCTTGTCTTCTGAGCTACTAACTTTCGGAGACTTCTTACCAGGAGAAGTGGATGACTTGACAGACAGCGACTGGTCCACGTGCTCAGACACGGACGACGAGTTATGACTAGACAGGGCAGGTGGGTATATATTCTCGTCGGACACCGGTCCAGGTCATTTACAACAGAAGTCAGTACGCCAGTCAGTGCTGCCGGTGAACACCCTGGAGGAAGTCCACGAGGAGAAGTGTTACCCACCTAAGCTGGATGAAGCAAAGGAGCAACTATTACTTAAGAAACTCCAGGAGAGTGCATCCATGGCCAACAGAAGCAGGTATCAGTCGCGCAAAGTAGAAAACATGAAAGCAGCAATCATCCAGAGACTAAAGAGAGGCTGTAGACTATACTTAATGTCAGAGACCCCAAAAGTCCCTACTTACCGGACTACATATCCGGCGCCTGTGTACTCGCCTCCGATCAACGTTCGATTGTCCAATCCCGAGTCCGCAGTGGCAGCATGCAATGAGTTCTTAGCTAGAAACTATCCAACTGTCTCATCATACCAAATTACCGACGAGTATGATGCATATCTAGACATGGTGGACGGGTCGGAGAGTTGCCTGGACCGAGCGACATTCAATCCGTCAAAACTCAGGAGCTACCCGAAACAGCACGCTTACCACGCGCCCTCCATCAGAAGCGCTGTACCGTCCCCATTCCAGAACACACTACAGAATGTACTGGCAGCAGCCACGAAAAGAAACTGCAACGTCACACAGATGAGGGAATTACCCACTTTGGACTCAGCAGTATTCAACGTGGAGTGTTTCAAAAAATTCGCATGCAACCAAGAATACTGGGAAGAATTTGCTGCCAGCCCTATTAGGATAACAACTGAGAATTTAGCAACCTATGTTACTAAACTAAAAGGGCCAAAAGCAGCAGCGCTATTCGCAAAAACCCATAATCTACTGCCACTACAGGAAGTACCAATGGATAGGTTCACAGTAGATATGAAAAGGGACGTGAAGGTGACTCCTGGTACAAAGCATACAGAGGAAAGACCTAAGGTGCAGGTTATACAGGCGGCTGAACCCTTGGCGACAGCATACCTATGTGGGATTCACAGAGAGCTGGTTAGGAGGCTGAACGCCGTCCTCCTACCCAATGTACATACACTATTTGACATGTCTGCCGAGGATTTCGATGCCATCATAGCCGCACACTTTAAGCCAGGAGACACTGTTTTGGAAACGGACATAGCCTCCTTTGATAAGAGCCAAGATGATTCACTTGCGCTTACTGCTTTGATGCTGTTAGAGGATTTAGGGGTGGATCACTCCCTGCTGGACTTGATAGAGGCTGCTTTCGGAGAGATTTCCAGCTGTCACCTACCGACAGGTACGCGCTTCAAGTTCGGCGCCATGATGAAATCAGGTATGTTCCTAACTCTGTTCGTCAACACACTGTTAAACATCACCATCGCCAGCCGAGTGCTGGAAGATCGTCTGACAAAATCCGCGTGCGCGGCCTTCATCGGCGACGACAACATAATACATGGAGTCGTCTCCGATGAATTGATGGCAGCCAGATGTGCCACTTGGATGAACATGGAAGTGAAGATCATAGATGCAGTTGTATCCTTGAAAGCCCCTTACTTTTGTGGAGGGTTTATACTGCACGATACTGTGACAGGAACAGCTTGCAGAGTGGCAGACCCGCTAAAAAGGCTTTTTAAACTGGGCAAACCGCTAGCGGCAGGTGACGAACAAGATGAAGATAGAAGACGAGCGCTGGCTGACGAAGTGATCAGATGGCAACGAACAGGGCTAATTGATGAGCTGGAGAAAGCGGTATACTCTAGGTACGAAGTGCAGGGTATATCAGTTGTGGTAATGTCCATGGCCACCTTTGCAAGCTCCAGATCCAATTTCGAGAAGCTCAGAGGACCCGTCATAACTTTGTACGGCGGTCCTAAATAGGTACGCACTACAGCTACCTATTTTGCAGAAGCCGACAGCAAGTATCTAAACACTAATCAGCTACAATGGAGTTCATCCCAACCCAAACTTTTTACAATAGGAGGTACCAGCCTCGACCCTGGACTCCGCGCTCTACTATCCAAATCATCAGGCCCAGACCGCGCCCTCAGAGGCAAGCTGGGCAACTTGCCCAGCTGATCTCAGCAGTTAATAAACTGACAATGCGCGCGGTACCCCAACAGAAGCCACGCAGGAATCGGAAGAATAAGAAGCAAAAGCAAAAACAACAGGCGCCACAAAACAACACAAATCAAAAGAAGCAGCCACCTAAAAAGAAACCGGCTCAAAAGAAAAAGAAGCCGGGCCGCAGAGAGAGGATGTGCATGAAAATCGAAAATGATTGTATTTTCGAAGTCAAGCACGAAGGTAAGGTAACAGGTTACGCGTGCCTGGTGGGGGACAAAGTAATGAAACCAGCACACGTAAAGGGGACCATCGATAACGCGGACCTGGCCAAACTGGCCTTTAAGCGGTCATCTAAGTATGACCTTGAATGCGCGCAGATACCCGTGCACATGAAGTCCGACGCTTCGAAGTTCACCCATGAGAAACCGGAGGGGTACTACAACTGGCACCACGGAGCAGTACAGTACTCAGGAGGCCGGTTCACCATCCCTACAGGTGCTGGCAAACCAGGGGACAGCGGCAGACCGATCTTCGACAACAAGGGACGCGTGGTGGCCATAGTCTTAGGAGGAGCTAATGAAGGAGCCCGTACAGCCCTCTCGGTGGTGACCTGGAATAAAGACATTGTCACTAAAATCACCCCCGAGGGGGCCGAAGAGTGGAGTCTTGCCATCCCAGTTATGTGCCTGTTGGCAAACACCACGTTCCCCTGCTCCCAGCCCCCTTGCACGCCCTGCTGCTACGAAAAGGAACCGGAGGAAACCCTACGCATGCTTGAGGACAACGTCATGAGACCTGGGTACTATCAGCTGCTACAAGCATCCTTAACATGTTCTCCCCACCGCCAGCGACGCAGCACCAAGGACAACTTCAATGTCTATAAAGCCACAAGACCATACTTAGCTCACTGTCCCGACTGTGGAGAAGGGCACTCGTGCCATAGTCCCGTAGCACTAGAACGCATCAGAAATGAAGCGACAGACGGGACGCTGAAAATCCAGGTCTCCTTGCAAATCGGAATAAAGACGGATGACAGCCACGATTGGACCAAGCTGCGTTATATGGACAACCACATGCCAGCAGACGCAGAGAGGGCGGGGCTATTTGTAAGAACATCAGCACCGTGTACGATTACTGGAACAATGGGACACTTCATCCTGGCCCGATGTCCAAAAGGGGAAACTCTGACGGTGGGATTCACTGACAGTAGGAAGATTAGTCATTCATGTACGCACCCATTTCACCACGACCCTCCTGTGATAGGTCGGGAAAAATTCCATTCCCGACCGCAGCACGGTAAAGAGCTACCTTGCAGCACGTACGTGCAGAGCACCGCCGCAACTACCGAGGAGATAGAGGTACACATGCCCCCAGACACCCCTGATCACACATTAATGTCACAACAGTCCGGCAACGTAAAGATCACAGTCAATGGCCAGACGGTGCGGTACAAGTGTAATTGCGGTGGCTCAAATGAAGGACTAACAACTACAGACAAAGTGATTAATAACTGCAAGGTTGATCAATGTCATGCCGCGGTCACCAATCACAAAAAGTGGCAGTATAACTCCCCTCTGGTCCCGCGTAATGCTGAACTTGGGGACCGAAAAGGAAAAATTCACATCCCGTTTCCGCTGGCAAATGTAACATGCAGGGTGCCTAAAGCAAGGAACCCCACCGTGACGTACGGGAAAAACCAAGTCATCATGCTACTGTATCCTGACCACCCAACACTCCTGTCCTACCGGAATATGGGAGAAGAACCAAACTATCAAGAAGAGTGGGTGATGCATAAGAAGGAAGTCGTGCTAACCGTGCCGACTGAAGGGCTCGAGGTCACGTGGGGCAACAACGAGCCGTATAAGTATTGGCCGCAGTTATCTACAAACGGTACAGCCCATGGCCACCCGCATGAGATAATTCTGTATTATTATGAGCTGTACCCTACTATGACTGTAGTAGTTGTGTCAGTGGCCACGTTCATACTCCTGTCGATGGTGGGTATGGCAGCGGGGATGTGCATGTGTGCACGACGCAGATGCATCACACCGTATGAACTGACACCAGGAGCTACCGTCCCTTTCCTGCTTAGCCTAATATGCTGCATCAGAACAGCTAAAGCGGCCACATACCAAGAGGCTGCGATATACCTGTGGAACGAGCAGCAACCTTTGTTTTGGCTACAAGCCCTTATTCCGCTGGCAGCCCTGATTATTCTATGCAACTGTCTGAGACTCTTACCATGCTGCTGTAAAACGTTGGCTTTTTTAGCCGTAATGAGCGTCGGTGCCCACACTGTGAGCGCGTACGAACACGTAACAGTGATCCCGAACACGGTGGGAGTACCGTATAAGACTCTAGTCAATAGACCTGGCTACAGCCCCATGGTATTGGAGATGGAACTACTGTCAGTCACTTTGGAGCCAACACTATCGCTTGATTACATCACGTGCGAGTACAAAACCGTCATCCCGTCTCCGTACGTGAAGTGCTGCGGTACAGCAGAGTGCAAGGACAAAAACCTACCTGACTACAGCTGTAAGGTCTTCACCGGCGTCTACCCATTTATGTGGGGCGGCGCCTACTGCTTCTGCGACGCTGAAAACACGCAGTTGAGCGAAGCACATGTGGAGAAGTCCGAATCATGCAAAACAGAATTTGCATCAGCATACAGGGCTCATACCGCATCTGCATCAGCTAAGCTCCGCGTCCTTTACCAAGGAAATAACATCACTGTAACTGCCTATGCAAACGGCGACCATGCCGTCACAGTTAAGGACGCCAAATTCATTGTGGGGCCAATGTCTTCAGCCTGGACACCTTTCGACAACAAAATTGTGGTGTACAAAGGTGACGTCTATAACATGGACTACCCGCCCTTTGGCGCAGGAAGACCAGGACAATTTGGCGATATCCAAAGTCGCACACCTGAGAGTAAAGACGTCTATGCTAATACACAACTGGTACTGCAGAGACCGGCTGCGGGTACGGTACACGTGCCATACTCTCAGGCACCATCTGGCTTTAAGTATTGGCTAAAAGAACGCGGGGCGTCACTGCAGCACACAGCACCATTTGGCTGCCAAATAGCAACAAACCCGGTAAGAGCGGTGAACTGCGCCGTAGGGAACATGCCCATCTCCATCGACATACCGGAAGCGGCCTTCACTAGGGTCGTCGACGCGCCCTCTTTAACGGACATGTCGTGCGAGGTACCAGCCTGCACCCATTCCTCAGACTTTGGGGGCGTCGCCATTATTAAATATGCAGCCAGCAAGAAAGGCAAGTGTGCGGTGCATTCGATGACTAACGCCGTCACTATTCGGGAAGCTGAGATAGAAGTTGAAGGGAATTCTCAGCTGCAAATCTCTTTCTCGACGGCCTTAGCCAGCGCCGAATTCCGCGTACAAGTCTGTTCTACACAAGTACACTGTGCAGCTGAGTGCCACCCCCCGAAGGACCACATAGTCAACTACCCGGCGTCACATACCACCCTCGGGGTCCAGGACATCTCCGCTACGGCGATGTCATGGGTGCAGAAGATCACGGGAGGTGTGGGACTGGTTGTTGCTGTTGCCGCACTGATTCTAATCGTGGTGCTATGCGTGTCGTTCAGCAGGCACTAACTTGACAATTAAGTATGAAGGTATATGTGTCCCCTAAGAGACACACTGTACATAGCAAATAATCTATAGATCAAAGGGCTACGCAACCCCTGAATAGTAACAAAATACAAAATCACTAAAAATTATAAAAACAGAAAAATACATAAATAGGTATACGTGTCCCCTAAGAGACACATTGTATGTAGGTGATAAGTATAGATCAAAGGGCCGAACAACCCCTGAATAGTAACAAAATATGAAAATCAATAAAAATCATAAAATAGAAAAACCATAAACAGAAGTAGTTTAAAGGGCTATAAAACCCCTGAATAGTAACAAAACATAAAGTTAATAAAAATCAAATGAATACCATAATTGGCAAACGGAAGAGATGTAGGTACTTAAGCTTCCTAAAAGCAGCCGAACTCACTTGAGAA

>GU199351|2008|China, Group 4

TTTGGCTGCGTGAGACACACGTAGCCTACCAGTTTCTTACTGCTCTACTCTGCAAAGCAAGAGATTAATAACCCATCATGGATCCTGTGTACGTGGACATAGACGCTGACAGCGCCTTTTTGAAGGCCCTGCAACGTGCGTACCCCATGTTTGAGGTGGAACCAAGGCAGGTCACACCGAATGACCATGCTAATGCTAGAGCGTTCTCGCATCTAGCTATAAAACTAATAGAGCAGGAAATTGACCCCGACTCAACCATCCTGGATATCGGCAGTGCGCCAGCAAGGAGGATGATGTCGGACAGGAAGTACCACTGCGTCTGCCCGATGCGCAGTGCGGAAGATCCCGAGAGACTCGCTAATTATGCGAGAAAGCTAGCATCTGCCGCAGGAAAAGTCCTGGACAGAAACATCTCTGGAAAGATCGGGGACTTACGAGCAGTAATGGCCGTGCCAGACAAGGAGACGCCAACATTCTGCTTACACACAGACGTCTCATGCAGACAGAGAGCAGACGTCGCTATATACCAAGACGTCTATGCTGTACACGCACCCACGTCGCTATACCACCAGGCGATTAAAGGGGTCCGAGTGGCGTACTGGGTTGGGTTCGACACAACCCCGTTCATGTACAATGCCATGGCGGGTGCCTACCCCTCATACTCGACAAACTGGGCAGATGAGCAGGTACTGAAGGCTAAGAACATAGGATTATGTTCAACAGACCTGACGGAAGGTAGACGAGGCAAGTTGTCTATTATGAGAGGGAAAAAGCTAAAACCGTGCGACCGTGTGCTGTTCTCAGTAGGGTCAACGCTCTACCCGGAAAGCCGCAAGCTACTTAAGAGCTGGCACCTGCCATCGGTGTTCCATTTAAAGGGCAAACTTAGCTTCACATGCCGCTGTGATACAGTGGTTTCGTGTGAGGGCTACGTCGTTAAGAGAATAACGATGAGCCCAGGCCTTTATGGAAAAACCACAGGGTATGCGGTAACCCACCACGCAGACGGATTTCTGATGTGCAAGACTACCGACACGGTTGACGGCGAAAGAGTGTCATTCTCGGTGTGCACATACGTGCCGGCGACCATTTGTGATCAAATGACCGGCATCCTTGCTACAGAAGTCACGCCGGAGGATGCACAGAAGCTGTTGGTGGGGCTGAACCAGAGAATAGTGGTTAACGGCAGAACGCAACGGAATATGAACACCATGAAAAATTATCTGCTTCCCGTGGTCGCCCAAGCCTTCAGTAAGTGGGCAAAGGAGTGCCGGAAAGACATGGAAGATGAAAAACTCCTGGGGGTCAGAGAAAGAACACTGACCTGCTGCTGTCTATGGGCATTCAAGAAGCAGAAAACACACACGGTCTACAAGAGGCCTGATACCCAGTCAATTCAGAAGGTTCAGGCCGAGTTTGACAGCTTTGTGGTACCGAGTCTGTGGTCGTCCGGGTTGTCAATCCCTTTGAGGACTAGAATCAAATGGTTGTTAAGTAAGGTGCCAAAAACCGACCTGATCCCATACAGCGGAGACGCCCGAGAAGCCCGGGACGCAGAAAAAGAAGCAGAGGAAGAACGAGAAGCAGAACTGACTCGCGAAGCCCTACCACCTCTACAGGCAGCACAGGAAGATGTTCAGGTCGAAATCGACGTGGAACAGCTTGAGGACAGAGCGGGCGCAGGAATAATAGAGACTCCGAGAGGAGCTATCAAAGTTACTGCCCAACCAACAGACCACGTCGTGGGAGAGTACCTGGTACTCTCCCCGCAGACCGTACTACGTAGCCAGAAGCTCAGTCTGATTCACGCTTTGGCGGAGCAAGTGAAGACGTGCACGCACAACGGACGAGCAGGGAGGTATGCGGTCGAAGCGTACGACGGCCGAGTCCTAGTGCCCTCAGGCTATGCAATCTCGCCTGAAGACTTCCAGAGTCTAAGCGAAAGCGCAACGATGGTGTATAACGAAAGAGAGTTCGTAAACAGAAAGCTACACCATATTGCGATGCACGGACCGGCCCTGAACACCGACGAAGAGTCGTATGAGCTGGTGAGGGCAGAGAGGACAGAACACGAGTACGTCTACGACGTGGATCAGAGAAGATGCTGTAAGAAGGAAGAAGCCGCAGGACTGGTACTGGTGGGCGACTTGACTAATCCGCCCTACCACGAATTCGCATATGAAGGGCTAAAAATCCGCCCTGCCTGCCCATACAAAATTGCAGTCATAGGAGTCTTCGGAGTACCGGGATCTGGCAAGTCAGCTATTATCAAGAACCTAGTTACCAGGCAGGACCTGGTGACTAGCGGAAAGAAAGAAAACTGCCAAGAAATCACCACCGACGTGATGAGACAGAGAGGTCTAGAGATATCTGCACGTACGGTTGACTCGCTGCTCTTGAATGGATGCAACAGACCAGTCGACGTGTTGTACGTAGACGAGGCGTTTGCGTGCCACTCTGGAACGCTACTTGCTTTGATCGCCTTGGTGAGACCAAGGCAGAAAGTTGTACTTTGTGGTGACCCGAAGCAGTGCGGCTTCTTCAATATGATGCAGATGAAAGTCAACTATAATCACAACATCTGCACCCAAGTGTACCACAAAAGCATCTCCAGGCGGTGTACACTGCCTGTGACCGCCATTGTGTCATCGTTGCATTACGAAGGCAAAATGCGCACTACGAATGAGTACAACAAGCCGATTGTAGTGGACACTACAGGCTCAACAAAACCTGACCCTGGAGACCTCGTGTTAACGTGCTTCAGAGGGTGGGTTAAACAACTGCAAATTGACTATCGTGGATACGAGGTCATGACAGCAGCCGCATCCCAAGGGTTAACCAGAAAAGGAGTTTACGCAGTTAGACAAAAAGTTAATGAAAACCCGCTCTATGCATCAACGTCAGAGCACGTCAACGTACTCCTAACGCGTACGGAAGGTAAACTGGTATGGAAGACACTTTCCGGCGACCCGTGGATAAAGACGCTGCAGAACCCACCGAAAGGAAACTTCAAAGCAACTATTAAGGAGTGGGAGGTGGAGCATGCATCAATAATGGCGGGCATCTGCAGTCACCAAATGACCTTCGATACATTCCAAAATAAAGCCAACGTTTGTTGGGCTAAGAGCTTGGTCCCTATCCTCGAAACAGCGGGGATAAAACTAAATGATAGGCAGTGGTCTCAGATAATTCAAGCCTTCAAAGAAGACAAAGCATACTCACCTGAAGTAGCCCTGAATGAAATATGTACGCGCATGTATGGGGTGGATCTAGACAGCGGGCTATTTTCTAAACCGTTGGTGTCTGTGTATTACGCGGATAACCACTGGGATAATAGGCCTGGAGGGAAAATGTTCGGATTTAACCCCGAGGCAGCATCCATTCTAGAAAGAAAGTATCCATTCACAAAAAGGAAGTGGAACATCAACAAGCAGATCTGCGTGACTACCAGGAGGATAGAAGACTTTAACCCTACCACCAACATCATACCGGCCAACAGGAGACTACCACACTCATTAGTGGCCGAACACCGCCCAGTAAAAGGGGAAAGAATGGAATGGCTGGTTAACAAGATAAGCGGCCACCACGTGCTCCTGGTCAGTGGCTATAACCTTGCACTGCCTACTAAGAGAGTCACTTGGGTAGCGCCGTTAGGTGTCCGCGGAGCGGACTACACATACAACCTAGAGTTGGGTCTGCCAGCAACGCTTGGTAGGTATGACCTTGTGGTCATAAACATCCACACACCTTTTCGCATACACCATTACCAACAGTGCGTCGACCACGCAATGAAACTGCAAATGCTCGGGGGTGACTCATTGAGACTGCTCAAACCGGGCGGCTCTCTATTGATCAGAGCATATGGTTACGCAGATAGAACCAGTGAACGAGTCATCTGCGTATTGGGACGCAAGTTTAGATCGTCTAGAGCGTTGAAACCACCATGTGTCACCAGCAACACTGAGATGTTTTTCCTATTCAGCAACTTTGACAATGGCAGAAGGAATTTCACAACTCATGTCATGAACAATCAACTGAATGCAGCCTTCGTAGGACAGGTCACCCGAGCAGGATGTGCACCGTCGTACCGGGTAAAACGCATGGACATCGCGAAGAACGATGAAGAGTGCGTAGTCAACGCCGCTAACCCTCGCGGGTTACCGGGTGACGGTGTTTGCAAGGCAGTATACAAAAAATGGCCGGAGTCCTTTAAGAACAGTGCAACACCAGTGGGAACCGCAAAAACAGTTATGTGCGGTACGTATCCAGTAATCCACGCTGTTGGACCAAACTTCTCTAATTATTCGGAGTCTGAAGGGGACCGGGAATTGGCAGCTGCCTATCGAGAAGTCGCAAAGGAAGTAACTAGGCTGGGAGTAAATAGTGTAGCTATACCTCTCCTCTCCACAGGTGTATACTCAGGAGGGAAAGACAGGCTGACCCAGTCACTGAACCACCTCTTTACAGCCATGGACTCGACGGATGCAGACGTGGTCATCTACTGCCGCGACAAAGAATGGGAGAAGAAAATATCTGAGGCCATACAGATGCGGACCCAAGTAGAGCTGCTGGATGAGCACATCTCCATAGACTGCGATATTGTTCGCGTGCACCCTGACAGCAGCTTGGCAGGCAGAAAAGGATACAGCACCACGGAAGGCGCACTGTACTCATATCTAGAAGGGACCCGTTTTCATCAGACGGCTGTGGATATGGCGGAGATACATACTATGTGGCCAAAGCAAACAGAGGCCAATGAGCAAGTCTGCCTATATGCCCTGGGGGAAAGTATTGAATCGATCAGGCAGAAATGCCCGGTGGATGATGCAGACGCATCATCTCCCCCCAAAACTGTCCCGTGCCTTTGCCGTTACGCTATGACTCCAGAACGCGTCACCCGGCTTCGCATGAACCACGTCACAAGCATAATTGTGTGTTCTTCGTTTCCCCTCCCAAAGTACAAAATAGAAGGAGTGCAAAAAGTCAAATGCTCTAAGGTAATGCTATTTGACCACAACGTGCCATCGCGCGTAAGTCCAAGGGAATATAGATCTTCCCAGGAGTCTGCACAGGAGGCGAGTACAATCACGTCACTGACGCATAGTCAATTCGACCTAAGCGTTGATGGCGAGATACTGCCCGTCCCGTCAGACCTGGATGCTGACGCCCCAGCCCTAGAACCAGCACTAAACGACGGGGCGACACACACGCTGCCATCCACAACCGGAAACCTTGCGGCCGTGTCTGACTGGGTAATGAGCACCGTACCTGTCGCGCCGCCCAGAAGAAGGCGAGGGAGAAACCTGACTGTGACATGTGACGAGAGAGAAGGGAATATAACACCCATGGCTAGCGTCCGATTCTTTAGGGCAGAGCTGTGTCCGGTCGTACAAGAAACAGCGGAGACGCGTGACACAGCAATGTCTCTTCAGGCACCACCGAGTACCGCCACGGAACCGAATCATCCGCCGATCTCCTTCGGAGCATCAAGCGAGACGTTCCCCATTACATTTGGGGACTTCAACGAAGGAGAAATCGAAAGCTTGTCTTCTGAGCTACTAACTTTCGGAGACTTCTTACCAGGAGAAGTGGATGACTTGACAGACAGCGACTGGTCCACGTGCTCAGACACGGACGACGAGTTATGACTAGACAGGGCAGGTGGGTATATATTCTCGTCGGACACCGGTCCAGGTCATTTACAACAGAAGTCAGTACGCCAGTCAGTGCTGCCGGTGAACACCCTGGAGGAAGTCCACGAGGAGAAGTGTTACCCACCTAAGCTGGATGAAGCAAAGGAGCAACTATTACTTAAGAAACTCCAGGAGAGTGCATCCATGGCCAACAGAAGCAGGTATCAGTCGCGCAAAGTAGAAAACATGAAAGCAGCAATCATCCAGAGACTAAAGAGAGGCTGTAGACTATACTTAATGTCAGAGACCCCAAAAGTCCCTACTTACCGGACTACATATCCGGCGCCTGTGTACTCGCCTCCGATCAACGTCCGATTGTCCAATCCCGAGTCCGCAGTGGCAGCATGCAATGAGTTCTTAGCTAGAAACTATCCAACTGTCTCATCATACCAAATTACCGACGAGTATGATGCATATCTAGACATGGTGGACGGGTCGGAGAGTTGCCTGGACCGAGCGACATTCAATCCGTCAAAACTCAGGAGCTACCCGAAACAGCACGCTTACCACGCGCCCTCCATCAGAAGCGCTGTACCGTCCCCATTCCAGAACACACTACAGAATGTACTGGCAGCAGCCACGAAAAGAAACTGCAACGTCACACAGATGAGGGAATTACCCACTTTGGACTCAGCAGTATTCAACGTGGAGTGTTTCAAAAAATTCGCATGCAACCAAGAATACTGGGAAGAATTTGCTGCCAGCCCTATTAGGATAACAACTGAGAATTTAGCAACCTATGTTACTAAACTAAAAGGGCCAAAAGCAGCAGCGCTATTCGCAAAAACCCATAATCTACTGCCACTACAGGAAGTACCAATGGATAGGTTCACAGTAGATATGAAAAGGGACGTGAAGGTGACTCCTGGTACAAAGCATACAGAGGAAAGACCTAAGGTGCAGGTTATACAGGCGGCTGAACCCTTGGCGACAGCATACCTATGTGGGATTCACAGAGAGCTGGTTAGGAGGCTGAACGCCGTCCTCCTACCCAATGTTCATACACTATTTGACATGTCTGCCGAGGATTTCGATGCCATCATAGCCGCACACTTTAAGCCAGGAGACACTGTTTTGGAAACGGACATAGCCTCCTTTGATAAGAGCCAAGATGATTCACTTGCGCTTACTGCTTTGATGCTGTTAGAGGATTTAGGGGTGGATCACTCCCTGCTGGACTTGATAGAGGCTGCTTTCGGAGAGATTTCCAGCTGTCACCTACCGACAGGTACGCGCTTCAAGTTCGGCGCCATGATGAAATCAGGTATGTTCCTAACTCTGTTCGTCAACACATTGTTAAACATCACCATCGCCAGCCGAGTGCTGGAAGATCGTCTGACAAAATCCGCGTGCGCGGCCTTCATCGGCGACGACAACATAATACATGGAGTCGTCTCCGATGAATTGATGGCAGCCAGATGTGCCACTTGGATGAACATGGAAGTGAAGATCATAGATGCAGTTGTATCCTTGAAAGCCCCTTACTTTTGTGGAGGGTTTATACTGCACGATACTGTGACAGGAACAGCTTGCAGAGTGGCAGACCCGCTAAAAAGGCTTTTTAAACTGGGCAAACCGCTAGCGGCAGGTGACGAACAAGATGAAGATAGAAGACGAGCGCTGGCTGACGAAGTGATCAGATGGCAACGAACAGGGCTAATTGATGAGCTGGAGAAAGCGGTATACTCTAGGTACGAAGTGCAGGGTATATCAGTTGTGGTAATGTCCATGGCCACCTTTGCAAGCTCCAGATCCAACTTCGAGAAGCTCAGAGGACCCGTCATAACTTTGTACGGCGGTCCTAAATAGGTACGCACTACAGCTACCTATTTTGCAGAAGCCGACAGCAAGTATCTAAACACTAATCAGCTACAATGGAGTTCATCCCAACCCAAACTTTTTACAATAGGAGGTACCAGCCTCGACCCTGGACTCCGCGCTCTACTATCCAAGTCATCAGGCCCAGACCGCGCCCTCAGAGGCAAGCTGGGCAACTTGCCCAGCTGATCTCAGCAGTTAATAAACTGACAATGCGCGCGGTACCCCAACAGAAGCCACGCAGGAATCGGAAGAATAAGAAGCAAAAGCAAAAACAACAGGCGCCACAAAACAACACAAATCAAAAGAAGCAGCCACCTAAAAAGAAACCGGCTCAAAAGAAAAAGAAGCCGGGCCGCAGAGAGAGGATGTGCATGAAAATCGAAAATGATTGTATTTTCGAAGTCAAGCACGAAGGTAAGGTAACAGGTTACGCGTGCCTGGTGGGGGACAAAGTAATGAAACCAGCACACGTAAAGGGGACCATCGATAACGCGGACCTGGCCAAACTGGCCTTTAAGCGGTCATCTAAGTATGACCTTGAATGCGCGCAGATACCCGTGCACATGAAGTCCGACGCTTCGAAGTTCACCCATGAGAAACCGGAGGGGTACTACAACTGGCACCACGGAGCAGTACAGTACTCAGGAGGCCGGTTCACCATCCCTACAGGTGCTGGCAAACCAGGGGACAGCGGCAGACCGATCTTCGACAACAAGGGACGCGTGGTGGCCATAGTCTTAGGAGGAGCTAATGAAGGAGCCCGTACAGCCCTCTCGGTGGTGACCTGGAATAAAGACATTGTCACTAAAATCACCCCCGAAGGGGCCGAAGAGTGGAGTCTTGCCATCCCAGTTATGTGCCTGTTGGCAAACACCACGTTCCCCTGCTCCCAGCCCCCTTGCACGCCCTGCTGCTACGAAAAGGAACCGGAGGAAACCCTACGCATGCTTGAGGACAACGTCATGAGACCTGGGTACTATCAGCTGCTACAAGCATCCTTAACATGCTCTCCCCACCGCCAGCGACGCAGCACCAAGGACAACTTCAATGTCTATAAAGCCACAAGACCATACTTAGCTCACTGTCCCGACTGTGGAGAAGGGCACTCGTGCCATAGTCCCGTAGCACTAGAACGCATCAGAAATGAAGCGACAGACGGGACGCTGAAAATCCAGGTCTCCTTGCAAATCGGAATAAAGACGGATGACAGCCACGATTGGACCAAGCTGCGTTATATGGACAACCACATGCCAGCAGACGCAGAGAGGGCGGGGCTATTTGTAAGAACATCAGCACCGTGTACGATTACTGGAACAATGGGACACTTCATCCTGGCCCGATGTCCAAAAGGGGAAACTCTGACGGTGGGATTCACTGACAGTAGGAAGATTAGTCATTCATGTACGCACCCATTTCACCACGACCCTCCAGTGATAGGTCGGGAAAAATTCCATTCCCGACCGCAGCACGGTAAAGAGCTACCTTGCAGCACGTACGTGCAGAGCACCGCCGCAACTACCGAGGAGATAGAGGTACACATGCCCCCAGACACCCCTGATCGCACATTAATGTCACAACAGTCCGGCAACGTAAAGATCACAGTCAATGGCCAGACGGTGCGGTACAAGTGTAATTGCGGTGGCTCAAATGAAGGACTAACAACTACAGACAAAGTGATTAATAACTGCAAGGTTGATCAATGTCATGCCGCGGTCACCAATCACAAAAAATGGCAGTATAACTCCCCTCTGGTCCCGCGTAATGCTGAACTTGGGGACCGAAAAGGAAAAATTCACATCCCGTTTCCGCTGGCAAATGTAACATGCAGGGTGCCTAAAGCAAGGAACCCCACCGTGACGTACGGGAAAAACCAAGTCATCATGCTACTGTATCCTGACCACCCAACACTCCTGTCCTACCGGAATATGGGAGAAGAACCAAACTATCAAGAAGAGTGGGTGATGCATAAGAAGGAAGTCGTGCTAACCGTGCCGACTGAAGGGCTCGAGGTCACGTGGGGCAACAACGAGCCGTATAAGTATTGGCCGCAGTTATCTACAAATGGTACAGCCCATGGCCACCCGCATGAGATAATTCTGTATTATTATGAGCTGTACCCTACTATGACTGTAGTAGTTGTGTCAGTGGCCACGTTCATACTCCTGTCGATGGTGGGTATGGCAGCGGGGATGTGCATGTGTGCACGACGCAGATGCATCACACCGTATGAACTGACACCAGGAGCTACCGTCCCTTTCCTGCTTAGCCTAATATGCTGCATCAGAACAGCTAAAGCGGCCACATACCAAGAGGCTGCGATATACCTGTGGAACGAGCAGCAACCTTTGTTTTGGCTACAAGCCCTTATTCCGCTGGCAGCCCTGATTGTTCTATGCAACTGTCTGAGACTCTTACCATGCTGCTGTAAAACGTTGGCTTTTTTAGCCGTAATGAGCGTCGGTGCCCACACTGTGAGCGCGTACGAACACGTAACAGTGATCCCGAACACGGTGGGAGTACCGTATAAGACTCTAGTCAATAGACCTGGCTACAGCCCCATGGTATTGGAGATGGAACTACTGTCGGTCACTTTGGAGCCAACACTATCGCTTGATTACATCACGTGCGAGTACAAAACCGTCATCCCGTCTCCGTACGTGAAGTGCTGCGGTACAGCAGAGTGCAAGGACAAAAACCTACCTGACTACAGCTGTAAGGTCTTCACCGGCGTCTACCCATTTATGTGGGGCGGCGCCTACTGCTTCTGCGACGCTGAAAACACGCAGTTGAGCGAAGCACATGTGGAGAAGTCCGAATCATGCAAAACAGAATTTGCATCAGCATACAGGGCTCATACCGCATCTGCATCAGCTAAGCTCCGCGTCCTTTACCAAGGAAATAACATCACTGTAACTGCCTATGCAAACGGCGACCATGCCGTCACAGTTAAGGACGCCAAATTCATTGTGGGGCCAATGTCTTCAGCCTGGACACCTTTCGACAACAAAATTGTGGTGTACAAAGGTGACGTCTATAACATGGACTACCCGCCCTTTGGCGCAGGAAGACCAGGACAATTTGGCGATATCCAAAGTCGCACACCTGAGAGTAAAGACGTCTATGCTAATACACAACTGGTACTGCAGAGACCGGCTGCGGGTACGGTACACGTGCCATACTCTCAGGCACCATCTGGCTTTAAGTATTGGCTAAAAGAACGCGGGGCGTCACTGCAGCACACAGCACCATTTGGCTGCCAAATAGCAACAAACCCGGTAAGAGCGATGAACTGCGCCGTAGGGAACATGCCCATCTCCATCGACATACCGGAAGCGGCCTTCACTAGGGTCGTCGACGCGCCCTCTTTAACGGACATGTCGTGCGAGGTACCAGCCTGCACCCATTCCTCAGACTTTGGGGGCGTCGCCATTATTAAATATGCAGCCAGCAAGAAAGGCAAGTGTGCGGTGCATTCGATGACTAACGCCGTCACTATTCGGGAAGCTGAGATAGAAGTTGAAGGGAATTCTCAGCTGCAAATCTCTTTCTCGACGGCCTTAGCCAGCGCCGAATTCCGCGTACAAGTCTGTTCTACACAAGTACACTGTGCAGCTGAGTGCCACCCCCCGAAGGACCACATAGTCAACTACCCGGCGTCACATACCACCCTCGGGGTCCAGGACATCTCCGCTACGGCGATGTCATGGGTGCAGAAGATCACGGGAGGTGTGGGACTGGTTGTTGCTGTTGCCGCACTGATTCTAATCGTGGTGCTATGCGTGTCGTTTAGCAGGCACTAACTTGACAATTAAGTATGAAGGTATATGTGTCCCCTAAGAGACACACTGTACATAGCAAATAATCTATAGATCAAAGGGCTACGCAACCCCTGAATAGTAACAAAATACAAAATCACTAAAAATTATAAAAACAGAAAAATACATAAATAGGTATACGTGTCCCCTAAGAGACACATTGTATGTAGGTGATAAGTATAGATCAAAGGGCCGAATAACCCCTGAATAGTAACAAAATATGAAAATCAATAAAAATCATAAAATAGAAAAACCATAAACAGAAGTAGTTCAAAGGGCTATAAAACCCCTGAATAGTAACAAAACATAAAATTAATAAAAATCAAATGAATACCATAATTGGCAAACGGAAGAGATGTAGGTACTTAAGCTTCCTAAAAGCAGCCGAACTCACTTTGAGAAGTAGGCATAGCATACCGAACTCTTCCATGATTCTCCGAACCCACAGGGACGTGGAGATGTTA

>GU199352|2008|China, Group 4

TCTACTCTGCAAAGCAAGAGATTAATAACCCATCATGGATCCTGTGTACGTGGACATAGACGCTGACAGCGCCTTTTTGAAGGCCCTGCAACGTGCGTACCCCATGTTTGAGGTGGAACCAAGGCAGGTCACACCGAATGACCATGCTAATGCTAGAGCGTTCTCGCATCTAGCTATAAAACTAATAGAGCAGGAAATTGACCCCGACTCAACCATCCTGGATATCGGCAGTGCGCCAGCAAGGAGGATGATGTCGGACAGGAAGTACCACTGCGTCTGCCCGATGCGCAGTGCGGAAGATCCCGAGAGACTCGCTAATTATGCGAGAAAGCTAGCATCTGCCGCAGGAAAAGTCCTGGACAGAAACATCTCTGGAAAGATCGGGGACTTACAAGCAGTAATGGCCGTGCCAGACAAGGAGACGCCAACATTCTGCTTACACACAGACGTCTCATGTAGACAGAGAGCAGACGTCGCTATATACCAAGACGTCTATGCTGTACACGCACCCACGTCGCTATACCACCAGGCGATTAAAGGGGTCCGAGTGGCGTACTGGGTTGGGTTCGACACAACCCCGTTCATGTACAATGCCATGGCGGGTGCCTACCCCTCATACTCGACAAACTGGGCAGATGAGCAGGTACTGAAGGCTAAGAACATAGGATTATGTTCAACAGACCTGACGGAAGGTAGACGAGGCAAGTTGTCTATTATGAGAGGGAAAAAGCTAAAACCGTGCGACCGTGTGCTGTTCTCAGTAGGGTCAACGCTCTACCCGGAAAGCCGCAAGCTACTTAAGAGCTGGCACCTGCCATCGGTGTTCCATTTAAAGGGCAAACTCAGCTTCACATGCCGCTGTGATACAGTGGTTTCGTGTGAGGGCTACGTCGTTAAGAGAATAACGATGAGCCCAGGCCTTTATGGAAAAACCACAGGGTATGCGGTAACCCACCACGCAGACGGATTCCTGATGTGCAAGACTACCGACACGGTTGACGGCGAAAGAGTGTCATTCTCGGTGTGCACATACGTGCCGGCGACCATTTGTGATCAAATGACCGGCATCCTTGCTACAGAAGTCACGCCGGAGGATGCACAGAAGCTGTTGGTGGGGCTGAACCAGAGAATAGTGGTTAACGGCAGAACGCAACGGAATATGAACACCATGAAAAATTATCTGCTTCCCGTGGTCGCCCAAGCCTTCAGTAAGTGGGCAAAGGAGTGCCGGAAAGACATGGAAGATGAAAAACTCCTGGGGGTCAGAGAAAGAACACTGACCTGCTGCTGTCTATGGGCATTCAAGAAGCAGAAAACACACACGGTCTACAAGAGGCCGGATACCCAGTCAATTCAGAAGGTTCAGGCCGAGTTTGACAGCTTTGTGGTACCGAGTCTGTGGTCGTCCGGGTTGTCAATCCCTTTGAGGACTAGAATCAAATGGTTGTTAAGCAAGGTGCCAAAAACCGACCTGATCCCGTATAGCGGAGACGCCCGAGAAGCCCGGGACGCAGAAAAAGAAGCAGAGGAAGAACGAGAAGCAGAACTGACTCGCGAAGCCCTACCACCTCTACAGGCAGCACAGGAAGATGTTCAGGTCGAAATCGACGTGGAACAGCTTGAGGACAGAGCGGGCGCAGGAATAATAGAGACTCCGAGAGGAGCTATCAAAGTTACTGCCCAACCAACAGACCACGTCGTGGGAGAGTACCTGGTACTCTCCCCGCAGACCGTACTACGTAGCCAGAAGCTCAGTCTGATTCACGCTTTGGCGGAGCAAGTGAAGACGTGCACGCACAACGGACGAGCAGGGAGGTATGCGGTCGAAGCGTACGACGGCCGAGTCCTAGTGCCCTCAGGCTATGCAATCTCGCCTGAAGACTTCCAGAGTCTAAGCGAAAGCGCAACGATGGTGTATAACGAAAGAGAGTTCGTAAACAGAAAGCTACACCATATTGCGATGCACGGACCAGCCCTGAACACCGACGAAGAGTCGTATGAGCTGGTGAGGGCAGAGAGGACAGAACACGAGTACGTCTACGACGTGGATCAGAGAAGATGCTGTAAGAAGGAAGAAGCCGCAGGACTGGTACTGGTGGGCGACTTGACTAATCCGCCCTACCACGAATTCGCATATGAAGGGCTAAAAATCCGCCCTGCCTGCCCATACAAAATTGCAGTCATAGGAGTCTTCGGAGTACCGGGATCTGGCAAGTCAGCTATTATCAAGAACCTAGTTACCAGGCAGGACCTGGTGACTAGCGGAAAGAAAGAAAACTGCCAAGAAATCACCACCGACGTGATGAGACAGAGAGGTCTAGAGATATCTGCACGTACGGTTGACTCGCTGCTCTTGAATGGATGCAACAGACCAGTCGACGTGTTGTACGTAGACGAGGCGTTTGCGTGCCACTCTGGAACGCTACTTGCTTTGATCGCCTTGGTGAGACCAAGGCAGAAAGTTGTACTTTGTGGTGACCCGAAGCAGTGCGGCTTCTTCAATATGATGCAGATGAAAGTCAACTATAATCACAACATCTGCACCCAAGTGTACCACAAAAGTATCTCCAGGCGGTGTACACTGCCTGTGACCGCCATTGTGTCATCGTTGCATTACGAAGGCAAAATGCGCACTACGAATGAGTACAACAAGCCGATTGTAGTGGACACTACAGGCTCAACAAAACCTGACCCTGGAGACCTCGTGTTAACGTGCTTCAGAGGGTGGGTTAAACAACTGCAAATTGACTATCGTGGATACGAGGTCATGACAGCAGCCGCATCCCAAGGGTTAACCAGAAAAGGAGTTTACGCAGTTAGACAAAAAGTTAATGAAAACCCGCTCTATGCATCAACGTCAGAGCACGTCAACGTACTCCTAACGCGTACGGAAGGTAAACTGGTATGGAAGACACTTTCCGGCGACCCGTGGATAAAGACGCTGCAGAACCCACCGAAAGGAAACTTCAAAGCAACTATTAAGGAGTGGGAGGTGGAGCATGCATCAATAATGGCGGGCATCTGCAGTCACCAAATGACCTTCGATACATTCCAAAATAAAGCCAACGTTTGTTGGGCTAAGAGCTTGGTCCCTATCCTCGAAACAGCGGGGATAAAACTAAATGATAGGCAGTGGTCTCAGATAATTCAAGCCTTCAAAGAAGACAAAGCATACTCACCTGAAGTAGCCCTGAATGAAATATGTACGCGCATGTATGGGGTGGATCTAGACAGCGGGCTATTTTCTAAACCGTCGGTGTCTGTGTATTACGCGGATAACCACTGGGATAATAGGCCTGGAGGGAAAATGTTCGGATTTAACCCCGAGGCAGCATCCATTCTAGAAAGAAAGTACCCATTCACAAAAGGGAAGTGGAACATCAACAAGCAGATCTGCGTGACTACCAGGAGGATAGAAGACTTTAACCCTACCACCAACATCATACCGGCCAACAGGAGACTACCACACTCATTAGTGGCCGAACACCGCCCAGTAAAAGGGGAAAGAATGGAATGGCTGGTTAACAAGATAAACGGCCACCACGTGCTCCTGGTCAGTGGCTATAACCTTGCACTGCCTACTAAGAGAGTCACTTGGGTAGCGCCGTTAGGTGTCCGCGGAGCGGACTACACATACAACCTAGAGTTGGGTCTGCCAGCAACGCTTGGTAGGTATGACCTTGTGGTCATAAACATCCACACACCTTTTCGCATACACCATTACCAACAGTGCGTCGACCACGCAATGAAACTGCAAATGCTCGGGGGTGACTCATTGAGACTGCTCAAACCGGGCGGCTCTCTATTGATCAGAGCATATGGTTACGCAGATAGAACCAGTGAACGAGTCATCTGCGTATTGGGACGCAAGTTTAGATCGTCTAGAGCGTTGAAACCACCATGTGTCACCAGCAACACTGAGATGTTTTTCCTATTCAGCAACTTTGACAATGGCAGAAGGAATTTCACAACTCATGTCATGAACAATCAACTGAATGCAGCCTTCGTAGGACAGGTCACCCGAGCAGGATGTGCACCGTCGTACCGGGTAAAACGCATGGACATCGCGAAGAACGATGAAGAGTGCGTAGTCAACGCCGCTAACCCTCGCGGGTTACCGGGTGACGGTGTTTGCAAGGCAGTATACAAAAAATGGCCGGAGTCCTTTAAGAACAGTGCAACACCAGTGGGAACCGCAAAAACAGTTATGTGTGGTACGTATCCAGTAATCCACGCTGTTGGACCAAACTTCTCTAATTATTCGGAGTCTGAAGGGGACCGGGAATTGGCAGCTGCCTATCGAGAAGTCGCAAAGGAAGTAACTAGGCTGGGAGTAAATAGTGTAGCTATACCTCTCCTCTCCACAGGTGTATACTCAGGAGGGAAAGACAGGCTGACCCAGTCACTGAACCACCTCTTTACAGCCATGGACTCGACGGATGCAGACGTGGTCATCTACTGCCGCGACAAAGAATGGGAGAAGAAAATATCTGAGGCCATACAGATGCGGACCCAAGTAGAGCTGCTGGATGAGCACATCTCCATAGACTGCGATATTGTTCGCGTGCACCCTGACAGCAGCTTGGCAGGCAGAAAAGGATACAGCACCACGGAAGGCGCACTGTACTCATATCTAGAAGGGACCCGTTTTCATCAGACGGCTGTGGATATGGCGGAGATACATACTATGTGGCCAAAGCAAACAGAGGCCAATGAGCAAGTCTGCCTATATGCCCTGGGGGAAAGTATTGAATCGATCAGGCAGAAATGCCCGGTGGATGATGCAGACGCATCATCTCCCCCCAAAACTGTCCCGTGCCTTTGCCGTTACGCTATGACTCCAGAACGCGTCACCCGGCTTCGCATGAACCACGTCACAAGCATAATTGTGTGTTCTTCGTTTCCCCTCCCAAAGTACAAAATAGAAGGAGTGCAAAAAGTCAAATGCTCTAAGGTAATGCTATTTGACCATAACGTGCCATCGCGCGTAAGTCCAAGGGAATATAGATCTTCCCAGGAGTCTGCACAGGAGGCGAGTACAATCACGTCACTGACGCATAGTCAATTCGACCTAAGCGTTGATGGCGAGATACTGCCCGTCCCGTCAGACCTGGATGCTGACGCCCCAGCCCTAGAACCAGCACTAGACGACGGGGCGACACACACGCTGCCATCCACAACCGGAAACCTTGCGGCCGTGTCTGACTGGGTAATGAGCACCGTACCTGTCGCGCCGCCCAGAAGAAGGCGAGGGAGAAACCTGACTGTGACATGTGACGAGAGAGAAGGGAATATAACACCCATGGCTAGCGTCCGATTCTTTAGGGCAGAGCTGTGTCCGGTCGTACAAGAAACAGCGGAGACGCGTGACACAGCAATGTCTCTTCAGGCACCACCGAGTACCGCCACGGAACCGAATCATCCGCCGATCTCCTTCGGAGCATCAAGCGAGACGTTCCCCATTACATTTGGGGACTTCAACGAAGGAGAAATCGAAAGCTTGTCTTCTGAGCTACTAACTTTCGGAGACTTCTTACCAGGAGAAGTGGATGACTTGACAGACAGCGACTGGTCCACGTGCTCAGACACGGACGACGAGTTATGACTAGACAGGGCAGGTGGGTATATATTCTCGTCGGACACCGGTCCAGGTCATTTACAACAGAAGTCAGTACGCCAGTCAGTGCTGCCGGTGAACACCCTGGAGGAAGTCCACGAGGAGAAGTGTTACCCACCTAAGCTGGATGAAGCAAAGGAGCAACTATTACTTAAGAAACTCCAGGAGAGTGCATCCATGGCCAACAGAAGCAGGTATCAGTCGCGCAAAGTAGAAAACATGAAAGCAGCAATCATCCAGAGACTAAAGAGAGGCTGTAGACTATACTTAATGTCAGAGACCCCAAAAGTCCCTACTTACCGGACTACATATCCGGCGCCTGTGTACTCGCCTCCGATCAACGTCCGATTGTCCAATCCCGAGTCCGCAGTGGCAGCATGCAATGAGTTCTTAGCTAGAAACTATCCAACTGTCTCGTCATACCAAATTACCGACGAGTATGATGCATATCTAGACATGGTGGACGGGTCGGAGAGTTGCCTGGACCGAGCGACATTCAATCCGTCAAAACTCAGGAGCTACCCGAAACAGCACGCTTACCACGCGTCCTCCATCAGAAGCGCTGTACCGTCCCCATTCCAGAACACACTACAGAATGTACTGGCAGCAGCCACGAAAAGAAACTGCAACGTCACACAGATGAGGGAATTACCCACTTTGGACTCAGCAGTATTCAACGTGGAGTGTTTCAAAAAATTCGCATGCAACCAAGAATACTGGGAAGAATTTGCTGCCAGCCCTATTAGGATAACAACTGAGAATTTAGCAACCTATGTTACTAAACTAAAAGGGCCAAAAGCAGCAGCGCTATTCGCAAAAACCCATAATCTACTGCCACTACAGGAAGTACCAATGGATAGGTTCACAGTAGATATGAAAAGGGACGTGAAGGTGACTCCTGGTACAAAGCATACAGAGGAAAGACCTAAGGTGCAGGTTATACAGGCGGCTGAACCCTTGGCGACAGCATACCTATGTGGGATTCACAGAGAGCTGGTTAGGAGGCTGAACGCCGTCCTCCTACCCAATGTACATACACTATTTGACATGTCTGCCGAGGATTTCGATGCCATCATAGCCGCACACTTTAAGCCAGGAGACACTGTTTTGGAAACGGACATAGCCTCCTTTGATAAGAGCCAAGACGATTCGCTTGCGCTTACTGCTTTGATGCTGTTAGAGGATTTAGGGGTGGATCACTCCCTGCTGGACTTGATAGAGGCTGCTTTCGGAGAGATTTCCAGCTGTCACCTACCGACAGGTACGCGCTTCAAGTTCGGCGCCATGATGAAATCAGGTATGTTCCTAACTCTGTTCGTCAACACATTGTTAAACATCACCATCGCCAGCCGAGTGCTGGAAGATCGTCTGACAAAATCCGCGTGCGCGGCCTTCATCGGCGACGACAACATAATACATGGAGTCGTCTCCGATGAATTGATGGCAGCCAGATGTGCCACTTGGATGAACATGGAAGTGAAGATCATAGATGCAGTTGTATCCTTGAAAGCCCCTTACTTTTGTGGAGGGTTTATACTGCACGATACTGTGACAGGAACAGCTTGCAGAGTGGCAGACCCGCTAAAAAGGCTTTTTAAACTGGGCAAACCGCTAGCGGCAGGTGACGAACAAGATGAAGATAGAAGACGAGCGCTGGCTGACGAAGTGATCAGATGGCAACGAACAGGGCTAATTGATGAGCTGGAGAAAGCGGTATACTCTAGGTACGAAGTGCAGGGTATATCAGTTGTGGTAATGTCCATGGCCACCTTTGCAAGCTCCAGATCCAATTTCGAGAAGCTCAGAGGACCCGTCATAACTTTGTACGGCGGTCCTAAATAGGTACGCACTACAGCTACCTATTTTGCAGAAGCCGACAGCAAGTATCTAAACACTAATCAGCTACAATGGAGTTCATCCCAACCCAAGCTTTTTACAATAGGAGGTACCAGCCTCGACCCTGGACTCCGCGCTCTACTATCCAAATCATCAGGCCCAGACCGCGCCCTCAGAGGCAAGCTGGGCAACTTGCCCAGCTGATCTCAGCAGTTAATAAACTGACAATGCGCGCGGTACCCCAACAGAAGCCACGCAGGAATCGGAAGAATAAGAAGCAAAAGCAAAAACAACAGGCGCCACAAAACAACACAAATCAAAAGAAGCAGCCACCTAAAAAGAAACCGGCTCAAAAGAAAAAGAAGCCGGGCCGCAGAGAGAGGATGTGCATGAAAATCGAAAATGATTGTATTTTCGAAGTCAAGCACGAAGGTAAGGTAACAGGTTACGCGTGCCTGGTGGGGGACAAAGTAATGAAACCAGCACATGTAAAGGGGACCATCGATAACGCGGACCTGGCCAAACTGGCCTTTAAGCGGTCATCTAAGTATGACCTTGAATGCGCGCAGATACCCGTGCACATGAAGTCCGACGCTTCGAAGTTCACCCATGAGAAACCGGAGGGGTACTACAACTGGCACCACGGAGCAGTACAGTACTCAGGAGGCCGGTTCACCATCCCTACAGGTGCTGGCAAACCAGGGGACAGCGGCAGACCGATCTTCGACAACAAGGGACGCGTGGTGGCCATAGTCTTAGGAGGAGCTAATGAAGGAGCCCGTACAGCCCTCTCGGTGGTGACCTGGAATAAAGACATTGTCACTAAAATCACCCCCGAGGGGGCCGAAGAGTGGAGTCTTGCCATCCCAGTTATGTGCCTGTTGGCAAACACCACGTTCCCCTGCTCCCAGCCCCCTTGCACGCCCTGCTGCTACGAAAAGGAACCGGAGGAAACCCTACGCATGCTTGAGGACAACGTCATGAGACCTGGGTACTATCAGCTGCTACAAGCATCCTTAACATGTTCTCCCCACCGCCAGCGACGCAGCACCAAGGACAACTTCAATGTCTATAAAGCCACAAGACCATACTTAGCTCACTGTCCCGACTGTGGAGAAGGGCACTCGTGCCATAGTCCCGTAGCACTAGAACGCATCAGAAATGAAGCGACAGACGGGACGCTGAAAATCCAGGTCTCCTTGCAAATCGGAATAAAGACGGATGACAGCCACGATTGGACCAAGCTGCGTTATATGGACAACCACATGCCAGCAGACGCAGAGAGGGCGGGGCTATTTGTAAGAACATCAGCACCGTGTACGATTACTGGAACAATGGGACACTTCATCCTGGCCCGATGTCCAAAAGGGGAAACTCTGACGGTGGGATTCACTGACAGTAGGAAGATTAGTCATTCATGTACGCACCCATTTCACCACGACCCTCCTGTGATAGGTCGGGAAAAATTCCATTCCCGACCGCAGCACGGTAAAGAGCTACCTTGCAGCACGTACGTGCAGAGCACCGCCGCAACTACCGAGGAGATAGAGGTACACATGCCCCCAGACACCCCTGATCGCACATTAATGTCACAACAGTCCGGCAACGTAAAGATCACAGTCAATGGCCAGACGGTGCGGTATAAGTGTAATTGCGGTGGCTCAAATGAAGGACTAACAACTACAGACAAAGTGATTAATAACTGCAAGGTTGATCAATGTCATGCCGCGGTCACCAATCACAAAAAGTGGCAGTATAACTCCCCTCTGGTCCCGCGTAATGCTGAACTTGGGGACCGACAAGGAAAAATTCACATCCCGTTTCCGCTGGCAAATGTAACATGCAGGGTGCCTAAAGCAAGGAACCCCACCGTGACGTACGGGAAAAACCAAGTCATCATGCTACTGTATCCTGACCACCCAACACTCCTGTCCTACCGGAATATGGGAGAAGAACCAAACTATCAAGAAGAGTGGGTGATGCATAAGAAGGAAGTCGTGCTAACCGTGCCGACTGAAGGGCTCGAGGTCACGTGGGGCAACAACGAGCCGTATAAGTATTGGCCGCAGTTATCTACAAACGGTACAGCCCATGGCCACCCGCATGAGATAATTCTGTATTATTATGAGCTGTACCCTACTATGACTGTAGTAGTTGTATCAGTGGCCACGTTCATACTCCTGTCAATGGTGGGTATGGCAGCGGGGATGTGCATGTGTGCACGACGCAGATGCATCACACCGTATGAACTGACACCAGGAGCTACCGTCCCTTTCCTGCTTAGCCTAATATGCTGCATCAGAACAGCTAAAGCGGCCACATACCAAGAGGCTGCGATATACCTGTGGAACGAGCAGCAACCTTTGTTTTGGCTACAAGCCCTTATTCCGCTGGCAGCCCTGATTGTTCTATGCAACTGTCTGAGACTCTTACCATGCTGCTGTAAAACGTTGGCTTTTTTAGCCGTAATGAGCGTCGGTGCCCACACTGTGAGCGCGTACGAACACGTAACAGTGATCCCGAACACGGTGGGAGTACCGTATAAGACTCTAGTCAATAGACCTGGCTACAGCCCCATGGTATTGGAGATGGAACTACTGTCAGTCACTTTGGAGCCAACACTATCGCTTGATTACATCACGTGCGAGTACAAAACCGTCATCCCGTCTCCGTACGTGAAGTGCTGCGGTACAGCAGAGTGCAAGGACAAAAACCTACCTGACTACAGCTGTAAGGTCTTCACCGGCGTCTACCCATTTATGTGGGGCGGCGCCTACTGCTTCTGCGACGCTGAAAATACGCAGTTGAGCGAAGCACATGTGGAGAAGTCCGAATCATGCAAAACAGAATTTGCATCAGCGTACAGGGCTCATACCGCATCTGCATCAGCTAAGCTCCGCGTCCTTTACCAAGGAAATAACATCACTGTAACTGCCTATGCAAACGGCGACCATGCCGTCACAGTTAAGGACGCCAAATTCATTGTGGGGCCAATGTCTTCAGCCTGGACACCTTTCGACAACAAAATTGTGGTGTACAAAGGTGACGTCTATAACATGGACTACCCGCCCTTTGGCGCAGGAAGACCAGGACAATTTGGCGATATCCAAAGTCGCACACCTGAGAGTAAAGACGTCTATGCTAATACACAACTGGTACTGCAGAGACCGGCTGTGGGTACGGTACACGTGCCATACTCTCAGGCACCATCTGGCTTTAAGTATTGGCTAAAAGAACGCGGGGCGTCACTGCAGCACACAGCACCATTTGGCTGCCAAATAGCAACAAACCCGGTAAGAGCGGTGAACTGCGCCGTAGGGAACATGCCCATCTCCATCGACATACCGGAAGCGGCCTTCACTAGGGTCGTCGACGCGCCCTCTTTAACGGACATGTCGTGCGAGGTACCAGCCTGCACCCATTCCTCAGACTTTGGGGGCGTCGTCATTATTAAATATGCAGCCAGCAAGAAAGGCAAGTGTGCGGTGCATTCGATGACTAACGCCGTCACTATTCGGGAAGCTGAGATAGAAGTTGAAGGGAATTCTCAGCTGCAAATCTCTTTCTCGACGGCCTTAGCCAGCGCCGAATTCCGCGTACAAGTCTGTTCTACACAAGTACACTGTGCAGCTGAGTGCCACCCCCCGAAGGACCACATAGTCAACTACCCGGCGTCACATACCACCCTCGGGGTCCAGGACATCTCCGCTACGGCGATGTCATGGGTGCAGAAGATCACGGGAGGTGTGGGACTGGTTGTTGCTGTTGCCGCACTGATTCTAATCGTGGTGCTATGCGTGTCGTTCAGCAGGCACTAACTTGACAATTAAGTATGAAGGTATATGTGTCCCCTAAGAGACACACTGTACATAGCAAATAATCTATAGATCAAAGGGCTACGCAACCCCTGAATAGTAACAAAATACAAAATCACTAAAAATTATAAAAACAGAAAAATACATAAATAGGTATACGTGTCCCCTAAGAGACACATTGTATGTAGGTGATAAGTATAGATCAAAGGGCCGAATAACCCCTGAATAGTAACAAAATATGAAAATCAATAAAAATCATAAAATAGAAAAACCATAAACAGAAGTAGTTTAAAGGGCTATAAAACCCCTGAATAGTAACAAAACATAAAGTTAATAAAAATCAAATGAATACCATAATTGGCAAACGGAAGAGATGTAGGTACTTAAGCTTCCTAAAAGCAGCCGAACT

>GU199353|2008|China, Group 4

TCTACTCTGCAAAGCAAGAGATTAATAACCCATCATGGATCCTGTGTACGTGGACATAGACGCTGACAGCGCCTTTTTGAAGGCCCTGCAACGTGCGTACCCCATGTTTGAGGTGGAACCAAGGCAGGTCACACCGAATGACCATGCTAATGCTAGAGCGTTCTCGCATCTAGCTATAAAACTAATAGAGCAGGAAATTGACCCCGACTCAACCATCCTGGATATCGGCAGTGCGCCAGCAAGGAGGATGATGTCGGACAGGAAGTACCACTGCGTCTGCCCGATGCGCAGTGCGGAAGATCCCGAGAGACTCGCTAATTATGCGAGAAAGCTAGCATCTGCCGCAGGAAAAGTCCTGGACAGAAACATCTCTGGAAAGATCGGGGACTTACAAGCAGTAATGGCCGTGCCAGACAAGGAGACGCCAACATTCTGCTTACACACAGACGTCTCATGTAGACAGAGAGCAGACGTCGCTATATACCAAGACGTCTATGCTGTACACGCACCCACGTCGCTATACCACCAGGCGATTAAAGGGGTCCGAGTGGCGTACTGGGTTGGGTTCGACACAACCCCGTTCATGTACAATGCCATGGCGGGTGCCTACCCCTCATACTCGACAAACTGGGCAGATGAGCAGGTACTGAAGGCTAAGAACATAGGATTATGTTCAACAGACCTGACGGAAGGTAGACGAGGCAAGTTGTCTATTATGAGAGGGAAAAAGCTAAAACCGTGCGACCGTGTGCTGTTCTCAGTAGGGTCAACGCTCTACCCGGAAAGCCGCAAGCTACTTAAGAGCTGGCACCTGCCATCGGTGTTCCATTTAAAGGGCAAACTCAGCTTCACATGCCGCTGTGATACAGTGGTTTCGTGTGAGGGCTACGTCGTTAAGAGAATAACGATGAGCCCAGGCCTTTATGGAAAAACCACAGGGTATGCGGTAACCCACCACGCAGACGGATTCCTGATGTGCAAGACTACCGACACGGTTGACGGCGAAAGAGTGTCATTCTCGGTGTGCACATACGTGCCGGCGACCATTTGTGATCAAATGACCGGCATCCTTGCTACAGAAGTCACGCCGGAGGATGCACAGAAGCTGTTGGTGGGGCTGAACCAGAGAATAGTGGTTAACGGCAGAACGCAACGGAATATGAACACCATGAAAAATTATCTGCTTCCCGTGGTCGCCCAAGCCTTCAGTAAGTGGGCAAAGGAGTGCCGGAAAGACATGGAAGATGAAAAACTCCTGGGGGTCAGAGAAAGAACACTGACCTGCTGCTGTCTATGGGCATTCAAGAAGCAGAAAACACACACGGTCTACAAGAGGCCGGATACCCAGTCAATTCAGAAGGTTCAGGCCGAGTTTGACAGCTTTGTGGTACCGAGTCTGTGGTCGTCCGGGTTGTCAATCCCTTTGAGGACTAGAATCAAATGGTTGTTAAGCAAGGTGCCAAAAACCGACCTGATCCCATACAGCGGAGACGCCCGAGAAGCCCGGGACGCAGAAAAAGAAGCAGAGGAAGAACGAGAAGCAGAACTGACTCGCGAAGCCCTACCACCTCTACAGGCAGCACAGGAAGATGTTCAGGTCGAAATCGACGTGGAACAGCTTGAGGACAGAGCGGGCGCAGGAATAATAGAGACTCCGAGAGGAGCTATCAAAGTTACTGCCCAACCAACAGACCACGTCGTGGGAGAGTACCTGGTACTCTCCCCGCAGACCGTACTACGTAGCCAGAAGCTCAGTCTGATTCACGCTTTGGCGGAGCAAGTGAAGACGTGCACGCACAACGGACGAGCAGGGAGGTATGCGGTCGAAGCGTACGACGGCCGAGTCCTAGTGCCCTCAGGCTATGCAATCTCGCCTGAAGACTTCCAGAGTCTAAGCGAAAGCGCAACGATGGTGTATAACGAAAGAGAGTTCGTAAACAGAAAGCTACACCATATTGCGATGCACGGACCAGCCCTGAACACCGACGAAGAGTCGTATGAGCTGGTGAGGGCAGAGAGGACAGAACACGAGTACGTCTACGACGTGGATCAGAGAAGATGCTGTAAGAAGGAAGAAGCCGCAGGACTGGTACTGGTGGGCGACTTGACTAATCCGCCCTACCACGAATTCGCATATGAAGGGCTAAAAATCCGCCCTGCCTGCCCATACAAAATTGCAGTCATAGGAGTCTTCGGAGTACCGGGATCTGGCAAGTCAGCTATTATCAAGAACCTAGTTACCAGGCAGGACCTGGTGACTAGCGGAAAGAAAGAAAACTGCCAAGAAATCACCACCGACGTGATGAGACAGAGAGGTCTAGAGATATCTGCACGTACGGTTGACTCGCTGCTCTTGAATGGATGCAACAGACCAGTCGACGTGTTGTACGTAGACGAGGCGTTTGCGTGCCACTCTGGAACGCTACTTGCTTTGATCGCCTTGGTGAGACCAAGGCAGAAAGTTGTACTTTGTGGTGACCCGAAGCAGTGCGGCTTCTTCAATATGATGCAGATGAAAGTCAACTATAATCACAACATCTGCACCCAAGTGTACCACAAAAGTATCTCCAGGCGGTGTACACTGCCTGTGACCGCCATTGTGTCATCGTTGCATTACGAAGGCAAAATGCGCACTACGAATGAGTACAACAAGCCGATTGTAGTGGACACTACAGGCTCAACAAAACCTGACCCTGGAGACCTCGTGTTAACGTGCTTCAGAGGGTGGGTTAAACAACTGCAAATTGACTATCGTGGATACGAGGTCATGACAGCAGCCGCATCCCAAGGGTTAACCAGAAAAGGAGTTTACGCAGTTAGACAAAAAGTTAATGAAAACCCGCTCTATGCATCAACGTCAGAGCACGTCAACGTACTCCTAACGCGTACGGAAGGTAAACTGGTATGGAAGACACTTTCCGGCGACCCGTGGATAAAGACGCTGCAGAACCCACCGAAAGGAAACTTCAAAGCAACTATTAAGGAGTGGGAGGTGGAGCATGCATCAATAATGGCGGGCATCTGCAGTCACCAAATGACCTTCGATACATTCCAAAATAAAGCCAACGTTTGTTGGGCTAAGAGCTTGGTCCCTATCCTCGAAACAGCGGGGATAAAACTAAATGATAGGCAGTGGTCTCAGATAATTCAAGCCTTCAAAGAAGACAAAGCATACTCACCTGAAGTAGCCCTGAATGAAATATGTACGCGCATGTATGGGGTGGATCTAGACAGCGGGCTATTTTCTAAACCGTCGGTGTCTGTGTATTACGCGGATAACCACTGGGATAATAGGCCTGGAGGGAAAATGTTCGGATTTAACCCCGAGGCAGCATCCATTCTAGAAAGAAAGTACCCATTCACAAAAGGGAAGTGGAACATCAACAAGCAGATCTGCGTGACTACCAGGAGGATAGAAGACTTTAACCCTACCACCAACATCATACCGGCCAACAGGAGACTACCACACTCATTAGTGGCCGAACACCGCCCAGTAAAAGGGGAAAGAATGGAATGGCTGGTTAACAAGATAAACGGCCACCACGTGCTCCTGGTCAGTGGCTATAACCTTGCACTGCCTACTAAGAGAGTCACTTGGGTAGCGCCGTTAGGTGTCCGCGGAGCGGACTACACATACAACCTAGAGTTGGGTCTGCCAGCAACGCTTGGTAGGTATGACCTTGTGGTCATAAACATCCACACACCTTTTCGCATACACCATTACCAACAGTGCGTCGACCACGCAATGAAACTGCAAATGCTCGGGGGTGACTCATTGAGACTGCTCAAACCGGGCGGCTCTCTATTGATCAGAGCATATGGTTACGCAGATAGAACCAGTGAACGAGTCATCTGCGTATTGGGACGCAAGTTTAGATCGTCTAGAGCGTTGAAACCACCATGTGTCACCAGCAACACTGAGATGTTTTTCCTATTCAGCAACTTTGACAATGGCAGAAGGAATTTCACAACTCATGTCATGAACAATCAACTGAATGCAGCCTTCGTAGGACAGGTCACCCGAGCAGGATGTGCACCGTCGTACCGGGTAAAACGCATGGACATCGCGAAGAACGATGAAGAGTGCGTAGTCAACGCCGCTAACCCTCGCGGGTTACCGGGTGACGGTGTTTGCAAGGCAGTATATAAAAAATGGCCGGAGTCCTTTAAGAACAGTGCAACACCAGTGGGAACCGCAAAAACAGTTATGTGTGGTACGTATCCAGTAATCCACGCTGTTGGACCAAACTTCTCTAATTATTCGGAGTCTGAAGGGGACCGGGAATTGGCAGCTGCCTATCGAGAAGTCGCAAAGGAAGTAACTAGGCTGGGAGTAAATAGTGTAGCTATACCTCTCCTCTCCACAGGTGTATACTCAGGAGGGAAAGACAGGCTGACCCAGTCACTGAACCACCTCTTTACAGCCATGGACTCGACGGATGCAGACGTGGTCATCTACTGCCGCGACAAAGAATGGGAGAAGAAAATATCTGAGGCCATACAGATGCGGACCCAAGTAGAGCTGCTGGATGAGCACATCTCCATAGACTGCGATATTGTTCGCGTGCACCCTGACAGCAGCTTGGCAGGCAGAAAAGGATACAGCACCACGGAAGGCGCACTGTACTCATATCTAGAAGGGACCCGTTTTCATCAGACGGCTGTGGATATGGCGGAGATACATACTATGTGGCCAAAGCAAACAGAGGCCAATGAGCAAGTCTGCCTATATGCCCTGGGGGAAAGTATTGAATCGATCAGGCAGAAATGCCCGGTGGATGATGCAGACGCATCATCTCCCCCCAAAACTGTCCCGTGCCTTTGCCGTTACGCTATGACTCCAGAACGCGTCACCCGGCTTCGCATGAACCACGTCACAAGCATAATTGTGTGTTCTTCGTTTCCCCTCCCAAAGTACAAAATAGAAGGAGTGCAAAAAGTCAAATGCTCTAAGGTAATGCTATTTGACCATAACGTGCCATCGCGCGTAAGTCCAAGGGAATATAGATCTTCCCAGGAGTCTGCACAGGAGGCGAGTACAATCACGTCACTGACGCATAGTCAATTCGACCTAAGCGTTGATGGCGAGATACTGCCCGTCCCGTCAGACCTGGATGCTGACGCCCCAGCCCTAGAACCAGCACTAGACGACGGGGCGACACACACGCTGCCATCCACAACCGGAAACCTTGCGGCCGTGTCTGACTGGGTAATGAGCACCGTACCTGTCGCGCCGCCCAGAAGAAGGCGAGGGAGAAACCTGACTGTGACATGTGACGAGAGAGAAGGGAATATAACACCCATGGCTAGCGTCCGATTCTTTAGGGCAGAGCTGTGTCCGGTCGTACAAGAAACAGCGGAGACGCGTGACACAGCAATGTCTCTTCAGGCACCACCGAGTACCGCCACGGAACCGAATCATCCGCCGATCTCCTTCGGAGCATCAAGCGAGACGTTCCCCATTACATTTGGGGACTTCAACGAAGGAGAAATCGAAAGCTTGTCTTCTGAGCTACTAACTTTCGGAGACTTCTTACCAGGAGAAGTGGATGACTTGACAGACAGCGACTGGTCCACGTGCTCAGACACGGACGACGAGTTATGACTAGACAGGGCAGGTGGGTATATATTCTCGTCGGACACCGGTCCAGGTCATTTACAACAGAAGTCAGTACGCCAGTCAGTGCTGCCGGTGAACACCCTGGAGGAAGTCCACGAGGAGAAGTGTTACCCACCTAAGCTGGATGAAGCAAAGGAGCAACTATTACTTAAGAAACTCCAGGAGAGTGCATCCATGGCCAACAGAAGCAGGTATCAGTCGCGCAAAGTAGAAAACATGAAAGCAGCAATCATCCAGAGACTAAAGAGAGGCTGTAGACTATACTTAATGTCAGAGACCCCAAAAGTCCCTACTTACCGGACTACATATCCGGCGCCTGTGTACTCGCCTCCGATCAACGTCCGATTGTCCAATCCCGAGTCCGCAGTGGCAGCATGCAATGAGTTCTTAGCTAGAAACTATCCAACTGTCTCGTCATACCAAATTACCGACGAGTATGATGCATATCTAGACATGGTGGACGGGTCGGAGAGTTGCCTGGACCGAGCGACATTCAATCCGTCAAAACTCAGGAGCTACCCGAAACAGCACGCTTACCACGCGCCCTCCATCAGAAGCGCTGTACCGTCCCCATTCCAGAACACACTACAGAATGTACTGGCAGCAGCCACGAAAAGAAACTGCAACGTCACACAGATGAGGGAATTACCCACTTTGGACTCAGCAGTATTCAACGTGGAGTGTTTCAAAAAATTCGCATGCAACCAAGAATACTGGGAAGAATTTGCTGCCAGCCCTATTAGGATAACAACTGAGAATTTAGCAACCTATGTTACTAAACTAAAAGGGCCAAAAGCAGCAGCGCTATTCGCAAAAACCCATAATCTACTGCCACTACAGGAAGTACCAATGGATAGGTTCACAGTAGATATGAAAAGGGACGTGAAGGTGACTCCTGGTACAAAGCATACAGAGGAAAGACCTAAGGTGCAGGTTATACAGGCGGCTGAACCCTTGGCGACAGCATACCTATGTGGGATTCACAGAGAGCTGGTTAGGAGGCTGAACGCCGTCCTCCTACCCAATGTACATACACTATTTGACATGTCTGCCGAGGATTTCGATGCCATCATAGCCGCACACTTTAAGCCAGGAGACACTGTTTTGGAAACGGACATAGCCTCCTTTGATAAGAGCCAAGACGATTCGCTTGCGCTTACTGCTTTGATGCTGTTAGAGGATTTAGGGGTGGATCACTCCCTGCTGGACTTGATAGAGGCTGCTTTCGGAGAGATTTCCAGCTGTCACCTACCGACAGGTACGCGCTTCAAGTTCGGCGCCATGATGAAATCAGGTATGTTCCTAACTCTGTTCGTCAACACATTGTTAAACATCACCATCGCCAGCCGAGTGCTGGAAGATCGTCTGACAAAATCCGCGTGCGCGGCCTTCATCGGCGACGACAACATAATACATGGAGTCGTCTCCGATGAATTGATGGCAGCCAGATGTGCCACTTGGATGAACATGGAAGTGAAGATCATAGATGCAGTTGTATCCTTGAAAGCCCCTTACTTTTGTGGAGGGTTTATACTGCACGATACTGTGACAGGAACAGCTTGCAGAGTGGCAGACCCGCTAAAAAGGCTTTTTAAACTGGGCAAACCGCTAGCGGCAGGTGACGAACAAGATGAAGATAGAAGACGAGCGCTGGCTGACGAAGTGATCAGATGGCAACGAACAGGGCTAATTGATGAGCTGGAGAAAGCGGTATACTCTAGGTACGAAGTGCAGGGTATATCAGTTGTGGTAATGTCCATGGCCACCTTTGCAAGCTCCAGATCCAATTTCGAGAAGCTCAGAGGACCCGTCATAACTTTGTACGGCGGTCCTAAATAGGTACGCACTACAGCTACCTATTTTGCAGAAGCCGACAGCAAGTATCTAAACACTAATCAGCTACAATGGAGTTCATCCCAACCCAAGCTTTTTACAATAGGAGGTACCAGCCTCGACCCTGGACTCCGCGCTCTACTATCCAAATCATCAGGCCCAGACCGCGCCCTCAGAGGCAAGCTGGGCAACTTGCCCAGCTGATCTCAGCAGTTAATAAACTGACAATGCGCGCGGTACCCCAACAGAAGCCACGCAGGAATCGGAAGAATAAGAAGCAAAAGCAAAAACAACAGGCGCCACAAAACAACACAAATCAAAAGAAGCAGCCACCTAAAAAGAAACCGGCTCAAAAGAAAAAGAAGCCGGGCCGCAGAGAGAGGATGTGCATGAAAATCGAAAATGATTGTATTTTCGAAGTCAAGCACGAAGGTAAGGTAACAGGTTACGCGTGCCTGGTGGGGGACAAAGTAATGAAACCAGCACATGTAAAGGGGACCATCGATAACGCGGACCTGGCCAAACTGGCCTTTAAGCGGTCATCTAAGTATGACCTTGAATGCGCGCAGATACCCGTGCACATGAAGTCCGACGCTTCGAAGTTCACCCATGAGAAACCGGAGGGGTACTACAACTGGCACCACGGAGCAGTACAGTACTCAGGAGGCCGGTTCACCATCCCTACAGGTGCTGGCAAACCAGGGGACAGCGGCAGACCGATCTTCGACAACAAGGGACGCGTGGTGGCCATAGTCTTAGGAGGAGCTAATGAAGGAGCCCGTACAGCCCTCTCGGTGGTGACCTGGAATAAAGACATTGTCACTAAAATCACCCCCGAGGGGGCCGAAGAGTGGAGTCTTGCCATCCCAGTTATGTGCCTGTTGGCAAACACCACGTTCCCCTGCTCCCAGCCCCCTTGCACGCCCTGCTGCTACGAAAAGGAACCGGAGGAAACCCTACGCATGCTTGAGGACAACGTCATGAGACCTGGGTACTATCAGCTGCTACAAGCATCCTTAACATGTTCTCCCCACCGCCAGCGACGCAGCACCAAGGACAACTTCAATGTCTATAAAGCCACAAGACCATACTTAGCTCACTGTCCCGACTGTGGAGAAGGGCACTCGTGCCATAGTCCCGTAGCACTAGAACGCATCAGAAATGAAGCGACAGACGGGACGCTGAAAATCCAGGTCTCCTTGCAAATCGGAATAAAGACGGATGACAGCCACGATTGGACCAAGCTGCGTTATATGGACAACCACATGCCAGCAGACGCAGAGAGGGCGGGGCTATTTGTAAGAACATCAGCACCGTGTACGATTACTGGAACAATGGGACACTTCATCCTGGCCCGATGTCCAAAAGGGGAAACTCTGACGGTGGGATTCACTGACAGTAGGAAGATTAGTCATTCATGTACGCACCCATTTCACCACGACCCTCCTGTGATAGGTCGGGAAAAATTCCATTCCCGACCGCAGCACGGTAAAGAGCTACCTTGCAGCACGTACGTGCAGAGCACCGCCGCAACTACCGAGGAGATAGAGGTACACATGCCCCCAGACACCCCTGATCGCACATTAATGTCACAACAGTCCGGCAACGTAAAGATCACAGTCAATGGCCAGACGGTGCGGTATAAGTGTAATTGCGGTGGCTCAAATGAAGGACTAACAACTACAGACAAAGTGATTAATAACTGCAAGGTTGATCAATGTCATGCCGCGGTCACCAATCACAAAAAGTGGCAGTATAACTCCCCTCTGGTCCCGCGTAATGCTGAACTTGGGGACCGACAAGGAAAAATTCACATCCCGTTTCCGCTGGCAAATGTAACATGCAGGGTGCCTAAAGCAAGGAACCCCACCGTGACGTACGGGAAAAACCAAGTCATCATGCTACTGTATCCTGACCACCCAACACTCCTGTCCTACCGGAATATGGGAGAAGAACCAAACTATCAAGAAGAGTGGGTGATGCATAAGAAGGAAGTCGTGCTAACCGTGCCGACTGAAGGGCTCGAGGTCACGTGGGGCAACAACGAGCCGTATAAGTATTGGCCGCAGTTATCTACAAACGGTACAGCCCATGGCCACCCGCATGAGATAATTCTGTATTATTATGAGCTGTACCCTACTATGACTGTAGTAGTTGTATCAGTGGCCACGTTCATACTCCTGTCAATGGTGGGTATGGCAGCGGGGATGTGCATGTGTGCACGACGCAGATGCATCACACCGTATGAACTGACACCAGGAGCTACCGTCCCTTTCCTGCTTAGCCTAATATGCTGCATCAGAACAGCTAAAGCGGCCACATACCAAGAGGCTGCGATATACCTGTGGAACGAGCAGCAACCTTTGTTTTGGCTACAAGCCCTTATTCCGCTGGCAGCCCTGATTGTTCTATGCAACTGTCTGAGACTCTTACCATGCTGCTGTAAAACGTTGGCTTTTTTAGCCGTAATGAGCGTCGGTGCCCACACTGTGAGCGCGTACGAACACGTAACAGTGATCCCGAACACGGTGGGAGTACCGTATAAGACTCTAGTCAATAGACCTGGCTACAGCCCCATGGTATTGGAGATGGAACTACTGTCAGTCACTTTGGAGCCAACACTATCGCTTGATTACATCACGTGCGAGTACAAAACCGTCATCCCGTCTCCGTACGTGAAGTGCTGCGGTACAGCAGAGTGCAAGGACAAAAACCTACCTGACTACAGCTGTAAGGTCTTCACCGGCGTCTACCCATTTATGTGGGGCGGCGCCTACTGCTTCTGCGACGCTGAAAATACGCAGTTGAGCGAAGCACATGTGGAGAAGTCCGAATCATGCAAAACAGAATTTGCATCAGCGTACAGGGCTCATACCGCATCTGCATCAGCTAAGCTCCGCGTCCTTTACCAAGGAAATAACATCACTGTAACTGCCTATGCAAACGGCGACCATGCCGTCACAGTTAAGGACGCCAAATTCATTGTGGGGCCAATGTCTTCAGCCTGGACACCTTTCGACAACAAAATTGTGGTGTACAAAGGTGACGTCTATAACATGGACTACCCGCCCTTTGGCGCAGGAAGACCAGGACAATTTGGCGATATCCAAAGTCGCACACCTGAGAGTAAAGACGTCTATGCTAATACACAACTGGTACTGCAGAGACCGGCTGTGGGTACGGTACACGTGCCATACTCTCAGGCACCATCTGGCTTTAAGTATTGGCTAAAAGAACGCGGGGCGTCACTGCAGCACACAGCACCATTTGGCTGCCAAATAGCAACAAACCCGGTAAGAGCGGTGAACTGCGCCGTAGGGAACATGCCCATCTCCATCGACATACCGGAAGCGGCCTTCACTAGGGTCGTCGACGCGCCCTCTTTAACGGACATGTCGTGCGAGGTACCAGCCTGCACCCATTCCTCAGACTTTGGGGGCGTCGCCATTATTAAATATGCAGCCAGCAAGAAAGGCAAGTGTGCGGTGCATTCGATGACTAACGCCGTCACTATTCGGGAAGCTGAGATAGAAGTTGAAGGGAATTCTCAGCTGCAAATCTCTTTCTCGACGGCCTTAGCCAGCGCCGAATTCCGCGTACAAGTCTGTTCTACACAAGTACACTGTGCAGCTGAGTGCCACCCCCCGAAGGACCACATAGTCAACTACCCGGCGTCACATACCACCCTCGGGGTCCAGGACATCTCCGCTACGGCGATGTCATGGGTGCAGAAGATCACGGGAGGTGTGGGACTGGTTGTTGCTGTTGCCGCACTGATTCTAATCGTGGTGCTATGCGTGTCGTTCAGCAGGCACTAACTTGACAATTAAGTATGAAGGTATATGTGTCCCCTAAGAGACACACTGTACATAGCAAATAATCTATAGATCAAAGGGCTACGCAACCCCTGAATAGTAACAAAATACAAAATCACTAAAAATTATAAAAACAGAAAAATACATAAATAGGTATACGTGTCCCCTAAGAGACACATTGTATGTAGGTGATAAGTATAGATCAAAGGGCCGAATAACCCCTGAATAGTAACAAAATATGAAAATCAATAAAAATCATAAAATAGAAAAACCATAAACAGAAGTAGTTTAAAGGGCTATAAAACCCCTGAATAGTAACAAAACATAAAGTTAATAAAAATCAAATGAATACCATAATTGGCAAACGGAAGAGATGTAGGTACTTAAGCTTCCTAAAAGCAGCCGAACTCACTTGAGAA

>JX088705|2010|China, Group 4

ATGGCTGCGTGAGACACACGTAGCCTACCAGTTTCTTACTGCTCTACTCTGCAAAGCAAGAGATTAATAACCCATCATGGATCCTGTGTACGTGGACATAGACGCTGACAGCGCCTTTTTGAAGGCCCTGCAACGTGCGTACCCCATGTTTGAGGTGGAACCAAGGCAGGTCACACCGAATGACCATGCTAATGCTAGAGCGTTCTCGCATCTAGCTATAAAACTAATAGAGCAGGAAATTGATCCCGACTCAACCATCCTGGATATCGGCAGTGCGCCAGCAAGGAGGATGATGTCGGACAGGAAGTACCACTGCGTCTGCCCGATGCGCAGTGCGGAAGATCCCGAGAGACTCGCTAATTATGCGAGAAAGCTAGCATCTGCCGCAGGAAAAGTCCTGGACAGAAACATCTCTGGAAAGATCGGGGACTTACAAGCAGTAATGGCCGTGCCAGACAAGGAGACGCCAACATTCTGCTTACACACAGACGTCTCATGTAGACAGAGAGCAGACGTCGCTATATACCAAGACGTCTATGCTGTATACGCACCCACGTCGCTATACCACCAGGCGATTAAAGGGGTCCGAGTGGCGTACTGGGTTGGGTTCGACACAACCCCGTTCATGTACAATGCCATGGCGGGTGCCTACCCCTCATACTCGACAAACTGGGCAGATGAGCAGGTACTGAAGGCTAAGAACATAGGATTATGTTCAACAGACCTGACGGAAGGTAGACGAGGCAAGTTGTCTATTATGAGAGGGAAAAAGCTAAAACCGTGCGACCGTGTGCTGTTCTCAGTAGGGTCAACGCTCTACCCGGAAAGCCGCAAGCTACTTAAGAGCTGGCACCTGCCATCGGTGTTCCATTTAAAGGGCAAACTCAGCTTCACATGCCGCTGTGATACAGTGGTTTCGTGTGAGGGCTACGTCGTTAAGAGAATAACGATGAGCCCGGGCCTTTATGGAGAAACCACAGGGTATGCGGTAACCCACCACGCAGACGGATTCCTGATGTGCAAGACTACCGACACGGTTGACGGCGAAAGAGTGTCATTCTCGGTGTGCACATACGTGCCGGCGACCATTTGTGATCAAATGACCGGCATCCTTGCTACGGAAGTCACGCCGGAGGATGCACAGAAGCTGTTGGTGGGGCTGAACCAGAGAATAGTGGTTAACGGCAGAACGCAACGGAATATGAACACCATGAAAAATTATCTGCTTCCCGTGGTCGCCCAAGCCTTCAGTAAGTGGGCAAAGGAGTGCCGGAAAGACATGGAAGATGAAAAACCCCTGGGGGTCAGAGAAAGAACACTGACCTGCTGCTGTCTATGGGCATTCAAGAAGCAGAAAACACACACGGTCTACAAGAGGCCGGATACCCAGTCAATTCAGAAGGTTCAGGCCGAGTTTGACAGCTTTGTGGTACCGAGTCTGTGGTCGTCCGGGTTGTCAATCCCTTTGAGGACTAGAATCAAATGGTTGTTAAGCAAGGTGCCAAAAACCGACCTGATCCCATACAGCGGAGACGCCCGAGAAGCCCGGGACGCAGAAAAAGAAGCAGAGGAAGAACGAGAAGCAGAACTGACTCGCGAAGCCCTACCACCTCTACAGGCAGCACAGGAAGATGTTCAGGTCGAAATCGACGTGGAACAGCTTGAGGACAGAGCGGGTGCAGGAATAATAGAGACTCCGAGAGGAGCTATCAAAGTTACTGCCCAACCAACAGACCACGTCGTGGGAGAGTACCTGGTACTCTCCCCGCAGACCGTACTACGTAGCCAGAAGCTCAGTCTGATTCACGCTTTGGCGGAGCAAGTGAAGACGTGCACGCACAACGGACGAGCAGGGAGGTATGCGGTCGAAGCGTACGACGGCCGAGTCCTAGTGCCCTCAGGCTATGCAATTTCGCCTGAAGACTTCCAGAGTCTAAGCGAAAGCGCAACGATGGTGTATAACGAAAGAGAGTTCGTAAACAGAAAGCTACACCATATTGCGATGCACGGACCAGCCCTGAACACCGACGAAGAGTCGTATGAGCTGGTGAGGGCAGAGAGGACAGAACACGAGTACGTCTACGACGTGGATCAGAGAAGATGCTGTAAGAAGGAAGAAGCCGCGGGACTGGTACTGGTGGGCGACTTGACTAATCCGCCCTACCACGAATTCGCATATGAAGGGCTAAAAATCCGCCCTGCCTGCCCATACAAAATTGCAGTCATAGGAGTCTTCGGAGTACCGGGATCTGGCAAGTCAGCTATTATCAAGAACCTAGTTACCAGGCAGGACCTGGTGACTAGCGGAAAGAAAGAAAACTGCCAAGAAATCACCACCGACGTGATGAGACAGAGAGGTCTAGAGATATCTGCACGTACGGTTGACTCGCTGCTCTTGAATGGATGCAACAGACCAGTCGACGTGTTGTACGTAGACGAGGCGTTTGCGTGCCACTCTGGAACGCTACTTGCTTTGATCGCCTTGGTGAGACCAAGGCAGAAAGTTGTACTTTGTGGTGACCCGAAGCAGTGCGGCTTCTTCAATATGATGCAGATGAAAGTCAACTATAATCACAACATCTGCACCCAAGTGTACCACAAAAGTATCTCCAGGCGGTGTACACTGCCTGTGACCGCCATCGTGTCATCGTTGCATTACGAAGGCAAAATGCGCACTACGAATGAGTACAACAAGCCGATTGTAGTGGACACTACAGGCTCAACAAAACCTGACCCTGGAGACCTCGTGTTAACGTGCTTCAGAGGGTGGGTTAAACAACTGCAAATTGACTATCGTGGATACGAGGTCATGACAGCAGCCGCATCCCAAGGGTTAACCAGAAAAGGAGTTTACGCAGTTAGACAAAAAGTTAATGAAAACCCGCTCTATGCATCAACGTCAGAGCACGTCAACGTACTCCTAACGCGTACGGAAGGTAAACTGGTATGGAAGACACTTTCCGGCGACCCGTGGATAAAGACGCTGCAGAACCCACCGAAAGGAAACTTCAAAGCAACTATTAAGGAGTGGGAGGTGGAGCATGCATCAATAATGGCGGGCATCTGCAGTCACCAAATGACCTTCGATACATTCCAAAATAAAGCCAACGTTTGTTGGGCTAAGAGCTTGGTCCCTATCCTCGAAACAGCGGGGATAAAACTAAATGATAGGCAGTGGTCTCAGATAATTCAAGCCTTCAAAGAAGACAAAGCATACTCACCTGAAGTAGCCCTGAATGAAATATGTACGCGCATGTATGGGGTGGATCTAGACAGCGGGCTATTTTCTAAACCGTCGGTGTCTGTGTATTACGCGGATAACCACTGGGATAATAGGCCTGGAGGGAAAATGTTCGGATTTAACCCCGAGGCAGCATCCATTCTAGAAAGAAAGTATCCATTCACAAAAGGGAAGTGGAACATCAACAAGCAGATCTGCGTGACTACCAGGAGGATAGAAGACTTTAACCCTACCACCAACATCATACCGGCCAACAGGAGACTACCACACTCATTAGTGGCCGAACACCGCCCAGTAAAAGGGGAAAGAATGGAATGGCTGGTTAACAAGATAAACGGCCACCACGTGCTCCTGGTCAGTGGCTATAACCTTGCACTGCCTACTAAGAGAGTCACTTGGGTAGCGCCGTTAGGTGTCCGCGGAGCGGACTACACATACAACCTAGAGTTGGGTCTGCCAGCAACGCTTGGTAGGTATGACCTTGTGGTCATAAACATCCACACACCTTTTCGCATACACCATTACCAACAGTGCGTCGACCACGCAATGAAACTGCAAATGCTCGGGGGTGACTCATTGAGACTGCTCAAACCGGGCGGCTCTCTATTGATCAGAGCATATGGTTACGCAGATAGAACCAGTGAACGAGTCATCTGCGTATTGGGACGCAAGTTTAGATCGTCTAGAGCGTTGAAACCACCATGTGTCACCAGCAACACTGAGATGTTTTTCCTATTCAGCAACTTTGACAATGGCAGAAGGAATTTCACAACTCATGTCATGAACAATCAACTGAATGCAGCCTTCGTAGGACAGGTCACCCGAGCAGGATGTGCACCGTCGTACCGGGTAAAACGCATGGACATCGCGAAGAACGATGAAGAGTGCGTAGTCAACGCCGCTAACCCTCGCGGGTTACCGGGTGACGGTGTTTGCAAGGCAGTATACAAAAAATGGCCGGAGTCCTTTAAGAACAGTGCAACACCAGTGGGAACCGCAAAAACAGTTATGTGCGGTACGTATCCAGTAATCCACGCTGTTGGACCAAACTTCTCTAATTATTCGGAGTCTGAAGGGGACCGGGAATTGGCAGCTGCCTATCGAGAAGTCGCAAAGGAAGTAACTAGGCTGGGAGTAAATAGTGTAGCTATACCTCTCCTCTCCACAGGTGTATACTCAGGAGGGAAAGACAGGCTGACCCAGTCACTGAACCACCTCTTTACAGCCATGGACTCGACGGATGCAGACGTGGTCATCTACTGCCGCGACAAAGAATGGGAGAAGAAAATATCTGAGGCCATACAGATGCGGACCCAAGTAGAGCTGCTGGATGAGCACATCTCCATAGACTGCGATATTGTTCGCGTGCACCCTGACAGCAGCTTGGCAGGCAGAAAAGGATACAGCACCACGGAAGGCGCACTGTACTCATATCTAGAAGGGACCCGTTTTCATCAGACGGCTGTGGATATGGCGGAGATACATACTATGTGGCCAAAGCAAACAGAGGCCAATGAGCAAGTCTGCCTATATGCCCTGGGGGAAAGTATTGAATCGATCAGGCAGAAATGCCCGGTGGATGATGCAGACGCATCATCTCCCCCCAAAACTGTCCCGTGCCTTTGCCGTTACGCTATGACTCCAGAACGCGTCACTCGGCTTCGCATGAACCACGTCACAAGCATAATTGTGTGTTCTTCGTTTCCCCTCCCAAAGTACAAAATAGAAGGAGTGCAAAAAGTCAAATGCTCTAAGGTAATGCTATTTGACCATAACGTGCCATCGCGCGTAAGTCCAAGGGAATATAGATCTTCCCAGGAGTCTGCACAGGAGGCGAGTACAATCACGTCACTGACGCATAGTCAATTCGACCTAAGCGTTGATGGCGAGATACTGCTCGTCCCGTCAGACCTGGATGCTGACGCCCCAGCCCTAGAACCAGCACTAGACGACGGGGCGACACACACGCTGCCATCCACAACCGGAAACCTTGCGGCCGTGTCTGACTGGGTAATGAGCACCGTACCTGTCGCGCCGCCCAGAAGAAGGCGAGGGAGAAACCTGACTGTGACATGTGACGAGAGAGAAGGGAATATAACACCCATGGCTAGCGTCCGATTCTTTAGGGCAGAGCTGTGTCCGGTCGTACAAGAAACAGCGGAGACGCGTGACACAGCAATGTCTCTTCAGGCACCACCGAGTACCGCCACGGAACCGAATCATCCGCCGATCTCCTTCGGAGCATCAAGCGAGACGTTCCCCATTACATTTGGGGACTTCAACGAAGGAGAAATCGAAAGCTTGTCTTCTGAGCTACTAACTTTCGGAGACTTCTTACCAGGAGAAGTGGATGACTTGACAGACAGCGACTGGTCCACGTGCTCAGACACGGACGACGAGTTACGACTAGACAGGGCAGGTGGGTATATATTCTCGTCGGACACCGGTCCAGGTCATTTACAACAGAAGTCAGTACGCCAGTCAGTGCTGCCGGTGAACACCCTGGAGGAAGTCCACGAGGAGAAGTGTTACCCACCTAAGCTGGATGAAGCAAAGGAGCAACTATTACTTAAGAAACTCCAGGAGAGTGCATCCATGGCCAACAGAAGCAGGTATCAGTCGCGCAAAGTAGAAAACATGAAAGCAGCAATCATCCAGAGACTAAAGAGTGGCTGTAGACTATACTTAATGTCAGAGACCCCAAAAGTCCCTACTTACCGGACTACATATCCGGCGCCTGTGTACTCGCCTCCGATCAACGTCCGATTGTCCAATCCCGAGTCCGCAGTGGCAGCATGCAATGAGTTCTTAGCTAGAAACTATCCAACTGTCTCGTCATACCAAATTACCGACGAGTATGATGCATATCTAGACATGGTGGACGGGTCGGAGAGTTGCCTGGACCGAGCGACATTTAATCCGTCAAAACTCAGGAGCTACCCGAAACAGCACGCTTACCACGCGCCCTCCATCAGAAGCGCTGTACCGTCCCCATTCCAGAACACACTACAGAATGTACTGGCAGCAGCCACGAAAAGAAACTGCAACGTCACACAGATGAGGGAATTACCCACTTTGGACTCAGCAGTATTCAACGTGGAGTGTTTCAAAAAATTCGCATGCAACCAAGAATACTGGGAAGAATTTGCTGCCAGCCCTATTAGGATAACAACTGAGAATTTAGCAACCTATGTTACTAAACTAAAAGGGCCAAAAGCAGCAGCGCTATTCGCAAAAACCCATAATCTACTGCCACTACAGGAAGTACCAATGGATAGGTTCACAGTAGATATGAAAAGGGACGTGAAGGTGACTCCTGGTACAAAGCATACAGAGGAAAGACCTAAGGTGCAGGTTATACAGGCGGCTGAACCCTTGGCGACAGCATACCTATGTGGGATTCACAGAGAGCTGGTTAGGAGGCTGAACGCCGTCCTCCTACCCAATGTACATACACTATTTGACATGTCTGCCGAGGATTTCGATGCCATCATAGCCGCACACTTTAAGCCAGGAGACACTGTTTTGGAAACGGACATAGCCTCCTTTGATAAGAGCCAAGATGATTCGCTTGCGCTTACTGCTTTGATGCTGTTAGAGGATTTAGGGGTAGATCACTCCCTGCTGGACTTGATAGAGGCTGCTTTCGGAGAGATTTCCAGCTGCCACCTACCGACAGGTACGCGCTTCAAGTTCGGCGCCATGATGAAATCAGGTATGTTCCTAACTCTGTTCGTCAACACATTGTTAAACATCACCATCGCCAGCCGAGTGCTGGAAGATCGTCTGACAAAATCCGCGTGCGCGGCCTTCATCGGCGACGACAACATAATACATGGAGTCGTCTCCGATGAATTGATGGCAGCCAGATGTGCCACTTGGATGAACATGGAAGTGAAGATCATAGATGCAGTTGTATCCTTGAAAGCCCCTTACTTTTGTGGAGGGTTTATACTGCACGATACTGTGACAGGAACAGCTTGCAGAGTGGCAGACCCGCTAAAAAGGCTTTTTAAACTGGGCAAACCGCTAGCGGCAGGTGACGAACAAGATGAAGATAGAAGACGAGCGCTGGCTGACGAAGTGATCAGATGGCAACGAACAGGGCTAATTGATGAGCTGGAGAAAGCGGTATACTCTAGGTACGAAGTGCAGGGTATATCAGTTGTGGTAATGTCCATGGCCACCTTTGCAAGCTCCAGATCCAATTTCGAGAAGCTCAGAGGACCCGTCATAACTTTGTACGGCGGTCCTAAATAGGTACGCACTACAGCTACCTATTTTGCAGAAGCCGACAGCAAGTATCTAAACACTAATCAGCTACAATGGAGTTCATCCCAACCCAAACTTTTTACAATAGGAGGTACCAGCCTCGACCCTGGACTCCGCGCTCTACTATCCAAATCATCAGGCCCAGACCGCGCCCTCAGAGGCAAGCTGGGCAACTTGCCCAGCTGATCTCAGCAGTTAATAAACTGACAATGCGCGCGGTACCCCAACAGAAGCCACGCAGGAATCGGAAGAATAAGAAGCAAAAGCAAAAACAACAGGCGCCACAAAACAACACAAATCAAAAGAAGCAGCCACCTAAAAAGAAACCGGCTCAAAAGAAAAAGAAGCCGGGCCGCAGAGAGAGGATGTGCATGAAAATCGAAAATGATTGTATTTTCGAAGTCAAGCACGAAGGTAAGGTAACAGGTTACGCGTGCCTGGTGGGGGACAAAGTAATGAAACCAGCACATGTAAAGGGGACCATCGATAACGCGGACCTGGCCAAATTGGCCTTTAAGCGGTCATCTAAGTATGACCTTGAATGCGCGCAGATACCCGTGCACATGAAGTCCGACGCTTCGAAGTTCACCCATGAGAAACCGGAGGGGTACTACAACTGGCACCACGGAGCAGTACAGTACTCAGGAGGCCGGTTCACCATCCCTACAGGTGCTGGCAAACCAGGGGACAGCGGCAGACCGATCTTCGACAACAAGGGACGCGTGGTGGCCATAGTCTTAGGAGGAGCTAATGAAGGAGCCCGTACAGCCCTCTCGGTGGTGACCTGGAATAAAGACATTGTCACTAAAATCACCCCCGAGGGGGCCGAAGAGTGGAGTCTTGCCATTCCAGTTATGTGCCTGTTGGCAAACACCACGTTCCCCTGCTCCCAGCCCCCTTGCACGCCCTGCTGCTACGAAAAGGAACCGGAGGAAACCCTACGCATGCTTGAGGACAACGTCATGAGACCTGGGTACTATCAGCTGCTACAAGCATCCTTAACATGTTCTCCCCACCGCCAGCGACGCAGCACCAAGGACAACTTCAATGTCTATAAAGCCACAAGACCATACTTAGCTCACTGTCCCGACTGTGGAGAAGGGCACTCGTGCCATAGTCCCGTAGCACTAGAACGCATCAGAAATGAAGCGACAGACGGGACGCTGAAAATCCAGGTCTCCTTGCAAATCGGAATAAAGACGGATGACAGCCACGATTGGACCAAGCTGCGTTATATGGACAACCACATGCCAGCAGACGCAGAGAGGGCGGGGCTATTTGTAAGAACATCAGCACCGTGTACGATTACTGGAACAATGGGACACTTCATCCTGGCCCGATGTCCAAAAGGGGAAACTCTGACGGTGGGATTCACTGACAGTAGGAAGATTAGTCATTCATGTACGCACCCATTTCACCACGACCCTCCTGTGATAGGTCGGGAAAAATTCCATTCCCGACCGCAGCACGGTAAAGAGCTACCTTGCAGCACGTACGTGCAGAGCACCGCCGCAACTACCGAGGAGATAGAGGTACACATGCCCCCAGACACCCCTGATCGCACATTAATGTCACAACAGTCCGGCAACGTAAAGATCACAGTCAATGGCCAGACGGTGCGGTATAAGTGTAATTGCGGTGGCTCAAATGAAGGACTAACAACTACAGACAAAGTGATTAATAACTGCAAGGTTGATCAATGTCATGCCGCGGTCACCAATCACAAAAAGTGGCAGTATAACTCCCCTCTGGTCCCGCGTAATGCTGAACTTGGGGACCGACAAGGAAAAATTCACATCCCGTTTCCGCTGGCAAATGTAACATGCAGGGTGCCTAAAGCAAGGAACCCCACCGTGACGTACGGGAAAAACCAAGTCATCATGCTACTGTATCCTGACCACCCAACACTCCTGTCCTACCGGAATATGGGAGAAGAACCAAACTATCAAGAAGAGTGGGTGATGTATAAGAAGGAAGTCGTGCTAACCGTGCCGACTGAAGGGCTCGAGGTCACGTGGGGCAACAACGAGCCGTATAAGTATTGGCCGCAGTTATCTACAAACGGTACAGCCCATGGCCACCCGCATGAGATAATTCTGTATTATTATGAGCTGTACCCTACTATGACTGTAGTAGTTGTATCAGTGGCCACGTTCATACTCCTGTCAATGGTGGGTATGGCAGCGGGGATGTGCATGTGTGCACGACGCAGATGCATCACACCGTATGAACTGACACCAGGAGCTACCGTCCCTTTCCTGCTTAGCCTAATATGCTGCATCAGAACAGCTAAAGCGGCCACATACCAAGAGGCTGCGATATACCTGTGGAACGAGCAGCAACCTTTGTTTTGGCTACAAGCCCTTATTCCGCTGGCAGCCCTGATTGTTCTATGCAACTGTCTGAGACTCTTACCATGCTGCTGTAAAACGTTGGCTTTTTTAGCCGTAATGAGCGTCGGTGCCCACACTGTGAGCGCGTACGAACACGTAACAGTGATCCCGAACACGGTGGGAGTACCGTATAAGACTCTAGTCAATAGACCTGGCTACAGCCCCATGGTATTGGAGATGGAACTACTGTCAGTCACTTTGGAGCCAACACTATCGCTTGATTACATCACGTGCGAGTACAAAACCGTCATCCCGTCTCCGTACGTGAAGTGCTGCGGTACAGCAGAGTGCAAGGACAAAAACCTACCTGACTACAGCTGTAAGGTCTTCACCGGCGTCTACCCATTTATGTGGGGCGGCGCCTACTGCTTCTGCGACGCTGAAAATACGCAGTTGAGCGAAGCACATGTGGAGAAGTCCGAATCATGCAAAACAGAATTTGCATCAGCGTACAGGGCTCATACCGCATCTGCATCAGCTAAGCTCCGCGTCCTTTACCAAGGAAATAACATCACTGTAACTGCCTATGCAAACGGCGACCATGCCGTCACAGTTAAGGACGCCAAATTCATTGTGGGGCCAATGTCTTCAGCCTGGACACCTTTCGACAACAAAATTGTGGTGTACAAAGGTGACGTCTATAACATGGACTACCCGCCCTTTGGCGCAGGAAGACCAGGACAATTTGGCGATATCCAAAGTCGCACACCTGAGAGTAAAGACGTCTATGCTAATACACAACTGGTACTGCAGAGACCGGCTGTGGGTACAGTACACGTGCCATACTCTCAGGCACCATCTGGCTTTAAGTATTGGCTAAAAGAACGCGGGGCGCCACTGCAGCACACAGCACCATTTGGCTGCCAAATAGCAACAAACCCGGTAAGAGCGGTGAACTGCGCCGTAGGGAACATGCCCATCTCCATCGACATACCGGAAGCGGCCTTCACTAGGGTCGTCGACGCGCCCTCTTTAACGGACATGTCGTGCGAGGTACCAGCCTGCACCCATTCCTCAGACTTTGGTGGCGTCGCCATTATTAAATATGCAGCCAGCAAGAAAGGCAAGTGTGCGGTGCATTCGATGACTAACGCCGTCACTATTCGGGAAGCTGAGATAGAAGTTGAAGGGAATTCTCAGCTGCAAATCTCTTTCTCGACGGCCTTAGCCAGCGCCGAATTCCGCGTACAAGTCTGTTCTACACAAGTACACTGTGCAGCTGAGTGCCACCCCCCGAAGGACCACATAGTCAACTACCCGGCGTCACATACCACCCTCGGGGTCCAGGACATCTCCGCTACGGCGATGTCATGGGTGCAGAAGATCACGGGAGGTGTGGGACTGGTTGTTGCTGTTGCCGCACTGATTCTAATCGTGGTGCTATGCGTGTCGTTCAGCAGGCACTAACTTGACAATTAAGTATGAAGGTATATGTGTCCCCTAAGAGACACACTGTACATAGCAAATAATCTATAGATCAAAGGGCTACGCAACCCCTGAATAGTAACAAAACACAAAATCACTAAAAATTATAAAAACAGAAAAATACATAAATAGGTATACGTGTCCCCTAAGAGACACATTGTATGTAGGTGATAAGTATAGATTAAAGGGCCGAATAACCCCTGAATAGTAACAAAATATGAAAATCAATAAAAATCATAAAATAGAAAAACCATAAACAGAAGTAGTTTAAAGGGCTATAAAACCCCTGAATAGTAACAAAACATAAAGTTAATAAAAATCAAATGAATACCATAATTGGCAAACGGAAGAGATGTAGGTACTTAAGCTTCCTAAAAGCAGCCGAACTCACTTTGAGAAGTAGGCATAGCATACCGAACTCTTCCACGATTCTCCGAACCCACAGGGACGTAGGAGATGTTATTTTGTTTTTAATATTTC

>FR717336|2005|France: La Reunion, Group 4

CAAAGCAAGAGATTAATAACCCATCATGGATCCTGTGTACGTGGACATAGACGCTGACAGCGCCTTTTTGAAGGCCCTGCAACGTGCGTACCCCATGTTTGAGGTGGAACCAAGGCAGGTCACACCGAATGACCATGCTAATGCTAGAGCGTTCTCGCATCTAGCTATAAAACTAATAGAGCAGGAAATTGACCCCGACTCAACCATCCTGGATATCGGCAGTGCGCCAGCAAGGAGGATGATGTCGGACAGGAAGTACCACTGCGTCTGCCCGATGCGCAGTGCGGAAGATCCCGAGAGACTCGCCAATTATGCGAGAAAGCTAGCATCTGCCGCAGGAAAAGTCCTGGACAGAAACATCTCTGGAAAGATCGGGGACTTACAAGCAGTAATGGCCGTGCCAGACACGGAGACGCCAACATTCTGCTTACACACAGACGTCTCATGTAGACAGAGAGCAGACGTCGCTATATACCAAGACGTCTATGCTGTACACGCACCCACGTCGCTATACCACCAGGCGATTAAAGGGGTCCGAGTGGCGTACTGGGTTGGGTTCGACACAACCCCGTTCATGTACAATGCCATGGCGGGTGCCTACCCCTCATACTCGACAAACTGGGCAGATGAGCAGGTACTGAAGGCTAAGAACATAGGATTATGTTCAACAGACCTGACGGAAGGTAGACGAGGCAAGTTGTCTATTATGAGAGGGAAAAAGCTAAAACCGTGCGACCGTGTGCTGTTCTCAGTAGGGTCAACGCTCTACCCGGAAAGCCGCAAGCTACTTAAGAGCTGGCACCTGCCATCGGTGTTCCATTTAAAGGGCAAACTCAGCTTCACATGCCGCTGTGATACAGTGGTTTCGTGTGAGGGCTACGTCGTTAAGAGAATAACGATGAGCCCAGGCCTTTATGGAAAAACCACAGGGTATGCGGTAACCCACCACGCAGACGGATTCCTGATGTGCAAGACTACCGACACGGTTGACGGCGAAAGAGTGTCATTCTCGGTGTGCACATACGTGCCGGCGACCATTTGTGATCAAATGACCGGCATCCTTGCTACAGAAGTCACGCCGGAGGATGCACAGAAGCTGTTGGTGGGGCTGAACCAGAGAATAGTGGTTAACGGCAGAACGCAACGGAATACGAACACCATGAAAAATTATCTGCTTCCCGTGGTCGCCCAAGCCTTCAGTAAGTGGGCAAAGGAGTGCCGGAAAGACATGGAAGATGAAAAACTCCTGGGGGTCAGAGAAAGAACACTGACCTGCTGCTGTCTATGGGCATTCAAGAAGCAGAAAACACACACGGTCTACAAGAGGCCTGATACCCAGTCAATTCAGAAGGTTCAGGCCGAGTTTGACAGCTTTGTGGTACCGAGTCTGTGGTCGTCCGGGTTGTCAATCCCTTTGAGGACTAGAATCAAATGGTTGTTAAGCAAGGTGCCAAAAACCGACCTGATCCCATACAGCGGAGACGCCCGAGAAGCCCGGGACGCAGAAAAAGAAGCAGAGGAAGAACGAGAAGCAGAACTGACTCGCGAAGCCCTACCACCTCTACAGGCAGCACAGGAAGATGTTCAGGTCGAAATCGACGTGGAACAGCTTGAGGACAGAGCGGGCGCAGGAATAATAGAGACTCCGAGAGGAGCTATCAAAGTTACTGCCCAACCAACAGACCACGTCGTGGGAGAGTACCTGGTACTCTCCCCGCAGACCGTACTACGTAGCCAGAAGCTCAGTCTGATTCACGCTTTGGCGGAGCAAGTGAAGACGTGCACGCACAACGGACGAGCAGGGAGGTATGCGGTCGAAGCGTACGACGGCCGAGTCCTAGTGCCCTCAGGCTATGCAATCTCGCCTGAAGACTTCCAGAGTCTAAGCGAAAGCGCAACGATGGTGTATAACGAAAGAGAGTTCGTAAACAGAAAGCTACACCATATTGCGATGCACGGACCAGCCCTGAACACCGACGAAGAGTCGTATGAGCTGGTGAGGGCAGAGAGGACAGAACACGAGTACGTCTACGACGTGGATCAGAGAAGATGCTGTAAGAAGGAAGAAGCCGCAGGACTGGTACTGGTGGGCGACTTGACTAATCCGCCCTACCACGAATTCGCATATGAAGGGCTAAAAATCCGCCCTGCCTGCCCATACAAAATTGCAGTCATAGGAGTCTTCGGAGTACCGGGATCTGGCAAGTCAGCTATTATCAAGAACCTAGTTACCAGGCAGGACCTGGTGACTAGCGGAAAGAAAGAAAACTGCCAAGAAATCACCACCGACGTGATGAGACAGAGAGGTCTAGAGATATCTGCACGTACGGTTGACTCGCTGCTCTTGAATGGATGCAACAGACCAGTCGACGTGTTGTACGTAGACGAGGCGTTTGCGTGCCACTCTGGAACGCTACTTGCTTTGATCGCCTTGGTGAGACCAAGGCAGAAAGTTGTACTTTGTGGTGACCCGAAGCAGTGCGGCTTCTTCAATATGATGCAGATGAAAGTCAACTATAATCACAACATCTGCACCCAAGTGTACCACAAAAGTATCTCCAGGCGGTGTACACTGCCTGTGACCGCCATTGTGTCATCGTTGCATTACGAAGGCAAAATGCGCACTACGAATGAGTACAACAAGCCGATTGTAGTGGACACTACAGGCTCAACAAAACCTGACCCTGGAGACCTCGTGTTAACGTGCTTCAGAGGGTGGGTTAAACAACTGCAAATTGACTATCGTGGATACGAGGTCATGACAGCAGCCGCATCCCAAGGGTTAACCAGAAAAGGAGTTTACGCAGTTAGACAAAAAGTTAATGAAAACCCGCTCTATGCATCAACGTCAGAGCACGTCAACGTACTCCTAACGCGTACGGAAGGTAAACTGGTATGGAAGACACTTTCCGGCGACCCGTGGATAAAGACGCTGCAGAACCCACCGAAAGGAAACTTCAAAGCAACTATTAAGGAGTGGGAGGTGGAGCATGCATCAATAATGGCGGGCATCTGCAGTCACCAAATGACCTTCGATACATTCCAAAATAAAGCCAACGTTTGTTGGGCTAAGAGCTTGGTCCCTATCCTCGAAACAGCGGGGATAAAACTAAATGATAGGCAGTGGTCTCAGATAATTCAAGCCTTCAAAGAAGACAAAGCATACTCACCTGAAGTAGCCCTGAATGAAATATGTACGCGCATGTATGGGGTGGATCTAGACAGCGGGCTATTTTCTAAACCGTTGGTGTCTGTGTATTACGCGGATAACCACTGGGATAATAGGCCTGGAGGGAAAATGTTCGGATTTAACCCCGAGGCAGCATCCATTCTAGAAAGAAAGTATCCATTCACAAAAGGGAAGTGGAACATCAACAAGCAGATCTGCGTGACTACCAGGAGGATAGAAGACTTTAACCCTACCACCAACATCATACCGGCCAACAGGAGACTACCACACTCATTAGTGGCCGAACACCGCCCAGTAAAAGGGGAAAGAATGGAATGGCTGGTTAACAAGATAAACGGCCACCACGTGCTCCTGGTCAGTGGCTATAACCTTGCACTGCCTACTAAGAGAGTCACTTGGGTAGCGCCGTTAGGTGTCCGCGGAGCGGACTACACATACAACCTAGAGTTGGGTCTGCCAGCAACGCTTGGTAGGTATGACCTAGTGGTCATAAACATCCACACACCTTTTCGCATACACCATTACCAACAGTGCGTCGACCACGCAATGAAACTGCAAATGCTCGGGGGTGACTCATTGAGACTGCTCAAACCGGGCGGCTCTCTATTGATCAGAGCATATGGTTACGCAGATAGAACCAGTGAACGAGTCATCTGCGTATTGGGACGCAAGTTTAGATCGTCTAGAGCGTTGAAACCACCATGTGTCACCAGCAACACTGAGATGTTTTTCCTATTCAGCAACTTTGACAATGGCAGAAGGAATTTCACAACTCATGTCATGAACAATCAACTGAATGCAGCCTTCGTAGGACAGGTCACCCGAGCAGGATGTGCACCGTCGTACCGGGTAAAACGCATGGACATCGCGAAGAACGATGAAGAGTGCGTAGTCAACGCCGCTAACCCTCGCGGGTTACCGGGTGACGGTGTTTGCAAGGCAGTATACAAAAAATGGCCGGAGTCCTTTAAGAACAGTGCAACACCAGTGGGAACCGCAAAAACAGTTATGTGCGGTACGTATCCAGTAATCCACGCTGTTGGACCAAACTTCTCTAATTATTCGGAGTCTGAAGGGGACCGGGAATTGGCAGCTGCCTATCGAGAAGTCGCAAAGGAAGTAACTAGGCTGGGAGTAAATAGTGTAGCTATACCTCTCCTCTCCACAGGTGTATACTCAGGAGGGAAAGACAGGCTGACCCAGTCACTGAACCACCTCTTTACAGCCATGGACTCGACGGATGCAGACGTGGTCATCTACTGCCGCGACAAAGAATGGGAGAAGAAAATATCTGAGGCCATACAGATGCGGACCCAAGTAGAGCTGCTGGATGAGCACATCTCCATAGACTGCGATATTGTTCGCGTGCACCCTGACAGCAGCTTGGCAGGCAGAAAAGGATACAGCACCACGGAAGGCGCACTGTACTCATATCTAGAAGGGACCCGTTTTCATCAGACGGCTGTGGATATGGCGGAGATACATACTATGTGGCCAAAGCAAACAGAGGCCAATGAGCAAGTCTGCCTATATGCCCTGGGGGAAAGTATTGAATCGATCAGGCAGAAATGCCCGGTGGATGATGCAGACGCATCATCTCCCCCCAAAACTGTCCCGTGCCTTTGCCGTTACGCTATGACTCCAGAACGCGTCACCCGGCTTCGCATGAACCACGTCACAAGCATAATTGTGTGTTCTTCGTTTCCCCTCCCAAAGTACAAAATAGAAGGAGTGCAAAAAGTCAAATGCTCTAAGGTAATGCTATTTGACCACAACGTGCCATCGCGCGTAAGTCCAAGGGAATATAGATCTTCCCAGGAGTCTGCACAGGAGGCGAGTACAATCACGTCACTGACGCATAGTCAATTCGACCTAAGCGTTGATGGCGAGATACTGCCCGTCCCGTCAGACCTGGATGCTGACGCCCCAGCCCTAGAACCAGCACTAGACGACGGGGCGACACACACGCTGCCATCCACAACCGGAAACCTTGCGGCCGTGTCTGATTGGGTAATGAGCACCGTACCTGTCGCGCCGCCCAGAAGAAGGCGAGGGAGAAACCTGACTGTGACATGTGACGAGAGAGAAGGGAATATAACACCCATGGCTAGCGTCCGATTCTTTAGGGCAGAGCTGTGTCCGGTCGTACAAGAAACAGCGGAGACGCGTGACACAGCAATGTCTCTTCAGGCACCACCGAGTACCGCCACGGAACCGAATCATCCGCCGATCTCCTTCGGAGCATCAAGCGAGACGTTCCCCATTACATTTGGGGACTTCAACGAAGGAGAAATCGAAAGCTTGTCTTCTGAGCTACTAACTTTCGGAGACTTCTTACCAGGAGAAGTGGATGACTTGACAGACAGCGACTGGTCCACGTGCTCAGACACGGACGACGAGTTATGACTAGACAGGGCAGGTGGGTATATATTCTCGTCGGACACCGGTCCAGGTCATTTACAACAGAAGTCAGTACGCCAGTCAGTGCTGCCGGTGAACACCCTGGAGGAAGTCCACGAGGAGAAGTGTTACCCACCTAAGCTGGATGAAGCAAAGGAGCAACTATTACTTAAGAAACTCCAGGAGAGTGCATCCATGGCCAACAGAAGCAGGTATCAGTCGCGCAAAGTAGAAAACATGAAAGCAGCAATCATCCAGAGACTAAAGAGAGGCTGTAGACTATACTTAATGTCAGAGACCCCAAAAGTCCCTACTTACCGGACTACATATCCGGCGCCTGTGTACTCGCCTCCGATCAACGTCCGATTGTCCAATCCCGAGTCCGCAGTGGCAGCATGCAATGAGTTCTTAGCTAGAAACTATCCAACTGTCTCATCATACCAAATTACCGACGAGTATGATGCATATCTAGACATGGTGGACGGGTCGGAGAGTTGCCTGGACCGAGCGACATTCAATCCGTCAAAACTCAGGAGCTACCCGAAACAGCACGCTTACCACGCGCCCTCCATCAGAAGCGCTGTACCGTCCCCATTCCAGAACACACTACAGAATGTACTGGCAGCAGCCACGAAAAGAAACTGCAACGTCACACAGATGAGGGAATTACCCACTTTGGACTCAGCAGTATTCAACGTGGAGTGTTTCAAAAAATTCGCATGCAACCAAGAATACTGGGAAGAATTTGCTGCCAGCCCTATTAGGATAACAACTGAGAATTTAGCAACCTATGTTACTAAACTAAAAGGGCCAAAAGCAGCAGCGCTATTCGCAAAAACCCATAATCTACTGCCACTACAGGAAGTACCAATGGATAGGTTCACAGTAGATATGAAAAGGGACGTAAAGGTGACTCCTGGTACAAAGCATACAGAGGAAAGACCTAAGGTGCAGGTTATACAGGCGGCTGAACCCTTGGCGACAGCATACCTATGTGGGATTCACAGAGAGCTGGTTAGGAGGCTGAACGCCGTCCTCCTACCCAATGTACATACACTATTTGACATGTCTGCCGAGGATTTCGATGCCATCATAGCCGCACACTTTAAGCCAGGAGACACTGTTTTGGAAACGGACATAGCCTCCTTTGATAAGAGCCAAGATGATTCACTTGCGCTTACTGCTTTGATGCTGTTAGAGGATTTAGGGGTGGATCACTCCCTGCTGGACTTGATAGAGGCTGCTTTCGGAGAGATTTCCAGCTGTCACCTACCGACAGGTACGCGCTTCAAGTTCGGCGCCATGATGAAATCAGGTATGTTCCTAACTCTGTTCGTCAACACATTGTTAAACATCACCATCGCCAGCCGAGTGCTGGAAGATCGTCTGACAAAATCCGCGTGCGCGGCCTTCATCGGCGACGACAACATAATACATGGAGTCGTCTCCGATGAATTGATGGCAGCCAGATGTGCCACTTGGATGAACATGGAAGTGAAGATCATAGATGCAGTTGTATCCTTGAAAGCCCCTTACTTTTGTGGAGGGTTTATACTGCACGATACTGTGACAGGAACAGCTTGCAGAGTGGCAGACCCGCTAAAAAGGCTTTTTAAACTGGGCAAACCGCTAGCGGCAGGTGACGAACAAGATGAAGATAGAAGACGAGCGCTGGCTGACGAAGTGATCAGATGGCAACGAACAGGGCTAATTGATGAGCTGGAGAAAGCGGTATACTCTAGGTACGAAGTGCAGGGTATATCAGTTGTGGTAATGTCCATGGCCACCTTTGCAAGCTCCAGATCCAACTTCGAGAAGCTCAGAGGACCCGTCATAACTTTGTACGGCGGTCCTAAATAGGTACGCACTACAGCTACCTATTTTGCAGAAGCCGACAGCAAGTATCTAAACACTAATCAGCTACAATGGAGTTCATCCCAACCCAAACTTTTTACAATAGGAGGTACCAGCCTCGACCCTGGACTCCGCGCCCTACTATCCAAGTCATCAGGCCCAGACCGCGCCCTCAGAGGCAAGCTGGGCAACTTGCCCAGCTGATCTCAGCAGTTAATAAACTGACAATGCGCGCGGTACCCCAACAGAAGCCACGCAGGAATCGGAAGAATAAGAAGCAAAAGCAAAAACAACAGGCGCCACAAAACAACACAAATCAAAAGAAGCAGCCACCTAAAAAGAAACCGGCTCAAAAGAAAAAGAAGCCGGGCCGCAGAGAGAGGATGTGCATGAAAATCGAAAATGATTGTATTTTCGAAGTCAAGCACGAAGGTAAGGTAACAGGTTACGCGTGCCTGGTGGGGGACAAAGTAATGAAACCAGCACACGTAAAGGGGACCATCGATAACGCGGACCTGGCCAAACTGGCCTTTAAGCGGTCATCTAAGTATGACCTTGAATGCGCGCAGATACCCGTGCACATGAAGTCCGACGCTTCGAAGTTCACCCATGAGAAACCGGAGGGGTACTACAACTGGCACCACGGAGCAGTACAGTACTCAGGAGGCCGGTTCACCATCCCTACAGGTGCTGGCAAACCAGGGGACAGCGGCAGACCGATCTTCGACAACAAGGGACGCGTGGTGGCCATAGTCTTAGGAGGAGCTAATGAAGGAGCCCGTACAGCCCTCTCGGTGGTGACCTGGAATAAAGACATTGTCACTAAAATCACCCCCGAGGGGGCCGAAGAGTGGAGTCTTGCCATCCCAGTTATGTGCCTGTTGGCAAACACCACGTTCCCCTGCTCCCAGCCCCCTTGCACGCCCTGCTGCTACGAAAAGGAACCGGAGGAAACCCTACGCATGCTTGAGGACAACGTCATGAGACCTGGGTACTATCAGCTGCTACAAGCATCCTTAACATGTTCTCCCCACCGCCAGCGACGCAGCACCAAGGACAACTTCAATGTCTATAAAGCCACAAGACCATACTTAGCTCACTGTCCCGACTGTGGAGAAGGGCACTCGTGCCATAGTCCCGTAGCACTAGAACGCATCAGAAATGAAGCGACAGACGGGACGCTGAAAATCCAGGTCTCCTTGCAAATCGGAATAAAGACGGATGACAGCCACGATTGGACCAAGCTGCGTTATATGGACAACCACATGCCAGCAGACGCAGAGAGGGCGGGGCTATTTGTAAGAACATCAGCACCGTGTACGATTACTGGAACAATGGGACACTTCATCCTGGCCCGATGTCCAAAAGGGGAAACTCTGACGGTGGGATTCACTGACAGTAGGAAGATTAGTCACTCATGTACGCACCCATTTCACCACGACCCTCCTGTGATAGGTCGGGAAAAATTCCATTCCCGACCGCAGCACGGTAAAGAGCTACCTTGCAGCACGTACGTGCAGAGCACCGCCGCAACTACCGAGGAGATAGAGGTACACATGCCCCCAGACACCCCTGATCGCACATTAATGTCACAACAGTCCGGCAACGTAAAGATCACAGTCAATGGCCAGACGGTGCGGTACAAGTGTAATTGCGGTGGCTCAAATGAAGGACTAACAACTACAGACAAAGTGATTAATAACTGCAAGGTTGATCAATGTCATGCCGCGGTCACCAATCACAAAAAGTGGCAGTATAACTCCCCTCTGGTCCCGCGTAATGCTGAACTTGGGGACCGAAAAGGAAAAATTCACATCCCGTTTCCGCTGGCAAATGTAACATGCAGGGTGCCTAAAGCAAGGAACCCCACCGTGACGTACGGGAAAAACCAAGTCATCATGCTACTGTATCCTGACCACCCAACACTCCTGTCCTACCGGAATATGGGAGAAGAACCAAACTATCAAGAAGAGTGGGTGATGCATAAGAAGGAAGTCGTGCTAACCGTGCCGACTGAAGGGCTCGAGGTCACGTGGGGCAACAACGAGCCGTATAAGTATTGGCCGCAGTTATCTACAAACGGTACAGCCCATGGCCACCCGCATGAGATAATTCTGTATTATTATGAGCTGTACCCCACTATGACTGTAGTAGTTGTGTCAGTGGCCACGTTCATACTCCTGTCGATGGTGGGTATGGCAGCGGGGATGTGCATGTGTGCACGACGCAGATGCATCACACCGTATGAACTGACACCAGGAGCTACCGTCCCTTTCCTGCTTAGCCTAATATGCTGCATCAGAACAGCTAAAGCGGCCACATACCAAGAGGCTGCGATATACCTGTGGAACGAGCAGCAACCTTTGTTTTGGCTACAAGCCCTTATTCCGCTGGCAGCCCTGATTGTTCTATGCAACTGTCTGAGACTCTTACCATGCTGCTGTAAAACGTTGGCTTTTTTAGCCGTAATGAGCGTCGGTGCCCACACTGTGAGCGCGTACGAACACGTAACAGTGATCCCGAACACGGTGGGAGTACCGTATAAGACTCTAGTCAATAGACCTGGCTACAGCCCCATGGTATTGGAGATGGAACTACTGTCAGTCACTTTGGAGCCAACACTATCGCTTGATTACATCACGTGCGAGTACAAAACCGTCATCCCGTCTCCGTACGTGAAGTGCTGCGGTACAGCAGAGTGCAAGGACAAAAACCTACCTGACTACAGCTGTAAGGTCTTCACCGGCGTCTACCCATTTATGTGGGGCGGCGCCTACTGCTTCTGCGACGCTGAAAACACGCAGTTGAGCGAAGCACACGTGGAGAAGTCCGAATCATGCAAAACAGAATTTGCATCAGCATACAGGGCTCATACCGCATCTGCATCAGCTAAGCTCCGCGTCCTTTACCAAGGAAATAACATCACTGTAACTGCCTATGCAAACGGCGACCATGCCGTCACAGTTAAGGACGCCAAATTCATTGTGGGGCCAATGTCTTCAGCCTGGACACCTTTCGACAACAAAATTGTGGTGTACAAAGGTGACGTCTATAACATGGACTACCCGCCCTTTGGCGCAGGAAGACCAGGACAATTTGGCGATATCCAAAGTCGCACACCTGAGAGTAAAGACGTCTATGCTAATACACAACTGGTACTGCAGAGACCGGCTGTGGGTACGGTACACGTGCCATACTCTCAGGCACCATCTGGCTTTAAGTATTGGCTAAAAGAACGCGGGGCGTCGCTGCAGCACACAGCACCATTTGGCTGCCAAATAGCAACAAACCCGGTAAGAGCGGTGAACTGCGCCGTAGGGAACATGCCCATCTCCATCGACATACCGGAAGCGGCCTTCACTAGGGTCGTCGACGCGCCCTCTTTAACGGACATGTCGTGCGAGGTACCAGCCTGCACCCATTCCTCAGACTTTGGGGGCGTCGCCATTATTAAATATGCAGCCAGCAAGAAAGGCAAGTGTGCGGTGCATTCGATGACTAACGCCGTCACTATTCGGGAAGCTGAGATAGAAGTTGAAGGGAATTCTCAGCTGCAAATCTCTTTCTCGACGGCCTTAGCCAGCGCCGAATTCCGCGTACAAGTCTGTTCTACACAAGTACACTGTGCAGCCGAGTGCCACCCCCCGAAGGACCACATAGTCAACTACCCGGCGTCACATACCACCCTCGGGGTCCAGGACATCTCCGCTACGGCGATGTCATGGGTGCAGAAGATCACGGGAGGTGTGGGACTGGTTGTTGCTGTTGCCGCACTGATTCTAATCGTGGTGCTATGCGTGTCGTTCAGCAGGCACTAACTTGACAATTAAGTATGAAGGTATATGTGTCCCCTAAGAGACACACTGTACATAGCAAATAATCTATAGATCAAAGGGCTACGCAACCCCTGAATAGTAACAAAATACAAAATCACTAAAAATTATAAAAACAGAAAAATACATAAATAGGTATACGTGTCCCCTAAGAGACACATTGTATGTAGGTGATAAGTATAGATCAAAGGGCCGAATAACCCCTGAATAGTAACAAAATATGAAAATCAATAAAAATCATAAAATAGAAAAACCATAAACAGAAGTAGTTCAAAGGGCTAT

>FR717337|2005|France: La Reunion, Group 4

CAAAGCAAGAGATTAATAACCCATCATGGATCCTGTGTACGTGGACATAGACGCTGACAGCGCCTTTTTGAAGGCCCTGCAACGTGCGTACCCCATGTTTGAGGTGGAACCAAGGCAGGTCACACCGAATGACCATGCTAATGCTAGAGCGTTCTCGCATCTAGCTATAAAACTAATAGAGCAGGAAATTGACCCCGACTCAACCATCCTGGATATCGGCAGTGCGCCAGCAAGGAGGATGATGTCGGACAGGAAGTACCACTGCGTCTGCCCGATGCGCAGTGCGGAAGATCCCGAGAGACTCGCCAATTATGCGAGAAAGCTAGCATCTGCCGCAGGAAAAGTCCTGGACAGAAACATCTCTGGAAAGATCGGGGACTTACAAGCAGTAATGGCCGTGCCAGACACGGAGACGCCAACATTCTGCTTACACACAGACGTCTCATGTAGACAGAGAGCAGACGTCGCTATATACCAAGACGTCTATGCTGTACACGCACCCACGTCGCTATACCACCAGGCGATTAAAGGGGTCCGAGTGGCGTACTGGGTTGGGTTCGACACAACCCCGTTCATGTACAATGCCATGGCGGGTGCCTACCCCTCATACTCGACAAACTGGGCAGATGAGCAGGTACTGAAGGCTAAGAACATAGGATTATGTTCAACAGACCTGACGGAAGGTAGACGAGGCAAGTTGTCTATTATGAGAGGGAAAAAGCTAAAACCGTGCGACCGTGTGCTGTTCTCAGTAGGGTCAACGCTCTACCCGGAAAGCCGCAAGCTACTTAAGAGCTGGCACCTGCCATCGGTGTTCCATTTAAAGGGCAAACTCAGCTTCACATGCCGCTGTGATACAGTGGTTTCGTGTGAGGGCTACGTCGTTAAGAGAATAACGATGAGCCCAGGCCTTTATGGAAAAACCACAGGGTATGCGGTAACCCACCACGCAGACGGATTCCTGATGTGCAAGACTACCGACACGGTTGACGGCGAAAGAGTGTCATTCTCGGTGTGCACATACGTGCCGGCGACCATTTGTGATCAAATGACCGGCATCCTTGCTACAGAAGTCACGCCGGAGGATGCACAGAAGCTGTTGGTGGGGCTGAACCAGAGAATAGTGGTTAACGGCAGAACGCAACGGAATACGAACACCATGAAAAATTATCTGCTTCCCGTGGTCGCCCAAGCCTTCAGTAAGTGGGCAAAGGAGTGCCGGAAAGACATGGAAGATGAAAAACTCCTGGGGGTCAGAGAAAGAACACTGACCTGCTGCTGTCTATGGGCATTCAAGAAGCAGAAAACACACACGGTCTACAAGAGGCCTGATACCCAGTCAATTCAGAAGGTTCAGGCCGAGTTTGACAGCTTTGTGGTACCGAGTCTGTGGTCGTCCGGGTTGTCAATCCCTTTGAGGACTAGAATCAAATGGTTGTTAAGCAAGGTGCCAAAAACCGACCTGATCCCATACAGCGGAGACGCCCGAGAAGCCCGGGACGCAGAAAAAGAAGCAGAGGAAGAACGAGAAGCAGAACTGACTCGCGAAGCCCTACCACCTCTACAGGCAGCACAGGAAGATGTTCAGGTCGAAATCGACGTGGAACAGCTTGAGGACAGAGCGGGCGCAGGAATAATAGAGACTCCGAGAGGAGCTATCAAAGTTACTGCCCAACCAACAGACCACGTCGTGGGAGAGTACCTGGTACTCTCCCCGCAGACCGTACTACGTAGCCAGAAGCTCAGTCTGATTCACGCTTTGGCGGAGCAAGTGAAGACGTGCACGCACAACGGACGAGCAGGGAGGTATGCGGTCGAAGCGTACGACGGCCGAGTCCTAGTGCCCTCAGGCTATGCAATCTCGCCTGAAGACTTCCAGAGTCTAAGCGAAAGCGCAACGATGGTGTATAACGAAAGAGAGTTCGTAAACAGAAAGCTACACCATATTGCGATGCACGGACCAGCCCTGAACACCGACGAAGAGTCGTATGAGCTGGTGAGGGCAGAGAGGACAGAACACGAGTACGTCTACGACGTGGATCAGAGAAGATGCTGTAAGAAGGAAGAAGCCGCAGGACTGGTACTGGTGGGCGACTTGACTAATCCGCCCTACCACGAATTCGCATATGAAGGGCTAAAAATCCGCCCTGCCTGCCCATACAAAATTGCAGTCATAGGAGTCTTCGGAGTACCGGGATCTGGCAAGTCAGCTATTATCAAGAACCTAGTTACCAGGCAGGACCTGGTGACTAGCGGAAAGAAAGAAAACTGCCAAGAAATCACCACCGACGTGATGAGACAGAGAGGTCTAGAGATATCTGCACGTACGGTTGACTCGCTGCTCTTGAATGGATGCAACAGACCAGTCGACGTGTTGTACGTAGACGAGGCGTTTGCGTGCCACTCTGGAACGCTACTTGCTTTGATCGCCTTGGTGAGACCAAGGCAGAAAGTTGTACTTTGTGGTGACCCGAAGCAGTGCGGCTTCTTCAATATGATGCAGATGAAAGTCAACTATAATCACAACATCTGCACCCAAGTGTACCACAAAAGTATCTCCAGGCGGTGTACACTGCCTGTGACCGCCATTGTGTCATCGTTGCATTACGAAGGCAAAATGCGCACTACGAATGAGTACAACAAGCCGATTGTAGTGGACACTACAGGCTCAACAAAACCTGACCCTGGAGACCTCGTGTTAACGTGCTTCAGAGGGTGGGTTAAACAACTGCAAATTGACTATCGTGGATACGAGGTCATGACAGCAGCCGCATCCCAAGGGTTAACCAGAAAAGGAGTTTACGCAGTTAGACAAAAAGTTAATGAAAACCCGCTCTATGCATCAACGTCAGAGCACGTCAACGTACTCCTAACGCGTACGGAAGGTAAACTGGTATGGAAGACACTTTCCGGCGACCCGTGGATAAAGACGCTGCAGAACCCACCGAAAGGAAACTTCAAAGCAACTATTAAGGAGTGGGAGGTGGAGCATGCATCAATAATGGCGGGCATCTGCAGTCACCAAATGACCTTCGATACATTCCAAAATAAAGCCAACGTTTGTTGGGCTAAGAGCTTGGTCCCTATCCTCGAAACAGCGGGGATAAAACTAAATGATAGGCAGTGGTCTCAGATAATTCAAGCCTTCAAAGAAGACAAAGCATACTCACCTGAAGTAGCCCTGAATGAAATATGTACGCGCATGTATGGGGTGGATCTAGACAGCGGGCTATTTTCTAAACCGTTGGTGTCTGTGTATTACGCGGATAACCACTGGGATAATAGGCCTGGAGGGAAAATGTTCGGATTTAACCCCGAGGCAGCATCCATTCTAGAAAGAAAGTATCCATTCACAAAAGGGAAGTGGAACATCAACAAGCAGATCTGCGTGACTACCAGGAGGATAGAAGACTTTAACCCTACCACCAACATCATACCGGCCAACAGGAGACTACCACACTCATTAGTGGCCGAACACCGCCCAGTAAAAGGGGAAAGAATGGAATGGCTGGTTAACAAGATAAACGGCCACCACGTGCTCCTGGTCAGTGGCTATAACCTTGCACTGCCTACTAAGAGAGTCACTTGGGTAGCGCCGTTAGGTGTCCGCGGAGCGGACTACACATACAACCTAGAGTTGGGTCTGCCAGCAACGCTTGGTAGGTATGACCTAGTGGTCATAAACATCCACACACCTTTTCGCATACACCATTACCAACAGTGCGTCGACCACGCAATGAAACTGCAAATGCTCGGGGGTGACTCATTGAGACTGCTCAAACCGGGCGGCTCTCTATTGATCAGAGCATATGGTTACGCAGATAGAACCAGTGAACGAGTCATCTGCGTATTGGGACGCAAGTTTAGATCGTCTAGAGCGTTGAAACCACCATGTGTCACCAGCAACACTGAGATGTTTTTCCTATTCAGCAACTTTGACAATGGCAGAAGGAATTTCACAACTCATGTCATGAACAATCAACTGAATGCAGCCTTCGTAGGACAGGTCACCCGAGCAGGATGTGCACCGTCGTACCGGGTAAAACGCATGGACATCGCGAAGAACGATGAAGAGTGCGTAGTCAACGCCGCTAACCCTCGCGGGTTACCGGGTGACGGTGTTTGCAAGGCAGTATACAAAAAATGGCCGGAGTCCTTTAAGAACAGTGCAACACCAGTGGGAACCGCAAAAACAGTTATGTGCGGTACGTATCCAGTAATCCACGCTGTTGGACCAAACTTCTCTAATTATTCGGAGTCTGAAGGGGACCGGGAATTGGCAGCTGCCTATCGAGAAGTCGCAAAGGAAGTAACTAGGCTGGGAGTAAATAGTGTAGCTATACCTCTCCTCTCCACAGGTGTATACTCAGGAGGGAAAGACAGGCTGACCCAGTCACTGAACCACCTCTTTACAGCCATGGACTCGACGGATGCAGACGTGGTCATCTACTGCCGCGACAAAGAATGGGAGAAGAAAATATCTGAGGCCATACAGATGCGGACCCAAGTAGAGCTGCTGGATGAGCACATCTCCATAGACTGCGATATTGTTCGCGTGCACCCTGACAGCAGCTTGGCAGGCAGAAAAGGATACAGCACCACGGAAGGCGCACTGTACTCATATCTAGAAGGGACCCGTTTTCATCAGACGGCTGTGGATATGGCGGAGATACATACTATGTGGCCAAAGCAAACAGAGGCCAATGAGCAAGTCTGCCTATATGCCCTGGGGGAAAGTATTGAATCGATCAGGCAGAAATGCCCGGTGGATGATGCAGACGCATCATCTCCCCCCAAAACTGTCCCGTGCCTTTGCCGTTACGCTATGACTCCAGAACGCGTCACCCGGCTTCGCATGAACCACGTCACAAGCATAATTGTGTGTTCTTCGTTTCCCCTCCCAAAGTACAAAATAGAAGGAGTGCAAAAAGTCAAATGCTCTAAGGTAATGCTATTTGACCACAACGTGCCATCGCGCGTAAGTCCAAGGGAATATAGATCTTCCCAGGAGTCTGCACAGGAGGCGAGTACAATCACGTCACTGACGCATAGTCAATTCGACCTAAGCGTTGATGGCGAGATACTGCCCGTCCCGTCAGACCTGGATGCTGACGCCCCAGCCCTAGAACCAGCACTAGACGACGGGGCGACACACACGCTGCCATCCACAACCGGAAACCTTGCGGCCGTGTCTGATTGGGTAATGAGCACCGTACCTGTCGCGCCGCCCAGAAGAAGGCGAGGGAGAAACCTGACTGTGACATGTGACGAGAGAGAAGGGAATATAACACCCATGGCTAGCGTCCGATTCTTTAGGGCAGAGCTGTGTCCGGTCGTACAAGAAACAGCGGAGACGCGTGACACAGCAATGTCTCTTCAGGCACCACCGAGTACCGCCACGGAACCGAATCATCCGCCGATCTCCTTCGGAGCATCAAGCGAGACGTTCCCCATTACATTTGGGGACTTCAACGAAGGAGAAATCGAAAGCTTGTCTTCTGAGCTACTAACTTTCGGAGACTTCTTACCAGGAGAAGTGGATGACTTGACAGACAGCGACTGGTCCACGTGCTCAGACACGGACGACGAGTTATGACTAGACAGGGCAGGTGGGTATATATTCTCGTCGGACACCGGTCCAGGTCATTTACAACAGAAGTCAGTACGCCAGTCAGTGCTGCCGGTGAACACCCTGGAGGAAGTCCACGAGGAGAAGTGTTACCCACCTAAGCTGGATGAAGCAAAGGAGCAACTATTACTTAAGAAACTCCAGGAGAGTGCATCCATGGCCAACAGAAGCAGGTATCAGTCGCGCAAAGTAGAAAACATGAAAGCAGCAATCATCCAGAGACTAAAGAGAGGCTGTAGACTATACTTAATGTCAGAGACCCCAAAAGTCCCTACTTACCGGACTACATATCCGGCGCCTGTGTACTCGCCTCCGATCAACGTCCGATTGTCCAATCCCGAGTCCGCAGTGGCAGCATGCAATGAGTTCTTAGCTAGAAACTATCCAACTGTCTCATCATACCAAATTACCGACGAGTATGATGCATATCTAGACATGGTGGACGGGTCGGAGAGTTGCCTGGACCGAGCGACATTCAATCCGTCAAAACTCAGGAGCTACCCGAAACAGCACGCTTACCACGCGCCCTCCATCAGAAGCGCTGTACCGTCCCCATTCCAGAACACACTACAGAATGTACTGGCAGCAGCCACGAAAAGAAACTGCAACGTCACACAGATGAGGGAATTACCCACTTTGGACTCAGCAGTATTCAACGTGGAGTGTTTCAAAAAATTCGCATGCAACCAAGAATACTGGGAAGAATTTGCTGCCAGCCCTATTAGGATAACAACTGAGAATTTAGCAACCTATGTTACTAAACTAAAAGGGCCAAAAGCAGCAGCGCTATTCGCAAAAACCCATAATCTACTGCCACTACAGGAAGTACCAATGGATAGGTTCACAGTAGATATGAAAAGGGACGTAAAGGTGACTCCTGGTACAAAGCATACAGAGGAAAGACCTAAGGTGCAGGTTATACAGGCGGCTGAACCCTTGGCGACAGCATACCTATGTGGGATTCACAGAGAGCTGGTTAGGAGGCTGAACGCCGTCCTCCTACCCAATGTACATACACTATTTGACATGTCTGCCGAGGATTTCGATGCCATCATAGCCGCACACTTTAAGCCAGGAGACACTGTTTTGGAAACGGACATAGCCTCCTTTGATAAGAGCCAAGATGATTCACTTGCGCTTACTGCTTTGATGCTGTTAGAGGATTTAGGGGTGGATCACTCCCTGCTGGACTTGATAGAGGCTGCTTTCGGAGAGATTTCCAGCTGTCACCTACCGACAGGTACGCGCTTCAAGTTCGGCGCCATGATGAAATCAGGTATGTTCCTAACTCTGTTCGTCAACACATTGTTAAACATCACCATCGCCAGCCGAGTGCTGGAAGATCGTCTGACAAAATCCGCGTGCGCGGCCTTCATCGGCGACGACAACATAATACATGGAGTCGTCTCCGATGAATTGATGGCAGCCAGATGTGCCACTTGGATGAACATGGAAGTGAAGATCATAGATGCAGTTGTATCCTTGAAAGCCCCTTACTTTTGTGGAGGGTTTATACTGCACGATACTGTGACAGGAACAGCTTGCAGAGTGGCAGACCCGCTAAAAAGGCTTTTTAAACTGGGCAAACCGCTAGCGGCAGGTGACGAACAAGATGAAGATAGAAGACGAGCGCTGGCTGACGAAGTGATCAGATGGCAACGAACAGGGCTAATTGATGAGCTGGAGAAAGCGGTATACTCTAGGTACGAAGTGCAGGGTATATCAGTTGTGGTAATGTCCATGGCCACCTTTGCAAGCTCCAGATCCAACTTCGAGAAGCTCAGAGGACCCGTCATAACTTTGTACGGCGGTCCTAAATAGGTACGCACTACAGCTACCTATTTTGCAGAAGCCGACAGCAAGTATCTAAACACTAATCAGCTACAATGGAGTTCATCCCAACCCAAACTTTTTACAATAGGAGGTACCAGCCTCGACCCTGGACTCCGCGCCCTACTATCCAAGTCATCAGGCCCAGACCGCGCCCTCAGAGGCAAGCTGGGCAACTTGCCCAGCTGATCTCAGCAGTTAATAAACTGACAATGCGCGCGGTACCCCAACAGAAGCCACGCAGGAATCGGAAGAATAAGAAGCAAAAGCAAAAACAACAGGCGCCACAAAACAACACAAATCAAAAGAAGCAGCCACCTAAAAAGAAACCGGCTCAAAAGAAAAAGAAGCCGGGCCGCAGAGAGAGGATGTGCATGAAAATCGAAAATGATTGTATTTTCGAAGTCAAGCACGAAGGTAAGGTAACAGGTTACGCGTGCCTGGTGGGGGACAAAGTAATGAAACCAGCACACGTAAAGGGGACCATCGATAACGCGGACCTGGCCAAACTGGCCTTTAAGCGGTCATCTAAGTATGACCTTGAATGCGCGCAGATACCCGTGCACATGAAGTCCGACGCTTCGAAGTTCACCCATGAGAAACCGGAGGGGTACTACAACTGGCACCACGGAGCAGTACAGTACTCAGGAGGCCGGTTCACCATCCCTACAGGTGCTGGCAAACCAGGGGACAGCGGCAGACCGATCTTCGACAACAAGGGACGCGTGGTGGCCATAGTCTTAGGAGGAGCTAATGAAGGAGCCCGTACAGCCCTCTCGGTGGTGACCTGGAATAAAGACATTGTCACTAAAATCACCCCCGAGGGGGCCGAAGAGTGGAGTCTTGCCATCCCAGTTATGTGCCTGTTGGCAAACACCACGTTCCCCTGCTCCCAGCCCCCTTGCACGCCCTGCTGCTACGAAAAGGAACCGGAGGAAACCCTACGCATGCTTGAGGACAACGTCATGAGACCTGGGTACTATCAGCTGCTACAAGCATCCTTAACATGTTCTCCCCACCGCCAGCGACGCAGCACCAAGGACAACTTCAATGTCTATAAAGCCACAAGACCATACTTAGCTCACTGTCCCGACTGTGGAGAAGGGCACTCGTGCCATAGTCCCGTAGCACTAGAACGCATCAGAAATGAAGCGACAGACGGGACGCTGAAAATCCAGGTCTCCTTGCAAATCGGAATAAAGACGGATGACAGCCACGATTGGACCAAGCTGCGTTATATGGACAACCACATGCCAGCAGACGCAGAGAGGGCGGGGCTATTTGTAAGAACATCAGCACCGTGTACGATTACTGGAACAATGGGACACTTCATCCTGGCCCGATGTCCAAAAGGGGAAACTCTGACGGTGGGATTCACTGACAGTAGGAAGATTAGTCACTCATGTACGCACCCATTTCACCACGACCCTCCTGTGATAGGTCGGGAAAAATTCCATTCCCGACCGCAGCACGGTAAAGAGCTACCTTGCAGCACGTACGTGCAGAGCACCGCCGCAACTACCGAGGAGATAGAGGTACACATGCCCCCAGACACCCCTGATCGCACATTAATGTCACAACAGTCCGGCAACGTAAAGATCACAGTCAATGGCCAGACGGTGCGGTACAAGTGTAATTGCGGTGGCTCAAATGAAGGACTAACAACTACAGACAAAGTGATTAATAACTGCAAGGTTGATCAATGTCATGCCGCGGTCACCAATCACAAAAAGTGGCAGTATAACTCCCCTCTGGTCCCGCGTAATGCTGAACTTGGGGACCGAAAAGGAAAAATTCACATCCCGTTTCCGCTGGCAAATGTAACATGCAGGGTGCCTAAAGCAAGGAACCCCACCGTGACGTACGGGAAAAACCAAGTCATCATGCTACTGTATCCTGACCACCCAACACTCCTGTCCTACCGGAATATGGGAGAAGAACCAAACTATCAAGAAGAGTGGGTGATGCATAAGAAGGAAGTCGTGCTAACCGTGCCGACTGAAGGGCTCGAGGTCACGTGGGGCAACAACGAGCCGTATAAGTATTGGCCGCAGTTATCTACAAACGGTACAGCCCATGGCCACCCGCATGAGATAATTCTGTATTATTATGAGCTGTACCCCACTATGACTGTAGTAGTTGTGTCAGTGGCCACGTTCATACTCCTGTCGATGGTGGGTATGGCAGCGGGGATGTGCATGTGTGCACGACGCAGATGCATCACACCGTATGAACTGACACCAGGAGCTACCGTCCCTTTCCTGCTTAGCCTAATATGCTGCATCAGAACAGCTAAAGCGGCCACATACCAAGAGGCTGCGATATACCTGTGGAACGAGCAGCAACCTTTGTTTTGGCTACAAGCCCTTATTCCGCTGGCAGCCCTGATTGTTCTATGCAACTGTCTGAGACTCTTACCATGCTGCTGTAAAACGTTGGCTTTTTTAGCCGTAATGAGCGTCGGTGCCCACACTGTGAGCGCGTACGAACACGTAACAGTGATCCCGAACACGGTGGGAGTACCGTATAAGACTCTAGTCAATAGACCTGGCTACAGCCCCATGGTATTGGAGATGGAACTACTGTCAGTCACTTTGGAGCCAACACTATCGCTTGATTACATCACGTGCGAGTACAAAACCGTCATCCCGTCTCCGTACGTGAAGTGCTGCGGTACAGCAGAGTGCAAGGACAAAAACCTACCTGACTACAGCTGTAAGGTCTTCACCGGCGTCTACCCATTTATGTGGGGCGGCGCCTACTGCTTCTGCGACGCTGAAAACACGCAGTTGAGCGAAGCACACGTGGAGAAGTCCGAATCATGCAAAACAGAATTTGCATCAGCATACAGGGCTCATACCGCATCTGCATCAGCTAAGCTCCGCGTCCTTTACCAAGGAAATAACATCACTGTAACTGCCTATGCAAACGGCGACCATGCCGTCACAGTTAAGGACGCCAAATTCATTGTGGGGCCAATGTCTTCAGCCTGGACACCTTTCGACAACAAAATTGTGGTGTACAAAGGTGACGTCTATAACATGGACTACCCGCCCTTTGGCGCAGGAAGACCAGGACAATTTGGCGATATCCAAAGTCGCACACCTGAGAGTAAAGACGTCTATGCTAATACACAACTGGTACTGCAGAGACCGGCTGTGGGTACGGTACACGTGCCATACTCTCAGGCACCATCTGGCTTTAAGTATTGGCTAAAAGAACGCGGGGCGTCGCTGCAGCACACAGCACCATTTGGCTGCCAAATAGCAACAAACCCGGTAAGAGCGGTGAACTGCGCCGTAGGGAACATGCCCATCTCCATCGACATACCGGAAGCGGCCTTCACTAGGGTCGTCGACGCGCCCTCTTTAACGGACATGTCGTGCGAGGTACCAGCCTGCACCCATTCCTCAGACTTTGGGGGCGTCGCCATTATTAAATATGCAGCCAGCAAGAAAGGCAAGTGTGCGGTGCATTCGATGACTAACGCCGTCACTATTCGGGAAGCTGAGATAGAAGTTGAAGGGAATTCTCAGCTGCAAATCTCTTTCTCGACGGCCTTAGCCAGCGCCGAATTCCGCGTACAAGTCTGTTCTACACAAGTACACTGTGCAGCCGAGTGCCACCCCCCGAAGGACCACATAGTCAACTACCCGGCGTCACATACCACCCTCGGGGTCCAGGACATCTCCGCTACGGCGATGTCATGGGTGCAGAAGATCACGGGAGGTGTGGGACTGGTTGTTGCTGTTGCCGCACTGATTCTAATCGTGGTGCTATGCGTGTCGTTCAGCAGGCACTAACTTGACAATTAAGTATGAAGGTATATGTGTCCCCTAAGAGACACACTGTACATAGCAAATAATCTATAGATCAAAGGGCTACGCAACCCCTGAATAGTAACAAAATACAAAATCACTAAAAATTATAAAAACAGAAAAATACATAAATAGGTATACGTGTCCCCTAAGAGACACATTGTATGTAGGTGATAAGTATAGATCAAAGGGCCGAATAACCCCTGAATAGTAACAAAATATGAAAATCAATAAAAATCATAAAATAGAAAAACCATAAACAGAAGTAGTTCAAAGGGCTAT

>EF210157|2006|India, Group 4

ATGGCTGCGTGAGACACACGTAGCCTACCAGTTTCTTACTGCTCTACTCTGCAAAGCAAGAGATTAATAACCCATCATGGATCCTGTGTACGTGGACATAGACGCTGACAGCGCCTTTTTGAAGGCCCTGCAACGTGCGTACCCCATGTTTGAGGTGGAACCAAGGCAGGTCACACCGAATGACCATGCTAATGCTAGAGCGTTCTCGCATCTAGCTATAAAACTAATAGAGCAGGAAATTGACCCCGACTCAACCATCCTGGATATCGGCAGTGCGCCAGCAAGGAGGATGATGTCGGACAGGAAGTACCACTGCGTCTGCCCGATGCGCAGTGCGGAAGATCCCGAGAGACTCGCTAATTATGCGAGAAAGCTAGCATCTGCCGCAGGAAAAGTCCTGGACAGAAACATCTCTGGAAAGATCGGGGACTTACAAGCAGTAATGGCCGTGCCAGACAAGGAGACGCCAACATTCTGCTTACACACAGACGTCTCATGTAGACAGAGAGCAGACGTCGCTATATACCAAGACGTCTATGCTGTACACGCACCCACGTCGCTATACCACCAGGCGATTAAAGGGGTCCGAGTGGCGTACTGGGTTGGGTTCGACACAACCCCGTTCATGTACAATGCCATGGCGGGTGCCTACCCCTCATACTCGACAAACTGGGCAGATGAGCAGGTACTGAAGGCTAAGAACATAGGATTATGTTCAACAGACCTGACGGAAGGTAGACGAGGCAAGTTGTCTATTATGAGAGGGAAAAAGCTAAAACCGTGCGACCGTGTGCTGTTCTCAGTAGGGTCAACGCTCTACCCGGAAAGCCGCAAGCTACTTAAGAGCTGGCACCTGCCATCGGTGTTCCATTTAAAGGGCAAACTCAGCTTCACATGCCGCTGTGATACAGTGGTTTCGTGTGAGGGCTACGTCGTTAAGAGAATAACGATGAGCCCAGGCCTTTATGGAAAAACCACAGGGTATGCGGTAACCCACCACGCAGACGGATTCCTGCTGTGCAAGACTACCGACACGGTTGACGGCGAAAGAGTGTCATTCTCGGTGTGCACATACGTGCCGGCGACCATTTGTGATCAAATGACCGGCATCCTTGCTACAGAAGTCACGCCGGAGGATGCACAGAAGCTGTTGGTGGGGCTGAACCAGAGAATAGTGGTTAACGGCAGAACGCAACGGAATATGAACACCATGAAAAATTATCTGCTTCCCGTGGTCGCCCAAGCCTTCAGTAAGTGGGCAAAGGAGTGCCGGAAAGACATGGAAGATGAAAAACTCCTGGGGGTCAGAGAAAGAACACTGACCTGCTGCTGTCTATGGGCATTCAAGAAGCAGAAAACACACACGGTCTACAAGAGGCCTGATACCCAGTCAATTCAGAAGGTTCAGGCCGAGTTTGACAGCTTTGTGGTACCGAGTCTGTGGTCGTCCGGGTTGTCAATCCCTTTGAGGACTAGAATCAAATGGTTGTTAAGCAAGGTGCCAAAAACCGACCTGATCCCATACAGCGGAGACGCCCGAGAAGCCCGGGACGCAGAAAAAGAAGCAGAGGAAGAACGAGAAGCAGAACTGACTCGCGAAGCCCTACCACCTCTACAGGCAGCACAGGAAGATGTTCAGGTCGAAATCGACGTGGAACAGCTTGAGGACAGAGCGGGCGCAGGAATAATAGAGACTCCGAGAGGAGCTATCAAAGTTACTGCCCAACCAACAGACCACGTCGTGGGAGAGTACCTGGTACTCTCCCCGCAGACCGTACTACGTAGCCAGAAGCTCAGTCTGATTCACGCTTTGGCGGAGCAAGTGAAGACGTGCACGCACAACGGACGAGCAGGGAGGTATGCGGTCGAAGCGTACGACGGCCGAGTCCTAGTGCCCTCAGGCTATGCAATCTCGCCTGAAGACTTCCAGAGTCTAAGCGAAAGCGCGACGATGGTGTATAACGAAAGAGAGTTCGTAAACAGAAAGCTACACCATATTGCGATGCACGGACCAGCCCTGAACACCGACGAAGAGTCGTATGAGCTGGTGAGGGCAGAGAGGACAGAACACGAGTACGTCTACGACGTGGATCAGAGAAGATGCTGTAAGAAGGAAGAAGCCGCAGGACTGGTACTGGTGGGCGACTTGACTAATCCGCCCTACCACGAATTCGCATATGAAGGGCTAAAAATCCGCCCTGCCTGCCCATACAAAATTGCAGTCATAGGAGTCTTCGGAGTACCGGGATCTGGCAAGTCAGCTATTATCAAGAACCTAGTTACCAGGCAGGACCTGGTGACTAGCGGAAAGAAAGAAAACTGCCAAGAAATCACCACCGACGTGATGAGACAGAGAGGTCTAGAGATATCTGCACGTACGGTTGACTCGCTGCTCTTGAATGGATGCAACAGACCAGTCGACGTGTTGTACGTAGACGAGGCGTTTGCGTGCCACTCTGGAACGCTACTTGCTTTGATCGCCTTGGTGAGACCAAGGCAGAAAGTTGTACTTTGTGGTGACCCGAAGCAGTGCGGCTTCTTCAATATGATGCAGATGAAAGTCAACTATAATCACAACATCTGCACCCAAGTGTACCACAAAAGTATCTCCAGGCGGTGTACACTGCCTGTGACCGCCATTGTGTCATCGTTGCATTACGAAGGCAAAATGCGCACTACGAATGAGTACAACAAGCCGATCGTAGTGGACACTACAGGCTCAACAAAACCTGACCCTGGAGACCTCGTGTTAACGTGCTTCAGAGGGTGGGTTAAACAACTGCAAATTGACTATCGTGGATACGAGGTCATGACAGCAGCCGCATCCCAAGGGTTAACCAGAAAAGGAGTTTACGCAGTTAGACAAAAAGTTAATGAAAACCCGCTCTATGCATCAACGTCAGAGCACGTCAACGTACTCCTAACGCGTACGGAAGGTAAACTGGTATGGAAGACACTTTCCGGCGACCCGTGGATAAAGACGCTGCAGAACCCACCGAAAGGAAACTTCAAAGCAACTATTAAGGAGTGGGAGGTGGAGCATGCATCAATAATGGCGGGCATCTGCAGTCACCAAATGACCTTCGATACATTCCAAAATAAAGCCAACGTTTGTTGGGCTAAGAGCTTGGTCCCTATCCTCGAAACAGCGGGGATAAAACTAAATGATAGGCAGTGGTCTCAGATAATTCAAGCCTTCAAAGAAGACAAAGCATACTCACCTGAAGTAGCCCTGAATGAAATATGTACGCGCATGTATGGGGTGGATCTAGACAGCGGGCTATTTTCTAAACCGTTGGTGTCTGTGTATTACGCGGATAACCACTGGGATAATAGGCCTGGAGGGAAAATGTTCGGATTTAACCCCGAGGCAGCATCCATTCTAGAAAGAAAGTATCCATTCACAAAAGGGAAGTGGAACATCAACAAGCAGATCTGCGTGACTACCAGGAGGATAGAAGACTTTAACCCTACCACCAACATCATACCGGCCAACAGGAGACTACCACACTCATTAGTGGCCGAACACCGCCCAGTAAAAGGGGAAAGAATGGAATGGCTGGTTAACAAGATAAACGGCCACCACGTGCTCCTGGTCAGTGGCTATAACCTTGCACTGCCTACTAAGAGAGTCACTTGGGTAGCGCCGTTAGGTGTCCGCGGAGCGGACTACACATACAACCTAGAGTTGGGTCTGCCAGCAACGCTTGGTAGGTATGACCTTGTGGTCATAAACATCCACACACCTTTTCGCATACACCATTACCAACAGTGCGTCGACCACGCAATGAAACTGCAAATGCTCGGGGGTGACTCATTGAGACTGCTCAAACCGGGCGGCTCTCTATTGATCAGAGCATATGGTTACGCAGATAGAACCAGTGAACGAGTCATCTGCGTATTGGGACGCAAGTTTAGATCGTCTAGAGCGTTGAAACCACCATGTGTCACCAGCAACACTGAGATGTTTTTCCTATTCAGCAACTTTGACAATGGCAGAAGGAATTTCACAACTCATGTCATGAACAATCAACTGAATGCAGCCTTCGTAGGACAGGTCACCCGAGCAGGATGTGCACCGTCGTACCGGGTAAAACGCATGGACATCGCGAAGAACGATGAAGAGTGCGTAGTCAACGCCGCTAACCCTCGCGGGTTACCGGGTGACGGTGTTTGCAAGGCAGTATACAAAAAATGGCCGGAGTCCTTTAAGAACAGTGCAACACCAGTGGGAACCGCAAAAACAGTTATGTGCGGTACGTATCCAGTAATCCACGCTGTTGGACCAAACTTCTCTAATTATTCGGAGTCTGAAGGGGACCGGGAATTGGCAGCTGCCTATCGAGAAGTCGCAAAGGAAGTAACTAGGCTGGGAGTAAATAGTGTAGCTATACCTCTCCTCTCCACAGGTGTATACTCAGGAGGGAAAGACAGGCTGACCCAGTCACTGAACCACCTCTTTACAGCCATGGACTCGACGGATGCAGACGTGGTCATCTACTGCCGCGACAAAGAATGGGAGAAGAAAATATCTGAGGCCATACAGATGCGGACCCAAGTAGAGCTGCTGGATGAGCACATCTCCATAGACTGCGATATTGTTCGCGTGCACCCTGACAGCAGCTTGGCAGGCAGAAAAGGATACAGCACCACGGAAGGCGCACTGTACTCATATCTAGAAGGGACCCGTTTTCATCAGACGGCTGTGGATATGGCGGAGATACATACTATGTGGCCAAAGCAAACAGAGGCCAATGAGCAAGTCTGCCTATATGCCCTGGGGGAAAGTATTGAATCGATCAGGCAGAAATGCCCGGTGGATGATGCAGACGCATCATCTCCCCCCAAAACTGTCCCGTGCCTTTGCCGTTACGCTATGACTCCAGAACGCGTCACCCGGCTTCGCATGAACCACGTCACAAGCATAATTGTGTGTTCTTCGTTTCCCCTCCCAAAGTACAAAATAGAAGGAGTGCAAAAAGTCAAATGCTCTAAGGTAATGCTATTTGACCACAACGTGCCATCGCGCGTAAGTCCAAGGGAATATAGATCTTCCCAGGAGTCTGCACAGGAGGCGAGTACAATCACGTCACTGACGCATAGTCAATTCGACCTAAGCGTTGATGGCGAGATACTGCCCGTCCCGTCAGACCTGGATGCTGACGCCCCAGCCCTAGAACCAGCACTAGACGACGGGGCGACACACACGCTGCCATCCACAACCGGAAACCTTGCGGCCGTGTCTGACTGGGTAATGAGCACCGTACCTGTCGCGCCGCCCAGAAGAAGGCGAGGGAGAAACCTGACTGTGACATGTGACGAGAGAGAAGGGAATATAACACCCATGGCTAGCGTCCGATTCTTTAGGGCAGAGCTGTGTCCGGTCGTACAAGAAACAGCGGAGACGCGTGACACAGCAATGTCTCTTCAGGCACCACCGAGTACCGCCACGGAACCGAATCATCCGCCGATCTCCTTCGGAGCATCAAGCGAGACGTTCCCCATTACATTTGGGGACTTCAACGAAGGAGAAATCGAAAGCTTGTCTTCTGAGCTACTAACTTTCGGAGACTTCTTACCAGGAGAAGTGGATGACTTGACAGACAGCGACTGGTCCACGTGCTCAGACACGGACGACGAGTTATGACTAGACAGGGCAGGTGGGTATATATTCTCGTCGGACACCGGTCCAGGTCATTTACAACAGAAGTCAGTACGCCAGTCAGTGCTGCCGGTGAACACCCTGGAGGAAGTCCACGAGGAGAAGTGTTACCCACCTAAGCTGGATGAAGCAAAGGAGCAACTATTACTTAAGAAACTCCAGGAGAGTGCATCCATGGCCAACAGAAGCAGGTATCAGTCGCGCAAAGTAGAAAACATGAAAGCAGCAATCATCCAGAGACTAAAGAGAGGCTGTAGACTATACTTAATGTCAGAGACCCCAAAAGTCCCTACTTACCGGACTACATATCCGGCGCCTGTGTACTCGCCTCCGATCAACGTCCGATTGTCCAATCCCGAGTCCGCAGTGGCAGCATGCAATGAGTTCTTAGCTAGAAACTATCCAACTGTCTCATCATACCAAATTACCGACGAGTATGATGCATATCTAGACATGGTGGACGGGTCGGAGAGTTGCCTGGACCGAGCGACATTCAATCCGTCAAAACTCAGGAGCTACCCGAAACAGCACGCTTACCACGCGCCCTCCATCAGAAGCGCTGTACCGTCCCCATTCCAGAACACACTACAGAATGTACTGGCAGCAGCCACGAAAAGAAACTGCAACGTCACACAGATGAGGGAATTACCCACTTTGGACTCAGCAGTATTCAACGTGGAGTGTTTCAAAAAGTTCGCATGCAACCAAGAATACTGGGAAGAATTTGCTGCCAGCCCTATTAGGATAACAACTGAGAATTTAGCAACCTATGTTACTAAACTAAAAGGGCCAAAAGCAGCAGCGCTATTCGCAAAAACCCATAATCTACTGCCACTACAGGAAGTACCAATGGATAGGTTCACAGTAGATATGAAAAGGGACGTGAAGGTGACTCCTGGTACAAAGCATACAGAGGAAAGACCTAAGGTGCAGGTTATACAGGCGGCTGAACCCTTGGCGACAGCATACCTATGTGGGATTCACAGAGAGCTGGTTAGGAGGCTGAACGCCGTCCTCCTACCCAATGTACATACACTATTTGACATGTCTGCCGAGGATTTCGATGCCATCATAGCCGCACACTTTAAGCCAGGAGACACTGTTTTGGAAACGGACATAGCCTCCTTTGATAAGAGCCAAGATGATTCACTTGCGCTTACTGCTTTGATGCTGTTAGAGGATTTAGGGGTGGATCACTCCCTGCTGGACTTGATAGAGGCTGCTTTCGGAGAGATTTCCAGCTGTCACCTACCGACAGGTACGCGCTTCAAGTTCGGCGCCATGATGAAATCAGGTATGTTCCTAACTCTGTTCGTCAACACATTGTTAAACATCACCATCGCCAGCCGAGTGCTGGAAGATCGTCTGACAAAATCCGCGTGCGCGGCCTTCATCGGCGACGACAACATAATACATGGAGTCGTCTCCGATGAATTGATGGCAGCCAGATGTGCCACTTGGATGAACATGGAAGTGAAGATCATAGATGCAGTTGTATCCTTGAAAGCCCCTTACTTTTGTGGAGGGTTTATACTGCACGATACTGTGACAGGAACAGCTTGCAGAGTGGCAGACCCGCTAAAAAGGCTTTTTAAACTGGGCAAACCGCTAGCGGCAGGTGACGAACAAGATGAAGATAGAAGACGAGCGCTGGCTGACGAAGTGATCAGATGGCAACGAACAGGGCTAATTGATGAGCTGGAGAAAGCGGTATACTCTAGGTACGAAGTGCAGGGTATATCAGTTGTGGTAATGTCCATGGCCACCTTTGCAAGCTCCAGATCCAACTTCGAGAAGCTCAGAGGACCCGTCATAACTTTGTACGGCGGTCCTAAATAGGTACGCACTACAGCTACCTATTTTGCAGAAGCCGACAGCAAGTATCTAAACACTAATCAGCTACAATGGAGTTCATCCCAACCCAAACTTTTTACAATAGGAGGTACCAGCCTCGACCCTGGACTCCGCGCTCTACTATCCAAATCATCAGGCCCAGACCGCGCCCTCAGAGGCAAGCTGGGCAACTTGCCCAGCTGATCTCAGCAGTTAATAAACTGACAATGCGCGCGGTACCCCAACAGAAGCCACGCAGGAATCGGAAGAATAAGAAGCAAAAGCAAAAACAACAGGCGCCACAAAACAACACAAATCAAAAGAAGCAGCCACCTAAAAAGAAACCGGCTCAAAAGAAAAAGAAGCCGGGCCGCAGAGAGAGGATGTGCATGAAAATCGAAAATGATTGTATTTTCGAAGTCAAGCACGAAGGTAAGGTAACAGGTTACGCGTGCCTGGTGGGGGACAAAGTAATGAAACCAGCACACGTAAAGGGGACCATCGATAACGCGGACCTGGCCAAACTGGCCTTTAAGCGGTCATCTAAGTATGACCTTGAATGCGCGCAGATACCCGTGCACATGAAGTCCGACGCTTCGAAGTTCACCCATGAGAAACCGGAGGGGTACTACAACTGGCACCACGGAGCAGTACAGTACTCAGGAGGCCGGTTCACCATCCCTACAGGTGCTGGCAAACCAGGGGACAGCGGCAGACCGATCTTCGACAACAAGGGACGCGTGGTGGCCATAGTCTTAGGAGGAGCTAATGAAGGAGCCCGTACAGCCCTCTCGGTGGTGACCTGGAATAAAGACATTGTCACTAAAATCACCCCCGAGGGGGCCGAAGAGTGGAGTCTTGCCATCCCAGTTATGTGCCTGTTGGCAAATACCACGTTCCCCTGCTCCCAGCCCCCTTGCACGCCCTGCTGCTACGAAAAGGAACCGGAGGAAACCCTACGCATGCTTGAGGACAACGTCATGAGACCTGGGTACTATCAGCTGCTACAAGCATCCTTAACATGTTCTCCCCACCGCCAGCGACGCAGCACCAAGGACAACTTCAATGTCTATAAAGCCACAAGACCATACTTAGCTCACTGTCCCGACTGTGGAGAAGGGCACTCGTGCCATAGTCCCGTAGCACTAGAACGCATCAGAAATGAAGCGACAGACGGGACGCTGAAAATCCAGGTCTCCTTGCAAATCGGAATAAAGACGGATGACAGCCACGATTGGACCAAGCTGCGTTATATGGACAACCACATGCCAGCAGACGCAGAGAGGGCGGGGCTATTTGTAAGAACATCAGCACCGTGTACGATTACTGGAACAATGGGACACTTCATCCTGGCCCGATGTCCAAAAGGGGAAACTCTGACGGTGGGATTCACTGACAGTAGGAAGATTAGTCATTCATGTACGCACCCATTTCACCACGACCCTCCTGTGATAGGTCGGGAAAAATTCCATTCCCGACCGCAGCACGGTAAAGAGCTACCTTGCAGCACGTACGTGCAGAGCACCGCCGCAACTACCGAGGAGATAGAGGTACACATGCCCCCAGACACCCCTGATCGCACATTAATGTCACAACAGTCCGGCAACGTAAAGATCACAGTCAATGGCCAGACGGTGCGGTACAAGTGTAATTGCGGTGGCTCAAATGAAGGACTAACAACTACAGACAAAGTGATTAATAACTGCAAGGTTGATCAATGTCATGCCGCGGTCACCAATCACAAAAAGTGGCAGTATAACTCCCCTCTGGTCCCGCGTAATGCTGAACTTGGGGACCGAAAAGGAAAAATTCACATCCCGTTTCCGCTGGCAAATGTAACATGCAGGGTGCCTAAAGCAAGGAACCCCACCGTGACGTACGGGAAAAACCAAGTCATCATGCTACTGTATCCTGACCACCCAACACTCCTGTCCTACCGGAATATGGGAGAAGAACCAAACTATCAAGAAGAGTGGGTGATGCATAAGAAGGAAGTCGTGCTAACCGTGCCGACTGAAGGGCTCGAGGTCACGTGGGGCAACAACGAGCCGTATAAGTATTGGCCGCAGTTATCTACAAACGGTACAGCCCATGGCCACCCGCATGAGATAATTCTGTATTATTATGAGCTGTACCCTACTATGACTGTAGTAGTTGTGTCAGTGGCCACGTTCATACTCCTGTCGATGGTGGGTATGGCAGCGGGGATGTGCATGTGTGCACGACGCAGATGCATCACACCGTATGAACTGACACCAGGAGCTACCGTCCCTTTCCTGCTCAGCCTAATATGCTGCATCAGAACAGCTAAAGCGGCCACATACCAAGAGGCTGCGATATACCTGTGGAACGAGCAGCAACCTTTGTTTTGGCTACAAGCCCTTATTCCGCTGGCAGCCCTGATTGTTCTATGCAACTGTCTGAGACTCTTACCATGCTGCTGTAAAACGTTGGCTTTTTTAGCCGTAATGAGCGTCGGTGCCCACACTGTGAGCGCGTACGAACACGTAACAGTGATCCCGAACACGGTGGGAGTACCGTATAAGACTCTAGTCAATAGACCTGGCTACAGCCCCATGGTATTGGAGATGGAACTACTGTCAGTCACTTTGGAGCCAACACTATCGCTTGATTACATCACGTGCGAGTACAAAACCGTCATCCCGTCTCCGTACGTGAAGTGCTGCGGTACAGCAGAGTGCAAGGACAAAAACCTACCTGACTACAGCTGTAAGGTCTTCACCGGCGTCTACCCATTTATGTGGGGCGGCGCCTACTGCTTCTGCGACGCTGAAAACACGCAGTTGAGCGAAGCACATGTGGAGAAGTCCGAATCATGCAAAACAGAATTTGCATCAGCATACAGGGCTCATACCGCATCTGCATCAGCTAAGCTCCGCGTCCTTTACCAAGGAAATAACATCACTGTAACTGCCTATGCAAACGGCGACCATGCCGTCACAGTTAAGGACGCCAAATTCATTGTGGGGCCAATGTCTTCAGCCTGGACACCTTTCGACAACAAAATTGTGGTGTACAAAGGTGACGTCTATAACATGGACTACCCGCCCTTTGGCGCAGGAAGACCAGGACAATTTGGCGATATCCAAAGTCGCACACCTGAGAGTAAAGACGTCTATGCTAATACACAACTGGTACTGCAGAGACCGGCTGCGGGTACGGTACACGTGCCATACTCTCAGGCACCATCTGGCTTTAAGTATTGGCTAAAAGAACGCGGGGCGTCACTGCAGCACACAGCACCATTTGGCTGCCAAATAGCAACAAACCCGGTAAGAGCGGTGAACTGCGCCGTAGGGAACATGCCCATCTCCATCGACATACCGGAAGCGGCCTTCACTAGGGTCGTCGACGCGCCCTCTTTAACGGACATGTCGTGCGAGGTACCAGCCTGCACCCATTCCTCAGACTTTGGGGGCGTCGCCATTATTAAATATGCAGCCAGCAAGAAAGGCAAGTGTGCGGTGCATTCGATGACTAACGCCGTCACTATTCGGGAAGCTGAGATAGAAGTTGAAGGGAATTCTCAGCTGCAAATCTCTTTCTCGACGGCCTTAGCCAGCGCCGAATTCCGCGTACAAGTCTGTTCTACACAAGTACACTGTGCAGCTGAGTGCCACCCCCCGAAGGACCACATAGTCAACTACCCGGCGTCACATACCACCCTCGGGGTCCAGGACATCTCCGCTACGGCGATGTCATGGGTGCAGAAGATCACGGGAGGTGTGGGACTGGTTGTTGCTGTTGCCGCACTGATTCTAATCGTGGTGCTATGCGTGTCGTTCAGCAGGCACTAACTTGACAATTAAGTATGAAGGTATATGTGTCCCCTAAGAGACACACTGTACATAGCAAATAATCTATAGATCAAAGGGCTACGCAACCCCTGAATAGTAACAAAATACAAAATCACTAAAAATTATAAAAACAGAAAAATACATAAATAGGTATACGTGTCCCCTAAGAGACACATTGTATGTAGGTGATAAGTATAGATCAAAGGGCCGAATAACCCCTGAATAGTAACAAAATATGAAAATCAATAAAAATCATAAAATAGAAAAACCATAAACAGAAGTAGTTCAAAGGGCTATAAAACCCCTGAATAGTAACAAAACATAAAGTTAATAAAAATCAAATGAATACCATAATTGGCAAACGGAAGAGATGTAGGTACTTAAGCTTCCTAAAAGCAGCCGAACTCACTTTGAGAAGTAGGCATAGCATACCGAACTCTTCCACGATTTCGAAACCACAC

>EU564335|2006|India: Rajasthan, Group 4

ATGGCTGCGTGAGACACACGTAGCCTACCAGTTTCTTACTGCTCTACTCTGCAAAGCAAGAGATTAATAACCCATCATGGATCCTGTGTACGTGGACATAGACGCTGACAGCGCCTTTTTGAAGGCCCTGCAACGTGCGTACCCCATGTTTGAGGTGGAACCAAGGCAGGTCACACCGAATGACCATGCTAATGCTAGAGCGTTCTCGCATCTAGCTATAAAACTAATAGAGCAGGAAATTGACCCCGACTCAACCATCCTGGATATCGGCAGTGCGCCAGCAAGGAGGATGATGTCGGACAGGAAGTACCACTGCGTCTGCCCGATGCGCAGTGCGGAAGATCCCGAGAGACTCGCTAATTATGCGAGAAAGCTAGCATCTGCCGCAGGAAAAGTCCTGGACAGAAACATCTCTGGAAAGATCGGGGACTTACAAGCAGTAATGGCCGTGCCAGACAAGGAGACGCCAACATTCTGCTTACACACAGACGTCTCATGTAGACAGAGAGCAGACGTCGCTATATACCAAGACGTCTATGCTGTACACGCACCCACGTCGCTATACCACCAGGCGATTAAAGGGGTCCGAGTGGCGTACTGGGTTGGGTTCGACACAACCCCGTTCATGTACAATGCCATGGCGGGTGCCTACCCCTCATACTCGACAAACTGGGCAGATGAGCAGGTACTGAAGGCTAAGAACATAGGATTATGTTCAACAGACCTGACGGAAGGTAGACGAGGCAAGTTGTCTATTATGAGAGGGAAAAAGCTAAAACCGTGCGACCGTGTGCTGTTCTCAGTAGGGTCAACGCTCTACCCGGAAAGCCGCAAGCTACTTAAGAGCTGGCACCTGCCATCGGTGTTCCATTTAAAGGGCAAACTCAGCTTCACATGCCGCTGTGATACAGTGGTTTCGTGTGAGGGCTACGTCGTTAAGAGAATAACGATGAGCCCAGGCCTTTATGGAAAAACCACAGGGTATGCGGTAACCCACCACGCAGACGGATTCCTGATGTGCAAGACTACCGACACGGTTGACGGCGAAAGAGTGTCATTCTCGGTGTGCACATACGTGCCGGCGACCATTTGTGATCAAATGACCGGCATCCTTGCTACAGAAGTCACGCCGGAGGATGCACAGAAGCTGTTGGTGGGGCTGAACCAGAGAATAGTGGTTAACGGCAGAACGCAACGGAATATGAACACCATGAAAAATTATCTGCTTCCCGTGGTCGCCCAAGCCTTCAGTAAGTGGGCAAAGGAGTGCCGGAAAGACATGGAAGATGAAAAACTCCTGGGGGTCAGAGAAAGAACACTGACCTGCTGCTGTCTATGGGCATTCAAGAAGCAGAAAACACACACGGTCTACAAGAGGCCTGATACCCAGTCAATTCAGAAGGTTCAGGCCGAGTTTGACAGCTTTGTGGTACCGAGTCTGTGGTCGTCCGGGTTGTCAATCCCTTTGAGGACTAGAATCAAATGGTTGTTAAGCAAGGTGCCAAAAACCGACCTGATCCCATACAGCGGAGACGCCCGAGAAGCCCGGGACGCAGAAAAAGAAGCAGAGGAAGAACGAGAAGCAGAACTGACTCGCGAAGCCCTACCACCTCTACAGGCAGCACAGGAAGATGTTCAGGTCGAAATCGACGTGGAACAGCTTGAGGACAGAGCGGGCGCAGGAATAATAGAGACTCCGAGAGGAGCTATCAAAGTTACTGCCCAACCAACAGACCACGTCGTGGGAGAGTACCTGGTACTCTCCCCGCAGACCGTACTACGTAGCCAGAAGCTCAGTCTGATTCACGCTTTGGCGGAGCAAGTGAAGACGTGCACGCACAACGGACGAGCAGGGAGGTATGCGGTCGAAGCGTACGACGGCCGAGTCCTAGTGCCCTCAGGCTATGCAATCTCGCCTGAAGACTTCCAGAGTCTAAGCGAAAGCGCAACGATGGTGTATAACGAAAGAGAGTTCGTAAACAGAAAGCTACACCATATTGCGATGCACGGACCAGCCCTGAACACCGACGAAGAGTCGTATGAGCTGGTGAGGGCAGAGAGGACAGAACACGAGTACGTCTACGACGTGGATCAGAGAAGATGCTGTAAGAAGGAAGAAGCCGCAGGACTGGTACTGGTGGGCGACTTGACTAATCCGCCCTACCACGAATTCGCATATGAAGGGCTAAAAATCCGCCCTGCCTGCCCATACAAAATTGCAGTCATAGGAGTCTTCGGAGTACCGGGATCTGGCAAGTCAGCTATTATCAAGAACCTAGTTACCAGGCAGGACCTGGTGACTAGCGGAAAGAAAGAAAACTGCCAAGAAATCACCACCGACGTGATGAGACAGAGAGGTCTAGAGATATCTGCACGTACGGTTGACTCGCTGCTCTTGAATGGATGCAACAGACCAGTCGACGTGTTGTACGTAGACGAGGCGTTTGCGTGCCACTCTGGAACGCTACTTGCTTTGATCGCCTTGGTGAGACCAAGGCAGAAAGTTGTACTTTGTGGTGACCCGAAGCAGTGCGGCTTCTTCAATATGATGCAGATGAAAGTCAACTATAATCACAACATCTGCACCCAAGTGTACCACAAAAGTATCTCCAGGCGGTGTACACTGCCTGTGACCGCCATTGTGTCATCGTTGCATTACGAAGGCAAAATGCGCACTACGAATGAGTACAACAAGCCGATTGTAGTGGACACTACAGGCTCAACAAAACCTGACCCTGGAGACCTCGTGTTAACGTGCTTCAGAGGGTGGGTTAAACAACTGCAAATTGACTATCGTGGATACGAGGTCATGACAGCAGCCGCATCCCAAGGGTTAACCAGAAAAGGAGTTTACGCAGTTAGACAAAAAGTTAATGAAAACCCGCTCTATGCATCAACGTCAGAGCACGTCAACGTACTCCTAACGCGTACGGAAGGTAAACTGGTATGGAAGACACTTTCCGGCGACCCGTGGATAAAGACGCTGCAGAACCCACCGAAAGGAAACTTCAAAGCAACTATTAAGGAGTGGGAGGTGGAGCATGCATCAATAATGGCGGGCATCTGCAGTCACCAAATGACCTTCGATACATTCCAAAATAAAGCCAACGTTTGTTGGGCTAAGAGCTTGGTCCCTATCCTCGAAACAGCGGGGATAAAACTAAATGATAGGCAGTGGTCTCAGATAATTCAAGCCTTCAAAGAAGACAAAGCATACTCACCTGAAGTAGCCCTGAATGAAATATGTACGCGCATGTATGGGGTGGATCTAGACAGCGGGCTATTTTCTAAACCGTTGGTGTCTGTGTATTACGCGGATAACCACTGGGATAATAGGCCTGGAGGGAAAATGTTCGGATTTAACCCCGAGGCAGCATCCATTCTAGAAAGAAAGTATCCATTCACAAAAGGGAAGTGGAACATCAACAAGCAGATCTGCGTGACTACCAGGAGGATAGAAGACTTTAACCCTACCACCAACATCATACCGGCCAACAGGAGACTACCACACTCATTAGTGGCCGAACACCGCCCAGTAAAAGGGGAAAGAATGGAATGGCTGGTTAACAAGATAAACGGCCACCACGTGCTCCTGGTCAGTGGCTATAACCTTGCACTGCCTACTAAGAGAGTCACTTGGGTAGCGCCGTTAGGTGTCCGCGGAGCGGACTACACATACAACCTAGAGTTGGGTCTGCCAGCAACGCTTGGTAGGTATGACCTTGTGGTCATAAACATCCACACACCTTTTCGCATACACCATTACCAACAGTGCGTCGACCACGCAATGAAACTGCAAATGCTCGGGGGTGACTCATTGAGACTGCTCAAACCGGGCGGCTCTCTATTGATCAGAGCATATGGTTACGCAGATAGAACCAGTGAACGAGTCATCTGCGTATTGGGACGCAAGTTTAGATCGTCTAGAGCGTTGAAACCACCATGTGTCACCAGCAACACTGAGATGTTTTTCCTATTCAGCAACTTTGACAATGGCAGAAGGAATTTCACAACTCATGTCATGAACAATCAACTGAATGCAGCCTTCGTAGGACAGGTCACCCGAGCAGGATGTGCACCGTCGTACCGGGTAAAACGCATGGACATCGCGAAGAACGATGAAGAGTGCGTAGTCAACGCCGCTAACCCTCGCGGGTTACCGGGTGACGGTGTTTGCAAGGCAGTATACAAAAAATGGCCGGAGTCCTTTAAGAACAGTGCAACACCAGTGGGAACCGCAAAAACAGTTATGTGCGGTACGTATCCAGTAATCCACGCTGTTGGACCAAACTTCTCTAATTATTCGGAGTCTGAAGGGGACCGGGAATTGGCAGCTGCCTATCGAGAAGTCGCAAAGGAAGTAACTAGGCTGGGAGTAAATAGTGTAGCTATACCTCTCCTCTCCACAGGTGTATACTCAGGAGGGAAAGACAGGCTGACCCAGTCACTGAACCACCTCTTTACAGCCATGGACTCGACGGATGCAGACGTGGTCATCTACTGCCGCGACAAAGAATGGGAGAAGAAAATATCTGAGGCCATACAGATGCGGACCCAAGTAGAGCTGCTGGATGAGCACATCTCCATAGACTGCGATATTGTTCGCGTGCACCCTGACAGCAGCTTGGCAGGCAGAAAAGGATACAGCACCACGGAAGGCGCACTGTACTCATATCTAGAAGGGACCCGTTTTCATCAGACGGCTGTGGATATGGCGGAGATACATACTATGTGGCCAAAGCAAACAGAGGCCAATGAGCAAGTCTGCCTATATGCCCTGGGGGAAAGTATTGAATCGATCAGGCAGAAATGCCCGGTGGATGATGCAGACGCATCATCTCCCCCCAAAACTGTCCCGTGCCTTTGCCGTTACGCTATGACTCCAGAACGCGTCACCCGGCTTCGCATGAACCACGTCACAAGCATAATTGTGTGTTCTTCGTTTCCCCTCCCAAAGTACAAAATAGAAGGAGTGCAAAAAGTCAAATGCTCTAAGGTAATGCTATTTGACCACAACGTGCCATCGCGCGTAAGTCCAAGGGAATATAGATCTTCCCAGGAGTCTGCACAGGAGGCGAGTACAATCACGTCACTGACGCATAGTCAATTCGACCTAAGCGTTGATGGCGAGATACTGCCCGTCCCGTCAGACCTGGATGCTGACGCCCCAGCCCTAGAACCAGCACTAGACGACGGGGCGACACACACGCTGCCATCCACAACCGGAAACCTTGCGGCCGTGTCTGACTGGGTAATGAGCACCGTACCTGTCGCGCCGCCCAGAAGAAGGCGAGGGAGAAACCTGACTGTGACATGTGACGAGAGAGAAGGGAATATAACACCCATGGCTAGCGTCCGATTCTTTAGGGCAGAGCTGTGTCCGGTCGTACAAGAAACAGCGGAGACGCGTGACACAGCAATGTCTCTTCAGGCACCACCGAGTACCGCCACGGAACCGAATCATCCGCCGATCTCCTTCGGAGCATCAAATGAGACGTTCCCCATTACATTTGGGGACTTCAACGAAGGAGAAATCGAAAGCTTGTCTTCTGAGCTACTAACTTTCGGAGACTTCTTACCAGGAGAAGTGGATGACTTGACAGACAGCGACTGGTCCACGTGCTCAGACACGGACGACGAGTTATGACTAGACAGGGCAGGTGGGTATATATTCTCGTCGGACACCGGTCCAGGTCATTTACAACAGAAGTCAGTACGCCAGTCAGTGCTGCCGGTGAACACCCTGGAGGAAGTCCACGAGGAGAAGTGTTACCCACCTAAGCTGGATGAAGCAAAGGAGCAACTATTACTTAAGAAACTCCAGGAGAGTGCATCCATGGCCAACAGAAGCAGGTATCAGTCGCGCAAAGTAGAAAACATGAAAGCAGCAATCATCCAGAGACTAAAGAGAGGCTGTAGACTATACTTAATGTCAGAGACCCCAAAAGTCCCTACTTACCGGACTACATATCCGGCGCCTGTGTACTCGCCTCCGATCAACGTCCGATTGTCCAATCCCGAGTCCGCAGTGGCAGCATGCAATGAGTTCTTAGCTAGAAACTATCCAACTGTCTCATCATACCAAATTACCGACGAGTATGATGCATATCTAGACATGGTGGACGGGTCGGAGAGTTGCCTGGACCGAGCGACATTCAATCCGTCAAAACTCAGGAGCTACCCGAAACAGCACGCTTACCACGCGCCCTCCATCAGAAGCGCTGTACCGTCCCCATTCCAGAACACACTACAGAATGTACTGGCAGCAGCCACGAAAAGAAACTGCAACGTCACACAGATGAGGGAATTACCCACTTTGGACTCAGCAGTATTCAACGTGGAGTGTTTCAAAAAATTCGCATGCAACCAAGAATACTGGGAAGAATTTGCTGCCAGCCCTATTAGGATAACAACTGAGAATTTAGCAACCTATGTTACTAAACTAAAAGGGCCAAAAGCAGCAGCGCTATTCGCAAAAACCCATAATCTACTGCCACTACAGGAAGTACCAATGGATAGGTTCACAGTAGATATGAAAAGGGACGTGAAGGTGACTCCTGGTACAAAGCATACAGAGGAAAGACCTAAGGTGCAGGTTATACAGGCGGCTGAACCCTTGGCGACAGCATACCTATGTGGGATTCACAGAGAGCTGGTTAGGAGGCTGAACGCCGTCCTCCTACCCAATGTACATACACTATTTGATATGTCTGCCGAGGATTTCGATGCCATCATAGCCGCACACTTTAAGCCAGGAGACACTGTTTTGGAAACGGACATAGCCTCCTTTGATAAGAGCCAAGATGATTCACTTGCGCTTACTGCTTTGATGCTGTTAGAGGATTTAGGGGTGGATCACTCCCTGCTGGACTTGATAGAGGCTGCTTTCGGAGAGATTTCCAGCTGTCACCTACCGACAGGTACGCGCTTCAAGTTCGGCGCCATGATGAAATCAGGTATGTTCCTAACTCTGTTCGTCAACACATTGTTAAACATCACCATCGCCAGCCGAGTGCTGGAAGATCGTCTGACAAAATCCGCGTGCGCGGCCTTCATCGGCGACGACAACATAATACATGGAGTCGTCTCCGATGAATTGATGGCAGCCAGATGTGCCACTTGGATGAACATGGAAGTGAAGATCATAGATGCAGTTGTATCCTTGAAAGCCCCTTACTTTTGTGGAGGGTTTATACTGCACGATACTGTGACAGGAACAGCTTGCAGAGTGGCAGACCCGCTAAAAAGGCTTTTTAAACTGGGCAAACCGCTAGCGGCAGGTGACGAACAAGATGAAGATAGAAGACGAGCGCTGGCTGACGAAGTGATCAGATGGCAACGAACAGGGCTAATTGATGAGCTGGAGAAAGCGGTATACTCTAGGTACGAAGTGCAGGGTATATCAGTTGTGGTAATGTCCATGGCCACCTTTGCAAGCTCCAGATCCAACTTCGAGAAGCTCAGAGGACCCGTCATAACTTTGTACGGCGGTCCTAAATAGGTACGCACTACAGCTACCTATTTTGCAGAAGCCGACAGCAAGTATCTAAACACTAATCAGCTACAATGGAGTTCATCCCAACCCAAACTTTTTACAATAGGAGGTACCAGCCTCGACCCTGGACTCCGCGCTCTACTATCCAAATCATCAGGCCCAGACCGCGCCCTCAGAGGCAAGCTGGGCAACTTGCCCAGCTGATCTCAGCAGTTAATAAACTGACAATGCGCGCGGTACCCCAACAGAAGCCACGCAGGAATCGGAAGAATAAGAAGCAAAAGCAAAAACAACAGGCGCCACAAAACAACACAAATCAAAAGAAGCAGCCACCTAAAAAGAAACCGGCTCAAAAGAAAAAGAAGCCGGGCCGCAGAGAGAGGATGTGCATGAAAATCGAAAATGATTGTATTTTCGAAGTCAAGCACGAAGGTAAGGTAACAGGTTACGCGTGCCTGGTGGGGGACAAAGTAATGAAACCAGCACACGTAAAGGGGACCATCGATAACGCGGACCTGGCCAAACTGGCCTTTAAGCGGTCATCTAAGTATGACCTTGAATGCGCGCAGATACCCGTGCACATGAAGTCCGACGCTTCGAAGTTCACCCATGAGAAACCGGAGGGGTACTACAACTGGCACCACGGAGCAGTACAGTACTCAGGAGGCCGGTTCACCATCCCTACAGGTGCTGGCAAACCAGGGGACAGCGGCAGACCGATCTTCGACAACAAGGGACGCGTGGTGGCCATAGTCTTAGGAGGAGCTAATGAAGGAGCCCGTACAGCCCTCTCGGTGGTGACCTGGAATAAAGACATTGTCACTAAAATCACCCCCGAGGGGGCCGAAGAGTGGAGTCTTGCCATCCCAGTTATGTGCCTGTTGGCAAACACCACGTTCCCCTGCTCCCAGCCCCCTTGCACGCCCTGCTGCTACGAAAAGGAACCGGAGGAAACCCTACGCATGCTTGAGGACAACGTCATGAGACCTGGGTACTATCAGCTGCTACAAGCATCCTTAACATGTTCTCCCCACCGCCAGCGACGCAGCACCAAGGACAACTTCAATGTCTATAAAGCCACAAGACCATACTTAGCTCACTGTCCCGACTGTGGAGAAGGGCACTCGTGCCATAGTCCCGTAGCACTAGAACGCATCAGAAATGAAGCGACAGACGGGACGCTGAAAATCCAGGTCTCCTTGCAAATCGGAATAAAGACGGATGACAGCCACGATTGGACCAAGCTGCGTTATATGGACAACCACATGCCAGCAGACGCAGAGAGGGCGGGGCTATTTGTAAGAACATCAGCACCGTGTACGATTACTGGAACAATGGGACACTTCATCCTGGCCCGATGTCCAAAAGGGGAAACTCTGACGGTGGGATTCACTGACAGTAGGAAGATTAGTCATTCATGTACGCACCCATTTCACCACGACCCTCCTGTGATAGGTCGGGAAAAATTCCATTCCCGACCGCAGCACGGTAAAGAGCTACCTTGCAGCACGTACGTGCAGAGCACCGCCGCAACTACCGAGGAGATAGAGGTACACATGCCCCCAGACACCCCTGATCGCACATTAATGTCACAACAGTCCGGCAACGTAAAGATCACAGTCAATGGCCAGACGGTGCGGTACAAGTGTAATTGCGGTGGCTCAAATGAAGGACTAACAACTACAGACAAAGTGATTAATAACTGCAAGGTTGATCAATGTCATGCCGCGGTCACCAATCACAAAAAGTGGCAGTATAACTCCCCTCTGGTCCCGCGTAATGCTGAACTTGGGGACCGAAAAGGAAAAATTCACATCCCGTTTCCGCTGGCAAATGTAACATGCAGGGTGCCTAAAGCAAGGAACCCCACCGTGACGTACGGGAAAAACCAAGTCATCATGCTACTGTATCCTGACCACCCAACACTCCTGTCCTACCGGAATATGGGAGAAGAACCAAACTATCAAGAAGAGTGGGTGATGCATAAGAAGGAAGTCGTGCTAACCGTGCCGACTGAAGGGCTCGAGGTCACGTGGGGCAACAACGAGCCGTATAAGTATTGGCCGCAGTTATCTACAAACGGTACAGCCCATGGCCACCCGCATGAGATAATTCTGTATTATTATGAGCTGTACCCTACTATGACTGTAGTAGTTGTGTCAGTGGCCACGTTCATACTCCTGTCGATGGTGGGTATGGCAGCGGGGATGTGCATGTGTGCACGACGCAGATGCATCACACCGTATGAACTGACACCAGGAGCTACCGTCCCTTTCCTGCTTAGCCTAATATGCTGCATCAGAACAGCTAAAGCGGCCACATACCAAGAGGCTGCGATATACCTGTGGAACGAGCAGCAACCTTTGTTTTGGCTACAAGCCCTTATTCCGCTGGCAGCCCTGATTGTTCTATGCAACTGTCTGAGACTCTTACCATGCTGCTGTAAAACGTTGGCTTTTTTAGCCGTAATGAGCGTCGGTGCCCACACTGTGAGCGCGTACGAACACGTAACAGTGATCCCGAACACGGTGGGAGTACCGTATAAGACTCTAGTCAATAGACCTGGCTACAGCCCCATGGTATTGGAGATGGAACTACTGTCAGTCACTTTGGAGCCAACACTATCGCTTGATTACATCACGTGCGAGTACAAAACCGTCATCCCGTCTCCGTACGTGAAGTGCTGCGGTACAGCAGAGTGCAAGGACAAAAACCTACCTGACTACAGCTGTAAGGTCTTCACCGGCGTCTACCCATTTATGTGGGGCGGCGCCTACTGCTTCTGCGACGCTGAAAACACGCAGTTGAGCGAAGCACATGTGGAGAAGTCCGAATCATGCAAAACAGAATTTGCATCAGCATACAGGGCTCATACCGCATCTGCATCAGCTAAGCTCCGCGTCCTTTACCAAGGAAATAACATCACTGTAACTGCCTATGCAAACGGCGACCATGCCGTCACAGTTAAGGACGCCAAATTCATTGTGGGGCCAATGTCTTCAGCCTGGACACCTTTCGACAACAAAATTGTGGTGTACAAAGGTGACGTCTATAACATGGACTACCCGCCCTTTGGCGCAGGAAGACCAGGACAATTTGGCGATATCCAAAGTCGCACACCTGAGAGTAAAGACGTCTATGCTAATACACAACTGGTACTGCAGAGACCGGCTGCGGGTACGGTACACGTGCCATACTCTCAGGCACCATCTGGCTTTAAGTATTGGCTAAAAGAACGCGGGGCGTCACTGCAGCACACAGCACCATTTGGCTGCCAAATAGCAACAAACCCGGTAAGAGCGGTGAACTGCGCCGTAGGGAACATGCCCATCTCCATCGACATACCGGAAGCGGCCTTCACTAGGGTCGTCGACGCGCCCTCTTTAACGGACATGTCGTGCGAGGTACCAGCCTGCACCCATTCCTCAGACTTTGGGGGCGTCGCCATTATTAAATATGCAGCCAGCAAGAAAGGCAAGTGTGCGGTGCATTCGATGACTAACGCCGTCACTATTCGGGAAGCTGAGATAGAAGTTGAAGGGAATTCTCAGCTGCAAATCTCTTTCTCGACGGCCTTAGCCAGCGCCGAATTCCGCGTACAAGTCTGTTCTACACAAGTACACTGTGCAGCTGAGTGCCACCCCCCGAAGGACCACATAGTCAACTACCCGGCGTCACATACCACCCTCGGGGTCCAGGACATCTCCGCTACGGCGATGTCATGGGTGCAGAAGATCACGGGAGGTGTGGGACTGGTTGTTGCTGTTGCCGCACTGATTCTAATCGTGGTGCTATGCGTGTCGTTCAGCAGGCACTAACTTGACAATTAAGTATGAAGGTATATGTGTCCCCTAAGAGACACACTGTACATAGCAAATAATCTATAGATCAAAGGGCTACGCAACCCCTGAATAGTAACAAAATACAAAATCACTAAAAATTATAAAAACAGAAAAATACATAAATAGGTATACGTGTCCCCTAAGAGACACATTGTATGTAGGTGATAAGTATAGATCAAAGGGCCGAATAACCCCTGAATAGTAACAAAATATGAAAATCAATAAAAATCATAAAATAGAAAAACCATAAACAGAAGTAGTTCAAAGGGCTATAAAACCCCTGAATAGTAACAAAACATAAAATTAATAAAAATCAAATGAATACCATAATTGGCAAACGGAAGAGATGTAGGTACTTAAGCTTCCTAAAAGCAGCCGAACTCACTTGAGAAGTAGGCATAGCATACCGAACTCTTCCACGATTCTCCGAACCCACAGGGACGTAGGAGATGTTATTTTGTTTTTAATATTTC

>FJ000062|2006|India: Gujarat, Group 4

TTTGGCTGCGTGAGACACACGTAGCCTACCAGTTTCTTACTGCTCTACTCTGCAAAGCAAGAGATTAATAACCCATCATGGATCCTGTGTACGTGGACATAGACGCTGACAGCGCCTTTTTGAAGGCCCTGCAACGTGCGTACCCCATGTTTGAGGTGGAACCAAGGCAGGTCACACCGAATGACCATGCTAATGCTAGAGCGTTCTCGCATCTAGCTATAAAACTAATAGAGCAGGAAATTGACCCCGACTCAACCATCCTGGATATCGGCAGTGCGCCAGCAAGGAGGATGATGTCGGACAGGAAGTACCACTGCGTCTGCCCGATGCGCAGTGCGGAAGATCCCGAGAGACTCGCTAATTATGCGAGAAAGCTAGCATCTGCCGCAGGAAAAGTCCTGGACAGAAACATCTCTGGAAAGATCGGGGACTTACAAGCAGTAATGGCCGTGCCAGACAAGGAGACGCCAACATTCTGCTTACACACAGACGTCTCATGTAGACAGAGAGCAGACGTCGCTATATACCAAGACGTCTATGCTGTACACGCACCCACGTCGCTATACCACCAGGCGATTAAAGGGGTCCGAGTGGCGTACTGGGTTGGGTTCGACACAACCCCGTTCATGTACAATGCCATGGCGGGTGCCTACCCCTCATACTCGACAAACTGGGCAGATGAGCAGGTACTGAAGGCTAAGAACATAGGATTATGTTCAACAGACCTGACGGAAGGTAGACGAGGCAAGTTGTCTATTATGAGAGGGAAAAAGCTAAAACCGTGCGACCGTGTGCTGTTCTCAGTAGGGTCAACGCTCTACCCGGAAAGCCGCAAGCTACTTAAGAGCTGGCACCTGCCATCGGTGTTCCATTTAAAGGGCAAACTCAGCTTCACATGCCGCTGTGATACAGTGGTTTCGTGTGAGGGCTACGTCGTTAAGAGAATAACGATGAGCCCAGGCCTTTATGGAAAAACCACAGGGTATGCGGTAACCCACCACGCAGACGGATTCCTGATGTGCAAGACTACCGACACGGTTGACGGCGAAAGAGTGTCATTCTCGGTGTGCACATACGTGCCGGCGACCATTTGTGATCAAATGACCGGCATCCTTGCTACAGAAGTCACGCCGGAGGATGCACAGAAGCTGTTGGTGGGGCTGAACCAGAGAATAGTGGTTAACGGCAGAACGCAACGGAATATGAACACCATGAAAAATTATCTGCTTCCCGTGGTCGCCCAAGCCTTCAGTAAGTGGGCAAAGGAGTGCCGGAAAGACATGGAAGATGAAAAACTCCTGGGGGTCAGAGAAAGAACACTGACCTGCTGCTGTCTATGGGCATTCAAGAAGCAGAAAACACACACGGTCTACAAGAGGCCTGATACCCAGTCAATTCAGAAGGTTCAGGCCGAGTTTGACAGCTTTGTGGTACCGAGTCTGTGGTCGTCCGGGTTGTCAATCCCTTTGAGGACTAGAATCAAATGGTTGTTAAGCAAGGTGCCAAAAACCGACCTGATCCCATACAGCGGAGACGCCCGAGAAGCCCGGGACGCAGAAAAAGAAGCAGAGGAAGAACGAGAAGCAGAACTGACTCGCGAAGCCCTACCACCTCTACAGGCAGCACAGGAAGATGTTCAGGTCGAAATCGACGTGGAACAGCTTGAGGACAGAGCGGGCGCAGGAATAATAGAGACTCCGAGAGGAGCTATCAAAGTTACTGCCCAACCAACAGACCACGTCGTGGGAGAGTACCTGGTACTCTCCCCGCAGACCGTACTACGTAGCCAGAAGCTCAGTCTGATTCACGCTTTGGCGGAGCAAGTGAAGACGTGCACGCACAACGGACGAGCAGGGAGGTATGCGGTCGAAGCGTACGACGGCCGAGTCCTAGTGCCCTCAGGCTATGCAATCTCGCCTGAAGACTTCCAGAGTCTAAGCGAAAGCGCAACGATGGTGTATAACGAAAGAGAGTTCGTAAACAGAAAGCTACACCATATTGCGATGCACGGACCAGCCCTGAACACCGACGAAGAGTCGTATGAGCTGGTGAGGGCAGAGAGGACAGAACACGAGTACGTCTACGACGTGGATCAGAGAAGATGCTGTAAGAAGGAAGAAGCCGCAGGACTGGTACTGGTGGGCGACTTGACTAATCCGCCCTACCACGAATTCGCATATGAAGGGCTAAAAATCCGCCCTGCCTGCCCATACAAAATTGCAGTCATAGGAGTCTTCGGAGTACCGGGATCTGGCAAGTCAGCTATTATCAAGAACCTGGTTACCAGGCAGGACCTGGTGACTAGCGGAAAGAAAGAAAACTGCCAAGAAATCACCACCGACGTGATGAGACAGAGAGGTCTAGAGATATCTGCACGTACGGTTGACTCGCTGCTCTTGAATGGATGCAACAGACCAGTCGACGTGTTGTACGTAGACGAGGCGTTTGCGTGCCACTCTGGAACGCTACTTGCTTTGATCGCCTTGGTGAGACCAAGGCAGAAAGTTGTACTTTGTGGTGACCCGAAGCAGTGCGGCTTCTTCAATATGATGCAGATGAAAGTCAACTATAATCACAACATCTGCACCCAAGTGTACCACAAAAGTATCTCCAGGCGGTGTACACTGCCTGTGACCGCCATTGTGTCATCGTTGCATTACGAAGGCAAAATGCGCACTACGAATGAGTACAACAAGCCGATTGTAGTGGACACTACAGGCTCAACAAAACCTGACCCTGGAGACCTCGTGTTAACGTGCTTCAGAGGGTGGGTTAAACAACTGCAAATTGACTATCGTGGATACGAGGTCATGACAGCAGCCGCATCCCAAGGGTTAACCAGAAAAGGAGTTTACGCAGTTAGACAAAAAGTTAATGAAAACCCGCTCTATGCATCAACGTCAGAGCACGTCAACGTACTCCTAACGCGTACGGAAGGTAAACTGGTATGGAAGACACTTTCCGGCGACCCGTGGATAAAGACGCTGCAGAACCCACCGAAAGGAAACTTCAAAGCAACTATTAAGGAGTGGGAGGTGGAGCATGCATCAATAATGGCGGGCATCTGCAGTCACCAAATGACCTTCGATACATTCCAAAATAAAGCCAACGTTTGTTGGGCTAAGAGCTTGGTCCCTATCCTCGAAACAGCGGGGATAAAACTAAATGATAGGCAGTGGTCTCAGATAATTCAAGCCTTCAAAGAAGACAAAGCATACTCACCTGAAGTAGCCCTGAATGAAATATGTACGCGCATGTATGGGGTGGATCTAGACAGCGGGCTATTTTCTAAACCGTTGGTGTCTGTGTATTACGCGGATAACCACTGGGATAATAGGCCTGGAGGGAAAATGTTCGGATTTAACCCCGAGGCAGCATCCATTCTAGAAAGAAAGTATCCATTCACAAAAGGGAAGTGGAACATCAACAAGCAGATCTGCGTGACTACCAGGAGGATAGAAGACTTTAACCCTATCACCAACATCATACCGGCCAACAGGAGACTACCACACTCATTAGTGGCCGAACACCGCCCAGTAAAAGGGGAAAGAATGGAATGGCTGGTTAACAAGATAAACGGCCACCACGTGCTCCTGGTCAGTGGCTATAACCTTGCACTGCCTACTAAGAGAGTCACTTGGGTAGCGCCGTTAGGTGTCCGCGGAGCGGACTACACATACAACCTAGAGTTGGGTCTGCCAGCAACGCTTGGTAGGTATGACCTTGTGGTCATAAACATCCACACACCTTTTCGCATACACCATTACCAACAGTGCGTCGACCACGCAATGAAACTGCAAATGCTCGGGGGTGACTCATTGAGACTGCTCAAACCGGGCGGCTCTCTATTGATCAGAGCATATGGTTACGCAGATAGAACCAGTGAACGAGTCATCTGCGTATTGGGACGCAAGTTTAGATCGTCTAGAGCGTTGAAACCACCATGTGTCACCAGCAACACTGAGATGTTTTTCCTATTCAGCAACTTTGACAATGGCAGAAGGAATTTCACAACTCATGTCATGAACAATCAACTGAATGCAGCCTTCGTAGGACAGGTCACCCGAGCAGGATGTGCACCGTCGTACCGGGTAAAACGCATGGACATCGCGAAGAACGATGAAGAGTGCGTAGTCAACGCCGCTAACCCTCGCGGGTTACCGGGTGACGGTGTTTGCAAGGCAGTATACAAAAAATGGCCGGAGTCCTTTAAGAACAGTGCAACACCAGTGGGAACCGCAAAAACAGTTATGTGCGGTACGTATCCAGTAATCCACGCTGTTGGACCAAACTTCTCTAATTATTCGGAGTCTGAAGGGGACCGGGAATTGGCAGCTGCCTATCGAGAAGTCGCAAAGGAAGTAACTAGGCTGGGAGTAAATAGTGTAGCTATACCTCTCCTCTCCACAGGTGTATACTCAGGAGGGAAAGACAGGCTGACCCAGTCACTGAACCACCTCTTTACAGCCATGGACTCGACGGATGCAGACGTGGTCATCTACTGCCGCGACAAAGAATGGGAGAAGAAAATATCTGAGGCCATACAGATGCGGACCCAAGTAGAGCTGCTGGATGAGCACATCTCCATAGACTGCGATATTGTTCGCGTGCACCCTGACAGCAGCTTGGCAGGCAGAAAAGGATACAGCACCACGGAAGGCGCACTGTACTCATATCTAGAAGGGACCCGTTTTCATCAGACGGCTGTGGATATGGCGGAGATACATACTATGTGGCCAAAGCAAACAGAGGCCAATGAGCAAGTCTGCCTATATGCCCTGGGGGAAAGTATTGAATCGATCAGGCAGAAATGCCCGGTGGATGATGCAGACGCATCATCTCCCCCCAAAACTGTCCCGTGCCTTTGCCGTTACGCTATGACTCCAGAACGCGTCACCCGGCTTCGCATGAACCACGTCACAAGCATAATTGTGTGTTCTTCGTTTCCCCTCCCAAAGTACAAAATAGAAGGAGTGCAAAAAGTCAAATGCTCTAAGGTAATGCTATTTGACCACAACGTGCCATCGCGCGTAAGTCCAAGGGAATATAGATCTTCCCAGGAGTCTGCACAGGAGGCGAGTACAATCACGTCACTGACGCATAGTCAATTCGACCTAAGCGTTGATGGCGAGATACTGCCCGTCCCGTCAGACCTGGATGCTGACGCCCCAGCCCTAGAACCAGCACTAGACGACGGGGCGACACACACGCTGCCATCCACAACCGGAAACCTTGCGGCCGTGTCCGACTGGGTAATGAGCACCGTACCTGTCGCGCCGCCCAGAAGAAGGCGAGGGAGAAACCTGACTGTGACATGTGACGAGAGAGAAGGGAATATAACACCCATGGCTAGCGTCCGATTCTTTAGGGCAGAGCTGTGTCCGGTCGTACAAGAAACAGCGGAGACGCGTGACACAGCAATGTCTCTTCAGGCACCACCGAGTACCGCCACGGAACCGAATCATCCGCCGATCTCCTTCGGAGCATCAAGCGAGACGTTCCCCATTACATTTGGGGACTTCAACGAAGGAGAAATCGAAAGCTTGTCTTCTGAGCTACTAACTTTCGGAGACTTCTCACCAGGAGAAGTGGATGACTTGACAGACAGCGACTGGTCCACGTGCTCAGACACGGACGACGAGTTATGACTAGACAGGGCAGGTGGGTATATATTCTCGTCGGACACCGGTCCAGGTCATTTACAACAGAAGTCAGTACGCCAGTCAGTGCTGCCGGTGAACACCCTGGAGGAAGTCCACGAGGAGAAGTGTTACCCACCTAAGCTGGATGAAGCAAAGGAGCAACTATTACTTAAGAAACTCCAGGAGAGTGCATCCATGGCCAACAGAAGCAGGTATCAGTCGCGCAAAGTAGAAAACATGAAAGCAGCAATCATCCAGAGACTAAAGAGAGGCTGTAGACTATACTTAATGTCAGAGACCCCAAAAGTCCCTACTTACCGGACTACATATCCGGCGCCTGTGTACTCGCCTCCGATCAACGTCCGATTGTCCAATCCCGAGTCCGCAGTGGCAGCATGCAATGAGTTCTTAGCTAGAAACTATCCAACTGTCTCATCATACCAAATTACCGACGAGTATGATGCATATCTAGACATGGTGGACGGGTCGGAGAGTTGCCTGGACCGAGCGACATTCAATCCGTCAAAACTCAGGAGCTACCCGAAACAGCACGCTTACCACGCGCCCTCCATCAGAAGCGCTGTACCGTCCCCATTCCAGAACACACTACAGAATGTACTGGCAGCAGCCACGAAAAGAAACTGCAACGTCACACAGATGAGGGAATTACCCACTTTGGACTCAGCAGTATTCAACGTGGAGTGTTTCAAAAAATTCGCATGCAACCAAGAATACTGGGAAGAATTTGCTGCCAGCCCTATTAGGATAACAACTGAGAATTTAGCAACCTATGTTACTAAACTAAAAGGGCCAAAAGCAGCAGCGCTATTCGCAAAAACCCATAATCTACTGCCACTACAGGAAGTACCAATGGATAGGTTCACAGTAGATATGAAAAGGGACGTGAAGGTGACTCCTGGTACAAAGCATACAGAGGAAAGACCTAAGGTGCAGGTTATACAGGCGGCTGAACCCTTGGCGACAGCATACCTATGTGGGATTCACAGAGAGCTGGTTAGGAGGCTGAACGCCGTCCTCCTACCCAATGTACATACACTATTTGACATGTCTGCCGAGGATTTCGATGCCATCATAGCCGCACACTTTAAGCCAGGAGACACTGTTTTGGAAACGGACATAGCCTCCTTTGATAAGAGCCAAGATGATTCACTTGCGCTTACTGCTTTGATGCTGTTAGAGGATTTAGGGGTGGATCACTCCCTGCTGGACTTGATAGAGGCTGCTTTCGGAGAGATTTCCAGCTGTCACCTACCGACAGGTACGCGCTTCAAGTTCGGCGCCATGATGAAATCAGGTATGTTCCTAACTCTGTTCGTCAACACATTGTTAAACATCACCATCGCCAGCCGAGTGCTGGAAGATCGTCTGACAAAATCCGCGTGTGCGGCCTTCATCGGCGACGACAACATAATACATGGAGTCGTCTCCGATGAATTGATGGCAGCCAGATGTGCCACTTGGATGAACATGGAAGTGAAGATCATAGATGCAGTTGTATCCTTGAAAGCCCCTTACTTTTGTGGAGGGTTTATACTGCACGATACTGTGACAGGAACAGCTTGCAGAGTGGCAGACCCGCTAAAAAGGCTTTTTAAACTGGGCAAACCGCTAGCGGCAGGTGACGAACAAGATGAAGATAGAAGACGAGCGCTGGCTGACGAAGTGATCAGATGGCAACGAACAGGGCTAATTGATGAGCTGGAGAAAGCGGTATACTCTAGGTACGAAGTGCAGGGTATATCAGTTGTGGTAATGTCCATGGCCACCTTTGCAAGCTCCAGATCCAACTTCGAGAAGCTCAGAGGACCCGTCATAACTTTGTACGGCGGTCCTAAATAGGTACGCACTACAGCTACCTATTTTGCAGAAGCCGACAGCAAGTATCTAAACACTAATCAGCTACAATGGAGTTCATCCCAACCCAAACTTTTTACAATAGGAGGTACCAGCCTCGACCCTGGACTCCGCGCTCTACTATCCAAATCATCAGGCCCAGACCGCGCCCTCAGAGGCAAGCTGGGCAACTTGCCCAGCTGATCTCAGCAGTTAATAAACTGACAATGCGCGCGGTACCCCAACAGAAGCCACGCAGGAATCGGAAGAATAAGAAGCAAAAGCAAAAACAACAGGCGCCACAAAACAACACAAATCAAAAGAAGCAGCCACCTAAAAAGAAACCGGCTCAAAAGAAAAAGAAGCCGGGCCGCAGAGAGAGGATGTGCATGAAAATCGAAAATGATTGTATTTTCGAAGTCAAGCACGAAGGTAAGGTAACAGGTTACGCGTGCCTGGTGGGGGACAAAGTAATGAAACCAGCACACGTAAAGGGGACCATCGATAACGCGGACCTGGCCAAACTGGCCTTTAAGCGGTCATCTAAGTATGACCTTGAATGCGCGCAGATACCCGTGCACATGAAGTCCGACGCTTCGAAGTTCACCCATGAGAAACCGGAGGGGTACTACAACTGGCACCACGGAGCAGTACAGTACTCAGGAGGCCGGTTCACCATCCCTACAGGTGCTGGCAAACCAGGGGACAGCGGCAGACCGATCTTCGACAACAAGGGACGCGTGGTGGCCATAGTCTTAGGAGGAGCTAATGAAGGAGCCCGTACAGCCCTCTCGGTGGTGACCTGGAATAAAGACATTGTCACTAAAATCACCCCCGAGGGGGCCGAAGAGTGGAGTCTTGCCATCCCAGTTATGTGCCTGTTGGCAAACACCACGTTCCCCTGCTCCCAGCCCCCTTGCACGCCCTGCTGCTACGAAAAGGAACCGGAGGAAACCCTACGCATGCTTGAGGACAACGTCATGAGACCTGGGTACTATCAGCTGCTACAAGCATCCTTAACATGTTCTCCCCACCGCCAGCGACGCAGCACCAAGGACAACTTCAATGTCTATAAAGCCACAAGACCATACTTAGCTCACTGTCCCGACTGTGGAGAAGGGCACTCGTGCCATAGTCCCGTAGCACTAGAACGCATCAGAAATGAAGCGACAGACGGGACGCTGAAAATCCAGGTCTCCTTGCAAATCGGAATAAAGACGGATGACAGCCACGATTGGACCAAGCTGCGTCATATGGACAACCACATGCCAGCAGACGCAGAGAGGGCGGGGCTATTTGTAAGAACATCAGCACCGTGTACGATTACTGGAACAATGGGACACTTCATCCTGGCCCGATGTCCAAAAGGGGAAACTCTGACGGTGGGATTCACTGACAGTAGGAAGATTAGTCATTCATGTACGCACCCATTTCACCACGACCCTCCTGTGATAGGTCGGGAAAAATTCCATTCCCGACCGCAGCACGGTAAAGAGCTACCTTGCAGCACGTACGTGCAGAGCACCGCCGCAACTACCGAGGAGATAGAGGTACACATGCCCCCAGACACCCCTGATCGCACATTAATGTCACAACAGTCCGGCAACGTAAAGATCACAGTCAATGGCCAGACGGTGCGGTACAAGTGTAATTGCGGTGGCTCAAATGAAGGACTAACAACTACAGACAAAGTGATTAATAACTGCAAGGTTGATCAATGTCATGCCGCGGTCACCAATCACAAAAAGTGGCAGTATAACTCCCCTCTGGTCCCGCGTAATGCTGAACTTGGGGACCGAAAAGGAAAAATTCACATCCCGTTTCCGCTGGCAAATGTAACATGCAGGGTGCCTAAAGCAAGGAACCCCACCGTGACGTACGGGAAAAACCAAGTCATCATGCTACTGTATCCTGACCACCCAACACTCCTGTCCTACCGGAATATGGGAGAAGAACCAAACTATCAAGAAGAGTGGGTGATGCATAAGAAGGAAGTCGTGCTAACCGTGCCGACTGAAGGGCTCGAGGTCACGTGGGGCAACAACGAGCCGTATAAGTATTGGCCGCAGTTATCTACAAACGGTACAGCCCATGGCCACCCGCATGAGATAATTCTGTATTATTATGAGCTGTACCCTACTATGACTGTAGTAGTTGTGTCAGTGGCCACGTTCATACTCCTGTCGATGGTGGGTATGGCAGCGGGGATGTGCATGTGTGCACGACGCAGATGCATCACACCGTATGAACTGACACCAGGAGCTACCGTCCCTTTCCTGCTTAGCCTAATATGCTGCATCAGAACAGCTAAAGCGGCCACATACCAAGAGGCTGCGATATACCTGTGGAACGAGCAGCAACCTTTGTTTTGGCTACAAGCCCTTATTCCGCTGGCAGCCCTGATTGTTCTATGCAACTGTCTGAGACTCGTACCATGCTGCTGTAAAACGTTGGCTTTTTTAGCCGTAATGAGCGTCGGTGCCCACACTGTGAGCGCGTACGAACACGTAACAGTGATCCCGAACACGGTGGGAGTACCGTATAAGACTCTAGTCAATAGACCTGGCTACAGCCCCATGGTATTGGAGATGGAACTACTGTCAGTCACTTTGGAGCCAACACTATCGCTTGATTACATCACGTGCGAGTACAAAACCGTCATCCCGTCTCCGTACGTGAAGTGCTGCGGTACAGCAGAGTGCAAGGACAAAAACCTACCTGACTACAGCTGTAAGGTCTTCACCGGCGTCTACCCATTTATGTGGGGCGGCGCCTACTGCTTCTGCGACGCTGAAAACACGCAGTTGAGCGAAGCACATGTGGAGAAGTCCGAATCATGCAAAACAGAATTTGCATCAGCATACAGGGCTCATACCGCATCTGCATCAGCTAAGCTCCGCGTCCTTTACCAAGGAAATAACATCACTGTAACTGCCTATGCAAACGGCGACCATGCCGTCACAGTTAAGGACGCCAAATTCATTGTGGGGCCAATGTCTTCAGCCTGGACACCTTTCGACAACAAAATTGTGGTGTACAAAGGTGACGTCTATAACATGGACTACCCGCCCTTTGGCGCAGGAAGACCAGGACAATTTGGCGATATCCAAAGTCGCACACCTGAGAGTAAAGACGTCTATGCTAATACACAACTGGTACTGCAGAGACCGGCTGCGGGTACGGTACACGTGCCATACTCTCAGGCACCATCTGGCTTTAAGTATTGGCTAAAAGAACGCGGGGCGTCACTGCAGCACACAGCACCATTTGGCTGCCAAATAGCAACAAACCCGGTAAGAGCGGTGAACTGCGCCGTAGGGAACATGCCCATCTCCATCGACATACCGGAAGCGGCCTTCACTAGGGTCGTCGACGCGCCCTCTTTAACGGACATGTCGTGCGAGGTACCAGCCTGCACCCATTCCTCAGACTTTGGGGGCGTCGCCATTATTAAATATGCAGCCAGCAAGAAAGGCAAGTGTGCGGTGCATTCGATGACTAACGCCGTCACTATTCGGGAAGCTGAGATAGAAGTTGAAGGGAATTCTCAGCTGCAAATCTCTTTCTCGACGGCCTTAGCCAGCGCCGAATTCCGCGTACAAGTCTGTTCTACACAAGTACACTGTGCAGCTGAGTGCCACCCCCCGAAGGACCACATAGTCAACTACCCGGCGTCACATACCACCCTCGGGGTCCAGGACATCTCCGCTACGGCGATGTCATGGGTGCAGAAGATCACGGGAGGTGTGGGACTGGTTGTTGCTGTTGCCGCACTGATTCTAATCGTGGTGCTATGCGTGTCGTTCAGCAGGCACTAACTTGATAATTAAGTATGAAGGTATATGTGTCCCCTAAGAGACACACTGTATATAGCAAATAATCTATAGATCAAAGGGCTACGCAACCCCTGAATAGTAACAAAATACAAAATCACTAAAAATTATAAAAACAGAAAAATACATAAATAGGTATACGTGTCCCCTAAGAGACACATTGTATGTAGGTGATAAGTATAGATCAAAGGGCCGAATAACCCCTGAATAGTAACAAAATATGAAAATCAATAAAAATCATAAAATAGAAAAACCATAAACAGAAGTAGTTCAAAGGGCTGTAAAACCCCTGAATAGTAACAAAACATAAAATTAATAAAAATCAAATGAATACCATAATTGGCAAACGGAAGAGATGTAGGTACTTAAGCTTCCTAAAAGCAGCCGAACTCACTTTGAGAAGTAGGCATAGCATACCGAACTCTTCCACGATTCTCCGAACCCACAGGGACGTAGGAGATGTTATTTTGTTTTTAATATTTC

>FJ000063|2006|India: Karnataka, Group 4

TTTGGCTGCGTGAGACACAAGTAGCCTACCAGTTTCTTACTGCTCTACTCTGCAAAGCAAGAGATTAATAACCCATCATGGATCCTGTGTACGTGGACATAGACGCTGACAGCGCCTTTTTGAAGGCCCTGCAACGTGCGTACCCCATGTTTGAGGTGGAACCAAGGCAGGTCACACCGAATGACCATGCTAATGCTAGAGCGTTCTCGCATCTAGCTATAAAACTAATAGAGCAGGAAATTGACCCCGACTCAACCATCCTGGATATTGGCAGTGCGCCAGCAAGGAGGATGATGTCGGACAGGAAGTACCACTGCGTCTGCCCGATGCGCAGTGCGGAAGACCCCGAGAGACTCGCTAATTATGCGAGAAAGCTAGCATCTGCCGCAGGAAAAGTCCTGGACAGAAACATTTCTGGAAAGATCGGGGACTTACAAGCAGTAATGGCCGTGCCAGACAAGGAGACGCCAACATTCTGCTTACACACAGACGTCTCATGTAGACAGAGAGCAGACGTCGCTATATACCAAGACGTCTATGCTGTACACGCACCCACGTCGCTATACCACCAGGCGATTAAAGGGGTCCGAGTGGCGTACTGGGTTGGGTTCGACACAACCCCGTTCATGTACAATGCCATGGCGGGTGCCTACCCCTCATACTCGACAAACTGGGCAGATGAGCAGGTACTGAAGGCTAAGAACATAGGATTATGTTCAACAGACCTGACGGAAGGTAGACGAGGCAAGTTGTCTATTATGAGAGGGAAAAAGCTAAAACCGTGCGACCGTGTGCTGTTCTCAGTAGGGTCAACGCTCTACCCGGAAAGCCGCAAGCTACTTAAGAGCTGGCACCTGCCATCGGTGTTCCATTTAAAGGGCAAACTCAGCTTCACATGCCGCTGTGATACAGTGGTTTCGTGTGAGGGCTACGTCGTTAAGAGAATAACGATGAGCCCAGGCCTTTATGGAAAAACCACAGGGTATGCGGTAACCCACCACGCAGACGGATTCCTGATGTGCAAGACTACCGACACGGTTGACGGCGAAAGAGTGTCATTCTCGGTGTGCACATACGTGCCGGCGACCATTTGTGATCAAATGACCGGCATCCTTGCTACAGAAGTCACGCCGGAGGATGCACAGAAGCTGTTGGTGGGGCTGAACCAGAGAATAGTGGTTAACGGCAGAACGCAACGGAATATGAACACCATGAAAAATTATCTGCTTCCCGTGGTCGCCCAAGCCTTCAGTAAGTGGGCAAAGGAGTGCCGGAAAGACATGGAAGATGAAAAACTCCTGGGGGTCAGAGAAAGAACACTGACCTGCTGCTGTCTATGGGCATTCAAGAAGCAGAAAACACACACGGTCTACAAGAGGCCTGATACCCAGTCAATTCAGAAGGTTCAGGCCGAGTTTGACAGCTTTGTGGTACCGAGTCTGTGGTCGTCCGGGTTGTCAATCCCTTTGAGGACTAGAATCAAATGGTTGTTAAGCAAGGTGCCAAAAACCGACCTGATCCCATACAGCGGAGACGCCCGAGAAGCCCGGGACGCAGAAAAAGAAGCAGAGGAAGAACGAGAAGCAGAACTGACTCGCGAAGCCCTACCACCTCTACAGGCAGCACAGGAAGATGTTCAGGTCGAAATCGACGTGGAACAGCTTGAGGACAGAGCGGGCGCAGGAATAATAGAGACTCCGAGAGGAGCTATCAAAGTTACTGCCCAACCAACAGACCACGTCGTGGGAGAGTACCTGGTACTCTCCCCGCAGACCGTACTACGTAGCCAGAAGCTCAGTCTGATTCACGCTTTGGCGGAGCAAGTGAAGACGTGCACGCACAACGGACGAGCAGGGAGGTATGCGGTCGAAGCGTACGATGGCCGAGTCCTAGTGCCCTCAGGCTATGCAATCTCGCCTGAAGACTTCCAGAGTCTAAGCGAAAGCGCAACGATGGTGTATAACGAAAGAGAGTTCGTAAACAGAAAGCTACACCATATTGCGATGCACGGACCAGCCCTGAACACCGACGAAGAGTCGTATGAGCTGGTGAGGGCAGAGAGGACAGAACACGAGTACGTCTACGACGTGGATCAGAGAAGATGCTGTAAGAAGGAAGAAGCCGCAGGACTGGTACTGGTGGGCGACTTGACTAATCCGCCCTACCACGAATTCGCATATGAAGGGCTAAAAATCCGCCCTGCCTGCCCATACAAAATTGCAGTCATAGGAGTCTTCGGAGTACCGGGATCTGGCAAGTCAGCTATTATCAAGAACCTAGTTACCAGGCAGGACCTGGTGACTAGCGGAAAGAAAGAAAACTGCCAAGAAATCACCACCGACGTGATGAGACAGAGAGGTCTAGAGATATCTGCACGTACGGTTGACTCGCTGCTCTTGAATGGATGCAACAGACCAGTCGACGTGTTGTACGTAGACGAGGCGTTTGCGTGCCACTCTGGAACGCTACTTGCTTTGATCGCCTTGGTGAGACCAAGGCAGAAAGTTGTACTTTGTGGTGACCCGAAGCAGTGCGGCTTCTTCAATATGATGCAGATGAAAGTCAACTATAATCACAACATCTGCACCCAAGTGTACCACAAAAGTATCTCCAGGCGGTGTACACTGCCTGTGACCGCCATTGTGTCATCGTTGCATTACGAAGGCAAAATGCGCACTACGAATGAGTACAACAAGCCGATTGTAGTGGACACTACAGGCTCAACAAAACCTGACCCTGGAGACCTCGTGTTAACGTGCTTCAGAGGGTGGGTTAAACAACTGCAAATTGACTATCGTGGATACGAGGTCATGACAGCAGCCGCATCCCAAGGGTTAACCAGAAAAGGAGTTTACGCAGTTAGACAAAAAGTTAATGAAAACCCGCTCTATGCATCAACGTCAGAGCACGTCAACGTACTCCTAACGCGTACGGAAGGTAAACTGGTATGGAAGACACTTTCCGGCGACCCGTGGATAAAGACGCTGCAGAACCCACCGAAAGGAAACTTCAAAGCAACTATTAAGGAGTGGGAGGTGGAGCATGCATCAATAATGGCGGGCATCTGCAGTCACCAAATGACCTTCGATACATTCCAAAATAAAGCCAACGTTTGTTGGGCTAAGAGCTTGGTCCCTATCCTCGAAACAGCGGGGATAAAACTAAATGATAGGCAGTGGTCTCAGATAATTCAAGCCTTCAAAGAAGACAAAGCATACTCACCTGAAGTAGCCCTGAATGAAATATGTACGCGCATGTATGGGGTGGATCTAGACAGCGGGCTATTTTCTAAACCGTTGGTGTCTGTGTATTACGCGGATAACCACTGGGATAATAGGCCTGGAGGGAAAATGTTCGGATTTAACCCCGAGGCAGCATCCATTCTAGAAAGAAAGTATCCATTCACAAAAGGGAAGTGGAACATCAACAAGCAGATCTGCGTGACTACCAGGAGGATAGAAGACTTTAACCCTACCACCAACATCATACCGGCCAACAGGAGACTACCACACTCATTAGTGGCCGAACACCGCCCAGTAAAAGGGGAAAGAATGGAATGGCTGGTTAACAAGATAAACGGCCACCACGTGCTCCTGGTCAGTGGCTATAACCTTGCACTGCCTACTAAGAGAGTCACTTGGGTAGCGCCGTTAGGTGTCCGCGGAGCGGACTACACATACAACCTAGAGTTGGGTCTGCCAGCAACGCTTGGTAGGTATGACCTTGTGGTCATAAACATCCACACACCTTTTCGCATACACCATTACCAACAGTGCGCCGACCACGCAATGAAACTGCAAATGCTCGGGGGTGACTCATTGAGACTGCTCAAACCGGGCGGCTCTCTATTGATCAGAGCATATGGTTACGCAGATAGAACCAGTGAACGAGTCATCTGCGTATTGGGACGCAAGTTCAGATCGTCTAGAGCGTTGAAACCACCATGTGTCACCAGCAACACTGAGATGTTTTTCCTATTCAGCAACTTTGACAATGGCAGAAGGAATTTCACAACTCATGTCATGAACAATCAACTGAATGCAGCCTTCGTAGGACAGGTCACCCGAGCAGGATGTGCACCGTCGTACCGGGTAAAACGCATGGACATCGCGAAGAACGATGAAGAGTGCGTAGTCAACGCCGCTAACCCTCGCGGGTTACCGGGTGACGGTGTTTGCAAGGCAGTATACAAAAAATGGCCGGAGTCCTTTAAGAACAGTGCAACACCAGTGGGAACCGCAAAAACAGTTATGTGCGGTACGTATCCAGTAATCCACGCTGTTGGACCAAACTTCTCTAATTATTCGGAGTCTGAAGGGGACCGGGAATTGGCAGCTGCCTATCGAGAAGTCGCAAAGGAAGTAACTAGGCTGGGAGTAAATAGTGTAGCTATACCTCTCCTCTCCACAGGTGTATACTCAGGAGGGAAAGACAGGCTGACCCAGTCACTGAACCACCTCTTTACAGCCATGGACTCGACGGATGCAGACGTGGTCATCTACTGCCGCGACAAAGAATGGGAGAAGAAAATATCTGAGGCCATACAGATGCGGACCCAAGTAGAGCTGCTGGATGAGCACATCTCCATAGACTGCGATATTGTTCGCGTGCACCCTGACAGCAGCTTGGCAGGCAGAAAAGGATACAGCACCACGGAAGGCGCACTGTACTCATATCTAGAAGGGACCCGTTTTCATCAGACGGCTGTGGATATGGCGGAGATACATACTATGTGGCCAAAGCAAACAGAGGCCAATGAGCAAGTCTGCCTATATGCCCTGGGGGAAAGTATTGAATCGATCAGGCAGAAATGCCCGGTGGATGATGCAGACGCATCATCTCCCCCCAAAACTGTCCCGTGCCTTTGCCGTTACGCTATGACTCCAGAACGCGTCACCCGGCTTCGCATGAACCACGTCACAAGCATAATTGTGTGTTCTTCGTTTCCCCTCCCAAAGTACAAAATAGAAGGAGTGCAAAAAGTCAAATGCTCTAAGGTAATGCTATTTGACCATAACGTGCCATCGCGCGTAAGTCCAAGGGAATATAGATCTTCCCAGGAGTCTGCACAGGAGGCGAGTACAATCACGTCACTGACGCATAGTCAATTCGACCTAAGCGTTGATGGCGAGATACTGCCCGTCCCGTCAGACCTGGATGCTGACGCCCCAGCCCTAGAACCAGCACTAGACGACGGGGCGACACACACGCTGCCATCCACGACCGGAAACCTTGCGGCCGTGTCTGACTGGGTAATGAGCACCGTACCTGTCGCGCCGCCCAGAAGAAGGCGAGGGAGAAACCTGACTGTGACATGTGACGAGAGAGAAGGGAATATAACACCCATGGCTAGCGTCCGATTCTTTAGGGCAGAGCTGTGTCCGGTCGTACAAGAAACAGCGGAGACGCGTGACACAGCAATGTCTCTTCAGGCACCACCGAGTACCGCCACGGAACCGAATCATCCGCCGATCTCCTTCGGAGCATCAAGCGCGACGTTCCCCATTACATTTGGGGACTTCAACGAAGGAGAAATCGAAAGCTTGTCTTCTGAGCTACTAACTTTCGGAGACTTCTTACCAGGAGAAGTGGATGACTTGACAGACAGCGACTGGTCCACGTGCTCAGACACGGACGACGAGTTATGACTAGACAGGGCAGGTGGGTATATATTCTCGTCGGACACCGGTCCAGGTCATTTACAACAGAAGTCAGTACGCCAGTCAGTGCTGCCGGTGAACACCCTGGAGGAAGTCCACGAGGAGAAGTGTTACCCACCTAAGCTGGATGAAGCAAAGGAGCAACTATTACTTAAGAAACTCCAGGAGAGTGCATCCATGGCCAACAGAAGCAGGTATCAGTCGCGCAAAGTAGAAAACATGAAAGCAGCAATCATCCAGAGACTAAAGAGAGGCTGTAGACTATACTTAATGTCAGAGACCCCAAAAGTCCCTACTTACCGGACTACATATCCGGCGCCTGTGTACTCGCCTCCGATCAACGTCCGATTGTCCAATCCCGAGTCCGCAGTGGCAGCATGCAATGAGTTCTTAGCTAGAAACTATCCAACTGTCTCATCATACCAAATTACCGACGAGTATGATGCATATCTAGACATGGTGGACGGGTCGGAGAGTTGCCTGGACCGAGCGACATTCAATCCGTCAAAACTCAGGAGCTACCCGAAACAGCACGCTTACCACGCGCCCTCCATCAGAAGCGCTGTACCGTCCCCATTCCAGAACACACTACAGAATGTACTGGCAGCAGCCACGAAAAGAAACTGCAACGTCACACAGATGAGGGAATTACCCACTTTGGACTCAGCAGTATTCAACGTGGAGTGTTTCAAAAAATTCGCATGCAACCAAGAATACTGGGAAGAATTTGCTGCCAGCCCTATTAGGATAACAACTGAGAATTTAGCAACCTATGTTACTAAACTAAAAGGGCCAAAAGCAGCAGCGCTATTCGCAAAAACCCATAATCTACTGCCACTACAGGAAGTACCAATGGATAGGTTCACAGTAGATATGAAAAGGGACGTGAAGGTGACTCCTGGTACAAAGCATACAGAGGAAAGACCTAAGGTGCAGGTTATACAGGCGGCTGAACCCTTGGCGACAGCATACCTATGTGGGATTCACAGAGAGCTGGTTAGGAGGCTGAACGCCGTCCTCCTACCCAATGTACATACACTATTTGACATGTCTGCCGAGGATTTCGATGCCATCATAGCCGCACACTTTAAGCCAGGAGACACTGTTTTGGAAACGGACATAGCCTCCTTTGATAAGAGCCAAGATGATTCACTTGCGCTTACTGCTTTGATGCTGTTAGAGGATTTAGGGGTGGATCACTCCCTGCTGGACTTGATAGAGGCTGCTTTCGGAGAGATTTCCAGCTGTCACCTACCGACAGGTACGCGCTTCAAGTTCGGCGCCATGATGAAATCAGGTATGTTCCTAACTCTGTTCGTCAACACATTGTTAAACATCACCATCGCCAGCCGAGTGCTGGAAGATCGTCTGACAAAATCCGCGTGCGCGGCCTTCATCGGCGACGACAACATAATACATGGAGTCGTCTCCGATGAATTGATGGCAGCCAGATGTGCCACTTGGATGAACATGGAAGTGAAGATCATAGATGCAGTTGTATCCTTGAAAGCCCCTTACTTTTGTGGAGGGTTTATACTGCACGATACTGTGACAGGAACAGCTTGCAGAGTGGCAGACCCGCTAAAAAGGCTTTTTAAACTGGGCAAACCGCTAGCGGCAGGTGACGAACAAGATGAAGATAGAAGACGAGCGCTGGCTGACGAAGTGATCAGATGGCAACGAACAGGGCTAATTGATGAGCTGGAGAAAGCGGTATACTCTAGGTACGAAGTGCAGGGTATATCAGTTGTGGTAATGTCCATGGCCACCTTTGCAAGCTCCAGATCCAACTTCGAGAAGCTCAGAGGACCCGTCATAACTTTGTACGGCGGTCCTAAATAGGTACGCACTACAGCTACCTATTTTGCAGAAGCCGACAGCAAGTATCTAAACACTAATCAGCTACAATGGAGTTCATCCCAACCCAAACTTTTTACAATAGGAGGTACCAGCCTCGACCCTGGACTCCGCGCTCTACTATCCAAATCATCAGGCCCAGACCGCGCCCTCAGAGGCAAGCTGGGCAACTTGCCCAGCTGATCTCAGCAGTTAATAAACTGACAATGCGCGCGGTACCCCAACAGAAGCCACGCAGGAATCGGAAGAATAAGAAGCAAAAGCAAAAACAACAGGCGCCACAAAACAACACAAATCAAAAGAAGCAGCCACCTAAAAAGAAACCGGCTCAAAAGAAAAAGAAGCCGGGCCGCAGAGAGAGGATGTGCATGAAAATCGAAAATGATTGTATTTTCGAAGTCAAGCACGAAGGTAAGGTAACAGGTTACGCGTGCCTGGTGGGGGACAAAGTAATGAAACCAGCACACGTAAAGGGGACCATCGATAACGCGGACCTGGCCAAACTGGCCTTTAAGCGGTCATCTAAGTATGACCTTGAATGCGCGCAGATACCCGTGCACATGAAGTCCGACGCTTCGAAGTTCACCCATGAGAAACCGGAGGGGTACTACAACTGGCACCACGGAGCAGTACAGTACTCAGGAGGCCGGTTCACCATCCCTACAGGTGCTGGCAAACCAGGGGACAGCGGCAGACCGATCTTCGACAACAAGGGACGCGTGGTGGCCATAGTCTTAGGAGGAGCTAATGAAGGAGCCCGTACAGCCCTCTCGGTGGTGACCTGGAATAAAGACATTGTCACTAAAATCACCCCCGAGGGGGCCGAAGAGTGGAGTCTTGCCATCCCAGTTATGTGCCTGTTGGCAAACACCACGTTCCCCTGCTCCCAGCCCCCTTGCACGCCCTGCTGCTACGAAAAGGAACCGGAGGAAACCCTACGCATGCTTGAGGACAACGTCATGAGACCTGGGTACTATCAGCTGCTACAAGCATCCTTAACATGTTCTCCCCACCGCCAGCGACGCAGCACCAAGGACAACTTCAATGTCTATAAAGCCACAAGACCATACTTAGCTCACTGTCCCGACTGTGGAGAAGGGCACTCGTGCCATAGTCCCGTAGCACTAGAACGCATCAGAAATGAAGCGACAGACGGGACGCTGAAAATCCAGGTCTCCTTGCAAATCGGAATAAAGACGGATGACAGCCACGATTGGACCAAGCTGCGTTATATGGACAACCACATGCCAGCAGACGCAGAGAGGGCGGGGCTATTTGTAAGAACATCAGCACCGTGTACGATTACTGGAACAATGGGACACTTCATCCTGGCCCGATGTCCAAAAGGGGAAACTCTGACGGTGGGATTCACTGACAGTAGGAAGATTAGTCATTCATGTACGCACCCATTTCACCACGACCCTCCTGTGATAGGTCGGGAAAAATTCTATTCCCGACCGCAGCACGGTAAAGAGCTACCTTGCAGCACGTACGTGCAGAGCACCGCCGCAACTACCGAGGAGATAGAGGTACACATGCCCCCAGACACCCCTGATCGCACATTAATGTCACAACAGTCCGGCAACGTAAAGATCACAGTCAATGGCCAGACGGTGCGGTACAAGTGTAATTGCGGTGGCTCAAATGAAGGACTAACAACTACAGACAAAGTGATTAATAACTGCAAGGTTGATCAATGTCATGCCGCGGTCACCAATCACAAAAAGTGGCAGTATAACTCCCCTCTGGTCCCGCGTAATGCTGAACTTGGGGACCGAAAAGGAAAAATTCACATCCCGTTTCCGCTGGCAAATGTAACATGCAGGGTGCCTAAAGCAAGGAACCCCACCGTGACGTACGGGAAAAACCAAGTCATCATGCTACTGTATCCTGACCACCCAACACTCCTGTCCTACCGGAATATGGGAGAAGAACCAAACTATCAAGAAGAGTGGGTGATGCATAAGAAGGAAGTCGTGCTAACCGTGCCGACTGAAGGGCTCGAGGTCACGTGGGGCAACAACGAGCCGTATAAGTATTGGCCGCAGTTATCTACAAACGGTACAGCCCATGGCCACCCGCATGAGATAATTCTGTATTATTATGAGCTGTACCCTACTATGACTGTAGTAGTTGTGTCAGTGGCCACGTTCATACTCCTGTCGATGGTGGGTATGGCAGCGGGGATGTGCATGTGTGCACGACGCAGATGCATCACACCGTATGAACTGACACCAGGAGCTACCGTCCCTTTCCTGCTTAGCCTAATATGCTGCATCAGAACAGCTAAAGCGGCCACATACCAAGAGGCTGCGATATACCTGTGGAACGAGCAGCAACCTTTGTTTTGGCTACAAGCCCTTATTCCGCTGGCAGCCCTGATTGTTCTATGCAACTGTCTGAGACTCTTACCATGCTGCTGTAAAACGTTGGCTTTTTTAGCCGTAATGAGCGTCGGTGCCCACACTGTGAGCGCGTACGAACACGTAACAGTGATCCCGAACACGGTGGGAGTACCGTATAAGACTCTAGTCAATAGACCTGGCTACAGCCCCATGGTATTGGAGATGGAACTACTGTCAGTCACTTTGGAGCCAACACTATCGCTTGATTACATCACGTGCGAGTACAAAACCGTCATCCCGTCCCCGTACGTGAAGTGCTGCGGTACAGCAGAGTGCAAGGACAAAAACCTACCTGACTACAGCTGTAAGGTCTTCACCGGCGTCTACCCATTTATGTGGGGCGGCGCCTACTGCTTCTGCGACGCTGAAAACACGCAGTTGAGCGAAGCACATGTGGAGAAGTCCGAATCATGCAAAACAGAATTTGCATCAGCATACAGGGCTCATACCGCATCTGCATCAGCTAAGCTCCGCGTCCTTTACCAAGGAAATAACATCACTGTAACTGCCTATGCAAACGGCGACCATGCCGTCACAGTTAAGGACGCCAAATTCATTGTGGGGCCAATGTCTTCAGCCTGGACACCTTTCGACAACAAAATTGTGGTGTACAAAGGTGACGTCTATAACATGGACTACCCGCCCTTTGGCGCAGGAAGACCAGGACAATTTGGCGATATCCAAAGTCGCACACCTGAGAGTAGAGACGTCTATGCTAATACACAACTGGTACTGCAGAGACCGGCTGCGGGTACGGTACACGTGCCATACTCTCAGGCACCATCTGGCTTTAAGTATTGGCTAAAAGAACGCGGGGCGTCACTGCAGCACACAGCACCATTTGGCTGCCAAATAGCAACAAACCCGGTAAGAGCGGTGAACTGCGCCGTAGGGAACATGCCCATCTCCATCGACATACCGGAAGCGGCCTTCACTAGGGTCGTCGACGCGCCCTCTTTAACGGACATGTCGTGCGAGGTACCAGCCTGCACCCATTCCTCAGACTTTGGGGGCGTCGCCATTATTAAATATGCAGCCAGCAAGAAAGGCAAGTGTGCGGTGCATTCGATGACTAACGCCGTCACTATTCGGGAAGCTGAGATAGAAGTTGAAGGGAATTCTCAGCTGCAAATCTCTTTCTCGACGGCCTTAGCCAGCGCCGAATTCCGCGTACAAGTCTGTTCTACACAAGTACACTGTGCAGCTGAGTGCCACCCCCCGAAGGACCACATAGTCAACTACCCGGCGTCACATACCACCCTCGGGGTCCAGGACATCTCCGCTACGGCGATGTCATGGGTGCAGAAGATCACGGGAGGTGTGGGACTGGTTGTTGCTGTTGCCGCACTGATTCTAATCGTGGTGCTATGCGTGTCGTTCAGCAGGCACTAACTTGACAATTAAGTATGAAGGTATATGTGTCCCCTAAGAGACACACTGTACATAGCAAATAATCTATAGATCAAAGGGCTACGCAACCCCTGAATAGTAACAAAATACAAAATCACTAAAAATTATAAAAACAGAAAAATACATAAATAGGTATACGTGTCCCCTAAGAGACACATTGTATGTAGGTGATAAGTATAGATCAAAGGGCCGAATAACCCCTGAATAGTAACAAAATATGAAAATCAATAAAAATCATAAAATAGAAAAACCATAAACAGAAGTAGTTCAAAGGGCTATAAAACCCCTGAATAGTAACAAAACATAAAGTTAATAAAAATCAAATGAATACCATAATTGGCAAACGGAAGAGATGTAGGTACTTAAGCTTCCTAAAAGCAGCCGAACTCACTTTGAGAAGTAGGCATAGCATACCGAACTCTTCCACGATTCTCCGAACCCACAGGGACGTAGGAGATGTTATTTTGTTTTTAATATTTC

>FJ000064|2006|India: Gujarat, Group 4

CTGCGTGAGACACAAGTAGCCTACCAGTTTCTTACTGCTCTACTCTGCAAAGCAAGAGATTAATAACCCATCATGGATCCTGTGTACGTGGACATAGACGCTGACAGCGCCTTTTTGAAGGCCCTGCAACGTGCGTACCCCATGTTTGAGGTGGAACCAAGGCAGGTCACACCGAATGACCATGCTAATGCTAGAGCGTTCTCGCATCTAGCTATAAAACTAATAGAGCAGGAAATTGACCCCGACTCAACCATCCTGGATATTGGCAGTGCGCCAGCAAGGAGGATGATGTCGGACAGGAAGTACCACTGCGTCTGCCCGATGCGCAGTGCGGAAGACCCCGAGAGACTCGCTAATTATGCGAGAAAGCTAGCATCTGCCGCAGGAAAAGTCCTGGACAGAAACATCTCTGGAAAGATCGGGGACTTACAAGCAGTAATGGCCGTGCCAGACAAGGAGACGCCAACATTCTGCTTACACACAGACGTCTCATGTAGACAGAGAGCAGACGTCGCTATATACCAAGACGTCTATGCTGTACACGCACCCACGTCGCTATACCACCAGGCGATTAAAGGGGTCCGAGTGGCGTACTGGGTTGGGTTCGACACAACCCCGTTCATGTACAATGCCATGGCGGGTGCCTACCCCTCATACTCGACAAACTGGGCAGATGAGCAGGTACTGAAGGCTAAGAACATAGGATTATGTTCAACAGACCTGACGGAAGGTAGACGAGGCAAGTTGTCTATTATGAGAGGGAAAAAGCTAAAACCGTGCGACCGTGTGCTGTTCTCAGTAGGGTCAACGCTCTACCCGGAAAGCCGCAAGCTACTTAAGAGCTGGCACCTGCCATCGGTGTTCCATTTAAAGGGCAAACTCAGCTTCACATGCCGCTGTGATACAGTGGTTTCGTGTGAGGGCTACGTCGTTAAGAGAATAACGATGAGCCCAGGCCTTTATGGAAAAACCACAGGGTATGCGGTAACCCACCACGCAGACGGATTCCTGATGTGCAAGACTACCGACACGGTTGACGGCGAAAGAGTGTCATTCTCGGTGTGCACATACGTGCCGGCGACCATTTGTGATCAAATGACCGGCATCCTTGCTACAGAAGTCACGCCGGAGGATGCACAGAAGCTGTTGGTGGGGCTGAACCAGAGAATAGTGGTTAACGGCAGAACGCAACGGAATATGAACACCATGAAAAATTATCTGCTTCCCGTGGTCGCCCAAGCCTTCAGTAAGTGGGCAAAGGAGTGCCGGAAAGACATGGAAGATGAAAAACTCCTGGGGGTCAGAGAAAGAACACTGACCTGCTGCTGTCTATGGGCATTCAAGAAGCAGAAAACACACACGGTCTACAAGAGGCCTGATACCCAGTCAATTCAGAAGGTTCAGGCCGAGTTTGACAGCTTTGTGGTACCGAGTCTGTGGTCGTCCGGGTTGTCAATCCCTTTGAGGACTAGAATCAAATGGTTGTTAAGCAAGGTGCCAAAAACCGACCTGATCCCATACAGCGGAGACGCCCGAGAAGCCCGGGACGCAGAAAAAGAAGCAGAGGAAGAACGAGAAGCAGAACTGACTCGCGAAGCCCTACCACCTCTACAGGCAGCACAGGAAGATGTTCAGGTCGAAATCGACGTGGAACAGCTTGAGGACAGAGCGGGCGCAGGAATAATAGAGACTCCGAGAGGAGCTATCAAAGTTACTGCCCAACCAACAGACCACGTCGTGGGAGAGTACCTGGTACTCTCCCCGCAGACCGTACTACGTAGCCAGAAGCTCAGTCTGATTCACGCTTTGGCGGAGCAAGTGAAGACGTGCACGCACAACGGACGAGCAGGGAGGTATGCGGTCGAAGCGTACGACGGCCGAGTCCTAGTGCCCTCAGGCTATGCAATCTCGCCTGAAGACTTCCAGAGTCTAAGCGAAAGCGCAACGATGGTGTATAACGAAAGAGAGTTCGTAAACAGAAAGCTACACCATATTGCGATGCACGGACCAGCCCTGAACACCGACGAAGAGTCGTATGAGCTGGTGAGGGCAGAGAGGACAGAACACGAGTACGTCTACGACGTGGATCAGAGAAGATGCTGTAAGAAGGAAGAAGCCGCAGGACTGGTACTGGTGGGCGACTTGACTAATCCGCCCTACCACGAATTCGCATATGAAGGGCTAAAAATCCGCCCTGCCTGCCCATACAAAATTGCAGTCATAGGAGTCTTCGGAGTACCGGGATCTGGCAAGTCAGCTATTATCAAGAACCTAGTTACCAGGCAGGACCTGGTGACTAGCGGAAAGAAAGAAAACTGCCAAGAAATCACCACCGACGTGATGAGACAGAGAGGTCTAGAGATATCTGCACGTACGGTTGACTCGCTGCTCTTGAATGGATGCAACAGACCAGTCGACGTGTTGTACGTAGACGAGGCGTTTGCGTGCCACTCTGGAACGCTACTTGCTTTGATCGCCTTGGTGAGACCAAGGCAGAAAGTTGTACTTTGTGGTGACCCGAAGCAGTGCGGCTTCTTCAATATGATGCAGATGAAAGTCAACTATAATCACAACATCTGCACCCAAGTGTACCACAAAAGTATCTCCAGGCGGTGTACACTGCCTGTGACCGCCATTGTGTCATCGTTGCATTACGAAGGCAAAATGCGCACTACGAATGAGTACAACAAGCCGATTGTAGTGGACACTACAGGCTCAACAAAACCCGACCCTGGAGACCTCGTGTTAACGTGCTTCAGAGGGTGGGTTAAACAACTGCAAATTGACTATCGTGGATACGAGGTCATGACAGCAGCCGCATCCCAAGGGTTAACCAGAAAAGGAGTTTACGCAGTTAGACAAAAAGTTAATGAAAACCCGCTCTATGCATCAACGTCAGAGCACGTCAACGTACTCCTAACGCGTACGGAAGGTAAACTGGTATGGAAGACACTTTCCGGCGACCCGTGGATAAAGACGCTGCAGAACCCACCGAAAGGAAACTTCAAAGCAACTATTAAGGAGTGGGAGGTGGAGCATGCATCAATAATGGCGGGCATCTGCAGTCACCAAATGACCTTCGATACATTCCAAAATAAAGCCAACGTTTGTTGGGCTAAGAGCTTGGTCCCTATCCTCGAAACAGCGGGGATAAAACTAAATGATAGGCAGTGGTCTCAGATAATTCAAGCCTTCAAAGAAGATAAAGCATACTCACCTGAAGTAGCCCTGAATGAAATATGTACGCGCATGTATGGGGTGGATCTAGACAGCGGGCTATTTTCTAAACCGTTGGTGTCTGTGTATTACGCGGATAACCACTGGGATAATAGGCCTGGAGGGAAAATGTTCGGATTTAACCCCGAGGCAGCATCCATTCTAGAAAGAAAGTATCCATTCACAAAAGGGAAGTGGAACATCAACAAGCAGATCTGCGTGACTACCAGGAGGATAGAAGACTTTAACCCTACCACCAACATCATACCGGCCAACAGGAGACTACCACACTCATTAGTGGCCGAACACCGCCCAGTAAAAGGGGAAAGAATGGAATGGCTGGTTAACAAGATAAACGGCCACCACGTGCTCCTGGTCAGTGGCTATAACCTTGCACTGCCTACTAAGAGAGTCACTTGGGTAGCGCCGTTAGGTGTCCGCGGAGCGGACTACACATACAACCTAGAGTTGGGTCTGCCAGCAACGCTTGGTAGGTATGACCTTGTGGTCATAAACATCCACACACCTTTTCGCATACACCATTACCAACAGTGCGTCGACCACGCAATGAAACTGCAAATGCTCGGGGGTGACTCATTGAGACTGCTCAAACCGGGCGGCTCTCTATTGATCAGAGCATATGGTTACGCAGATAGAACCAGTGAACGAGTCATCTGCGTATTGGGACGCAAGTTTAGATCGTCTAGAGCGTTGAAACCACCATGTGTCACCAGCAACACTGAGATGTTTTTCCTATTCAGCAACTTTGACAATGGCAGAAGGAATTTCACAACTCATGTCATGAACAATCAACTGAATGCAGCCTTCGTAGGACAGGTCACCCGAGCAGGATGTGCACCGTCGTACCGGGTAAAACGCATGGACATCGCGAAGAACGATGAAGAGTGCGTAGTCAACGCCGCTAACCCTCGCGGGTTACCGGGTGACGGTGTTTGCAAGGCAGTATACAAAAAATGGCCGGAGTCCTTTAAGAACAGTGCAACACCAGTGGGAACCGCAAAAACAGTTATGTGCGGTACGTATCCAGTAATCCACGCTGTTGGACCAAACTTCTCTAATTATTCGGAGTCTGAAGGGGACCGGGAATTGGCAGCTGCCTATCGAGAAGTCGCAAAGGAAGTAACTAGGCTGGGAGTAAATAGTGTAGCTATACCTCTCCTCTCCACAGGTGTATACTCAGGAGGGAAAGACAGGCTGACCCAGTCACTGAACCACCTCTTTACAGCCATGGACTCGACGGATGCAGACGTGGTCATCTACTGCCGCGACAAAGAATGGGAGAAGAAAATATCTGAGGCCATACAGATGCGGACCCAAGTAGAGCTGCTGGATGAGCACATCTCCATAGACTGCGATATTGTTCGCGTGCACCCTGACAGCAGCTTGGCAGGCAGAAAAGGATACAGCACCACGGAAGGCGCACTGTACTCATATCTAGAAGGGACCCGTTTTCATCAGACGGCTGTGGATATGGCGGAGATACATACTATGTGGCCAAAGCAAACAGAGGCCAATGAGCAAGTCTGCCTATATGCCCTGGGGGAAAGTATTGAATCGATCAGGCAGAAATGCCCGGTGGATGATGCAGACGCATCATCTCCCCCCAAAACTGTCCCGTGCCTTTGCCGTTACGCTATGACTCCAGAACGCGTCACCCGGCTTCGCATGAACCACGTCACAAGCATAATTGTGTGTTCTTCGTTTCCCCTCCCAAAGTACAAAATAGAAGGAGTGCAAAAAGTCAAATGCTCTAAGGTAATGCTATTTGACCACAACGTGCCATCGCGCGTAAGTCCAAGGGAATATAGATCTTCCCAGGAGTCTGCACAGGAGGCGAGTACAATCACGTCACTGACGCATAGTCAATTCGACCTAAGCGTTGATGGCGAGATACTGCCCGTCCCGTCAGACCTGGATGCTGACGCCCCAGCCCTAGAACCAGCACTAGACGACGGGGCGACACACACGCTGCCATCCACAACCGGAAACCTTGCGGCCGTGTCTGACTGGGTAATGAGCACCGTACCTGTCGCGCCGCCCAGAAGAAGGCGAGGGAGAAACCTGACTGTGACATGTGACGAGAGAGAAGGGAATATAACACCCATGGCTAGCGTCCGATTCTTTAGGGCAGAGCTGTGTCCGGTCGTACAAGAAACAGCGGAGACGCGTGACACAGCAATGTCTCTTCAGGCACCACCGAGTACCGCCACGGAACCGAATCATCCGCCGATCTCCTTCGGAGCATCAAGCGAGACGTTCCCCATTACATTTGGGGACTTCAACGAAGGAGAAATCGAAAGCTTGTCTTCTGAGCTACTAACTTTCGGAGACTTCTTACCAGGAGAAGTGGATGACTTGACAGACAGCGACTGGTCCACGTGCTCAGACACGGACGACGAGTTATGACTAGACAGGGCAGGTGGGTATATATTCTCGTCGGACACCGGTCCAGGTCATTTACAACAGAAGTCAGTACGCCAGTCAGTGCTGCCGGTGAACACCCTGGAGGAAGTCCACGAGGAGAAGTGTTACCCACCTAAGCTGGATGAAGCAAAGGAGCAACTATTACTTAAGAAACTCCAGGAGAGTGCATCCATGGCCAACAGAAGCAGGTATCAGTCGCGCAAAGTAGAAAACATGAAAGCAGCAATCATCCAGAGACTAAAGAGAGGCTGTAGACTATACTTAATGTCAGAGACCCCAAAAGTCCCTACTTACCGGACTACATATCCGGCGCCTGTGTACTCGCCTCCGATCAACGTCCGATTGTCCAATCCCGAGTCCGCAGTGGCAGCATGCAATGAGTTCTTAGCTAGAAACTATCCAACTGTCTCATCATACCAAATTACCGACGAGTATGATGCATATCTAGACATGGTGGACGGGTCGGAGAGTTGCCTGGACCGAGCGACATTCAATCCGTCAAAACTCAGGAGCTACCCGAAACAGCACGCTTACCACGCGCCCTCCATCAGAAGCGCTGTACCGTCCCCATTCCAGAACACACTACAGAATGTACTGGCAGCAGCCACGAAAAGAAACTGCAACGTCACACAGATGAGGGAATTACCCACTTTGGACTCAGCAGTATTCAACGTGGAGTGTTTCAAAAAATTCGCATGCAACCAAGAATACTGGGAAGAATTTGCTGCCAGCCCTATTAGGATAACAACTGAGAATTTAGCAACCTATGTTACTAAACTAAAAGGGCCAAAAGCAGCAGCGCTATTCGCAAAAACCCATAATCTACTGCCACTACAGGAAGTACCAATGGATAGGTTCACAGTAGATATGAAAAGGGACGTGAAGGTGACTCCTGGTACAAAGCATACAGAGGAAAGACCTAAGGTGCAGGTTATACAGGCGGCTGAACCCTTGGCGACAGCATACCTATGTGGGATTCACAGAGAGCTGGTTAGGAGGCTGAACGCCGTCCTCCTACCCAATGTACATACACTATTTGACATGTCTGCCGAGGATTTCGATGCCATCATAGCCGCACACTTTAAGCCAGGAGACACTGTTTTGGAAACGGACATAGCCTCCTTCGATAAGAGCCAAGATGATTCACTTGCGCTTACTGCTTTGATGCTGTTAGAGGATTTAGGGGTGGATCACTCCCTGCTGGACTTGATAGAGGCTGCTTTCGGAGAGATTTCCAGCTGTCACCTACCGACAGGTACGCGCTTCAAGTTCGGCGCCATGATGAAATCAGGTATGTTCCTAACTCTGTTCGTCAACACATTGTTAAACATCACCATCGCCAGCCGAGTGCTGGAAGATCGTCTGACAAAATCCGCGTGCGCGGCCTTCATCGGCGACGACAACATAATACATGGAGTCGTCTCCGATGAATTGATGGCAGCCAGATGTGCCACTTGGATGAACATGGAAGTGAAGATCATAGATGCAGTTGTATCCTTGAAAGCCCCTTACTTTTGTGGAGGGTTTATACTGCACGATACTGTGACAGGAACAGCTTGCAGAGTGGCAGACCCGCTAAAAAGGCTTTTTAAACTGGGCAAACCGCTAGCGGCAGGTGACGAACAAGATGAAGATAGAAGACGAGCGCTGGCTGACGAAGTGATCAGATGGCAACGAACAGGGCTAATTGATGAGCTGGAGAAAGCGGTATACTCTAGGTACGAAGTGCAGGGTATATCAGTTGTGGTAATGTCCATGGCCACCTTTGCAAGCTCCAGATCCAACTTCGAGAAGCTCAGAGGACCCGTCATAACTTTGTACGGCGGTCCTAAATAGGTACGCACTACAGCTACCTATTTTGCAGAAGCCGACAGCAAGTATCTAAACACTAATCAGCTACAATGGAGTTCATCCCAACCCAAACTTTTTACAATAGGAGGTACCAGCCTCGACCCTGGACTCCGCGCTCTACTATCCAAATCATCAGGCCCAGACCGCGCCCTCAGAGGCAAGCTGGGCAACTTGCCCAGCTGATCTCAGCAGTTAATAAACTGACAATGCGCGCGGTACCCCAACAGAAGCCACGCAGGAATCGGAAGAATAAGAAGCAAAAGCAAAAACAACAGGCGCCACAAAACAACACAAATCAAAAGAAGCAGCCACCTAAAAAGAAACCGGCTCAAAAGAAAAAGAAGCCGGGCCGCAGAGAGAGGATGTGCATGAAAATCGAAAATGATTGTATTTTCGAAGTCAAGCACGAAGGTAAGGTAACAGGTTACGCGTGCCTGGTGGGGGACAAAGTAATGAAACCAGCACACGTAAAGGGGACCATCGATAACGCGGACCTGGCCAAACTGGCCTTTAAGCGGTCATCTAAGTATGACCTTGAATGCGCGCAGATACCCGTGCACATGAAGTCCGACGCTTCGAAGTTCACCCATGAGAAACCGGAGGGGTACTACAACTGGCACCACGGAGCAGTACAGTACTCAGGAGGCCGGTTCACCATCCCTACAGGTGCTGGCAAACCAGGGGACAGCGGCAGACCGATCTTCGACAACAAGGGACGCGTGGTGGCCATAGTCTTAGGAGGAGCTAATGAAGGAGCCCGTACAGCCCTCTCGGTGGTGACCTGGAATAAAGACATTGTCACTAAAATCACCCCCGAGGGGGCCGAAGAGTGGAGTCTTGCCATCCCAGTTATGTGCCTGTTGGCAAACACCACGTTCCCCTGCTCCCAGCCCCCTTGCACGCCCTGCTGCTACGAAAAGGAACCGGAGGAAACCCTACGCATGCTTGAGGACAACGTCATGAGACCTGGGTACTATCAGCTGCTACAAGCATCCTTAACATGTTCTCCCCACCGCCAGCGACGCAGCACCAAGGACAACTTCAATGTCTATAAAGCCACAAGACCATACTTAGCTCACTGTCCCGACTGTGGAGAAGGGCACTCGTGCCATAGTCCCGTAGCACTAGAACGCATCAGAAATGAAGCGACAGACGGGACGCTGAAAATCCAGGTCTCCTTGCAAATCGGAATAAAGACGGATGACAGCCACGATTGGACCAAGCTGCGTTATATGGACAACCACATGCCAGCAGACGCAGAGAGGGCGGGGCTATTTGTAAGAACATCAGCACCGTGTACGATTACTGGAACAATGGGACACTTCATCCTGGCCCGATGTCCAAAAGGGGAAACTCTGACGGTGGGATTCACTGACAGTAGGAAGATTAGTCATTCATGTACGCACCCATTTCACCACGACCCTCCTGTGATAGGTCGGGAAAAATTCTATTCCCGACCGCAGCACGGTAAAGAGCTACCTTGCAGCACGTACGTGCAGAGCACCGCCGCAACTACCGAGGAGATAGAGGTACACATGCCCCCAGACACCCCTGATCGCACATTAATGTCACAACAGTCCGGCAACGTAAAGATCACAGTCAATGGCCAGACGGTGCGGTACAAGTGTAATTGCGGTGGCTCAAATGAAGGACTAACAACTACAGACAAAGTGATTAATAACTGCAAGGTTGATCAATGTCATGCCGCGGTCACCAATCACAAAAAGTGGCAGTATAACTCCCCTCTGGTCCCGCGTAATGCTGAACTTGGGGACCGAAAAGGAAAAATTCACATCCCGTTTCCGCTGGCAAATGTAACATGCAGGGTGCCTAAAGCAAGGAACCCCACCGTGACGTACGGGAAAAACCAAGTCATCATGCTACTGTATCCTGACCACCCAACACTCCTGTCCTACCGGAATATGGGAGAAGAACCAAACTATCAAGAAGAGTGGGTGATGCATAAGAAGGAAGTCGTGCTAACCGTGCCGACTGAAGGGCTCGAGGTCACGTGGGGCAACAACGAGCCGTATAAGTATTGGCCGCAGCTATCTACAAACGGTACAGCCCATGGCCACCCGCATGAGATAATTCTGTATTATTATGAGCTGTACCCTACTATGACTGTAGTAGTTGTGTCAGTGGCCACGTTCATACTCCTGTCGATGGTGGGTATGGCAGCGGGGATGTGCATGTGTGCACGACGCAGATGCATCACACCGTATGAACTGACACCAGGAGCTACCGTCCCTTTCCTGCTTAGCCTAATATGCTGCATCAGAACAGCTAAAGCGGCCACATACCAAGAGGCTGCGATATACCTGTGGAACGAGCAGCAACCTTTGTTTTGGCTACAAGCCCTTATTCCGCTGGCAGCCCTGATTGTTCTATGCAACTGTCTGAGACTCTTACCATGCTGCTGTAAAACGTTGGCTTTTTTAGCCGTAATGAGCGTCGGTGCCCACACTGTGAGCGCGTACGAACACGTAACAGTGATCCCGAACACGGTGGGAGTACCGTATAAGACTCTAGTCAATAGACCTGGCTACAGCCCCATGGTATTGGAGATGGAACTACTGTCAGTCACTTTGGAGCCAACACTATCGCTTGATTACATCACGTGCGAGTACAAAACCGTCATCCCGTCTCCGTACGTGAAGTGCTGCGGTACAGCAGAGTGCAAGGACAAAAACCTACCTGACTACAGCTGTAAGGTCTTCACCGGCGTCTACCCATTTATGTGGGGCGGCGCCTACTGCTTCTGCGACGCTGAAAACACGCAGTTGAGCGAAGCACATGTGGAGAAGTCCGAATCATGCAAAACAGAATTTGCATCAGCATACAGGGCTCATACCGCATCTGCATCAGCTAAGCTCCGCGTCCTTTACCAAGGAAATAACATCACTGTAACTGCCTATGCAAACGGCGACCATGCCGTCACAGTTAAGGACGCCAAATTCATTGTGGGGCCAATGTCTTCAGCCTGGACACCTTTCGACAACAAAATTGTGGTGTACAAAGGTGACGTCTATAACATGGACTACCCGCCCTTTGGCGCAGGAAGACCAGGACAATTTGGCGATATCCAAAGTCGCACACCTGAGAGTAACGACGTCTATGCTAATACACAACTGGTACTGCAGAGACCGGCTGCGGGTACGGTACACGTGCCATACTCTCAGGCACCATCTGGCTTTAAGTATTGGCTAAAGGAACGCGGGGCGTCACTGCAGCACACAGCACCATTTGGCTGCCAAATAGCAACAAACCCGGTAAGAGCGGTGAACTGCGCCGTAGGGAACATGCCCATCGCCATCGACATACCGGAAGCGGCCTTCACTAGGGTCGTCGACGCGCCCTCTTTAACGGACATGTCGTGCGAGGTACCAGCCTGCACCCATTCCTCAGACTTTGGGGGCGTCGCCATTATTAAATATGCAGCCAGCAAGAAAGGCAAGTGTGCGGTGCATTCGATGACTAACGCCGTCACTATTCGGGAAGCTGAGATAGAAGTTGAAGGGAATTCTCAGCTGCAAATCTCTTTCTCGACGGCCTTAGCCAGCGCCGAATTCCGCGTACAAGTCTGTTCTACACAAGTACACTGTGCAGCTGAGTGCCACCCCCCGAAGGACCACATAGTCAACTACCCGGCGTCACATACCACCCTCGGGGTCCAGGACATCTCCGCTACGGCGATGTCATGGGTGCAGAAGATCACGGGAGGTGTGGGACTGGTTGTTGCTGTTGCCGCACTGATTCTAATCGTGGTGCTATGCGTGTCGTTCAGCAGGCACTAACTTGACAATTAAGTATGAAGGTATATGTGTCCCCTAAGAGACACACTGTACATAGCAAATAATCTATAGATCAAAGGGCTACGCAACCCCTGAATAGTAACAAAATACAAAATCACTAAAAATTATAAAAACAGAAAAATACATAAATAGGTATACGTGTCCCCTAAGAGACACATTGTATGTAGGTGATAAGTATAGATCAAAGGGCCGAATAACCCCTGAATAGTAACAAAATATGAAAATCAATAAAAATCATAAAATAGAAAAACCATAAACAGAAGTAGTTCAAAGGGCTATAAAACCCCTGAATAGTAACAAAACATAAAGTTAATAAAAATCAAATGAATACCATAATTGGCAAACGGAAGAGATGTAGGTACTTAAGCTTCCTAAAAGCAGCCGAACTCACTTTGAGAAGTAGGCATAGCATACCGAACTCTTCCACGATTCTCCGAACCCACAGGGACGTAGGAGATGTTATTTTGTTTTTAATATTTC

>FJ000065|2006|India: Gujarat, Group 4

TTTGGCTGCGTGAGACACACGTAGCCTACCAGTTTCTTACTGCTCTACTCTGCAAAGCAAGAGATTAATAACCCATCATGGATCCTGTGTACGTGGACATAGACGCTGACAGCGCCTTTTTGAAGGCCCTGCAACGTGCGTACCCCATGTTTGAGGTGGAACCAAGGCAGGTCACACCGAATGACCATGCTAATGCTAGAGCGTTCTCGCATCTAGCTATAAAACTAATAGAGCAGGAAATTGACCCCGACTCAACCATCCTGGATATCGGCAGTGCGCCAGCAAGGAGGATGATGTCGGACAGGAAGTACCACTGCGTCTGCCCGATGCGCAGTGCGGAAGATCCCGAGAGACTCGCTAATTATGCGAGAAAGCTAGCATCTGCCGCAGGAAAAGTCCTGGACAGAAACATCTCTGGAAAGATCGGGGACTTACAAGCAGTAATGGCCGTGCCAGACAAGGAGACGCCAACATTCTGCTTACACACAGACGTCTCATGTAGACAGAGAGCAGACGTCGCTATATACCAAGACGTCTATGCTGTACACGCACCCACGTCGCTATACCACCAGGCGATTAAAGGGGTCCGAGTGGCGTACTGGGTTGGGTTCGACACAACCCCGTTCATGTACAATGCCATGGCGGGTGCCTACCCCTCATACTCGACAAACTGGGCAGATGAGCAGGTACTGAAGGCTAAGAACATAGGATTATGTTCAACAGACCTGACGGAAGGTAGACGAGGCAAGTTGTCTATTATGAGAGGGAAAAAGCTAAAACCGTGCGACCGTGTGCTGTTCTCAGTAGGGTCAACGCTCTACCCGGAAAGCCGCAAGCTACTTAAGAGCTGGCACCTGCCATCGGTGTTCCATTTAAAGGGCAAACTCAGCTTCACATGCCGCTGTGATACAGTGGTTTCGTGTGAGGGCTACGTCGTTAAGAGAATAACGATGAGCCCAGGCCTTTATGGAAAAACCACAGGGTATGCGGTAACCCACCACGCAGACGGATTCCTGATGTGCAAGACTACCGACACGGTTGACGGCGAAAGAGTGTCATTCTCGGTGTGCACATACGTGCCGGCGACCATTTGTGATCAAATGACCGGCATCCTTGCTACAGAAGTCACGCCGGAGGATGCACAGAAGCTGTTGGTGGGGCTGAACCAGAGAATAGTGGTTAACGGCAGAACGCAACGGAATATGAACACCATGAAAAATTATCTGCTTCCCGTGGTCGCCCAAGCCTTCAGTAAGTGGGCAAAGGAGTGCCGGAAAGACATGGAAGATGAAAAACTCCTGGGGGTCAGAGAAAGAACACTGACCTGCTGCTGTCTATGGGCATTCAAGAAGCAGAAAACACACACGGTCTACAAGAGGCCTGATACCCAGTCAATTCAGAAGGTTCAGGCCGAGTTTGACAGCTTTGTGGTACCGAGTCTGTGGTCGTCCGGGTTGTCAATCCCTTTGAGGACTAGAATCAAATGGTTGTTAAGCAAGGTGCCAAAAACCGACCTGATCCCATACAGCGGAGACGCCCGAGAAGCCCGGGACGCAGAAAAAGAAGCAGAGGAAGAACGAGAAGCAGAACTGACTCGCGAAGCCCTACCACCTCTACAGGCAGCACAGGAAGATGTTCAGGTCGAAATCGACGTGGAACAGCTTGAGGACAGAGCGGGCGCAGGAATAATAGAGACTCCGAGAGGAGCTATCAAAGTTACTGCCCAACCAACAGACCACGTCGTGGGAGAGTACCTGGTACTCTCCCCGCAGACCGTACTACGTAGCCAGAAGCTCAGTCTGATTCACGCTTTGGCGGAGCAAGTGAAGACGTGCACGCACAACGGACGAGCAGGGAGGTATGCGGTCGAAGCGTACGACGGCCGAGTCCTAGTGCCCTCAGGCTATGCAATCTCGCCTGAAGACTTCCAGAGTCTAAGCGAAAGCGCAACGATGGTGTATAACGAAAGAGAGTTCGTAAACAGAAAGCTACACCATATTGCGATGCACGGACCAGCCCTGAACACCGACGAAGAGTCGTATGAGCTGGTGAGGGCAGAGAGGACAGAACACGAGTACGTCTACGACGTGGATCAGAGAAGATGCTGTAAGAAGGAAGAAGCCGCAGGACTGGTACTGGTGGGCGACTTGACTAATCCGCCCTACCACGAATTCGCATATGAAGGGCTAAAAATCCGCCCTGCCTGCCCATACAAAATTGCAGTCATAGGAGTCTTCGGAGTACCGGGATCTGGCAAGTCAGCTATTATCAAGAACCTAGTTACCAGGCAGGACCTGGTGACTAGCGGAAAGAAAGAAAACTGCCAAGAAATCACCACCGACGTGATGAGACAGAGAGGTCTAGAGATATCTGCACGTACGGTTGACTCGCTGCTCTTGAATGGATGCAACAGACCAGTCGACGTGTTGTACGTAGACGAGGCGTTTGCGTGCCACTCTGGAACGCTACTTGCTTTGATCGCCTTGGTGAGACCAAGGCAGAAAGTTGTACTTTGTGGTGACCCGAAGCAGTGCGGCTTCTTCAATATGATGCAGATGAAAGTCAACTATAATCACAACATCTGCACCCAAGTGTACCACAAAAGTATCTCCAGGCGGTGTACACTGCCTGTGACCGCCATTGTGTCATCGTTGCATTACGAAGGCAAAATGCGCACTACGAATGAGTACAACAAGCCGATTGTAGTGGACACTACAGGCTCAACAAAACCTGACCCTGGAGACCTCGTGTTAACGTGCTTCAGAGGGTGGGTTAAACAACTGCAAATTGACTATCGTGGATACGAGGTCATGACAGCAGCCGCATCCCAAGGGTTAACCAGAAAAGGAGTTTACGCAGTTAGACAAAAAGTTAATGAAAACCCGCTCTATGCATCAACGTCAGAGCACGTCAACGTACTCCTAACGCGTACGGAAGGTAAACTGGTATGGAAGACACTTTCCGGCGACCCGTGGATAAAGACGCTGCAGAACCCACCGAAAGGAAACTTCAAAGCAACTATTAAGGAGTGGGAGGTGGAGCATGCATCAATAATGGCGGGCATCTGCAGTCACCAAATGACCTTCGATACATTCCAAAATAAAGCCAACGTTTGCTGGGCTAAGAGCTTGGTCCCTATCCTCGAAACAGCGGGGATAAAACTAAATGATAGGCAGTGGTCTCAGATAATTCAAGCCTTCAAAGAAGACAAAGCATACTCACCTGAAGTAGCCCTGAATGAAATATGTACGCGCATGTATGGGGTGGATCTAGACAGCGGGCTATTTTCTAAACCGTTGGTGTCTGTGTATTACGCGGATAACCACTGGGATAATAGGCCTGGAGGGAAAATGTTCGGATTTAACCCCGAGGCAGCATCCATTCTAGAAAGAAAGTATCCATTCACAAAAGGGAAGTGGAACATCAACAAGCAGATCTGCGTGACTACCAGGAGGATAGAAGACTTTAACCCTACCACCAACATCATACCGGCCAACAGGAGACTACCACACTCATTAGTGGCCGAACACCGCCCAGTAAAAGGGGAAAGAATGGAATGGCTGGTTAACAAGATAAACGGCCACCACGTGCTCCTGGTCAGTGGCTATAACCTTGCACTGCCTACTAAGAGAGTCACTTGGGTAGCGCCGTTAGGTGTCCGCGGAGCGGACTACACATACAACCTAGAGTTGGGTCTGCCAGCAACGCTTGGTAGGTATGACCTTGTGGTCATAAACATCCACACACCTTTTCGCATACACCATTACCAACAGTGCGTCGACCACGCAATGAAACTGCAAATGCTCGGGGGTGACTCATTGAGACTGCTCAAACCGGGCGGCTCTCTATTGATCAGAGCATATGGTTACGCAGATAGAACCAGTGAACGAGTCATCTGCGTATTGGGACGCAAGTTTAGATCGTCTAGAGCGTTGAAACCACCATGTGTCACCAGCAACACTGAGATGTTTTTCCTATTCAGCAACTTTGACAATGGCAGAAGGAATTTCACAACTCATGTCATGAACAATCAACTGAATGCAGCCTTCGTAGGACAGGTCACCCGAGCAGGATGTGCACCGTCGTACCGGGTAAAACGCATGGACATCGCGAAGAACGATGAAGAGTGCGTAGTCAACGCCGCTAACCCTCGCGGGTTACCGGGTGACGGTGTTTGCAAGGCAGTATACAAAAAATGGCCGGAGTCCTTTAAGAACAGTGCAACACCAGTGGGAACCGCAAAAACAGTTATGTGCGGTACGTATCCAGTAATCCACGCTGTTGGACCAAACTTCTCTAATTATTCGGAGTCTGAAGGGGACCGGGAATTGGCAGCTGCCTATCGAGAAGTCGCAAAGGAAGTAACTAGGCTGGGAGTAAATAGTGTAGCTATACCTCTCCTCTCCACAGGTGTATACTCAGGAGGGAAAGACAGGCTGACCCAGTCACTGAACCACCTCTTTACAGCCATGGACTCGACGGATGCAGACGTGGTCATCTACTGCCGCGACAAAGAATGGGAGAAGAAAATATCTGAGGCCATACAGATGCGGACCCAAGTAGAGCTGCTGGATGAGCACATCTCCATAGACTGCGATATTGTTCGCGTGCACCCTGACAGCAGCTTGGCAGGCAGAAAAGGATACAGCACCACGGAAGGCGCACTGTACTCATATCTAGAAGGGACCCGTTTTCATCAGACGGCTGTGGATATGGCGGAGATACATACTATGTGGCCAAAGCAAACAGAGGCCAATGAGCAAGTCTGCCTATATGCCCTGGGGGAAAGTATTGAATCGATCAGGCAGAAATGCCCGGTGGATGATGCAGACGCATCATCTCCCCCCAAAACTGTCCCGTGCCTTTGCCGTTACGCTATGACTCCAGAACGCGTCACCCGGCTTCGCATGAACCACGTCACAAGCATAATTGTGTGTTCTTCGTTTCCCCTCCCAAAGTACAAAATAGAAGGAGTGCAAAAAGTCAAATGCTCTAAGGTAATGCTATTTGACCACAACGTGCCATCGCGCGTAAGTCCAAGGGAATATAGATCTTCCCAGGAGTCTGCACAGGAGGCGAGTACAATCACGTCACTGACGCATAGTCAATTCGACCTAAGCGTTGATGGCGAGATACTGCCCGTCCCGTCAGACCTGGATGCTGACGCCCCAGCCCTAGAACCAGCACTAGACGACGGGGCGACACACACGCTGCCATCCACAACCGGAAACCTTGCGGCCGTGTCTGACTGGGTAATGAGCACCGTACCTGTCGCGCCGCCCAGAAGAAGGCGAGGGAGAAACCTGACTGTGACATGTGACGAGAGAGAAGGGAATATAACACCCATGGCTAGCGTCCGATTCTTTAGGGCAGAGCTGTGTCCGGTCGTACAAGAAACAGCGGAGACGCGTGACACAGCAATGTCTCTTCAGGCACCACCGAGTACCGCCACGGAACCGAATCATCCGCCGATCTCCTTCGGAGCATCAAGCGAGACGTTCCCCATTACATTTGGGGACTTCAACGAAGGAGAAATCGAAAGCTTGTCTTCTGAGCTACTAACTTTCGGAGACTTCTTACCAGGAGAAGTGGATGACTTGACAGACAGCGACTGGTCCACGTGCTCAGACACGGACGACGAGTTATGACTAGACAGGGCAGGTGGGTATATATTCTCGTCGGACACCGGTCCAGGTCATTTACAACAGAAGTCAGTACGCCAGTCAGTGCTGCCGGTGAACACCCTGGAGGAAGTCCACGAGGAGAAGTGTTACCCACCTAAGCTGGATGAAGCAAAGGAGCAACTATTACTTAAGAAACTCCAGGAGAGTGCATCCATGGCCAACAGAAGCAGGTATCAGTCGCGCAAAGTAGAAAACATGAAAGCAGCAATCATCCAGAGACTAAAGAGAGGCTGTAGACTATACTTAATGTCAGAGACCCCAAAAGTCCCTACTTACCGGACTACATATCCGGCGCCTGTGTACTCGCCTCCGATCAACGTCCGATTGTCCAATCCCGAGTCCGCAGTGGCAGCATGCAATGAGTTCTTAGCTAGAAACTATCCAACTGTCTCATCATACCAAATTACCGACGAGTATGATGCATATCTAGACATGGTGGACGGGTCGGAGAGTTGCCTGGACCGAGCGACATTCAATCCGTCAAAACTCAGGAGCTACCCGAAACAGCACGCTTACCACGCGCCCTCCATCAGAAGCGCTGTACCGTCCCCATTCCAGAACACACTACAGAATGTACTGGCAGCAGCCACGAAAAGAAACTGCAACGTCACACAGATGAGGGAATTACCCACTTTGGACTCAGCAGTATTCAACGTGGAGTGTTTCAAAAAATTCGCATGCAACCAAGAATACTGGGAAGAATTTGCTGCCAGCCCTATTAGGATAACAACTGAGAATTTAGCAACCTATGTTACTAAACTAAAAGGGCCAAAAGCAGCAGCGCTATTCGCAAAAACCCATAATCTACTGCCACTACAGGAAGTACCAATGGATAGGTTCACTGTAGATATGAAAAGGGACGTGAAGGTGACTCCTGGTACAAAGCATACAGAGGAAAGACCTAAGGTGCAGGTTATACAGGCGGCTGAACCCTTGGCGACAGCATACCTATGTGGGATTCACAGAGAGCTGGTTAGGAGGCTGAACGCCGTCCTCCTACCCAATGTACATACACTATTTGACATGTCTGCCGAGGATTTCGATGCCATCATAGCCGCACACTTTAAGCCAGGAGACACTGTTTTGGAAACGGACATAGCCTCCTTTGATAAGAGCCAAGATGATTCACTTGCGCTTACTGCTTTGATGCTGTTAGAGGATTTAGGGGTGGATCACTCCCTGCTGGACTTGATAGAGGCTGCTTTCGGAGAGATTTCCAGCTGTCACCTACCGACAGGTACGCGCTTCAAGTTCGGCGCCATGATGAAATCAGGTATGTTCCTAACTCTGTTCGTCAACACATTGTTAAACATCACCATCGCCAGCCGAGTGCTGGAAGATCGTCTGACAAAATCCGCGTGCGCGGCCTTCATCGGCGACGACAATATAATACATGGAGTCGTCTCCGATGAATTGATGGCAGCCAGATGTGCCACTTGGATGAACATGGAAGTGAAGATCATAGATGCAGTTGTATCCTTGAAAGCCCCTTACTTTTGTGGAGGGTTTATACTGCACGATACTGTGACAGGAACAGCTTGCAGAGTGGCAGACCCGCTAAAAAGGCTTTTTAAACTGGGCAAACCGCTAGCGGCAGGTGACGAACAAGATGAAGATAGAAGACGAGCGCTGGCTGACGAAGTGATCAGATGGCAACGAACAGGGCTAATTGATGAGCTGGAGAAAGCGGTATACTCTAGGTACGAAGTGCAGGGTATATCAGTTGTGGTAATGTCCATGGCCACCTTTGCAAGCTCCAGATCCAACTTCGAGAAGCTCAGAGGACCCGTCATAACTTTGTACGGCGGTCCTAAATAGGTACGCACTACAGCTACCTATTTTGCAGAAGCCGACAGCAAGTATCTAAACACTAATCAGCTACAATGGAGTTCATCCCAACCCAAACTTTTTACAATAGGAGGTACCAGCCTCGACCCTGGACTCCGCGCTCTACTATCCAAATCATCAGGCCCAGACCGCGCCCTCAGAGGCAAGCTGGGCAACTTGCCCAGCTGATCTCAGCAGTTAATAAACTGACAATGCGCGCGGTACCCCAACAGAAGCCACGCAGGAATCGGAAGAATAAGAAGCAAAAGCAAAAACAACAGGCGCCACAAAACAACACAAATCAAAAGAAGCAGCCACCTAAAAAGAAACCGGCTCAAAAGAAAAAGAAGCCGGGCCGCAGAGAGAGGATGTGCATGAAAATCGAAAATGATTGTATTTTCGAAGTCAAGCACGAAGGTAAGGTAACAGGTTACGCGTGCCTGGTGGGGGACAAAGTAATGAAACCAGCACACGTAAAGGGGACCATCGATAACGCGGACCTGGCCAAACTGGCCTTTAAGCGGTCATCTAAGTATGACCTTGAATGCGCGCAGATACCCGTGCACATGAAGTCCGACGCTTCGAAGTTCACCCATGAGAAACCGGAGGGGTACTACAACTGGCACCACGGAGCAGTACAGTACTCAGGAGGCCGGTTCACCATCCCTACAGGTGCTGGCAAACCAGGGGACAGCGGCAGACCGATCTTCGACAACAAGGGACGCGTGGTGGCCATAGTCTTAGGAGGAGCTAATGAAGGAGCCCGTACAGCCCTCTCGGTGGTGACCTGGAATAAAGACATTGTCACTAAAATCACCCCCGAGGGGGCCGAAGAGTGGAGTCTTGCCATCCCAGTTATGTGCCTGTTGGCAAACACCACGTTCCCCTGCTCCCAGCCCCCTTGCACGCCCTGCTGCTACGAAAAGGAACCGGAGGAAACCCTACGCATGCTTGAGGACAACGTCATGAGACCTGGGTACTATCAGCTGCTACAAGCATCCTTAACATGTTCTCCCCACCGCCAGCGACGCAGCACCAAGGACAACTTCAATGTCTATAAAGCCACAAGACCATACTTAGCTCACTGTCCCGACTGTGGAGAAGGGCACTCGTGCCATAGTCCCGTAGCACTAGAACGCATCAGAAATGAAGCGACAGACGGGACGCTGAAAATCCAGGTCTCCTTGCAAATCGGAATAAAGACGGATGACAGCCACGATTGGACCAAGCTGCGTTATATGGACAACCACATGCCAGCAGACGCAGAGAGGGCGGGGCTATTTGTAAGAACATCAGCACCGTGTACGATTACTGGAACAATGGGACACTTCATCCTGGCCCGATGTCCAAAAGGGGAAACTCTGACGGTGGGATTCACTGACAGTAGGAAGATTAGTCATTCATGTACGCACCCATTTCACCACGACCCTCCTGTGATAGGTCGGGAAAAATTCCATTCCCGACCGCAGCACGGTAAAGAGCTACCTTGCAGCACGTACGTGCAGAGCACCGCCGCAACTACCGAGGAGATAGAGGTACACATGCCCCCAGACACCCCTGATCGCACATTAATGTCACAACAGTCCGGCAACGTAAAGATCACAGTCAATGGCCAGACGGTGCGGTACAAGTGTAATTGCGGTGGCTCAAATGAAGGACTAACAACTACAGACAAAGTGATTAATAACTGCAAGGTTGATCAATGTCATGCCGCGGTCACCAATCACAAAAAGTGGCAGTATAACTCCCCTCTGGTCCCGCGTAATGCTGAACTTGGGGACCGAAAAGGAAAAATTCACATCCCGTTTCCGCTGGCAAATGTAACATGCAGGGTGCCTAAAGCAAGGAACCCCACCGTGACGTACGGGAAAAACCAAGTCATCATGCTACTGTATCCTGACCACCCAACACTCCTGTCCTACCGGAATATGGGAGAAGAACCAAACTATCAAGAAGAGTGGGTGATGCATAAGAAGGAAGTCGTGCTAACCGTGCCGACTGAAGGGCTCGAGGTCACGTGGGGCAACAACGAGCCGTATAAGTATTGGCCGCAGTTATCTACAAACGGTACAGCCCATGGCCACCCGCATGAGATAATTCTGTATTATTATGAGCTGTACCCTACTATGACTGTAGTAGTTGTGTCAGTGGCCACGTTCATACTCCTGTCGATGGTGGGTATGGCAGCGGGGATGTGCATGTGTGCACGACGCAGATGCATCACACCGTATGAACTGACACCAGGAGCTACCGTCCCTTTCCTGCTTAGCCTAATATGCTGCATCAGAACAGCTAAAGCGGCCACATACCAAGAGGCTGCGATATACCTGTGGAACGAGCAGCAACCTTTGTTTTGGCTACAAGCCCTTATTCCGCTGGCAGCCCTGATTGTTCTATGCAACTGTCTGAGACTCTTACCATGCTGCTGTAAAACGTTGGCTTTTTTAGCCGTAATGAGCGTCGGTGCCCACACTGTGAGCGCGTACGAACACGTAACAGTGATCCCGAACACGGTGGGAGTACCGTATAAGACTCTAGTCAATAGACCTGGCTACAGCCCCATGGTATTGGAGATGGAACTACTGTCAGTCACTTTGGAGCCAACACTATCGCTTGATTACATCACGTGCGAGTACAAAACCGTCATCCCGTCTCCGTACGTGAAGTGCTGCGGTACAGCAGAGTGCAAGGACAAAAACCTACCTGACTACAGCTGTAAGGTCTTCACCGGCGTCTACCCATTTATGTGGGGCGGCGCCTACTGCTTCTGCGACGCTGAAAACACGCAGTTGAGCGAAGCACATGTGGAGAAGTCCGAATCATGCAAAACAGAATTTGCATCAGCATACAGGGCTCATACCGCATCTGCATCAGCTAAGCTCCGCGTCCTTTACCAAGGAAATAACATCACTGTAACTGCCTATGCAAACGGCGACCATGCCGTCACAGTTAAGGACGCCAAATTCATTGTGGGGCCAATGTCTTCAGCCTGGACACCTTTCGACAACAAAATTGTGGTGTACAAAGGTGACGTCTATAACATGGACTACCCGCCCTTTGGCGCAGGAAGACCAGGACAATTTGGCGATATCCAAAGTCGCACACCTGAGAGTAAAGACGTCTATGCTAATACACAACTGGTACTGCAGAGACCGGCTGCGGGTACGGTACACGTGCCATACTCTCAGGCACCATCTGGCTTTAAGTATTGGCTAAAAGAACGCGGGGCGTCACTGCAGCACACAGCACCATTTGGCTGCCAAATAGCAACAAACCCGGTAAGAGCGGTGAACTGCGCCGTAGGGAACATGCCCATCTCCATCGACATACCGGAAGCGGCCTTCACTAGGGTCGTCGACGCGCCCTCTTTAACGGACATGTCGTGCGAGGTACCAGCCTGCACCCATTCCTCAGACTTTGGGGGCGTCGCCATTATTAAATATGCAGCCAGCAAGAAAGGCAAGTGTGCGGTGCATTCGATGACTAACGCCGTCACTATTCGGGAAGCTGAGATAGAAGTTGAAGGGAATTCTCAGCTGCAAATCTCTTTCTCGACGGCCTTAGCCAGCGCCGAATTCCGCGTACAAGTCTGTTCTACACAAGTACACTGTGCAGCTGAGTGCCACCCCCCGAAGGACCACATAGTCAACTACCCGGCGTCACATACCACCCTCGGGGTCCAGGACATCTCCGCTACGGCGATGTCATGGGTGCAGAAGATCACGGGAGGTGTGGGACTGGTTGTTGCTGTTGCCGCACTGATTCTAATCGTGGTGCTATGCGTGTCGTTCAGCAGGCACTAACTTGACAATTAAGTATGAAGGTATATGTGTCCCCTAAGAGACACACTGTACATAGCAAATAATCTATAGATCAAAGGGCTACGCAACCCCTGAATAGTAACAAAATACAAAATCACTAAAAATTATAAAAACAGAAAAATACATAAATAGGTATACGTGTCCCCTAAGAGACACATTGTATGTAGGTGATAAGTATAGATCAAAGGGCCGAATAACCCCTGAATAGTAACAAAATATGAAAATCAATAAAAATCATAAAATAGAAAAAACCATAAACAGAAGTAGTTCAAAGGGCTATAAAACCCCTGAATAGTAACAAAACATAAAATTAATAAAAATCAAATGAATACCATAATTGGCAAACGGAAGAGATGTAGGTACTTAAGCTTCCTAAAAGCAGCCGAACTCACTTTGAGAAGTAGGCATAGCATACCGAACTCTTCCACGATTCTCCGAACCCACAGGGACGTAGGAGATGTTATTTTGTTTTTAATATTTC

>FJ000066|2006|India: Kerala, Group 4

TTTGGCTGCGTGAGACACACGTAGCCTACCAGTTTCTTACTGCTCTACTCTGCAAAGCAAGAGATTAATAACCCATCATGGATCCTGTGTACGTGGACATAGACGCTGACAGCGCCTTTTTGAAGGCCCTGCAACGTGCGTACCCCATGTTTGAGGTGGAACCAAGGCAGGTCACACCGAATGACCATGCTAATGCTAGAGCGTTCTCGCATCTAGCTATAAAACTAATAGAGCAGGAAATTGACCCCGACTCAACCATCCTGGATATCGGCAGTGCGCCAGCAAGGAGGATGATGTCGGACAGGAAGTACCACTGCGTCTGCCCGATGCGCAGTGCGGAAGATCCCGAGAGACTCGCTAATTATGCGAGAAAGCTAGCATCTGCCGCAGGAAAAGTCCTGGACAGAAACATCTCTGGAAAGATCGGGGACTTACAAGCAGTAATGGCCGTGCCAGACAAGGAGACGCCAACATTCTGCTTACACACAGACGTCTCATGTAGACAGAGAGCAGACGTCGCTATATACCAAGACGTCTATGCTGTACACGCACCCACGTCGCTATACCACCAGGCGATCAAAGGGGTCCGAGTGGCGTACTGGGTTGGGTTCGACACAACCCCGTTCATGTACAATGCCATGGCGGGTGCCTACCCCTCATACTCGACAAACTGGGCAGATGAGCAGGTACTGAAGGCTAAGAACATAGGATTATGTTCAACAGACCTGACGGAAGGTAGACGAGGCAAGTTGTCTATTATGAGAGGGAAAAAGCTAAAACCGTGCGACCGTGTGCTGTTCTCAGTAGGGTCAACGCTCTACCCGGAAAGCCGCAAGCTACTTAAGAGCTGGCACCTGCCATCGGTGTTCCATTTAAAGGGCAAACTCAGCTTCACATGCCGCTGTGATACAGTGGTTTCGTGTGAGGGCTACGTCGTTAAGAGAATAACGATGAGCCCAGGCCTTTATGGAAAAACCACAGGGTATGCGGTAACCCACCACGCAGACGGATTCCTGATGTGCAAGACTACCGACACGGTTGACGGCGAAAGAGTGTCATTCTCGGTGTGCACATACGTGCCGGCGACCATTTGTGATCAAATGACCGGCATCCTTGCTACAGAAGTCACGCCGGAGGATGCACAGAAGCTGTTGGTGGGGCTGAACCAGAGAATAGTGGTTAACGGCAGAACGCAACGGAATATGAACACCATGAAAAATTATCTGCTTCCCGTGGTCGCCCAAGCCTTCAGTAAGTGGGCAAAGGAGTGCCGGAAAGACATGGAAGATGAAAAACTCCTGGGGGTCAGAGAAAGAACACTGACCTGCTGCTGTCTATGGGCATTCAAGAAGCAGAAAACACACACGGTCTACAAGAGGCCTGATACCCAGTCAATTCAGAAGGTTCAGGCCGAGTTTGACAGCTTTGTGGTACCGAGTCTGTGGTCGTCCGGGTTGTCAATCCCTTTGAGGACTAGAATCAAATGGTTGTTAAGCAAGGTGCCAAAAACCGACCTGATCCCATACAGCGGAGACGCCCGAGAAGCCCGGGACGCAGAAAAAGAAGCAGAGGAAGAACGAGAAGCAGAACTGACTCGCGAAGCCCTACCACCTCTACAGGCAGCACAGGAAGATGTTCAGGTCGAAATCGACGTGGAACAGCTTGAGGACAGAGCGGGCGCAGGAATAATAGAGACTCCGAGAGGAGCTATCAAAGTTACTGCCCAACCAACAGACCACGTCGTGGGAGAGTACCTGGTACTCTCCCCGCAGACCGTACTACGTAGCCAGAAGCTCAGTCTGATTCACGCTTTGGCGGAGCAAGTGAAGACGTGCACGCACAACGGACGAGCAGGGAGGTATGCGGTCGAAGCGTACGACGGCCGAGTCCTAGTGCCCTCAGGCTATGCAATCTCGCCTGAAGACTTCCAGAGTCTAAGCGAAAGCGCAACGATGGTGTATAACGAAAGAGAGTTCGTAAACAGAAAGCTACACCATATTGCGATGCACGGACCAGCCCTGAACACCGACGAAGAGTCGTGTGAGCTGGTGAGGGCAGAGAGGACAGAACACGAGTACGTCTACGACGTGGATCAGAGAAGATGCTGTAAGAAGGAAGAAGCCGCAGGACTGGTACTGGTGGGCGACTTGACTAATCCGCCCTACCACGAATTCGCATATGAAGGGCTAAAAATCCGCCCTGCCTGCCCATACAAAATTGCAGTCATAGGAGTCTTCGGAGTACCGGGATCTGGCAAGTCAGCTATTATCAAGAACCTAGTTACCAGGCAGGACCTGGTGACTAGCGGAAAGAAAGAAAACTGCCAAGAAATCACCACCGACGTGATGAGACAGAGAGGTCTAGAGATATCTGCACGTACGGTTGACTCGCTGCTCTTGAATGGATGCAACAGACCAGTCGACGTGTTGTACGTAGACGAGGCGTTTGCGTGCCACTCTGGAACGCTACTTGCTTTGATCGCCTTGGTGAGACCAAGGCAGAAAGTTGTACTTTGTGGTGACCCGAAGCAGTGCGGCTTCTTCAATATGATGCAGATGAAAGTCAACTATAATCACAACATCTGCACCCAAGTGTACCACAAAAGTATCTCCAGGCGGTGTACACTGCCTGTGACCGCCATTGTGTCATCGTTGCATTACGAAGGCAAAATGCGCACTACGAATGAGTACAACAAGCCGATTGTAGTGGACACTACAGGCTCAACAAAACCTGACCCTGGAGACCTCGTGTTAACGTGCTTCAGAGGGTGGGTTAAACAACTGCAAATTGACTATCGTGGATACGAGGTCATGACAGCAGCCGCATCCCAAGGGTTAACCAGAAAAGGAGTTTACGCAGTTAGACAAAAAGTTAATGAAAACCCGCTCTATGCATCAACGTCAGAGCACGTCAACGTACTCCTAACGCGTACGGAAGGTAAACTGGTATGGAAGACACTTTCCGGCGACCCGTGGATAAAGACGCTGCAGAACCCACCGAAAGGAAACTTCAAAGCAACTATTAAGGAGTGGGAGGTGGAGCATGCATCAATAATGGCGGGCATCTGCAGTCACCAAATGACCTTCGATACATTCCAAAATAAAGCCAACGTTTGTTGGGCTAAGAGCTTGGTCCCTATCCTCGAAACAGCGGGGATAAAACTAAATGATAGGCAGTGGTCTCAGATAATTCAAGCCTTCAAAGAAGACAAAGCATACTCACCTGAAGTAGCCCTGAATGAAATATGTACGCGCATGTATGGGGTGGATCTAGACAGCGGGCTATTTTCTAAACCGTTGGTGTCTGTGTATTACGCGGATAACCACTGGGATAATAGGCCTGGAGGGAAAATGTTCGGATTTAACCCCGAGGCAGCATCCATTCTAGAAAGAAAGTATCCATTCACAAAAGGGAAGTGGAACATCAACAAGCAGATCTGCGTGACTACCAGGAGGATAGAAGACTTTAACCCTACCACCAACATCATACCGGCCAACAGGAGACTACCACACTCATTAGTGGCCGAACACCGCCCAGTAAAAGGGGAAAGAATGGAATGGCTGGTTAACAAGATAAACGGCCACCACGTGCTCCTGGTCAGTGGCTATAACCTTGCACTGCCTACTAAGAGAGTCACTTGGGTAGCGCCGTTAGGTGTCCGCGGAGCGGACTACACATACAACCTAGAGTTGGGTCTGCCAGCAACGCTTGGTAGGTATGACCTTGTGGTCATAAACATCCACACACCTTTTCGCATACACCATTACCAACAGTGCGTCGACCACGCAATGAAACTGCAAATGCTCGGGGGTGACTCATTGAGACTGCTCAAACCGGGCGGCTCTCTATTGATCAGAGCATATGGTTACGCAGATAGAACCAGTGAACGAGTCATCTGCGTATTGGGACGCAAGTTTAGATCGTCTAGAGCGTTGAAACCACCATGTGTCACCAGCAACACTGAGATGTTTTTCCTATTCAGCAACTTTGACAATGGCAGAAGGAATTTCACAACTCATGTCATGAACAATCAACTGAATGCAGCCTTCGTAGGACAGGTCACCCGAGCAGGATGTGCACCGTCGTACCGGGTAAAACGCATGGACATCGCGAAGAACGATGAAGAGTGCGTAGTCAACGCCGCTAACCCTCGCGGGTTACCGGGTGACGGTGTTTGCAAGGCAGTATACAAAAAATGGCCGGAGTCCTTTAAGAACAGTGCAACACCAGTGGGAACCGCAAAAACAGTTATGTGCGGTACGTATCCAGTAATCCACGCTGTTGGACCAAACTTCTCTAATTATTCGGAGTCTGAAGGGGACCGGGAATTGGCAGCTGCCTATCGAGAAGTCGCAAAGGAAGTAACTAGGCTGGGAGTAAATAGTGTAGCTATACCTCTCCTCTCCACAGGTGTATACTCAGGAGGGAAAGACAGGCTGACCCAGTCACTGAACCACCTCTTTACAGCCATGGACTCGACGGATGCAGACGTGGTCATCTACTGCCGCGACAAAGAATGGGAGAAGAAAATATCTGAGGCCATACAGATGCGGACCCAAGTAGAGCTGCTGGATGAGCACATCTCCATAGACTGCGATATTGTTCGCGTGCACCCTGACAGCAGCTTGGCAGGCAGAAAAGGATACAGCACCACGGAAGGCGCACTGTACTCATATCTAGAAGGGACCCGTTTTCATCAGACGGCTGTGGATATGGCGGAGATACATACTATGTGGCCAAAGCAAACAGAGGCCAATGAGCAAGTCTGCCTATATGCCCTGGGGGAAAGTATTGAATCGATCAGGCAGAAATGCCCGGTGGATGATGCAGACGCATCATCTCCCCCCAAAACTGTCCCGTGCCTTTGCCGTTACGCTATGACTCCAGAACGCGTCACCCGGCTTCGCATGAACCACGTCACAAGCATAATTGTGTGTTCTTCGTTTCCCCTCCCAAAGTACAAAATAGAAGGAGTGCAAAAAGTCAAATGCTCTAAGGTAATGCTATTTGACCACAACGTGCCATCGCGCGTAAGTCCAAGGGAATATAGATCTTCCCAGGAGTCTGCACAGGAGGCGAGTACAATCACGTCACTGACGCATAGTCAATTCGACCTAAGCGTTGATGGCGAGATACTGCCCGTCCCGTCAGACCTGGATGCTGACGCCCCAGCCCTAGAACCAGCACTAGACGACGGGGCGACACACACGCTGCCATCCACAACCGGAAACCTTGCGGCCGTGTCTGACTGGGTAATGAGCACCGTACCTGTCGCGCCGCCCAGAAGAAGGCGAGGGAGAAACCTGACTGTGACATGTGACGAGAGAGAAGGGAATATAACACCCATGGCTAGCGTCCGATTCTTTAGGGCAGAGCTGTGTCCGGTCGTACAAGAAACAGCGGAGACGCGTGACACAGCAATGTCTCTTCAGGCACCACCGAGTACCGCCACGGAACCGAATCATCCGCCGATCTCCTTCGGAGCATCAAGCGAGACGTTCCCCATTACATTTGGGGACTTCAACGAAGGAGAAATCGAAAGCTTGTCTTCTGAGCTACTAACTTTCGGAGACTTCTTACCAGGAGAAGTGGATGACTTGACAGACAGCGACTGGTCCACGTGCTCAGACACGGACGACGAGTTATGACTAGACAGGGCAGGTGGGTATATATTCTCGTCGGACACCGGTCCAGGTCATTTACAACAGAAGTCAGTACGCCAGTCAGTGCTGCCGGTGAACACCCTGGAGGAAGTCCACGAGGAGAAGTGTTACCCACCTAAGCTGGATGAAGCAAAGGAGCAACTATTACTTAAGAAACTCCAGGAGAGTGCATCCATGGCCAACAGAAGCAGGTATCAGTCGCGCAAAGTAGAAAACATGAAAGCAGCAATCATCCAGAGACTAAAGAGAGGCTGTAGACTATACTTAATGTCAGAGACCCCAAAAGTCCCTACTTACCGGACTACATATCCGGCGCCTGTGTACTCGCCTCCGATCAACGTCCGATTGTCCAATCCCGAGTCCGCAGTGGCAGCATGCAATGAGTTCTTAGCTAGAAACTATCCAACTGTCTCATCATACCAAATTACCGACGAGTATGATGCATATCTAGACATGGTGGACGGGTCGGAGAGTTGCCTGGACCGAGCGACATTCAATCCGTCAAAACTCAGGAGCTACCCGAAACAGCACGCTTACCACGCGCCCTCCATCAGAAGCGCTGTACCGTCCCCATTCCAGAACACACTACAGAATGTACTGGCAGCAGCCACGAAAAGAAACTGCAACGTCACACAGATGAGGGAATTACCCACTTTGGACTCAGCAGTATTCAACGTGGAGTGTTTCAAAAAATTCGCATGCAACCAAGAATACTGGGAAGAATTTGCTGCCAGCCCTATTAGGATAACAACTGAGAATTTAGCAACCTATGTTACTAAACTAAAAGGGCCAAAAGCAGCAGCGCTATTCGCAAAAACCCATAATCTACTGCCACTACAGGAAGTACCAATGGATAGGTTCACAGTAGATATGAAAAGGGACGTGAAGGTGACTCCTGGTACAAAGCATACAGAGGAAAGACCTAAGGTGCAGGTTATACAGGCGGCTGAACCCTTGGCGACAGCATACCTATGTGGGATTCACAGAGAGCTGGTTAGGAGGCTGAACGCCGTCCTCCTACCCAATGTACATACACTATTTGACATGTCTGCCGAGGATTTCGATGCCATCATAGCCGCACACTTTAAGCCAGGAGACACTGTTTTGGAAACGGACATAGCCTCCTTTGATAAGAGCCAAGATGATTCACTTGCGCTTACTGCTTTGATGCTGTTAGAGGATTTAGGGGTGGATCACTCCCTGCTGGACTTGATAGAGGCTGCTTTCGGAGAGATTTCCAGCTGTCACCTACCGACAGGTACGCGCTTCAAGTTCGGCGCCATGATGAAATCAGGTATGTTCCTAACTCTGTTCGTCAACACATTGTTAAACATCACCATCGCCAGCCGAGTGCTGGAAGATCGTCTGACAAAATCCGCGTGCGCGGCCTTCATCGGCGACGACAACATAATACATGGAGTCGTCTCCGATGAATTGATGGCAGCCAGATGTGCCACTTGGATGAACATGGAAGTGAAGATCATAGATGCAGTTGTATCCTTGAAAGCCCCTTACTTTTGTGGAGGGTTTATACTGCACGATACTGTGACAGGAACAGCTTGCAGAGTGGCAGACCCGCTAAAAAGGCTTTTTAAACTGGGCAAACCGCTAGCGGCAGGTGACGAACAAGATGAAGATAGAAGACGAGCGCTGGCTGACGAAGTGATCAGATGGCAACGAACAGGGCTAATTGATGAGCTGGAGAAAGCGGTATACTCTAGGTACGAAGTGCAGGGTATATCAGTTGTGGTAATGTCCATGGCCACCTTTGCAAGCTCCAGATCCAACTTCGAGAAGCTCAGAGGACCCGTCATAACTTTGTACGGCGGTCCTAAATAGGTACGCACTACAGCTACCTATTTTGCAGAAGCCGACAGCAAGTATCTAAACACTAATCAGCTACAATGGAGTTCATCCCAACCCAAACTTTTTACAATAGGAGGTACCAGCCTCGACCCTGGACTCCGCGCTCTACTATCCAAATCATCAGGCCCAGACCGCGCCCTCAGAGGCAAGCTGGGCAACTTGCCCAGCTGATCTCAGCAGTTAATAAACTGACAATGCGCGCGGTACCCCAACAGAAGCCACGCAGGAATCGGAAGAATAAGAAGCAAAAGCAAAAACAACAGGCGCCACAAAACAACACAAATCAAAAGAAGCAGCCACCTAAAAAGAAACCGGCTCAAAAGAAAAAGAAGCCGGGCCGCAGAGAGAGGATGTGCATGAAAATCGAAAATGATTGTATTTTCGAAGTCAAGCACGAAGGTAAGGTAACAGGTTACGCGTGCCTGGTGGGGGACAAAGTAATGAAACCAGCACACGTAAAGGGGACCATCGATAACGCGGACCTGGCCAAACTGGCCTTTAAGCGGTCATCTAAGTATGACCTTGAATGCGCGCAGATACCCGTGCACATGAAGTCCGACGCTTCGAAGTTCACCCATGAGAAACCGGAGGGGTACTACAACTGGCACCACGGAGCAGTACAGTACTCAGGAGGCCGGTTCACCATCCCTACAGGTGCTGGCAAACCAGGGGACAGCGGCAGACCGATCTTCGACAACAAGGGACGCGTGGTGGCCATAGTCTTAGGAGGAGCTAATGAAGGAGCCCGTACAGCCCTCTCGGTGGTGACCTGGAATAAAGACATTGTCACTAAAATCACCCCCGAGGGGGCCGAAGAGTGGAGTCTTGCCATCCCAGTTATGTGCCTGTTGGCAAACACCACGTTCCCCTGCTCCCAGCCCCCTTGCACGCCCTGCTGCTACGAAAAGGAACCGGAGGAAACCCTACGCATGCTTGAGGACAACGTCATGAGACCTGGGTACTATCAGCTGCTACAAGCATCCTTAACATGTTCTCCCCACCGCCAGCGACGCAGCACCAAGGACAACTTCAATGTCTATAAAGCCACAAGACCATACTTAGCTCACTGTCCCGACTGTGGAGAAGGGCACTCGTGCCATAGTCCCGTAGCACTAGAACGCATCAGAAATGAAGCGACAGACGGGACGCTGAAAATCCAGGTCTCCTTGCAAATCGGAATAAAGACGGATGACAGCCACGATTGGACCAAGCTGCGTTATATGGACAACCACATGCCAGCAGACGCAGAGAGGGCGGGGCTATTTGTAAGAACATCAGCACCGTGTACGATTACTGGAACAATGGGACACTTCATCCTGGCCCGATGTCCAAAAGGGGAAACTCTGACGGTGGGATTCACTGACAGTAGGAAGATTAGTCATTCATGTACGCACCCATTTCACCACGACCCTCCTGTGATAGGTCGGGAAAAATTCCATTCCCGACCGCAGCACGGTAAAGAGCTACCTTGCAGCACGTACGTGCAGAGCACCGCCGCAACTACCGAGGAGATAGAGGTACACATGCCCCCAGACACCCCTGATCGCACATTAATGTCACAACAGTCCGGCAACGTAAAGATCACAGTCAATGGCCAGACGGTGCGGTACAAGTGTAATTGCGGTGGCTCAAATGAAGGACTAACAACTACAGACAAAGTGATTAATAACTGCAAGGTTGATCAATGTCATGCCGCGGTCACCAATCACAAAAAGTGGCAGTATAACTCCCCTCTGGTCCCGCGTAATGCTGAACTTGGGGACCGAAAAGGAAAAATTCACATCCCGTTTCCGCTGGCAAATGTAACATGCAGGGTGCCTAAAGCAAGGAACCCCACCGTGACGTACGGGAAAAACCAAGTCATCATGCTACTGTATCCTGACCACCCAACACTCCTGTCCTACCGGAATATGGGAGAAGAACCAAACTATCTAGAAGAGTGGGTGATGCCTAAGAAGGAAGTCGTGCTAACCGTGCCGACTGAAGGGCTCGAGGTCACGTGGGGCAACAACGAGCCGTATAAGTATTGGCCGCAGTTATCTACAAACGGTACAGCCCATGGCCACCCGCATGAGATAATTCTGTATTATTATGAGCTGTACCCTACTATGACTGTAGTAGTTGTGTCAGTGGCCACGTTCATACTCCTGTCGATGGTGGGTATGGCAGCGGGGATGTGCATGTGTGCACGACGCAGATGCATCACACCGTATGAACTGACACCAGGAGCTACCGTCCCTTTCCTGCTTAGCCTAATATGCTGCATCAGAACAGCTAAAGCGGCCACATACCAAGAGGCTGCGATATACCTGTGGAACGAGCAGCAACCTTTGTTTTGGCTACAAGCCCTTATTCCGCTGGCAGCCCTGATTGTTCTATGCAACTGTCTGAGACTCTTACCATGCTGCTGTAAAACGTTGGCTTTTTTAGCCGTAATGAGCGTCGGTGCCCACACTGTGAGCGCGTACGAACACGTAACAGTGATCCCGAACACGGTGGGAGTACCGTATAAGACTCTAGTCAATAGACCTGGCTACAGCCCCATGGTATTGGAGATGGAACTACTGTCAGTCACTTTGGAGCCAACACTATCGCTTGATTACATCACGTGCGAGTACAAAACCGTCATCCCGTCTCCGTACGTGAAGTGCTGCGGTACAGCAGAGTGCAAGGACAAAAACCTACCTGACTACAGCTGTAAGGTCTTCACCGGCGTCTACCCATTTATGTGGGGCGGCGCCTACTGCTTCTGCGACGCTGAAAACACGCAGTTGAGCGAAGCACAGGTGGAGAAGTCCGAATCATGCAAAACAGAATTTGCATCAGCATACAGGGCTCATACCGCATCTGCATCAGCTAAGCTCCGCGTCCTTTACCAAGGAAATAACATCACTGTAACTGCCTATGCAAACGGCGACCATGCCGTCACAGTTAAGGACGCCAAATTCATTGTGGGGCCAATGTCTTCAGCCTGGACACCTTTCGACAACAAAATTGTGGTGTACAAAGGTGACGTCTATAACATGGACTACCCGCCCTTTGGCGCAGGAAGACCAGGACAATTTGGCGATATCCAAAGTCGCACACCTGAGAGTAAAGACGTCTATGCTAATACACAACTGGTACTGCAGAGACCGGCTGCGGGTACGGTACACGTGCCATACTCTCAGGCACCATCTGGCTTTAAGTATTGGCTAAAAGAACGCGGGGCGTCACTGCAGCACACAGCACCATTTGGCTGCCAAATAGCAACAAACCCGGTAAGAGCGGTGAACTGCGCCGTAGGGAACATGCCCATCTCCATCGACATACCGGAAGCGGCCTTCACTAGGGTCGTCGACGCGCCCTCTTTAACGGACATGTCGTGCGAGGTACCAGCCTGCACCCATTCCTCAGACTTTGGGGGCGTCGCCATTATTAAATATGCAGCCAGCAAGAAAGGCAAGTGTGCGGTGCATTCGATGACTAACGCCGTCACTATTCGGGAAGCTGAGATAGAAGTTGAAGGGAATTCTCAGCTGCAAATCTCTTTCTCGACGGCCTTAGCCAGCGCCGAATTCCGCGTACAAGTCTGTTCTACACAAGTACACTGTGCAGCTGAGTGCCACCCCCCGAAGGACCACATAGTCAACTACCCGGCGTCACATACCACCCTCGGGGTCCAGGACATCTCCGCTACGGCGATGTCATGGGTGCAGAAGATCACGGGAGGTGTGGGACTGGTTGTTGCTGTTGCCGCACTGATTCTAATCGTGGTGCTATGCGTGTCGTTCAGCAGGCACTAACTTGACAATTAAGTATGAAGGTATATGTGTCCCCTAAGAGACACACTGTACATAGCAAATAATCTATAGATCAAAGGGCTACGCAACCCCTGAATAGTAACAAAATACAAAATCACTAAAAATTATAAAAACAGAAAAATACATAAATAGGTATACGTGTCCCCTAAGAGACACATTGTATGTAGGTGATAAGTATAGATCAAAGGGCCGAATAACCCCTGAATAGTAACAAAATATGAAAATCAATAAAAATCATAAAATAGAAAAACCATAAACAGAAGTAGTTTAAAGGGCTATAAAACCCCTGAATAGTAACAAAACATAAAGTTAATAAAAATCAAATGAATACCATAATTGGCAAACGGAAGAGATGTAGGTACTTAAGCTTCCTAAAAGCAGCCGAACTCACTTTGAGAAGTAGGCATAGCATACCGAACTCTTCCACGATTCTCCGAACCCACAGGGACGTAGGAGATGTTATTTTGTTTTTAATATTTC

>FJ000067|2006|India: Maharashtra, Group 4

TTTGGCTGCGTGAGACACACGTAGCCTACCAGTTTCTTACTGCTCTACTCTGCAAAGCAAGAGATTAATAACCCATCATGGATCCTGTGTACGTGGACATAGACGCTGACAGCGCCTTTTTGAAGGCCCTGCAACGTGCGTACCCCATGTTTGAGGTGGAACCAAGGCAGGTCACACCGAATGACCATGCTAATGCTAGAGCGTTCTCGCATCTAGCTATAAAACTAATAGAGCAGGAAATTGACCCCGACTCAACCATCCTGGATATCGGCAGTGCGCCAGCAAGGAGGATGATGTCGGACAGGAAGTACCACTGCGTCTGCCCGATGCGCAGTGCGGAAGATCCCGAGAGACTCGCTAATTATGCGAGAAAGCTAGCATCTGCCGCAGGAAAAGTCCTGGACAGAAACATCTCTGGAAAGATCGGGGACTTACAAGCAGTAATGGCCGTGCCAGACAAGGAGACGCCAACATTCTGCTTACACACAGACGTCTCATGCAGACAGAGAGCAGACGTCGCTATATACCAAGACGTCTATGCTGTACACGCACCCACGTCGCTATACCACCAGGCGATTAAAGGGGTCCGAGTGGCGTACTGGGTTGGGTTCGACACAACCCCGTTCATGTACAATGCCATGGCGGGTGCCTACCCCTCATACTCGACAAACTGGGCAGATGAGCAGGTACTGAAGGCTAAGAACATAGGATTATGTTCAACAGACCTGACGGAAGGTAGACGAGGCAAGTTGTCTATTATGAGAGGGAAAAAGCTAAAACCGTGCGACCGTGTGCTGTTCTCAGTAGGGTCAACGCTCTACCCGGAAAGCCGCAAGCTACTTAAGAGCTGGCACCTGCCATCGGTGTTCCATTTAAAGGGCAAACTCAGCTTCACATGCCGCTGTGATACAGTGGTTTCGTGTGAGGGCTACGTCGTTAAGAGAATAACGATGAGCCCAGGCCTTTATGGAAAAACCACAGGGTATGCGGTAACCCACCACGCAGACGGATTCCTGATGTGCAAGACTACCGACACGGTTGACGGCGAAAGAGTGTCATTCTCGGTGTGCACATACGTGCCGGCGACCATTTGTGATCAAATGACCGGCATCCTTGCTACAGAAGTCACGCCGGAGGATGCACAGAAGCTGTTGGTGGGGCTGAACCAGAGAATAGTGGTTAACGGCAGAACGCAACGGAATATGAACACCATGAAAAATTATCTGCTTCCCGTGGTCGCCCAAGCCCTCAGTAAGTGGGCAAAGGAGTGCCGGAAAGACATGGAAGATGAAAAACTCCTGGGGGTCAGAGAAAGAACACTGACCTGCTGCTGTCTATGGGCATTCAAGAAGCAGAAAACACACACGGTCTACAAGAGGCCTGATACCCAGTCAATTCAGAAGGTTCAGGCCGAGTTTGACAGCTTTGTGGTACCGAGTCTGTGGTCGTCCGGGTTGTCAATCCCTTTGAGGACTAGAATCAAATGGTTGTTAAGCAAGGTGCCAAAAACCGACCTGATCCCATACAGCGGAGACGCCCGAGAAGCCCGGGACGCAGAAAAAGAAGCAGAGGAAGAACGAGAAGCAGAACTGACTCGCGAAGCCCTACCACCTCTACAGGCAGCACAGGAAGATGTTCAGGTCGAAATCGACGTGGAACAGCTTGAGGACAGAGCGGGCGCAGGAATAATAGAGACTCCGAGAGGAGCTATCAAAGTTACTGCCCAACCAACAGACCACGTCGTGGGAGAGTACCTGGTACTCTCCCCGCAGACCGTACTACGTAGCCAGAAGCTCAGTCTGATTCACGCTTTGGCGGAGCAAGTGAAGACGTGCACGCACAACGGACGAGCAGGGAGGTATGCGGTCGAAGCGTACGACGGCCGAGTCCTAGTGCCCTCAGGCTATGCAATCTCGCCTGAAGACTTCCAGAGTCTAAGCGAAAGCGCAACGATGGTGTATAACGAAAGAGAGTTCGTAAACAGAAAGCTACACCATATTGCGATGCACGGACCAGCCCTGAACACCGACGAAGAGTCGTATGAGCTGGTGAGGGCAGAGAGGACAGAACACGAGTACGTCTACGACGTGGATCAGAGAAGATGCTGTAAGAAGGAAGAAGCCGCAGGACTGGTACTGGTGGGCGACTTGACTAATCCGCCCTACCACGAATTCGCATATGAAGGGCTAAAAATCCGCCCTGCCTGCCCATACAAAATTGCAGTCATAGGAGTCTTCGGAGTACCGGGATCTGGCAAGTCAGCTATTATCAAGAACCTAGTTACCAGGCAGGACCTGGTGACTAGCGGAAAGAAAGAAAACTGCCAAGAAATCACCACCGACGTGATGAGACAGAGAGGTCTAGAGATATCTGCACGTACGGTTGACTCGCTGCTCTTGAATGGATGCAACAGACCAGTCGACGTGTTGTACGTAGACGAGGCGTTTGCGTGCCACTCTGGAACGCTACTTGCTTTGATCGCCTTGGTGAGACCAAGGCAGAAAGTTGTACTTTGTGGTGACCCGAAGCAGTGCGGCTTCTTCAATATGATGCAGATGAAAGTCAACTATAATCACAACATCTGCACCCAAGTGTACCACAAAAGTATCTCCAGGCGGTGTACACTGCCTGTGACCGCCATTGTGTCATCGTTGCATTACGAAGGCAAAATGCGCACTACGAATGAGTACAACAAGCCGATTGTAGTGGACACTACAGGCTCAACAAAACCTGACCCTGGAGACCTCGTGTTAACGTGCTTCAGAGGGTGGGTTAAACAACTGCAAATTGACTATCGTGGATACGAGGTCATGACAGCAGCCGCATCCCAAGGGTTAACCAGAAAAGGAGTTTACGCAGTTAGACAAAAAGTTAATGAAAACCCGCTCTATGCATCAACGTCAGAGCACGTCAACGTACTCCTAACGCGTACGGAAGGTAAACTGGTATGGAAGACACTTTCCGGCGACCCGTGGATAAAGACGCTGCAGAACCCACCGAAAGGAAACTTCAAAGCAACTATTAAGGAGTGGGAGGTGGAGCATGCATCAATAATGGCGGGCATCTGCAGTCACCAAATGACCTTCGATACATTCCAAAATAAAGCCAACGTTTGTTGGGCTAAGAGCTTGGTCCCTATCCTCGAAACAGCGGGGATAAAACTAAATGATAGGCAGTGGTCTCAGATAATTCAAGCCTTCAAAGAAGACAAAGCATACTCACCTGAAGTAGCCCTGAATGAAATATGTACGCGCATGTATGGGGTGGATCTAGACAGCGGGCTATTTTCTAAACCGTTGGTGTCTGTGTATTACGCGGATAACCACTGGGATAATAGGCCTGGAGGGAAAATGTTCGGATTTAACCCCGAGGCAGCATCCATTCTAGAAAGAAAGTATCCATTCACAAAAGGGAAGTGGAACATCAACAAGCAGATCTGCGTGACTACCAGGAGGATAGAAGACTTTAACCCTACCACCAACATCATACCGGCCAACAGGAGACTACCACACTCATTAGTGGCCGAACACCGCCCAGTAAAAGGGGAAAGAATGGAATGGCTGGTTAACAAGATAAACGGCCACCACGTGCTCCTGGTCAGTGGCTATAACCTTGCACTGCCTACTAAGAGAGTCACTTGGGTAGCGCCGTTAGGTGTCCGCGGAGCGGACTACACATACAACCTAGAGTTGGGTCTGCCAGCAACGCTTGGTAGGTATGACCTTGTGGTCATAAACATCCACACACCTTTTCGCATACACCATTACCAACAGTGCGTCGACCACGCAATGAAACTGCAAATGCTCGGGGGTGACTCATTGAGACTGCTCAAACCGGGCGGCTCTCTATTGATCAGAGCATATGGTTACGCAGATAGAACCAGTGAACGAGTCATCTGCGTATTGGGACGCAAGTTTAGATCGTCTAGAGCGTTGAAACCACCATGTGTCACCAGCAACACTGAGATGTTTTTCCTATTCAGCAACTTTGACAATGGCAGAAGGAATTTCACAACTCATGTCATGAACAATCAACTGAATGCAGCCTTCGTAGGACAGGTCACCCGAGCAGGATGTGCACCGTCGTACCGGGTAAAACGCATGGACATCGCGAAGAACGATGAAGAGTGCGTAGTCAACGCCGCTAACCCTCGCGGGTTACCGGGTGACGGTGTTTGCAAGGCAGTATACAAAAAATGGCCGGAGTCCTTTAAGAACAGTGCAACACCAGTGGGAACCGCAAAAACAGTTATGTGCGGTACGTATCCAGTAATCCACGCTGTTGGACCAAACTTCTCTAACTATTCGGAGTCTGAAGGGGACCGGGAATTGGCAGCTGCCTATCGAGAAGTCGCAAAGGAAGTAACTAGGCTGGGAGTAAATAGTGTAGCTATACCTCTCCTCTCCACAGGTGTATACTCAGGAGGGAAAGACAGGCTGACCCAGTCACTGAACCACCTCTTTACAGCCATGGACTCGACGGATGCAGACGTGGTCATCTACTGCCGCGACAAAGAATGGGAGAAGAAAATATCTGAGGCCATACAGATGCGGACCCAAGTAGAGCTGCTGGATGAGCACATCTCCATAGACTGCGATATTGTTCGCGTGCACCCTGACAGCAGCTTGGCAGGCAGAAAAGGATACAGCACCACGGAAGGCGCACTGTACTCATATCTAGAAGGGACCCGTTTTCATCAGACGGCTGTGGATATGGCGGAGATACATACTATGTGGCCAAAGCAAACAGAGGCCAATGAGCAAGTCTGCCTATATGCCCTGGGGGAAAGTATTGAATCGATCAGGCAGAAATGCCCGGTGGATGATGCAGACGCATCATCTCCCCCCAAAACTGTCCCGTGCCTTTGCCGTTACGCTATGACTCCAGAACGCGTCACCCGGCTTCGCATGAACCACGTCACAAGCATAATTGTGTGTTCTTCGTTTCCCCTCCCAAAGTACAAAATAGAAGGAGTGCAAAAAGTCAAATGCTCTAAGGTAATGCTATTTGACCACAACGTGCCATCGCGCGTAAGTCCAAGGGAATATAGATCTTCCCAGGAGTCTGCACAGGAGGCGAGTACAATCACGTCACTGACGCATAGTCAATTCGACCTAAGCGTTGATGGCGAGATACTGCCCGTCCCGTCAGACCTGGATGCTGACGCCCCAGCCCTAGAACCAGCACTAGACGACGGGGCGACACACACGCTGCCATCCACAACCGGAAACCTTGCGGCCGTGTCTGACTGGGTAATGAGCACCGTACCTGTCGCGCCGCCCAGAAGAAGGCGAGGGAGAAACCTGACTGTGACATGTGACGAGAGAGAAGGGAATATAACACCCATGGCTAGCGTCCGATTCTTTAGGGCAGAGCTGTGTCCGGTCGTACAAGAAACAGCGGAGACGCGTGACACAGCAATGTCTCTTCAGGCACCACCGAGTACCGCCACGGAACCGAATCATCCGCCGATCTCCTTCGGAGCATCAAGCGAGACGTTCCCCATTACATTTGGGGACTTCAACGAAGGAGAAATCGAAAGCTTGTCTTCTGAGCTACTAACTTTCGGAGACTTCTTACCAGGAGAAGTGGATGACTTGACAGACAGCGACTGGTCCACGTGCTCAGACACGGACGACGAGTTATGACTAGACAGGGCAGGTGGGTATATATTCTCGTCGGACACCGGTCCAGGTCATTTACAACAGAAGTCAGTACGCCAGTCAGTGCTGCCGGTGAACACCCTGGAGGAAGTCCACGAGGAGAAGTGTTACCCACCTAAGCTGGATGAAGCAAAGGAGCAACTATTACTTAAGAAACTCCAGGAGAGTGCATCCATGGCCAACAGAAGCAGGTATCAGTCGCGCAAAGTAGAAAACATGAAAGCAGCAATCATCCAGAGACTAAAGAGAGGCTGTAGACTATACTTAATGTCAGAGACCCCAAAAGTCCCTACTTACCGGACTACATATCCGGCGCCTGTGTACTCGCCTCCGATCAACGTCCGATTGTCCAATCCCGAGTCCGCAGTGGCAGCATGCAATGAGTTCTTAGCTAGAAACTATCCAACTGTCTCATCATACCAAATTACCGACGAGTATGATGCATATCTAGACATGGTGGACGGGTCGGAGAGTTGCCTGGACCGAGCGACATTCAATCCGTCAAAACTCAGGAGCTACCCGAAACAGCACGCTTACCACGCGCCCTCCATCAGAAGCGCTGTACCGTCCCCATTCCAGAACACACTACAGAATGTACTGGCAGCAGCCACGAAAAGAAACTGCAACGTCACACAGATGAGGGAATTACCCACTTTGGACTCAGCAGTATTCAACGTGGAGTGTTTCAAAAAATTCGCATGCAACCAAGAATACTGGGAAGAATTTGCTGCCAGCCCTATTAGGATAACAACTGAGAATTTAGCAACCTATGTTACTAAACTAAAAGGGCCAAAAGCAGCAGCGCTATTCGCAAAAACCCATAATCTACTGCCACTACAGGAAGTACCAATGGATAGGTTCACAGTAGATATGAAAAGGGACGTGAAGGTGACTCCTGGTACAAAGCATACAGAGGAAAGACCTAAGGTGCAGGTTATACAGGCGGCTGAACCCTTGGCGACAGCATACCTATGTGGGATTCACAGAGAGCTGGTTAGGAGGCTGAACGCCGTCCTCCTACCCAATGTACATACACTATTTGACATGTCTGCCGAGGATTTCGATGCCATCATAGCCGCACACTTTAAGCCAGGAGACACTGTTTTGGAAACGGACATAGCCTCCTTTGATAAGAGCCAAGATGATTCACTTGCGCTTACTGCTTTGATGCTGTTAGAGGATTTAGGGGTGGATCACTCCCTGCTGGACTTGATAGAGGCTGCTTTCGGAGAGATTTCCAGCTGTCACCTACCGACAGGTACGCGCTTCAAGTTCGGCGCCATGATGAAATCAGGTATGTTCCTAACTCTGTTCGTCAACACATTGTTAAACATCACCATCGCCAGCCGAGTGCTGGAAGATCGTCTGACAAAATCCGCGTGCGCGGCCTTCATCGGCGACGACAACATAATACATGGAGTCGTCTCCGATGAATTGATGGCAGCCAGATGTGCCACTTGGATGAACATGGAAGTGAAGATCATAGATGCAGTTGTATCCTTGAAAGCCCCTTACTTTTGTGGAGGGTTTATACTGCACGATACTGTGACAGGAACAGCTTGCAGAGTGGCAGACCCGCTAAAAAGGCTTTTTAAACTGGGCAAACCGCTAGCGGCAGGTGACGAACAAGATGAAGATAGAAGACGAGCGCTGGCTGACGAAGTGATCAGATGGCAACGAACAGGGCTAATTGATGAGCTGGAGAAAGCGGTATACTCTAGGTACGAAGTGCAGGGTATATCAGTTGTGGTAATGTCCATGGCCACCTTTGCAAGCTCCAGATCCAACTTCGAGAAGCTCAGAGGACCCGTCATAACTTTGTACGGCGGTCCTAAATAGGTACGCACTACAGCTACCTATTTTGCAGAAGCCGACAGCAAGTATCTAAACACTAATCAGCTACAATGGAGTTCATCCCAACCCAAACTTTTTACAATAGGAGGTACCAGCCTCGACCCTGGACTCCGCGCTCTACTATCCAAGTCATCAGGCCCAGACCGCGCCCTCAGAGGCAAGCTGGGCAACTTGCCCAGCTGATCTCAGCAGTTAATAAACTGACAATGCGCGCGGTACCCCAACAGAAGCCACGCAGGAATCGGAAGAATAAGAAGCAAAAGCAAAAACAACAGGCGCCACAAAACAACACAAATCAAAAGAAGCAGCCACCTAAAAAGAAACCGGCTCAAAAGAAAAAGAAGCCGGGCCGCAGAGAGAGGATGTGCATGAAAATCGAAAATGATTGTATTTTCGAAGTCAAGCACGAAGGTAAGGTAACAGGTTACGCGTGCCTGGTGGGGGACAAAGTAATGAAACCAGCACACGTAAAGGGGACCATCGATAACGCGGACCTGGCCAAACTGGCCTTTAAGCGGTCATCTAAGTATGACCTTGAATGCGCGCAGATACCCGTGCACATGAAGTCCGACGCTTCGAAGTTCACCCATGAGAAACCGGAGGGGTACTACAACTGGCACCACGGAGCAGTACAGTACTCAGGAGGCCGGTTCACCATCCCTACAGGTGCTGGCAAACCAGGGGACAGCGGCAGACCGATCTTCGACAACAAGGGACGCGTGGTGGCCATAGTCTTAGGAGGAGCTAATGAAGGAGCCCGTACAGCCCTCTCGGTGGTGACCTGGAATAAAGACATTGTCACTAAAATCACCCCCGAGGGGGCCGAAGAGTGGAGTCTTGCCATCCCAGTTATGTGCCTGTTGGCAAACACCACGTTCCCCTGCTCCCAGCCCCCTTGCACGCCCTGCTGCTACGAAAAGGAACCGGAGGAAACCCTACGCATGCTTGAGGACAACGTCATGAGACCTGGGTACTATCAGCTGCTACAAGCATCCTTAACATGTTCTCCCCACCGCCAGCGACGCAGCACCAAGGACAACTTCAATGTCTATAAAGCCACAAGACCATACTTAGCTCACTGTCCCGACTGTGGAGAAGGGCACTCGTGCCATAGTCCCGTAGCACTAGAACGCATCAGAAATGAAGCGACAGACGGGACGCTGAAAATCCAGGTCTCCTTGCAAATCGGAATAAAGACGGATGACAGCCACGATTGGACCAAGCTGCGTTATATGGACAACCACATGCCAGCAGACGCAGAGAGGGCGGGGCTATTTGTAAGAACATCAGCACCGTGTACGATTACTGGAACAATGGGACACTTCATCCTGGCCCGATGTCCAAAAGGGGAAACTCTGACGGTGGGATTCACTGACAGTAGGAAGATTAGTCATTCATGTACGCACCCATTTCACCACGACCCTCCTGTGATAGGTCGGGAAAAATTCCATTCCCGACCGCAGCACGGTAAAGAGCTACCTTGCAGCACGTACGTGCAGAGCACCGCCGCAACTACCGAGGAGATAGAGGTACACATGCCCCCAGACACCCCTGATCGCACATTAATGTCACAACAGTCCGGCAACGTAAAGATCACAGTCAATGGCCAGACGGTGCGGTACAAGTGTAATTGCGGTGGCTCAAATGAAGGACTAACAACTACAGACAAAGTGATTAATAACTGCAAGGTAGATCAATGTCATGCCGCGGTCACCAATCACAAAAAGTGGCAGTATAACTCCCCTCTGGTCCCGCGTAATGCTGAACTTGGGGACCGAAAAGGAAAAATTCACATCCCGTTTCCGCTGGCAAATGTAACATGCAGGGTGCCTAAAGCAAGGAACCCCACCGTGACGTACGGGAAAAACCAAGTCATCATGCTACTGTATCCTGACCACCCAACACTCCTGTCCTACCGGAATATGGGAGAAGAACCAAACTATCAAGAAGAGTGGGTGATGCATAAGAAGGAAGTCGTGCTAACCGTGCCGACTGAAGGGCTCGAGGTCACGTGGGGCAACAACGAGCCGTATAAGTATTGGCCGCAGTTATCTACAAACGGTACAGCCCATGGCCACCCGCATGAGATAATTCTGTATTATTATGAGCTGTACCCTACTATGACTGTAGTAGTTGTGTCAGTGGCCACGTTCATACTCCTGTCGATGGTGGGTATGGCAGCGGGGATGTGCATGTGTGCACGACGCAGATGCATCACACCGTATGAACTGACACCAGGAGCTACCGTCCCTTTCCTGCTTAGCCTAATATGCTGCATCAGAACAGCTAAAGCGGCCACATACCAAGAGGCTGCGATATACCTGTGGAACGAGCAGCAACCTTTGTTTTGGCTACAAGCCCTTATTCCGCTGGCAGCCCTGATTGTTCTATGCAACTGTCTGAGACTCTTACCATGCTGCTGTAAAACGTTGGCTTTTTTAGCCGTAATGAGCGTCGGTGCCCACACTGTGAGCGCGTACGAACACGTAACAGTGATCCCGAACACGGTGGGAGTACCGTATAAGACTCTAGTCAATAGACCTGGCTACAGCCCCATGGTATTGGAGATGGAACTACTGTCAGTCACTTTGGAGCCAACACTATCGCTTGATTACATCACGTGCGAGTACAAAACCGTCATCCCGTCTCCGTACGTGAAGTGCTGCGGTACAGCAGAGTGCAAGGACAAAAACCTACCTGACTACAGCTGTAAGGTCTTCACCGGCGTCTACCCATTTATGTGGGGCGGCGCCTACTGCTTCTGCGACGCTGAAAACACGCAGTTGAGCGAAGCACACGTGGAGAAGTCCGAATCATGCAAAACAGAATTTGCATCAGCATACAGGGCTCATACCGCATCTGCATCAGCTAAGCTCCGCGTCCTTTACCAAGGAAATAACATCACTGTAACTGCCTATGCAAACGGCGACCATGCCGTCACAGTTAAGGACGCCAAATTCATTGTGGGGCCAATGTCTTCAGCCTGGACACCTTTCGACAACAAAATTGTGGTGTACAAAGGTGACGTCTATAACATGGACTACCCGCCCTTTGGCGCAGGAAGACCAGGACAATTTGGCGATATCCAAAGTCGCACACCTGAGAGTAAAGACGTCTATGCTAATACACAACTGGTACTGCAGAGACCGGCTGCGGGTACGGTACACGTGCCATACTCTCAGGCACCATCTGGCTTTAAGTATTGGCTAAAAGAACGCGGGGCGTCACTGCAGCACACAGCACCATTTGGCTGCCAAATAGCAACAAACCCGGTAAGAGCGGTGAACTGCGCCGTAGGGAACATGCCCATCTCCATCGACATACCGGAAGCGGCCTTCACTAGGGTCGTCGACGCGCCCTCTTTAACGGACATGTCGTGCGAGGTACCAGCCTGCACCCATTCCTCAGACTTTGGGGGCGTCGCCATTATTAAATATGCAGCCAGCAAGAAAGGCAAGTGTGCGGTGCATTCGATGACTAACGCCGTCACTATTCGGGAAGCTGAGATAGAAGTTGAAGGGAATTCTCAGCTGCAAATCTCTTTCTCGACGGCCTTAGCCAGCGCCGAATTCCGCGTACAAGTCTGTTCTACACAAGTACACTGTGCAGCTGAGTGCCACCCCCCGAAGGACCACATAGTCAACTACCCGGCGTCACATACCACCCTCGGGGTCCAGGACATCTCCGCTACGGCGATGTCATGGGTGCAGAAGATCACGGGAGGTGTGGGACTGGTTGTTGCTGTTGCCGCACTGATTCTAATCGTGGTGCTATGCGTGTCGTTCAGCAGGCACTAACTTGACAATTAAGTATGAAGGTATATGTGTCCCCTAAGAGACACACTGTACATAGCAAATAATCTATAGATCAAAGGGCTACGCAACCCCTGAATAGTAACAAAATACAAAATCACTAAAAATTATAAAAACAGAAAAATACATAAATAGGTATACGTGTCCCCTAAGAGACACATTGTATGTAGGTGATAAGTATAGATCAAAGGGCCGAATAACCCCTGAATAGTAACAAAATATGAAAATCAATAAAAATCATAAAATAGAAAAACCATAAACAGAAGTAGTTCAAAGGGCTATAAAACCCCTGAATAGTAACAAAACATAAAATTAATAAAAATCAAATGAATACCATAATTGGCAAACGGAAGAGATGTAGGTACTTAAGCTTCCTAAAAGCAGCCGAACTCACTTTGAGAAGTAGGCATAGCATACCGAACTCTTCCACGATTCTCCGAACCCACAGGGACGTAGGAGATGTTATTTTGTTTTTAATATTTC

>FJ000068|2006|India: Karnataka, Group 4

TTTGGCTGCGTGAGACACACGTAGCCTACCAGTTTCTTACTGCTCTACTCTGCAAAGCAAGAGATTAATAACCCATCATGGATCCTGTGTACGTGGACATAGACGCTGACAGCGCCTTTTTGAAGGCCCTGCAACGTGCGTACCCCATGTTTGAGGTGGAACCAAGGCAGGTCACACCGAATGACCATGCTAATGCTAGAGCGTTCTCGCATCTAGCTATAAAACTAATAGAGCAGGAAATTGACCCCGACTCAACCATCCTGGATATCGGCAGTGCGCCAGCAAGGAGGATGATGTCGGACAGGAAGTACCACTGCGTCTGCCCGATGCGCAGTGCGGAAGATCCCGAGAGACTCGCTAATTATGCGAGAAAGCTAGCATCTGTCGCAGGAAAAGTCCTGGACAGAAACATCTCTGGAAAGATCGGGGACTTACAAGCAGTGATGGCCGTGCCAGACAAGGAGACGCCAACATTCTGCTTACACACAGACGTCTCATGTAGACAGAGAGCAGACGTCGCTATATACCAAGACGTCTATGCTGTACACGCACCCACGTCGCTATACCACCAGGCGATTAAAGGGGTCCGAGTGGCGTACTGGGTTGGGTTCGACACAACCCCGTTCATGTACAATGCCATGGCGGGTGCCTACCCCTCATACTCGACAAACTGGGCAGATGAGCAGGTACTGAAGGCTAAGAACATAGGATTATGTTCAACAGACCTGACGGAAGGTAGACGAGGCAAGTTGTCTATTATGAGAGGGAAAAAGCTAAAACCGTGCGACCGTGTGCTGTTCTCAGTAGGGTCAACGCTCTACCCGGAAAGCCGCAAGCTACTTAAGAGCTGGCACCTGCCATCGGTGTTCCATTTAAAGGGCAAACTCAGCTTCACATGCCGCTGTGATACAGTGGTTTCGTGTGAGGGCTACGTCGTTAAGAGAATAACGATGAGCCCAGGCCTTTATGGAAAAACCACAGGGTATGCGGTAACCCACCACGCAGACGGATTCCTGATGTGCAAGACTACCGACACGGTTGACGGCGAAAGAGTGTCATTCTCGGTGTGCACATACGTGCCGGCGACCATTTGTGATCAAATGACCGGCATCCTTGCTACAGAAGTCACGCCGGAGGATGCACAGAAGCTGTTGGTGGGGCTGAACCAGAGAATAGTGGTTAACGGCAGAACGCAACGGAATATGAACACCATGAAAAATTATCTGCTTCCCGTGGTCGCCCAAGCCTTCAGTAAGTGGGCAAAGGAGTGCCGGAAAGACATGGAAGATGAAAAACTCCTGGGGGTCAGAGAAAGAACACTGACCTGCTGCTGTCTATGGGCATTCAAGAAGCAGAAAACACACACGGTCTACAAGAGGCCTGATACCCAGTCAATTCAGAAGGTTCAGGCCGAGTTTGACAGCTTTGTGGTACCGAGTCTGTGGTCGTCCGGGTTGTCAATCCCTTTGAGGACTAGAATCAAATGGTTGTTAAGCAAGGTGCCAAAAACCGACCTGATCCCATACAGCGGAGACGCCCGAGAAGCCCGGGACGCAGAAAAAGAAGCAGAGGAAGAACGAGAAGCAGAACTGACTCGCGAAGCCCTACCACCTCTACAGGCAGCACAGGAAGATGTTCAGGTCGAAATCGACGTGGAACAGCTTGAGGACAGAGCGGGCGCAGGAATAATAGAGACTCCGAGAGGAGCTATCAAAGTTACTGCCCAACCAACAGACCACGTCGTGGGAGAGTACCTGGTACTCTCCCCGCAGACCGTACTACGTAGCCAGAAGCTCAGTCTGATTCACGCTTTGGCGGAGCAAGTGAAGACGTGCACGCACAACGGACGAGCAGGGAGGTATGCGGTCGAAGCGTACGACGGCCGAGTCCTAGTGCCCTCAGGCTATGCAATCTCGCCTGAAGACTTCCAGAGTCTAAGCGAAAGCGCAACGATGGTGTATAACGAAAGAGAGTTCGTAAACAGAAAGCTACACCATATTGCGATGCACGGACCAGCCCTGAACACCGACGAAGAGTCGTATGAGCTGGTGAGGGCAGAGAGGACAGAACACGAGTACGTCTACGACGTGGATCAGAGAAGATGCTGTAAGAAGGAAGAAGCCGCAGGACTGGTACTGGTGGGCGACTTGACTAATCCGCCCTACCACGAATTCGCATATGAAGGGCTAAAAATCCGCCCTGCCTGCCCATACAAAATTGCAGTCATAGGAGTCTTCGGAGTACCGGGATCTGGCAAGTCAGCTATTATCAAGAACCTAGTTACCAGGCAGGACCTGGTGACTAGCGGAAAGAAAGAAAACTGCCAAGAAATCACCACCGACGTGATGAGACAGAGAGGTCTAGAGATATCTGCACGTACGGTTGACTCGCTGCTCTTGAATGGATGCAACAGACCAGTCGACGTGTTGTACGTAGACGAGGCGTTTGCGTGCCACTCTGGAACGCTACTTGCTTTGATCGCCTTGGTGAGACCAAGGCAGAAAGTTGTACTTTGTGGTGACCCGAAGCAGTGCGGCTTCTTCAATATGATGCAGATGAAAGTCAACTATAATCACAACATCTGCACCCAAGTGTACCACAAAAGTATCTCCAGGCGGTGTACACTGCCTGTGACCGCCATTGTGTCATCGTTGCATTACGAAGGCAAAATGCGCACTACGAATGAGTACAACAAGCCGATTGTAGTGGACACTACAGGCTCAACAAAACCTGACCCTGGAGACCTCGTGTTAACGTGCTTCAGAGGGTGGGTTAAACAACTGCAAATTGACTATCGTGGATACGAGGTCATGACAGCAGCCGCATCCCAAGGGTTAACCAGAAAAGGAGTTTACGCAGTTAGACAAAAAGTTAATGAAAACCCGCTCTATGCATCAACGTCAGAGCACGTCAACGTACTCCTAACGCGTACGGAAGGTAAACTGGTATGGAAGACACTTTCCGGCGACCCGTGGATAAAGACGCTGCAGAACCCACCGAAAGGAAACTTCAAAGCAACTATTAAGGAGTGGGAGGTGGAGCATGCATCAATAATGGCGGGCATCTGCAGTCACCAAATGACCTTCGATACATTCCAAAATAAAGCCAACGTTTGTTGGGCTAAGAGCTTGGTCCCTATCCTCGAAACAGCGGGGATAAAACTAAATGATAGGCAGTGGTCTCAGATAATTCAAGCCTTCAAAGAAGACAAAGCATACTCACCTGAAGTAGCCCTGAATGAAATATGTACGCGCATGTATGGGGTGGATCTAGACAGCGGGCTATTTTCTAAACCGTTGGTGTCTGTGTATTACGCGGATAACCACTGGGATAATAGGCCTGGAGGGAAAATGTTCGGATTTAACCCCGAGGCAGCATCCATTCTAGAAAGAAAGTATCCATTCACAAAAGGGAAGTGGAACATCAACAAGCAGATCTGCGTGACTACCAGGAGGATAGAAGACTTTAACCCTACCACCAACATCATACCGGCCAACAGGAGACTACCACACTCATTAGTGGCCGAACACCGCCCAGTAAAAGGGGAAAGAATGGAATGGCTGGTTAACAAGATAAACGGCCACCACGTGCTCCTGGTCAGTGGCTATAACCTTGCACTGCCTACTAAGAGAGTCACTTGGGTAGCGCCGTTAGGTGTCCGCGGAGCGGACTACACATACAACCTAGAGTTGGGTCTGCCAGCAACGCTTGGTAGGTATGACCTTGTGGTCATAAACATCCACACACCTTTTCGCATACACCATTACCAACAGTGCGTCGACCACGCAATGAAACTGCAAATGCTCGGGGGTGACTCATTGAGACTGCTCAAACCGGGCGGCTCTCTATTGATCAGAGCATATGGTTACGCAGATAGAACCAGTGAACGAGTCATCTGCGTATTGGGACGCAAGTTTAGATCGTCTAGAGCGTTGAAACCACCATGTGTCACCAGCAACACTGAGATGTTTTTCCTATTCAGCAACTTTGACAATGGCAGAAGGAATTTCACAACTCATGTCATGAACAATCAACTGAATGCAGCCTTCGTAGGACAGGTCACCCGAGCAGGATGTGCACCGTCGTACCGGGTAAAACGCATGGACATCGCGAAGAACGATGAAGAGTGCGTAGTCAACGCCGCTAACCCTCGCGGGTTACCGGGTGACGGTGTTTGCAAGGCAGTATACAAAAAATGGCCGGAGTCCTTTAAGAACAGTGCAACACCAGTGGGAACCGCAAAAACAGTTATGTGCGGTACGTATCCAGTAATCCACGCTGTTGGACCAAACTTCTCTAATTATTCGGAGTCTGAAGGGGACCGGGAATTGGCAGCTGCCTATCGAGAAGTCGCAAAGGAAGTAACTAGGCTGGGAGTAAATAGTGTAGCTATACCTCTCCTCTCCACAGGTGTATACTCAGGAGGGAAAGACAGGCTGACCCAGTCACTGAACCACCTCTTTACAGCCATGGACTCGACGGATGCAGACGTGGTCATCTACTGCCGCGACAAAGAATGGGAGAAGAAAATATCTGAGGCCATACAGATGCGGACCCAAGTAGAGCTGCTGGATGAGCACATCTCCATAGACTGCGATATTGTTCGCGTGCACCCTGACAGCAGCTTGGCAGGCAGAAAAGGATACAGCACCACGGAAGGCGCACTGTACTCATATCTAGAAGGGACCCGTTTTCATCAGACGGCTGTGGATATGGCGGAGATACATACTATGTGGCCAAAGCAAACAGAGGCCAATGAGCAAGTCTGCCTATATGCCCTGGGGGAAAGTATTGAATCGATCAGGCAGAAATGCCCGGTGGATGATGCAGACGCATCATCTCCCCCCAATACTGTCCCGTGCCTTTGCCGTTACGCTATGACTCCAGAACGCGTCACCCGGCTTCGCATGAACCACGTCACAAGCATAATTGTGTGTTCTTCGTTTCCCCTCCCAAAGTACAAAATAGAAGGAGTGCAAAAAGTCAAATGCTCTAAGGTAATGCTATTTGACCACAACGTGCCATCGCGCGTAAGTCCAAGGGAATATAGATCTTCCCAGGAGTCTGCACAGGAGGCGAGTACAATCACGTCACTGACGCATAGTCAATTCGACCTAAGCGTTGATGGCGAGATACTGCCCGTCCCGTCAGACCTGGATGCTGACGCCCCAGCCCTAGAACCAGCACTAGACGACGGGGCGACACACACGCTGCCATCCACAACCGGAAACCTTGCGGCCGTGTCTGACTGGGTAATGAGCACCGTACCTGTCGCGCCGCCCAGAAGAAGGCGAGGGAGAAACCTGACTGTGACATGTGACGAGAGAGAAGGGAATATAACACCCATGGCTAGCGTCCGATTCTTTAGGGCAGAGCTGTGTCCGGTCGTACAAGAAACAGCGGAGACGCGTGACACAGCAATGTCTCTTCAGGCACCACCGAGTACCGCCACGGAACCGAATCATCCGCCGATCTCCTTCGGAGCATCAAGCGAGACGTTCCCCATTACATTTGGGGACTTCAACGAAGGAGAAATCGAAAGCTTGTCTTCTGAGCTACTAACTTTCGGAGACTTCTCACCAGGAGAAGTGGATGACTTGACAGACAGCGACTGGTCCACGTGCTCAGACACGGACGACGAGTTATGACTAGACAGGGCAGGTGGGTATATATTCTCGTCGGACACCGGTCCAGGTCATTTACAACAGAAGTCAGTACGCCAGTCAGTGCTGCCGGTGAACACCCTGGAGGAAGTCCACGAGGAGAAGTGTTACCCACCTAAGCTGGATGAAGCAAAGGAGCAACTATTACTTAAGAAACTCCAGGAGAGTGCATCCATGGCCAACAGAAGCAGGTATCAGTCGCGCAAAGTAGAAAACATGAAAGCAGCAATCATCCAGAGACTAAAGAGAGGCTGTAGACTATACTTAATGTCAGAGACCCCAAAAGTCCCTACTTACCGGACTACATATCCGGCGCCTGTGTACTCGCCTCCGATCAACGTCCGATTGTCCAATCCCGAGTCCGCAGTGGCAGCATGCAATGAGTTCTTAGCTAGAAACTATCCAACTGTCTCATCATACCAAATTACCGACGAGTATGATGCATATCTAGACATGGTGGACGGGTCGGAGAGTTGCCTGGACCGAGCGACATTCAATCCGTCAAAACTCAGGAGCTACCCGAAACAGCACGCTTACCACGCGCCCTCCATCAGAAGCGCTGTACCGTCCCCATTCCAGAACACACTACAGAATGTACTGGCAGCAGCCACGAAAAGAAACTGCAACGTCACACAGATGAGGGAATTACCCACTTTGGACTCAGCAGTATTCAACGTGGAGTGTTTCAAAAAATTCGCATGCAACCAAGAATACTGGGAAGAATTTGCTGCCAGCCCTATTAGGATAACAACTGAGAATTTAGCAACCTATGTTACTAAACTAAAAGGGCCAAAAGCAGCAGCGCTATTCGCAAAAACCCATAATCTACTGCCACTACAGGAAGTACCAATGGATAGGTTCACAGTAGATATGAAAAGGGACGTAAAGGTGACTCCTGGTACAAAGCATACAGAGGAAAGACCTAAGGTGCAGGTTATACAGGCGGCTGAACCCTTGGCGACAGCATACCTATGTGGGATTCACAGAGAGCTGGTTAGGAGGCTGAACGCCGTCCTCCTACCCAATGTACATACACTATTTGACATGTCTGCCGAGGATTTCGATGCCATCATAGCCGCACACTTTAAGCCAGGAGACACTGTTTTGGAAACGGACATAGCCTCCTTTGATAAGAGCCAAGATGATTCACTTGCGCTTACTGCTTTGATGCTGTTAGAGGATTTAGGGGTGGATCACTCCCTGCTGGACTTGATAGAGGCTGCTTTCGGAGAGATTTCCAGCTGTCACCTACCGACAGGTACGCGCTTCAAGTTCGGCGCCATGATGAAATCAGGTATGTTCCTAACTCTGTTCGTCAACACATTGTTAAACATCACCATCGCCAGCCGAGTGCTGGAAGATCGTCTGACAAAATCCGCGTGCGCGGCCTTCATCGGCGACGACAACATAATACATGGAGTCGTCTCCGATGAATTGATGGCAGCCAGATGTGCCACTTGGATGAACATGGAAGTGAAGATCATAGATGCAGTTGTATCCTTGAAAGCCCCTTACTTTTGTGGAGGGTTTATACTGCACGATACTGTGACAGGAACAGCTTGCAGAGTGGCAGACCCGCTAAAAAGGCTTTTTAAACTGGGCAAACCGCTAGCGGCAGGTGACGAACAAGATGAAGATAGAAGACGAGCGCTGGCTGACGAAGTGATCAGATGGCAACGAACAGGGCTAATTGATGAGCTGGAGAAAGCGGTATACTCTAGGTACGAAGTGCAGGGTATATCAGTTGTGGTAATGTCCATGGCCACCTTTGCAAGCTCCAGATCCAACTTCGAGAAGCTCAGAGGACCCGTCATAACTTTGTACGGCGGTCCTAAATAGGTACGCACTACAGCTACCTATTTTGCAGAAGCCGACAGCAAGTATCTAAACACTAATCAGCTACAATGGAGTTCATCCCAACCCAAACTTTTTACAATAGGAGGTACCAGCCTCGACCCTGGACTCCGCGCTCTACTATCCAAATCATCAGGCCCAGACCGCGCCCTCAGAGGCAAGCTGGGCAACTTGCCCAGCTGATCTCAGCAGTTAATAAACTGACAATGCGCGCGGTACCCCAACAGAAGCCACGCAGGAATCGGAAGAATAAGAAGCAAAAGCAAAAACAACAGGCGCCACAAAACAACACAAATCAAAAGAAGCAGCCACCTAAAAAGAAACCGGCTCAAAAGAAAAAGAAGCCGGGCCGCAGAGAGAGGATGTGCATGAAAATCGAAAATGATTGTATTTTCGAAGTCAAGCACGAAGGTAAGGTAACAGGTTACGCGTGCCTGGTGGGGGACAAAGTAATGAAACCAGCACACGTAAAGGGGACCATCGATAACGCGGACCTGGCCAAACTGGCCTTTAAGCGGTCATCTAAGTATGACCTTGAATGCGCGCAGATACCCGTGCACATGAAGTCCGACGCTTCGAAGTTCACCCATGAGAAACCGGAGGGGTACTACAACTGGCACCACGGAGCAGTACAGTACTCAGGAGGCCGGTTCACCATCCCTACAGGTGCTGGCAAACCAGGGGACAGCGGCAGACCGATCTTCGACAACAAGGGACGCGTGGTGGCCATAGTCTTAGGAGGAGCTAATGAAGGAGCCCGTACAGCCCTCTCGGTGGTGACCTGGAATAAAGACATTGTCACTAAAATCACCCCCGAGGGGGCCGAAGAGTGGAGTCTTGCCATCCCAGTTATGTGCCTGTTGGCAAACACCACGTTCCCCTGCTCCCAGCCCCCTTGCACGCCCTGCTGCTACGAAAAGGAACCGGAGGAAACCCTACGCATGCTTGAGGACAACGTCATGAGACCTGGGTACTATCAGCTGCTACAAGCATCCTTAACATGTTCTCCCCACCGCCAGCGACGCAGCACCAAGGACAACTTCAATGTCTATAAAGCCACAAGACCATACTTAGCTCACTGTCCCGACTGTGGAGAAGGGCACTCGTGCCATAGTCCCGTAGCACTAGAACGCATCAGAAATGAAGCGACAGACGGGACGCTGAAAATCCAGGTCTCCTTGCAAATCGGAATAAAGACGGATGACAGCCACGATTGGACCAAGCTGCGTTATATGGACAACCACATGCCAGCAGACGCAGAGAGGGCGGGGCTATTTGTAAGAACATCAGCACCGTGTACGATTACTGGAACAATGGGACACTTCATCCTGGCCCGATGTCCAAAAGGGGAAACTCTGACGGTGGGATTCACTGACAGTAGGAAGATTAGTCATTCATGTACGCACCCATTTCACCACGACCCTCCTGTGATAGGTCGGGAAAAATTCCATTCCCGACCGCAGCACGGTAAAGAGCTACCTTGCAGCACGTACGTGCAGAGCACCGCCGCAACTACCGAGGAGATAGAGGTACACATGCCCCCAGACACCCCTGATCGCACATTAATGTCACAACAGTCCGGCAACGTAAAGATCACAGTCAATGGCCAGACGGTGCGGTACAAGTGTAATTGCGGTGGCTCAAATGAAGGACTAACAACTACAGACAAAGTGATTAATAACTGCAAGGTTGATCAATGTCATGCCGCGGTCACCAATCACAAAAAGTGGCAGTATAACTCCCCTCTGGTCCCGCGTAATGCTGAACTTGGGGACCGAAAAGGAAAAATTCACATCCCGTTTCCGCTGGCAAATGTAACATGCAGGGTGCCTAAAGCAAGGAACCCCACCGTGACGTACGGGAAAAACCAAGTCATCATGCTACTGTATCCTGACCACCCAACACTCCTGTCCTACCGGAATATGGGAGAAGAACCAAACTATCAAGAAGAGTGGGTGATGCATAAGAAGGAAGTCGTGCTAACCGTGCCGACTGAAGGGCTCGAGGTCACGTGGGGCAACAACGAGCCGTATAAGTATTGGCCGCAGTTATCTACAAACGGTACAGCCCATGGCCACCCGCATGAGATAATTCTGTATTATTATGAGCTGTACCCTACTATGACTGTAGTAGTTGTGTCAGTGGCCACGTTCATACTCCTGTCGATGGTGGGTATGGCAGCGGGGATGTGCATGTGTGCACGACGCAGATGCATCACACCGTATGAACTGACACCAGGAGCTACCGTCCCTTTCCTGCTTAGCCTAATATGCTGCATCAGAACAGCTAAAGCGGCCACATACCAAGAGGCTGCGATATACCTGTGGAACGAGCAGCAACCTTTGTTTTGGCTACAAGCCCTTATTCCGCTGGCAGCCCTGATTGTTCTATGCAACTGTCTGAGACTCTTACCATGCTGCTGTAAAACGTTGGCTTTTTTAGCCGTAATGAGCGTCGGTGCCCACACTGTGAGCGCGTACGAACACGTAACAGTGATCCCGAACACGGTGGGAGTACCGTATAAGACTCTAGTCAATAGACCTGGCTACAGCCCCATGGTATTGGAGATGGAACTACTGTCAGTCACTTTGGAGCCAACACTATCGCTTGATTACATCACGTGCGAGTACAAAACCGTCATCCCGTCTCCGTACGTGAAGTGCTGCGGTACAGCAGAGTGCAAGGACAAAAACCTACCTGACTACAGCTGTAAGGTCTTCACCGGCGTCTACCCATTTATGTGGGGCGGCGCCTACTGCTTCTGCGACGCTGAAAACACGCAGTTGAGCGAAGCACACGTGGAGAAGTCCGAATCATGCAAAACAGAATTTGCATCAGCATACAGGGCTCATACCGCATCTGCATCAGCTAAGCTCCGCGTCCTTTACCAAGGAAATAACATCACTGTAACTGCCTATGCAAACGGCGACCATGCCGTCACAGTTAAGGACGCCAAATTCATTGTGGGGCCAATGTCTTCAGCCTGGACACCTTTCGACAACAAAATTGTGGTGTACAAAGGTGACGTCTATAACATGGACTACCCGCCCTTTGGCGCAGGAAGACCAGGACAATTTGGCGATATCCAAAGTCGCACACCTGAGAGTAAAGACGTCTATGCTAATACACAACTGGTACTGCAGAGACCGGCTGCGGGTACGGTACACGTGCCATACTCTCAGGCACCATCTGGCTTTAAGTATTGGCTAAAAGAACGCGGGGCGTCACTGCAGCACACAGCACCATTTGGCTGCCAAATAGCAACAAACCCGGTAAGAGCGGTGAATTGCGCCGTAGGGAACATGCCCATCTCCATCGACATACCGGAAGCGGCCTTCACTAGGGTCGTCGACGCGCCCTCTTTAACGGACATGTCGTGCGAGGTACCAGCCTGCACCCATTCCTCAGACTTTGGGGGCGTCGCCATTATTAAATATGCAGCCAGCAAGAAAGGCAAGTGTGCGGTGCATTCGATGACTAACGCCGTCACTATTCGGGAAGCTGAGATAGAAGTTGAAGGGAATTCTCAGCTGCAAATCTCTTTCTCGACGGCCTTAGCCAGCGCCGAATTCCGCGTACAAGTCTGTTCTACACAAGTACACTGTGCAGCTGAGTGCCACCCCCCGAAGGACCACATAGTCAACTACCCGGCGTCACATACCACCCTCGGGGTCCAGGACATCTCCGCTACGGCGATGTCATGGGTGCAGAAGATCACGGGAGGTGTGGGACTGGTTGTTGCTGTTGCCGCACTGATTCTAATCGTGGTGCTATGCGTGTCGTTCAGCAGGCACTAACTTGACAATTAAGTATGAAGGTATATGTGTCCCCTAAGAGACACACTGTACATAGCAAATAATCTATAGATCAAAGGGCTACGCAACCCCTGAATAGTAACAAAATACAAAATCACTAAAAATTATAAAAACAGAAAAATACATAAATAGGTATACGTGTCCCCTAAGAGACACATTGTATGTAGGTGATAAGTATAGATCAAAGGGCCGAATAACCCCTGAATAGTAACAAAATATGAAAATCAATAAAAATCATAAAATAGAAAAACCATAAACAGAAGTAGTTCAAAGGGCTATAAAACCCCTGAATAGTAACAAAACATAAAATTAATAAAAATCAAATGAATACCATAATTGGCAAACGGAAGAGATGTAGGTACTTAAGCTTCCTAAAAGCAGCCGAACTCACTTTGAGAAGTAGGCATAGCATACCGAACTCTTCCACGATTCTCCGAACCCACAGGGACGTAGGAGATGTTATTTTGTTTTTAATATTTC

>GQ428210|2006|India, Group 4
[truncated: 852,496 more chars]
