# Supplementary material for: Development of a single-tube one-step RT-LAMP assay to detect the Chikungunya virus genome
Source: PLoS Negl Trop Dis. 2018 May 29;12(5):e0006448. doi: 10.1371/journal.pntd.0006448 (PMC5973553; doi:10.1371/journal.pntd.0006448)
Supplement: S1 Table — (DOCX) [file pntd.0006448.s003.docx]

**S1 Table. RNA samples collected from the CHIKV outbreak and analysed in this study.**

| IPD number | Initial C_T_ values | Current C_T_ values | T_T_ values (min) |
| --- | --- | --- | --- |
| 274438 | 27.51 | 29.28 | 18-19 |
| 274530 | 23.22 | 33.22 | 16-17 |
| 274754 | 24.07 | 25.13 | 16-17 |
| 274755 | 23.87 | 25.30 | 16-17 |
| 274836 | 23.34 | 26.87 | 15-16 |
| 274455 | 33.68 | 35.77 | 22-27 |
| 274507 | 24.52 | 25.25 | 16-17 |
| 274524 | 23.45 | 24.92 | 16-17 |
| 274523 | 25.07 | 33.08 | 17-18 |
| 274665 | 26.10 | 24.82 | 15-18 |
| 274687 | 25.24 | 26.66 | 16 |
| 274647 | 25.14 | 27.65 | 17-18 |
| 274646 | 23.65 | 25.36 | 15-16 |
| 274642 | 24.38 | 26.02 | 17-18 |
| 274382 | 30.38 | 27.71 | 18 |
| 274496 | 27.93 | 28.87 | 19-20 |
| 274426 | 34.91 | 30.59 | 18-19 |
| 265125 | 30.46 | 25.18 | 17-18 |
| 265119 | 36.93 | 26.51 | 17-18 |
| 277599 | 25.62 | Negative | Negative |
| 277530 | 25.08 | Negative | Negative |
| 274843 | 27.33 | Negative | Negative |
| 264781 | Negative | Negative | Negative |
| 264842 | Negative | Negative | 29-42 |
| 274461 | 24.06 | Negative | 16-17 |
| 274688 | 29.45 | Negative | 15-16 |
| 274464 | 27.91 | Negative | 18-19 |
| 277604 | 29.00 | Negative | 35-55 |
| 277586 | 38.10 | Negative | 55 (2/3 negative) |
| 264998 | 30.01 | Negative | 43-52 (1/3 negative) |
| 274443 | 32.81 | Negative | 30-35 (1/3 negative) |
| 277593 | 31.06 | Negative | 35-42 (1/3 negative) |
| 277551 | 39.00 | Negative | 45 (2/3 negative) |
| 277545 | 27.29 | Negative | 32 (2/3 negative) |
| 264779 | 29.60 | Negative | 50 (2/3 negative) |

T_T_ values represent the range when the 3 replicates were detected. Between brackets there is information about negative results obtained within the 3 replicates.
